# Bioisosterism-driven design of orally active, safe, and broad-spectrum biphenyl-DAPY derivatives as highly potent HIV-1 non-nucleoside reverse transcriptase inhibitors

**DOI:** 10.1016/j.apsb.2025.06.016

**Published:** 2025-06-24

**Authors:** Xiao-Mei Chen, Qing-Qing Hao, Christophe Pannecouque, Erik De Clercq, Shuai Wang, Fen-Er Chen

**Affiliations:** a Sichuan Research Center for Drug Precision Industrial Technology, West China School of Pharmacy, Sichuan University, Chengdu 610041, China; b Institute of Flow Chemistry and Engineering, School of Chemistry and Materials, Jiangxi Normal University, Nanchang 330022, China; c Engineering Center of Catalysis and Synthesis for Chiral Molecules, Department of Chemistry, Fudan University, Shanghai 200433, China; d Shanghai Engineering Center of Industrial Asymmetric Catalysis for Chiral Drugs, Shanghai 200433, China; e Rega Institute for Medical Research, KU Leuven, Herestraat 49, Leuven B-3000, Belgium

**Keywords:** AIDS, HIV-1, NNRTIs, Diarylpyrimidines, Bioisosterism, Anti-resistance potency, Safety, Oral bioavailability

## Abstract

This study aimed to identify ideal pharmaceutical candidates featuring strong anti-HIV-1 activity and desirable drug-like characteristics. Our endeavor involved the implementation of a bioisosterism strategy, leading to the discovery of an assemblage of halogen-containing biphenyl-diarylpyrimidines as potent HIV-1 non-nucleoside reverse transcriptase inhibitors. Notably, compound **A12** demonstrated exceptional efficacy against both WT HIV-1 (EC_50_ = 1.9 nmol/L) and seven mutant strains (EC_50_ = 1.7–157 nmol/L), surpassing that of the lead compound **6** and comparable to etravirine. Furthermore, this analog exhibited minimal adverse effects with significantly reduced cytotoxicity (CC_50_ = 195 μmol/L) and a high selectivity index (SI = 102,608), superior to those of etravirine (CC_50_ > 4.6 μmol/L, SI > 1436) and rilpivirine (CC_50_ = 3.98 μmol/L, SI = 3989). It displayed low inhibition of CYP (IC_50_ = 6.99–25 μmol/L) and hERG (IC_50_ > 40 μmol/L), indicating a safer profile compared to etravirine and rilpivirine. No acute toxicity or organ pathological damage was observed at a single dose of 2 g/kg. Additionally, **A12** exhibited favorable oral bioavailability (*F* = 29.2%) and an extended elimination half-life (*T*_1/2_ = 13.56 h), enabling convenient oral administration at minimal doses. These findings indicated that **A12** could serve as a promising drug candidate for HIV treatment.

## Graphical abstract

This study reported the discovery of a highly potent NNRTI **A12** featuring broad-spectrum anti-HIV-1 activities and favorable drug-like profiles through bioisosterism-based drug design strategy.

**Figure:**
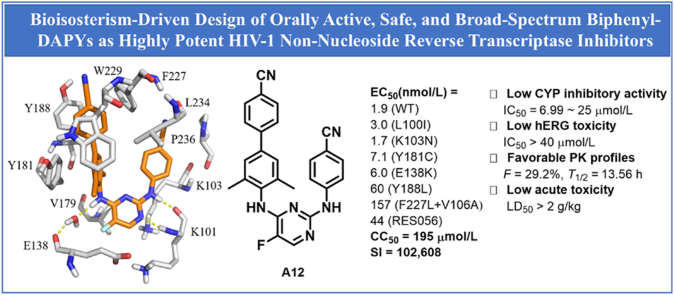

## 1 Introduction

**Figure 1:**
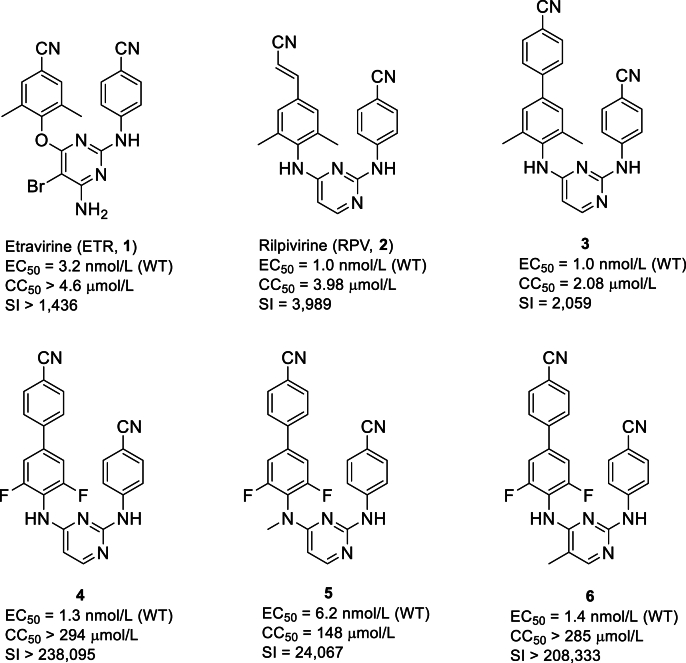
Structures of representative DAPYs.

Reverse transcriptase (RT) is an attractive target for treating acquired immunodeficiency syndrome (AIDS), which is responsible for converting viral RNA into DNA^1^. Non-nucleoside reverse transcriptase inhibitors (NNRTIs) noncompetitively bind to the allosteric site (NNRTI-binding pocket, NNIBP) of RT, change the conformation of the catalytic domain, and then suppress the replication of HIV-1^2^^,^^3^. On account of distinctive antiviral properties and low toxicity, NNRTIs are extensively utilized in antiretroviral therapy for managing HIV infection in clinic^4^, ^5^, ^6^. Diarylpyrimidine (DAPY) derivatives, represented by FDA-approved etravirine (ETR, **1**) and rilpivirine (RPV, **2**), are the most promising category of NNRTIs (Fig. 1)^7^. These DAPYs typically exhibit robust efficacy against wild-type (WT) HIV-1 and certain mutant strains owing to their inherent structural flexibility, which promotes them to better adapt to conformational alterations within NNIBP induced by genetic mutations^8^^,^^9^. However, several associated challenges, including the rapid emergence of drug resistance, undesired safety, and pharmacokinetic (PK) profiles, remain unresolved^10^. For example, both ETR and RPV exhibit a CC_50_ value of ∼5 μmol/L and have severe adverse effects like hypersensitivity reactions and hepatotoxicity^11^^,^^12^. They can also induce serious drug–drug interactions as their inhibitory or inductive effects on CYP2C9, CYP2C19, and CYP3A4^13^. Moreover, RPV has been documented as a potent hERG blocker with a high affinity (IC_50_ = 0.50 μmol/L)^14^. Besides, ETR and RPV suffer from poor oral bioavailability^15^.

In recent years, our medicinal research group has diligently endeavored to discover novel DAPY NNRTIs with optimal anti-HIV-1 potency and desirable drug-like properties^16^. Targeting the optimization of the left wing, several series of DAPYs containing conjugated fragments, such as quinolone, naphthyl, and biphenyl, were identified^17^, ^18^, ^19^. Among them, biphenyl-DAPYs demonstrate the highest activity. Initially, compound **3** was discovered by substituting the phenyl ring in ETR or RPV with a biphenyl moiety, which possesses enhanced *π*–*π* interactions with residues, *i.e.*, Y181, Y188, F227, and W229, in NNIBP. This resultant compound displayed excellent antiviral activity with EC_50_ values of 1.0 nmol/L and 0.84–110 nmol/L against WT HIV-1 and the clinically drug-resistant variants, respectively. However, the high cytotoxicity, low selectivity, and undesirable metabolic stability hinder its further evaluation^19^. Subsequently, the dimethyl moiety of compound **3** was replaced with fluorine to block the metabolic site^20^. This modification is attributed to the predominant mono- and dimethyl-hydroxylation metabolism of ETR or RPV, which results in suboptimal safety profiles. As anticipated, the inclusion of a 3,5-difluoro moiety in compound **4** improved the safety profiles. However, this modification decreased its efficacy against double mutants F227L + V106A and K103N + Y181C (RES056). Further alkylation of the left linker of compound **4** significantly reduced the efficacy of compound **5**. Successful transfer of the methyl group to the C-5 position of the pyrimidine yielded compound **6**, which exhibited strong potency (EC_50_ = 1.4 nmol/L) and minimal cytotoxicity (CC_50_ > 285 μmol/L, SI > 208,333). However, it was unresponsive to mutants Y188L, F227L + V106A, and RES056 (EC_50_ > 2 μmol/L) and displayed suboptimal oral bioavailability (*F* = 2.39%)^21^. Hence, establishing a meticulous balance between inhibitory effectiveness and drug-like attributes of DAPY-derived NNRTIs is necessary to discern the most suitable pharmaceutical agents for combating HIV-1.

**Figure 2:**
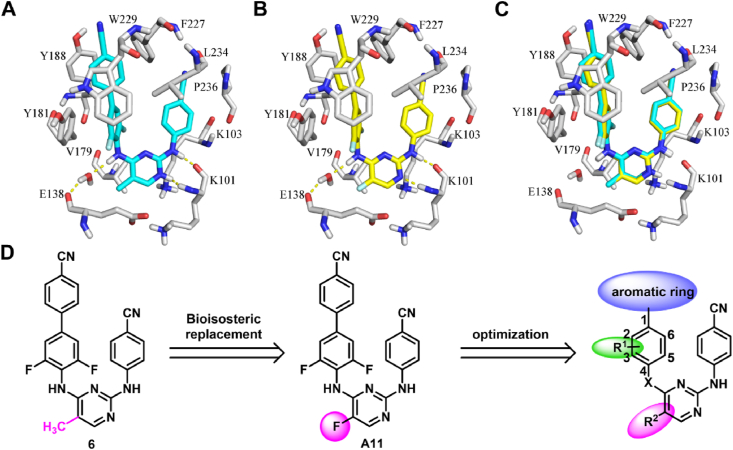
Rendering of **6** (A) and **A11** (B) with WT HIV-1 RT (PDB code: 2ZD1); (C) Overlay of **6** and **A11**; (D) Halogen-containing biphenyl-DAPYs designed by bioisosterism-driven strategy.

Halogen atoms, especially fluorine and chlorine, are extensively employed in the field of drug design^22^, ^23^, ^24^, ^25^. Specifically, fluorination has been widely recognized as a potential strategy for improving metabolic stability, bioavailability, and safety of molecules^26^, ^27^, ^28^, ^29^. Moreover, the ability of fluorine to form hydrogen bonding or electrostatic interactions can reinforce protein-ligand binding affinity^30^^,^^31^. Chlorine can engage in halogen bonding, and according to previous studies, the phenomenon of the “magic chloro effect” contributes to significant enhancements in drug potency and profound effects on PK profiles^32^. To the best of our knowledge, the characteristics of halogenated biphenyl-DAPYs remain hitherto unexplored. Therefore, in the present study, we substituted the methyl moiety located at the C-5 position of the pyrimidine ring of compound **6** with a halogen substituent (fluorine or chlorine) *via* a bioisosterism-driven drug design approach, aiming to develop potential candidates with desirable anti-HIV-1 activity and drug-like properties. Molecular modeling results indicate that the designed compound **A11** exhibits a conformation reminiscent of a “U” shape within NNIBP of RT, akin to compound **6** (Fig. 2A‒C). The biphenyl fragment engages in face-to-face *π*–*π* stacking interactions with Y181 and Y188. Hydrogen bonds generated between the ligand and K101 or E138 remain intact. The introduced 5-F partially occupies the entrance channel and possesses hydrophobic interaction with V179. Given the significance and practicality of the aforementioned assumptions, a collection of innovative halogen-containing biphenyl-DAPYs was synthesized, and their anti-HIV-1 efficacies were evaluated (Fig. 2D).

## 2 Results and discussion

### 2.1 Chemistry

**Scheme 1:**
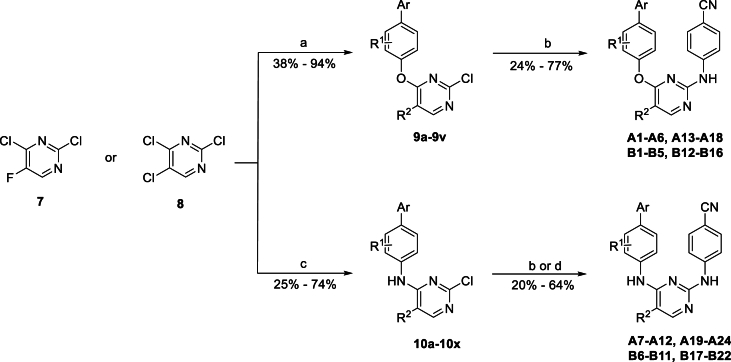
Reagents and conditions: (a) Appropriate 4′-hydroxy[1,1′-biphenyl]-4-carbonitriles or 4-(4-pyridinyl)phenol, K_2_CO_3_, dry DMF, r.t., 2–4 h; (b) 4-cyanoaniline, Pd(OAc)_2_, BINAP, Cs_2_CO_3_, dry 1,4-dioxane, N_2_, 110 °C, 2–4 h; (c) Appropriate 4′-amino-[1,1′-biphenyl]-4-carbonitrile or 4-(4-pyridinyl)benzenamine, Pd(OAc)_2_, Xantphos, Cs_2_CO_3_, dry 1,4-dioxane, N_2_, 110 °C, 1–2 h; (d) 4-cyanoaniline, 12 mol/L HCl, *n*-BuOH, 100 °C, 5–8 h.

The synthetic routes of the target DAPYs, *viz*. **A1**–**A24** and **B1**–**B22** are depicted in Scheme 1. Nucleophilic substitution of commercially available 2,4-dichloro-5-fluoropyrimidine **7** or 2,4,5-trichloropyrimidine **8** with appropriate 4′-hydroxy[1,1′-biphenyl]-4-carbonitriles or 4-(4-pyridinyl)phenol was conducted in the presence of K_2_CO_3_ in dry DMF to obtain monoarylpyrimidines **9a**–**9v** with 38%–94% yields. These monoarylpyrimidines were further subjected to the Buchwald–Hartwig reaction, catalyzed by Pd(OAc)_2_, at 110 °C to deliver the expected DAPYs, *viz*. **A1**–**A6**, **A13**–**A18**, **B1**–**B5**, and **B12**–**B16** with yields in the range of 24%–77%^33^^,^^34^. Moreover, the Pd-catalyzed coupling reaction of **7** or **8** with appropriate 4′-amino-[1,1′-biphenyl]-4-carbonitrile or 4-(4-pyridinyl)benzenamine yielded monoarylpyrimidines **10a**–**10x** in 25%–74% yields. Subsequently, **10a**–**10r** were reacted with 4-cyanoaniline under an acidic condition to derive **A7**–**A12**, **A19**–**A24**, and **B6**–**B11** with 20%–64% yields. The Pd-catalyzed Buchwald–Hartwig reaction between **10s**–**10x** and 4-cyanoaniline also yielded 31%–63% of **B17**–**B22**.

### 2.2 Anti-HIV-1 activity and cytotoxicity

The anti-HIV-1 activity and cytotoxicity of these novel biphenyl-DAPYs were evaluated in MT-4 cells, and NVP, EFV, ETR were tested for reference. As shown in Table 1, all the newly obtained derivatives **A1**–**A24** and **B1**–**B22**, display effective potency against WT HIV-1 strain, with EC_50_ values in the range of 1.1–536 nmol/L, and most of them have low cytotoxicity (CC_50_ > 45 μmol/L). Notably, compounds **A7**, **A9**, **A11**, **A12**, **A24**, **B10**, and **B21** exhibit an exceptional inhibitory activity (EC_50_ = 1.1–2.0 nmol/L), which is equivalent to or slightly higher than that of ETR (EC_50_ = 3.2 nmol/L). Among them, **A9**, **A12**, **A24**, and **B10** show drastically decreased cytotoxicity (CC_50_ > 166 μmol/L) and significantly increased selectivity index (SI ≥ 102,608), thereby outperforming ETR. The detailed SAR analysis is outlined below: the activity of the fluorine-substituted biphenyl-DAPYs **A1**–**A24** does not show a general dependence on the steric and electron effects of the substituents. For instance, although the compounds **A1–A4** exhibit significantly improved electron donating ability and steric hindrance (F < Cl < Me < OMe), their potency follows a pattern of **A4** > **A1** > **A3** > **A2**. Subsequently, the linker moiety that bridges pyrimidine and the left wing changes from oxygen (O) to nitrogen (NH), exhibiting a slight rise in activity (except for **A2**, **A4**, **A13**, and **A16**). This trend is exemplified by the contrasting potency of the monosubstituted compounds **A1** (EC_50_ = 4.2 nmol/L) and **A7** (EC_50_ = 1.8 nmol/L) as well as the disubstituted analogs **A6** (EC_50_ = 7.1 nmol/L) and **A12** (EC_50_ = 1.9 nmol/L). Inspired by our previous research on heteroaromatic-biphenyl-DAPYs^15^, the 4-cyanophenyl group on the biphenyl moiety was replaced with a privileged 4-pyridyl group for further evaluation. Results of pairwise comparisons between **A1**–**A12** and **A13**–**A24** reveal that the compounds containing a 4-pyridinyl substituent exhibit a sustained activity at a low nanomolar range (**A13**–**A24**, EC_50_ = 1.3–56 nmol/L), although the activity is marginally lower than that of their counterparts, which contain a 4-cyanophenyl substituent; exceptions are observed in the **A2** *versus* **A14**, **A8** *versus* **A20**, **A10** *versus* **A22**, and **A12** *versus* **A24** results. The halogen substituent at the pyrimidine changes from 5-F to 5-Cl, showing a discernible decrease in activity. The efficacy of **A7**–**A12**, as evidenced by their EC_50_ values of 1.8, 43, 2.0, 23, 1.1, and 1.9 nmol/L, is higher than that of the corresponding analogs **B6**–**B11** (EC_50_ = 12, 46, 8.2, 104, 1.9, 5.3 nmol/L). This trend is observed in the majority of the other analogs as well. The detailed SARs of **B1**–**B22** are mostly similar to that of **A1**–**A24**. However, the inhibitory activity decreases when the linker O is replaced with NH, except in the case of **B5** *versus* **B11**, **B14** *versus* **B19**, and **B16** *versus* **B22**; this result is in contrast to that of the fluorine-substituted biphenyl-DAPYs. Furthermore, the incorporation of disubstituted groups into the compounds with an NH linker appears to be more favorable. For instance, **B21** and **B22** exhibit a remarkable efficacy, with EC_50_ values of 2.0, 8.9 nmol/L, which is 71- and 5-fold higher than the potency of the corresponding compounds **B17** and **B19** (EC_50_ = 142, 44 nmol/L). A similar trend is observed in **A11**–**A12**, **A23**–**A24**, **B10**–**B11**, and their respective monosubstituted compounds.

**Table 1:**
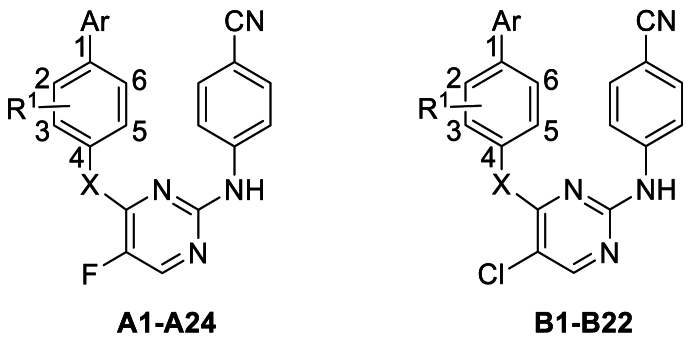
Anti-HIV-1(WT) activity of **A1**–**A24** and **B1**–**B22**.

Compd.	R^1^	X	Ar	EC_50_ (nmol/L)^a^	CC_50_ (μmol/L)^b^	SI (III_B_)^c^
				HIV-1 (III_B_)		
**A1**	3-F	O	4-Cyanophenyl	4.2 ± 1.4	>47	>10,870
**A2**	3-Cl	O	4-Cyanophenyl	27 ± 25	>45	>1670
**A3**	3-Me	O	4-Cyanophenyl	7.6 ± 2.6	>48	>6349
**A4**	3-OMe	O	4-Cyanophenyl	3.0 ± 1.1	>46	>16,000
**A5**	3,5-F_2_	O	4-Cyanophenyl	6.3 ± 3.2	>45	>7143
**A6**	3,5-Me_2_	O	4-Cyanophenyl	7.1 ± 3.2	219 ± 12	30,805
**A7**	3-F	NH	4-Cyanophenyl	1.8 ± 0.9	>47	>25,806
**A8**	3-Cl	NH	4-Cyanophenyl	43 ± 23	>45	>1038
**A9**	3-Me	NH	4-Cyanophenyl	2.0 ± 0.4	288 ± 9	146,974
**A10**	3-OMe	NH	4-Cyanophenyl	23 ± 18	>46	>1961
**A11**	3,5-F_2_	NH	4-Cyanophenyl	1.1 ± 0.2	>45	>40,000
**A12**	3,5-Me_2_	NH	4-Cyanophenyl	1.9 ± 0.4	195 ± 58	102,608
**A13**	3-F	O	4-Pyridyl	9.7 ± 3.7	>50	>5147
**A14**	3-Cl	O	4-Pyridyl	12 ± 3.1	>48	>3846
**A15**	3-Me	O	4-Pyridyl	11 ± 2.5	>50	>4734
**A16**	3-OMe	O	4-Pyridyl	3.1 ± 2.2	>48	>15,094
**A17**	3,5-F_2_	O	4-Pyridyl	11 ± 4.1	>48	>4396
**A18**	3,5-Me_2_	O	4-Pyridyl	56 ± 44	>49	>868
**A19**	3-F	NH	4-Pyridyl	18 ± 14	>50	>2765
**A20**	3-Cl	NH	4-Pyridyl	10 ± 1.7	>48	>4819
**A21**	3-Me	NH	4-Pyridyl	6.1 ± 1.3	>51	>8421
**A22**	3-OMe	NH	4-Pyridyl	8.0 ± 2.7	>49	>6154
**A23**	3,5-F_2_	NH	4-Pyridyl	7.6 ± 1.2	21 ± 18	>2753
**A24**	3,5-Me_2_	NH	4-Pyridyl	1.3 ± 0.2	166 ± 44	129,523
**B1**	3-F	O	4-Cyanophenyl	8.4 ± 3.4	39 ± 12	4572
**B2**	3-Cl	O	4-Cyanophenyl	8.7 ± 2.0	35 ± 7.3	3949
**B3**	3-Me	O	4-Cyanophenyl	7.1 ± 1.6	>46	>6542
**B4**	3-OMe	O	4-Cyanophenyl	26 ± 13	11 ± 3.7	412
**B5**	3,5-Me_2_	O	4-Cyanophenyl	11 ± 4.2	39 ± 9.1	3478
**B6**	3-F	NH	4-Cyanophenyl	12 ± 2.7	>45	>3911
**B7**	3-Cl	NH	4-Cyanophenyl	46 ± 13	>273	>6026
**B8**	3-Me	NH	4-Cyanophenyl	8.2 ± 1.6	>46	>5556
**B9**	3-OMe	NH	4-Cyanophenyl	104 ± 68	236 ± 24	2261
**B10**	3,5-F_2_	NH	4-Cyanophenyl	1.9 ± 0.3	200 ± 31	104,732
**B11**	3,5-Me_2_	NH	4-Cyanophenyl	5.3 ± 1.8	247 ± 11	47,357
**B12**	3-F	O	4-Pyridyl	7.7 ± 1.9	>48	>6173
**B13**	3-Cl	O	4-Pyridyl	6.2 ± 1.6	>46	>7477
**B14**	3-Me	O	4-Pyridyl	48 ± 22	>48	>1005
**B15**	3-OMe	O	4-Pyridyl	6.7 ± 2.3	>47	>6897
**B16**	3,5-Me_2_	O	4-Pyridyl	105 ± 51	243 ± 62	2327
**B17**	3-F	NH	4-Pyridyl	142 ± 14	>48	>342
**B18**	3-Cl	NH	4-Pyridyl	83 ± 69	>46	>563
**B19**	3-Me	NH	4-Pyridyl	44 ± 9.7	>48	>1122
**B20**	3-OMe	NH	4-Pyridyl	536 ± 326	164 ± 51	306
**B21**	3,5-F_2_	NH	4-Pyridyl	2.0 ± 0.7	1.3 ± 0.3	680
**B22**	3,5-Me_2_	NH	4-Pyridyl	8.9 ± 0.7	260 ± 6.5	29,634
**6**	–	–	–	1.4 ± 0.3	>285	>208,333
NVP	–	–	–	188 ± 68	>15	>80
EFV	–	–	–	3.8 ± 1.0	>6.3	>1618
ETR	–	–	–	3.2 ± 0.7	>4.6	>1436

a EC_50_: the effective concentration required to protect MT-4 cells against HIV-induced cytopathogenicity by 50%. Data are mean ± SD, n ≥ 3.

b CC_50_: the cytotoxic concentration of compound that reduced the viability of uninfected MT-4 cells by 50%. Data are mean ± SD, n ≥ 3.

c SI: selectivity index, ratio CC_50_/EC_50_.

### 2.3 Antiviral activity of representative compounds against HIV-1 mutants

Motivated by the promising anti-HIV-1 efficacy of these biphenyl-DAPYs, a subset of ten compounds (**A1**, **A4**, **A7**, **A9**, **A11**, **A12**, **A16**, **A24**, **B10**, and **B11**), which demonstrated exceptional activity (EC_50_ ≤ 5.3 nmol/L) and minimal cytotoxicity (CC_50_ > 45 μmol/L), were further assessed to determine their inhibition toward seven clinically relevant NNRTI-resistant variants. Table 2 indicates that most of these derivatives display significant potency against L100I, K103N, Y181C, and E138K. In the case of L100I, compounds **A12**, **A24**, and **B11** demonstrate comparable efficacy to ETR, exhibiting EC_50_ values of 3.0, 4.1, and 5.5 nmol/L, respectively. Apart from **A1** and **A7**, the remaining compounds display double-digit nanomolar inhibitory activities, being equipotent to that of EFV. As for K103N and Y181C, the inhibitory potency of **A9**, **A11**, **A12**, **A24**, and **B11** keeps at a single-digit nanomolar level (EC_50(K103N)_ = 1.7–9.4 nmol/L; EC_50(Y181C)_ = 7.1–9.8 nmol/L), comparable to that of ETR (EC_50(K103N)_ = 2.8 nmol/L, EC_50(Y181C)_ = 13 nmol/L). Moreover, all tested compounds show great efficacy against E138K, with EC_50_ values spanning from 2.5 to 35 nmol/L. For Y188L, F227L + V106A, and K103N + Y181C (RES056), though some of the derivatives were less active or inactive, compounds **A12**, **A24**, and **B11** demonstrate more potent than NVP and EFV in terms of Y188L and RES056, and comparable to EFV toward F227L + V106A. Obviously, compounds containing dimethyl substituents (**A12**, **A24**, **B11**) exhibit markedly enhanced anti-resistance potency, especially toward Y188L and double mutant strains. This effect was also observed in RPV-associated derivatives, which can be attributed to the ability of dimethyl groups to form extensive hydrophobic interactions and limit conformational flexibility^35^. Overall, **A12** was identified as the most promising inhibitor, possessing EC_50_ values of 3.0 nmol/L (L100I), 1.7 nmol/L (K103N), 7.1 nmol/L (Y181C), 6.0 nmol/L (E138K), 60 nmol/L (Y188L), 157 nmol/L (F227L + V106A), and 44 nmol/L (RES056). These values surpass those of the lead compound **6** and are comparable to that of the established agent ETR. Consequently, compound **A12** can be developed as a prospective drug candidate for HIV-1 management and requires further extensive investigations.

**Table 2:** Antiviral activity of the representative DAPYs toward HIV-1 mutants.

Compd.	EC_50_ (nmol/L)^a^						
	L100I	K103N	Y181C	E138K	Y188L	F227L + V106A	K103N + Y181C
**A1**	174 ± 49	52 ± 42	306 ± 117	28 ± 12	>47,015	>47,015	>47,015
**A4**	41 ± 25	9.4 ± 2.7	48 ± 14	7.8 ± 2.3	1372 ± 206	≥1600	>45,721
**A7**	5443 ± 2403	68 ± 38	259 ± 165	35 ± 17	>47,124	>47,124	>47,124
**A9**	17 ± 6.7	7.1 ± 1.9	9.3 ± 0.7	10 ± 1.9	1625 ± 382	37,698 ± 3520	24,926 ± 16,982
**A11**	10 ± 2.9	5.0 ± 1.1	8.1 ± 2.0	2.5 ± 0.7	>45,207	>45,207	>45,207
**A12**	3.0 ± 0.5	1.7 ± 0.5	7.1 ± 1.2	6.0 ± 0.5	60 ± 9.2	157 ± 64	44 ± 18
**A16**	39 ± 9.7	12 ± 3.9	104 ± 17	13 ± 3.4	1524 ± 556	1234 ± 556	>48,378
**A24**	4.1 ± 3.7	1.9 ± 0.5	9.3 ± 5.6	8.3 ± 5.4	90 ± 27	244 ± 146	63 ± 12
**B10**	17 ± 8.1	5.0 ± 2.4	11 ± 1.5	5.7 ± 2.8	187 ± 52	959 ± 327	676 ± 349
**B11**	5.5 ± 1.8	4.4 ± 1.1	9.8 ± 3.3	12 ± 6.9	33 ± 6.7	164 ± 71	102 ± 24
**6**	16 ± 5.3	5.4 ± 1.8	11 ± 1.9	8.3 ± 1.5	>2000	>2000	>2000
NVP	1802 ± 1427	5858 ± 2779	9012 ± 2629	143 ± 38	>15,020	>15,020	>15,020
EFV	24 ± 9.5	60 ± 19	6.3 ± 2.5	5.4 ± 1.6	244 ± 95	181 ± 89	314 ± 146
ETR	4.4 ± 1.6	2.8 ± 0.7	13 ± 2.8	7.6 ± 2.3	18 ± 5.3	12 ± 9.4	44 ± 23

a EC_50_: the effective concentration required to protect MT-4 cells against HIV-induced cytopathogenicity by 50%. Data are mean ± SD, n ≥ 3.

### 2.4 Anti-HIV-1 RT activity

Next, the efficacy of ten representative DAPYs against WT RT was evaluated, with NVP and ETR serving as references. Table 3 indicates that these tested compounds are 22–44 times more active than NVP (IC_50_ = 1202 nmol/L), with IC_50_ values of 27–54 nmol/L, most of which are equivalent to that of ETR (IC_50_ = 30 nmol/L). The results suggest that these biphenyl-DAPYs exhibit a high affinity to HIV-1 RT and can be categorized as NNRTIs.

**Table 3:** Antiviral activity of representative compounds toward WT HIV-1 RT.

Compd.	IC_50_ (nmol/L)^a^	Compd.	IC_50_ (nmol/L)
**A1**	54 ± 9.4	**A16**	34 ± 2.4
**A4**	46 ± 4.6	**A24**	39 ± 2.4
**A7**	28 ± 4.7	**B10**	31 ± 2.2
**A9**	36 ± 4.8	**B11**	53 ± 0.0
**A11**	27 ± 2.3	NVP	1202 ± 413
**A12**	41 ± 6.9	ETR	30 ± 4.6

a IC_50_: inhibitory concentration of test compound required to inhibit WT HIV-1 RT polymerase activity by 50%. Data are mean ± SD, *n* ≥ 3.

### 2.5 Molecular docking of **A12** with HIV-1 RT

**Figure 3:**
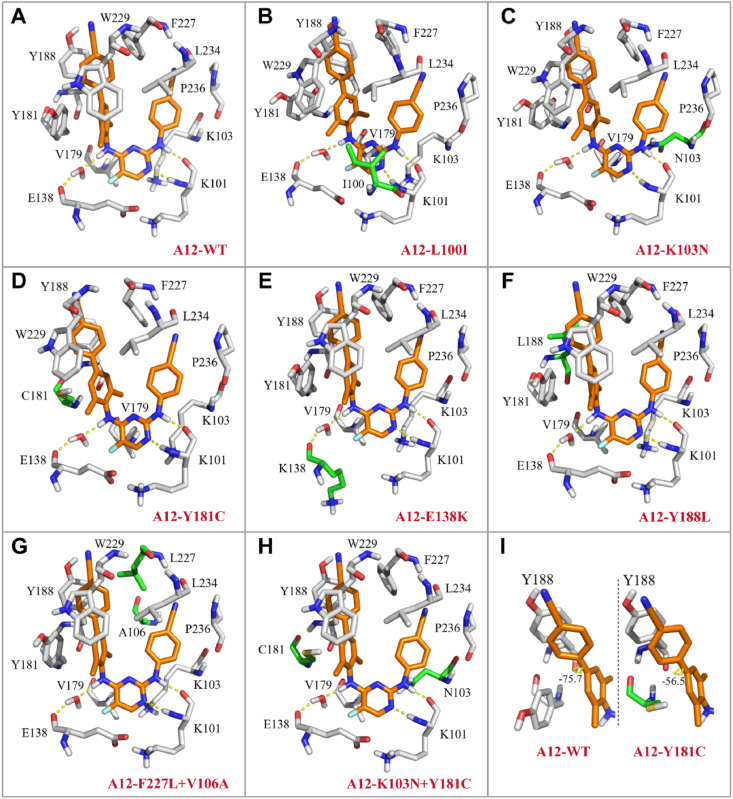
Simulated docking complexes of **A12** with WT and mutant HIV-1 RTs (PDB code: 2ZD1). (A) WT with **A12**; (B) L100I with **A12**; (C) K103N with **A12**; (D) Y181C with **A12**; (E) E138K with **A12**; (F) Y188L with **A12**; (G) F227L + V106A with **A12**; (H) K103N + Y181C with **A12**; (I) The interphenyl dihedral angle of **A12** in WT and Y181C. Mutated residues are visually represented by green sticks.

Molecular docking was conducted to evaluate the binding pattern of **A12** in NNIBP and elucidate its significant anti-resistance profiles. Fig. 3A illustrates that **A12** displays a typical “U” shape conformation, resembling the prevalent conformation observed in DAPY NNRTIs. The biphenyl fragment is effectively embedded into the hydrophobic tunnel, establishing robust *π*–*π* interactions with the adjacent residues Y181, Y188, F227, and W229. The 5-F substituent at the pyrimidine moiety is well positioned in the entrance channel, forming hydrophobic interactions with residue V179. Water-mediated hydrogen bonding involving the NH linker at the left wing and carbonyl group in E138 was observed. The N atom on the pyrimidine and the right linker NH form two hydrogen bonds with K101, which are crucial for retaining anti-HIV-1 activity. The observed outcomes collectively provide a plausible explanation for the robust efficacy of **A12** against WT HIV-1. Furthermore, **A12** exhibits commendable positional adaptability in the binding pockets of L100I, K103N, Y181C, E138K, Y188L, F227L + V106A, and K103N + Y181C (RES056) mutants (Fig. 3B–H). Similar to binding in the WT HIV-1 RT, **A12** displays a binding conformation featuring a “U” shape. Moreover, the mutant RTs retain several distinctive interactions, including hydrogen bonding and *π*–*π* interactions. The remarkable antiviral efficacy could be ascribed to the insensitivity of the binding affinity between **A12** and the NNIBP to the mutation of the residues L100I, K103N, and E138K. In Y181C, the interphenyl dihedral angle of the biphenyl moiety changed from −75.7° to −56.5° (Fig. 3I), which might contribute to strengthening its *π–π* interactions with Y188 and partially compensating for the diminished *π*–*π* interactions with Y181. Similarly, the modification of the interphenyl dihedral angle (from −75.7° to −53°) in RES056 possibly counterbalanced the disrupted interactions induced by Y181C. However, when combined with K103N, the hydrophobic interactions, particularly the *π*–alkyl interactions between K103 and **A12**, might attenuate due to the shortening of the alkyl chain of K103. Hence, the efficacy of **A12** against Y181C was 6.2-fold higher than that toward RES056. The slightly diminished potency of **A12** can be attributed to the absence of *π*–*π* interactions with Y188 or F227 in terms of Y188L and F227L + V106A mutations. The docking analysis conducted in this study preliminarily elucidated the intricate binding mechanism of the compound **A12** with RT, furnishing valuable insights for subsequent endeavors related to structural refinement.

### 2.6 Molecular dynamics (MD) simulations

**Figure 4:**
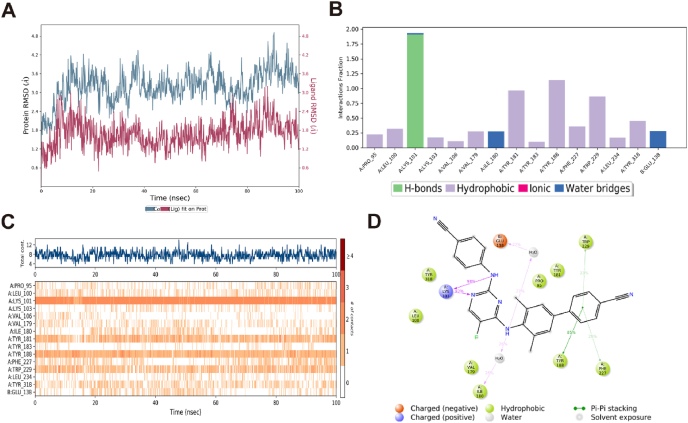
MD simulations of **A12** with WT HIV-1 RT (PDB code: 2ZD1). (A) RMSD of **A12**; (B, C) Protein–ligand contacts; (D) 2D interaction diagram and interactions that occur >20.0% of the simulation time.

To assess the dynamic properties and stability of **A12** bound to WT HIV-1 RT, MD simulation was performed for a duration of 100 ns using Schrödinger Maestro 11.4 (Fig. 4). As depicted in Fig. 4A. After an initial fluctuation caused by equilibration, the RMSD fluctuations rapidly reach a permissible threshold of 3 Å, demonstrating the high stability of the ligand–protein complex. Fig. 4B–D provides a comprehensive portrayal of the molecular interplay, which sheds light on the intricate and dynamic protein-ligand interactions. Multiple significant interactions, such as hydrogen bonding, hydrophobic contacts, and water bridges, are observed. A value of 0.75 signifies a 75% likelihood of occurrence for the interaction, whereas values surpassing 1.0 can be ascribed to the occurrence of multiple interactions between the protein residue and ligand (Fig. 4B). Fig. 4C illustrates the comprehensive count of distinct interactions established by the protein with the ligand, along with the identified residue species that are involved in the ligand interactions throughout the trajectory. Certain residues, such as LYS101 (K101) and TYR188 (Y188), indicated by a deep hue of orange within the trajectory frame, exhibit a propensity for establishing multiple distinct interactions with the ligand. The dynamic simulation interactions diagram in Fig. 4D indicates the persistence of two hydrogen bonds throughout the dynamic process, primarily involving the residue LYS101 (K101) and **A12**. Moreover, **A12** demonstrates sporadic associations with GLU138 (E138) and ILE180 (I180) through the formation of water-bridge hydrogen bonds. Additionally, hydrophobic interactions between **A12** and the proximal residues (VAL179 (V179), TYR318(Y318), TYR188 (Y188), TRP229 (W229), etc.) are observed.

### 2.7 Metabolic stability of **A12** in human liver microsomes

To evaluate the metabolic stability of **A12**, an *in vitro* human liver microsomal stability assay was conducted. As depicted in Table 4^7^, **A12** displays a half-life of 24.3 min, along with moderate clearance rates in microsomes (CL_int(mic)_ = 57.0 μL/min/mg) and liver (CL_int(liver)_ = 51.3 mL/min/kg). Although the outcome is not as favorable as ETR, it demonstrates about 2 times better than RPV.

**Table 4:** Human liver microsomal stability of **A12**.

Compd.	Human liver microsomal stability			
	*R*^2^	*t*_1/2_ (min)	CL_int(mic)_ (μL/min/mg)	CL_int(liver)_ (mL/min/kg)
**A12**	0.9871	24.3	57.0	51.3
ETR^a^	0.9798	65.5	21.1	19.0
RPV^a^	0.9544	12.8	108.4	97.6

a The data of ETR and RPV were obtained from Ref. 7.

### 2.8 Inhibitory effect of **A12** on six CYP enzymes

Given the potential for significant drug–drug interactions resulting from the inhibition of CYP2C9, CYP2C19, and CYP3A4 by ETR or RPV^7^^,^^36^, compound **A12** was further assessed for its inhibitory effect on six cytochrome P450 (CYP) isoenzymes. As displayed in Table 5^37^, **A12** is insensitive to CYP1A2, CYP3A4-T, and CYP3A4-M, which is substantiated by its >25 μmol/L IC_50_ values. Moreover, the inhibition of **A12** on CYP2C19 and CYP2D6 can be disregarded owing to its high IC_50_ value (>10 μmol/L). For CYP2C9, **A12** exhibits a moderate inhibitory efficacy (IC_50_ = 6.99 μmol/L), which is 25- and 20-fold less potent than that of ETR and RPV (IC_50_ = 0.277, 0.346 μmol/L), respectively. These findings demonstrate that **A12** has a lower propensity for drug–drug interactions.

**Table 5:** Inhibitory activity of **A12** against CYP.

Compd.	IC_50_ (μmol/L)					
	CYP1A2	CYP2C9	CYP2C19	CYP2D6	CYP3A4-M	CYP3A4-T
**A12**	>25	6.99	14.5	14.5	>25	>25
ETR^a^	7.48	0.277	0.496	12.0	41.3	–
RPV^a^	9.11	0.346	0.335	3.41	2.17	–
References	0.01^b^	8.29^c^	3.72^d^	0.224^e^	0.0627^f^	0.0423^g^

a The data of ETR and RPV were obtained from Ref. 37.

b Phenacetin.

c Tolbutamide.

d Mephenytoin.

e Dextromethorphan.

f Midazolam and.

g Testosterone were selected as the references.

### 2.9 hERG inhibitory activity

**Figure 5:**
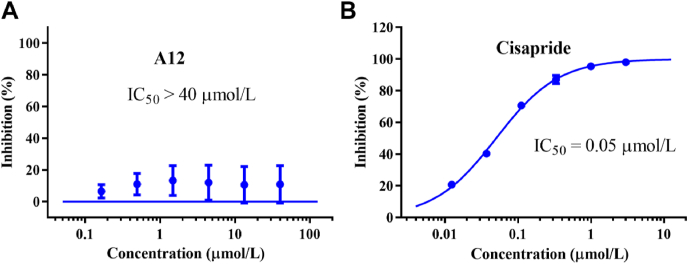
Inhibitory effects of **A12** (A) and cisapride (B) on the hERG channel.

The inhibition of the hERG channel by drugs has emerged as a significant obstacle in the development of pharmacologically safe agents because of cardiac side effects^38^^,^^39^. RPV has been examined as a potent hERG channel inhibitor (IC_50_ = 0.5 μmol/L), which reminds us that the assessment of hERG toxicity at an early stage is essential for the development of novel DAPY-type NNRTIs. Thus, the hERG inhibitory activity of **A12** was evaluated. Fig. 5 shows that **A12** only negligibly inhibits hERG, and its inhibitory potency (IC_50_ > 40 μmol/L) is considerably lower than that of the positive control cisapride (IC_50_ = 0.05 μmol/L). This result provides additional evidence for endorsing **A12** as a secure anti-HIV inhibitor.

### 2.10 PK analysis

The PK profile of **A12** was assessed in SD rats through the administration of a single dose *via* intravenous (i.v.) or oral (*p.o.*) routes. Table 6 indicates that **A12** exhibits a half-life of 5.53 h, following an intravenous administration at 1 mg/kg. Additionally, a maximum concentration (*C*_max_) of 2347 ng/mL and AUC_0‒∞_ of 3064 h ng/mL were detected. Following a gavage administration at 10 mg/kg, the half-life of **A12** is significantly prolonged, reaching 13.56 h, which is longer than that of RPV (*T*_1/2_ = 2.8 h)^40^; Further, a *C*_max_ of 1387 ng/mL is attained at 1 h and the AUC_0‒∞_ is up to 8934 h ng/mL. Notably, the oral bioavailability (*F*) of **A12** is 29.2%, which surpasses those of the lead compound **6** (*F* = 2.39%) and ETR (undetectable), and remains comparable to that of RPV · HCl (*F* = 32%)^15^^,^^41^. Then, the plasma protein binding rate (%PPB) of **A12** was evaluated. The result suggests that **A12** has a high %PPB of 99.5% in rat plasma, which may be attributed to its hydrophobic aromatic structure^42^. The high %PPB of **A12** can initially rationalize its long half-life *in vivo*.

**Table 6:** PK profiles of **A12** in rat^a^.

Parameter	A12	
	1.0 mg/kg (i.v.)	10.0 mg/kg (*p.o*.)
*T*_1/2_ (h)	5.53 ± 2.04	13.56 ± 6.64
*T*_max_ (h)	0.0833 ± 0.00	1.00 ± 0.00
*C*_max_ (ng/mL)	2347 ± 178.0	1387 ± 421.7
AUC_0–*t*_ (h·ng/mL)	2998 ± 141.7	7336 ± 1894
AUC_0–∞_ (h·ng/mL)	3064 ± 139.4	8934 ± 1848
MRT_0–*t*_ (h)	3.25 ± 0.575	6.18 ± 0.306
MRT_0–∞_ (h)	3.89 ± 1.035	13.52 ± 6.489
*F* (%)	–	29.2 ± 6.0

a PK parameters are presented mean ± SD (*n* = 3).

### 2.11 Acute toxicity assay

**Figure 6:**
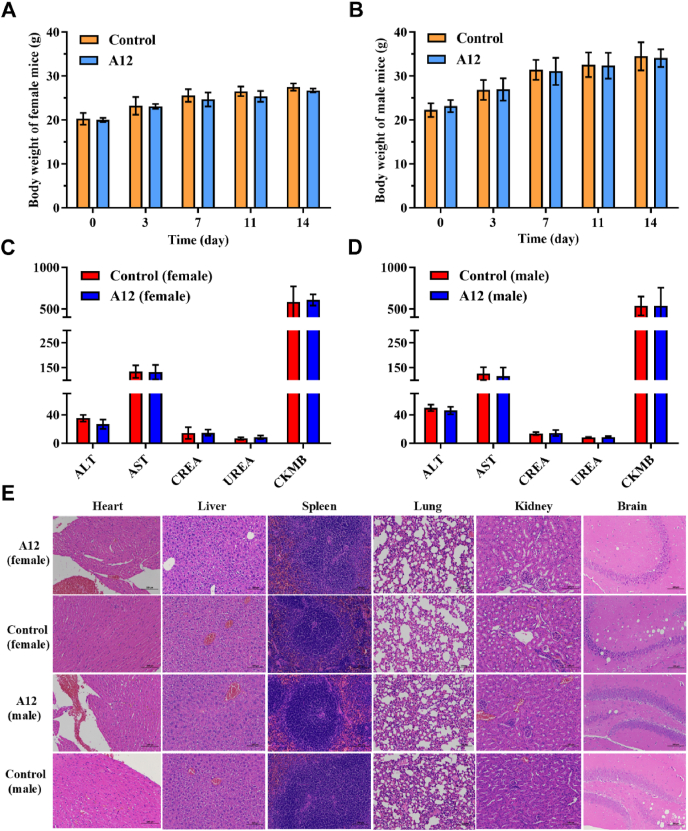
Temporal progression of body weight in panel A (female) and B (male). The blood biochemical parameters in panel C (female) and D (male). Panel E shows the histological alterations in mice assessed by HE staining (magnification: 200 × ; scale bars: 100 μm).

The acute toxicity of **A12**, administered as a single dose, was investigated in male and female ICR mice. Fig. 6 indicates that after intragastric (i.g.) administration of 2 g/kg of **A12**, all mice in the experimental group display growth patterns similar to that shown by the control group (Fig. 6A and B). No instances of mortality were recorded, and no noteworthy deviations in behavior were detected. Fourteen days after administration, no significant differences in blood biochemical parameters (including ALT, AST, CREA, UREA, CKMB) were observed between the groups of **A12** and control (Fig. 6C and D). Hematoxylin-eosin (HE) staining does not show any discernible pathological alteration in vital organs (brain, heart, liver, spleen, lung, and kidney), indicating that **A12** exhibits excellent tolerance at doses reaching 2 g/kg, without any manifestation of immediate toxicity (Fig. 6E).

## 3 Conclusions

In conclusion, a series of novel halogen-containing biphenyl-DAPY derivatives were developed using a bioisosterism strategy. The biological assessment results unveiled the notable inhibitory potency of these compounds against both WT HIV-1 and drug-resistant variants, alongside their reduced cytotoxicity. Notably, compound **A12** exhibited an exceptional inhibitory efficacy (EC_50_ = 1.9 nmol/L) against WT HIV-1, coupled with drastically reduced cytotoxicity (CC_50_ = 195 μmol/L) and a markedly elevated selectivity index (SI = 102,608), outperforming ETR and RPV. This analog also showed single-digit nanomolar activity against mutant strains, namely L100I, K103N, Y181C, and E138K (EC_50_ = 1.7–7.1 nmol/L), comparable to that of ETR. **A12** exhibited robust inhibitory properties against Y188L, F227L + V106A, and RES056, albeit with a slightly reduced potency compared to ETR, but a significantly increased activity compared to that of the lead compound **6**. The potent anti-HIV efficacy of **A12** was elucidated through molecular docking, which unveiled the binding mode of the ligand with RT. Furthermore, **A12** demonstrated improved drug-like profiles compared to the currently approved NNRTIs ETR and RPV: (1) the inhibition of **A12** on CYP enzymes was significantly lower than that of ETR and RPV, indicating a reduced likelihood of CYP-related drug–drug interactions; (2) compared to RPV, **A12** was less potent to hERG channel, suggesting its minimal potential for hERG-related cardiovascular side effects; (3) compared to ETR and RPV, **A12** demonstrated favorable PK profiles, including remarkable oral bioavailability of 29.2% and a significantly prolonged half-life (*T*_1/2_ = 13.56 h); (4) **A12** exhibited no discernible acute toxicity in mice of sound health upon oral administration of a dosage of 2 g/kg, and no notable pathological alterations were detected in the vital organs. Therefore, **A12** can be considered a viable drug candidate for efficacious oral therapy in managing HIV-1 infection.

## 4 Experimental

### 4.1 Chemistry

Chemical reagents and solvents procured from commercial suppliers were employed without undergoing additional purification. Silica gel (200−300 mesh, Qingdao Haiyang Chemical Company) was utilized for the execution of column chromatography. TLC was carried out on 0.25 mm silica gel plates visualized with UV light (*λ* = 254 nm) or iodine. ^1^H, ^13^C and ^19^F NMR were recorded in DMSO-*d*_6_ or CDCl_3_ on a Bruker AV-400 spectrometer with TMS as the internal standard. Chemical shifts (*δ*) are given in ppm relative to TMS, and coupling constants (*J*) in Hz. Melting points were measured at 589 nm by a SRS-optic melting point apparatus. HRMS were obtained on a Waters Quattro Micromass instrument and Brukersolari X-70 FT-MS instrument, respectively, using electrospray ionization (ESI) techniques. The purity of **A1**−**A24** and **B1**–**B22** was analyzed by high-performance liquid chromatography (HPLC) (Thermo fisher U3000) using a C18 column (Eslipse XDB, 4.6 mm × 150 mm, 5 μm) with gradient methanol/water (0.1% formic acid) or acetonitrile/water (0.1% formic acid) as the mobile phase at a flow rate of 0.8 mL/min: For **A1**–**A6**, **A11**–**A19**, **A21**–**A24**, **B1**–**B5**, **B7**–**B16**, **B19**, **B21**, and **B22**; (a) 0–7 min, 50%–85% MeOH; (b) 7–20 min, 85% MeOH; (c) 20–23 min, 85%–50% MeOH; (d) 23–25 min, 50% MeOH; For **A8**–**A10**, and **B6**: (a) 0–5 min, 50%–75% MeCN; (b) 5–20 min, 75% MeCN; (c) 20–23 min, 75%–50% MeCN; (d) 23–25 min, 50% MeCN; For **A7**, **A20**, **B17**, **B18**, and **B20**: (a) 0–5 min, 50%–60% MeCN; (b) 5–20 min, 60% MeCN; (c) 20–23 min, 60%–50% MeCN; (d) 23–25 min, 50% MeCN. The purity of all target compounds **A1**−**A24** and **B1**–**B22** used in subsequent experiments is ≥ 95% as determined by HPLC.

#### 4.1.1 General procedure for preparation of intermediates **9a**–**9v**

2,4-Dichloro-5-fluoropyrimidine or 2,4,5-trichloropyrimidine (1.2 mmol, 1.2 equiv.) and appropriate 4′-hydroxy[1,1′-biphenyl]-4-carbonitriles or 4-(4-pyridinyl)phenol (1.0 mmol, 1.0 equiv.) were dissolved in dry DMF (15 mL) in the present of K_2_CO_3_ (1.5 mmol, 1.5 equiv.). The reaction mixture was stirred at room temperature for 2–4 h until complete consumption of starting material as judged by TLC and then poured into water (20 mL), extracted with ethyl acetate (15 mL × 4). The organic layers were washed with water (10 mL × 2) and saturated NaCl aq. (10 mL × 2), dried over anhydrous Na_2_SO_4_, filtered, and concentrated under reduced pressure. The residue was then purified by silica gel column chromatography, eluting with ethyl acetate/petroleum ether to obtain **9a**–**9v**.

4′-((2-Chloro-5-fluoropyrimidin-4-yl)oxy)-3′-fluoro-[1,1′-biphenyl]-4-carbonitrile (**9a**). Yield 90%, white solid, mp: 172‒173 °C. ^1^H NMR (400 MHz, DMSO-*d*_6_) *δ*: 8.90 (d, *J* = 2.4 Hz, 1H), 8.22–7.89 (m, 5H), 7.80–7.73 (m, 1H), 7.70–7.61 (m, 1H). ^13^C NMR (100 MHz, DMSO-*d*_6_) *δ*: 157.4 (d, *J* = 11.9 Hz), 153.6 (d, *J* = 248.2 Hz), 152.0 (d, *J* = 4.7 Hz), 147.7 (d, *J* = 20.6 Hz), 145.5 (d, *J* = 262.6 Hz), 142.3 (d, *J* = 1.8 Hz), 138.5 (d, *J* = 7.0 Hz), 138.2 (d, *J* = 12.8 Hz), 132.9, 127.8, 124.6, 124.2 (d, *J* = 3.2 Hz), 118.7, 115.8 (d, *J* = 19.6 Hz), 110.8. ^19^F NMR (376 MHz, DMSO-*d*_6_) *δ*: −127.8, −154.3. HRMS (ESI): Calcd. for C_17_H_9_ClF_2_N_3_O [M + H]^+^, 344.0397; found, 344.0400.

3′-Chloro-4′-((2-chloro-5-fluoropyrimidin-4-yl)oxy)-[1,1′-biphenyl]-4-carbonitrile (**9b**). Yield 88%, white solid, mp: 174‒175 °C. ^1^H NMR (400 MHz, DMSO-*d*_6_) *δ*: 8.91 (d, *J* = 2.5 Hz, 1H), 8.10 (d, *J* = 2.2 Hz, 1H), 8.04–7.94 (m, 4H), 7.92–7.87 (m, 1H), 7.69 (d, *J* = 8.5 Hz, 1H). ^13^C NMR (100 MHz, DMSO-*d*_6_) *δ*: 157.5 (d, *J* = 11.9 Hz), 152.0 (d, *J* = 4.7 Hz), 147.7 (d, *J* = 20.5 Hz), 147.1, 145.5 (d, *J* = 262.7 Hz), 142.2, 138.3, 132.9, 129.1, 127.9, 127.6, 126.4, 124.6, 118.7, 110.9. ^19^F NMR (376 MHz, DMSO-*d*_6_) *δ*: −154.3. HRMS (ESI): Calcd. for C_17_H_9_Cl_2_FN_3_O [M + H]^+^, 360.0101, Found 360.0105.

4′-((2-Chloro-5-fluoropyrimidin-4-yl)oxy)-3′-methyl-[1,1′-biphenyl]-4-carbonitrile (**9c**). Yield 80%, white solid, mp: 184‒186 °C. ^1^H NMR (400 MHz, DMSO-*d*_6_) *δ*: 8.83 (d, *J* = 2.5 Hz, 1H), 8.04–7.86 (m, 4H), 7.80 (d, *J* = 2.3 Hz, 1H), 7.74–7.64 (m, 1H), 7.42 (d, *J* = 8.4 Hz, 1H), 2.23 (s, 3H). ^13^C NMR (100 MHz, DMSO-*d*_6_) *δ*: 158.1 (d, *J* = 11.7 Hz), 152.0 (d, *J* = 4.7 Hz), 150.1, 146.9 (d, *J* = 20.6 Hz), 145.9 (d, *J* = 262 Hz), 143.7, 136.6, 132.8, 130.7, 130.3, 127.6, 126.2, 122.6, 118.8, 110.2, 15.8. ^19^F NMR (376 MHz, DMSO-*d*_6_) *δ*: −154.4. HRMS (ESI): Calcd. for C_18_H_12_ClFN_3_O [M + H]^+^, 340.0647, Found 340.0651.

4′-((2-Chloro-5-fluoropyrimidin-4-yl)oxy)-3′-methoxy-[1,1′-biphenyl]-4-carbonitrile (**9d**). Yield 76%, white solid, mp: 221‒222 °C. ^1^H NMR (400 MHz, DMSO-*d*_6_) *δ*: 8.83 (d, *J* = 2.6 Hz, 1H), 8.14–7.90 (m, 4H), 7.56 (d, *J* = 1.8 Hz, 1H), 7.49–7.40 (m, 2H), 3.87 (s, 3H). ^13^C NMR (100 MHz, DMSO-*d*_6_) *δ*: 158.1 (d, *J* = 11.5 Hz), 152.1 (d, *J* = 4.7 Hz), 151.0, 147.1 (d, *J* = 20.6 Hz), 145.5 (d, *J* = 261.9 Hz), 143.8, 140.0, 138.0, 132.8, 127.9, 123.2, 119.8, 118.8, 112.3, 110.3, 56.2. ^19^F NMR (376 MHz, DMSO-*d*_6_) *δ*: −154.8. HRMS (ESI): Calcd. for C_18_H_12_ClFN_3_O_2_ [M + H]^+^, 356.0597, Found 356.0601.

4′-((2-Chloro-5-fluoropyrimidin-4-yl)oxy)-3′,5′-difluoro-[1,1′-biphenyl]-4-carbonitrile (**9e**). Yield 47%, white solid, mp: 198‒199 °C. ^1^H NMR (400 MHz, CDCl_3_) *δ*: 8.45 (d, *J* = 1.8 Hz, 1H), 7.82–7.73 (m, 2H), 7.74–7.61 (m, 2H), 7.29 (d, *J* = 8.4 Hz, 2H). ^13^C NMR (100 MHz, CDCl_3_) *δ*: 157.2 (d, *J* = 11.6 Hz), 155.5 (dd, *J* = 252.9, 4.4 Hz), 153.6 (d, *J* = 5.0 Hz), 146.76 (d, *J* = 19.8 Hz), 146.75, 144.1, 142.6 (t, *J* = 2.3 Hz), 139.0 (t, *J* = 8.5 Hz), 133.1, 127.8, 118.5, 112.7, 111.5 (d, *J* = 22.7 Hz). ^19^F NMR (376 MHz, CDCl_3_) *δ*: −123.7, −153.9. HRMS (ESI): Calcd. for C_17_H_6_ClF_3_N_3_O [M − H]^-^, 360.0157, Found 360.0168.

4′-((2-Chloro-5-fluoropyrimidin-4-yl)oxy)-3′,5′-dimethyl-[1,1′-biphenyl]-4-carbonitrile (**9f**). Yield 71%, white solid, mp: 215‒216 °C. ^1^H NMR (400 MHz, DMSO-*d*_6_) *δ*: 8.86 (d, *J* = 2.5 Hz, 1H), 8.55–7.81 (m, 4H), 7.61 (s, 2H), 2.17 (s, 6H). ^13^C NMR (100 MHz, DMSO-*d*_6_) *δ*: 157.3 (d, *J* = 11.6 Hz), 152.3 (d, *J* = 4.7 Hz), 148.7, 147.2 (d, *J* = 20.6 Hz), 145.5 (d, *J* = 262 Hz), 143.7, 136.4, 132.8, 130.9, 127.7, 127.6, 118.8, 110.1, 16.0. ^19^F NMR (376 MHz, DMSO-*d*_6_) *δ*: −154.4. HRMS (ESI): Calcd. for C_19_H_14_ClFN_3_O [M + H]^+^, 354.0804, Found 354.0807.

2-Chloro-5-fluoro-4-(2-fluoro-4-(pyridin-4-yl)phenoxy)pyrimidine (**9g**). Yield 94%. Pale yellow solid, mp: 136–138 °C. ^1^H NMR (400 MHz, DMSO-*d*_6_) *δ*: 8.91 (d, *J* = 2.5 Hz, 1H), 8.77–8.61 (m, 2H), 8.07–7.97 (m, 1H), 7.87–7.78 (m, 3H), 7.72–7.65 (m, 1H). ^13^C NMR (100 MHz, DMSO-*d*_6_) *δ*: 157.4 (d, *J* = 11.8 Hz), 153.7 (d, *J* = 248.3 Hz), 152.0 (d, *J* = 4.6 Hz), 150.4, 147.7 (d, *J* = 20.5 Hz), 145.5 (d, *J* = 262.6 Hz), 144.8 (d, *J* = 1.9 Hz), 138.5 (d, *J* = 12.7 Hz), 137.4 (d, *J* = 7.0 Hz), 124.7, 123.9 (d, *J* = 3.4 Hz), 121.3, 115.6 (d, *J* = 19.4 Hz). ^19^F NMR (376 MHz, DMSO-*d*_6_) *δ*: −127.7, −154.3. HRMS (ESI): Calcd. for C_15_H_9_ClF_2_N_3_O [M + H]^+^, 320.0397, Found 320.0390.

2-Chloro-4-(2-chloro-4-(pyridin-4-yl)phenoxy)-5-fluoropyrimidine (**9h**). Yield 92%. Pale yellow solid, mp: 117–119 °C. ^1^H NMR (400 MHz, DMSO-*d*_6_) *δ*: 8.91 (d, *J* = 2.4 Hz, 1H), 8.72–8.65 (m, 2H), 8.14 (d, *J* = 2.2 Hz, 1H), 7.98–7.89 (m, 1H), 7.86–7.78 (m, 2H), 7.70 (d, *J* = 8.5 Hz, 1H). ^13^C NMR (100 MHz, DMSO-*d*_6_) *δ*: 157.4 (d, *J* = 11.9 Hz), 152.0 (d, *J* = 4.7 Hz), 150.3, 147.7 (d, *J* = 20.5 Hz), 147.5, 145.5 (d, *J* = 262.9 Hz), 144.7, 137.3, 128.9, 127.4, 126.5, 124.7, 121.4. ^19^F NMR (376 MHz, DMSO-*d*_6_) *δ*: −154.2. HRMS (ESI): Calcd. for C_15_H_9_Cl_2_FN_3_O [M + H]^+^, 336.0101, Found 336.0110.

2-Chloro-5-fluoro-4-(2-methyl-4-(pyridin-4-yl)phenoxy)pyrimidine (**9i**). Yield 93%. Pale yellow solid, mp: 113–115 °C. ^1^H NMR (400 MHz, DMSO-*d*_6_) *δ*: 8.84 (d, *J* = 2.5 Hz, 1H), 8.70–8.63 (m, 2H), 7.87–7.72 (m, 4H), 7.43 (d, *J* = 8.4 Hz, 1H), 2.24 (s, 3H). ^13^C NMR (100 MHz, DMSO-*d*_6_) *δ*: 158.1 (d, *J* = 11.7 Hz), 152.0 (d, *J* = 4.6 Hz), 150.5, 150.2, 147.0 (d, *J* = 20.7 Hz), 146.1, 145.9 (d, *J* = 261.9 Hz), 135.5, 130.8, 130.0, 126.0, 122.7, 121.3, 15.8. ^19^F NMR (376 MHz, DMSO-*d*_6_) *δ*: −154.8. HRMS (ESI): Calcd. for C_16_H_12_ClFN_3_O [M + H]^+^, 316.0647, Found 316.0644.

2-Chloro-5-fluoro-4-(2-methoxy-4-(pyridin-4-yl)phenoxy)pyrimidine (**9j**). Yield 84%. Pale yellow solid, mp: 157–158 °C. ^1^H NMR (400 MHz, DMSO-*d*_6_) *δ*: 8.83 (d, *J* = 2.5 Hz, 1H), 8.74–8.60 (m, 2H), 8.11–7.71 (m, 2H), 7.60 (d, *J* = 1.8 Hz, 1H), 7.56–7.31 (m, 2H), 3.87 (s, 3H). ^13^C NMR (100 MHz, DMSO-*d*_6_) *δ*: 158.1 (d, *J* = 11.6 Hz), 152.1 (d, *J* = 4.7 Hz), 151.1, 150.1, 147.1 (d, *J* = 20.6 Hz), 146.3, 145.4 (d, *J* = 262.0 Hz), 140.4, 136.9, 123.3, 121.5, 119.6, 112.1, 56.3. ^19^F NMR (376 MHz, DMSO-*d*_6_) *δ*: −154.4. HRMS (ESI): Calcd. for C_16_H_12_ClFN_3_O_2_ [M + H]^+^, 332.0597, Found 332.0600.

2-Chloro-4-(2,6-difluoro-4-(pyridin-4-yl)phenoxy)-5-fluoropyrimidine (**9k**). Yield 38%. white solid, mp: 160–161 °C. ^1^H NMR (400 MHz, DMSO-*d*_6_) *δ*: 8.98 (d, *J* = 2.5 Hz, 1H), 8.78–8.46 (m, 2H), 8.10–7.90 (m, 2H), 7.89–7.83 (m, 2H).^13^C NMR (100 MHz, DMSO-*d*_6_) *δ*: 156.3 (d, *J* = 12.2 Hz), 154.7 (dd, *J* = 249.8, 4.4 Hz), 152.0 (d, *J* = 4.8 Hz), 150.4, 148.6 (d, *J* = 20.3 Hz), 146.4, 143.8, 137.4 (t, *J* = 8.9 Hz), 127.0 (t, *J* = 16.0 Hz), 121.3, 111.6 (d, *J* = 22.3 Hz). ^19^F NMR (376 MHz, DMSO-*d*_6_) *δ*: −124.8, −154.1. HRMS (ESI): Calcd. for C_15_H_8_ClF_3_N_3_O [M + H]^+^, 338.0303, Found 338.0311.

2-Chloro-4-(2,6-dimethyl-4-(pyridin-4-yl)phenoxy)-5-fluoropyrimidine (**9l**). Yield 93%, white solid, mp: 210–211 °C. ^1^H NMR (400 MHz, DMSO-*d*_6_) *δ*: 8.86 (d, *J* = 2.3 Hz, 1H), 8.73–8.61 (m, 2H), 7.78–7.71 (m, 2H), 7.66 (s, 2H), 2.17 (s, 6H). ^13^C NMR (100 MHz, DMSO-*d*_6_) *δ*: 157.3 (d, *J* = 11.4 Hz), 152.3, 150.2, 149.0, 147.2 (d, *J* = 20.5 Hz), 146.2, 145.5 (d, *J* = 262.1 Hz), 135.3, 131.0, 127.5, 121.2, 16.0. ^19^F NMR (376 MHz, DMSO-*d*_6_) *δ*: −154.6. HRMS (ESI): Calcd. for C_17_H_14_ClFN_3_O [M + H]^+^, 330.0804, Found 330.0808.

4′-((2,5-Dichloropyrimidin-4-yl)oxy)-3′-fluoro-[1,1′-biphenyl]-4-carbonitrile (**9m**). Yield 91%, white solid, mp: 216‒218 °C. ^1^H NMR (400 MHz, DMSO-*d*_6_) *δ*: 8.92 (s, 1H), 8.08–7.90 (m, 5H), 7.79–7.72 (m, 1H), 7.70–7.61 (m, 1H). ^13^C NMR (100 MHz, DMSO-*d*_6_) *δ*: 164.0, 159.8, 156.3, 153.5 (d, *J* = 248.1 Hz), 142.3 (d, *J* = 2.0 Hz), 138.49 (d, *J* = 19.0 Hz), 138.46, 132.9, 127.8, 124.6, 124.2 (d, *J* = 3.2 Hz), 118.7, 116.3, 115.8 (d, *J* = 19.5 Hz), 110.8. ^19^F NMR (376 MHz, DMSO-*d*_6_) *δ*: −117.9. HRMS (ESI): Calcd. for C_17_H_9_Cl_2_FN_3_O [M + H]^+^, 360.0101, Found 360.0101.

3′-Chloro-4′-((2,5-dichloropyrimidin-4-yl)oxy)-[1,1′-biphenyl]-4-carbonitrile (**9n**). Yield 80%, white solid, mp: 202‒204 °C. ^1^H NMR (400 MHz, DMSO-*d*_6_) *δ*: 8.94 (s, 1H), 8.09 (d, *J* = 2.2 Hz, 1H), 8.05–7.93 (m, 4H), 7.92–7.85 (m, 1H), 7.67 (d, *J* = 8.5 Hz, 1H). ^13^C NMR (100 MHz, DMSO-*d*_6_) *δ*: 164.1, 159.8, 156.2, 147.4, 142.1, 138.3, 132.9, 129.0, 127.9, 127.6, 126.3, 124.6, 118.7, 116.4, 110.8. HRMS (ESI): Calcd. for C_17_H_9_Cl_3_N_3_O [M + H]^+^, 375.9806, Found 375.9810.

4′-((2,5-Dichloropyrimidin-4-yl)oxy)-3′-methyl-[1,1′-biphenyl]-4-carbonitrile (**9o**). Yield 64%, white solid, mp: 199‒200 °C. ^1^H NMR (400 MHz, DMSO-*d*_6_) *δ*: 8.87 (s, 1H), 8.02–7.89 (m, 4H), 7.81–7.78 (m, 1H), 7.75–7.63 (m, 1H), 7.40 (d, *J* = 8.4 Hz, 1H), 2.20 (s, 3H). ^13^C NMR (100 MHz, DMSO-*d*_6_) *δ*: 164.4, 159.2, 156.3, 150.4, 143.7, 136.5, 132.8, 130.6, 130.2, 127.6, 126.2, 122.6, 118.8, 116.5, 110.1, 15.7. HRMS (ESI): Calcd. for C_18_H_12_Cl_2_N_3_O [M + H]^+^, 356.0352, Found 356.0355.

4′-((2,5-Dichloropyrimidin-4-yl)oxy)-3′-methoxy-[1,1′-biphenyl]-4-carbonitrile (**9p**). Yield 89%, white solid, mp: 228‒230 °C. ^1^H NMR (400 MHz, DMSO-*d*_6_) *δ*: 8.86 (s, 1H), 8.08–7.92 (m, 4H), 7.64–7.52 (m, 1H), 7.52–7.36 (m, 2H), 3.86 (s, 3H). ^13^C NMR (100 MHz, DMSO-*d*_6_) *δ*: 164.6, 159.3, 156.3, 150.9, 143.8, 140.4, 137.9, 132.8, 127.9, 123.1, 119.8, 118.8, 116.1, 112.3, 110.3, 56.2. HRMS (ESI): Calcd. for C_18_H_12_Cl_2_N_3_O_2_ [M + H]^+^, 372.0301, Found 372.0303.

4′-((2,5-Dichloropyrimidin-4-yl)oxy)-3′,5′-dimethyl-[1,1′-biphenyl]-4-carbonitrile (**9q**). Yield 49%, white solid, mp: 228‒230 °C. ^1^H NMR (400 MHz, DMSO-*d*_6_) *δ*: 8.89 (s, 1H), 8.05–7.86 (m, 4H), 7.61 (s, 2H), 2.14 (s, 6H). ^13^C NMR (100 MHz, DMSO-*d*_6_) *δ*: 163.8, 159.4, 156.6, 149.2, 143.8, 136.4, 132.8, 130.8, 127.7, 127.6, 118.9, 116.1, 110.1, 16.0. HRMS (ESI): Calcd. for C_19_H_14_Cl_2_N_3_O [M + H]^+^, 370.0508, Found 370.0514.

2,5-Dichloro-4-(2-fluoro-4-(pyridin-4-yl)phenoxy)pyrimidine (**9r**). Yield 85%, pale yellow solid, mp: 140‒142 °C. ^1^H NMR (400 MHz, DMSO-*d*_6_) *δ*: 8.93 (s, 1H), 8.72–8.66 (m, 2H), 8.04–7.98 (m, 1H), 7.86–7.77 (m, 3H), 7.73–7.64 (m, 1H). ^13^C NMR (100 MHz, DMSO-*d*_6_) *δ*: 164.0, 159.8, 156.3, 153.6 (d, *J* = 248.1 Hz), 150.4, 144.8 (d, *J* = 2.2 Hz), 138.9 (d, *J* = 12.7 Hz), 137.4 (d, *J* = 7.0 Hz), 124.7, 123.9 (d, *J* = 3.2 Hz), 121.3, 116.3, 115.6 (d, *J* = 19.5 Hz). ^19^F NMR (376 MHz, DMSO-*d*_6_) *δ*: −127.9. HRMS (ESI): Calcd. for C_15_H_9_Cl_2_FN_3_O [M + H]^+^, 336.0101, Found 336.0097.

2,5-Dichloro-4-(2-chloro-4-(pyridin-4-yl)phenoxy)pyrimidine (**9s**). Yield 38%, pale yellow solid, mp: 140‒141 °C. ^1^H NMR (400 MHz, DMSO-*d*_6_) *δ*: 8.94 (s, 1H), 8.73–8.63 (m, 2H), 8.14 (d, *J* = 2.2 Hz, 1H), 7.99–7.91 (m, 1H), 7.89–7.79 (m, 2H), 7.69 (d, *J* = 8.5 Hz, 1H). ^13^C NMR (100 MHz, DMSO-*d*_6_) *δ*: 164.0, 159.8, 156.2, 150.3, 147.8, 144.7, 137.2, 128.8, 127.4, 126.4, 124.7, 121.4, 116.4. HRMS (ESI): Calcd. for C_15_H_9_Cl_3_N_3_O [M + H]^+^, 351.9806, Found 351.9807.

2,5-Dichloro-4-(2-methyl-4-(pyridin-4-yl)phenoxy)pyrimidine (**9t**). Yield 53%, pale yellow solid, mp: 125–127 °C. ^1^H NMR (400 MHz, DMSO-*d*_6_) *δ*: 8.87 (s, 1H), 8.75–8.51 (m, 2H), 7.90–7.72 (m, 4H), 7.42 (d, *J* = 8.4 Hz, 1H), 2.21 (s, 3H). ^13^C NMR (100 MHz, DMSO-*d*_6_) *δ*: 164.4, 159.2, 156.4, 150.8, 150.2, 146.1, 135.4, 130.7, 130.0, 125.9, 122.7, 121.3, 116.5, 15.7. HRMS (ESI): Calcd. for C_16_H_12_Cl_2_N_3_O [M + H]^+^, 332.0352, Found 332.0356.

2,5-Dichloro-4-(2-methoxy-4-(pyridin-4-yl)phenoxy)pyrimidine (**9u**). Yield 61%, pale yellow solid, mp: 193–195 °C. ^1^H NMR (400 MHz, DMSO-*d*_6_) *δ*: 8.86 (s, 1H), 8.76–8.61 (m, 2H), 7.86–7.78 (m, 2H), 7.60 (d, *J* = 2.0 Hz, 1H), 7.57–7.39 (m, 2H), 3.87 (s, 3H). ^13^C NMR (100 MHz, DMSO-*d*_6_) *δ*: 164.6, 159.3, 156.3, 151.0, 150.2, 146.3, 140.7, 136.8, 123.2, 121.5, 119.6, 116.1, 112.1, 56.3. HRMS (ESI): Calcd. for C_16_H_12_Cl_2_N_3_O_2_ [M + H]^+^, 348.0301, Found 348.0292.

2,5-Dichloro-4-(2,6-dimethyl-4-(pyridin-4-yl)phenoxy)pyrimidine (**9v**). Yield 83%, white solid, mp: 244‒245 °C. ^1^H NMR (400 MHz, DMSO-*d*_6_) *δ*: 8.90 (d, *J* = 0.7 Hz, 1H), 8.69–8.61 (m, 2H), 7.81–7.57 (m, 4H), 2.15 (s, 6H). ^13^C NMR (100 MHz, DMSO-*d*_6_) *δ*: 163.7, 159.4, 156.6, 150.2, 149.5, 146.1, 135.2, 130.8, 127.4, 121.2, 116.1, 15.9. HRMS (ESI): Calcd. for C_17_H_14_Cl_2_N_3_O [M + H]^+^, 346.0508, Found 346.0516.

#### 4.1.2 General procedure for preparation of intermediates **10a**–**10x**

Under nitrogen atmosphere, a mixture of Pd(OAc)_2_ (0.1 mmol, 0.05 equiv.) and Xantphos (0.12 mmol, 0.06 equiv.) was stirred in dry 1,4-dioxane (5 mL) at room temperature for 15 min and then transferred to a solution of 2,4-dichloro-5-fluoropyrimidine or 2,4,5-trichloropyrimidine (2.4 mmol, 1.2 equiv.), Cs_2_CO_3_ (8.0 mmol, 4.0 equiv.) and appropriate substituted 4′-amino-[1,1′-biphenyl]-4-carbonitrile or 4-(4-pyridinyl)benzenamine (2.0 mmol, 1.0 equiv.) in dry 1,4-dioxane (30 mL). The reaction mixture was stirred at 110 °C for 1–2 h under a nitrogen atmosphere. Until completion, the solution was cooled to room temperature, then evaporated under reduced pressure, diluted with water, and extracted with ethyl acetate (20 mL × 3). The combined organic layers were washed with saturated NaCl aq. (15 mL × 2), dried over anhydrous Na_2_SO_4_, filtered, and concentrated under reduced pressure. The obtained residue was then purified by silica gel column chromatography, eluting with ethyl acetate/petroleum ether, to give **10a**–**10x**.

4′-((2-Chloro-5-fluoropyrimidin-4-yl)amino)-3′-fluoro-[1,1′-biphenyl]-4-carbonitrile (**10a**). Yield 54%, white solid, mp: 213‒214 °C. ^1^H NMR (400 MHz, DMSO-*d*_6_) *δ*: 10.06 (s, 1H), 8.36 (d, *J* = 3.3 Hz, 1H), 8.00–7.91 (m, 4H), 7.80 (dd, *J* = 11.9, 2.0 Hz, 1H), 7.73–7.58 (m, 2H). ^13^C NMR (100 MHz, DMSO-*d*_6_) *δ*: 156.5 (d, *J* = 248.3 Hz), 153.1 (d, *J* = 3.2 Hz), 151.8 (d, *J* = 12.3 Hz), 145.4 (d, *J* = 258.0 Hz), 142.6 (d, *J* = 2.0 Hz), 142.2 (d, *J* = 20.6 Hz), 137.6 (d, *J* = 7.6 Hz), 132.8, 127.9 (d, *J* = 2.0 Hz), 127.5, 124.9 (d, *J* = 12.5 Hz), 123.1 (d, *J* = 3.2 Hz), 118.7, 114.7 (d, *J* = 21.4 Hz), 110.5. ^19^F NMR (376 MHz, DMSO-*d*_6_) *δ*: −118.4, −154.6. HRMS (ESI): Calcd. for C_17_H_10_ClF_2_N_4_ [M + H]^+^, 343.0557, Found 343.0556.

3′-Chloro-4′-((2-chloro-5-fluoropyrimidin-4-yl)amino)-[1,1′-biphenyl]-4-carbonitrile (**10b**). Yield 51%, white solid, mp: 235‒236 °C. ^1^H NMR (400 MHz, DMSO-*d*_6_) *δ*: 10.06 (s, 1H), 8.38–8.34 (m, 1H), 8.06–7.91 (m, 5H), 7.85–7.77 (m, 1H), 7.70–7.56 (m, 1H). ^13^C NMR (100 MHz, DMSO-*d*_6_) *δ*: 153.2 (d, *J* = 3.2 Hz), 152.2 (d, *J* = 12.2 Hz), 145.3 (d, *J* = 257.8 Hz), 142.5, 142.1 (d, *J* = 20.5 Hz), 138.0, 134.5, 132.9, 131.1, 129.3, 128.3, 127.7, 126.4, 118.7, 110.7. ^19^F NMR (376 MHz, DMSO-*d*_6_) *δ*: −154.8. HRMS (ESI): Calcd. for C_17_H_10_Cl_2_FN_4_ [M + H]^+^, 359.0261, Found 359.0261.

4′-((2-Chloro-5-fluoropyrimidin-4-yl)amino)-3′-methyl-[1,1′-biphenyl]-4-carbonitrile (**10c**).Yield 56%. White solid, mp: 215‒216 °C. ^1^H NMR (400 MHz, DMSO-*d*_6_) *δ*: 9.77 (s, 1H), 8.29 (d, *J* = 3.5 Hz, 1H), 8.02–7.84 (m, 4H), 7.76–7.59 (m, 2H), 7.47–7.36 (m, 1H), 2.28 (s, 3H). ^13^C NMR (100 MHz, DMSO-*d*_6_) *δ*: 153.3 (d, *J* = 3.0 Hz), 152.4 (d, *J* = 12.3 Hz), 145.3 (d, *J* = 257.0 Hz), 144.0, 141.5 (d, *J* = 20.7 Hz), 136.4, 136.0, 135.1, 132.8, 129.3, 127.55, 127.46, 125.0, 118.9, 109.9, 18.0. ^19^F NMR (376 MHz, DMSO-*d*_6_) *δ*: −154.8. HRMS (ESI): Calcd. for C_18_H_13_ClFN_4_ [M + H]^+^, 339.0807, Found 339.0806.

4′-((2-Chloro-5-fluoropyrimidin-4-yl)amino)-3′-methoxy-[1,1′-biphenyl]-4-carbonitrile (**10d**). Yield 57%. Pale yellow solid, mp: 257‒259 °C. ^1^H NMR (400 MHz, DMSO-*d*_6_) *δ*: 9.37 (s, 1H), 8.30 (d, *J* = 3.4 Hz, 1H), 8.06–7.90 (m, 4H), 7.72–7.62 (m, 1H), 7.52–7.35 (m, 2H), 3.93 (s, 3H).^13^C NMR (100 MHz, DMSO-*d*_6_) *δ*: 153.2, 153.1, 151.9 (d, *J* = 11.7 Hz), 145.3 (d, *J* = 257.7 Hz), 144.1, 141.5 (d, *J* = 20.8 Hz), 136.8, 132.7, 127.6, 126.2, 125.9, 119.2, 118.9, 110.7, 110.0, 56.0. ^19^F NMR (376 MHz, DMSO-*d*_6_) *δ*: −155.1. HRMS (ESI): Calcd. for C_18_H_13_ClFN_4_O [M + H]^+^, 355.0756, Found 355.0757.

4′-((2-Chloro-5-fluoropyrimidin-4-yl)amino)-3′,5′-difluoro-[1,1′-biphenyl]-4-carbonitrile (**10e**). Yield 74%. White solid, mp: 238‒239 °C. ^1^H NMR (400 MHz, DMSO-*d*_6_) *δ*: 10.16 (s, 1H), 8.61–8.33 (m, 1H), 8.13–7.92 (m, 4H), 7.82–7.63 (m, 2H). ^13^C NMR (100 MHz, DMSO-*d*_6_) *δ*: 158.3 (dd, *J* = 249.2, 5.6 Hz), 153.3 (d, *J* = 3.2 Hz), 152.1 (d, *J* = 12.4 Hz), 145.4 (d, *J* = 257.9 Hz), 142.6 (d, *J* = 20.4 Hz), 141.5, 138.8 (t, *J* = 9.6 Hz), 132.9, 127.8, 118.6, 113.8 (t, *J* = 16.8 Hz), 111.2, 110.9 (d, *J* = 24.0 Hz). ^19^F NMR (376 MHz, DMSO-*d*_6_) *δ*: −116.2, −155.0. HRMS (ESI): Calcd. for C_17_H_9_ClF_3_N_4_ [M + H]^+^, 361.0462, Found 361.0465.

4′-((2-Chloro-5-fluoropyrimidin-4-yl)amino)-3′,5′-dimethyl-[1,1′-biphenyl]-4-carbonitrile (**10f**). Yield 72%. White solid, mp: 245‒246 °C. ^1^H NMR (400 MHz, DMSO-*d*_6_) *δ*: 9.65 (s, 1H), 8.26 (d, *J* = 2.7 Hz, 1H), 7.99–7.86 (m, 4H), 7.55 (s, 2H), 2.22 (s, 6H). ^13^C NMR (100 MHz, DMSO-*d*_6_) *δ*: 153.6 (d, *J* = 2.9 Hz), 152.4 (d, *J* = 12.5 Hz), 145.2 (d, *J* = 256.8 Hz), 144.1, 141.0 (d, *J* = 19.3 Hz), 136.9, 136.6, 134.8, 132.7, 127.5, 126.7, 118.8, 110.0, 18.1. ^19^F NMR (376 MHz, DMSO-*d*_6_) *δ*: −155.7. HRMS (ESI): Calcd. for C_19_H_15_ClFN_4_ [M + H]^+^, 353.0964, Found 353.0961.

2-Chloro-5-fluoro*-N-*(2-fluoro-4-(pyridin-4-yl)phenyl)pyrimidin-4-amine (**10g**). Yield 38%, white solid, mp: 177‒179 °C. ^1^H NMR (400 MHz, DMSO-*d*_6_) *δ*: 10.08 (s, 1H), 8.73–8.63 (m, 2H), 8.37 (d, *J* = 3.3 Hz, 1H), 7.86 (dd, *J* = 11.8, 2.1 Hz, 1H), 7.82–7.71 (m, 3H), 7.70–7.59 (m, 1H). ^13^C NMR (100 MHz, DMSO-*d*_6_) *δ*: 156.6 (d, *J* = 248.5 Hz), 153.2 (d, *J* = 3.3 Hz), 151.8 (d, *J* = 12.2 Hz), 150.3, 145.4 (d, *J* = 258.0 Hz), 145.1 (d, *J* = 2.0 Hz), 142.2 (d, *J* = 20.6 Hz), 136.5 (d, *J* = 7.6 Hz), 128.0 (d, *J* = 2.0 Hz), 125.4 (d, *J* = 12.6 Hz), 122.9 (d, *J* = 3.3 Hz), 121.1, 114.5 (d, *J* = 21.4 Hz). ^19^F NMR (376 MHz, DMSO-*d*_6_) *δ*: −118.3, −154.6. HRMS (ESI): Calcd. for C_15_H_10_ClF_2_N_4_ [M + H]^+^, 319.0557, Found 319.0554.

2-Chloro*-N-*(2-chloro-4-(pyridin-4-yl)phenyl)-5-fluoropyrimidin-4-amine (**10h**). Yield 26%, white solid, mp: 155‒156 °C. ^1^H NMR (400 MHz, DMSO-*d*_6_) *δ*: 10.05 (s, 1H), 8.77–8.61 (m, 2H), 8.36 (d, *J* = 3.3 Hz, 1H), 8.05 (d, *J* = 2.1 Hz, 1H), 7.91–7.76 (m, 3H), 7.67 (d, *J* = 8.3 Hz, 1H). ^13^C NMR (100 MHz, DMSO-*d*_6_) *δ*: 153.2 (d, *J* = 3.2 Hz), 152.1 (d, *J* = 12.2 Hz), 150.3, 145.2 (d, *J* = 257.7 Hz), 144.9, 142.1 (d, *J* = 20.5 Hz), 137.0, 134.9, 131.1, 129.2, 128.0, 126.1, 121.2. ^19^F NMR (376 MHz, DMSO-*d*_6_) *δ*: −154.9. HRMS (ESI): Calcd. for C_15_H_10_Cl_2_FN_4_ [M + H]^+^, 335.0261, Found 335.0257.

2-Chloro-5-fluoro*-N-*(2-methyl-4-(pyridin-4-yl)phenyl)pyrimidin-4-amine (**10i**). Yield 63%, pale yellow solid, mp: 213‒214 °C. ^1^H NMR (400 MHz, DMSO-*d*_6_) *δ*: 9.79 (s, 1H), 8.86–8.54 (m, 2H), 8.29 (d, *J* = 3.7 Hz, 1H), 7.81–7.65 (m, 4H), 7.53–7.37 (m, 1H), 2.28 (s, 3H). ^13^C NMR (100 MHz, DMSO-*d*_6_) *δ*: 153.3 (d, *J* = 3.0 Hz), 152.3 (d, *J* = 12.3 Hz), 150.2, 146.4, 145.3 (d, *J* = 257.4 Hz), 141.5 (d, *J* = 20.6 Hz), 136.4, 135.3, 135.1, 129.0, 127.6, 124.7, 121.1, 18.0. ^19^F NMR (376 MHz, DMSO-*d*_6_) *δ*: −155.0. HRMS (ESI): Calcd. for C_16_H_13_ClFN_4_ [M + H]^+^, 315.0807, Found 315.0809.

2-Chloro-5-fluoro*-N-*(2-methoxy-4-(pyridin-4-yl)phenyl)pyrimidin-4-amine (**10j**). Yield 55%, yellow solid, mp: 207‒208 °C. ^1^H NMR (400 MHz, DMSO-*d*_6_) *δ*: 9.39 (s, 1H), 8.65 (dd, *J* = 4.8, 2.7 Hz, 2H), 8.30 (d, *J* = 3.4 Hz, 1H), 7.82–7.64 (m, 3H), 7.62–7.37 (m, 2H), 3.93 (s, 3H). ^13^C NMR (100 MHz, DMSO-*d*_6_) *δ*: 153.2, 153.1 (d, *J* = 3.2 Hz), 151.9 (d, *J* = 11.7 Hz), 150.1, 146.5, 145.3 (d, *J* = 257.8 Hz), 141.6 (d, *J* = 20.7 Hz), 135.7, 126.6, 125.9, 121.2, 118.9, 110.4, 56.0. ^19^F NMR (376 MHz, DMSO-*d*_6_) *δ*: −155.0. HRMS (ESI): Calcd. for C_16_H_13_ClFN_4_O [M + H]^+^, 331.0756, Found 331.0756.

2-Chloro*-N-*(2,6-difluoro-4-(pyridin-4-yl)phenyl)-5-fluoropyrimidin-4-amine (**10k**). Yield 37%, white solid, mp: 241‒242 °C. ^1^H NMR (400 MHz, DMSO-*d*_6_) *δ*: 10.18 (s, 1H), 8.74–8.60 (m, 2H), 8.49–8.36 (m, 1H), 7.96–7.72 (m, 4H). ^13^C NMR (100 MHz, DMSO-*d*_6_) *δ*: 158.4 (dd, *J* = 249.2, 5.6 Hz), 153.3 (d, *J* = 3.0 Hz), 152.0 (d, *J* = 12.4 Hz), 150.4, 145.4 (d, *J* = 260.6 Hz), 144.1, 142.6 (d, *J* = 20.4 Hz), 137.8 (t, *J* = 9.6 Hz), 121.2, 114.3 (t, *J* = 16.9 Hz), 110.7 (d, *J* = 24.2 Hz). ^19^F NMR (376 MHz, DMSO-*d*_6_) *δ*: −116.0, −155.0. HRMS (ESI): Calcd. for C_15_H_9_ClF_3_N_4_ [M + H]^+^, 337.0462, Found 337.0468.

2-Chloro*-N-*(2,6-dimethyl-4-(pyridin-4-yl)phenyl)-5-fluoropyrimidin-4-amine (**10l**). Yield 44%, yellow solid, mp: 183‒185 °C. ^1^H NMR (400 MHz, DMSO-*d*_6_) *δ*: 9.69 (s, 1H), 8.75–8.58 (m, 2H), 8.28 (d, *J* = 5.4 Hz, 1H), 7.74 (d, *J* = 5.1 Hz, 2H), 7.61 (s, 2H), 2.22 (s, 6H). ^13^C NMR (100 MHz, DMSO-*d*_6_) *δ*: 153.6 (d, *J* = 3.0 Hz), 152.5 (d, *J* = 12.7 Hz), 150.2, 146.5, 145.2 (d, *J* = 256.7 Hz), 141.1 (d, *J* = 21.8 Hz), 136.7, 135.9, 135.2, 126.5, 121.2, 18.2. ^19^F NMR (376 MHz, DMSO-*d*_6_) *δ*: −155.7. HRMS (ESI): Calcd. for C_17_H_15_ClFN_4_ [M + H]^+^, 329.0964, Found 329.0960.

4′-((2,5-Dichloropyrimidin-4-yl)amino)-3′-fluoro-[1,1′-biphenyl]-4-carbonitrile (**10m**). Yield 57%, white solid, mp: 227‒229 °C. ^1^H NMR (400 MHz, DMSO-*d*_6_) *δ*: 9.69 (s, 1H), 8.42 (s, 1H), 8.00–7.91 (m, 4H), 7.86–7.77 (m, 1H), 7.73–7.64 (m, 1H), 7.64–7.54 (m, 1H). ^13^C NMR (100 MHz, DMSO-*d*_6_) *δ*: 157.8, 157.1, 156.9 (d, *J* = 248.5 Hz), 155.6, 142.6, 138.1 (d, *J* = 7.7 Hz), 132.9, 128.7, 127.6, 125.2 (d, *J* = 12.4 Hz), 123.1 (d, *J* = 3.1 Hz), 118.7, 114.6 (d, *J* = 21.5 Hz), 113.5, 110.6. ^19^F NMR (376 MHz, DMSO-*d*_6_) *δ*: −117.9. HRMS (ESI): Calcd. for C_17_H_10_Cl_2_FN_4_ [M + H]^+^, 359.0261, Found 359.0254.

3′-Chloro-4′-((2,5-dichloropyrimidin-4-yl)amino)-[1,1′-biphenyl]-4-carbonitrile (**10n**). Yield 47%, white solid, mp: 269‒271 °C. ^1^H NMR (400 MHz, DMSO-*d*_6_) *δ*: 9.66 (s, 1H), 8.43 (s, 1H), 8.03–7.92 (m, 5H), 7.87–7.80 (m, 1H), 7.67 (d, *J* = 8.3 Hz, 1H). ^13^C NMR (100 MHz, DMSO-*d*_6_) *δ*: 157.9, 157.1, 155.6, 142.4, 138.1, 134.8, 132.9, 131.3, 129.4, 128.1, 127.7, 126.4, 118.7, 113.5, 110.7. HRMS (ESI): Calcd. for C_17_H_10_Cl_3_N_4_ [M + H]^+^, 374.9966, Found 374.9957.

4′-((2,5-Dichloropyrimidin-4-yl)amino)-3′-methyl-[1,1′-biphenyl]-4-carbonitrile (**10o**). Yield 51%, white solid, mp: 207‒208 °C. ^1^H NMR (400 MHz, DMSO-*d*_6_) *δ*: 9.46 (s, 1H), 8.36 (s, 1H), 8.00–7.89 (m, 4H), 7.74 (d, *J* = 2.2 Hz, 1H), 7.70–7.62 (m, 1H), 7.41 (d, *J* = 8.2 Hz, 1H), 2.24 (s, 3H).^13^C NMR (100 MHz, DMSO-*d*_6_) *δ*: 158.1, 157.2, 155.1, 144.0, 136.6, 136.4, 135.5, 132.8, 129.2, 128.1, 127.5, 125.0, 118.9, 113.2, 110.0, 17.9. HRMS (ESI): Calcd. for C_18_H_13_Cl_2_N_4_ [M + H]^+^, 355.0512, Found 355.0510.

4′-((2,5-Dichloropyrimidin-4-yl)amino)-3′-methoxy-[1,1′-biphenyl]-4-carbonitrile (**10p**). Yield 56%, white solid, mp: 233‒234 °C. ^1^H NMR (400 MHz, DMSO-*d*_6_) *δ*: 8.99 (s, 1H), 8.41 (s, 1H), 7.96 (q, *J* = 8.4 Hz, 4H), 7.80 (d, *J* = 8.2 Hz, 1H), 7.52–7.37 (m, 2H), 3.95 (s, 3H). ^13^C NMR (100 MHz, DMSO-*d*_6_) *δ*: 157.1, 157.0, 155.1, 152.4, 144.1, 136.4, 132.7, 127.6, 126.6, 124.9, 119.3, 118.9, 113.7, 110.5, 110.0, 56.2. HRMS (ESI): Calcd. for C_18_H_13_Cl_2_N_4_O [M + H]^+^, 371.0461, Found 371.0454.

4′-((2,5-Dichloropyrimidin-4-yl)amino)-3′,5′-difluoro-[1,1′-biphenyl]-4-carbonitrile (**10q**). Yield 37%, white solid, mp: 242‒244 °C. ^1^H NMR (400 MHz, DMSO-*d*_6_) *δ*: 9.72 (s, 1H), 8.47 (s, 1H), 8.17–7.92 (m, 4H), 7.77 (d, *J* = 9.1 Hz, 2H). ^13^C NMR (100 MHz, DMSO-*d*_6_) *δ*: 158.5 (dd, *J* = 243.6, 5.5 Hz), 158.1, 157.2, 156.0, 141.5, 139.0 (t, *J* = 9.8 Hz), 132.9, 127.8, 118.6, 114.3 (t, *J* = 16.9 Hz), 113.4, 111.3, 110.8 (d, *J* = 24.1 Hz). ^19^F NMR (376 MHz, DMSO-*d*_6_) *δ*: −116.2. HRMS (ESI): Calcd. for C_17_H_9_Cl_2_F_2_N_4_ [M + H]^+^, 377.0167, Found 377.0169.

4′-((2,5-Dichloropyrimidin-4-yl)amino)-3′,5′-dimethyl-[1,1′-biphenyl]-4-carbonitrile (**10r**). Yield 41%, white solid, mp: 215‒217 °C. ^1^H NMR (400 MHz, DMSO-*d*_6_) *δ*: 9.36 (s, 1H), 8.35 (s, 1H), 8.16–7.80 (m, 4H), 7.57 (s, 2H), 2.19 (s, 6H). ^13^C NMR (100 MHz, DMSO-*d*_6_) *δ*: 158.1, 157.5, 154.9, 144.1, 137.0, 136.7, 135.4, 132.8, 127.5, 126.7, 118.9, 112.9, 110.0, 18.1. HRMS (ESI): Calcd. for C_19_H_15_Cl_2_N_4_ [M + H]^+^, 369.0668, Found 369.0664.

2,5-Dichloro*-N-*(2-fluoro-4-(pyridin-4-yl)phenyl)pyrimidin-4-amine (**10s**). Yield 58%, pale yellow solid, mp: 126‒128 °C. ^1^H NMR (400 MHz, DMSO-*d*_6_) *δ*: 9.72 (s, 1H), 8.68–8.62 (m, 2H), 8.43 (s, 1H), 7.92–7.83 (m, 1H), 7.83–7.78 (m, 2H), 7.77–7.71 (m, 1H), 7.67–7.57 (m, 1H). ^13^C NMR (100 MHz, DMSO-*d*_6_) *δ*: 157.8, 157.1, 157.0 (d, *J* = 248.5 Hz), 155.7, 150.3, 145.1, 137.0 (d, *J* = 7.6 Hz), 128.8, 125.7 (d, *J* = 12.4 Hz), 122.8 (d, *J* = 3.2 Hz), 121.1, 114.4 (d, *J* = 21.5 Hz), 113.6. ^19^F NMR (376 MHz, DMSO-*d*_6_) *δ*: −117.8. HRMS (ESI): Calcd. for C_15_H_10_Cl_2_FN_4_ [M + H]^+^, 335.0261, Found 335.0261.

2,5-Dichloro*-N-*(2-chloro-4-(pyridin-4-yl)phenyl)pyrimidin-4-amine (**10t**). Yield 62%, pale yellow solid, mp: 157‒159 °C. ^1^H NMR (400 MHz, DMSO-*d*_6_) *δ*: 9.68 (s, 1H), 8.71–8.63 (m, 2H), 8.44 (s, 1H), 8.07 (d, *J* = 2.1 Hz, 1H), 7.96–7.79 (m, 3H), 7.69 (d, *J* = 8.3 Hz, 1H). ^13^C NMR (100 MHz, DMSO-*d*_6_) *δ*: 157.9, 157.1, 155.6, 150.3, 145.0, 137.0, 135.3, 131.3, 129.5, 127.9, 126.2, 121.2, 113.5. HRMS (ESI): Calcd. for C_15_H_10_Cl_3_N_4_ [M + H]^+^, 350.9966, Found 350.9970.

2,5-Dichloro*-N-*(2-methyl-4-(pyridin-4-yl)phenyl)pyrimidin-4-amine (**10u**). Yield 51%, pale yellow solid, mp: 186‒188 °C. ^1^H NMR (400 MHz, DMSO-*d*_6_) *δ*: 9.47 (s, 1H), 8.68–8.60 (m, 2H), 8.37 (s, 1H), 7.86–7.63 (m, 4H), 7.43 (d, *J* = 8.2 Hz, 1H), 2.25 (s, 3H). ^13^C NMR (100 MHz, DMSO-*d*_6_) *δ*: 158.1, 157.2, 155.1, 150.2, 146.3, 136.8, 135.5, 128.9, 128.2, 124.7, 121.1, 113.25, 17.9. HRMS (ESI): Calcd. for C_16_H_13_Cl_2_N_4_ [M + H]^+^, 331.0512, Found 331.0513.

2,5-Dichloro*-N-*(2-methoxy-4-(pyridin-4-yl)phenyl)pyrimidin-4-amine (**10v**). Yield 69%, pale yellow solid, mp: 171‒172 °C. ^1^H NMR (400 MHz, DMSO-*d*_6_) *δ*: 8.99 (s, 1H), 8.69–8.61 (m, 2H), 8.41 (s, 1H), 7.88–7.76 (m, 3H), 7.60–7.43 (m, 2H), 3.96 (s, 3H). ^13^C NMR (100 MHz, DMSO-*d*_6_) *δ*: 157.1, 157.0, 155.1, 152.4, 150.1, 146.4, 135.3, 127.0, 124.8, 121.1, 119.0, 113.8, 110.2, 56.2. HRMS (ESI): Calcd. for C_16_H_13_Cl_2_N_4_O [M + H]^+^, 347.0461, Found 347.0460.

2,5-Dichloro*-N-*(2,6-difluoro-4-(pyridin-4-yl)phenyl)pyrimidin-4-amine (**10w**). Yield 37%, pale yellow solid, mp: 164‒166 °C. ^1^H NMR (400 MHz, DMSO-*d*_6_) *δ*: 9.74 (s, 1H), 9.02–8.60 (m, 2H), 8.48 (s, 1H), 7.99–7.58 (m, 4H). ^13^C NMR (100 MHz, DMSO-*d*_6_) *δ*: 158.5 (dd, *J* = 249.4, 5.4 Hz), 158.1, 157.2, 156.0, 150.4, 144.1 (d, *J* = 2.4 Hz), 138.1 (t, *J* = 9.7 Hz), 121.2, 114.7 (t, *J* = 16.8 Hz), 113.4, 110.6 (d, *J* = 24.2 Hz). ^19^F NMR (376 MHz, DMSO-*d*_6_) *δ*: −116.1. HRMS (ESI): Calcd. for C_15_H_9_Cl_2_F_2_N_4_ [M + H]^+^, 353.0167, Found 353.0170.

2,5-Dichloro*-N-*(2,6-dimethyl-4-(pyridin-4-yl)phenyl)pyrimidin-4-amine (**10x**). Yield 25%, pale yellow solid, mp: 129‒131 °C. ^1^H NMR (400 MHz, DMSO-*d*_6_) *δ*: 9.37 (s, 1H), 8.66–8.61 (m, 2H), 8.35 (s, 1H), 7.77–7.71 (m, 2H), 7.62 (s, 2H), 2.20 (s, 6H). ^13^C NMR (100 MHz, DMSO-*d*_6_) *δ*: 158.1, 157.5, 154.9, 150.2, 146.5, 136.8, 135.9, 135.8, 126.4, 121.2, 112.9, 18.0. HRMS (ESI): Calcd. for C_17_H_15_Cl_2_N_4_ [M + H]^+^, 345.0668, Found 345.0667.

#### 4.1.3 General procedure for the preparation of target compounds **A1**–**A6**, **A13**–**A18**, **B1**–**B5** and **B12**–**B16**

Under nitrogen atmosphere, a mixture of Pd(OAc)_2_ (0.1 mmol, 0.2 equiv.) and BINAP (0.2 mmol, 0.4 equiv.) was stirred in dry 1,4-dioxane (5 mL) at room temperature for 15 min and then transferred to a solution of 4-cyanoaniline (0.5 mmol, 1.0 equiv.), Cs_2_CO_3_ (1.0 mmol, 2.0 equiv.) and appropriate intermediates **9a**–**9v** (0.5 mmol, 1.0 equiv.) in dry 1,4-dioxane (20 mL). The reaction mixture was stirred at 110 °C for 2–4 h (monitored by TLC) under a nitrogen atmosphere. Once completed, the solution was cooled to room temperature, then evaporated under reduced pressure, diluted with water, and extracted with ethyl acetate (10 mL × 3). The separated organic layers were washed with saturated NaCl aq. (10 mL × 2), dried over anhydrous Na_2_SO_4_, filtered and concentrated to afford the crude product, which was subsequently purified by silica gel column chromatography, eluting with ethyl acetate/petroleum ether, to give target compounds **A1**–**A6**, **A13**–**A18**, **B1**–**B5** and **B12**–**B16**.

4′-((2-((4-Cyanophenyl)amino)-5-fluoropyrimidin-4-yl)oxy)-3′-fluoro-[1,1′-biphenyl]-4-carbonitrile (**A1**). Yield 48%, white solid, mp: 245‒246 °C. ^1^H NMR (400 MHz, DMSO-*d*_6_) *δ*: 10.20 (s, 1H), 8.66 (d, *J* = 2.8 Hz, 1H), 8.06–7.96 (m, 5H), 7.83–7.75 (m, 1H), 7.72–7.65 (m, 1H), 7.63–7.56 (m, 2H), 7.52–7.45 (m, 2H). ^13^C NMR (100 MHz, DMSO-*d*_6_) *δ*: 156.3 (d, *J* = 11.5 Hz), 154.2 (d, *J* = 3.8 Hz), 154.1 (d, *J* = 247.7 Hz), 146.4 (d, *J* = 19.5 Hz), 144.3, 142.4 (d, *J* = 1.9 Hz), 140.1 (d, *J* = 251.5 Hz), 139.0 (d, *J* = 12.8 Hz), 138.2 (d, *J* = 7.1 Hz), 133.0, 132.7, 127.8, 125.1, 124.1 (d, *J* = 3.1 Hz), 119.3, 118.7, 118.0, 115.7 (d, *J* = 19.6 Hz), 110.8, 102.7. ^19^F NMR (376 MHz, DMSO-*d*_6_) *δ*: −127.7, −165.0. IR: 3273, 3191, 3041, 2229, 1612, 1532, 1494, 1437, 1219, 1178, 836, 827, 768 cm^−1^. HRMS (ESI): Calcd. for C_24_H_14_F_2_N_5_O [M + H]^+^, 426.1161, Found 426.1157. HPLC purity: 98.02%.

3′-Chloro-4′-((2-((4-cyanophenyl)amino)-5-fluoropyrimidin-4-yl)oxy)-[1,1′-biphenyl]-4-carbonitrile (**A2**). Yield 60%, white solid, mp: 262‒264 °C. ^1^H NMR (400 MHz, DMSO-*d*_6_) *δ*: 10.20 (s, 1H), 8.67 (d, *J* = 2.7 Hz, 1H), 8.14 (d, *J* = 2.2 Hz, 1H), 8.09–7.88 (m, 5H), 7.78–7.67 (m, 1H), 7.65–7.52 (m, 2H), 7.46 (d, *J* = 8.6 Hz, 2H).^13^C NMR (100 MHz, DMSO-*d*_6_) *δ*: 156.3 (d, *J* = 11.6 Hz), 154.2 (d, *J* = 3.8 Hz), 147.9, 146.4 (d, *J* = 19.8 Hz), 144.3, 142.3, 140.2 (d, *J* = 251.4 Hz), 138.0, 133.0, 132.6, 129.0, 127.8, 127.5, 127.0, 125.1, 119.2, 118.7, 118.0, 110.8, 102.7. ^19^F NMR (376 MHz, DMSO-*d*_6_) *δ*: −164.9. IR: 3272, 3186, 3100, 2227, 1609, 1524, 1434, 1218, 1208, 1177, 835, 826, 768 cm^−1^. HRMS (ESI): Calcd. for C_24_H_14_ClFN_5_O [M + H]^+^, 442.0865, Found 442.0867. HPLC purity: 95.05%.

4′-((2-((4-Cyanophenyl)amino)-5-fluoropyrimidin-4-yl)oxy)-3′-methyl-[1,1′-biphenyl]-4-carbonitrile (**A3**). Yield 52%, white solid, mp: 251‒252 °C. ^1^H NMR (400 MHz, DMSO-*d*_6_) *δ*: 10.11 (s, 1H), 8.60 (d, *J* = 2.9 Hz, 1H), 8.18–7.91 (m, 4H), 7.83 (d, *J* = 2.4 Hz, 1H), 7.78–7.71 (m, 1H), 7.60–7.55 (m, 2H), 7.50–7.38 (m, 3H), 2.23 (s, 3H). ^13^C NMR (100 MHz, DMSO-*d*_6_) *δ*: 156.9 (d, *J* = 11.3 Hz), 154.3 (d, *J* = 3.8 Hz), 150.9, 145.8 (d, *J* = 19.7 Hz), 144.4, 143.8, 140.5 (d, *J* = 251.0 Hz), 136.3, 132.9, 132.6, 131.2, 130.1, 127.5, 126.2, 123.1, 119.3, 118.8, 117.9, 110.1, 102.5, 15.9. ^19^F NMR (376 MHz, DMSO-*d*_6_) *δ*: −164.8. IR: 3279, 2227, 1607, 1574, 1434, 1229, 1176, 1120, 836, 777 cm^−1^. HRMS (ESI): Calcd. for C_25_H_17_FN_5_O [M + H]^+^, 422.1412, Found 422.1413. HPLC purity: 98.19%.

4′-((2-((4-Cyanophenyl)amino)-5-fluoropyrimidin-4-yl)oxy)-3′-methoxy-[1,1′-biphenyl]-4-carbonitrile (**A4**). Yield 42%, white solid, mp: 252‒254 °C. ^1^H NMR (400 MHz, DMSO-*d*_6_) *δ*: 10.14 (s, 1H), 8.59 (d, *J* = 2.9 Hz, 1H), 8.11–7.91 (m, 4H), 7.73–7.49 (m, 3H), 7.48–7.35 (m, 4H), 3.86 (s, 3H). ^13^C NMR (100 MHz, DMSO-*d*_6_) *δ*: 156.9 (d, *J* = 11.3 Hz), 154.2 (d, *J* = 3.7 Hz), 151.5, 145.7 (d, *J* = 20.0 Hz), 144.4, 144.0, 140.8, 140.3 (d, *J* = 250.9 Hz), 137.7, 132.8, 132.6, 127.8, 123.7, 119.8, 119.3, 118.8, 117.8, 112.1, 110.3, 102.4, 56.1. ^19^F NMR (376 MHz, DMSO-*d*_6_) *δ*: −165.0. IR: 3272, 3187, 3100, 2228, 1606, 1493, 1430, 1294, 1233, 1164, 1114, 1021, 825, 770 cm^−1^. HRMS (ESI): Calcd. for C_25_H_17_FN_5_O_2_ [M + H]^+^, 438.1361, Found 438.1369. HPLC purity: 98.44%.

4′-((2-((4-Cyanophenyl)amino)-5-fluoropyrimidin-4-yl)oxy)-3′,5′-difluoro-[1,1′-biphenyl]-4-carbonitrile (**A5**). Yield 70%, white solid, mp: 253‒254 °C. ^1^H NMR (400 MHz, DMSO-*d*_6_) *δ*: 10.29 (s, 1H), 8.72 (d, *J* = 2.6 Hz, 1H), 8.10–7.90 (m, 6H), 7.72–7.42 (m, 4H). ^13^C NMR (100 MHz, DMSO-*d*_6_) *δ*: 155.3 (d, *J* = 11.7 Hz), 155.0 (dd, *J* = 248.8, 4.7 Hz), 154.2 (d, *J* = 3.7 Hz), 147.2 (d, *J* = 19.3 Hz), 144.1, 141.3 (d, *J* = 2.4 Hz), 139.7 (d, *J* = 252.1 Hz), 138.0 (t, *J* = 9.1 Hz), 133.0, 132.7, 127.8, 127.4 (t, *J* = 16.0 Hz), 119.2, 118.5, 118.1, 111.7 (d, *J* = 22.2 Hz), 111.4, 103.0. ^19^F NMR (376 MHz, DMSO-*d*_6_) *δ*: −125.4, −165.2. IR: 3258, 3185, 2921, 2222, 1601, 1524, 1432, 1218, 1174, 1046, 833, 771 cm^−1^. HRMS (ESI): Calcd. for C_24_H_13_F_3_N_5_O [M + H]^+^, 444.1067, Found 444.1066. HPLC purity: 96.93%.

4′-((2-((4-Cyanophenyl)amino)-5-fluoropyrimidin-4-yl)oxy)-3′,5′-dimethyl-[1,1′-biphenyl]-4-carbonitrile (**A6**). Yield 51%, white solid, mp: 254‒255 °C. ^1^H NMR (400 MHz, DMSO-*d*_6_) *δ*: 10.14 (s, 1H), 8.63 (d, *J* = 2.9 Hz, 1H), 8.17–7.83 (m, 4H), 7.67 (s, 2H), 7.59–7.51 (m, 2H), 7.47–7.35 (m, 2H), 2.19 (s, 6H). ^13^C NMR (100 MHz, DMSO-*d*_6_) *δ*: 156.1 (d, *J* = 11.5 Hz), 154.4 (d, *J* = 3.8 Hz), 149.5, 145.9 (d, *J* = 19.8 Hz), 144.4, 143.9, 140.2 (d, *J* = 251.0 Hz), 136.1, 132.8, 132.5, 131.2, 127.6, 127.4, 119.2, 118.8, 117.8, 110.0, 102.4, 16.0. ^19^F NMR (376 MHz, DMSO-*d*_6_) *δ*: −165.1. IR: 3342, 2220, 1604, 1519, 1426, 1294, 1146, 1025, 830, 772 cm^−1^. HRMS (ESI): Calcd. for C_26_H_19_FN_5_O [M + H]^+^, 436.1568, Found 436.1561. HPLC purity: 99.41%.

4-((5-Fluoro-4-(2-fluoro-4-(pyridin-4-yl)phenoxy)pyrimidin-2-yl)amino)benzonitrile (**A13**). Yield 28%, pale yellow solid, mp: 290‒291 °C. ^1^H NMR (400 MHz, DMSO-*d*_6_) *δ*: 10.21 (s, 1H), 8.77–8.64 (m, 3H), 8.11–7.99 (m, 1H), 7.89–7.81 (m, 3H), 7.78–7.56 (m, 3H), 7.52–7.44 (m, 2H). ^13^C NMR (100 MHz, DMSO-*d*_6_) *δ*: 156.3 (d, *J* = 11.6 Hz), 154.21, 154.18 (d, *J* = 247.7 Hz), 150.4, 146.4 (d, *J* = 19.7 Hz), 144.9, 144.3, 140.1 (d, *J* = 251.5 Hz), 139.4 (d, *J* = 12.4 Hz), 137.2, 132.7, 125.2, 123.9, 121.3, 119.3, 118.0, 115.5 (d, *J* = 19.2 Hz), 102.7. ^19^F NMR (376 MHz, DMSO-*d*_6_) *δ*: −127.7, −165.0. IR: 2919, 2849, 2220, 1601, 1450, 1413, 1281, 1227, 1190, 842, 820 cm^−1^. HRMS (ESI): Calcd. for C_22_H_14_F_2_N_5_O [M + H]^+^, 402.1161, Found 402.1167. HPLC purity: 98.50%.

4-((4-(2-Chloro-4-(pyridin-4-yl)phenoxy)-5-fluoropyrimidin-2-yl)amino)benzonitrile (**A14**). Yield 24%, white solid, mp: 278‒280 °C. ^1^H NMR (400 MHz, DMSO-*d*_6_) *δ*: 10.19 (s, 1H), 8.75–8.64 (m, 3H), 8.18 (d, *J* = 2.1 Hz, 1H), 8.04–7.96 (m, 1H), 7.89–7.83 (m, 2H), 7.72 (d, *J* = 8.4 Hz, 1H), 7.62–7.55 (m, 2H), 7.50–7.42 (m, 2H). ^13^C NMR (100 MHz, DMSO-*d*_6_) *δ*: 156.3 (d, *J* = 11.7 Hz), 154.2 (d, *J* = 3.8 Hz), 150.4, 148.3, 146.4 (d, *J* = 19.6 Hz), 144.8, 144.2, 140.2 (d, *J* = 251.5 Hz), 137.0, 132.6, 128.8, 127.3, 127.1, 125.2, 121.3, 119.2, 118.0, 102.7. ^19^F NMR (376 MHz, DMSO-*d*_6_) *δ*: −165.0. IR: 2921, 2219, 1600, 1539, 1444, 1410, 1338, 1281, 1226, 1173, 1057, 842, 811, 772 cm^−1^. HRMS (ESI): Calcd. for C_22_H_14_ClFN_5_O [M + H]^+^, 418.0865, Found 418.0874. HPLC purity: 95.45%.

4-((5-Fluoro-4-(2-methyl-4-(pyridin-4-yl)phenoxy)pyrimidin-2-yl)amino)benzonitrile (**A15**). Yield 42%, white solid, mp: 277–278 °C. ^1^H NMR (400 MHz, DMSO-*d*_6_) *δ*: 10.11 (s, 1H), 8.71–8.55 (m, 3H), 7.90–7.75 (m, 4H), 7.60–7.53 (m, 2H), 7.53–7.37 (m, 3H), 2.23 (s, 3H). ^13^C NMR (100 MHz, DMSO-*d*_6_) *δ*: 156.9 (d, *J* = 11.3 Hz), 154.3 (d, *J* = 3.7 Hz), 151.3, 150.3, 146.2, 145.9 (d, *J* = 19.8 Hz), 144.4, 140.6 (d, *J* = 251.0 Hz), 135.2, 132.6, 131.2, 129.9, 126.0, 123.2, 121.2, 119.3, 117.95, 102.5, 15.9. ^19^F NMR (376 MHz, DMSO-*d*_6_) *δ*: −164.8. IR: 2923, 2213, 1602, 1538, 1436, 1404, 1340, 1271, 1221, 1175, 1118, 833, 821, 772 cm^−1^. HRMS (ESI): Calcd. for C_23_H_17_FN_5_O [M + H]^+^, 398.1412, Found 398.1417. HPLC purity: 98.53%.

4-((5-Fluoro-4-(2-methoxy-4-(pyridin-4-yl)phenoxy)pyrimidin-2-yl)amino)benzonitrile (**A16**). Yield 51%, pale yellow solid, mp: 264‒266 °C. ^1^H NMR (400 MHz, DMSO-*d*_6_) *δ*: 10.12 (s, 1H), 8.80–8.54 (m, 3H), 7.88–7.80 (m, 2H), 7.73–7.39 (m, 7H), 3.86 (s, 3H). ^13^C NMR (100 MHz, DMSO-*d*_6_) *δ*: 156.9 (d, *J* = 11.3 Hz), 154.2 (d, *J* = 3.8 Hz), 151.6, 150.3, 146.4, 145.8 (d, *J* = 19.9 Hz), 144.4, 141.1, 140.3 (d, *J* = 251.0 Hz), 136.6, 132.6, 123.8, 121.4, 119.6, 119.3, 117.8, 111.8, 102.4, 56.2. ^19^F NMR (376 MHz, DMSO-*d*_6_) *δ*: −165.0. IR: 3265, 2224, 1603, 1448, 1402, 1280, 1207, 1021, 820, 770 cm^−1^. HRMS (ESI): Calcd. for C_23_H_17_FN_5_O_2_ [M + H]^+^, 414.1361, Found 414.1362. HPLC purity: 97.96%.

4-((4-(2,6-Difluoro-4-(pyridin-4-yl)phenoxy)-5-fluoropyrimidin-2 yl)amino)benzonitrile (**A17**). Yield 26%, white solid, mp: 284–286 °C. ^1^H NMR (400 MHz, DMSO-*d*_6_) *δ*: 10.29 (s, 1H), 8.81–8.64 (m, 3H), 8.05–7.82 (m, 4H), 7.66–7.43 (m, 4H). ^13^C NMR (100 MHz, DMSO-*d*_6_) *δ*: 155.2 (d, *J* = 11.7 Hz), 155.1 (dd, *J* = 249.1, 4.5 Hz), 154.2 (d, *J* = 3.6 Hz), 150.5, 147.2 (d, *J* = 19.4 Hz), 144.05, 143.90, 139.7 (d, *J* = 252.0 Hz), 137.1 (t, *J* = 9.0 Hz), 132.7, 127.8 (t, *J* = 15.9 Hz), 121.2, 119.2, 118.1, 111.5 (d, *J* = 22.4 Hz), 103.0. ^19^F NMR (376 MHz, DMSO-*d*_6_) *δ*: −125.2, −165.1. IR: 2915, 2221, 1603, 1540, 1447, 1413, 1277, 1233, 1176, 1043, 845, 821, 772 cm^−1^. HRMS (ESI): Calcd. for C_22_H_13_F_3_N_5_O [M + H]^+^, 420.1067, Found 420.1062. HPLC purity: 99.45%.

4-((4-(2,6-Dimethyl-4-(pyridin-4-yl)phenoxy)-5-fluoropyrimidin-2-yl)amino)benzonitrile (**A18**). Yield 51%, white solid, mp: 332–334 °C. ^1^H NMR (400 MHz, DMSO-*d*_6_) *δ*: 10.12 (s, 1H), 8.71–8.59 (m, 3H), 7.86–7.69 (m, 4H), 7.53 (d, 2H), 7.42–7.15 (m, 2H), 2.19 (s, 6H). ^13^C NMR (100 MHz, DMSO-*d*_6_) *δ*: 156.1 (d, *J* = 11.3 Hz), 154.5 (d, *J* = 3.6 Hz), 150.2, 150.0, 146.5, 146.1 (d, *J* = 19.6 Hz), 144.5, 140.2 (d, *J* = 251.0 Hz), 135.0, 132.6, 131.4, 127.4, 121.2, 119.3, 117.9, 102.5, 16.1. ^19^F NMR (376 MHz, DMSO-*d*_6_) *δ*: −165.1. IR: 2920, 2221, 2212, 1602, 1540, 1445, 1412, 1281, 1229, 1190, 1154, 1011, 843, 820, 773 cm^−1^. HRMS (ESI): Calcd. for C_24_H_19_FN_5_O [M + H]^+^, 412.1568, Found 412.1566. HPLC purity: 96.63%.

4′-((5-Chloro-2-((4-cyanophenyl)amino)pyrimidin-4-yl)oxy)-3′-fluoro-[1,1′-biphenyl]-4-carbonitrile (**B1**). Yield 55%, white solid, mp: 258‒259 °C. ^1^H NMR (400 MHz, DMSO-*d*_6_) *δ*: 10.33 (s, 1H), 8.65 (s, 1H), 8.09–7.95 (m, 5H), 7.84–7.75 (m, 1H), 7.72–7.61 (m, 1H), 7.61–7.40 (m, 4H). ^13^C NMR (100 MHz, DMSO-*d*_6_) *δ*: 163.2, 158.6, 156.9, 154.0 (d, *J* = 247.6 Hz), 143.9, 142.4 (d, *J* = 1.9 Hz), 139.4 (d, *J* = 12.7 Hz), 138.1 (d, *J* = 7.1 Hz), 133.0, 132.6, 127.7, 125.1, 124.1 (d, *J* = 3.3 Hz), 119.2, 118.7, 118.6, 115.7 (d, *J* = 19.7 Hz), 110.8, 106.0, 103.1. ^19^F NMR (376 MHz, DMSO-*d*_6_) *δ*: −127.7. IR: 3314, 2920, 2239, 2223, 1602, 1577, 1528, 1516, 1447, 1419, 1407, 1295, 1176, 1130, 823, 774 cm^−1^. HRMS (ESI): Calcd. for C_24_H_14_ClFN_5_O [M + H]^+^, 442.0865, Found 442.0860. HPLC purity: 99.03%.

3′-Chloro-4′-((5-chloro-2-((4-cyanophenyl)amino)pyrimidin-4-yl)oxy)-[1,1′-biphenyl]-4-carbonitrile (**B2**). Yield 49%, white solid, mp: 264‒265 °C. ^1^H NMR (400 MHz, DMSO-*d*_6_) *δ*: 10.31 (s, 1H), 8.65 (s, 1H), 8.14 (d, *J* = 2.2 Hz, 1H), 8.06–7.97 (m, 4H), 7.95–7.91 (m, 1H), 7.67 (d, *J* = 8.5 Hz, 1H), 7.55–7.50 (m, 2H), 7.46–7.40 (m, 2H). ^13^C NMR (100 MHz, DMSO-*d*_6_) *δ*: 163.2, 158.6, 156.9, 148.3, 143.9, 142.3, 138.0, 133.0, 132.6, 128.9, 127.8, 127.5, 127.0, 125.2, 119.2, 118.7, 118.5, 110.8, 106.1, 103.1. IR: 3342, 2223, 1598, 1524, 1419, 1296, 1177, 1061, 825, 773 cm^−1^. HRMS (ESI): Calcd. for C_24_H_14_Cl_2_N_5_O [M + H]^+^, 458.0570, Found 458.0570. HPLC purity: 98.82%.

4′-((5-Chloro-2-((4-cyanophenyl)amino)pyrimidin-4-yl)oxy)-3′-methyl-[1,1′-biphenyl]-4-carbonitrile (**B3**). Yield 40%, white solid, mp: 253‒255 °C. ^1^H NMR (400 MHz, DMSO-*d*_6_) *δ*: 10.25 (s, 1H), 8.60 (s, 1H), 8.03–7.92 (m, 4H), 7.87–7.82 (m, 1H), 7.79–7.73 (m, 1H), 7.57–7.51 (m, 2H), 7.48–7.36 (m, 3H), 2.20 (s, 3H). ^13^C NMR (100 MHz, DMSO-*d*_6_) *δ*: 163.6, 158.2, 157.0, 151.3, 144.1, 143.8, 136.3, 132.9, 132.5, 131.0, 130.1, 127.5, 126.2, 123.2, 119.2, 118.8, 118.4, 110.1, 106.3, 102.9, 15.8. IR: 3266, 3045, 2224, 1604, 1578, 1505, 1407, 1289, 1212, 1166, 1121, 1019, 844, 818, 782 cm^−1^. HRMS (ESI): Calcd. for C_25_H_16_ClN_5_NaO [M + Na]^+^, 460.0936, Found 460.0938. HPLC purity: 98.26%.

4′-((5-Chloro-2-((4-cyanophenyl)amino)pyrimidin-4-yl)oxy)-3′-methoxy-[1,1′-biphenyl]-4-carbonitrile (**B4**). Yield 41%, white solid, mp: 302‒304 °C. ^1^H NMR (400 MHz, DMSO-*d*_6_) *δ*: 10.25 (s, 1H), 8.57 (s, 1H), 8.12–7.89 (m, 4H), 7.61 (d, *J* = 2.0 Hz, 1H), 7.56–7.50 (m, 2H), 7.48–7.37 (m, 4H), 3.84 (s, 3H). ^13^C NMR (100 MHz, DMSO-*d*_6_) *δ*: 163.7, 158.1, 157.0, 151.5, 144.1, 144.0, 141.2, 137.7, 132.9, 132.6, 127.8, 123.7, 119.8, 119.3, 118.9, 118.4, 112.1, 110.3, 106.1, 102.9, 56.2. IR: 3305, 2240, 2218, 1599, 1510, 1438, 1419, 1274, 1233, 1177, 1121, 1024, 845, 822, 773 cm^−1^. HRMS (ESI): Calcd. for C_25_H_17_ClN_5_O_2_ [M + H]^+^, 454.1065, Found 454.1066. HPLC purity: 97.70%.

4′-((5-Chloro-2-((4-cyanophenyl)amino)pyrimidin-4-yl)oxy)-3′,5′-dimethyl-[1,1′-biphenyl]-4-carbonitrile (**B5**). Yield 44%, white solid, mp: 269‒271 °C. ^1^H NMR (400 MHz, DMSO-*d*_6_) *δ*: 10.27 (s, 1H), 8.61 (s, 1H), 8.07–7.85 (m, 4H), 7.66 (s, 2H), 7.60–7.47 (m, 2H), 7.44–7.31 (m, 2H), 2.15 (s, 6H). ^13^C NMR (100 MHz, DMSO-*d*_6_) *δ*: 162.9, 158.3, 157.2, 150.1, 144.1, 143.9, 136.0, 132.9, 132.5, 131.1, 127.6, 127.5, 119.2, 118.9, 118.3, 110.1, 106.0, 102.9, 16.1. IR: 3297, 2240, 2217, 1580, 1509, 1416, 1274, 1176, 1149, 1093, 834, 776 cm^−1^. HRMS (ESI): Calcd. for C_26_H_19_ClN_5_O [M + H]^+^, 452.1273, Found 452.1274. HPLC purity: 98.03%, 4-((5-Chloro-4-(2-fluoro-4-(pyridin-4-yl)phenoxy)pyrimidin-2-yl)amino)benzonitrile (**B12**). Yield 44%, white solid, mp: 292‒294 °C. ^1^H NMR (400 MHz, DMSO-*d*_6_) *δ*: 10.31 (s, 1H), 8.71–8.60 (m, 3H), 8.09–7.97 (m, 1H), 7.90–7.78 (m, 3H), 7.75–7.62 (m, 1H), 7.59–7.51 (m, 2H), 7.46–7.40 (m, 2H). ^13^C NMR (100 MHz, DMSO-*d*_6_) *δ*: 163.2, 158.6, 156.9, 154.1 (d, *J* = 247.6 Hz), 150.4, 145.0 (d, *J* = 1.8 Hz), 143.9, 139.8 (d, *J* = 12.7 Hz), 137.1 (d, *J* = 6.9 Hz), 132.6, 125.2, 123.9 (d, *J* = 3.2 Hz), 121.3, 119.2, 118.6, 115.5 (d, *J* = 19.6 Hz), 106.0, 103.2. ^19^F NMR (376 MHz, DMSO-*d*_6_) *δ*: −127.7. IR: 3046, 2901, 2217, 1579, 1529, 1404, 1281, 1174, 1122, 816, 775 cm^−1^. HRMS (ESI): Calcd. for C_22_H_14_ClFN_5_O [M + H]^+^, 418.0865, Found 418.0862. HPLC purity: 99.12%.

4-((5-Chloro-4-(2-chloro-4-(pyridin-4-yl)phenoxy)pyrimidin-2-yl)amino)benzonitrile (**B13**). Yield 57%, white solid, mp: 274‒276 °C. ^1^H NMR (400 MHz, DMSO-*d*_6_) *δ*: 10.32 (s, 1H), 8.78–8.59 (m, 3H), 8.18 (d, *J* = 2.2 Hz, 1H), 8.09–7.92 (m, 1H), 7.92–7.81 (m, 2H), 7.75–7.65 (m, 1H), 7.57–7.38 (m, 4H). ^13^C NMR (100 MHz, DMSO-*d*_6_) *δ*: 163.2, 158.6, 156.8, 150.4, 148.7, 144.8, 143.9, 137.0, 132.6, 128.7, 127.3, 127.1, 125.2, 121.3, 119.1, 118.5, 106.1, 103.1. IR: 2918, 2219, 1580, 1528, 1505, 1407, 1282, 1217, 1171, 838, 810, 776 cm^−1^. HRMS (ESI): Calcd. for C_22_H_14_Cl_2_N_5_O [M + H]^+^, 434.0570, Found 434.0569. HPLC purity: 97.73%.

4-((5-Chloro-4-(2-methyl-4-(pyridin-4-yl)phenoxy)pyrimidin-2-yl)amino)benzonitrile (**B14**). Yield 55%, white solid, mp: 266‒267 °C. ^1^H NMR (400 MHz, DMSO-*d*_6_) *δ*: 10.26 (s, 1H), 8.71–8.59 (m, 3H), 7.94–7.76 (m, 4H), 7.59–7.36 (m, 5H), 2.20 (s, 3H). ^13^C NMR (100 MHz, DMSO-*d*_6_) *δ*: 163.5, 158.2, 157.0, 151.6, 150.3, 146.2, 144.0, 135.2, 132.5, 131.1, 129.8, 125.9, 123.2, 121.1, 119.2, 118.4, 106.3, 102.9, 15.8. IR: 2917, 2223, 1574, 1530, 1400, 1211, 1166, 1008, 843, 775 cm^−1^. HRMS (ESI): Calcd. for C_23_H_17_ClN_5_O [M + H]^+^, 414.1116, Found 414.1118. HPLC purity: 98.00%.

4-((5-Chloro-4-(2-methoxy-4-(pyridin-4-yl)phenoxy)pyrimidin-2-yl)amino)benzonitrile (**B15**). Yield 59%, white solid, mp: 273‒275 °C. ^1^H NMR (400 MHz, DMSO-*d*_6_) *δ*: 10.27 (s, 1H), 8.71–8.67 (m, 2H), 8.59 (s, 1H), 7.87–7.83 (m, 2H), 7.67 (d, *J* = 2.0 Hz, 1H), 7.56–7.48 (m, 3H), 7.48–7.39 (m, 3H), 3.85 (s, 3H). ^13^C NMR (100 MHz, DMSO-*d*_6_) *δ*: 163.6, 158.1, 156.9, 151.5, 150.3, 146.4, 144.1, 141.5, 136.5, 132.5, 123.7, 121.4, 119.5, 119.2, 118.3, 111.8, 106.0, 102.8, 56.2. IR: 2922, 2221, 1579, 1532, 1425, 1411, 1308, 1174, 1023, 819, 774 cm^−1^. HRMS (ESI): Calcd. for C_23_H_17_ClN_5_O_2_ [M + H]^+^, 430.1065, Found 430.1066. HPLC purity: 99.31%.

4-((5-Chloro-4-(2,6-dimethyl-4-(pyridin-4-yl)phenoxy)pyrimidin-2-yl)amino)benzonitrile (**B16**). Yield 77%, white solid, mp: 330‒332 °C. ^1^H NMR (400 MHz, DMSO-*d*_6_) *δ*: 10.27 (s, 1H), 8.78–8.50 (m, 3H), 7.89–7.65 (m, 4H), 7.68–7.31 (m, 4H), 2.17 (s, 6H). ^13^C NMR (100 MHz, DMSO-*d*_6_) *δ*: 162.9, 158.3, 157.1, 150.4, 150.3, 146.3, 144.1, 134.9, 132.5, 131.2, 127.3, 121.1, 119.2, 118.3, 105.9, 102.8, 16.0. IR: 2918, 2221, 1578, 1528, 1408, 1276, 1158, 840, 822, 777 cm^−1^. HRMS (ESI): Calcd. for C_24_H_19_ClN_5_O [M + H]^+^, 428.1273, Found 428.1269. HPLC purity: 97.37%.

#### 4.1.4 General procedure for the preparation of target compounds **A7**–**A12**, **A19**–**A24**, **B6**–**B11**, **B17**–**B22**

To a solution of **10a**–**10r** (1.0 mmol, 1.0 eq.) and 4-cyanoaniline (1.0 mmol, 1.0 eq.) in *n*-BuOH (25 mL) was added 10 drops of 12 mol/L HCl aq. The reaction mixture was then stirred at 100 °C for 5–8 h until complete consumption of starting material. After cooling to room temperature, the reaction solution was treated with saturated NaHCO_3_ aq. (10 mL), and stirred at room temperature for another 0.5 h. A white precipitate formed, which was collected by filtration, washed with H_2_O, and purified *via* silica gel column chromatography using methanol/dichloromethane as the eluent to provide **A7**–**A12**, **A19**–**A24**, **B6**–**B11**. The target compounds **B17**–**B22** were synthesized following the general procedure for **B1**–**B5**, taking intermediates **10s**–**10x** as the starting material.

4′-((2-((4-Cyanophenyl)amino)-5-fluoropyrimidin-4-yl)amino)-3′-fluoro-[1,1′-biphenyl]-4-carbonitrile (**A7**). Yield 26%, white solid, mp: 305‒307 °C. ^1^H NMR (400 MHz, DMSO-*d*_6_) *δ*: 9.77 (s, 1H), 9.54 (s, 1H), 8.22 (d, *J* = 3.3 Hz, 1H), 8.07–7.91 (m, 4H), 7.86 (d, *J* = 11.9 Hz, 1H), 7.80–7.65 (m, 4H), 7.61–7.48 (m, 2H). ^13^C NMR (100 MHz, DMSO-*d*_6_) *δ*: 156.8 (d, *J* = 247.3 Hz), 154.8 (d, *J* = 3.0 Hz), 150.5 (d, *J* = 11.8 Hz), 145.2, 142.8 (d, *J* = 1.8 Hz), 141.3 (d, *J* = 248.3 Hz), 141.1 (d, *J* = 19.5 Hz), 137.0 (d, *J* = 7.6 Hz), 133.0, 132.7, 128.2, 127.5, 126.3 (d, *J* = 12.4 Hz), 123.1 (d, *J* = 3.2 Hz), 119.6, 118.8, 117.7, 114.6 (d, *J* = 21.5 Hz), 110.5, 101.7. ^19^F NMR (376 MHz, DMSO-*d*_6_) *δ*: −118.5, −163.1. IR: 3431, 3044, 2228, 1649, 1532, 1436, 1220, 1179, 1114, 820, 767 cm^−1^. HRMS (ESI): Calcd. for C_24_H_15_F_2_N_6_ [M + H]^+^, 425.1321, Found 425.1319. HPLC purity: 95.96%.

3′-Chloro-4′-((2-((4-cyanophenyl)amino)-5-fluoropyrimidin-4-yl)amino)-[1,1′-biphenyl]-4-carbonitrile (**A8**). Yield 21%, pale yellow solid, mp: 265‒267 °C. ^1^H NMR (400 MHz, DMSO-*d*_6_) *δ*: 9.76 (s, 1H), 9.48 (s, 1H), 8.22 (s, 1H), 8.18–7.94 (m, 5H), 7.92–7.65 (m, 4H), 7.57–7.39 (m, 2H). ^13^C NMR (100 MHz, DMSO-*d*_6_) *δ*: 154.7 (d, *J* = 3.0 Hz), 150.7 (d, *J* = 11.5 Hz), 145.1, 142.6, 141.2 (d, *J* = 248.1 Hz), 141.0 (d, *J* = 19.6 Hz), 137.4, 135.7, 133.0, 132.6, 131.1, 129.3, 128.1, 127.6, 126.3, 119.6, 118.7, 117.7, 110.6, 101.6. ^19^F NMR (376 MHz, DMSO-*d*_6_) *δ*: −163.2. IR: 3399, 3038, 2227, 1638, 1590, 1527, 1437, 1221, 1176, 1052, 820, 766 cm^−1^. HRMS (ESI): Calcd. for C_24_H_15_ClFN_6_ [M + H]^+^, 441.1025, Found 441.1020. HPLC purity: 95.04%.

4′-((2-((4-Cyanophenyl)amino)-5-fluoropyrimidin-4-yl)amino)-3′-methyl-[1,1′-biphenyl]-4-carbonitrile (**A9**). Yield 24%, white solid, mp: 266‒268 °C. ^1^H NMR (400 MHz, DMSO-*d*_6_) *δ*: 9.68 (s, 1H), 9.27 (s, 1H), 8.16 (d, *J* = 3.5 Hz, 1H), 8.03–7.89 (m, 4H), 7.80–7.63 (m, 4H), 7.56–7.39 (m, 3H), 2.29 (s, 3H). ^13^C NMR (100 MHz, DMSO-*d*_6_) *δ*: 154.9 (d, *J* = 3.1 Hz), 151.1 (d, *J* = 11.7 Hz), 145.3, 144.2, 141.3 (d, *J* = 247.8 Hz), 140.3 (d, *J* = 19.5 Hz), 137.3, 135.9, 135.2, 132.9, 132.6, 129.2, 127.9, 127.3, 124.9, 119.6, 118.9, 117.6, 109.9, 101.4, 18.1. ^19^F NMR (376 MHz, DMSO-*d*_6_) *δ*: −163.3. IR: 3437, 2922, 2221, 1631, 1604, 1523, 1425, 1222, 1172, 824, 771 cm^−1^. HRMS (ESI): Calcd. for C_25_H_18_FN_6_ [M + H]^+^, 421.1571, Found 421.1566. HPLC purity: 95.32%.

4′-((2-((4-Cyanophenyl)amino)-5-fluoropyrimidin-4-yl)amino)-3′-methoxy-[1,1′-biphenyl]-4-carbonitrile (**A10**). Yield 28%, white solid, mp: 301‒303 °C. ^1^H NMR (400 MHz, DMSO-*d*_6_) *δ*: 9.79 (s, 1H), 8.71 (s, 1H), 8.19 (d, *J* = 3.5 Hz, 1H), 8.07–7.88 (m, 5H), 7.80 (d, *J* = 8.4 Hz, 2H), 7.67–7.37 (m, 4H), 3.94 (s, 3H). ^13^C NMR (100 MHz, DMSO-*d*_6_) *δ*: 154.7 (d, *J* = 3.3 Hz), 152.4, 150.3 (d, *J* = 10.8 Hz), 145.2, 144.3, 141.4 (d, *J* = 248.3 Hz), 140.4 (d, *J* = 19.8 Hz), 135.6, 132.8, 132.7, 127.5, 127.4, 125.0, 119.7, 119.1, 118.9, 117.7, 110.2, 109.8, 101.6, 56.0. ^19^F NMR (376 MHz, DMSO-*d*_6_) *δ*: −163.5. IR: 3400, 3037, 2218, 1638, 1581, 1535, 1436, 1170, 1030, 840, 799 cm^−1^. HRMS (ESI): Calcd. for C_25_H_18_FN_6_O [M + H]^+^, 437.1521, Found 437.1522. HPLC purity: 95.32%.

4′-((2-((4-Cyanophenyl)amino)-5-fluoropyrimidin-4-yl)amino)-3′,5′-difluoro-[1,1′-biphenyl]-4-carbonitrile (**A11**). Yield 29%, white solid, mp: 309‒310 °C. ^1^H NMR (400 MHz, DMSO-*d*_6_) *δ*: 9.77 (s, 1H), 9.58 (s, 1H), 8.24 (d, *J* = 3.8 Hz, 1H), 8.19–7.95 (m, 4H), 7.95–7.63 (m, 4H), 7.57–7.37 (m, 2H). ^13^C NMR (100 MHz, DMSO-*d*_6_) *δ*: 158.8 (dd, *J* = 248.4, 5.7 Hz), 154.9 (d, *J* = 3.1 Hz), 150.8 (d, *J* = 12.1 Hz), 145.1, 141.7, 141.5 (d, *J* = 19.0 Hz), 141.3 (d, *J* = 247.9 Hz), 138.4 (t, *J* = 9.8 Hz), 133.0, 132.6, 127.7, 119.5, 118.6, 117.7, 115.2 (t, *J* = 17.1 Hz), 111.2, 110.8 (d, *J* = 24.4 Hz), 101.8. ^19^F NMR (376 MHz, DMSO-*d*_6_) *δ*: −116.3, −163.7. IR: 3346, 2226, 2212, 1607, 1580, 1525, 1428, 1223, 1173, 1061, 1020, 831, 775 cm^−1^. HRMS (ESI): Calcd. for C_24_H_14_F_3_N_6_ [M + H]^+^, 443.1227, Found 443.1225. HPLC purity: 95.16%.

4′-((2-((4-Cyanophenyl)amino)-5-fluoropyrimidin-4-yl)amino)-3′,5′-dimethyl-[1,1′-biphenyl]-4-carbonitrile (**A12**). Yield 39%, white solid, mp: 301‒302 °C. ^1^H NMR (400 MHz, DMSO-*d*_6_) *δ*: 9.63 (s, 1H), 9.18 (s, 1H), 8.13 (d, *J* = 5.6 Hz, 1H), 7.99–7.90 (m, 4H), 7.67–7.58 (m, 4H), 7.37 (d, *J* = 8.4 Hz, 2H), 2.24 (s, 6H). ^13^C NMR (100 MHz, DMSO-*d*_6_) *δ*: 155.0 (d, *J* = 2.6 Hz), 151.2 (d, *J* = 12.0 Hz), 145.4, 144.3, 141.2 (d, *J* = 247.4 Hz), 140.0 (d, *J* = 19.9 Hz), 137.1, 136.5, 136.3, 132.8, 132.4, 127.4, 126.6, 119.5, 118.8, 117.4, 109.9, 101.3, 18.2. ^19^F NMR (376 MHz, DMSO-*d*_6_) *δ*: −164.1. IR: 3354, 3332, 2915, 2227, 2213, 1616, 1606, 1521, 1423, 1223, 1170, 829, 772 cm^−1^. HRMS (ESI): Calcd. for C_26_H_20_FN_6_ [M + H]^+^, 435.1728, Found 435.1721. HPLC purity: 98.16%.

4-((5-Fluoro-4-((2-fluoro-4-(pyridin-4-yl)phenyl)amino)pyrimidin-2-yl)amino)benzonitrile (**A19**). Yield 29%, white solid, mp: 287‒289 °C. ^1^H NMR (400 MHz, DMSO-*d*_6_) *δ*: 9.78 (s, 1H), 9.56 (s, 1H), 8.70–8.61 (m, 2H), 8.22 (d, *J* = 3.2 Hz, 1H), 7.91 (d, *J* = 11.8 Hz, 1H), 7.85–7.63 (m, 6H), 7.57–7.49 (m, 2H). ^13^C NMR (100 MHz, DMSO-*d*_6_) *δ*: 156.8 (d, *J* = 247.5 Hz), 154.8 (d, *J* = 3.1 Hz), 150.5 (d, *J* = 11.7 Hz), 150.4, 145.3 (d, *J* = 1.7 Hz), 145.2, 141.4 (d, *J* = 248.3 Hz), 141.2 (d, *J* = 19.5 Hz), 136.0 (d, *J* = 7.5 Hz), 132.7, 128.2 (d, *J* = 2.1 Hz), 126.7 (d, *J* = 12.5 Hz), 122.8 (d, *J* = 3.1 Hz), 121.1, 119.6, 117.7, 114.4 (d, *J* = 21.4 Hz), 101.7. ^19^F NMR (376 MHz, DMSO-*d*_6_) *δ*: −118.4, −163.1. IR: 3430, 2216, 1624, 1605, 1530, 1509, 1483, 1415, 1318, 1177, 829, 804 cm^−1^. HRMS (ESI): Calcd. for C_22_H_15_F_2_N_6_ [M + H]^+^, 401.1321, Found 401.1322. HPLC purity: 95.59%.

4-((4-((2-Chloro-4-(pyridin-4-yl)phenyl)amino)-5-fluoropyrimidin-2-yl)amino)benzonitrile (**A20**). Yield 20%, white solid, mp: 236‒238 °C. ^1^H NMR (400 MHz, DMSO-*d*_6_) *δ*: 9.75 (s, 1H), 9.47 (s, 1H), 8.76–8.60 (m, 2H), 8.22 (d, *J* = 3.6 Hz, 1H), 8.10 (d, *J* = 2.9 Hz, 1H), 7.95–7.65 (m, 6H), 7.55–7.44 (m, 2H). ^13^C NMR (100 MHz, DMSO-*d*_6_) *δ*: 154.7 (d, *J* = 3.1 Hz), 150.7 (d, *J* = 11.7 Hz), 150.4, 145.1, 145.0, 141.2 (d, *J* = 248.2 Hz), 141.1 (d, *J* = 19.4 Hz), 136.3, 136.1, 132.6, 131.1, 129.3, 127.9, 126.0, 121.1, 119.6, 117.7, 101.6. ^19^F NMR (376 MHz, DMSO-*d*_6_) *δ*: −163.2. IR: 3401, 3249, 2926, 2222, 1614, 1594, 1525, 1501, 1477, 1414, 1234, 1176, 832, 800 cm^−1^. HRMS (ESI): Calcd. for C_22_H_15_ClFN_6_ [M + H]^+^, 417.1025, Found 417.1020. HPLC purity: 95.10%.

4-((5-Fluoro-4-((2-methyl-4-(pyridin-4-yl)phenyl)amino)pyrimidin-2-yl)amino)benzonitrile (**A21**). Yield 50%, yellow solid, mp: 236‒238 °C. ^1^H NMR (400 MHz, DMSO-*d*_6_) *δ*: 9.68 (s, 1H), 9.28 (s, 1H), 8.84–8.59 (m, 2H), 8.16 (d, *J* = 3.4 Hz, 1H), 7.83–7.66 (m, 6H), 7.56–7.33 (m, 3H), 2.31 (s, 3H). ^13^C NMR (100 MHz, DMSO-*d*_6_) *δ*: 154.8 (d, *J* = 3.2 Hz), 151.0 (d, *J* = 11.7 Hz), 149.6, 147.1, 145.3, 141.3 (d, *J* = 247.9 Hz), 140.4 (d, *J* = 19.6 Hz), 137.9, 135.2, 134.5, 132.5, 129.0, 127.9, 124.7, 121.1, 119.6, 117.6, 101.4, 18.1. ^19^F NMR (376 MHz, DMSO-*d*_6_) *δ*: −163.7. IR: 3456, 3266, 2222, 1605, 1521, 1467, 1412, 1175, 834, 806 cm^−1^. HRMS (ESI): Calcd. for C_23_H_18_FN_6_ [M + H]^+^, 397.1571, Found 397.1572. HPLC purity: 95.87%.

4-((5-Fluoro-4-((2-methoxy-4-(pyridin-4-yl)phenyl)amino)pyrimidin-2-yl)amino)benzonitrile (**A22**). Yield 64%, yellow solid, mp: 278‒279 °C. ^1^H NMR (400 MHz, DMSO-*d*_6_) *δ*: 9.79 (s, 1H), 8.75–8.59 (m, 3H), 8.19 (d, *J* = 2.2 Hz, 1H), 8.00–7.76 (m, 5H), 7.65–7.43 (m, 4H), 3.94 (s, 3H). ^13^C NMR (100 MHz, DMSO-*d*_6_) *δ*: 154.7 (d, *J* = 3.3 Hz), 152.4, 150.3 (d, *J* = 10.9 Hz), 150.2, 146.7, 145.2, 141.4 (d, *J* = 248.1 Hz), 140.4 (d, *J* = 19.5 Hz), 134.5, 132.7, 127.9, 125.0, 121.2, 119.7, 118.9, 117.7, 109.9, 101.7, 56.0. ^19^F NMR (376 MHz, DMSO-*d*_6_) *δ*: −163.5. IR: 3406, 2216, 1616, 1603, 1502, 1486, 1414, 1215, 1173, 1037, 833, 799 cm^−1^. HRMS (ESI): Calcd. for C_23_H_18_FN_6_O [M + H]^+^, 413.1521, Found 413.1523. HPLC purity: 96.28%.

4-((4-((2,6-Difluoro-4-(pyridin-4-yl)phenyl)amino)-5-fluoropyrimidin-2-yl)amino)benzonitrile (**A23**). Yield 21%, white solid, mp: 284‒286 °C. ^1^H NMR (400 MHz, DMSO-*d*_6_) *δ*: 9.78 (s, 1H), 9.62 (s, 1H), 8.73–8.66 (m, 2H), 8.24 (d, *J* = 3.3 Hz, 1H), 7.91–7.80 (m, 4H), 7.74–7.63 (m, 2H), 7.48–7.43 (m, 2H). ^13^C NMR (100 MHz, DMSO-*d*_6_) *δ*: 158.9 (dd, *J* = 248.4, 5.8 Hz), 154.9 (d, *J* = 3.1 Hz), 150.8 (d, *J* = 12.1 Hz), 150.5, 145.1, 144.2, 141.5 (d, *J* = 18.9 Hz), 141.3 (d, *J* = 247.9 Hz), 137.4 (t, *J* = 9.6 Hz), 132.6, 121.2, 119.6, 117.7, 115.6 (t, *J* = 17.0 Hz), 110.6 (d, *J* = 24.5 Hz), 101.8. ^19^F NMR (376 MHz, DMSO-*d*_6_) *δ*: −116.2, −163.7. IR: 3308, 2926, 2219, 1601, 1585, 1498, 1481, 1455, 1404, 1345, 1170, 1047, 822, 775 cm^−1^. HRMS (ESI): Calcd. for C_22_H_14_F_3_N_6_ [M + H]^+^, 419.1227, Found 419.1229. HPLC purity: 96.13%.

4-((4-((2,6-Dimethyl-4-(pyridin-4-yl)phenyl)amino)-5-fluoropyrimidin-2-yl)amino)benzonitrile (**A24**). Yield 22%, white solid, mp: 335‒337 °C. ^1^H NMR (400 MHz, DMSO-*d*_6_) *δ*: 9.62 (s, 1H), 9.20 (s, 1H), 8.72–8.61 (m, 2H), 8.13 (d, *J* = 3.5 Hz, 1H), 7.83–7.72 (m, 2H), 7.72–7.58 (m, 4H), 7.42–7.31 (m, 2H), 2.25 (s, 6H). ^13^C NMR (100 MHz, DMSO-*d*_6_) *δ*: 155.1 (d, *J* = 3.1 Hz), 151.2 (d, *J* = 12.2 Hz), 150.2, 146.7, 145.4, 141.2 (d, *J* = 247.4 Hz), 140.0 (d, *J* = 19.5 Hz), 137.1, 136.7, 135.5, 132.4, 126.4, 121.1, 119.6, 117.5, 101.3, 18.3. ^19^F NMR (376 MHz, DMSO-*d*_6_) *δ*: −164.1. IR: 3412, 2922, 2216, 1600, 1581, 1400, 1345, 1175, 847, 821 cm^−1^. HRMS (ESI): Calcd. for C_24_H_20_FN_6_ [M + H]^+^, 411.1728, Found 411.1728. HPLC purity: 95.02%.

4′-((5-Chloro-2-((4-cyanophenyl)amino)pyrimidin-4-yl)amino)-3′-fluoro-[1,1′-biphenyl]-4-carbonitrile (**B6**). Yield 26%, white solid, mp: 286‒288 °C. ^1^H NMR (400 MHz, DMSO-*d*_6_) *δ*: 9.88 (s, 1H), 9.14 (s, 1H), 8.25 (s, 1H), 8.05–7.95 (m, 4H), 7.90–7.84 (m, 1H), 7.75–7.61 (m, 4H), 7.51–7.43 (m, 2H). ^13^C NMR (100 MHz, DMSO-*d*_6_) *δ*: 157.2 (d, *J* = 247.4 Hz), 157.1, 156.8, 154.8, 144.8, 142.7 (d, *J* = 2.0 Hz), 137.5 (d, *J* = 7.6 Hz), 132.9, 132.6, 129.0 (d, *J* = 2.1 Hz), 127.5, 126.6 (d, *J* = 12.3 Hz), 123.1 (d, *J* = 3.0 Hz), 119.5, 118.8, 118.2, 114.5 (d, *J* = 21.7 Hz), 110.5, 105.3, 102.1. ^19^F NMR (376 MHz, DMSO-*d*_6_) *δ*: −118.4. IR: 3403, 2919, 2228, 1635, 1609, 1572, 1526, 1421, 1278, 1176, 822, 770 cm^−1^. HRMS (ESI): Calcd. for C_24_H_15_ClFN_6_ [M + H]^+^, 441.1025, Found 441.1022. HPLC purity: 95.16%.

3′-Chloro-4′-((5-chloro-2-((4-cyanophenyl)amino)pyrimidin-4-yl)amino)-[1,1′-biphenyl]-4-carbonitrile (**B7**). Yield 32%, white solid, mp: 302‒304 °C. ^1^H NMR (400 MHz, DMSO-*d*_6_) *δ*: 9.89 (s, 1H), 9.08 (s, 1H), 8.26 (s, 1H), 8.10–7.94 (m, 5H), 7.90–7.77 (m, 2H), 7.71–7.63 (m, 2H), 7.56–7.39 (m, 2H). ^13^C NMR (100 MHz, DMSO-*d*_6_) *δ*: 157.0, 156.7, 154.7, 144.7, 142.6, 137.4, 136.1, 133.0, 132.5, 131.1, 129.2, 128.0, 127.6, 126.3, 119.4, 118.7, 118.2, 110.6, 105.3, 102.1. IR: 3018, 2226, 1609, 1580, 1523, 1423, 1277, 1176, 822, 770 cm^−1^. HRMS (ESI): Calcd. for C_24_H_15_Cl_2_N_6_ [M + H]^+^, 457.0730, Found 457.0728. HPLC purity: 98.96%.

4′-((5-Chloro-2-((4-cyanophenyl)amino)pyrimidin-4-yl)amino)-3′-methyl-[1,1′-biphenyl]-4-carbonitrile (**B8**). Yield 25%, white solid, mp: 280‒282 °C. ^1^H NMR (400 MHz, DMSO-*d*_6_) *δ*: 9.80 (s, 1H), 8.97 (s, 1H), 8.20 (s, 1H), 8.02–7.92 (m, 4H), 7.79 (d, *J* = 2.2 Hz, 1H), 7.74–7.59 (m, 3H), 7.47 (d, *J* = 8.2 Hz, 1H), 7.44–7.35 (m, 2H), 2.26 (s, 3H). ^13^C NMR (100 MHz, DMSO-*d*_6_) *δ*: 157.2, 157.1, 154.3, 144.9, 144.1, 137.8, 136.2, 135.7, 132.9, 132.5, 129.1, 128.5, 127.4, 124.9, 119.5, 118.9, 118.0, 109.9, 105.1, 101.8, 18.0. IR: 3418, 3012, 2224, 1626, 1570, 1524, 1417, 1214, 1021, 828, 773 cm^−1^. HRMS (ESI): Calcd. for C_25_H_18_ClN_6_ [M + H]^+^, 437.1276, Found 437.1274. HPLC purity: 95.91%.

4′-((5-Chloro-2-((4-cyanophenyl)amino)pyrimidin-4-yl)amino)-3′-methoxy-[1,1′-biphenyl]-4-carbonitrile (**B9**). Yield 29%, white solid, mp: 327‒329 °C. ^1^H NMR (400 MHz, DMSO-*d*_6_) *δ*: 9.94 (s, 1H), 8.45 (s, 1H), 8.26 (s, 1H), 8.13–7.92 (m, 5H), 7.78 (d, *J* = 8.7 Hz, 2H), 7.65–7.48 (m, 3H), 7.48–7.39 (m, 1H), 3.96 (s, 3H). ^13^C NMR (100 MHz, DMSO-*d*_6_) *δ*: 157.1, 155.8, 154.2, 151.7, 144.8, 144.3, 135.3, 132.8, 132.8, 127.8, 127.5, 124.1, 119.5, 119.2, 119.0, 118.4, 110.0, 109.8, 105.8, 102.3, 56.2. IR: 3384, 3009, 2224, 1631, 1570, 1530, 1426, 1215, 825 cm^−1^. HRMS (ESI): Calcd. for C_25_H_18_ClN_6_O [M + H]^+^, 453.1225, Found 453.1217. HPLC purity: 95.04%.

4′-((5-Chloro-2-((4-cyanophenyl)amino)pyrimidin-4-yl)amino)-3′,5′-difluoro-[1,1′-biphenyl]-4-carbonitrile (**B10**). Yield 24%, white solid, mp: 260‒261 °C. ^1^H NMR (400 MHz, DMSO-*d*_6_) *δ*: 9.90 (s, 1H), 9.18 (s, 1H), 8.27 (s, 1H), 8.15–7.97 (m, 4H), 7.92–7.77 (m, 2H), 7.65–7.54 (m, 2H), 7.46–7.36 (m, 2H). ^13^C NMR (100 MHz, DMSO-*d*_6_) *δ*: 159.0 (dd, *J* = 248.1, 5.8 Hz), 157.2, 157.1, 155.1, 144.7, 141.7, 138.7 (t, *J* = 9.8 Hz), 133.0, 132.5, 127.8, 119.4, 118.6, 118.1, 115.7 (t, *J* = 16.9 Hz), 111.3, 110.8 (d, *J* = 24.5 Hz), 105.1, 102.2. ^19^F NMR (376 MHz, DMSO-*d*_6_) *δ*: −116.4. IR: 3267, 2221, 1606, 1573, 1510, 1427, 1220, 1174, 1039, 829, 777 cm^−1^. HRMS (ESI): Calcd. for C_24_H_14_ClF_2_N_6_ [M + H]^+^, 459.0931, Found 459.0933. HPLC purity: 96.57%.

4′-((5-Chloro-2-((4-cyanophenyl)amino)pyrimidin-4-yl)amino)-3′,5′-dimethyl-[1,1′-biphenyl]-4-carbonitrile (**B11**). Yield 27%, white solid, mp: 277‒279 °C. ^1^H NMR (400 MHz, DMSO-*d*_6_) *δ*: 9.76 (s, 1H), 8.90 (s, 1H), 8.18 (s, 1H), 8.15–7.84 (m, 4H), 7.64 (s, 2H), 7.57–7.50 (m, 2H), 7.33–7.25 (m, 2H), 2.22 (s, 6H). ^13^C NMR (100 MHz, DMSO-*d*_6_) *δ*: 157.3, 157.1, 154.2, 145.0, 144.3, 137.2, 137.0, 136.6, 132.9, 132.3, 127.4, 126.6, 119.4, 118.9, 117.9, 110.0, 104.7, 101.7, 18.2. IR: 3388, 2220, 1569, 1507, 1405, 1321, 1220, 1169, 1019, 831, 776 cm^−1^. HRMS (ESI): Calcd. for C_26_H_20_ClN_6_ [M + H]^+^, 451.1432, Found 451.1427. HPLC purity: 99.50%.

4-((5-Chloro-4-((2-fluoro-4-(pyridin-4-yl)phenyl)amino)pyrimidin-2-yl)amino)benzonitrile (**B17**). Yield 63%, white solid, mp: 318‒320 °C. ^1^H NMR (400 MHz, DMSO-*d*_6_) *δ*: 9.88 (s, 1H), 9.17 (s, 1H), 8.78–8.58 (m, 2H), 8.26 (s, 1H), 8.01–7.58 (m, 7H), 7.61–7.39 (m, 2H). ^13^C NMR (100 MHz, DMSO-*d*_6_) *δ*: 157.2 (d, *J* = 247.4 Hz), 157.1, 156.8, 154.8, 150.4, 145.2, 144.8, 136.4 (d, *J* = 7.6 Hz), 132.6, 129.1, 127.1 (d, *J* = 12.3 Hz), 122.8 (d, *J* = 3.0 Hz), 121.0, 119.4, 118.2, 114.3 (d, *J* = 21.7 Hz), 105.3, 102.1. ^19^F NMR (376 MHz, DMSO-*d*_6_) *δ*: −118.2. IR: 3410, 2918, 2850, 2219, 1600, 1566, 1481, 1412, 1175, 829, 803, 770 cm^−1^.HRMS (ESI): Calcd. for C_22_H_15_ClFN_6_ [M + H]^+^, 417.1025, Found 417.1027. HPLC purity: 95.65%.

4-((5-Chloro-4-((2-chloro-4-(pyridin-4-yl)phenyl)amino)pyrimidin-2-yl)amino)benzonitrile (**B18**). Yield 31%, pale yellow solid, mp: 271‒272 °C. ^1^H NMR (400 MHz, DMSO-*d*_6_) *δ*: 9.89 (s, 1H), 9.11 (s, 1H), 8.73–8.63 (m, 2H), 8.26 (s, 1H), 8.12 (d, *J* = 2.0 Hz, 1H), 7.92–7.78 (m, 4H), 7.64 (d, *J* = 8.6 Hz, 2H), 7.44 (d, *J* = 8.7 Hz, 2H). ^13^C NMR (100 MHz, DMSO-*d*_6_) *δ*: 157.0, 156.7, 154.8, 150.4, 145.1, 144.8, 136.6, 136.3, 132.5, 131.2, 129.3, 127.8, 126.1, 121.2, 119.5, 118.2, 105.3, 102.1. IR: 3363, 2919, 2850, 2223, 1600, 1562, 1507, 1406, 1174, 832, 804, 769 cm^−1^. HRMS (ESI): Calcd. for C_22_H_15_Cl_2_N_6_ [M + H]^+^, 433.0730, Found 433.0734. HPLC purity: 95.21%.

4-((5-Chloro-4-((2-methyl-4-(pyridin-4-yl)phenyl)amino)pyrimidin-2-yl)amino)benzonitrile (**B19**). Yield 57%, white solid, mp: 261 ‒ 263 °C. ^1^H NMR (400 MHz, DMSO-*d*_6_) *δ*: 9.80 (s, 1H), 8.98 (s, 1H), 8.76–8.59 (m, 2H), 8.20 (s, 1H), 7.88–7.70 (m, 4H), 7.68–7.59 (m, 2H), 7.49 (d, *J* = 8.2 Hz, 1H), 7.44–7.31 (m, 2H), 2.27 (s, 3H). ^13^C NMR (100 MHz, DMSO-*d*_6_) *δ*: 157.14, 157.11, 154.3, 150.3, 146.5, 144.9, 138.2, 135.8, 135.0, 132.4, 128.8, 128.6, 124.7, 121.0, 119.5, 118.0, 105.1, 101.8, 18.0. IR: 3422, 2920, 2850, 2219, 1604, 1568, 1493, 1406, 1174, 834, 806, 769 cm^−1^. HRMS (ESI): Calcd. for C_23_H_18_ClN_6_ [M + H]^+^, 413.1276, Found 413.1276. HPLC purity: 98.96%.

4-((5-Chloro-4-((2-methoxy-4-(pyridin-4-yl)phenyl)amino)pyrimidin-2-yl)amino)benzonitrile (**B20**). Yield 47%, pale yellow solid, mp: 298–230 °C. ^1^H NMR (400 MHz, DMSO-*d*_6_) *δ*: 9.95 (s, 1H), 8.70–8.63 (m, 2H), 8.48 (s, 1H), 8.27 (s, 1H), 8.09 (d, *J* = 8.2 Hz, 1H), 7.96–7.73 (m, 4H), 7.63–7.43 (m, 4H), 3.97 (s, 3H). ^13^C NMR (100 MHz, DMSO) *δ*: 157.1, 155.8, 154.2, 151.7, 150.2, 146.6, 144.8, 134.2, 132.7, 128.2, 124.1, 121.1, 119.5, 118.9, 118.3, 109.7, 105.8, 102.2, 56.2. IR: 3397, 2920, 2216, 1604, 1568, 1480, 1413, 1212, 1035, 801, 770 cm^−1^. HRMS (ESI): Calcd. for C_23_H_18_ClN_6_O [M + H]^+^, 429.1225, Found 429.1218. HPLC purity: 95.06%.

4-((5-Chloro-4-((2,6-difluoro-4-(pyridin-4-yl)phenyl)amino)pyrimidin-2-yl)amino)benzonitrile (**B21**). Yield 61%, white solid, mp: 277‒278 °C. ^1^H NMR (400 MHz, DMSO-*d*_6_) *δ*: 9.90 (s, 1H), 9.19 (s, 1H), 8.78–8.68 (m, 2H), 8.28 (s, 1H), 7.94–7.80 (m, 4H), 7.65–7.55 (m, 2H), 7.48–7.37 (m, 2H). ^13^C NMR (100 MHz, DMSO-*d*_6_) *δ*: 159.1 (dd, *J* = 248.5, 5.8 Hz), 157.2, 157.1, 155.1, 150.5, 144.7, 144.2, 137.7 (t, *J* = 9.6 Hz), 132.5, 121.2, 119.4, 118.1, 116.2 (t, *J* = 17.0 Hz), 110.6 (d, *J* = 24.7 Hz), 105.0, 102.2. ^19^F NMR (376 MHz, DMSO-*d*_6_) *δ*: −116.3. IR: 3163, 2927, 2220, 1571, 1526, 1408, 1223, 1042, 822, 781 cm^−1^. HRMS (ESI): Calcd. for C_22_H_14_ClF_2_N_6_ [M + H]^+^, 435.0931, Found 435.0927. HPLC purity: 98.64%.

4-((5-Chloro-4-((2,6-dimethyl-4-(pyridin-4-yl)phenyl)amino)pyrimidin-2-yl)amino)benzonitrile (**B22**). Yield 35%, pale yellow solid, mp: 300‒302 °C. ^1^H NMR (400 MHz, DMSO-*d*_6_) *δ*: 9.76 (s, 1H), 8.92 (s, 1H), 8.67 (d, *J* = 5.6 Hz, 2H), 8.18 (s, 1H), 7.82–7.65 (m, 4H), 7.62–7.47 (m, 2H), 7.39–7.23 (m, 2H), 2.22 (s, 6H). ^13^C NMR (100 MHz, DMSO-*d*_6_) *δ*: 157.3, 157.1, 154.2, 150.3, 146.7, 145.0, 137.4, 137.2, 135.5, 132.3, 126.3, 121.1, 119.4, 117.9, 104.7, 101.7, 18.2. IR: 3398, 2920, 2850, 2218, 1565, 1577, 1465, 1410, 1390, 1317, 1173, 842, 822, 777 cm^−1^. HRMS (ESI): Calcd. for C_24_H_20_ClN_6_ [M + H]^+^, 427.1432, Found 427.1428. HPLC purity: 95.03%.

### 4.2 Anti-HIV-1 assays

The anti-HIV-1 activity of **A1**–**A24** and **B1**–**B22** at the cellular or enzyme level were evaluated following our previously reported method^15^.

### 4.3 Molecular modelling studies

The specific process of molecular docking and MD simulation for **A12** were performed using previously established protocol^11^.

### 4.4 Human liver microsomal stability assay

A mixed system of human liver microsomes (0.5 mg/mL), MgCl_2_ (3 mmol/L), tested compound (1.0 μmol/L), and NADPH (1.0 mmol/L) in PBS (0.1 mol/L) was incubated at 37 °C. After 0, 5, 15, 30, 45, and 60 min, aliquots were sampled respectively and then terminated by adding methanol containing internal standard. After centrifugation using a Thermo Scientific Fresco 17 microcentrifuge (4 °C, 3800 rpm, 15 min), the supernatant was removed for LC‒MS/MS analysis.

### 4.5 CYP enzyme inhibition assay

An incubation system of human liver microsomes (20 μL, 1.5 mg/mL), 20 μL probe substrates, and 50 μL test compound in Tris (0.1 mol/L) was pre-incubated at 37 °C for 10 min, and then initiated by adding NADPH (10 μL, 10 mmol/L). The system was incubated for another 15 min and subsequently terminated by 100 μL MeCN containing internal standard (propranolol, nadolol). Finally, the sample was centrifugated to obtain the supernatant for LC‒MS/MS analysis.

### 4.6 hERG inhibition assay

The stock solution of **A12** (20 mmol/L, dissolving in DMSO) was thawed and serially diluted 3-fold with DMSO to obtain six working solutions with gradient concentrations and subsequently diluted 500-fold to the external solution to obtain the final solution (40.00, 13.33, 4.44, 1.48, 0.49, and 0.16 μmol/L). CHO-hERG cells were cultured under the condition of 37 °C and 5% CO_2_. The culture medium was removed when the cell density grew to 60%–80%. Then, the cells were washed (PBS, 7 mL) and digested. After completion, culture medium (7 mL) was added for neutralization and then centrifuged to obtain the cells, which were subsequently resuspended into 5 mL of culture medium for automated Qpatch assays. The electrophysiology process was recorded using the QPatch instrument. The cells were clamped at an equilibrium potential of −80 mV. Voltage stimulation was conducted every 15 s, which concludes a 5 s, +40 mV depolarizing pulse and a 5 s, −50 mV repolarizing pulse. After 2 min of recording, extracellular fluid was given and recorded for 5 min. Next, **A12** was tested sequentially from low to high concentration. After completing the administration of **A12**, cisapride (maximum concentration 3 μmol/L) was tested as a positive control. Each concentration was evaluated on 3 cells (*n* = 3).

### 4.7 Plasma protein binding assay

The DMSO solution of **A12** was diluted to 100 μmol/L with MeOH:H_2_O (1:1) and then added to the pre-loaded plasma to obtain plasma samples with a test concentration of 1 μmol/L. Subsequently, 100 μL of control dialysis buffer (phosphate buffer + 0.002% Tween-80) and 100 μL of **A12** plasma sample were transferred to the receiver and the donor side of dialysis chambers, respectively, two copies of each sample. The dialysis system was incubated in a shaker (37 °C, 6 h). Upon completion, 20 μL of samples were removed from each of the buffer and plasma chambers. After adding aliquots control plasma, PBS (0.002% Tween-80) respectively, 250 μL methanol solution with internal standard was added into each sample. The solution was mixed and centrifuged (3800 rpm, 10 min) to obtain the supernatant for LC‒MS/MS analysis.

### 4.8 PK study

All animal treatments were conducted using an approved protocol of Fudan University (2020CHEM-JS-009). **A12** was dissolved in a mixture of DMSO/Solutol HS 15/normal saline (10/10/80, *v*/*v*/*v*). Six Sprague–Dawley rats (male, 180−220 g) were assigned into two groups (*n* = 3) randomly to receive i.v. (1 mg/kg) or *p.o.* administration (10 mg/kg) of **A12**. At the time points of 5, 15, 30 min, 1, 2, 4, 6, 8, 10, 12, and 24 h, blood samples were collected and centrifuged (4 °C, 4500 rpm, 10 min) to obtain plasma. Before LC‒MS/MS analysis, the plasma needs to be stored at −80 °C. Subsequently, 120 μL of MeCN containing internal standard was added to 30 μL of plasma. The mixture was shaken and centrifuged (4 °C, 10,000 rpm, 15 min) to afford the supernatant layer for LC‒MS/MS analysis. Finally, The WinNolin software was applied to calculate the pharmacokinetic parameters.

### 4.9 Acute toxicity assay

**A12** was suspended in PEG400/normal saline (80/20, *v*/*v*) to achieve a concentration of 100 mg/ml. Sixteen ICR mice (eight male/eight female, 18–22 g) were assigned into 4 groups randomly, and each group contained 4 male mice or 4 female mice (*n* = 4). After fasting for 12 h, the experimental groups were administered **A12** (2 g/kg) by gavage, and the controls were dosed with vehicle solution. Over the next two weeks, the mortality, body weight, and abnormal behaviors of these mice were monitored daily. All mice were dissected after blood collection on Day 14, and several vital organs were removed and then assessed for pathological damage by HE staining. The collected blood was submitted for blood biochemistry testing.

## Author contributions

Xiao-Mei Chen conducted the synthesis and structure confirmation. Erik De Clercq and Christophe Pannecouque completed the biological evaluation. Shuai Wang, Xiao-Mei Chen and Qing–Qing Hao conducted druggability evaluation experiments and molecular docking studies. Shuai Wang, Xiao-Mei Chen, and Fen-Er Chen were in charge of writing this article and summarizing the data. Fen-Er Chen and Shuai Wang conceived the project and provided resources, supervision, and funding assistance.

All authors critically evaluated the manuscript prior to submission.

## Conflicts of interest

The authors declared no conflicts of interest.

## Appendix A Supporting information

The following is the Supporting Information to this article:

## References

[1] Reynolds C., de Koning C.B., Pelly S.C., van Otterlo W.A., Bode M.L. (2012). In search of a treatment for HIV-current therapies and the role of non-nucleoside reverse transcriptase inhibitors (NNRTIs). Chem Soc Rev.

[2] Campiani G., Ramunno A., Maga G., Nacci V., Fattorusso C., Catalanotti B. (2002). Non-nucleoside HIV-1 reverse transcriptase (RT) inhibitors: past, present, and future perspectives. Curr Pharm Des.

[3] Zhao L.M., Pannecouque C., De Clercq E., Wang S., Chen F.E. (2023). Structure-directed expansion of biphenyl-pyridone derivatives as potent non-nucleoside reverse transcriptase inhibitors with significantly improved potency and safety. Chin Chem Lett.

[4] Wang Y., De Clercq E., Li G. (2019). Current and emerging non-nucleoside reverse transcriptase inhibitors (NNRTIs) for HIV-1 treatment. Expert Opin Drug Metab Toxicol.

[5] Li G., Wang Y., De Clercq E. (2022). Approved HIV reverse transcriptase inhibitors in the past decade. Acta Pharm Sin B.

[6] Hao Q., Ling X., Pannecouque C., De Clercq E., Chen F. (2023). Linker optimization of HEPT derivatives as potent non-nucleoside HIV-1 reverse transcriptase inhibitors: from S=O to CHOR. Chin Chem Lett.

[7] Wang Z., Sharma P.P., Rathi B., Xie M., De Clercq E., Pannecouque C. (2023). Escaping from flatland: multiparameter optimization leads to the discovery of novel tetrahydropyrido[4,3-*d*]pyrimidine derivatives as human immunodeficiency virus-1 non-nucleoside reverse transcriptase inhibitors with superior antiviral activities against non-nucleoside reverse transcriptase inhibitor-resistant variants and favorable drug-like profiles. J Med Chem.

[8] Huang B., Ginex T., Luque F.J., Jiang X., Gao P., Zhang J. (2021). Structure-based design and discovery of pyridyl-bearing fused bicyclic HIV-1 inhibitors: synthesis, biological characterization, and molecular modeling studies. J Med Chem.

[9] Gu S.X., Lu H.H., Liu G.Y., Ju X.L., Zhu Y.Y. (2018). Advances in diarylpyrimidines and related analogues as HIV-1 nonnucleoside reverse transcriptase inhibitors. Eur J Med Chem.

[10] Ding L., Zhuang C.L., Chen F. (2021). Druggability modification strategies of the diarylpyrimidine-type non-nucleoside reverse transcriptase inhibitors. Med Res Rev.

[11] Huang W.J., Pannecouque C., De Clercq E., Wang S., Chen F.E. (2024). Fragment addition-based design of heteroaromatic-biphenyl-DAPYs as potent and orally available non-nucleoside reverse transcriptase Inhibitors featuring significantly enhanced safety. J Med Chem.

[12] Wang Z., Zalloum W.A., Wang W., Jiang X., De Clercq E., Pannecouque C. (2021). Discovery of novel dihydrothiopyrano[4,3-*d*]pyrimidine derivatives as potent HIV-1 NNRTIs with significantly reduced hERG inhibitory activity and improved resistance profiles. J Med Chem.

[13] Kang D., Feng D., Sun Y., Fang Z., Wei F., De Clercq E. (2020). Structure-based bioisosterism yields HIV-1 NNRTIs with improved drug-resistance profiles and favorable pharmacokinetic properties. J Med Chem.

[14] Kang D.W., Huo Z.P., Wu G.C., Xu J.B., Zhan P., Liu X.Y. (2017). Novel fused pyrimidine and isoquinoline derivatives as potent HIV-1 NNRTIs: a patent evaluation of WO2016105532A1, WO2016105534A1 and WO2016105564A1. Expert Opin Ther Pat.

[15] Ding L., Pannecouque C., De Clercq E., Zhuang C.L., Chen F.E. (2021). Improving druggability of novel diarylpyrimidine NNRTIs by a fragment-based replacement strategy: from biphenyl-DAPYs to heteroaromatic-biphenyl-DAPYs. J Med Chem.

[16] Zhuang C., Pannecouque C., De Clercq E., Chen F. (2020). Development of non-nucleoside reverse transcriptase inhibitors (NNRTIs): our past twenty years. Acta Pharm Sin B.

[17] Liang Y.H., He Q.Q., Zeng Z.S., Liu Z.Q., Feng X.Q., Chen F.E. (2010). Synthesis and anti-HIV activity of 2-naphthyl substituted DAPY analogues as non-nucleoside reverse transcriptase inhibitors. Bioorg Med Chem.

[18] Mao T.Q., He Q.Q., Wan Z.Y., Chen W.X., Chen F.E., Tang G.F. (2015). Anti-HIV diarylpyrimidine–quinolone hybrids and their mode of action. Bioorg Med Chem.

[19] Jin K., Yin H., De Clercq E., Pannecouque C., Meng G., Chen F. (2018). Discovery of biphenyl-substituted diarylpyrimidines as non-nucleoside reverse transcriptase inhibitors with high potency against wild-type and mutant HIV-1. Eur J Med Chem.

[20] Sang Y., Han S., Pannecouque C., De Clercq E., Zhuang C., Chen F. (2019). Ligand-based design of nondimethylphenyl-diarylpyrimidines with improved metabolic stability, safety, and oral pharmacokinetic profiles. J Med Chem.

[21] Ding L., Pannecouque C., De Clercq E., Zhuang C., Chen F.E. (2021). Hydrophobic pocket occupation design of difluoro-biphenyl-diarylpyrimidines as non-nucleoside HIV-1 reverse transcriptase inhibitors: from *N*-alkylation to methyl hopping on the pyrimidine ring. J Med Chem.

[22] Hernandes M.Z., Cavalcanti S.M.T., Moreira D.R.M., de Azevedo W.F., Leite A.C.L. (2010). Halogen atoms in the modern medicinal chemistry: hints for the drug design. Curr Drug Targets.

[23] Benedetto Tiz D., Bagnoli L., Rosati O., Marini F., Sancineto L., Santi C. (2022). New halogen-containing drugs approved by FDA in 2021: an overview on their syntheses and pharmaceutical use. Molecules.

[24] Shinada N.K., de Brevern A.G., Schmidtke P. (2019). Halogens in protein-ligand binding mechanism: a structural perspective. J Med Chem.

[25] Huo T.Y., Zhao X.Y., Cheng Z.R., Wei J.L., Zhu M.H., Dou X.D. (2024). Late-stage modification of bioactive compounds: improving druggability through efficient molecular editing. Acta Pharm Sin B.

[26] Shah P., Westwell A.D. (2007). The role of fluorine in medicinal chemistry. J Enzym Inhib Med Chem.

[27] Gillis E.P., Eastman K.J., Hill M.D., Donnelly D.J., Meanwell N.A. (2015). Applications of fluorine in medicinal chemistry. J Med Chem.

[28] Han S., Lu Y. (2023). Fluorine in anti-HIV drugs approved by FDA from 1981 to 2023. Eur J Med Chem.

[29] Park B.K., Kitteringham N.R., O′Neill P.M. (2001). Metabolism of fluorine-containing drugs. Annu Rev Pharmacol Toxicol.

[30] Purser S., Moore P.R., Swallow S., Gouverneur V. (2008). Fluorine in medicinal chemistry. Chem Soc Rev.

[31] Meanwell N.A. (2018). Fluorine and fluorinated motifs in the design and application of bioisosteres for drug design. J Med Chem.

[32] Chiodi D., Ishihara Y. (2023). "Magic Chloro": profound effects of the chlorine atom in drug discovery. J Med Chem.

[33] Deng Q., Zheng Q., Zuo B., Tu T. (2020). Robust NHC-palladacycles-catalyzed Suzuki−Miyaura cross-coupling of amides *via* C–N activation. Green Synth Catal.

[34] Gong B., Zhu H., Liu Y., Li Q., Yang L., Wu G. (2022). Palladium-catalyzed sulfonylative coupling of benzyl(allyl) carbonates with arylsulfonyl hydrazides. Green Synth Catal.

[35] Mordant C., Schmitt B., Pasquier E., Demestre C., Queguiner L., Masungi C. (2007). Synthesis of novel diarylpyrimidine analogues of TMC278 and their antiviral activity against HIV-1 wild-type and mutant strains. Eur J Med Chem.

[36] Zhao L.M., Pannecouque C., De Clercq E., Wang S., Chen F.E. (2023). Structure-based design of novel heterocycle-substituted ATDP analogs as non-nucleoside reverse transcriptase inhibitors with improved selectivity and solubility. Acta Pharm Sin B.

[37] Jiang X., Huang B., Rumrill S., Pople D., Zalloum W.A., Kang D. (2023). Discovery of diarylpyrimidine derivatives bearing piperazine sulfonyl as potent HIV-1 nonnucleoside reverse transcriptase inhibitors. Commun Chem.

[38] Kalyaanamoorthy S., Barakat K.H. (2018). Development of safe drugs: the hERG challenge. Med Res Rev.

[39] Garrido A., Lepailleur A., Mignani S.M., Dallemagne P., Rochais C. (2020). hERG toxicity assessment: useful guidelines for drug design. Eur J Med Chem.

[40] Janssen P.A., Lewi P.J., Arnold E., Daeyaert F., de Jonge M., Heeres J. (2005). In search of a novel anti-HIV drug: multidisciplinary coordination in the discovery of 4-[[4-[[4-[(1*E*)-2-cyanoethenyl]-2,6-dimethylphenyl]amino]-2-pyrimidinyl]amino]benzonitrile (R278474, rilpivirine). J Med Chem.

[41] Zainuddin R., Zaheer Z., Sangshetti J.N., Momin M. (2017). Enhancement of oral bioavailability of anti-HIV drug rilpivirine HCl through nanosponge formulation. Drug Dev Ind Pharm.

[42] Du F., Sun W., Morisseau C., Hammock B.D., Bao X., Liu Q. (2021). Discovery of memantyl urea derivatives as potent soluble epoxide hydrolase inhibitors against lipopolysaccharide-induced sepsis. Eur J Med Chem.

