# Supplementary material for: Bioisosterism-driven design of orally active, safe, and broad-spectrum biphenyl-DAPY derivatives as highly potent HIV-1 non-nucleoside reverse transcriptase inhibitors
Source: Acta Pharm Sin B. 2025 Jun 24;15(8):4115–36. doi: 10.1016/j.apsb.2025.06.016 (PMC12399242; doi:10.1016/j.apsb.2025.06.016)
Supplement: Multimedia component 1 [file mmc1.pdf]

Supporting Information for

ORIGINAL ARTICLE

Bioisosterism-driven design of orally active, safe, and broad-spectrum biphenyl-DAPY derivatives as highly potent HIV-1 non-nucleoside reverse transcriptase inhibitors

Xiao-Mei Chen[a], Qing-Qing Hao[a], Christophe Pannecouque[e], Erik De Clercq[e], Shuai Wang[c,d,*], Fen-Er Chen[a,b,c,d,*]

[a]*Sichuan Research Center for Drug Precision Industrial Technology, West China School of Pharmacy, Sichuan University, Chengdu 610041, China*

[b]*Institute of Flow Chemistry and Engineering, School of Chemistry and Materials, Jiangxi Normal University, Nanchang 330022, China*

[c]*Engineering Center of Catalysis and Synthesis for Chiral Molecules, Department of Chemistry, Fudan University, Shanghai 200433, China*

[d]*Shanghai Engineering Center of Industrial Asymmetric Catalysis for Chiral Drugs, Shanghai 200433, China*

[e]*Rega Institute for Medical Research, KU Leuven, Herestraat 49, Leuven B-3000, Belgium*

Received 16 January 2025; received in revised form 28 March 2025; accepted 25 April 2025

*Corresponding authors.

E-mail addresses: rfchen@fudan.edu.cn (Fen-Er Chen), shuaiwang@fudan.edu.cn (Shuai Wang).

Running title: Discovery of highly potent and orally active NNRTIs

Table of contents

1. ^{1}H NMR, ^{13}C NMR, ^{19}F NMR, HRMS spectra of **9a–9v**, **10a–10x** 3

2. ^{1}H NMR, ^{13}C NMR, ^{19}F NMR, HRMS, HPLC spectra of **A1−A24**, **B1−B22** 87

1. ^{1}H NMR, ^{13}C NMR, ^{19}F NMR, HRMS spectra of 9a–9v, 10a–10x

^{1}H NMR, ^{13}C NMR, ^{13}F NMR and HRMS spectra of 9a

^{1}H NMR

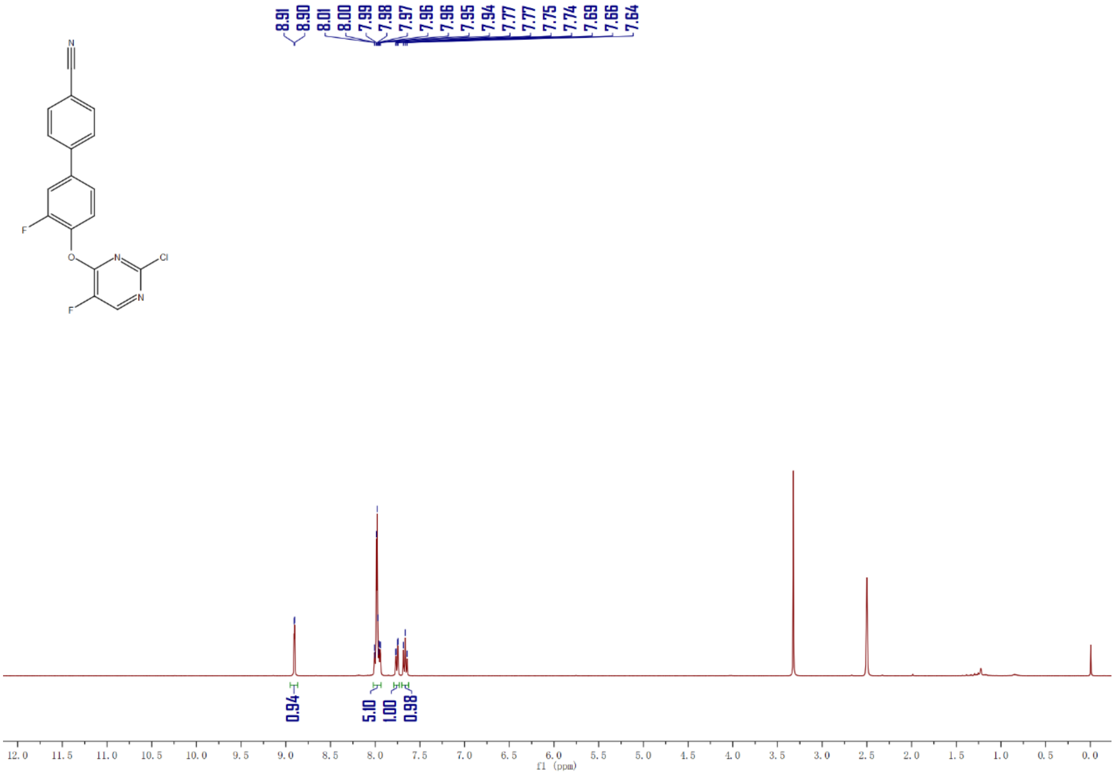

^{13}C NMR

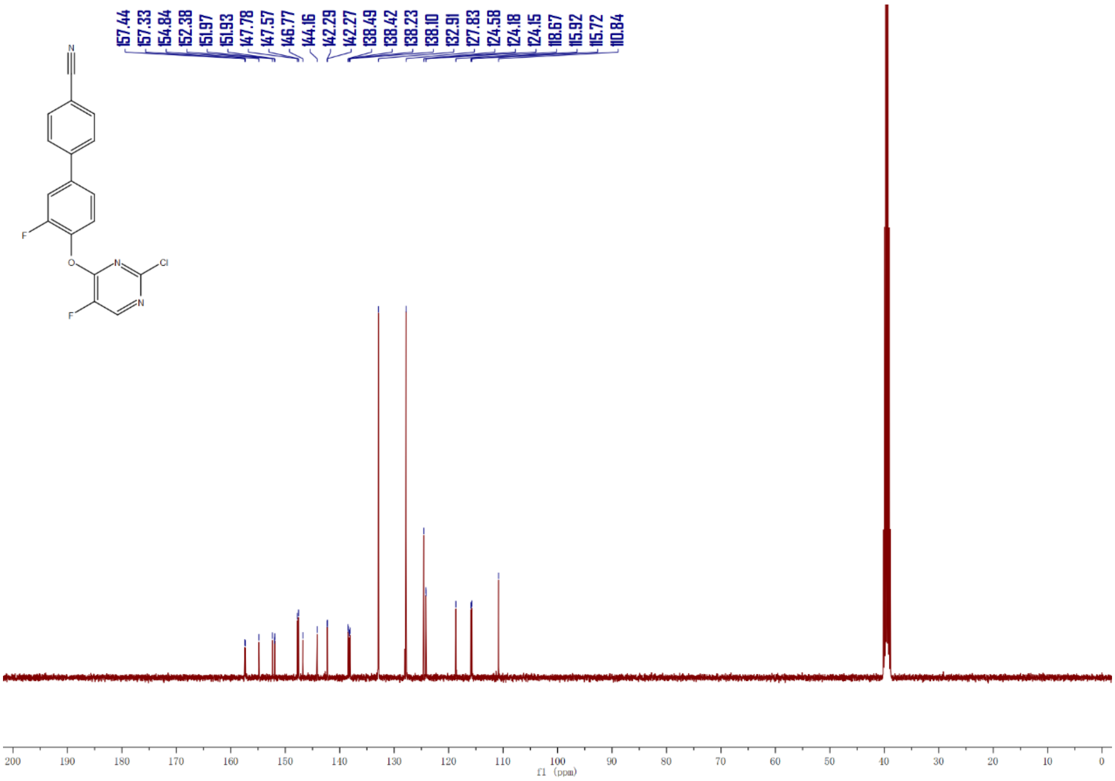

^{13}F NMR

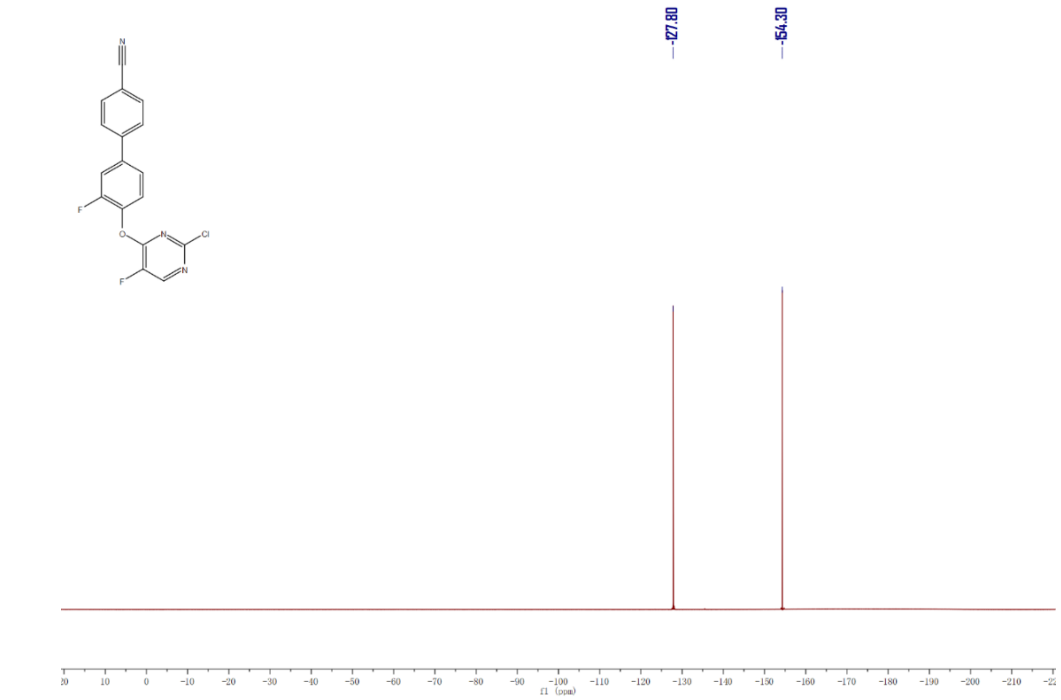

HRMS

Display Report

Analysis Info

Analysis Name	D:\CXM\CXM-20230518\20230518-CXM-002_RA2_01_9658.d	Acquisition Date	5/18/2023 5:02:29 PM
Method	MS-2MIN-POS.m	Operator	BDAL@DE
Sample Name	20230518-CXM-002	Instrument	compact 8255754.20127
Comment			

Acquisition Parameter

Source Type	ESI	Ion Polarity	Positive	Set Nebulizer	2.0 Bar
Focus	Active	Set Capillary	4500 V	Set Dry Heater	200 °C
Scan Begin	50 m/z	Set End Plate Offset	-500 V	Set Dry Gas	8.0 l/min
Scan End	3000 m/z	Set Charging Voltage	2000 V	Set Divert Valve	Waste
		Set Corona	0 nA	Set APCI Heater	0 °C

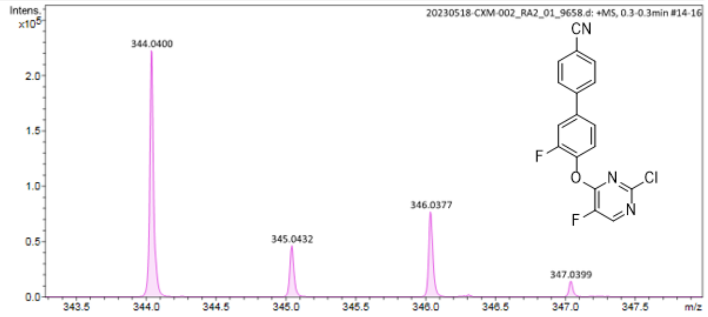

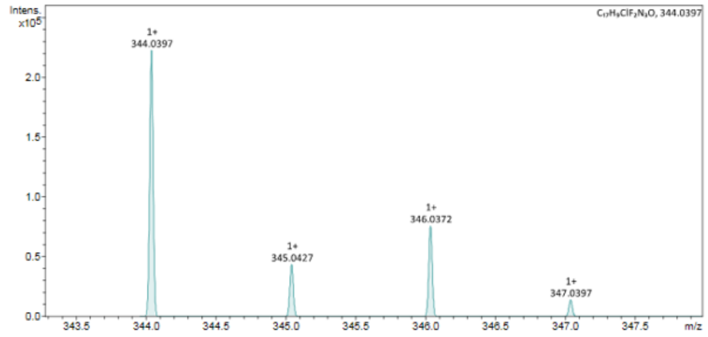

20230518-CXM-002_RA2_01_9658.d

Bruker Compass DataAnalysis 4.2 printed: 5/19/2023 9:01:03 PM by: BDAL@DE Page 1 of 1

^{1}H NMR, ^{13}C NMR, ^{13}F NMR and HRMS spectra of 9b

^{1}H NMR

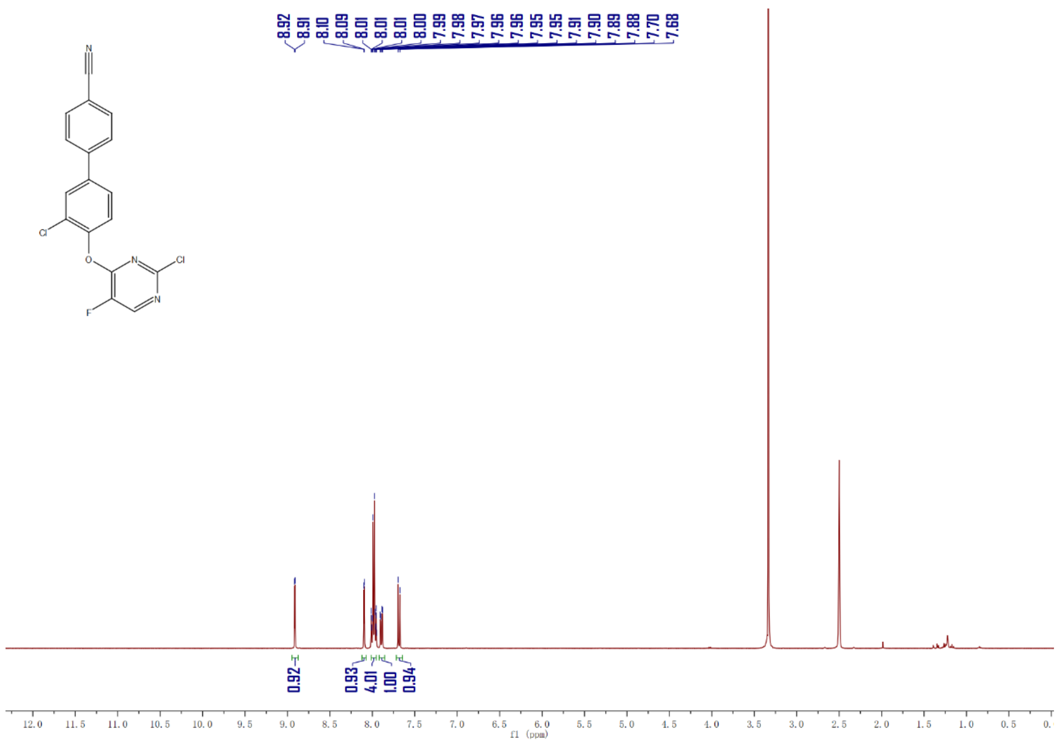

^{13}C NMR

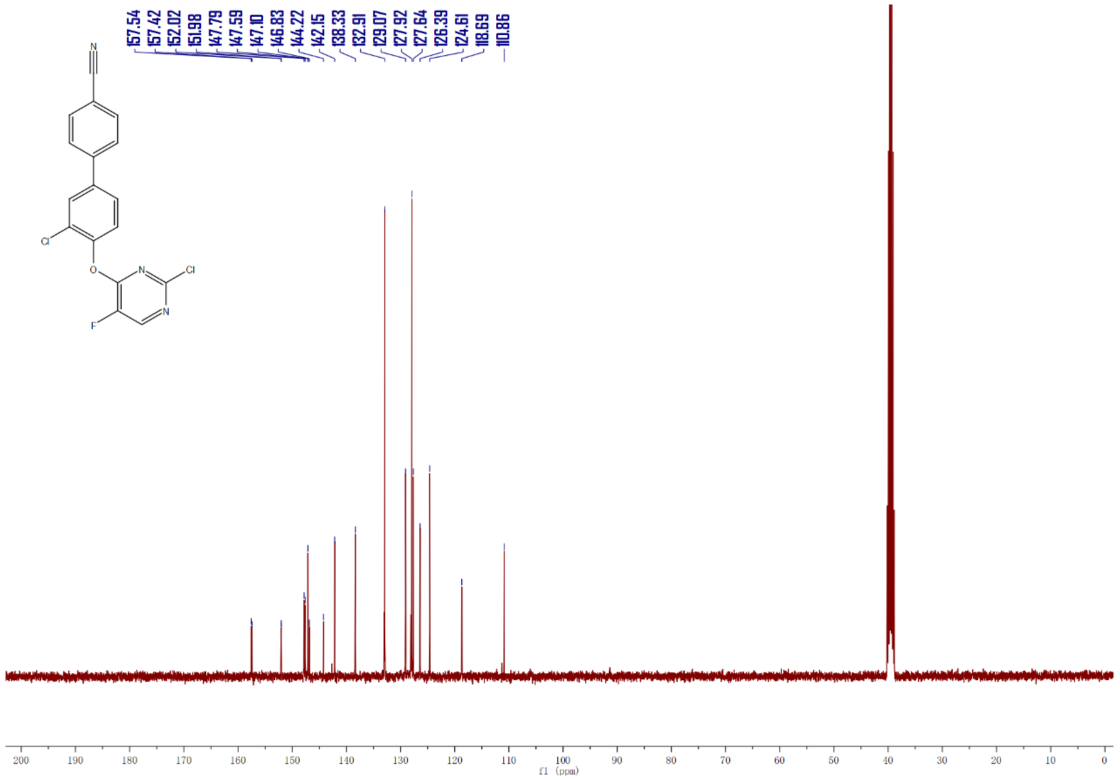

^{13}F NMR

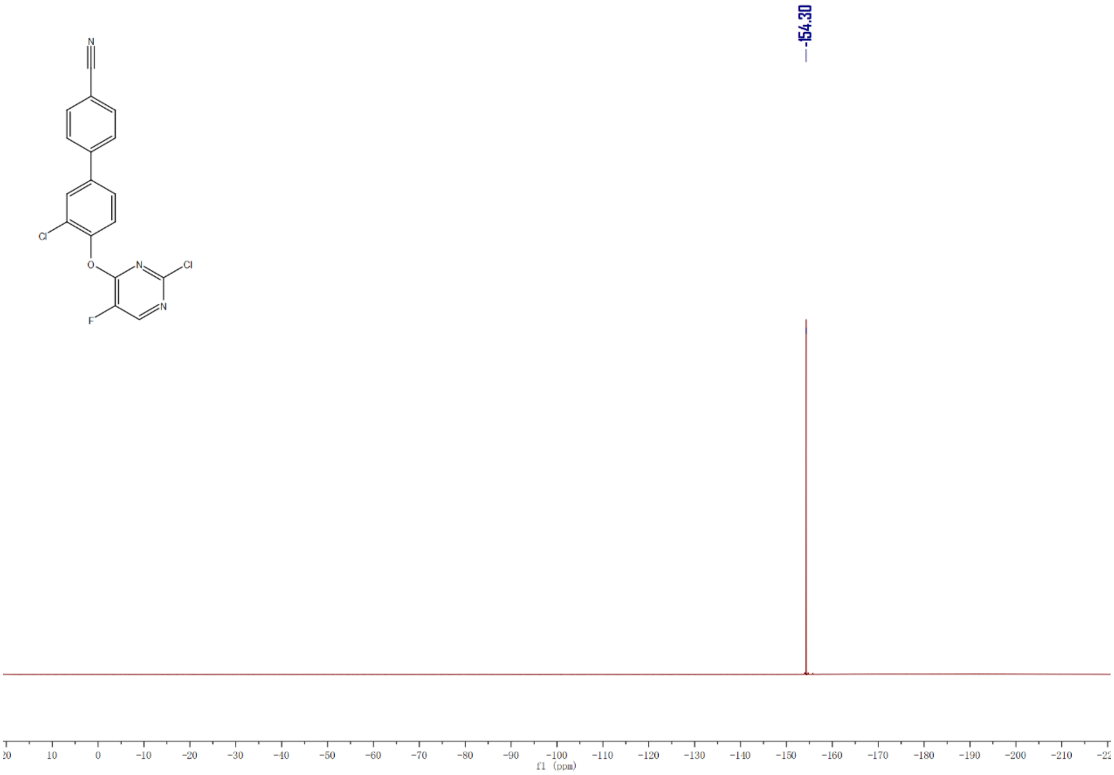
-154.30
f1 (ppm)

HRMS

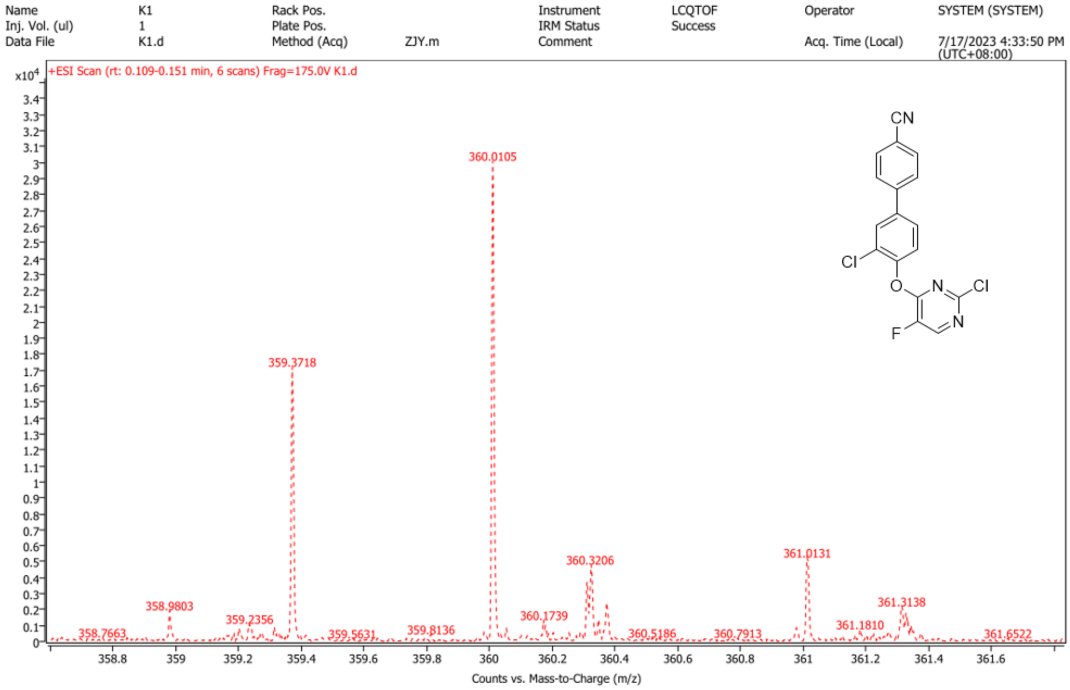
Name K1
Inj. Vol. (ul) 1
Data File K1.d
Rack Pos.
Plate Pos.
Method (Acq) ZJY.m
Instrument LCQTOF
IRM Status Success
Comment
Operator SYSTEM (SYSTEM)
Acq. Time (Local) 7/17/2023 4:33:50 PM (UTC+08:00)
+ESI Scan (rt: 0.109-0.151 min, 6 scans) Frag=175.0V K1.d
360.0105
359.3718
360.3206
361.0131
358.9803
359.2356
360.1739
361.3138
361.1810
Counts vs. Mass-to-Charge (m/z)

^{1}H NMR, ^{13}C NMR, ^{13}F NMR and HRMS spectra of 9c

^{1}H NMR

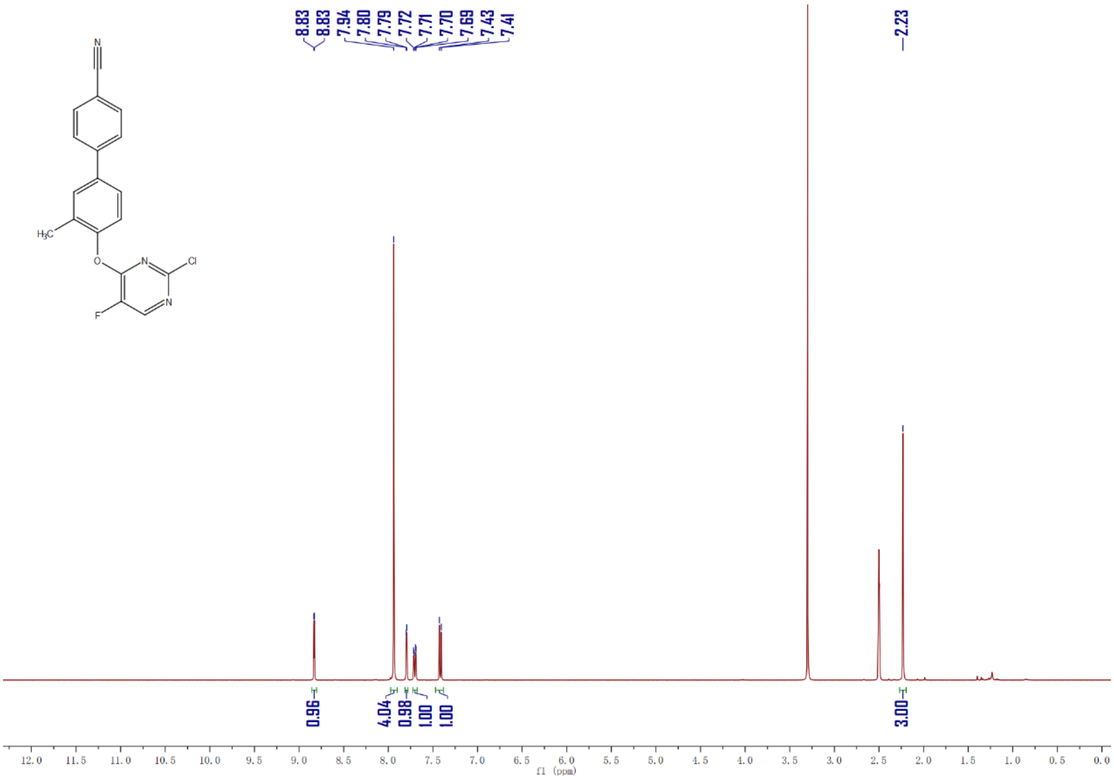

^{13}C NMR

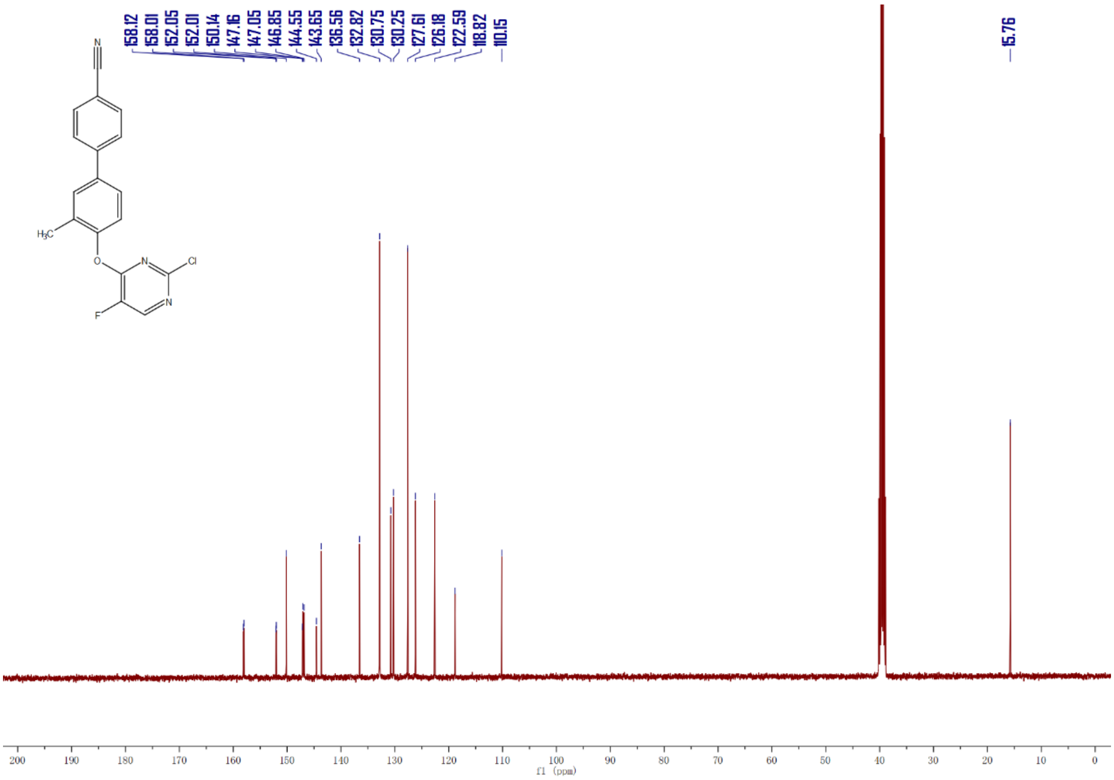

13F NMR

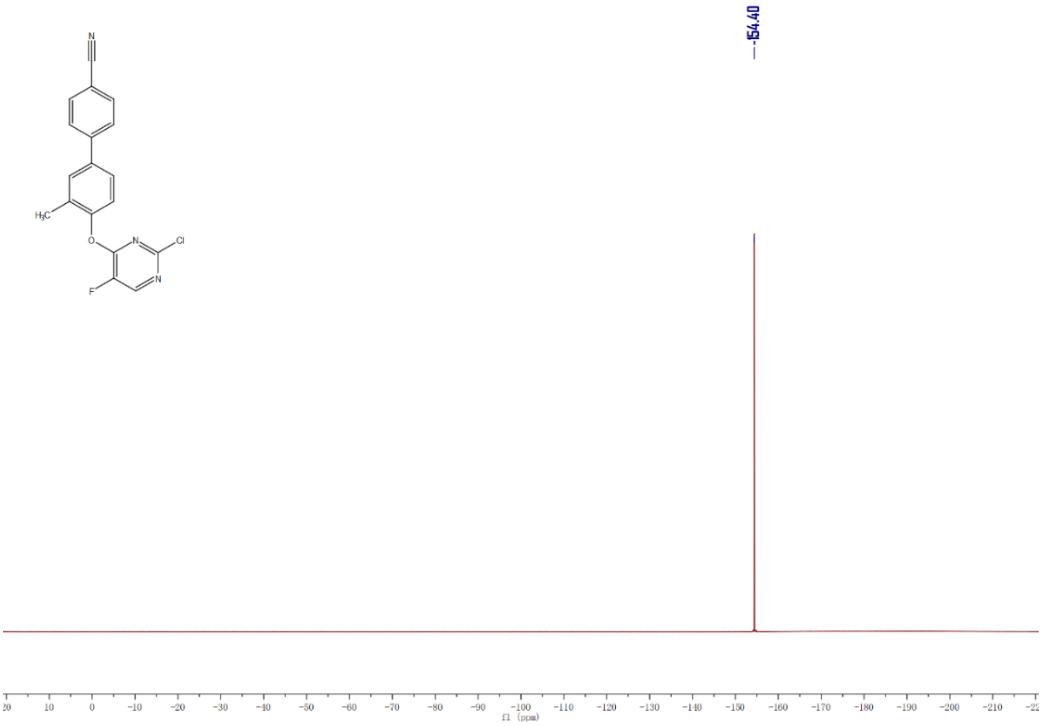

-154.40
H3C
O
N
Cl
F
f1 (ppm)

HRMS

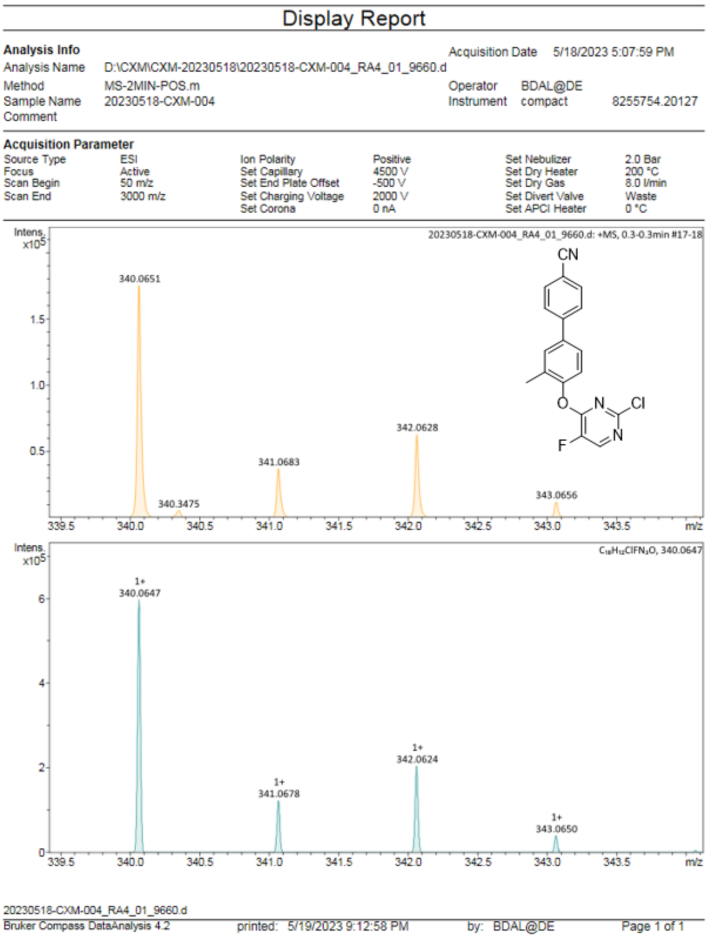

Display Report
Analysis Info
Analysis Name D:\CXM\CXM-20230518\20230518-CXM-004_RA4_01_9660.d
Method MS-2MIN-POS.m
Sample Name 20230518-CXM-004
Comment
Acquisition Date 5/18/2023 5:07:59 PM
Operator BDAL@DE
Instrument compact 8255754.20127
Acquisition Parameter
Source Type ESI
Focus Active
Scan Begin 50 m/z
Scan End 3000 m/z
Ion Polarity Positive
Set Capillary 4500 V
Set End Plate Offset -500 V
Set Charging Voltage 2000 V
Set Corona 0 nA
Set Nebulizer 2.0 Bar
Set Dry Heater 200 °C
Set Dry Gas 8.0 l/min
Set Divert Valve Waste
Set APCI Heater 0 °C
Intens. x10^5
20230518-CXM-004_RA4_01_9660.d: +MS, 0.3-0.3min #17-18
340.0651
340.3475
341.0683
342.0628
343.0656
CN
O
N
Cl
F
m/z
C18H12ClFN3O, 340.0647
340.0647
341.0678
342.0624
343.0650
20230518-CXM-004_RA4_01_9660.d
Bruker Compass DataAnalysis 4.2
printed: 5/19/2023 9:12:58 PM
by: BDAL@DE
Page 1 of 1

^{1}H NMR, ^{13}C NMR, [13]F NMR and HRMS spectra of 9d

^{1}H NMR

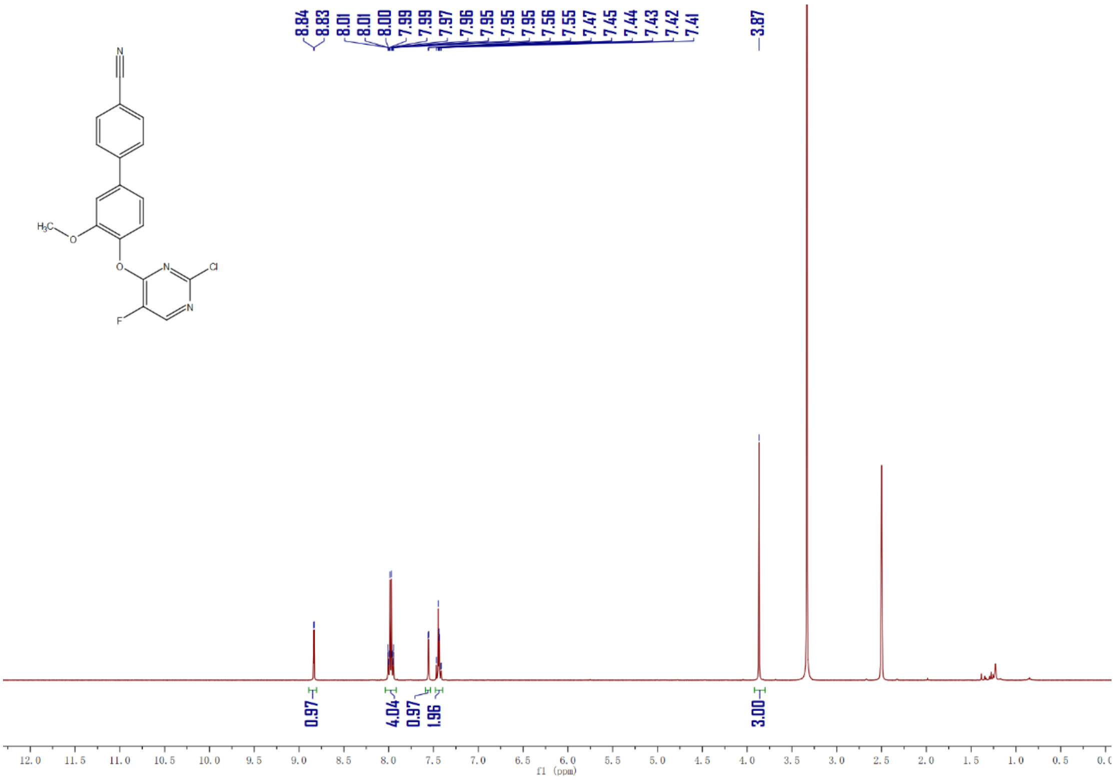

^{13}C NMR

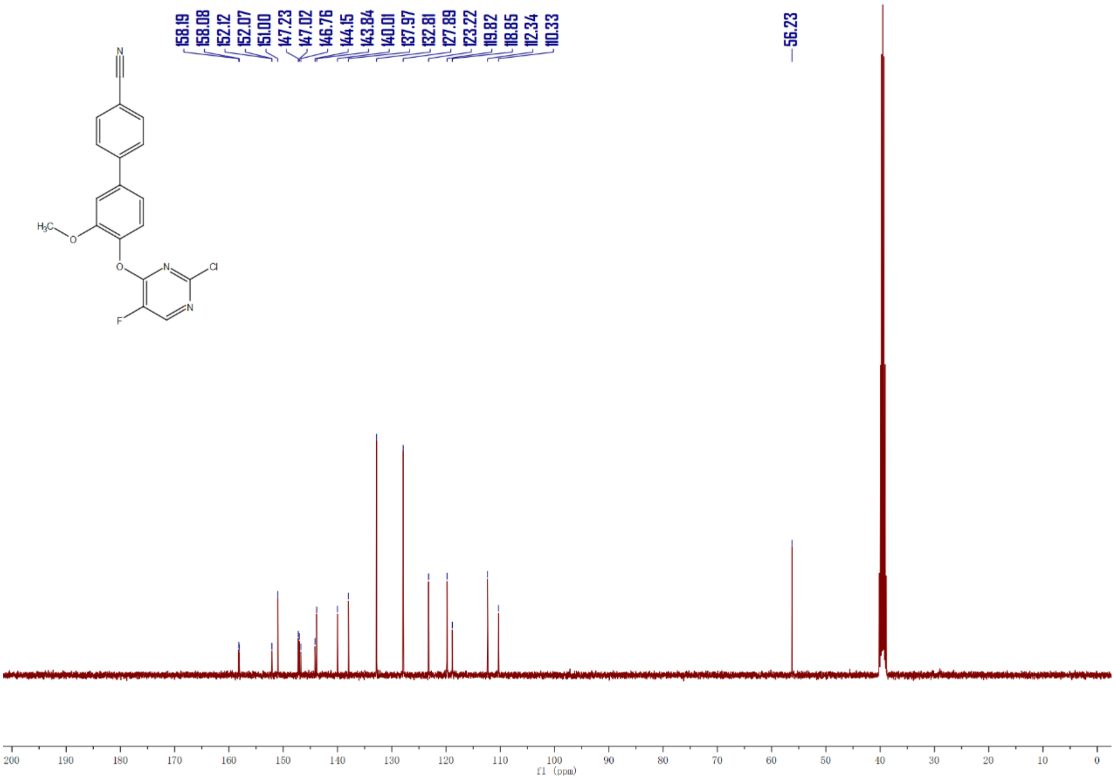

^{13}F NMR

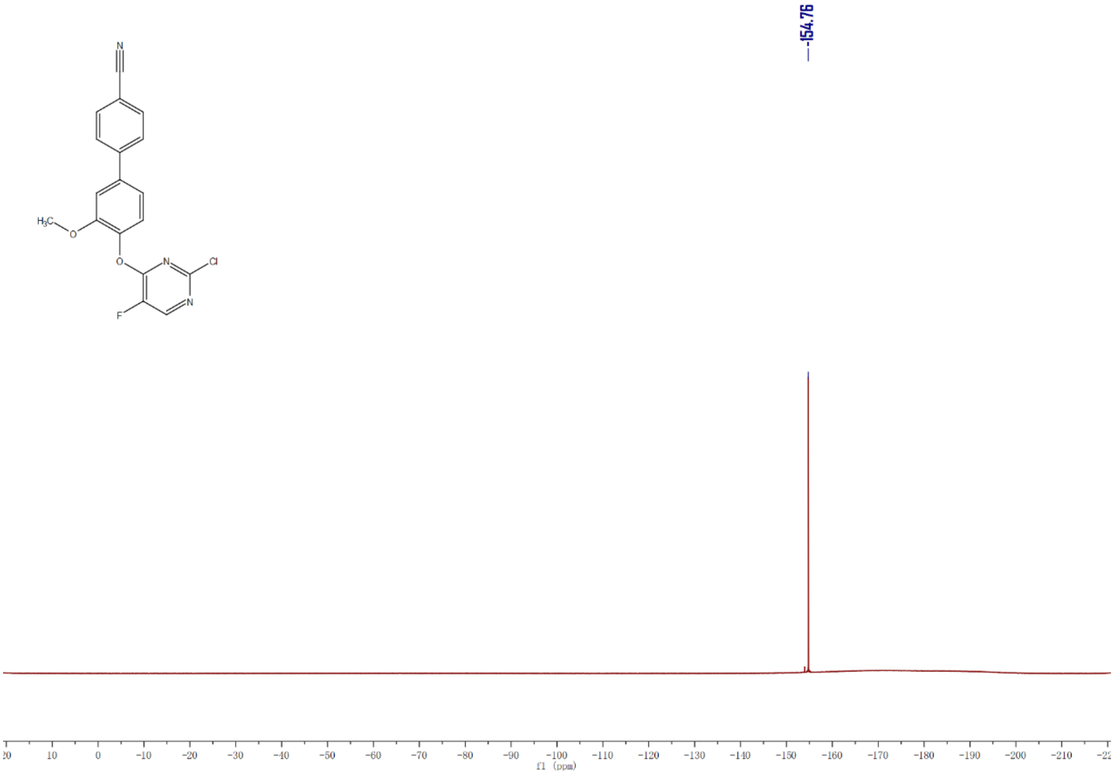

-154.76
f1 (ppm)

HRMS

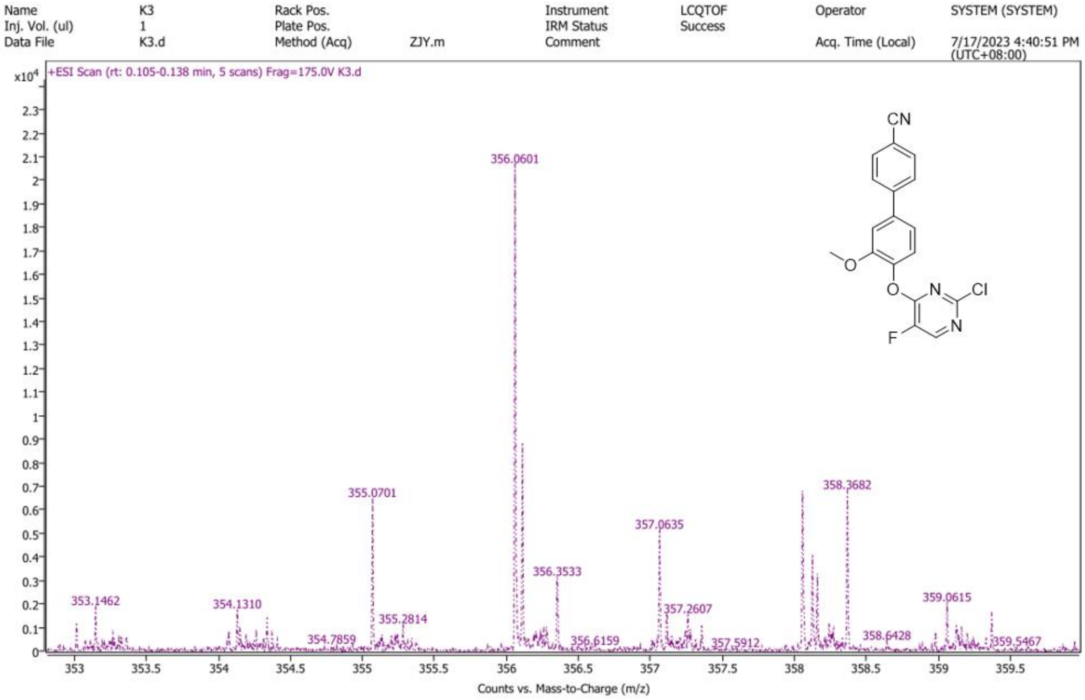

Name K3
Inj. Vol. (ul) 1
Data File K3.d
Rack Pos.
Plate Pos.
Method (Acq) ZJY.m
Instrument LCQTOF
IRM Status Success
Comment
Operator SYSTEM (SYSTEM)
Acq. Time (Local) 7/17/2023 4:40:51 PM (UTC+08:00)
+ESI Scan (rt: 0.105-0.138 min, 5 scans) Frag=175.0V K3.d
356.0601
355.0701
358.3682
357.0635
356.3533
353.1462
354.1310
355.2814
359.0615
357.2607
Counts vs. Mass-to-Charge (m/z)

^{1}H NMR, ^{13}C NMR, ^{13}F NMR and HRMS spectra of 9e

^{1}H NMR

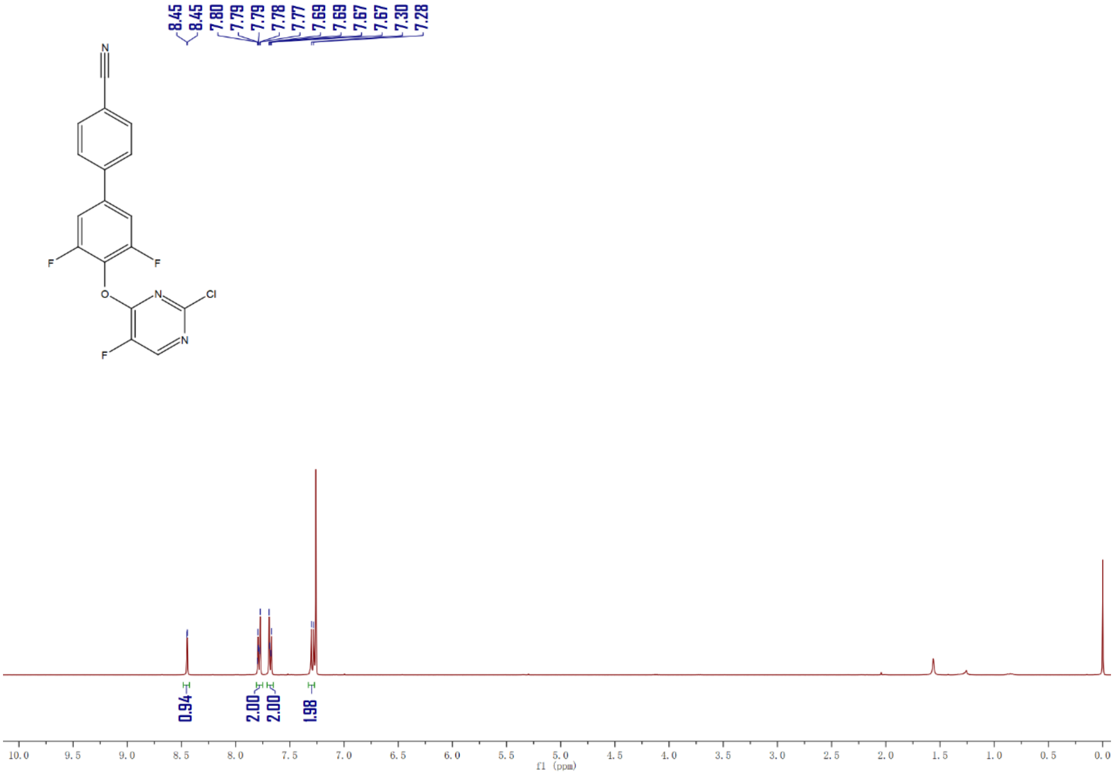

^{13}C NMR

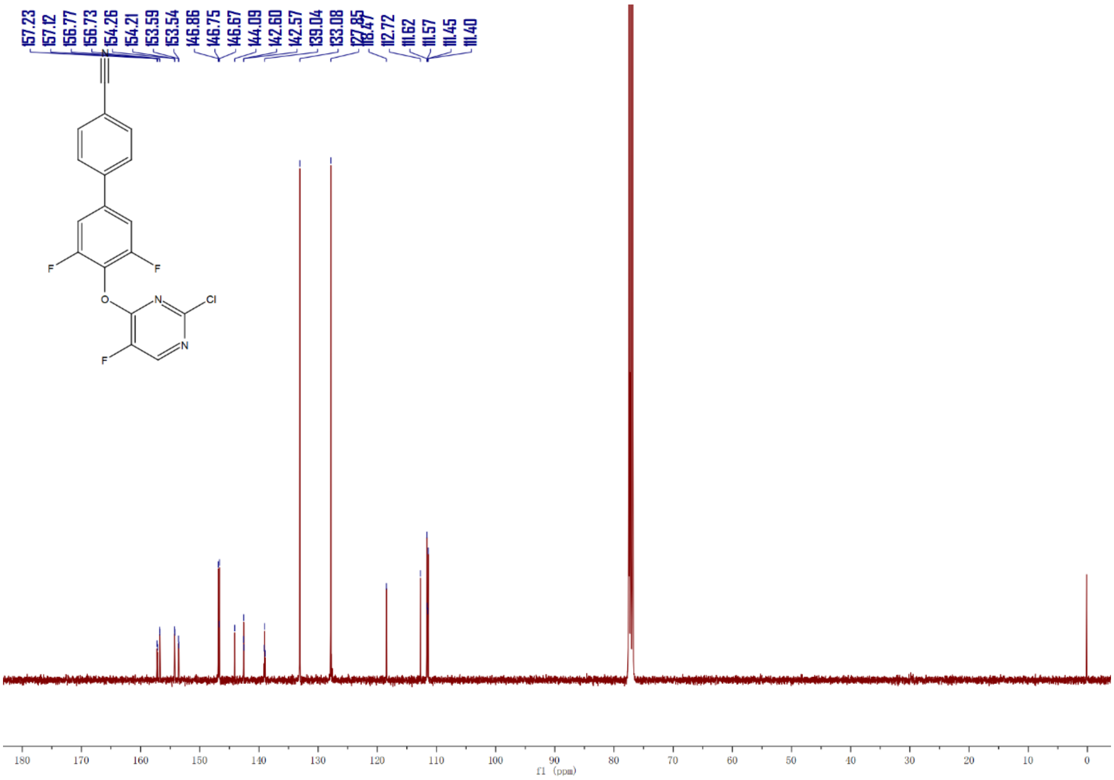

^{13}F NMR

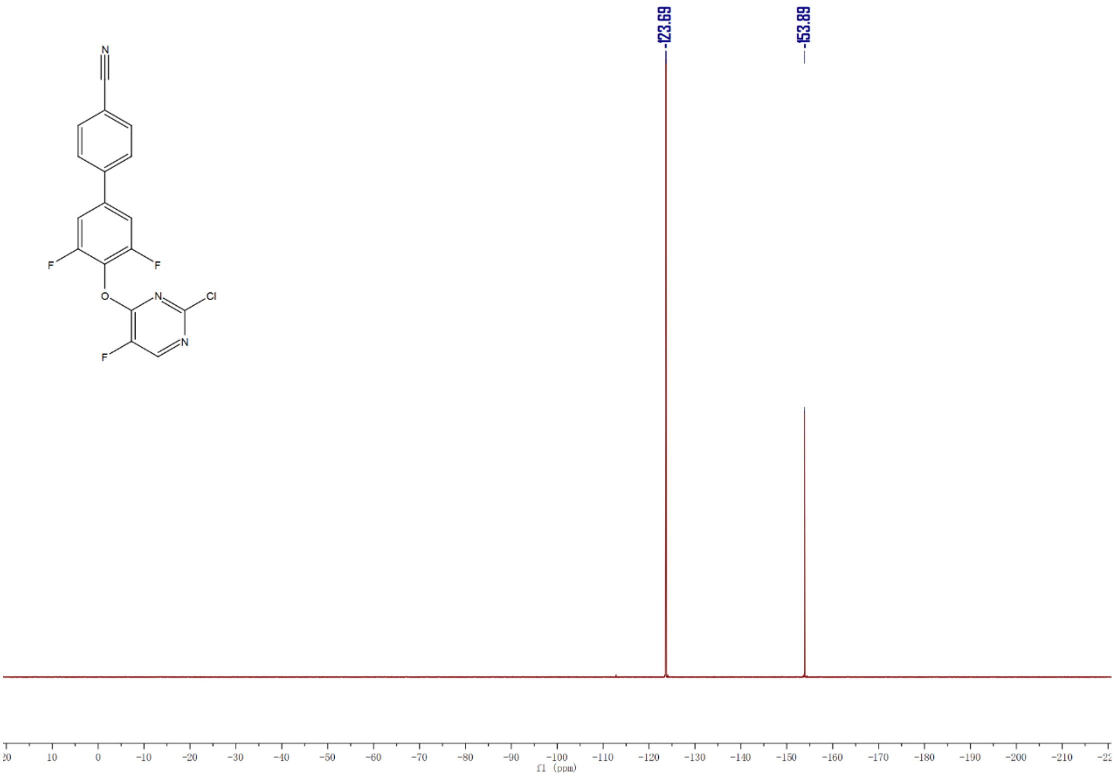

-123.69
-153.89
N
F
F
O
N
Cl
F
N
f1 (ppm)

HRMS

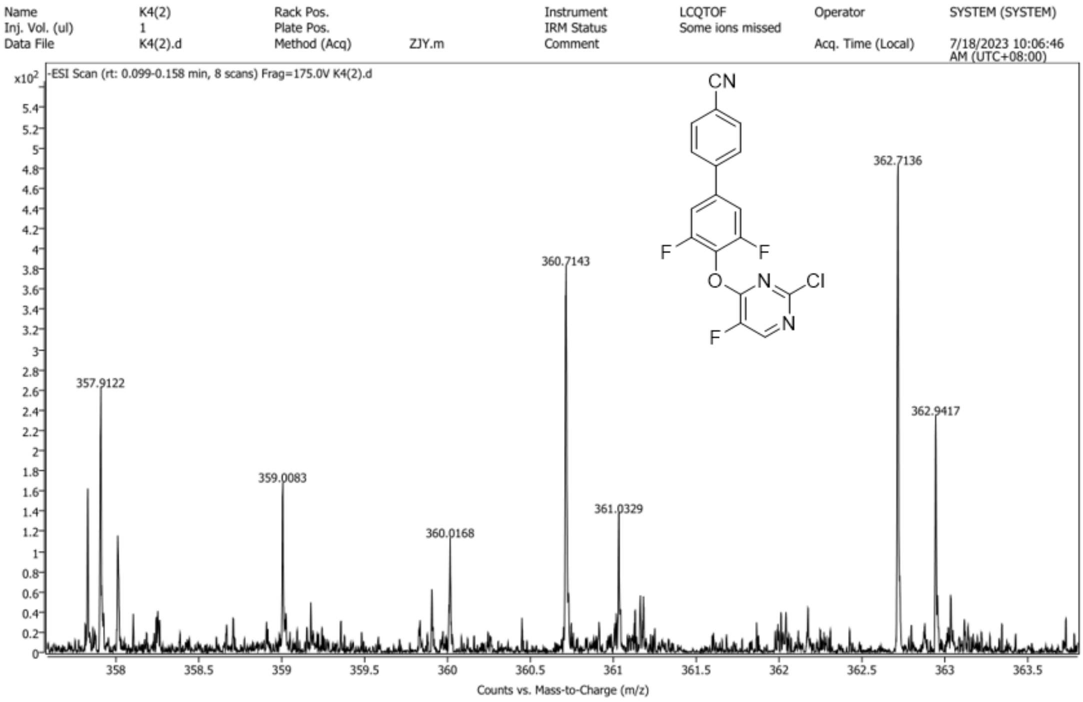

Name K4(2)
Inj. Vol. (ul) 1
Data File K4(2).d
Rack Pos.
Plate Pos.
Method (Acq) ZJY.m
Instrument LCQTOF
IRM Status Some ions missed
Comment
Operator SYSTEM (SYSTEM)
Acq. Time (Local) 7/18/2023 10:06:46 AM (UTC+08:00)
-ESI Scan (rt: 0.099-0.158 min, 8 scans) Frag=175.0V K4(2).d
CN
F
F
O
N
Cl
F
N
357.9122
359.0083
360.0168
360.7143
361.0329
362.7136
362.9417
Counts vs. Mass-to-Charge (m/z)

^{1}H NMR, ^{13}C NMR, ^{13}F NMR and HRMS spectra of 9f

^{1}H NMR

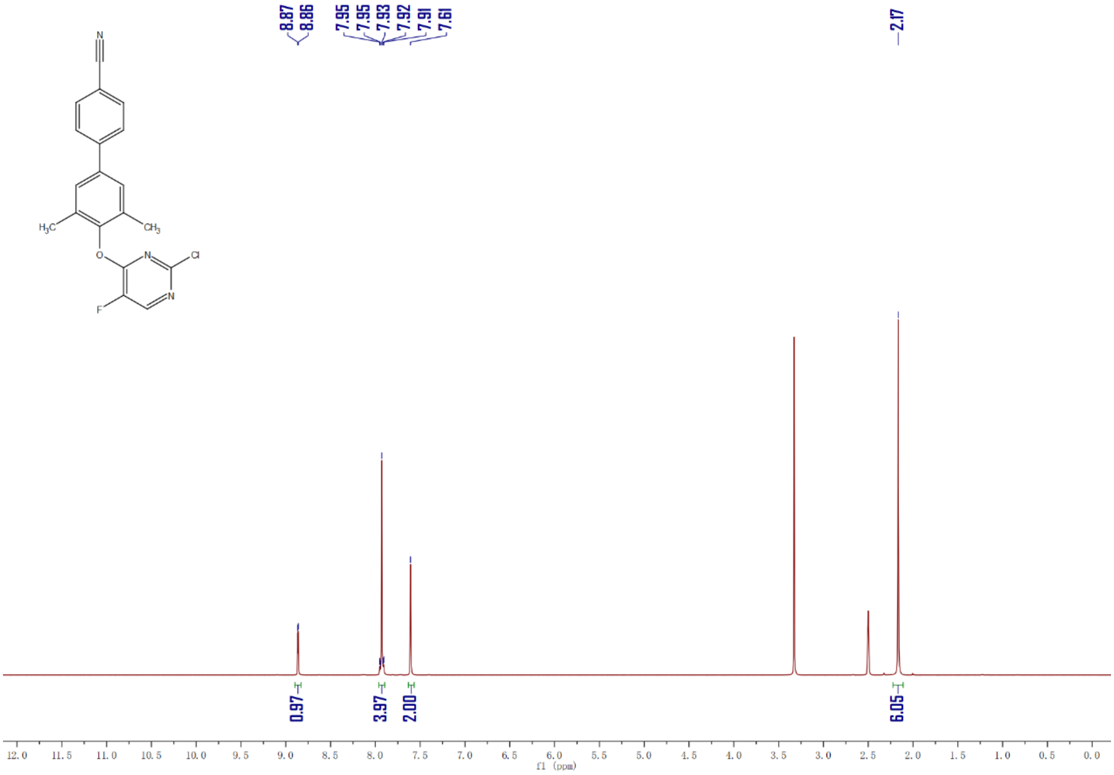

^{13}C NMR

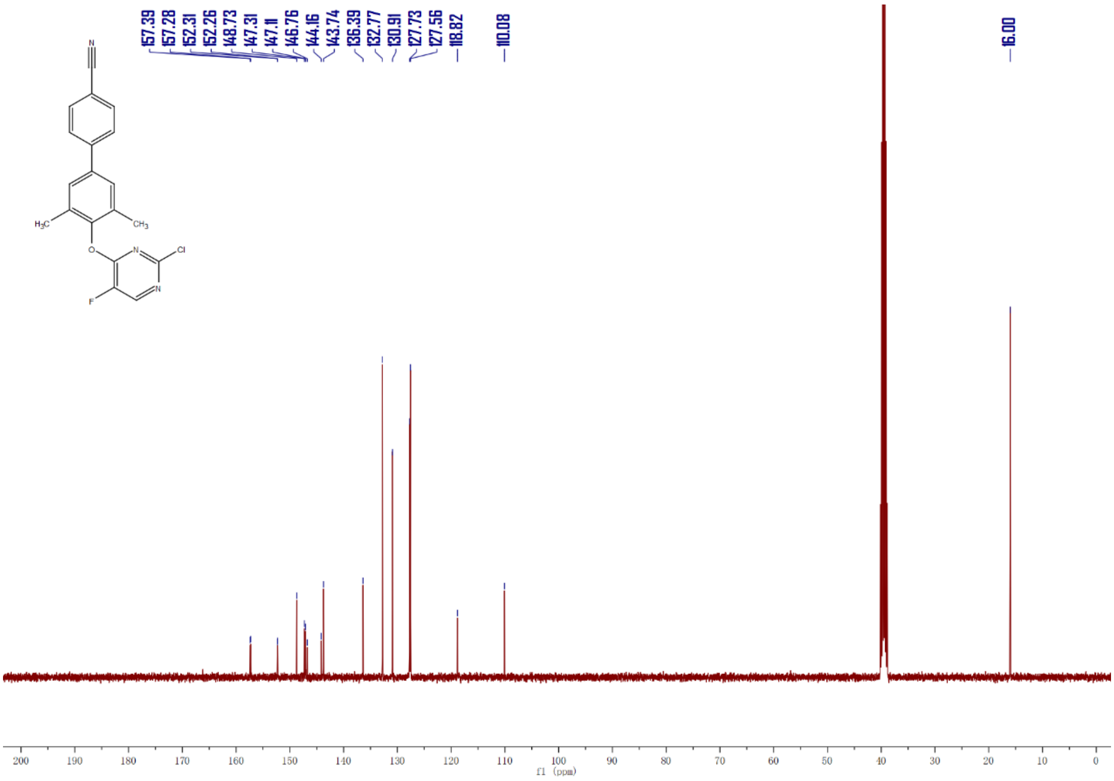

^{13}F NMR

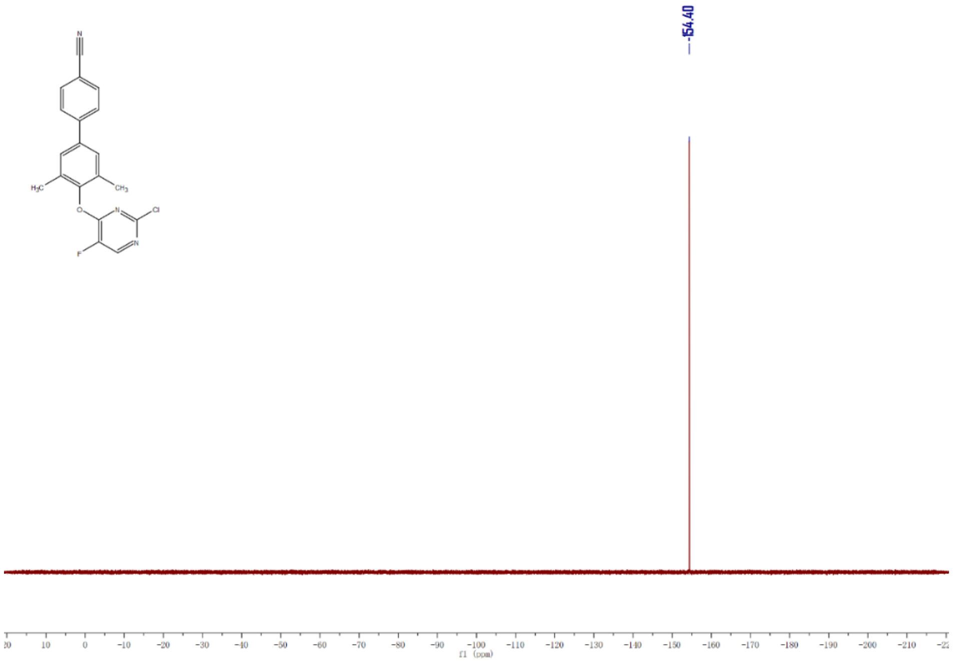

HRMS

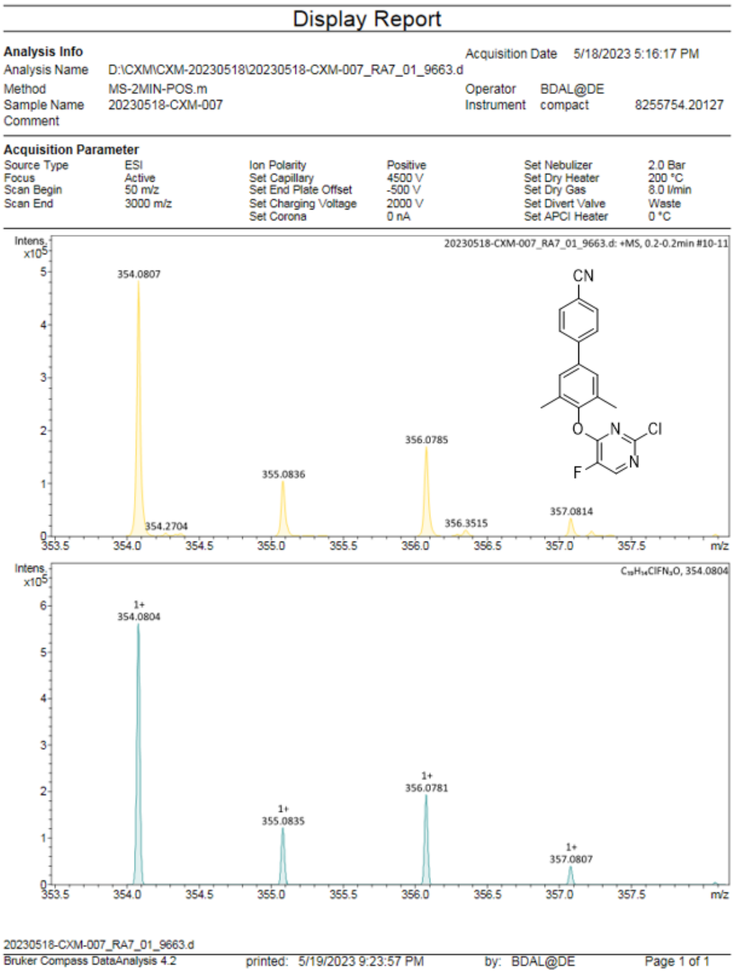
Display Report

Analysis Info

Analysis Name	D:\CXM\CXM-20230518\20230518-CXM-007_RA7_01_9663.d	Acquisition Date	5/18/2023 5:16:17 PM
Method	MS-2MIN-POS.m	Operator	BDAL@DE
Sample Name	20230518-CXM-007	Instrument	compact 8255754.20127
Comment			

Acquisition Parameter

Source Type	ESI	Ion Polarity	Positive	Set Nebulizer	2.0 Bar
Focus	Active	Set Capillary	4500 V	Set Dry Heater	200 °C
Scan Begin	50 m/z	Set End Plate Offset	-500 V	Set Dry Gas	8.0 l/min
Scan End	3000 m/z	Set Charging Voltage	2000 V	Set Divert Valve	Waste
		Set Corona	0 nA	Set APCI Heater	0 °C

20230518-CXM-007_RA7_01_9663.d
Bruker Compass DataAnalysis 4.2 printed: 5/19/2023 9:23:57 PM by: BDAL@DE Page 1 of 1

^{1}H NMR, ^{13}C NMR, ^{13}F NMR and HRMS spectra of 9g

^{1}H NMR

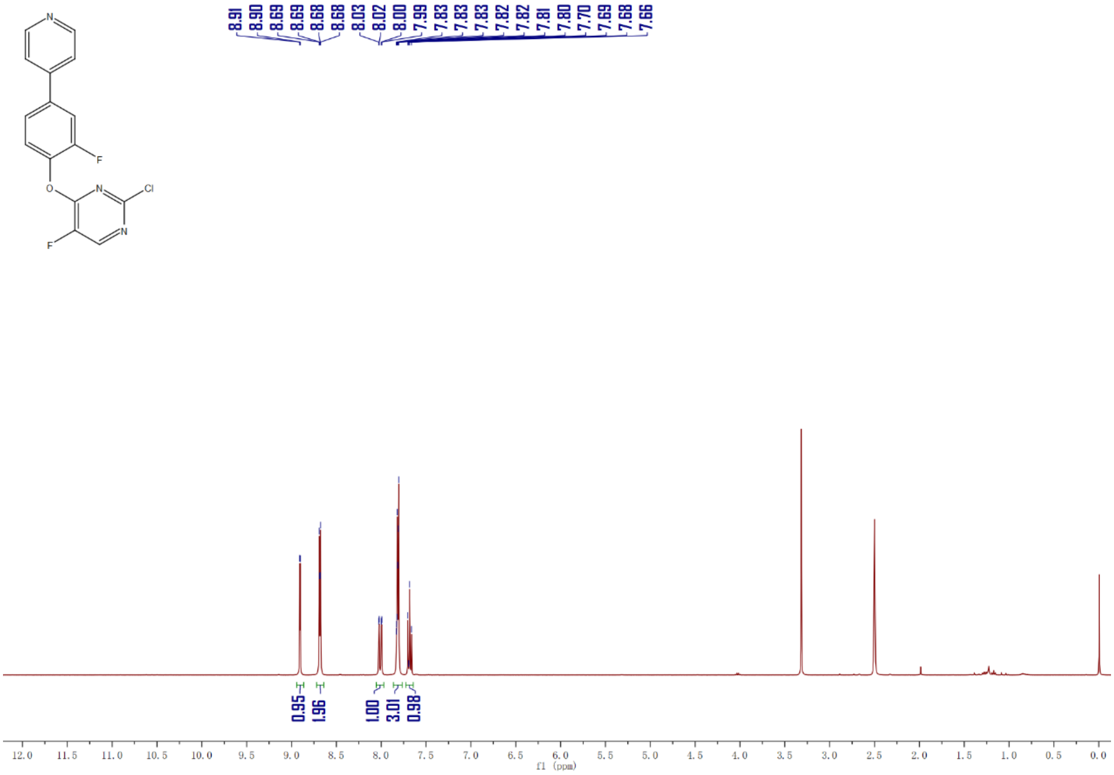

^{13}C NMR

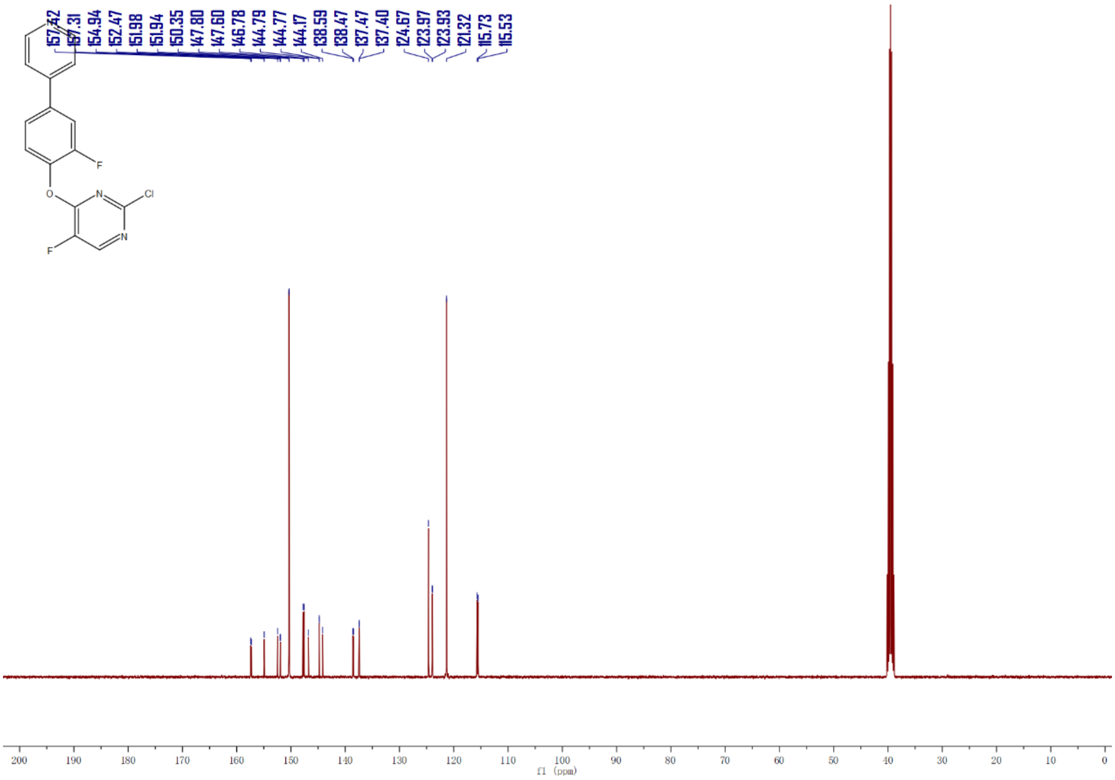

^{13}F NMR

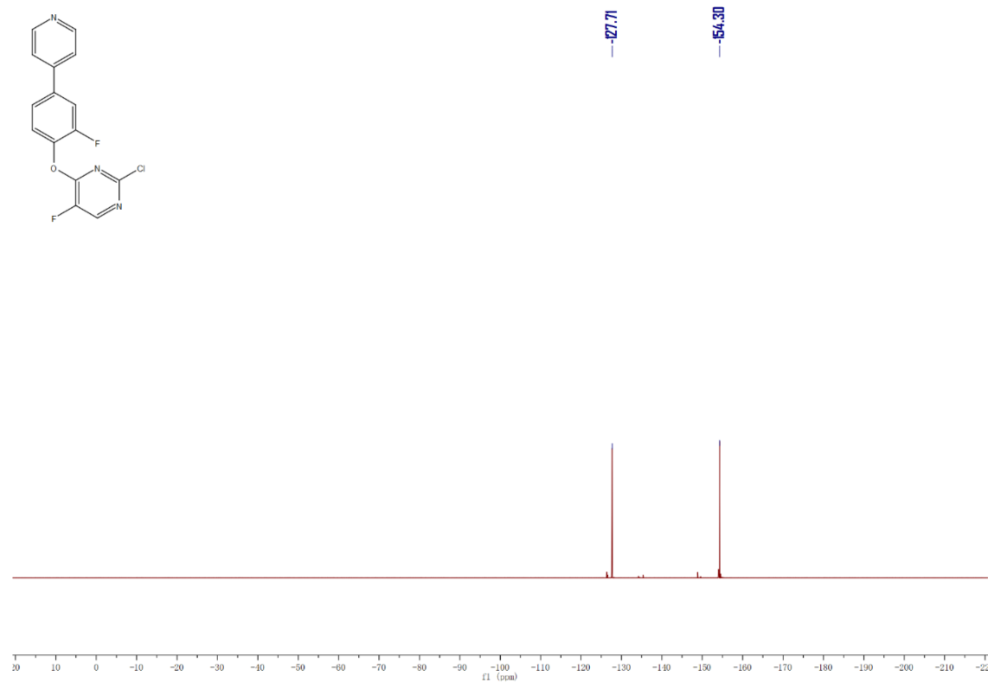

-127.71
-154.30
f1 (ppm)

HRMS

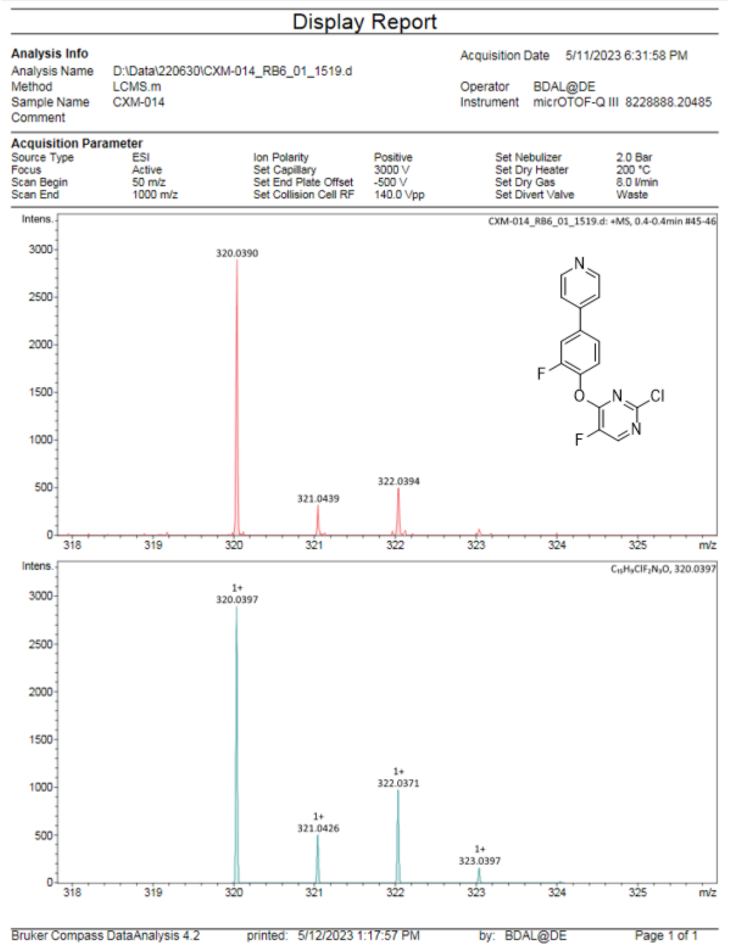

Display Report
Analysis Info
Analysis Name D:\Data\220630\CXM-014_RB6_01_1519.d
Method LCMS.m
Sample Name CXM-014
Comment
Acquisition Date 5/11/2023 6:31:58 PM
Operator BDAL@DE
Instrument micrOTOF-Q III 8228888.20485
Acquisition Parameter
Source Type ESI
Focus Active
Scan Begin 50 m/z
Scan End 1000 m/z
Ion Polarity Positive
Set Capillary 3000 V
Set End Plate Offset -500 V
Set Collision Cell RF 140.0 Vpp
Set Nebulizer 2.0 Bar
Set Dry Heater 200 °C
Set Dry Gas 8.0 l/min
Set Divert Valve Waste
CXM-014_RB6_01_1519.d: +MS, 0.4-0.4min #45-46
320.0390
321.0439
322.0394
C15H9ClF2N3O, 320.0397
320.0397
321.0426
322.0371
323.0397
Intens.
m/z
Bruker Compass DataAnalysis 4.2
printed: 5/12/2023 1:17:57 PM
by: BDAL@DE
Page 1 of 1

^{1}H NMR, ^{13}C NMR, [13]F NMR and HRMS spectra of 9h

^{1}H NMR

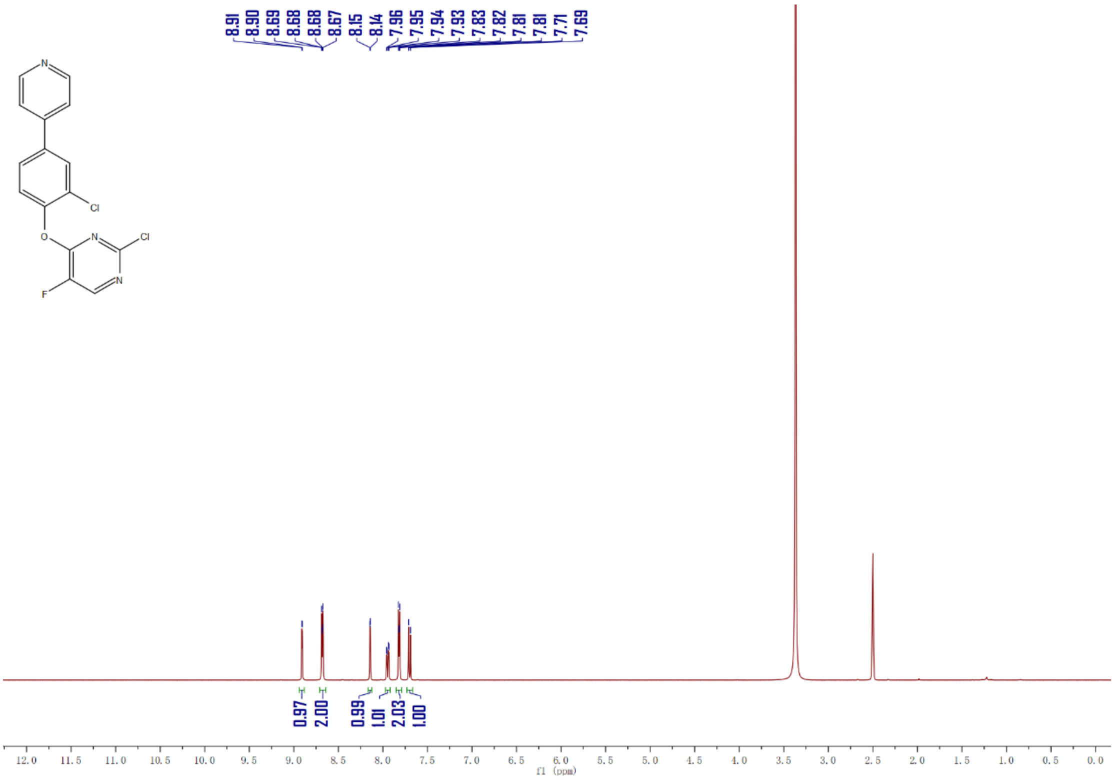

^{13}C NMR

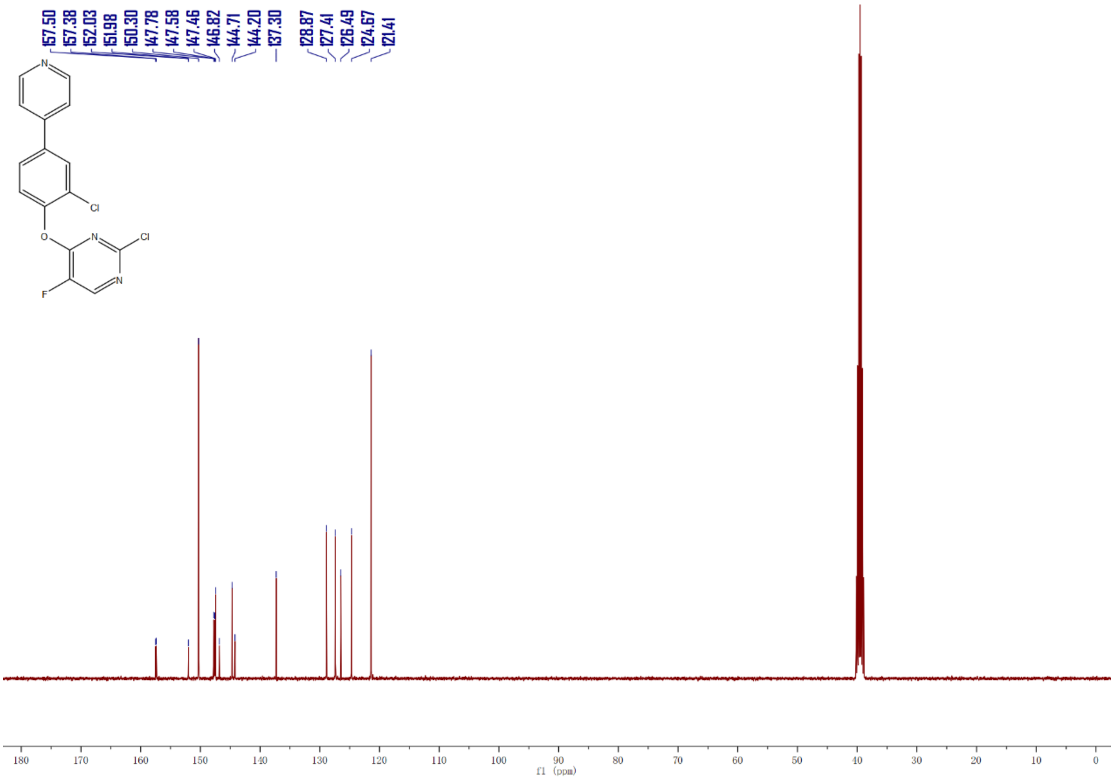

13F NMR

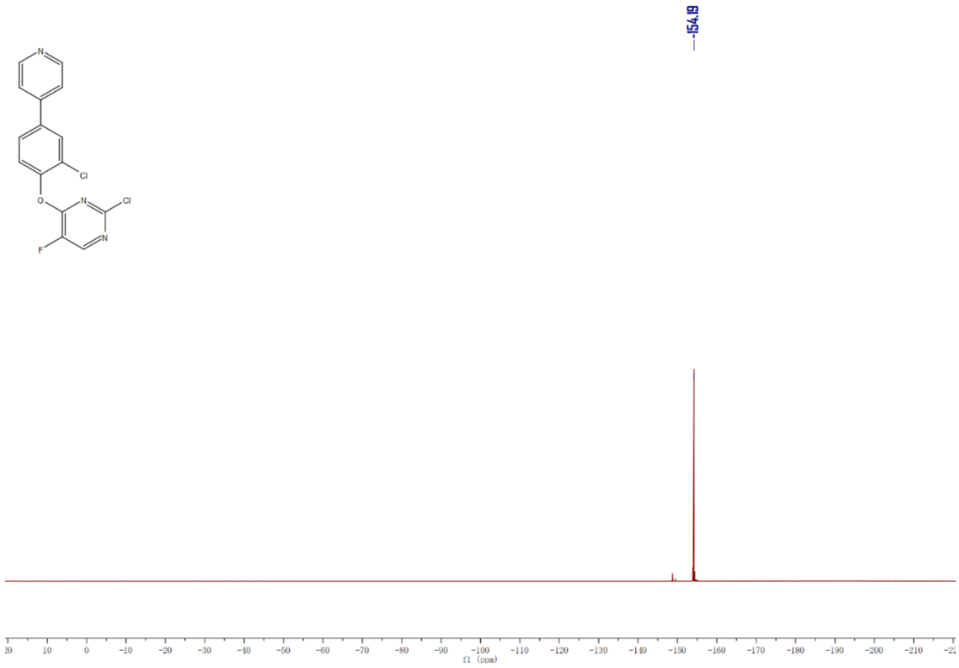

HRMS

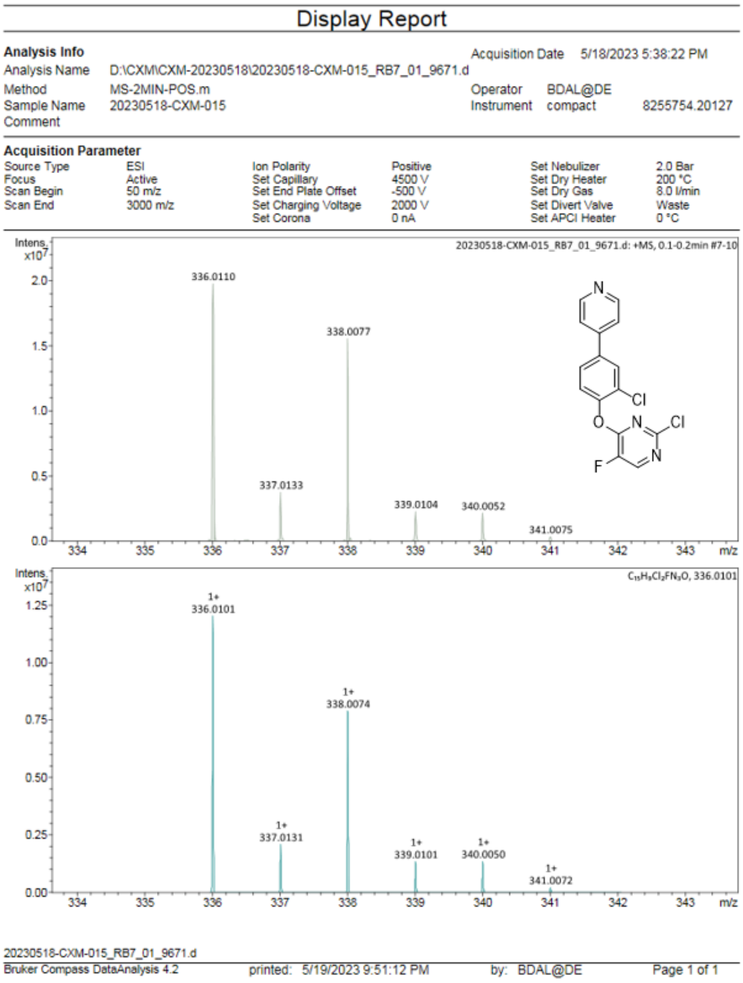

Display Report

Analysis Info

Analysis Name	D:\CXM\CXM-20230518\20230518-CXM-015_RB7_01_9671.d	Acquisition Date	5/18/2023 5:38:22 PM
Method	MS-2MIN-POS.m	Operator	BDAL@DE
Sample Name	20230518-CXM-015	Instrument	compact 8255754.20127
Comment			

Acquisition Parameter

Source Type	ESI	Ion Polarity	Positive	Set Nebulizer	2.0 Bar
Focus	Active	Set Capillary	4500 V	Set Dry Heater	200 °C
Scan Begin	50 m/z	Set End Plate Offset	-500 V	Set Dry Gas	8.0 l/min
Scan End	3000 m/z	Set Charging Voltage	2000 V	Set Divert Valve	Waste
		Set Corona	0 nA	Set APCI Heater	0 °C

20230518-CXM-015_RB7_01_9671.d

Bruker Compass DataAnalysis 4.2 printed: 5/19/2023 9:51:12 PM by: BDAL@DE Page 1 of 1

^{1}H NMR, ^{13}C NMR, ^{13}F NMR and HRMS spectra of 9i

^{1}H NMR

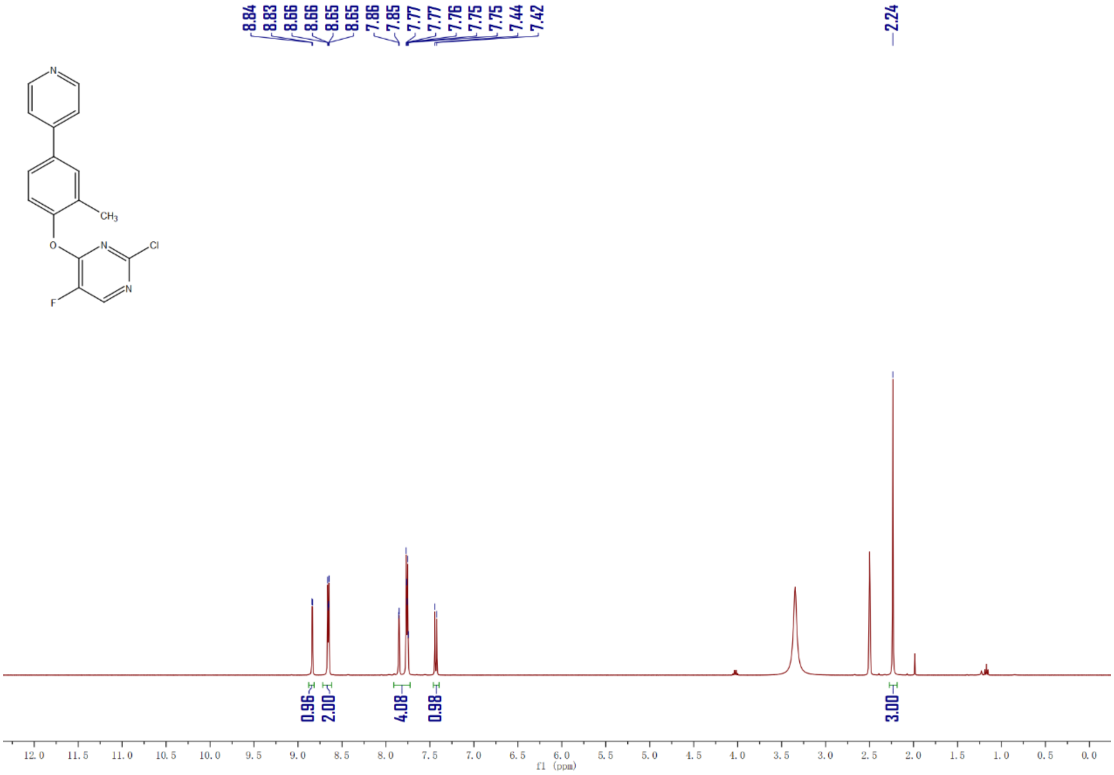

^{13}C NMR

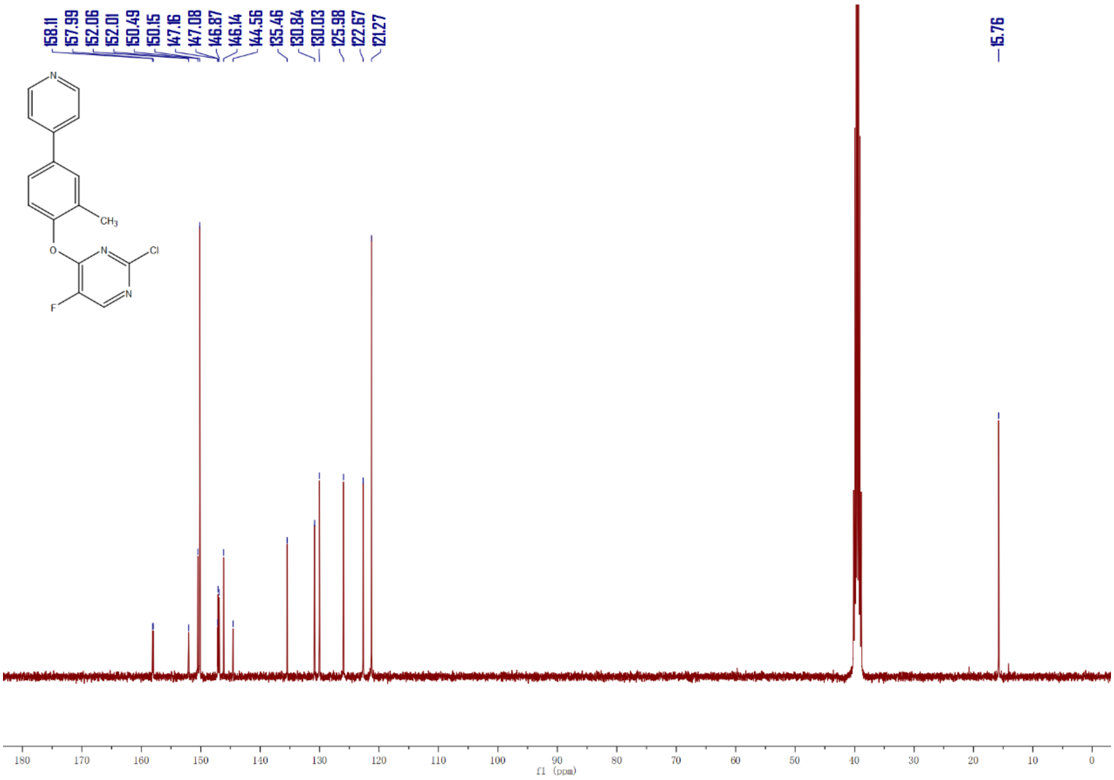

^{13}F NMR

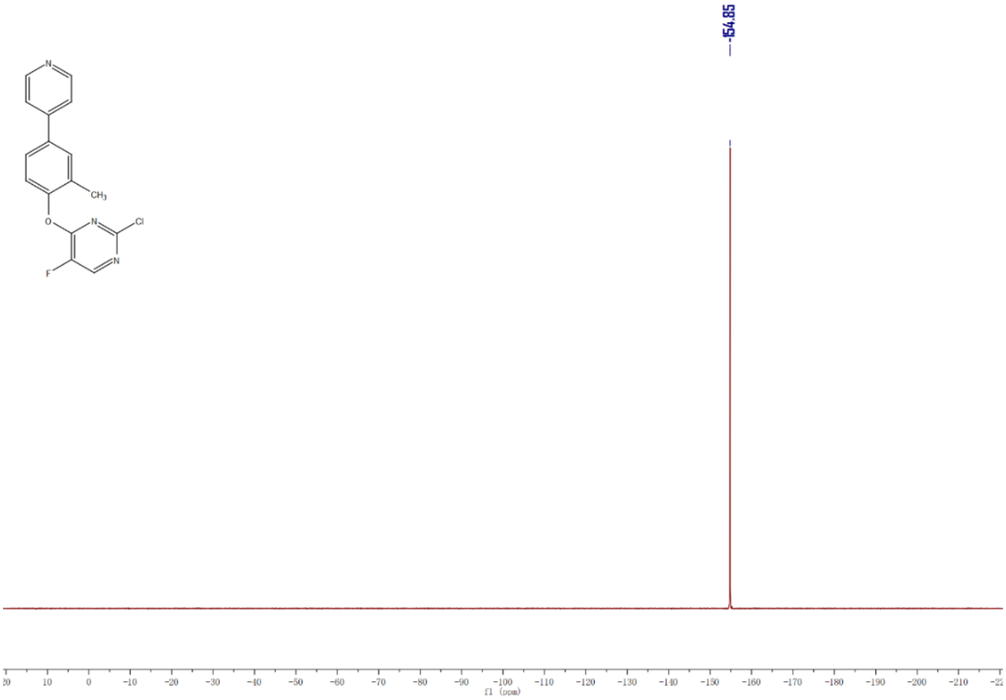

-154.85
CH3
Cl
N
O
F
f1 (ppm)

HRMS

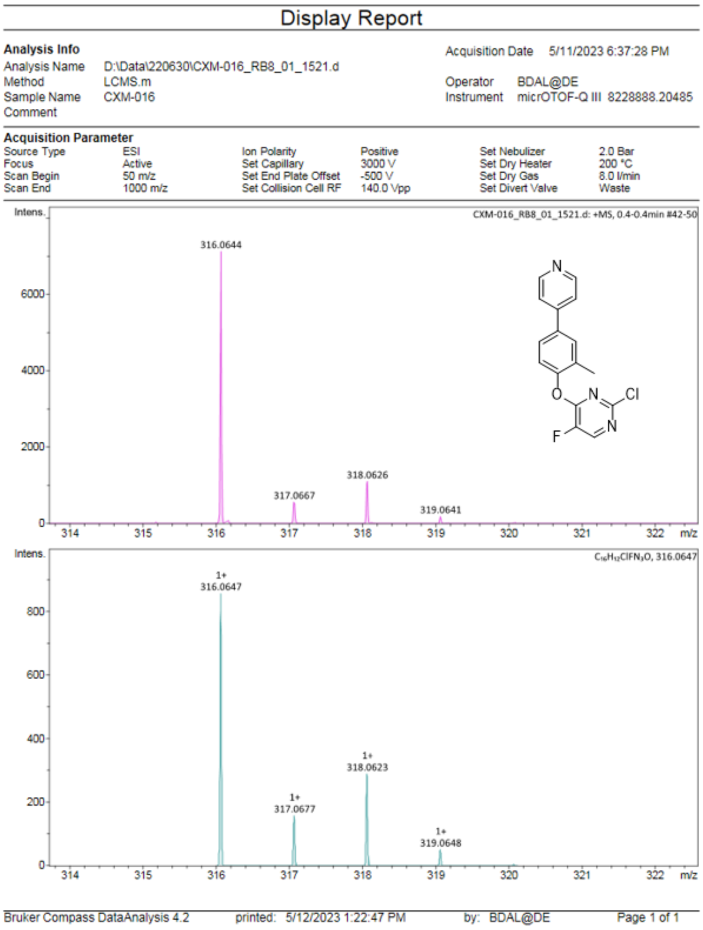

Display Report
Analysis Info
Analysis Name D:\Data\220630\CXM-016_RB8_01_1521.d
Method LCMS.m
Sample Name CXM-016
Comment
Acquisition Date 5/11/2023 6:37:28 PM
Operator BDAL@DE
Instrument micrOTOF-Q III 8228888.20485
Acquisition Parameter
Source Type ESI
Focus Active
Scan Begin 50 m/z
Scan End 1000 m/z
Ion Polarity Positive
Set Capillary 3000 V
Set End Plate Offset -500 V
Set Collision Cell RF 140.0 Vpp
Set Nebulizer 2.0 Bar
Set Dry Heater 200 °C
Set Dry Gas 8.0 l/min
Set Divert Valve Waste
Intens.
CXM-016_RB8_01_1521.d: +MS, 0.4-0.4min #42-50
316.0644
317.0667
318.0626
319.0641
m/z
C16H12ClFN3O, 316.0647
1+ 316.0647
1+ 317.0677
1+ 318.0623
1+ 319.0648
Bruker Compass DataAnalysis 4.2
printed: 5/12/2023 1:22:47 PM
by: BDAL@DE
Page 1 of 1

^{1}H NMR, ^{13}C NMR, [13]F NMR and HRMS spectra of 9j

^{1}H NMR

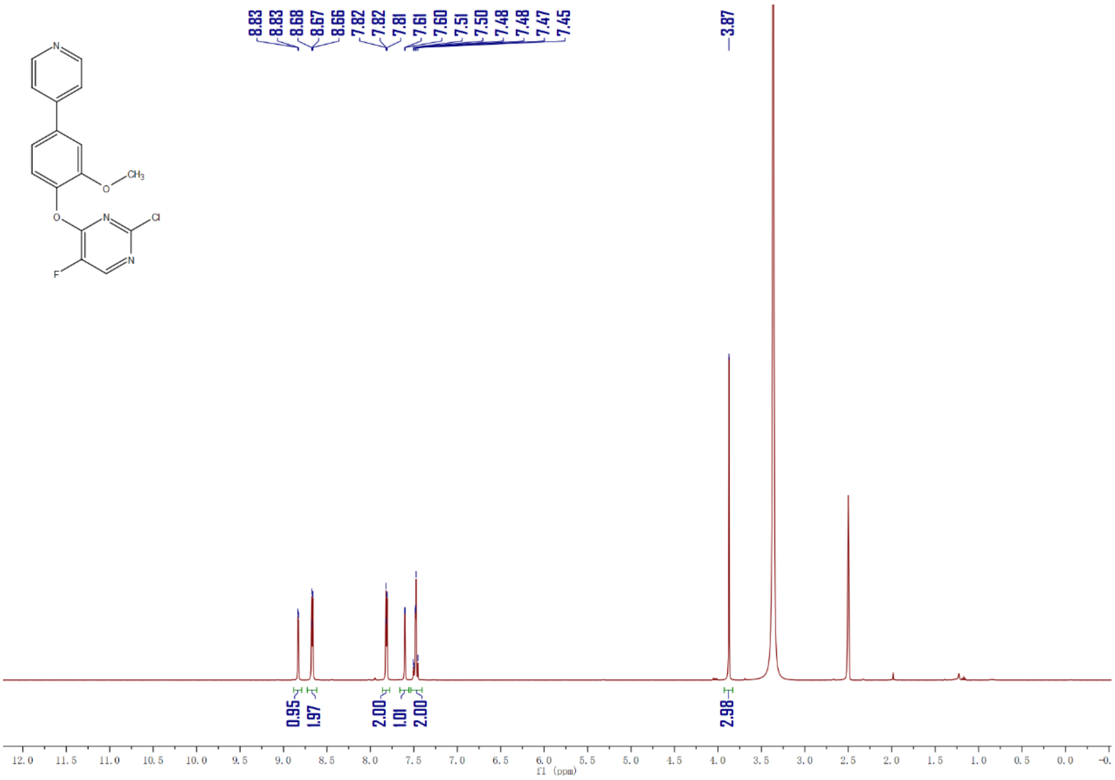

^{13}C NMR

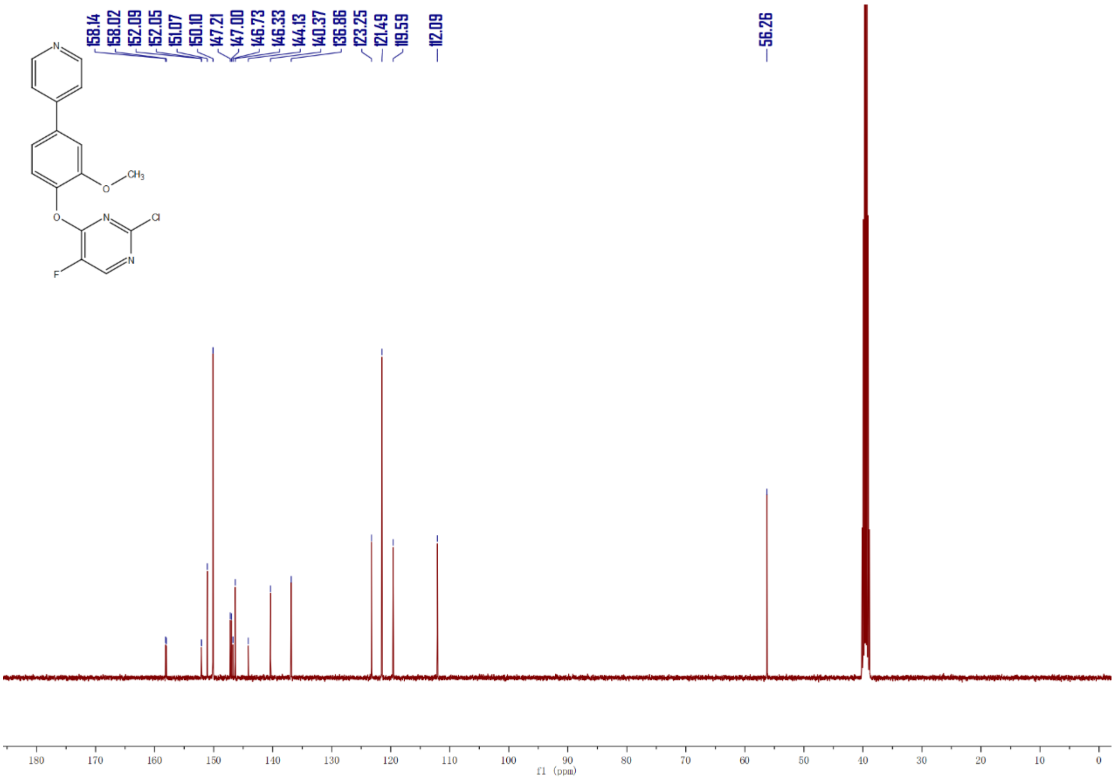

^{13}F NMR

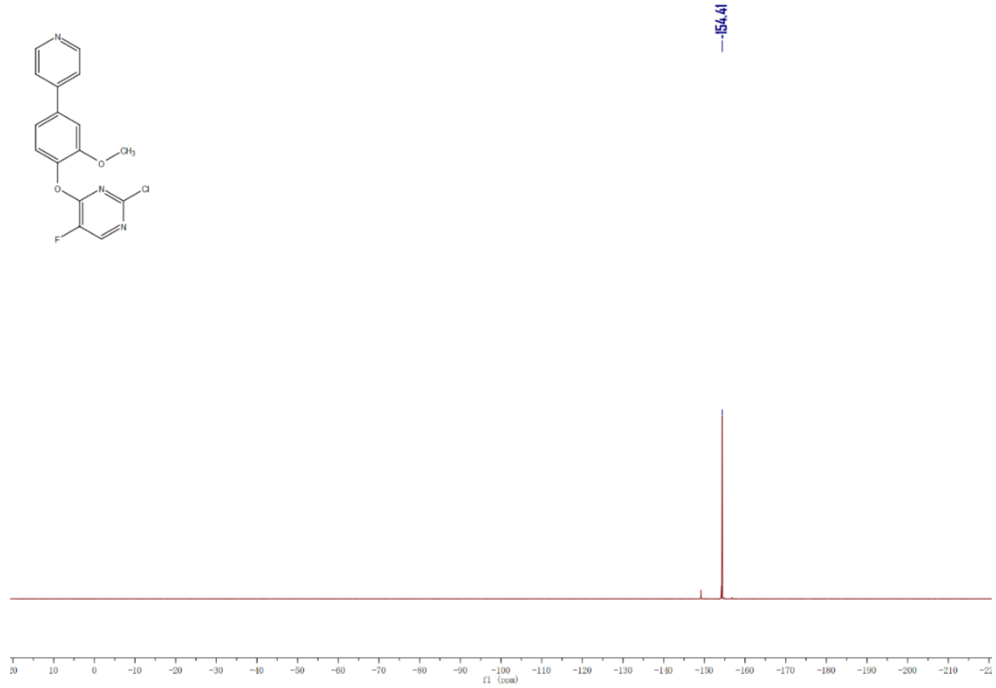

-154.41
f1 (ppm)

HRMS

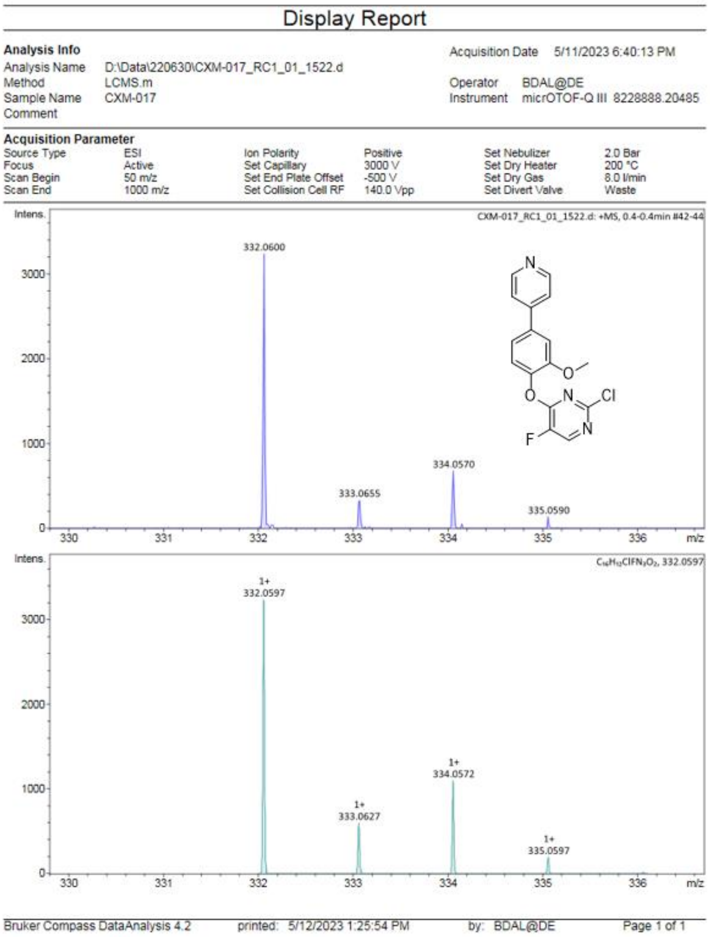

Display Report
Analysis Info
Analysis Name D:\Data\220630\CXM-017_RC1_01_1522.d
Method LCMS.m
Sample Name CXM-017
Comment
Acquisition Date 5/11/2023 6:40:13 PM
Operator BDAL@DE
Instrument micrOTOF-Q III 8228888.20485
Acquisition Parameter
Source Type ESI
Focus Active
Scan Begin 50 m/z
Scan End 1000 m/z
Ion Polarity Positive
Set Capillary 3000 V
Set End Plate Offset -500 V
Set Collision Cell RF 140.0 Vpp
Set Nebulizer 2.0 Bar
Set Dry Heater 200 °C
Set Dry Gas 8.0 l/min
Set Divert Valve Waste
CXM-017_RC1_01_1522.d: +MS, 0.4-0.4min #42-44
332.0600
333.0655
334.0570
335.0590
$C_{16}H_{11}ClFN_3O_2$, 332.0597
1+ 332.0597
1+ 333.0627
1+ 334.0572
1+ 335.0597
Bruker Compass DataAnalysis 4.2
printed: 5/12/2023 1:25:54 PM
by: BDAL@DE
Page 1 of 1

^{1}H NMR, ^{13}C NMR, ^{13}F NMR and HRMS spectra of 9k

^{1}H NMR

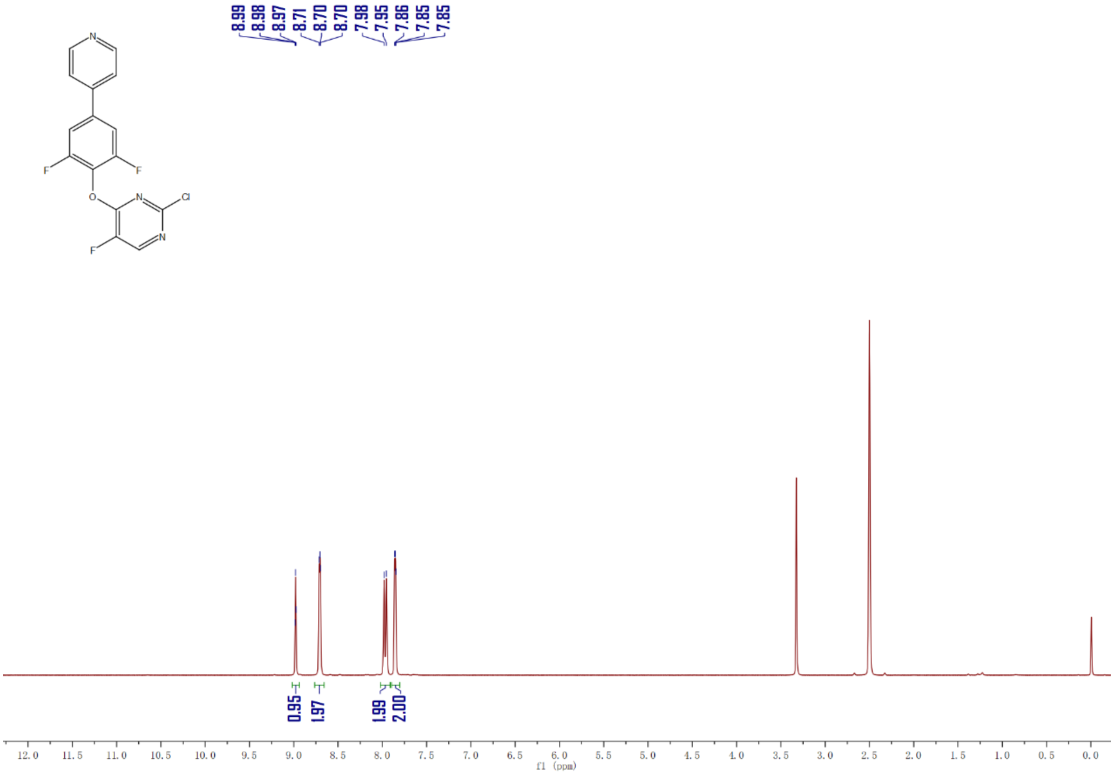

^{13}C NMR

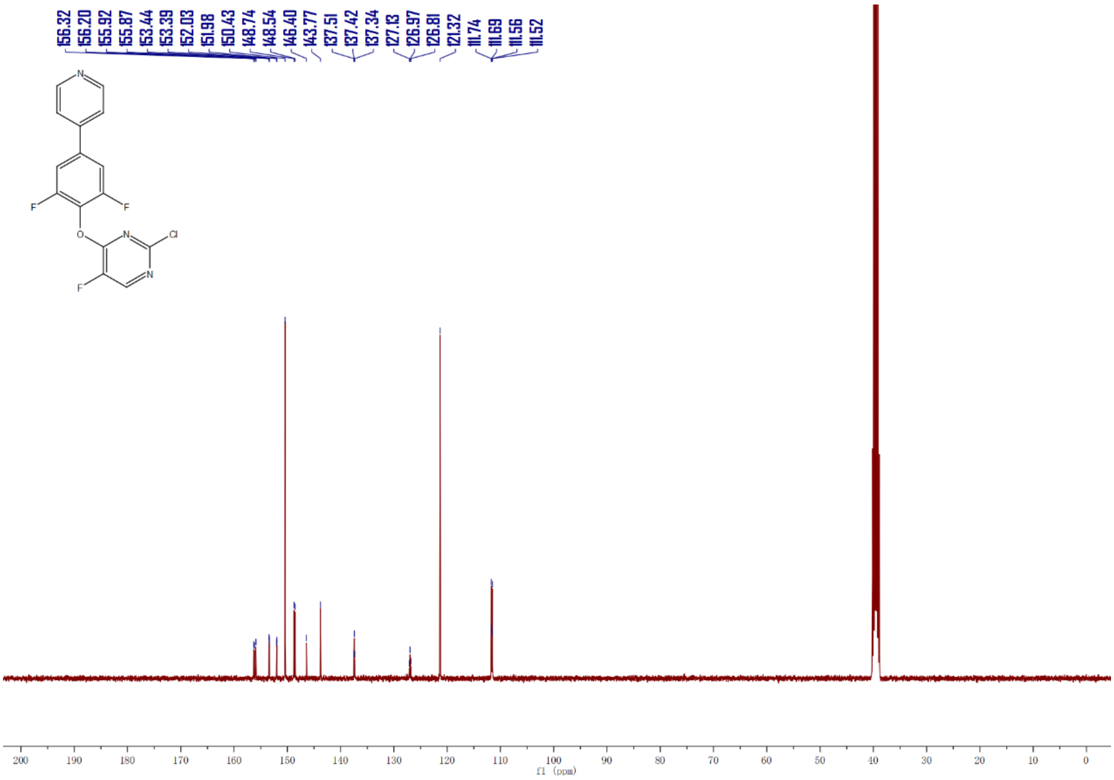

^{13}F NMR

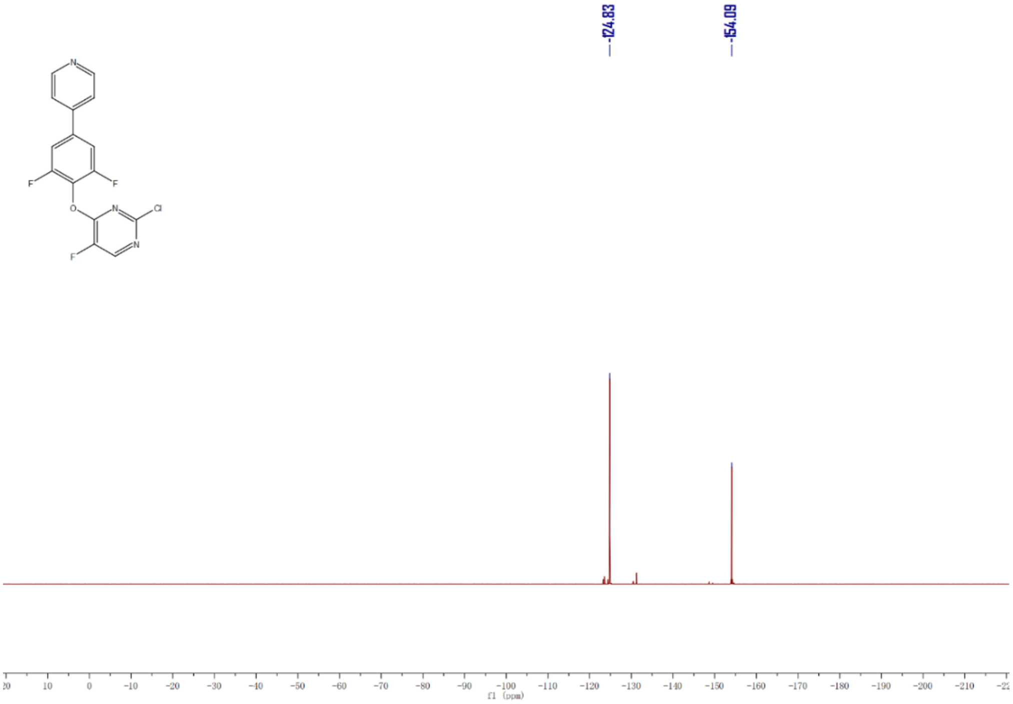

-124.83
-154.09
f1 (ppm)

HRMS

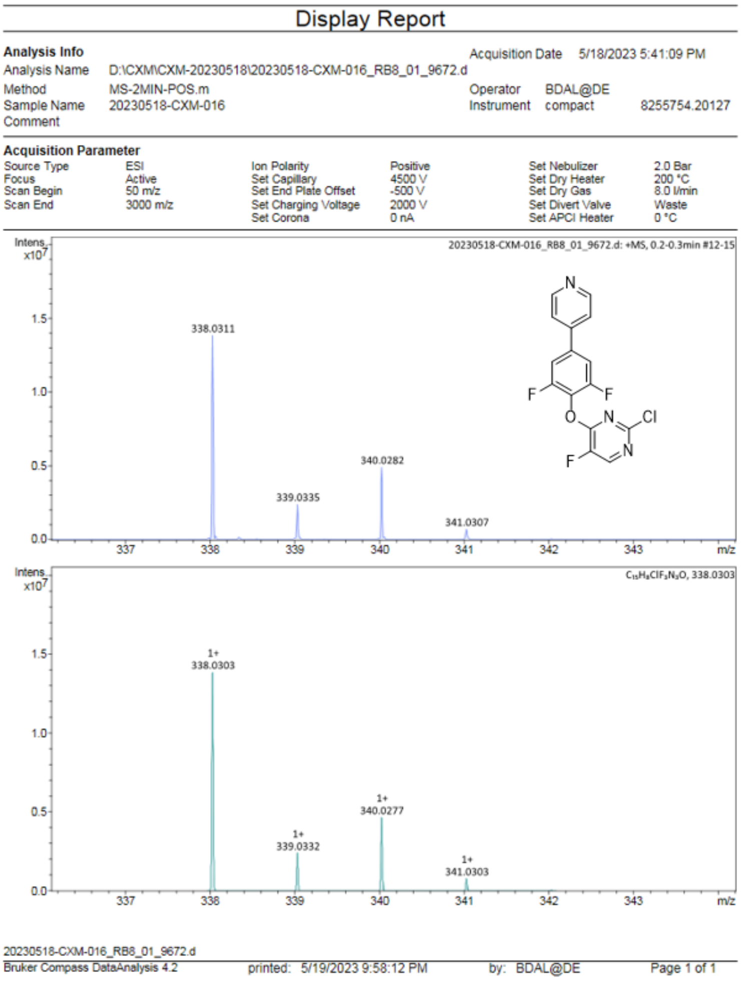

Display Report
Analysis Info
Analysis Name D:\CXM\CXM-20230518\20230518-CXM-016_RB8_01_9672.d
Method MS-2MIN-POS.m
Sample Name 20230518-CXM-016
Comment
Acquisition Date 5/18/2023 5:41:09 PM
Operator BDAL@DE
Instrument compact 8255754.20127
Acquisition Parameter
Source Type ESI
Focus Active
Scan Begin 50 m/z
Scan End 3000 m/z
Ion Polarity Positive
Set Capillary 4500 V
Set End Plate Offset -500 V
Set Charging Voltage 2000 V
Set Corona 0 nA
Set Nebulizer 2.0 Bar
Set Dry Heater 200 °C
Set Dry Gas 8.0 l/min
Set Divert Valve Waste
Set APCI Heater 0 °C
Intens. x10^7
20230518-CXM-016_RB8_01_9672.d: +MS, 0.2-0.3min #12-15
338.0311
339.0335
340.0282
341.0307
m/z
C15H8ClF3N3O, 338.0303
1+ 338.0303
1+ 339.0332
1+ 340.0277
1+ 341.0303
20230518-CXM-016_RB8_01_9672.d
Bruker Compass DataAnalysis 4.2 printed: 5/19/2023 9:58:12 PM by: BDAL@DE Page 1 of 1

^{1}H NMR, ^{13}C NMR, [13]F NMR and HRMS spectra of 9l

^{1}H NMR

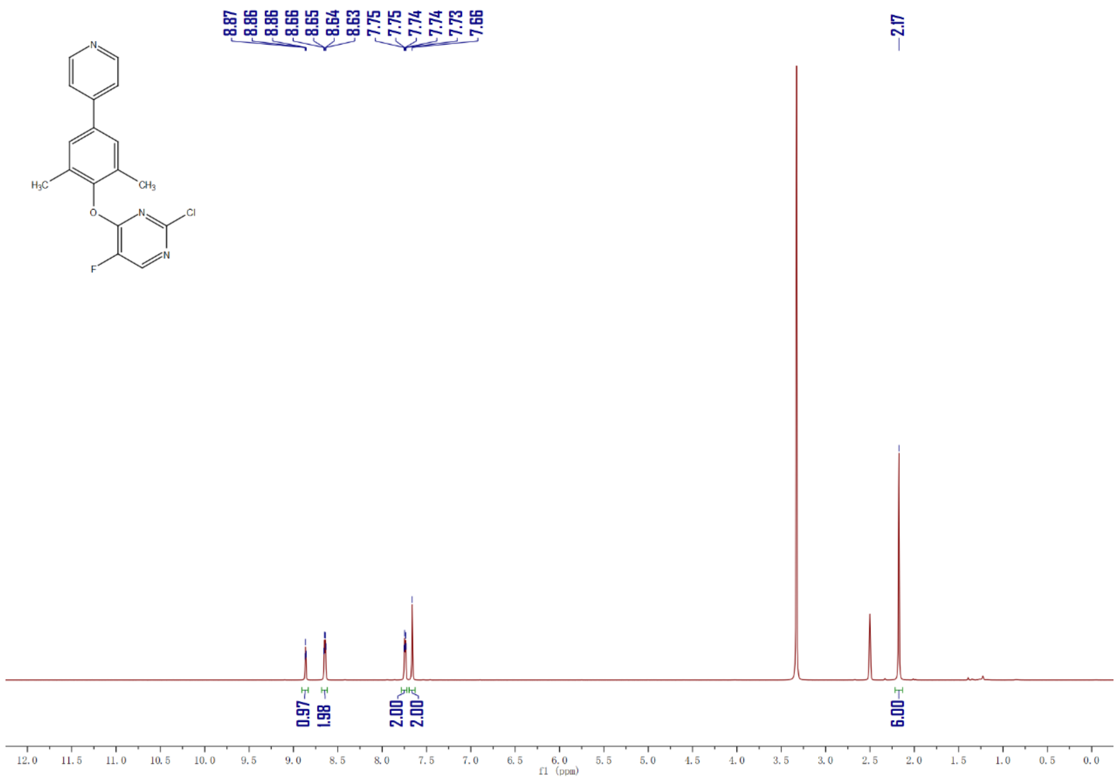

^{13}C NMR

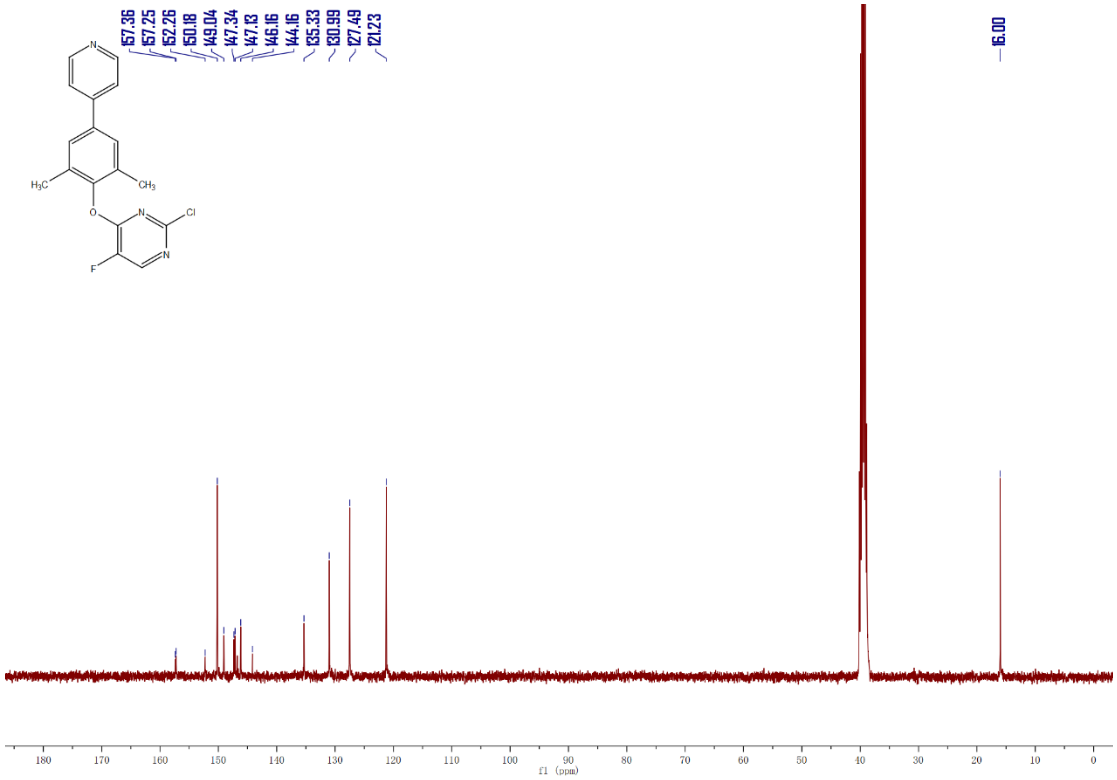

^{13}F NMR

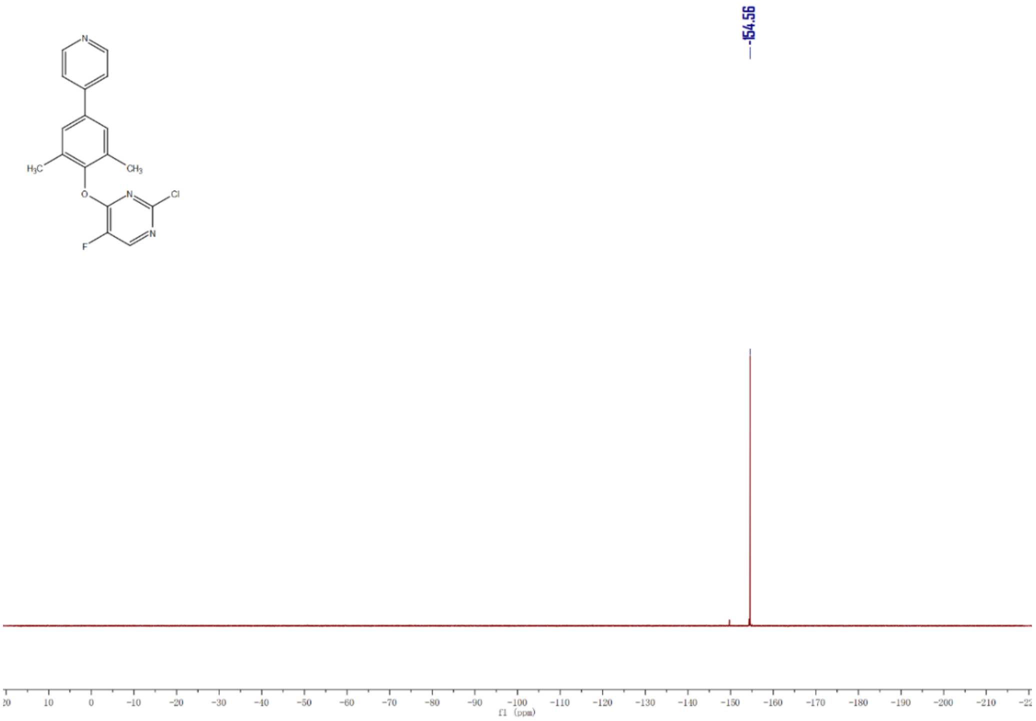
-154.56
f1 (ppm)

HRMS

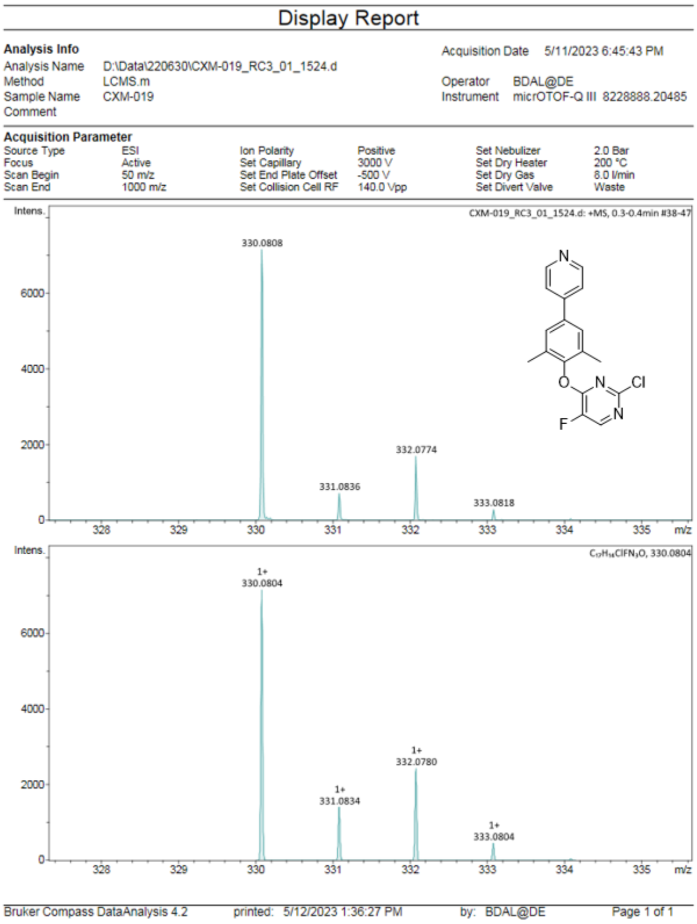
Display Report
Analysis Info
Analysis Name D:\Data\220630\CXM-019_RC3_01_1524.d
Method LCMS.m
Sample Name CXM-019
Comment
Acquisition Date 5/11/2023 6:45:43 PM
Operator BDAL@DE
Instrument micrOTOF-Q III 8228888.20485
Acquisition Parameter
Source Type ESI
Focus Active
Scan Begin 50 m/z
Scan End 1000 m/z
Ion Polarity Positive
Set Capillary 3000 V
Set End Plate Offset -500 V
Set Collision Cell RF 140.0 Vpp
Set Nebulizer 2.0 Bar
Set Dry Heater 200 °C
Set Dry Gas 8.0 l/min
Set Divert Valve Waste
CXM-019_RC3_01_1524.d: +MS, 0.3-0.4min #38-47
330.0808
331.0836
332.0774
333.0818
C17H14ClFN3O, 330.0804
330.0804
331.0834
332.0780
333.0804
Bruker Compass DataAnalysis 4.2
printed: 5/12/2023 1:36:27 PM
by: BDAL@DE
Page 1 of 1

^{1}H NMR, ^{13}C NMR, ^{13}F NMR and HRMS spectra of 9m

^{1}H NMR

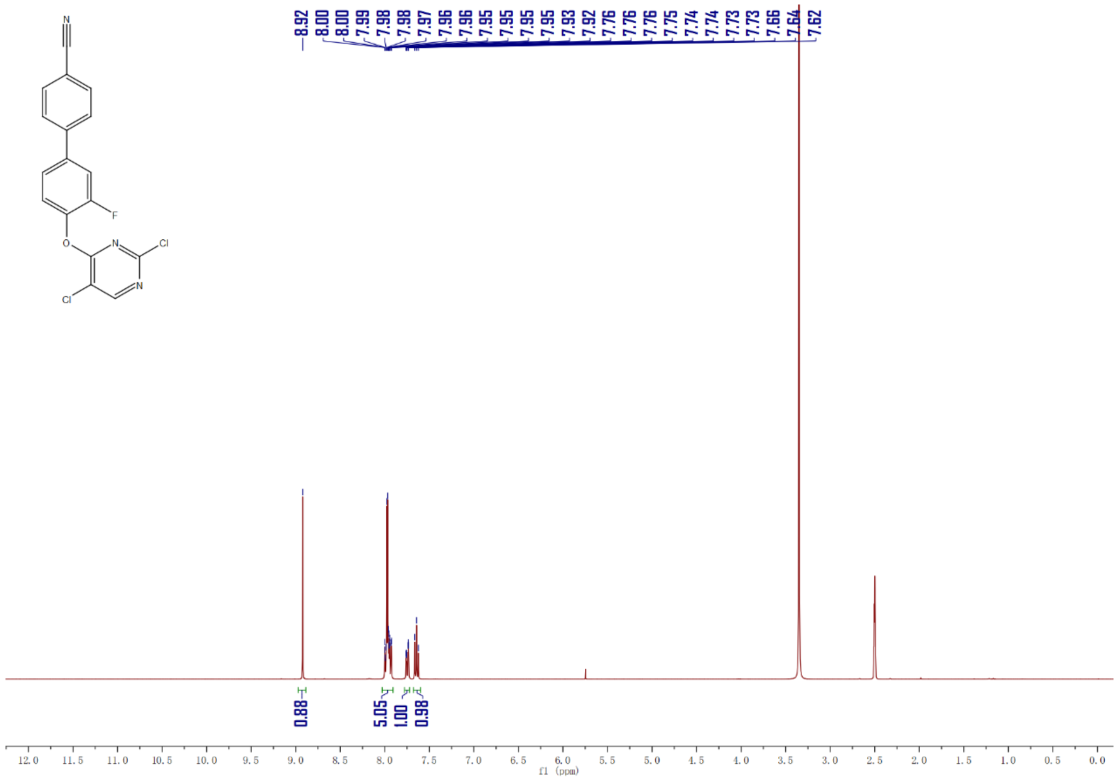

^{13}C NMR

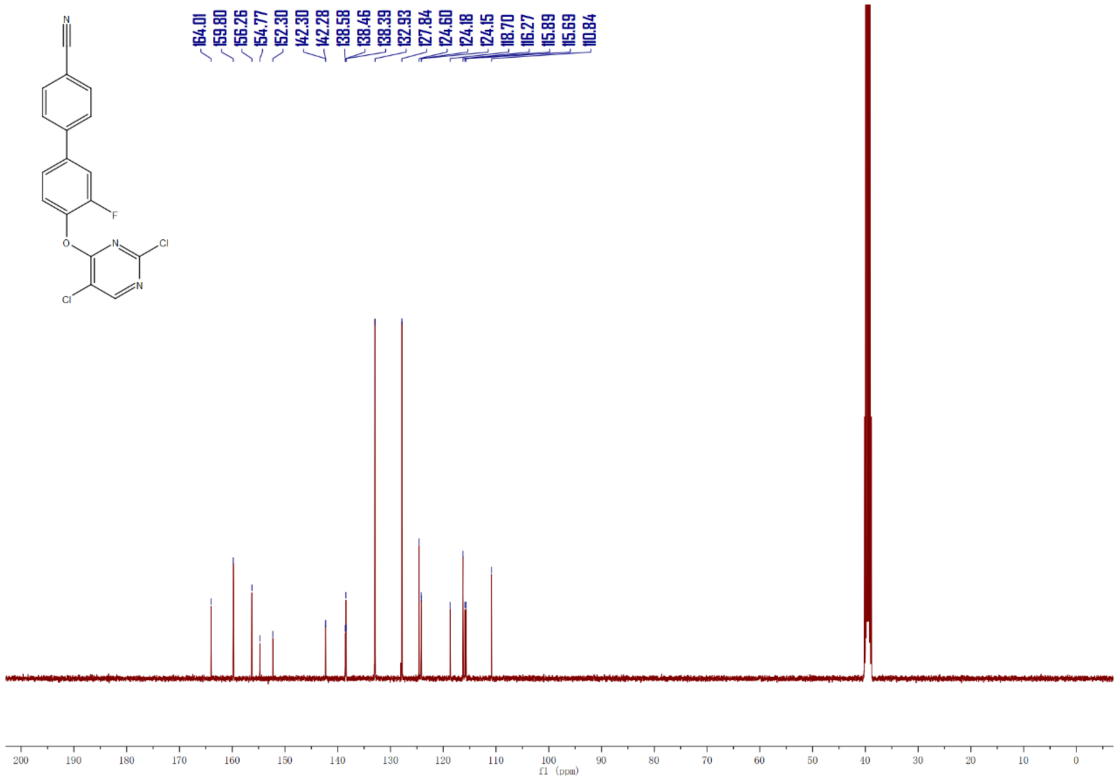

^{13}F NMR

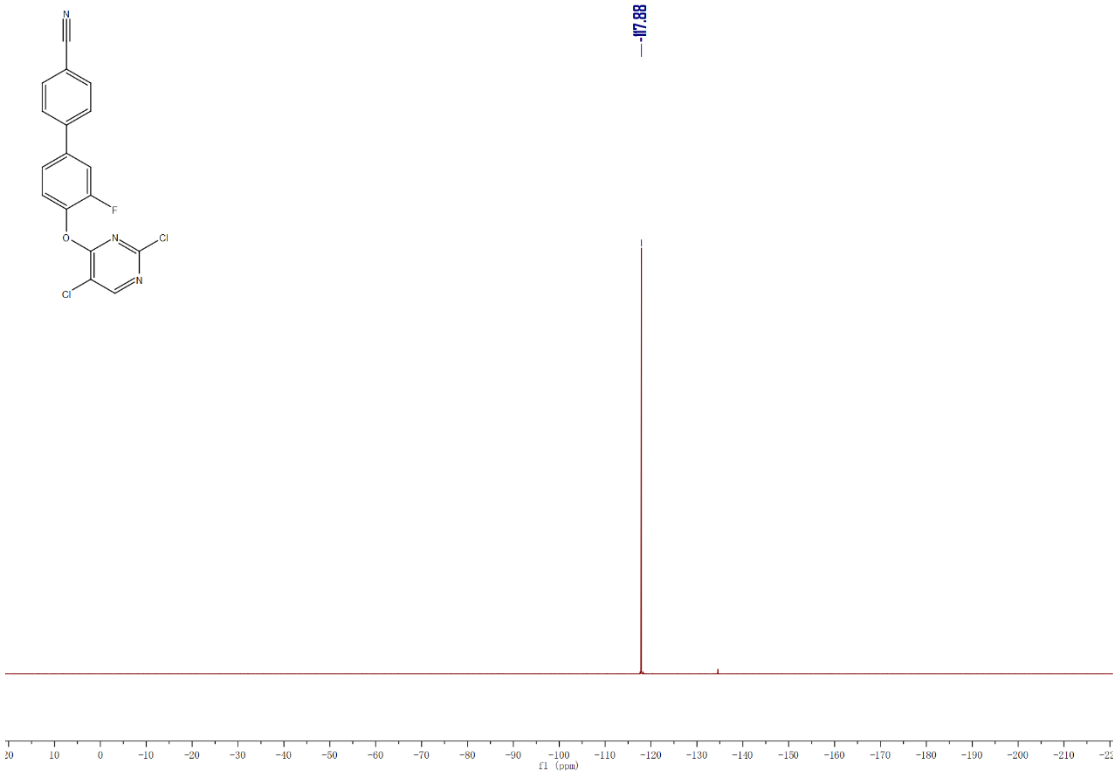

-117.88
f1 (ppm)

HRMS

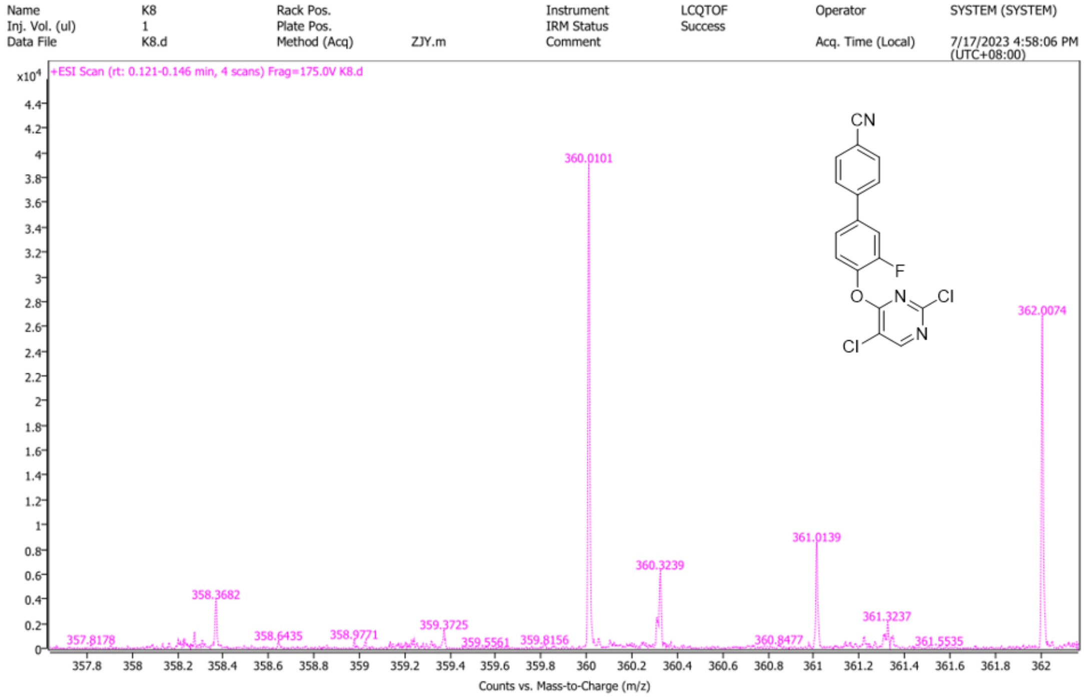

Name K8
Inj. Vol. (ul) 1
Data File K8.d
Rack Pos.
Plate Pos.
Method (Acq) ZJY.m
Instrument LCQTOF
IRM Status Success
Comment
Operator SYSTEM (SYSTEM)
Acq. Time (Local) 7/17/2023 4:58:06 PM (UTC+08:00)
+ESI Scan (rt: 0.121-0.146 min, 4 scans) Frag=175.0V K8.d
360.0101
362.0074
361.0139
360.3239
358.3682
361.3237
Counts vs. Mass-to-Charge (m/z)

^{1}H NMR, ^{13}C NMR and HRMS spectra of 9n

^{1}H NMR

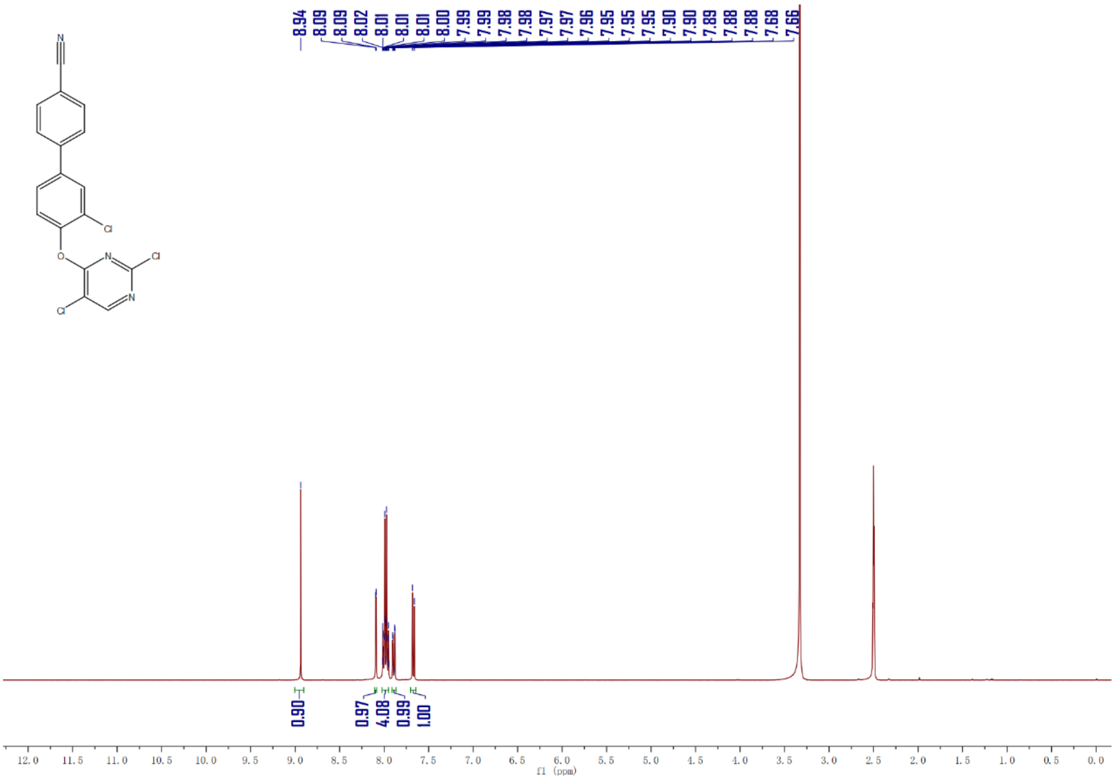

^{13}C NMR

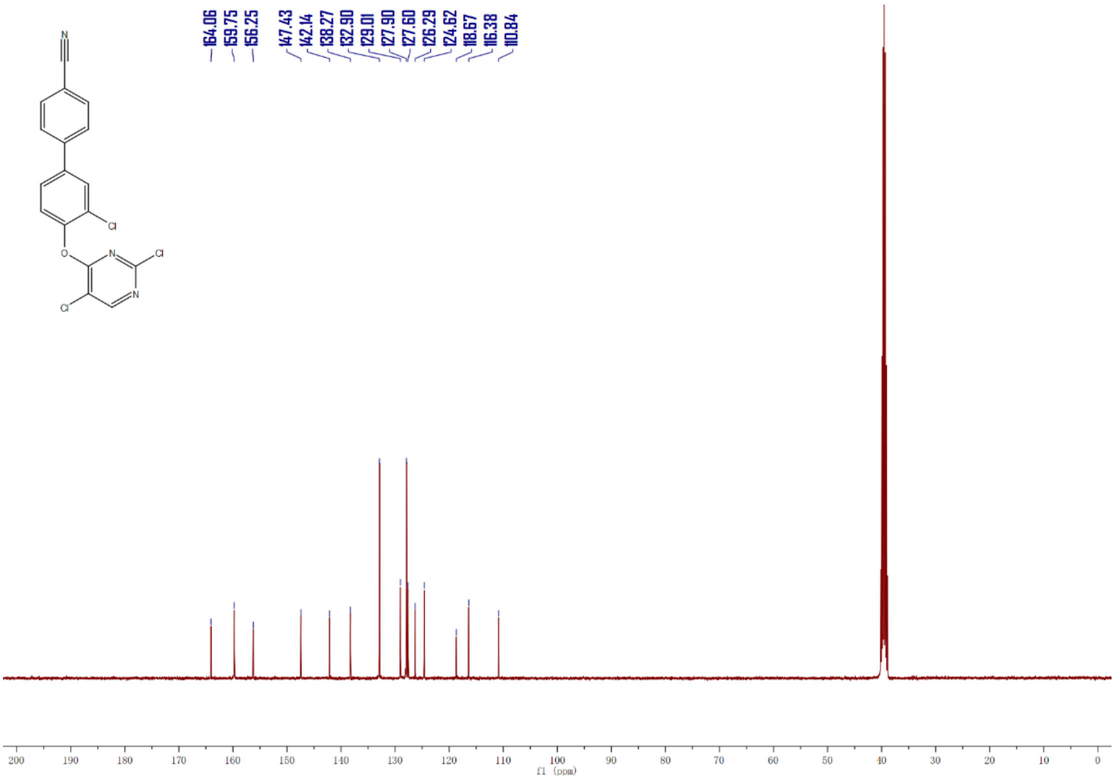

HRMS

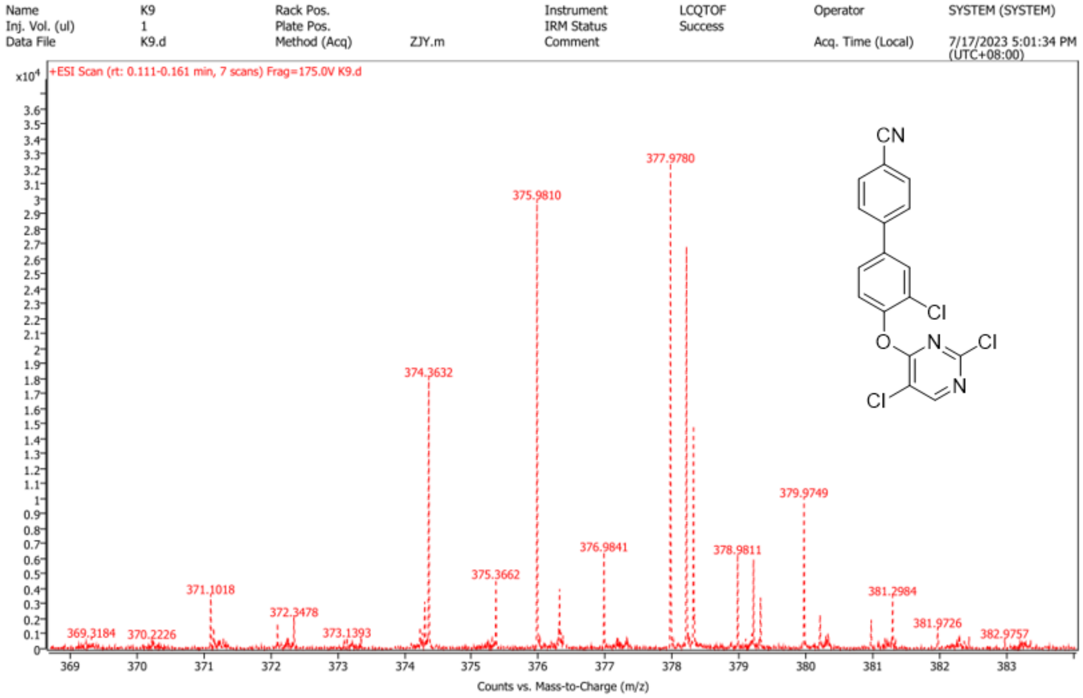

^{1}H NMR, ^{13}C NMR and HRMS spectra of 9o

^{1}H NMR

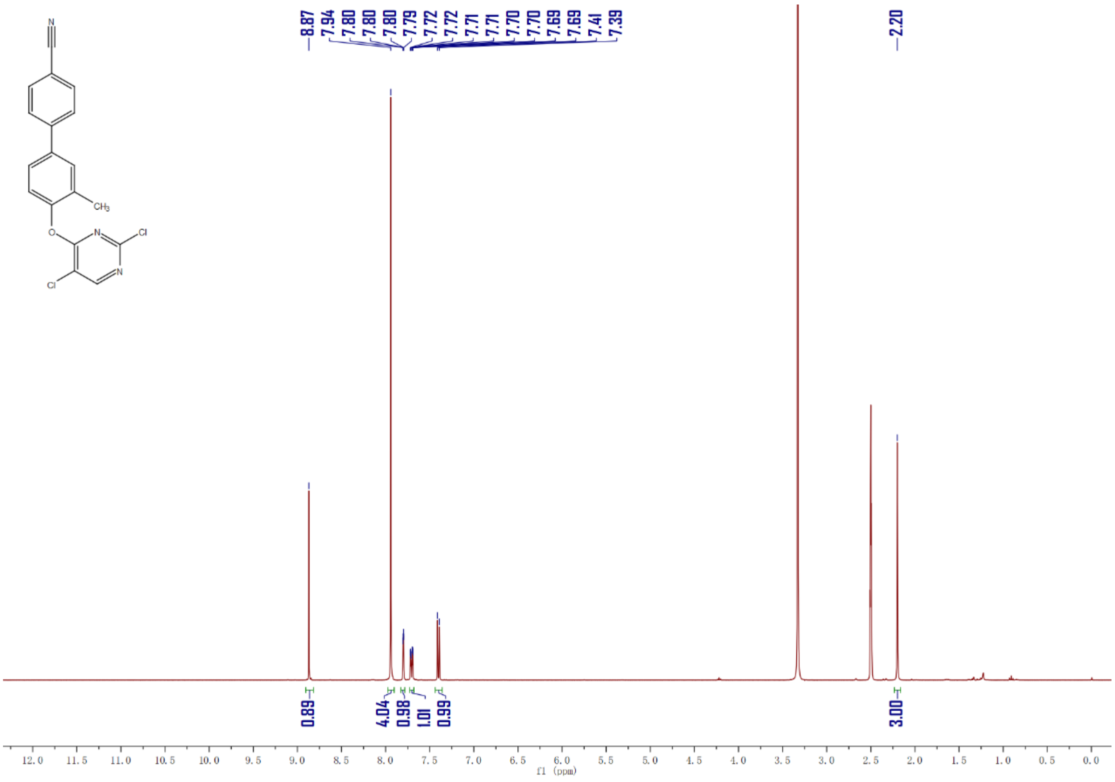

^{13}C NMR

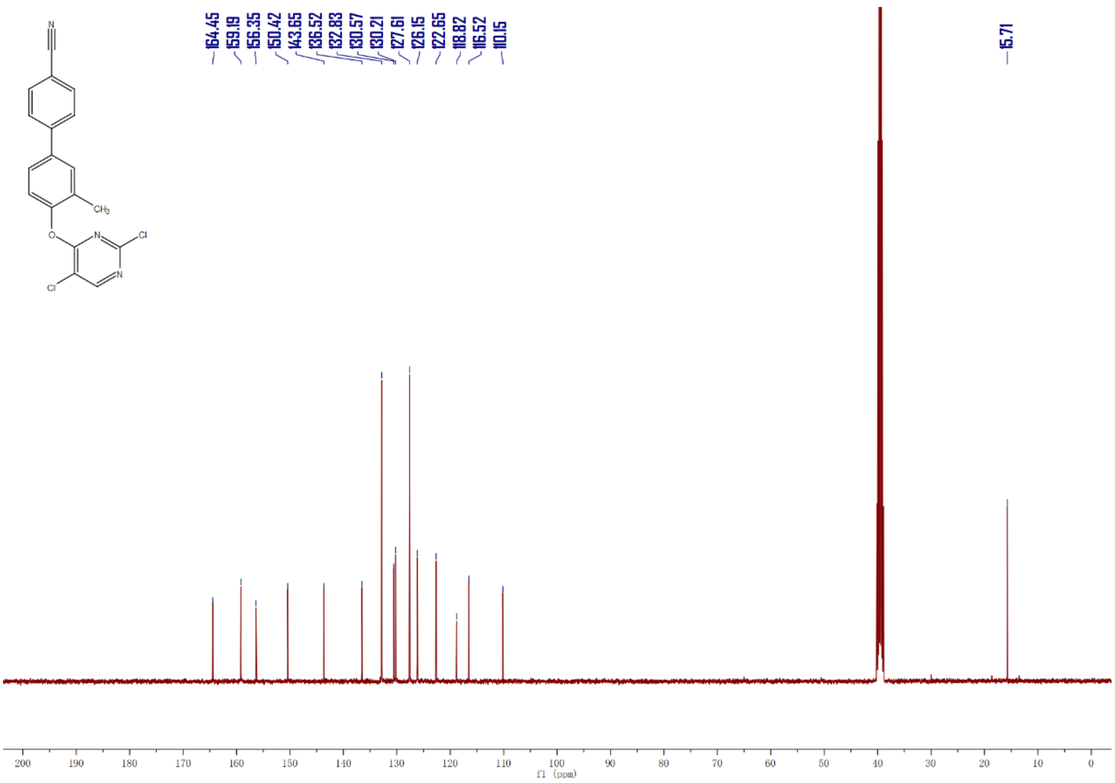

164.45
159.19
156.35
150.42
143.65
136.52
132.83
130.57
130.21
127.61
126.15
122.65
118.82
116.52
110.15
15.71
f1 (ppm)

HRMS

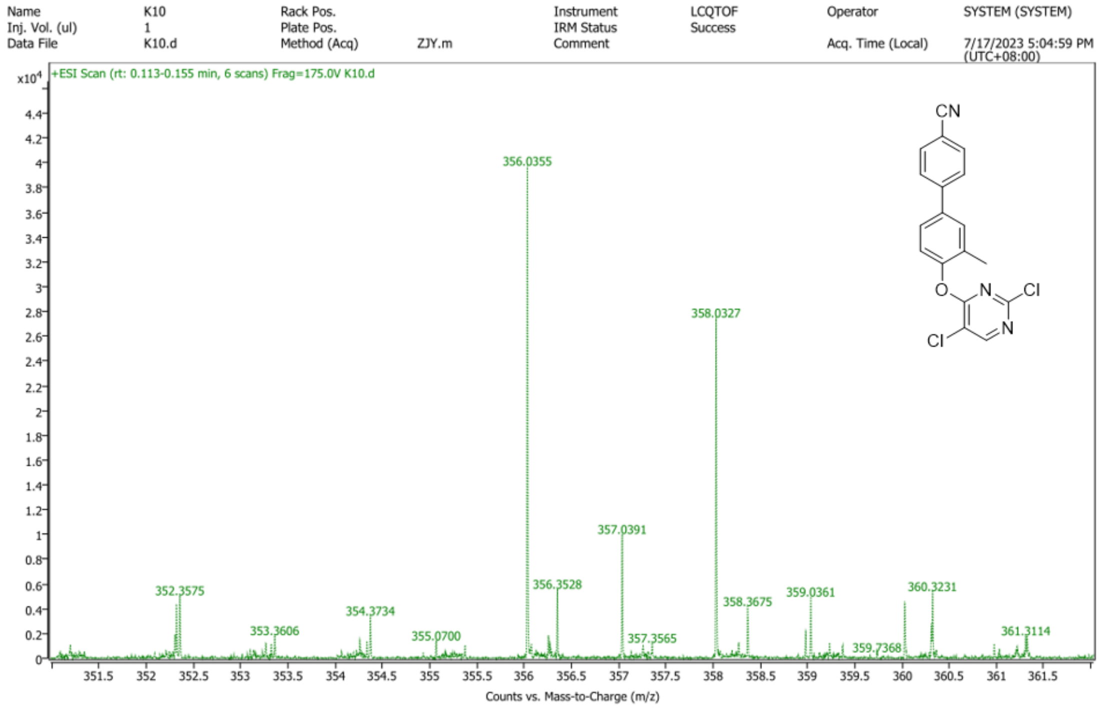

Name K10
Inj. Vol. (ul) 1
Data File K10.d
Rack Pos.
Plate Pos.
Method (Acq) ZJY.m
Instrument LCQTOF
IRM Status Success
Comment
Operator SYSTEM (SYSTEM)
Acq. Time (Local) 7/17/2023 5:04:59 PM (UTC+08:00)
+ESI Scan (rt: 0.113-0.155 min, 6 scans) Frag=175.0V K10.d
356.0355
358.0327
357.0391
352.3575
356.3528
360.3231
359.0361
358.3675
354.3734
353.3606
355.0700
357.3565
359.7368
361.3114
Counts vs. Mass-to-Charge (m/z)

^{1}H NMR, ^{13}C NMR and HRMS spectra of 9p

^{1}H NMR

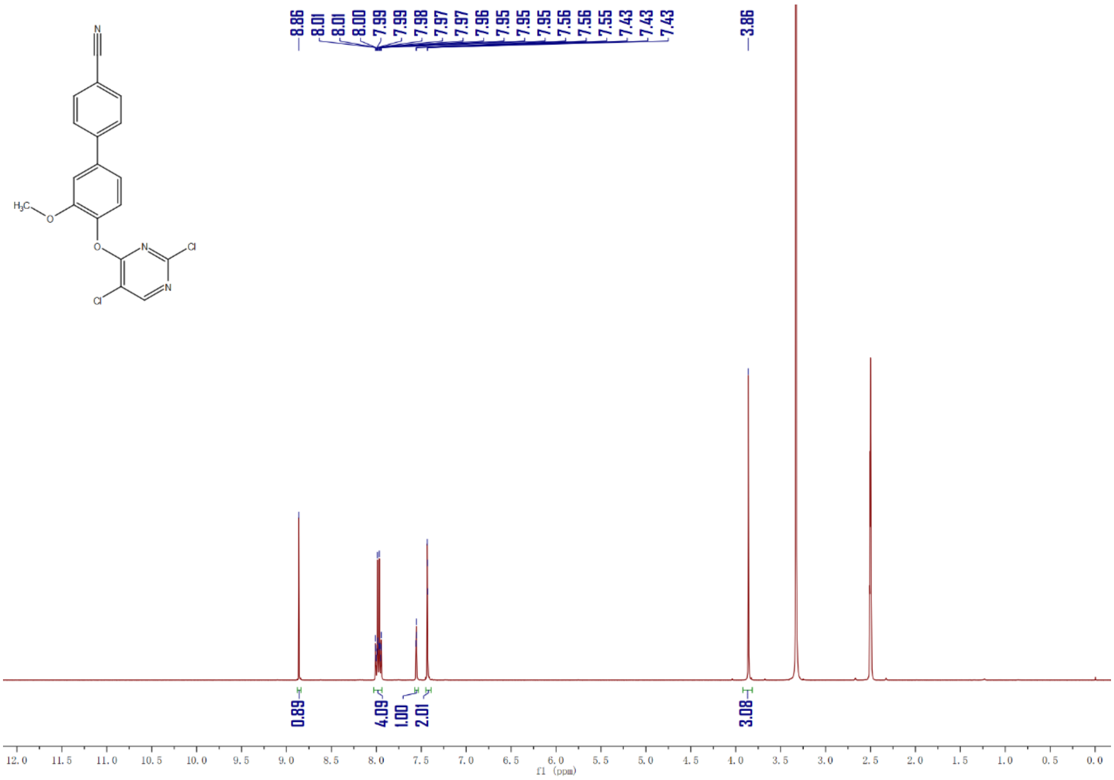

^{13}C NMR

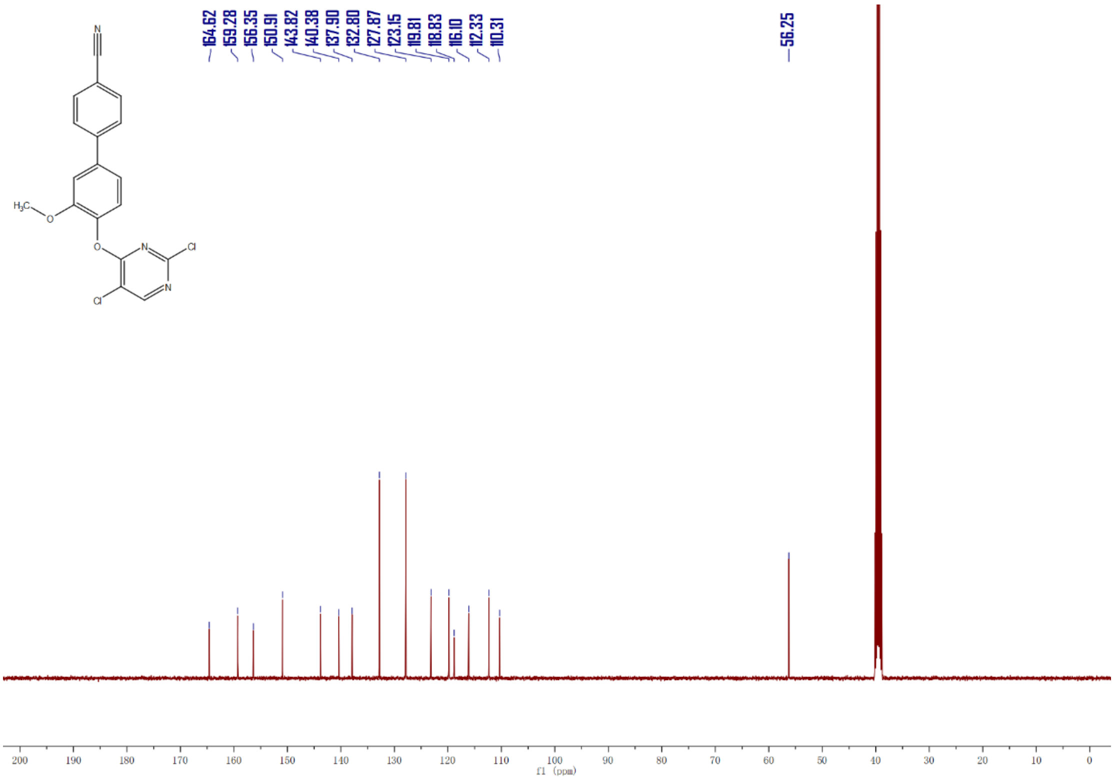

HRMS

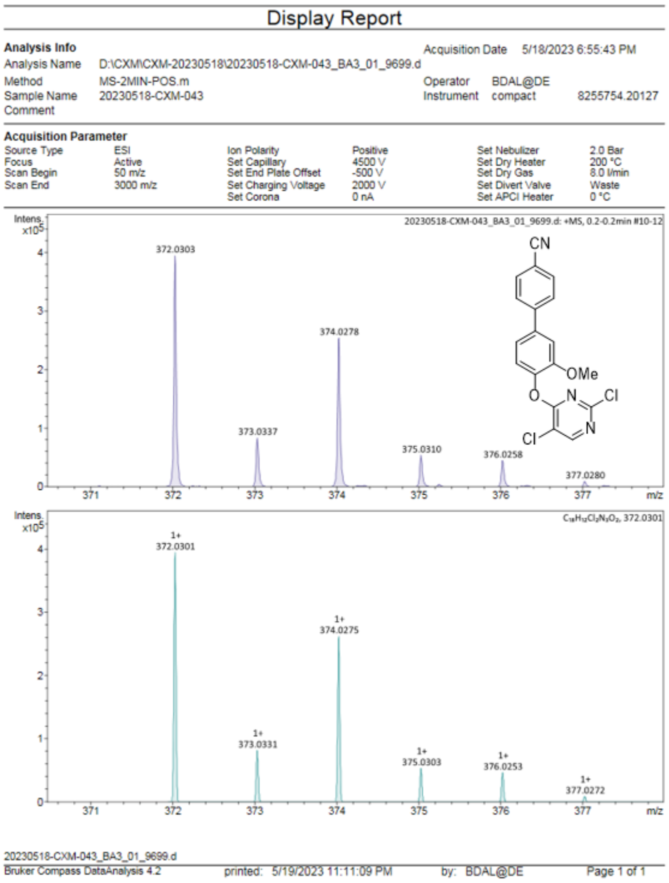

^{1}H NMR, ^{13}C NMR and HRMS spectra of 9q

^{1}H NMR

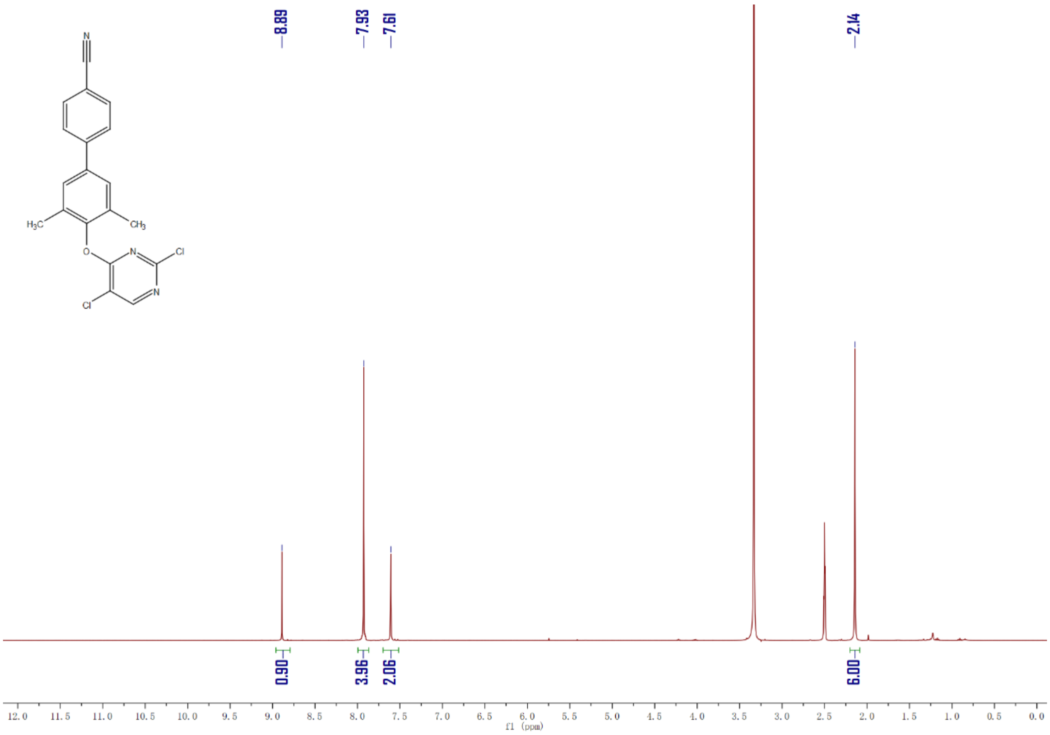

^{13}C NMR

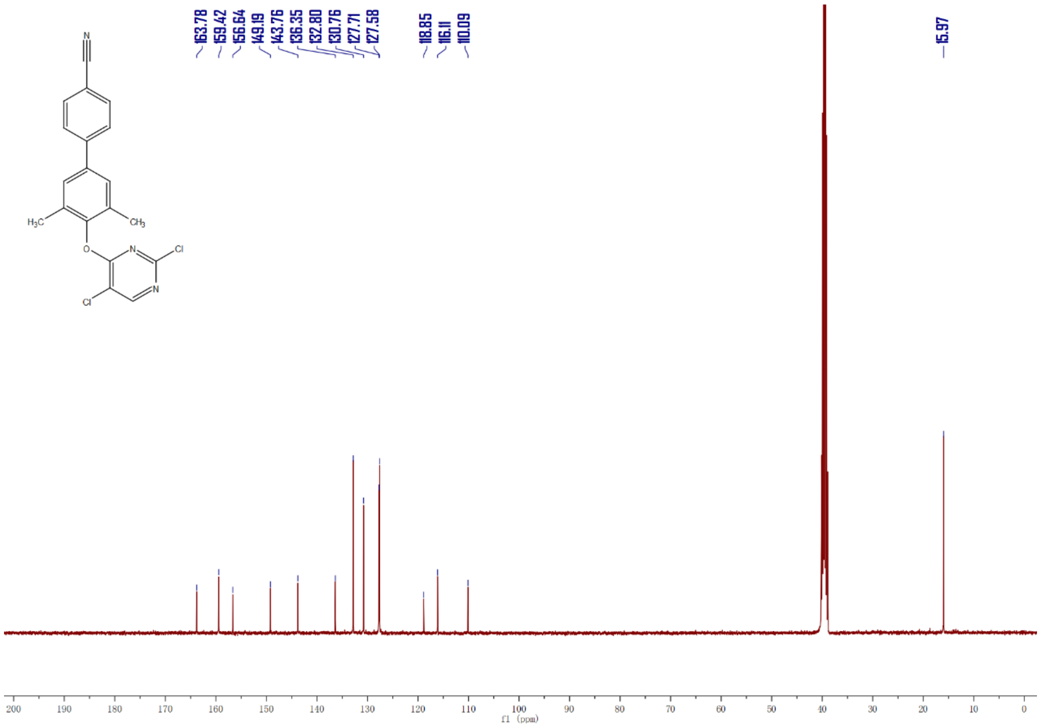
163.78
159.42
156.64
149.19
143.76
136.35
132.80
130.76
127.71
127.58
118.85
116.11
110.09
15.97
f1 (ppm)

HRMS

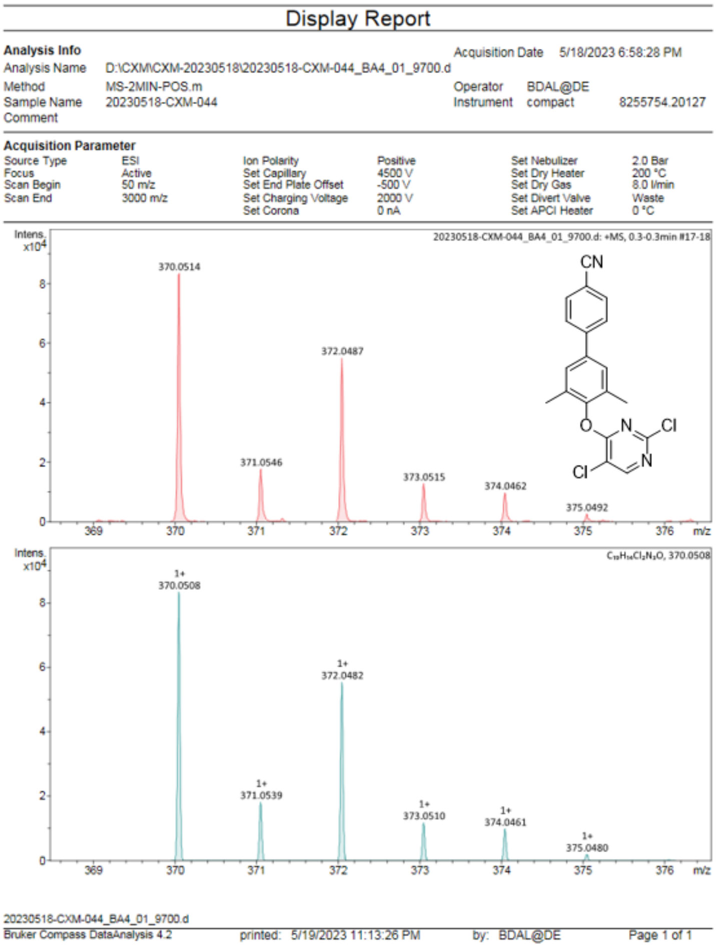
Display Report
Analysis Info
Analysis Name D:\CXM\CXM-20230518\20230518-CXM-044_BA4_01_9700.d
Acquisition Date 5/18/2023 6:58:28 PM
Method MS-2MIN-POS.m
Operator BDAL@DE
Sample Name 20230518-CXM-044
Instrument compact 8255754.20127
Comment
Acquisition Parameter
Source Type ESI
Focus Active
Scan Begin 50 m/z
Scan End 3000 m/z
Ion Polarity Positive
Set Capillary 4500 V
Set End Plate Offset -500 V
Set Charging Voltage 2000 V
Set Corona 0 nA
Set Nebulizer 2.0 Bar
Set Dry Heater 200 °C
Set Dry Gas 8.0 l/min
Set Divert Valve Waste
Set APCI Heater 0 °C
20230518-CXM-044_BA4_01_9700.d: +MS, 0.3-0.3min #17-18
370.0514
371.0546
372.0487
373.0515
374.0462
375.0492
C19H14Cl2N3O, 370.0508
1+ 370.0508
1+ 371.0539
1+ 372.0482
1+ 373.0510
1+ 374.0461
1+ 375.0480
20230518-CXM-044_BA4_01_9700.d
Bruker Compass DataAnalysis 4.2
printed: 5/19/2023 11:13:26 PM
by: BDAL@DE
Page 1 of 1

^{1}H NMR, ^{13}C NMR, ^{13}F NMR and HRMS spectra of 9r

^{1}H NMR

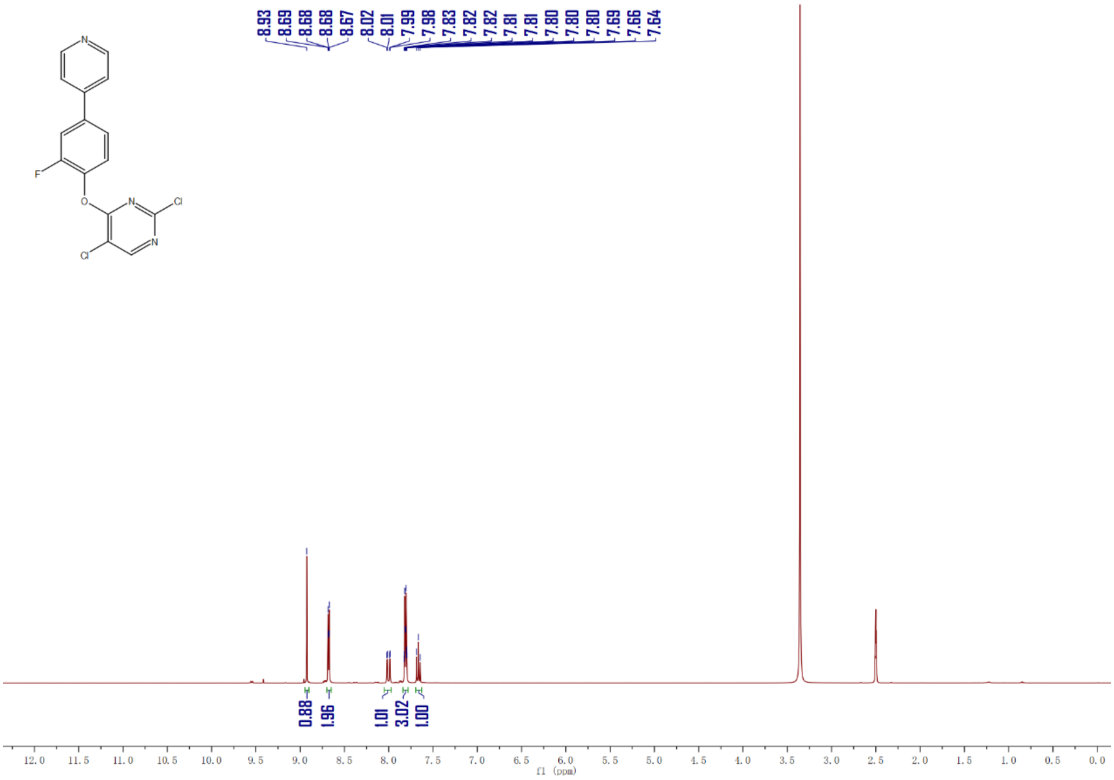

^{13}C NMR

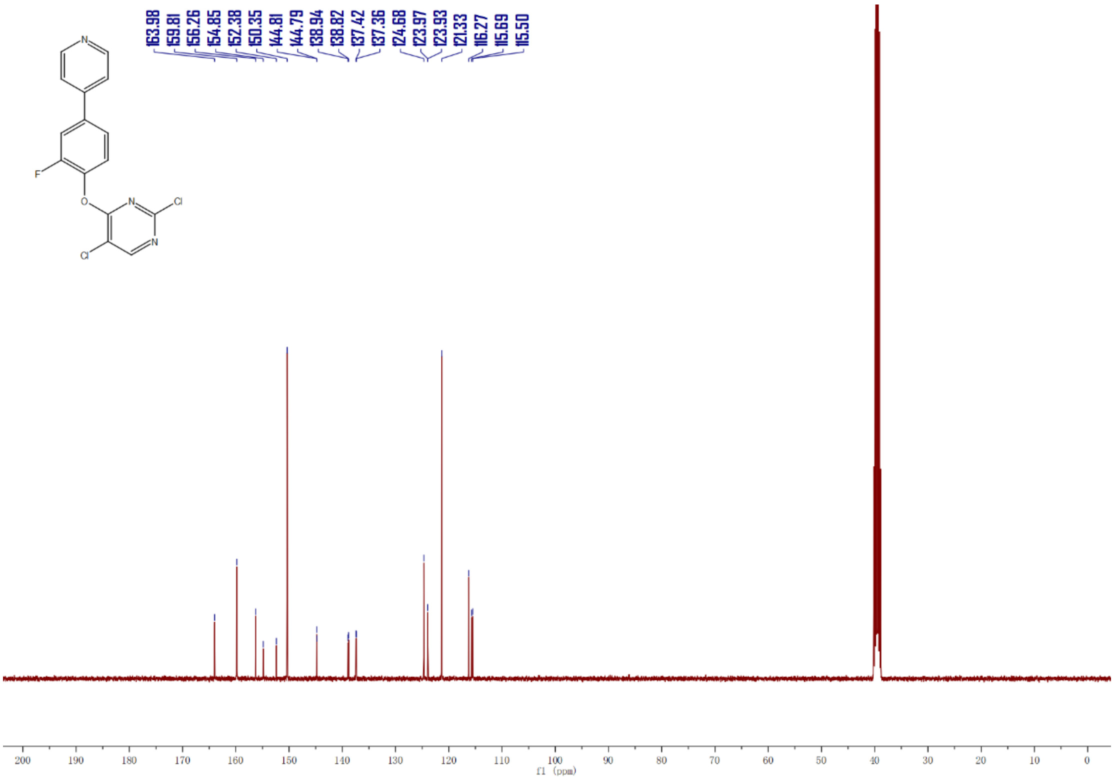

^{13}F NMR

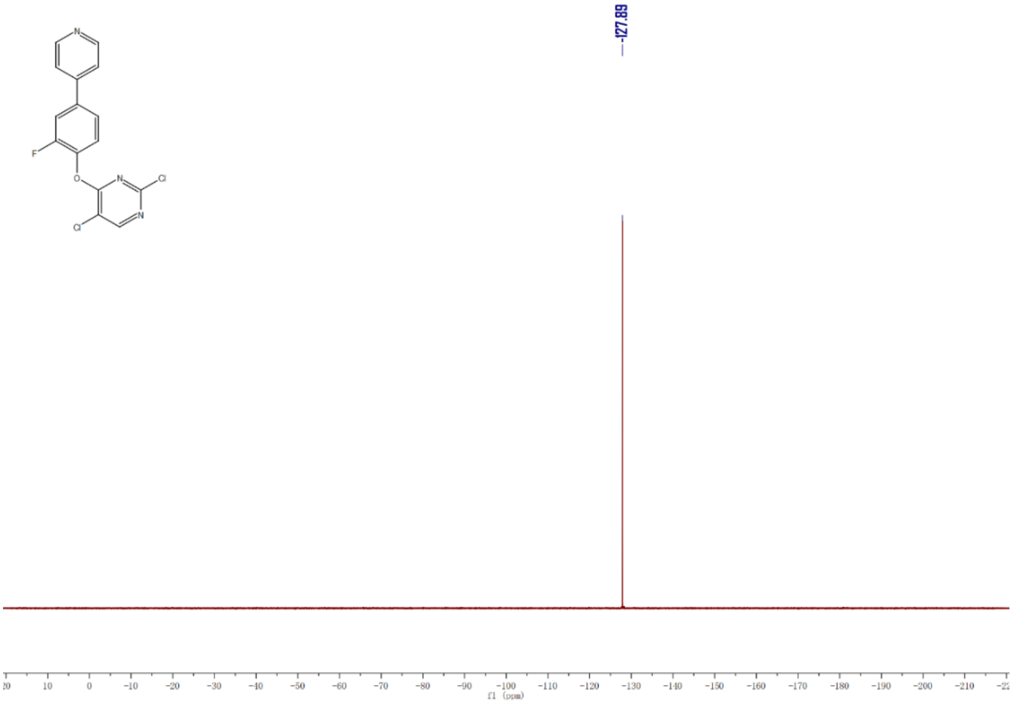

HRMS

Display Report

Analysis Info

Analysis Name	D:\CXM\CXM-20230518\20230518-CXM-051_BB3_01_9707.d	Acquisition Date	5/18/2023 7:17:48 PM
Method	MS-2MIN-POS.m	Operator	BDAL@DE
Sample Name	20230518-CXM-051	Instrument	compact 8255754.20127
Comment			

Acquisition Parameter

Source Type	ESI	Ion Polarity	Positive	Set Nebulizer	2.0 Bar
Focus	Active	Set Capillary	4500 V	Set Dry Heater	200 °C
Scan Begin	50 m/z	Set End Plate Offset	-500 V	Set Dry Gas	8.0 l/min
Scan End	3000 m/z	Set Charging Voltage	2000 V	Set Divert Valve	Waste
		Set Corona	0 nA	Set APCI Heater	0 °C

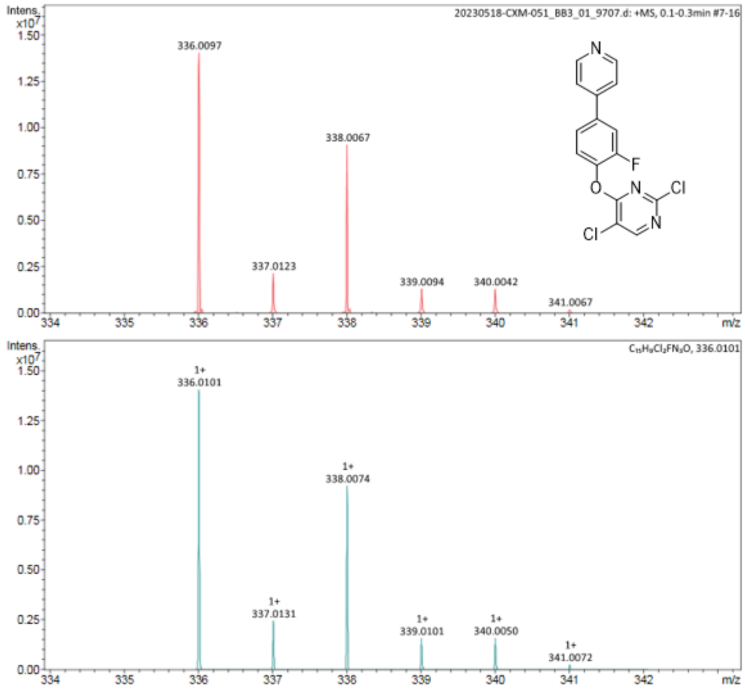

20230518-CXM-051_BB3_01_9707.d
Bruker Compass DataAnalysis 4.2 printed: 5/19/2023 11:39:47 PM by: BDAL@DE Page 1 of 1

^{1}H NMR, ^{13}C NMR and HRMS spectra of 9s

^{1}H NMR

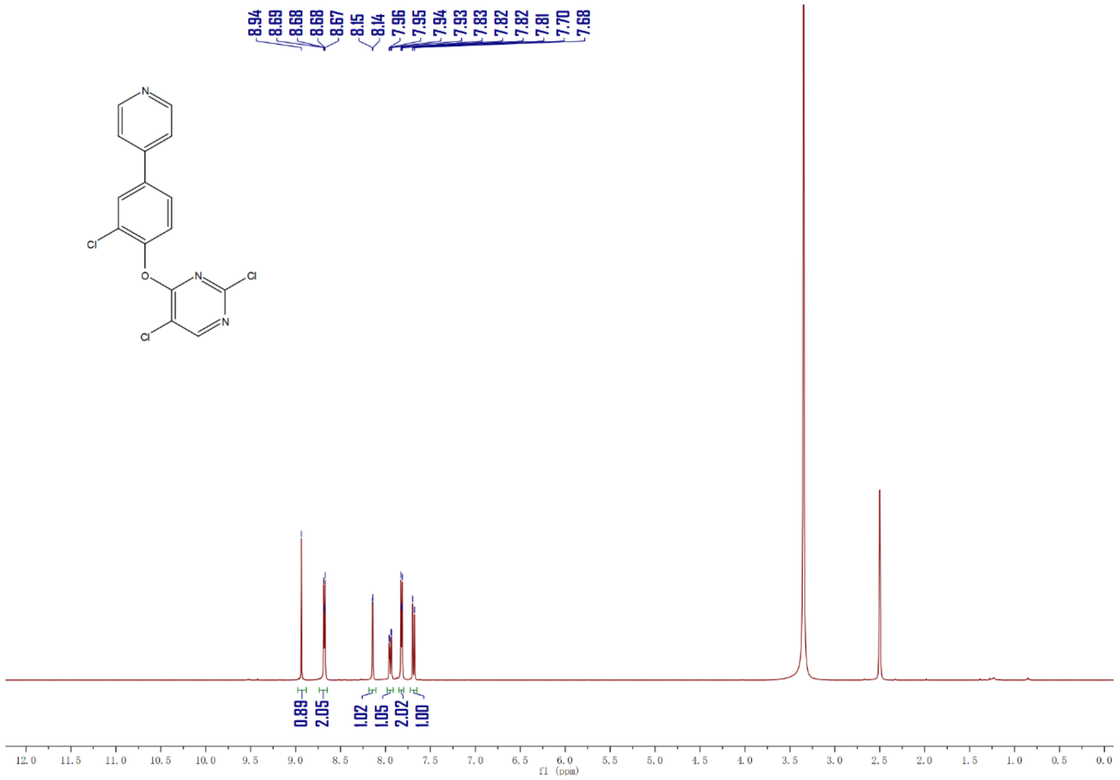

^{13}C NMR

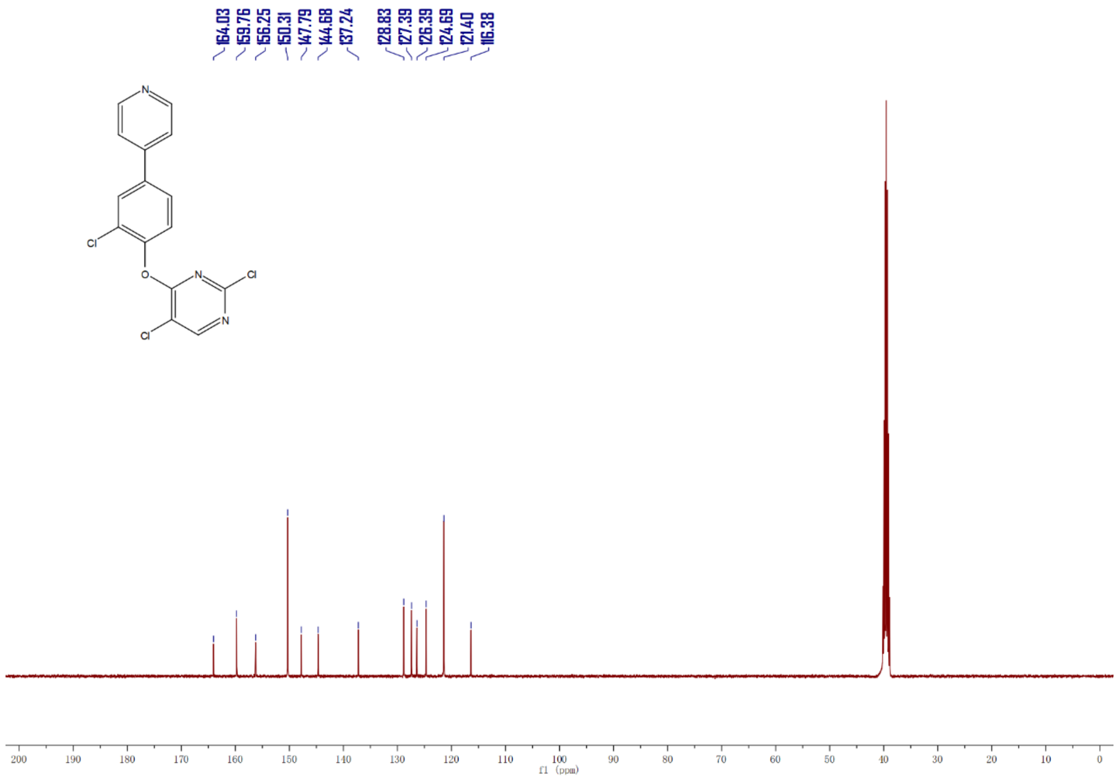

HRMS

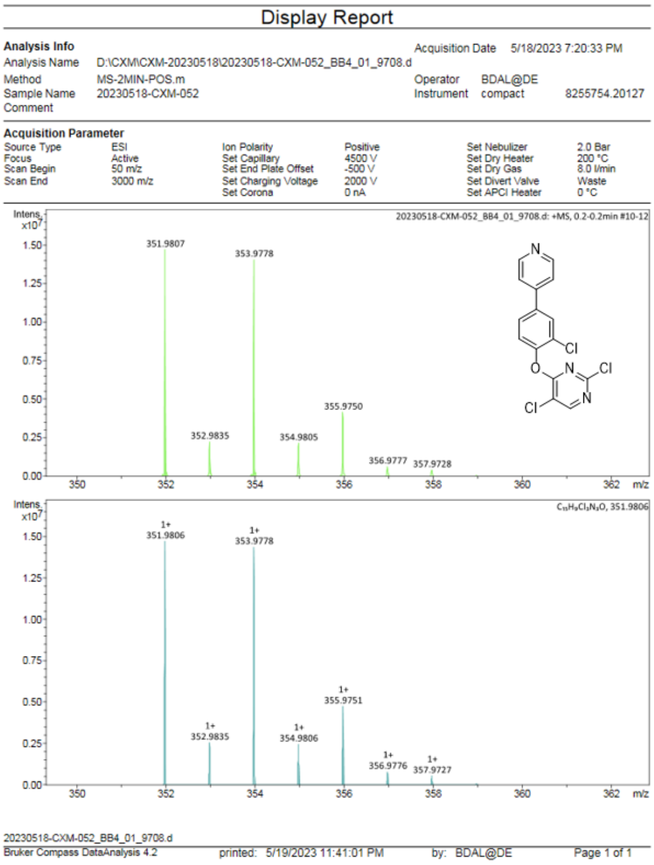

^{1}H NMR, ^{13}C NMR and HRMS spectra of 9t

^{1}H NMR

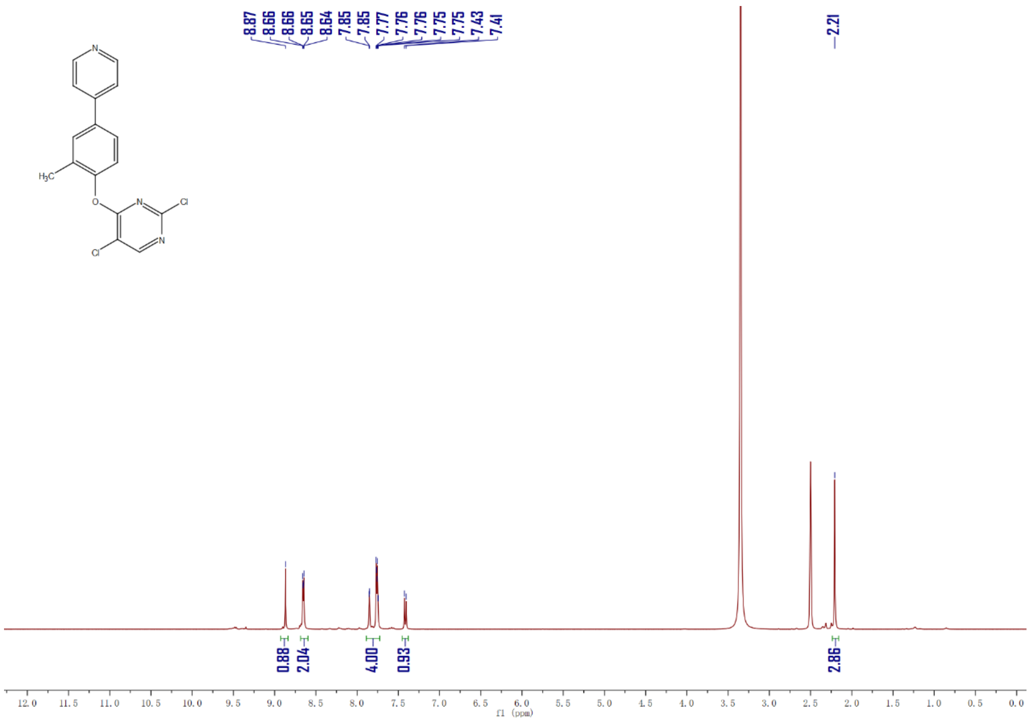

^{13}C NMR

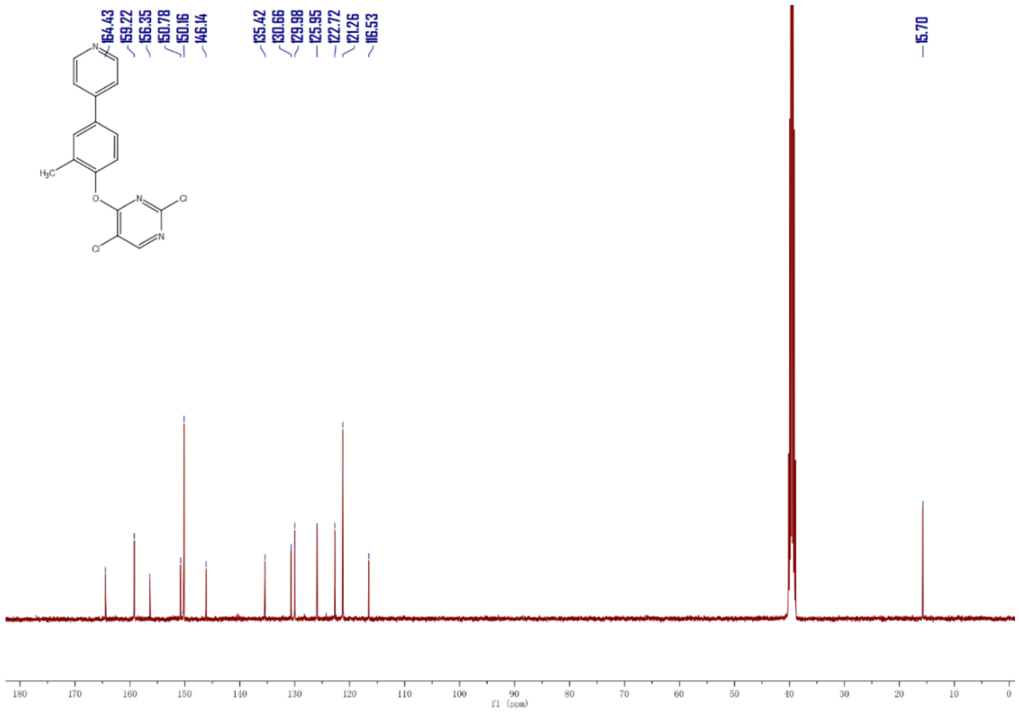

HRMS

Display Report

Analysis Info

Analysis Name	D:\Data\220630\CXM-063_BC7_01_1568.d	Acquisition Date	5/11/2023 8:46:51 PM
Method	LCMS.m	Operator	BDAL@DE
Sample Name	CXM-063	Instrument	micrOTOF-Q III 8228888.20485
Comment			

Acquisition Parameter

Source Type	ESI	Ion Polarity	Positive	Set Nebulizer	2.0 Bar
Focus	Active	Set Capillary	3000 V	Set Dry Heater	200 °C
Scan Begin	50 m/z	Set End Plate Offset	-500 V	Set Dry Gas	8.0 l/min
Scan End	1000 m/z	Set Collision Cell RF	140.0 Vpp	Set Divert Valve	Waste

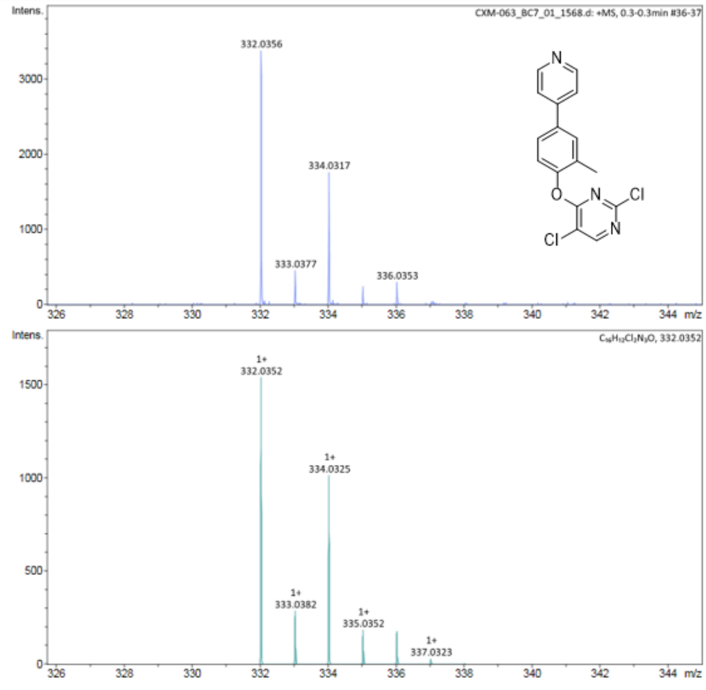

Bruker Compass DataAnalysis 4.2 printed: 5/12/2023 3:34:54 PM by: BDAL@DE Page 1 of 1

^{1}H NMR, ^{13}C NMR and HRMS spectra of 9u

^{1}H NMR

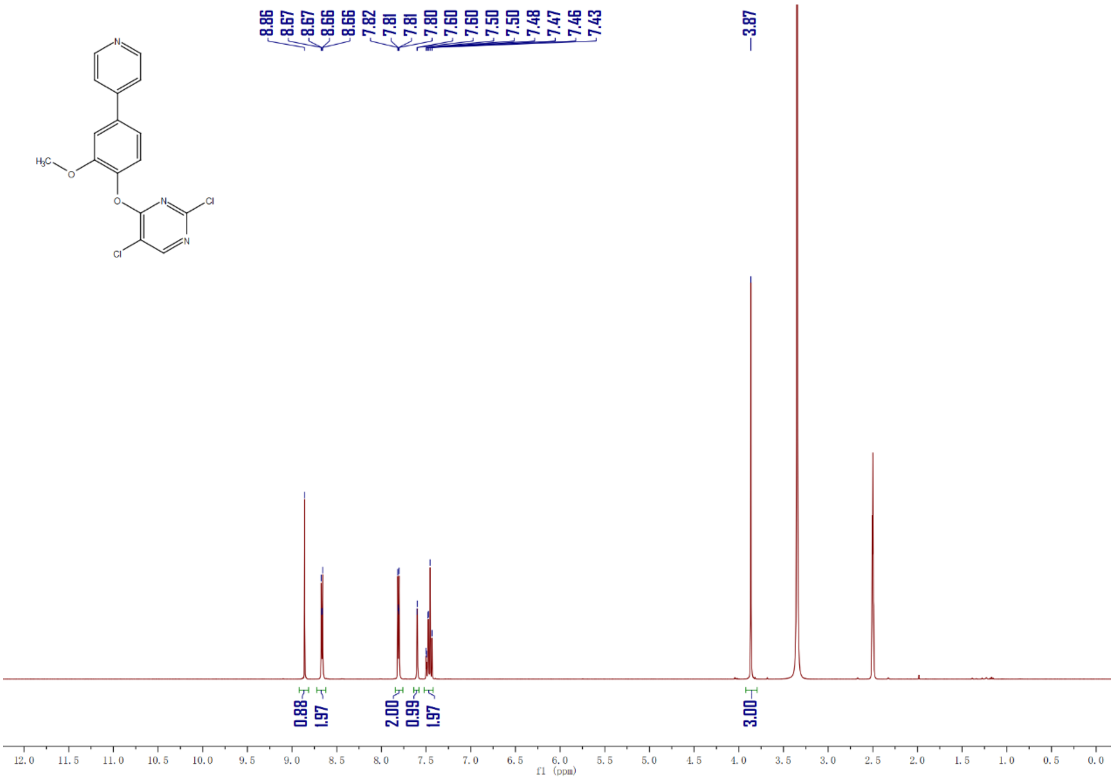

^{13}C NMR

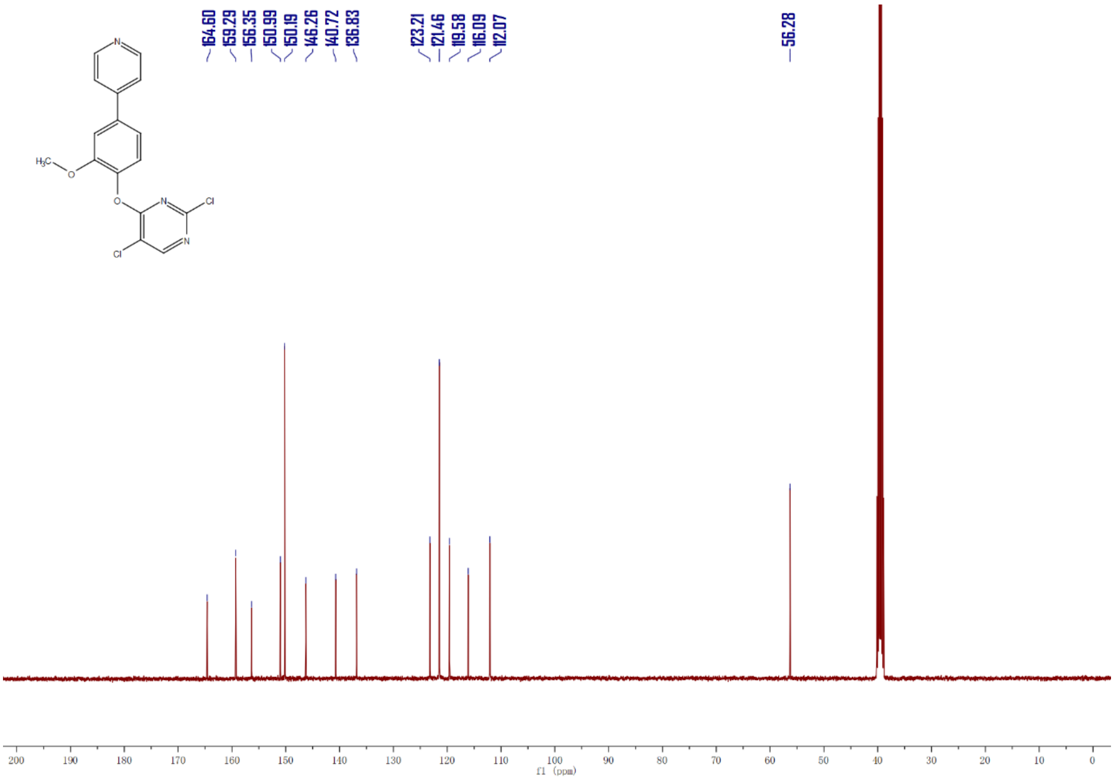

HRMS

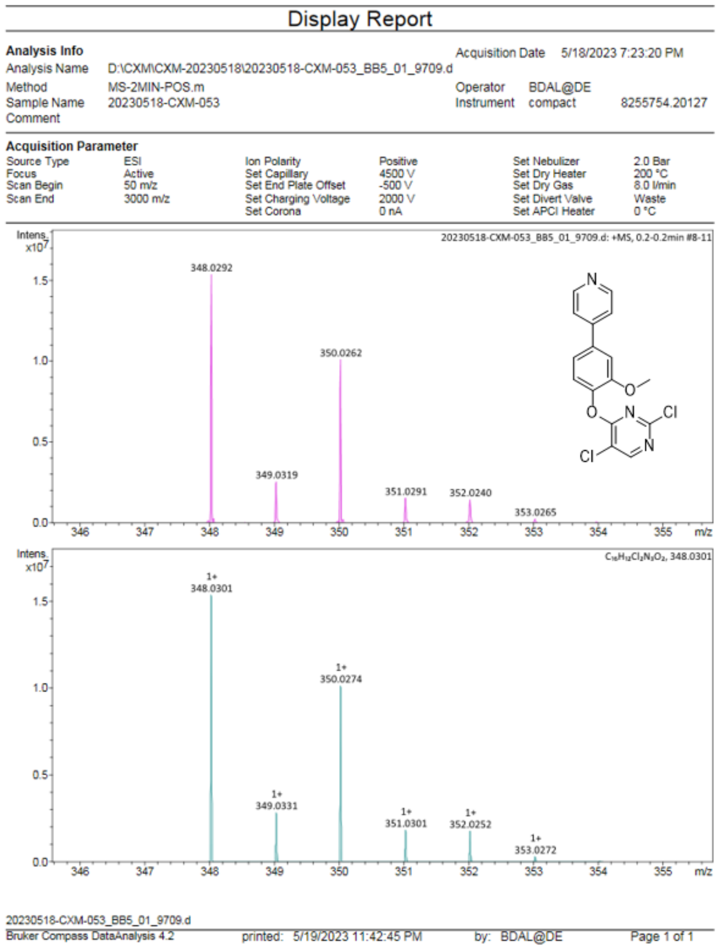

^{1}H NMR, ^{13}C NMR and HRMS spectra of 9v

^{1}H NMR

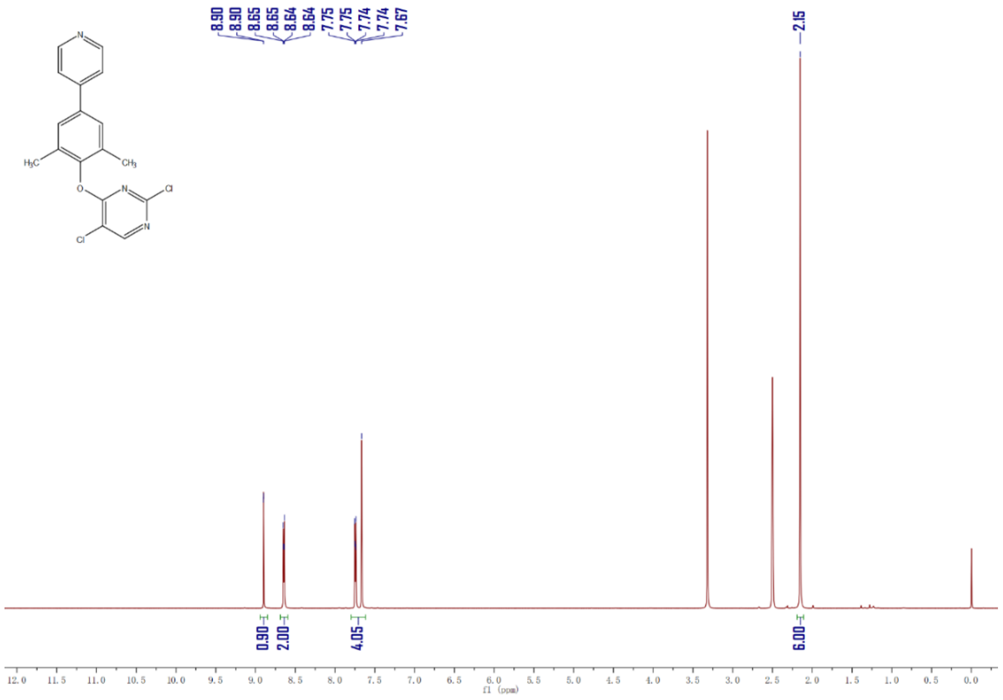

^{13}C NMR

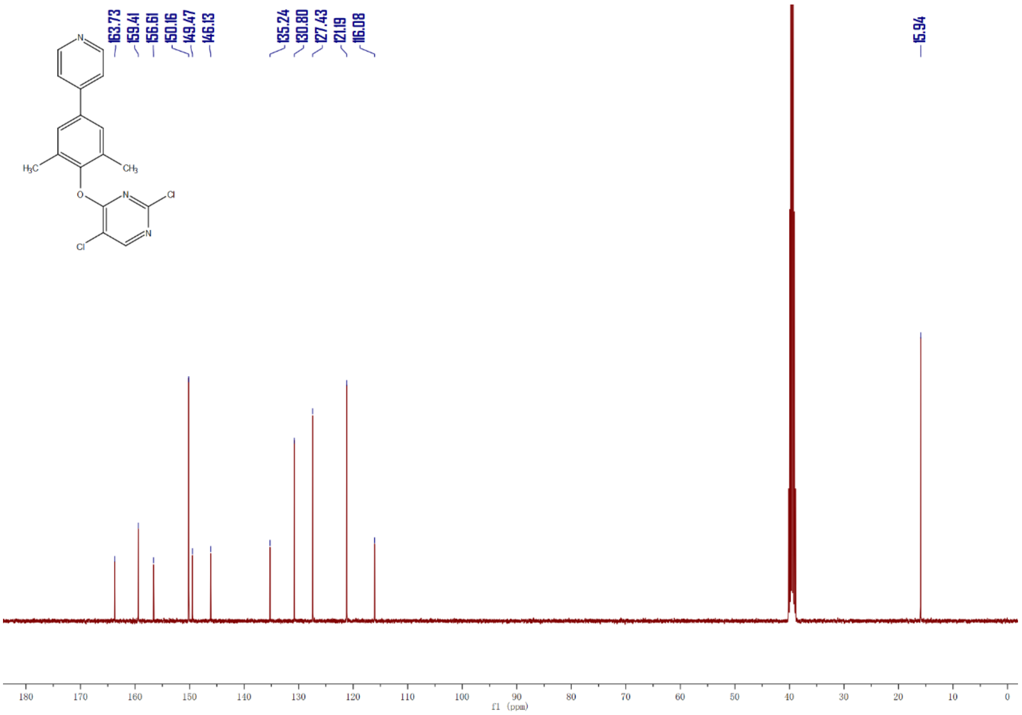

163.73
159.41
156.61
150.16
149.47
146.13
135.24
130.80
127.43
121.19
116.08
15.94
f1 (ppm)

HRMS

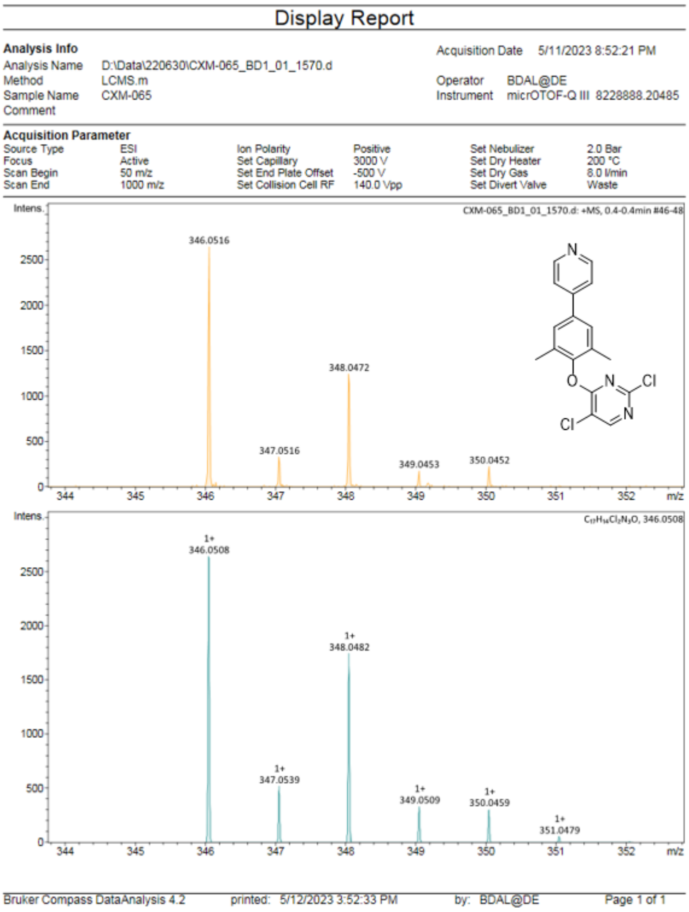

Display Report
Analysis Info
Analysis Name D:\Data\220630\CXM-065_BD1_01_1570.d
Method LCMS.m
Sample Name CXM-065
Comment
Acquisition Date 5/11/2023 8:52:21 PM
Operator BDAL@DE
Instrument micrOTOF-Q III 8228888.20485
Acquisition Parameter
Source Type ESI
Focus Active
Scan Begin 50 m/z
Scan End 1000 m/z
Ion Polarity Positive
Set Capillary 3000 V
Set End Plate Offset -500 V
Set Collision Cell RF 140.0 Vpp
Set Nebulizer 2.0 Bar
Set Dry Heater 200 °C
Set Dry Gas 8.0 l/min
Set Divert Valve Waste
CXM-065_BD1_01_1570.d: +MS, 0.4-0.4min #46-48
346.0516
348.0472
347.0516
349.0453
350.0452
346.0508
348.0482
347.0539
349.0509
350.0459
351.0479
Intens.
m/z
Bruker Compass DataAnalysis 4.2
printed: 5/12/2023 3:52:33 PM
by: BDAL@DE
Page 1 of 1

2.1.2. ^{1}H NMR, ^{13}C NMR, ^{19}F NMR, HRMS spectra of 10a-10x

^{1}H NMR, ^{13}C NMR, ^{19}F NMR, HRMS spectra of 10a

^{1}H NMR

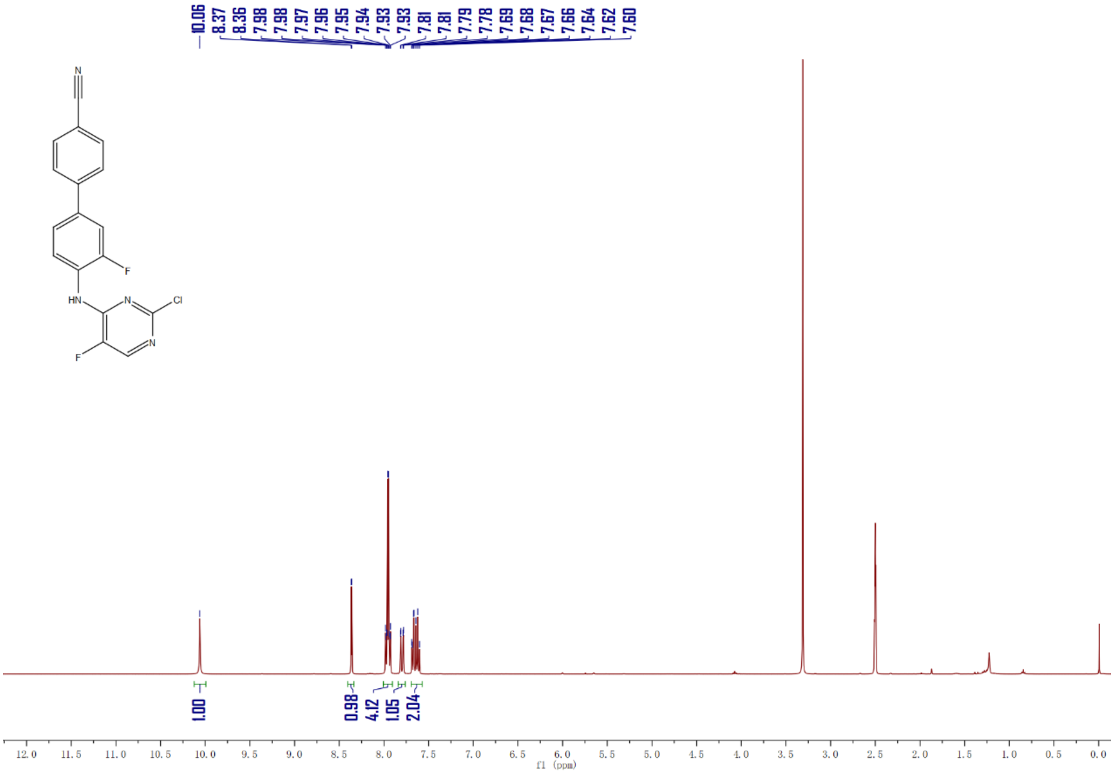

^{13}C NMR

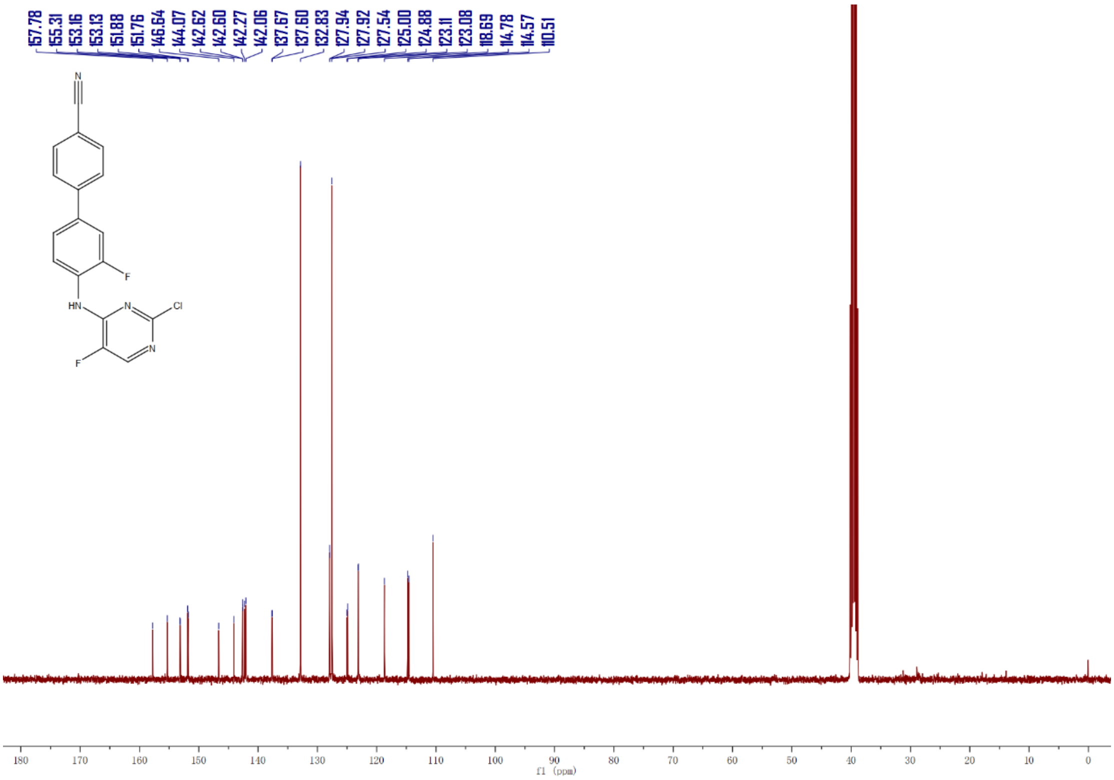

^{19}F NMR

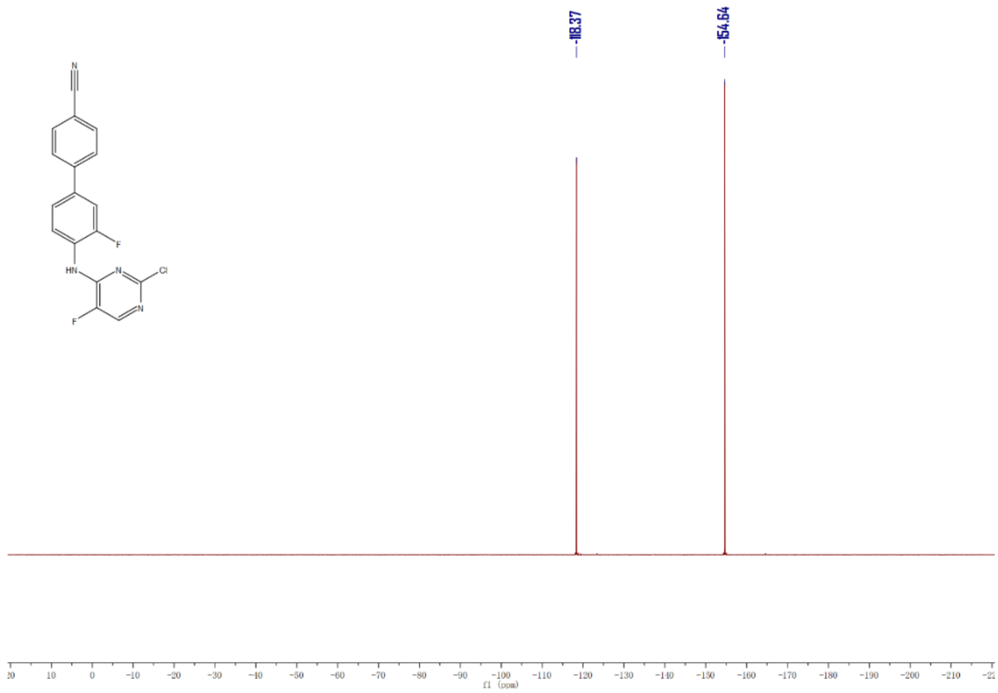

HRMS

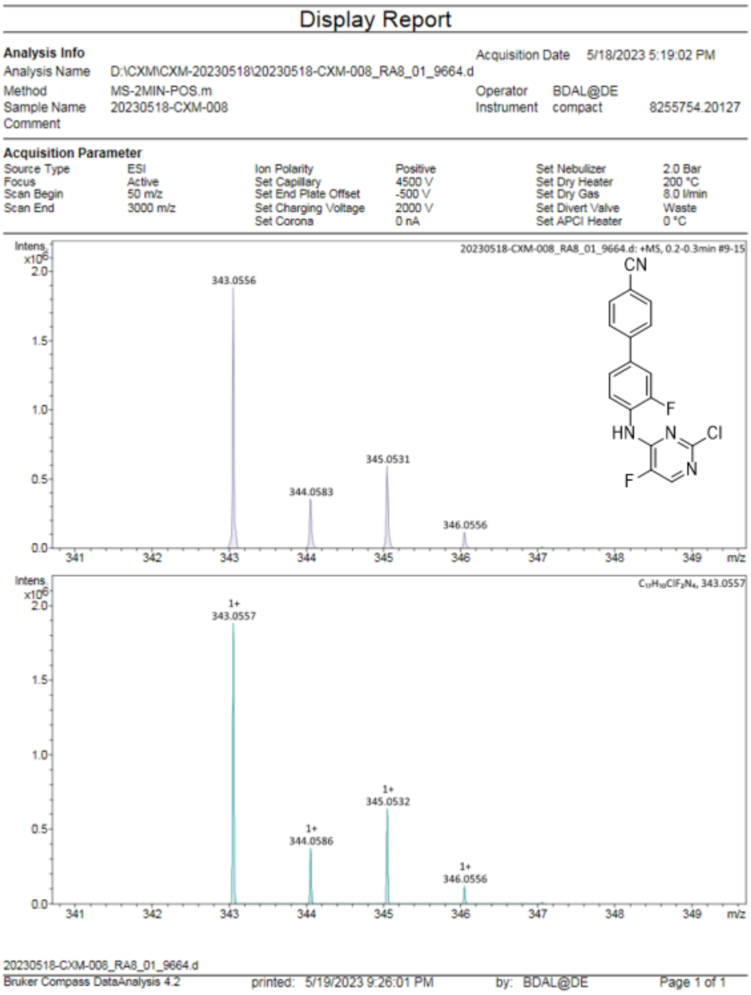

Display Report

Analysis Info

Analysis Name D:\CXM\CXM-20230518\20230518-CXM-008_RA8_01_9664.d
Method MS-2MIN-POS.m
Sample Name 20230518-CXM-008
Comment

Acquisition Date 5/18/2023 5:19:02 PM
Operator BDAL@DE
Instrument compact 8255754.20127

Acquisition Parameter

Source Type	ESI	Ion Polarity	Positive	Set Nebulizer	2.0 Bar
Focus	Active	Set Capillary	4500 V	Set Dry Heater	200 °C
Scan Begin	50 m/z	Set End Plate Offset	-500 V	Set Dry Gas	8.0 l/min
Scan End	3000 m/z	Set Charging Voltage	2000 V	Set Divert Valve	Waste
		Set Corona	0 nA	Set APCI Heater	0 °C

20230518-CXM-008_RA8_01_9664.d
Bruker Compass DataAnalysis 4.2 printed: 5/19/2023 9:26:01 PM by: BDAL@DE Page 1 of 1

^{1}H NMR, ^{13}C NMR, ^{19}F NMR, HRMS spectra of 10b

^{1}H NMR

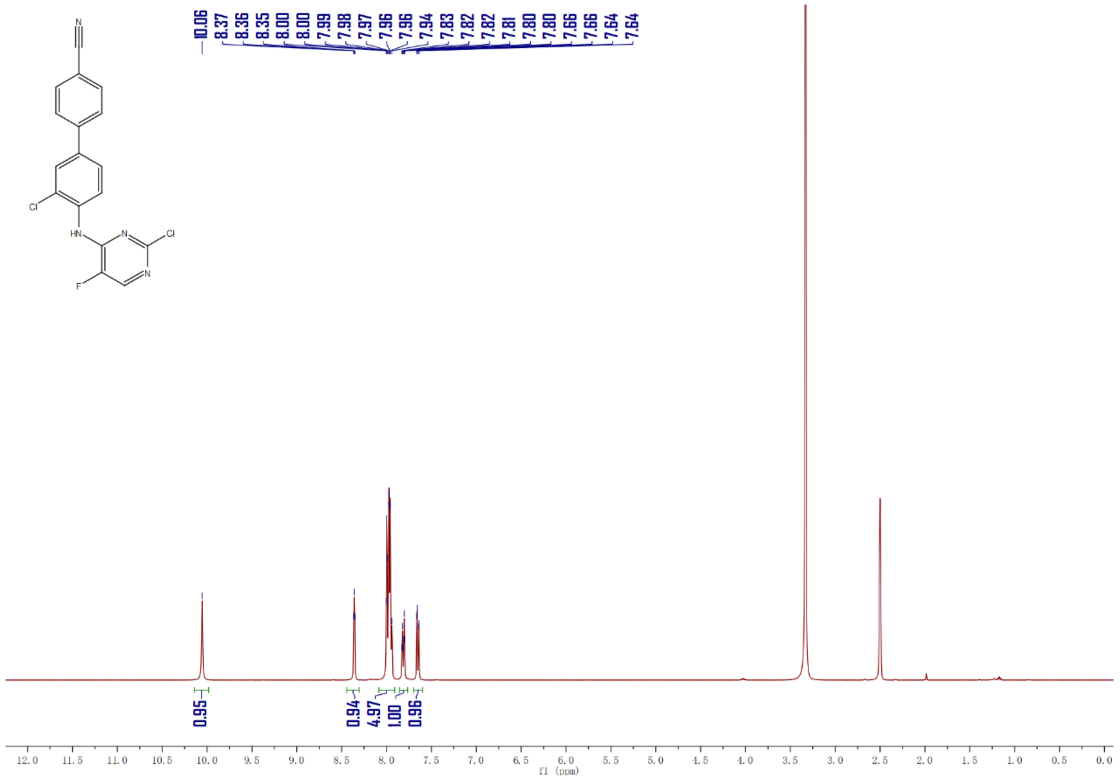

^{13}C NMR

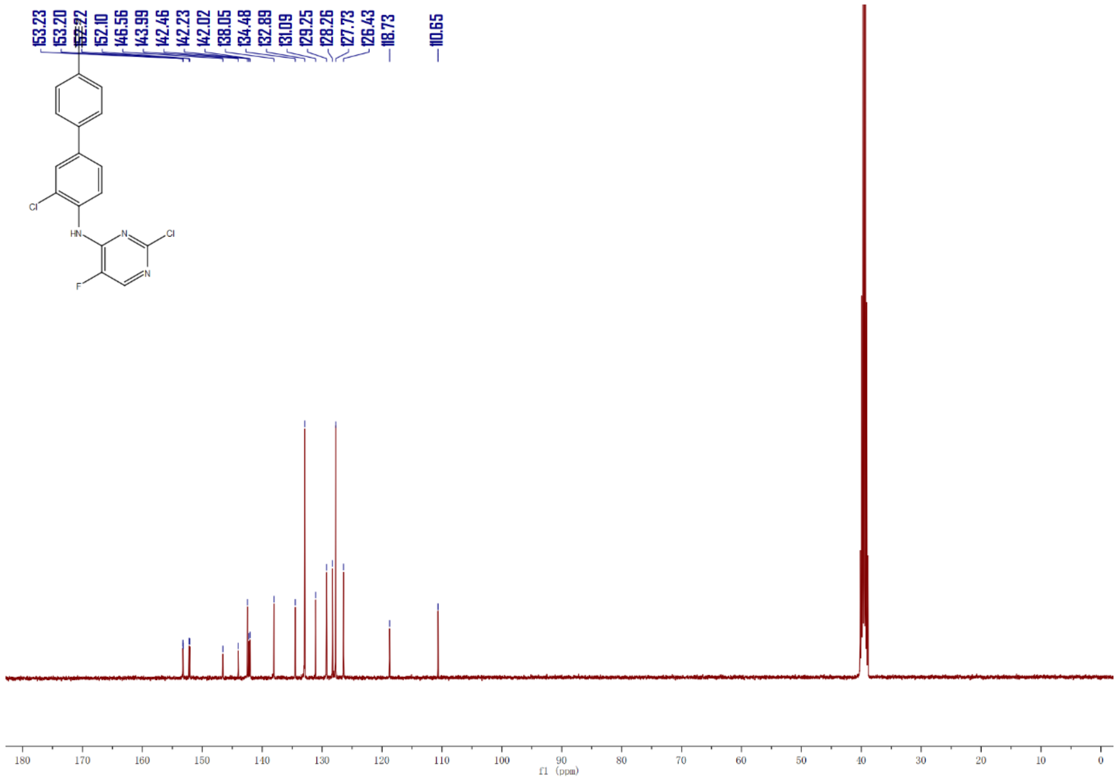

^{19}F NMR

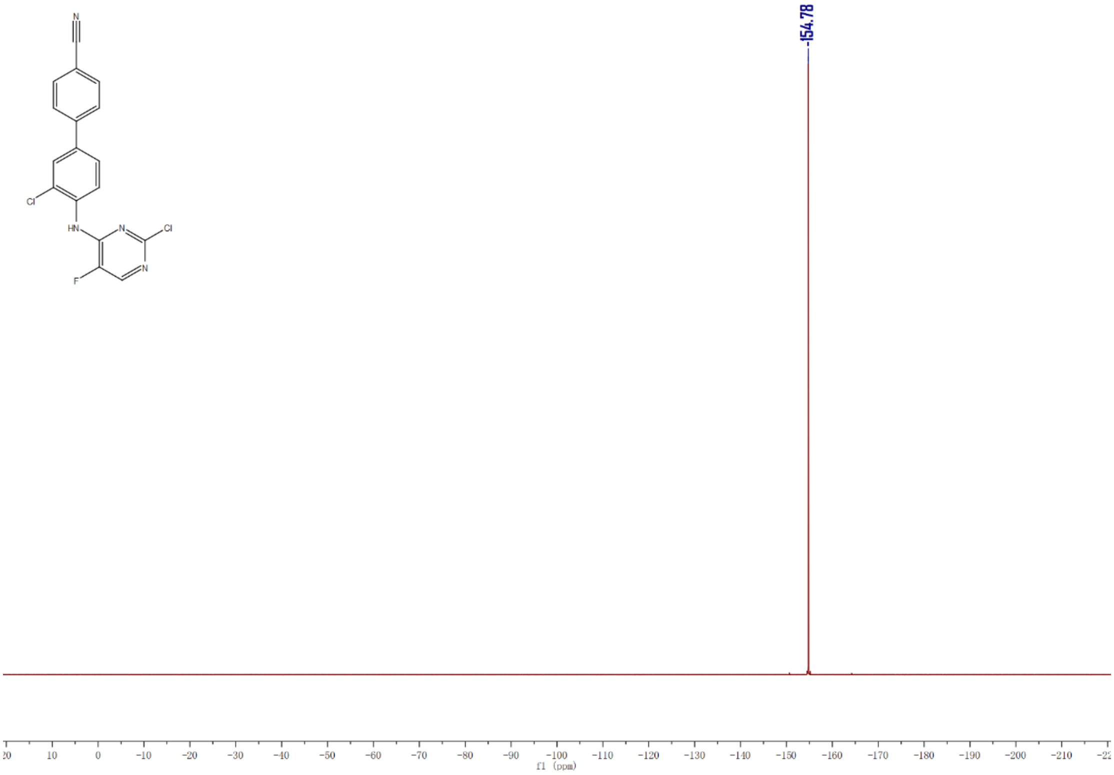

-154.78
f1 (ppm)

HRMS

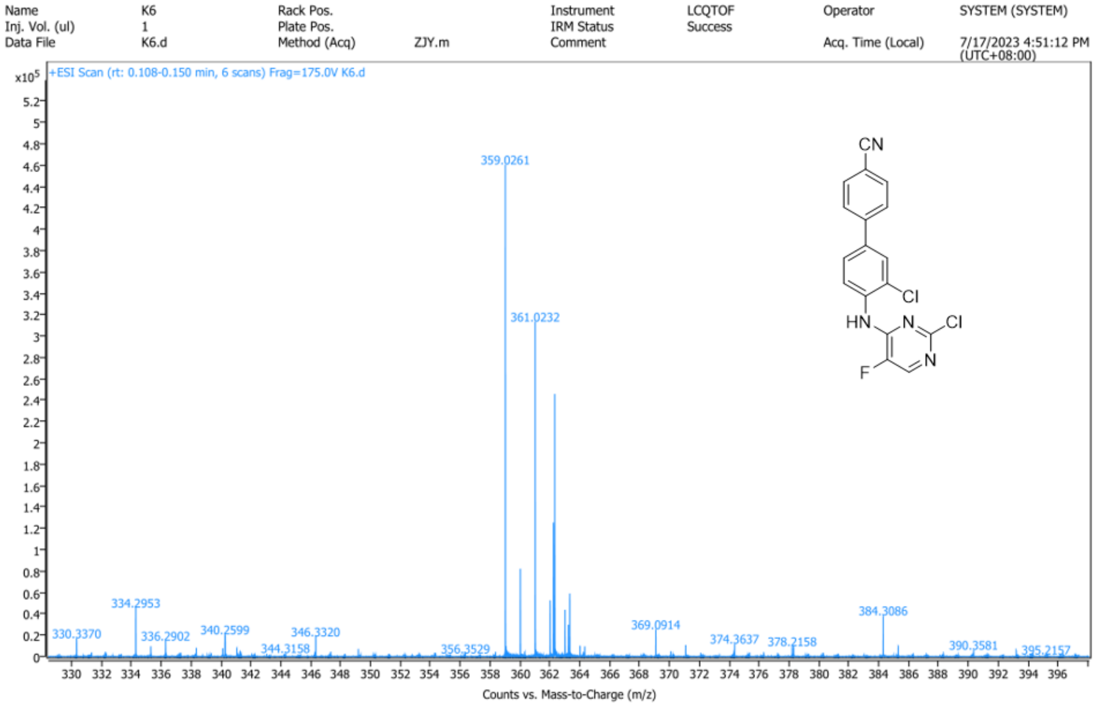

Name K6
Inj. Vol. (ul) 1
Data File K6.d
Rack Pos.
Plate Pos.
Method (Acq) ZJY.m
Instrument LCQTOF
IRM Status Success
Comment
Operator SYSTEM (SYSTEM)
Acq. Time (Local) 7/17/2023 4:51:12 PM (UTC+08:00)
+ESI Scan (rt: 0.108-0.150 min, 6 scans) Frag=175.0V K6.d
359.0261
361.0232
334.2953
330.3370
336.2902
340.2599
344.3158
346.3320
356.3529
369.0914
374.3637
378.2158
384.3086
390.3581
395.2157
Counts vs. Mass-to-Charge (m/z)

^{1}H NMR, ^{13}C NMR, ^{19}F NMR, HRMS spectra of 10c

^{1}H NMR

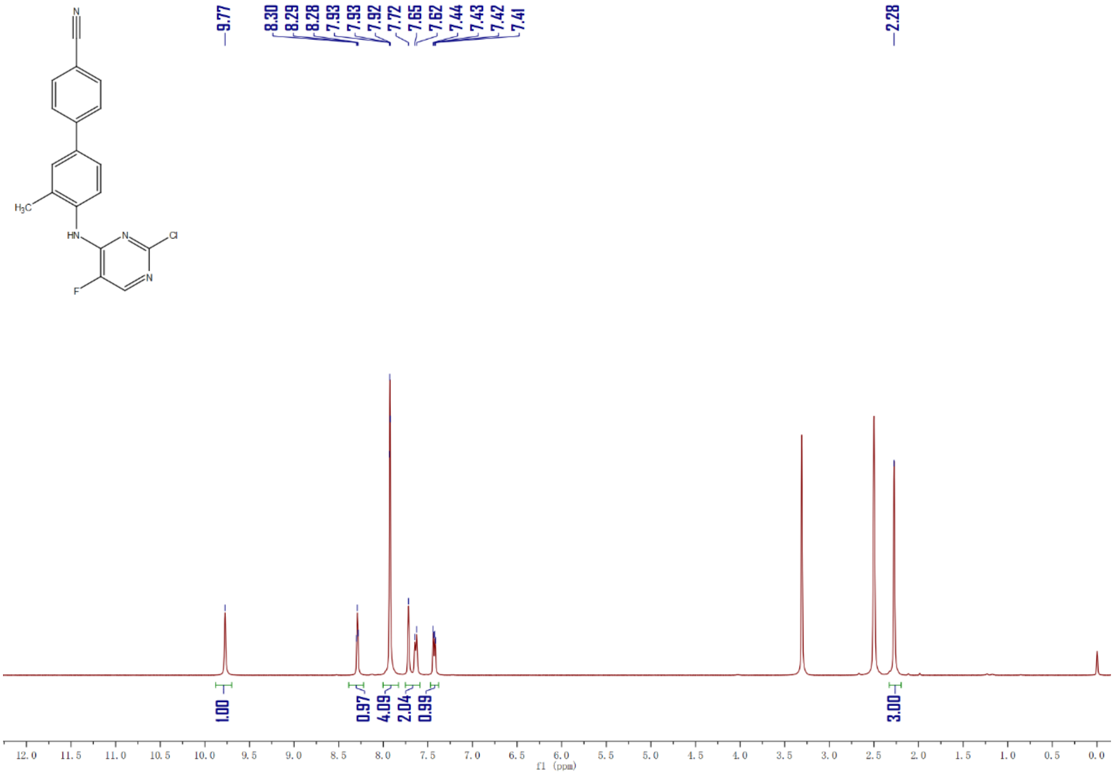

^{13}C NMR

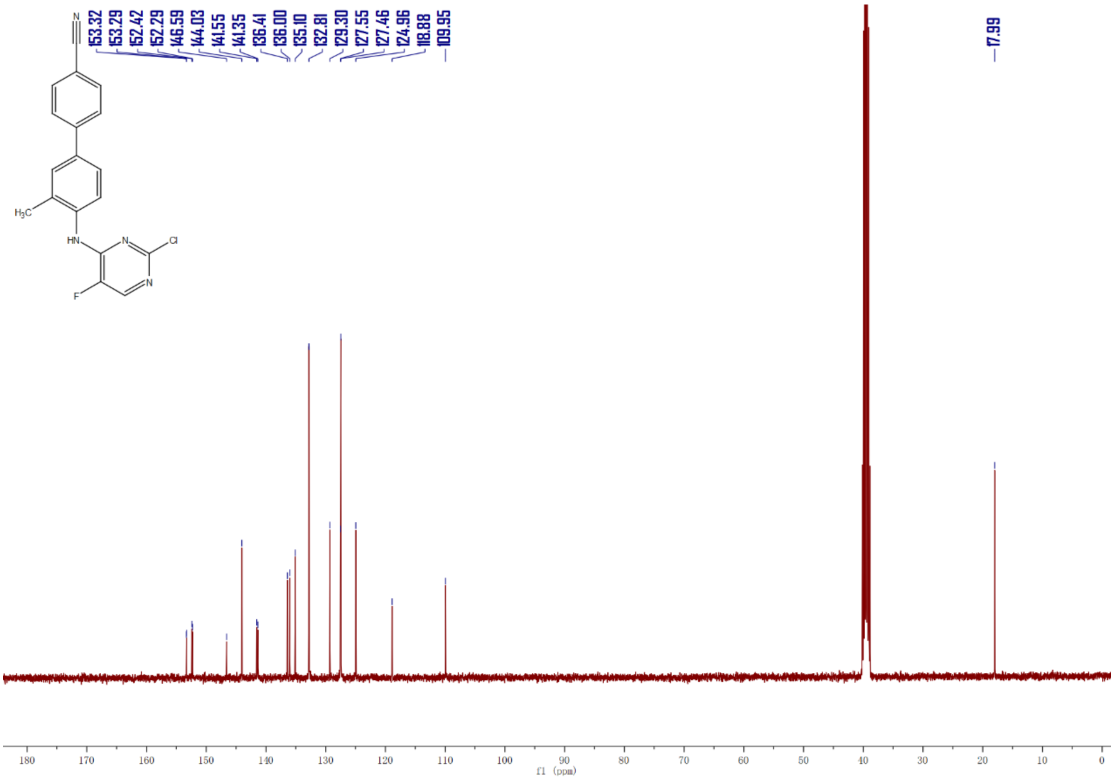

^{19}F NMR

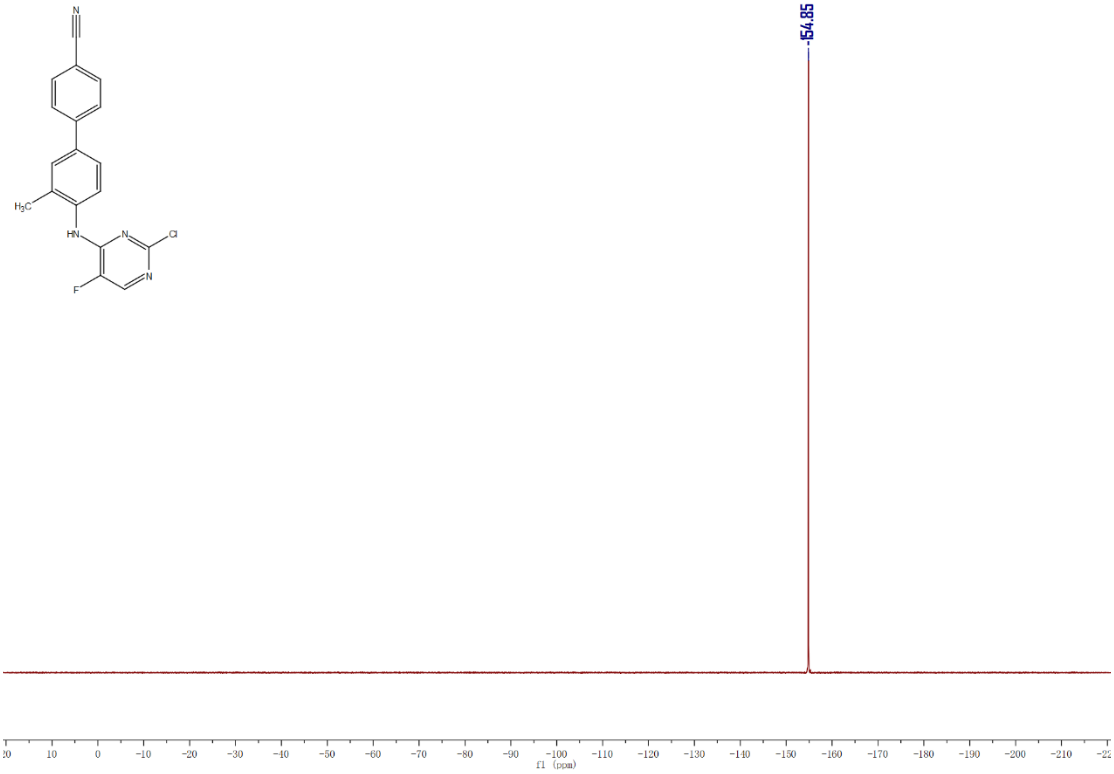

HRMS

Display Report

Analysis Info

Analysis Name	D:\CXM\CXM-20230518\20230518-CXM-010_RB2_01_9666.d	Acquisition Date	5/18/2023 5:24:33 PM
Method	MS-2MIN-POS.m	Operator	BDAL@DE
Sample Name	20230518-CXM-010	Instrument	compact 8255754.20127
Comment			

Acquisition Parameter

Source Type	ESI	Ion Polarity	Positive	Set Nebulizer	2.0 Bar
Focus	Active	Set Capillary	4500 V	Set Dry Heater	200 °C
Scan Begin	50 m/z	Set End Plate Offset	-500 V	Set Dry Gas	8.0 l/min
Scan End	3000 m/z	Set Charging Voltage	2000 V	Set Divert Valve	Waste
		Set Corona	0 nA	Set APCI Heater	0 °C

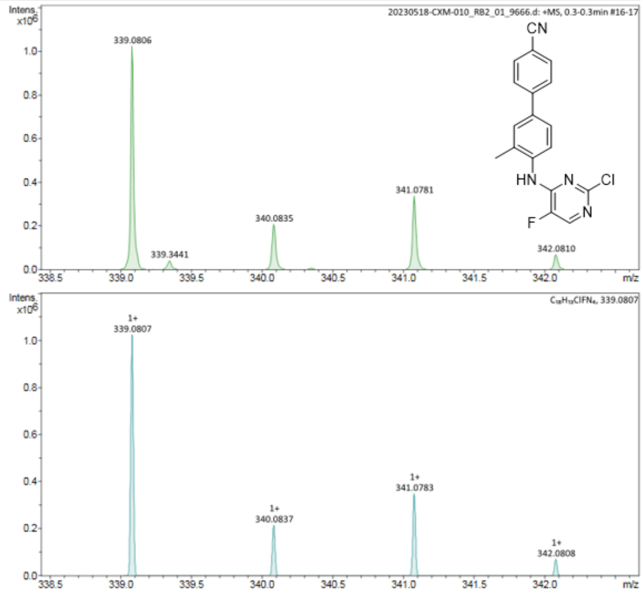

20230518-CXM-010_RB2_01_9666.d
Bruker Compass DataAnalysis 4.2 printed: 5/19/2023 9:34:57 PM by: BDAL@DE Page 1 of 1

^{1}H NMR, ^{13}C NMR, ^{19}F NMR, HRMS spectra of 10d

^{1}H NMR

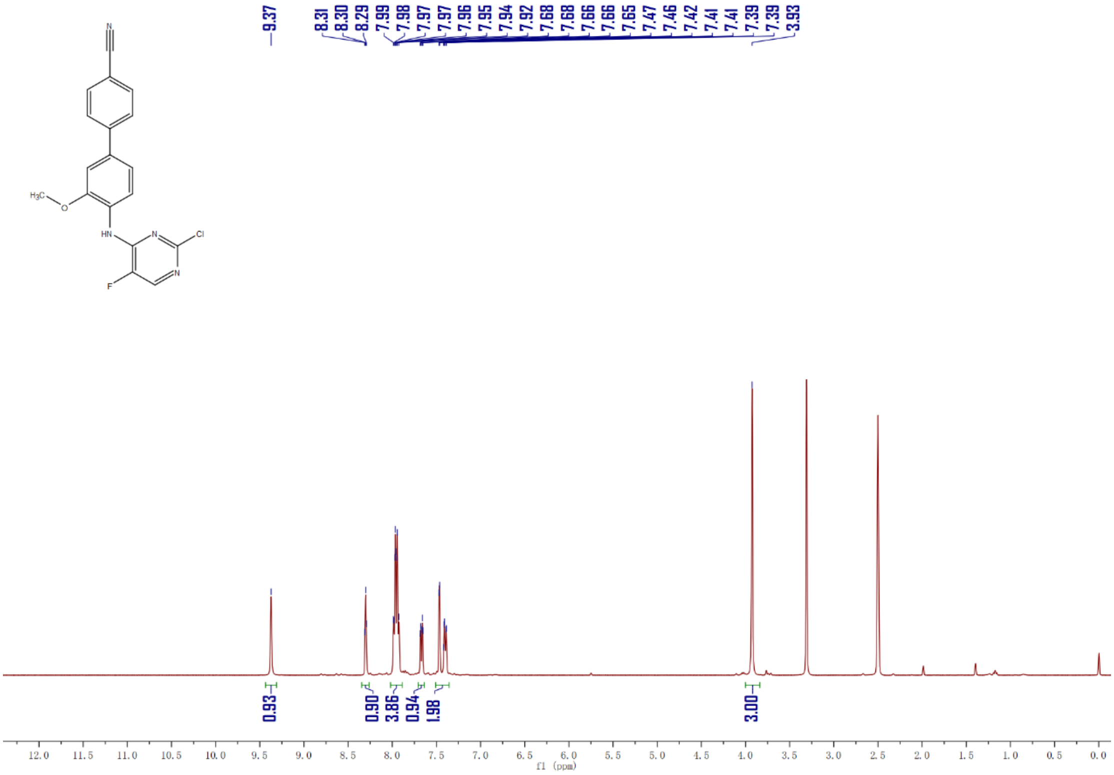

^{13}C NMR

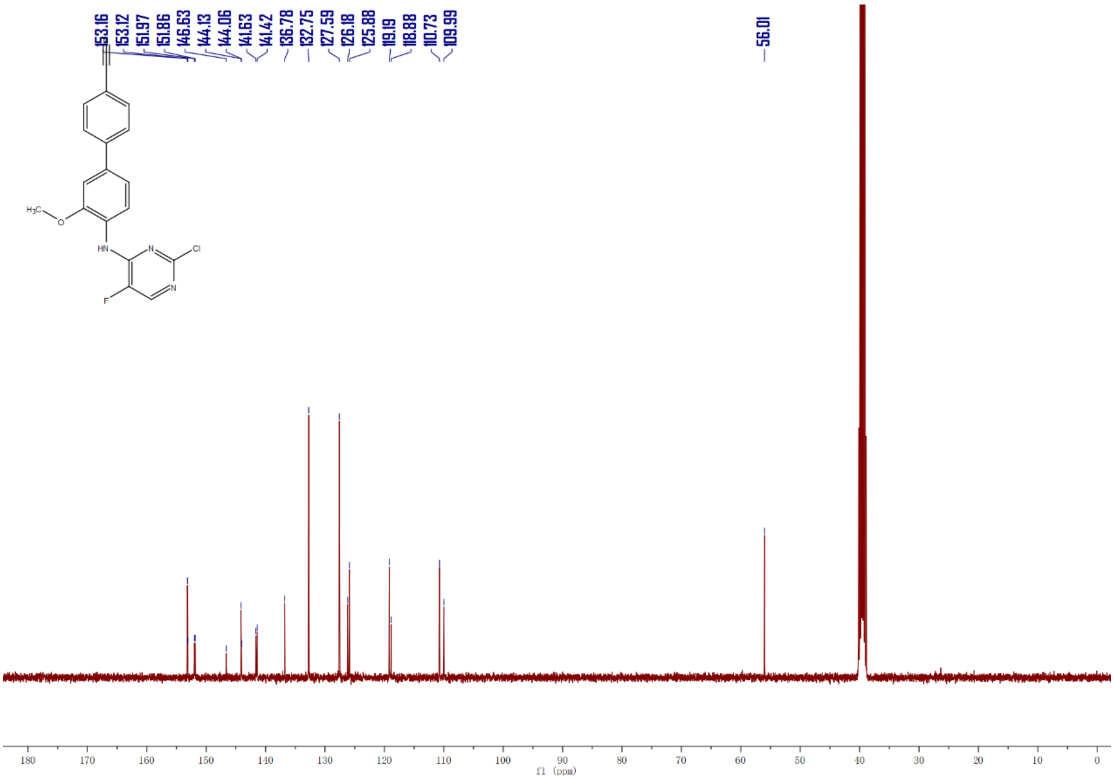

^{19}F NMR

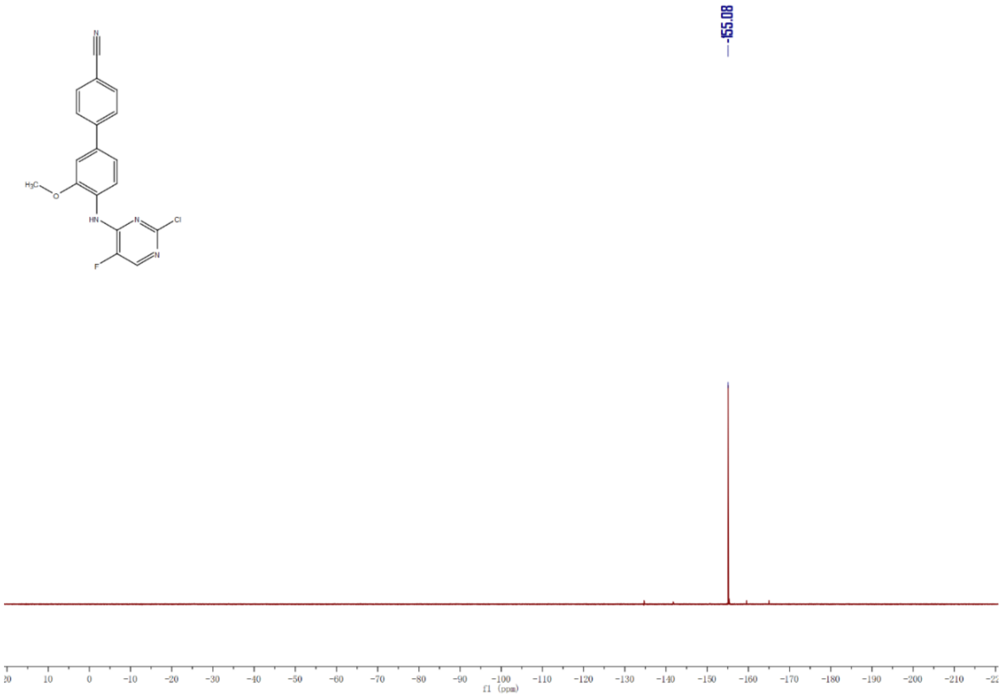

HRMS

Display Report

Analysis Info

Analysis Name D:\CXM\CXM-20230518\20230518-CXM-011_RB3_01_9667.d
Method MS-2MIN-POS.m
Sample Name 20230518-CXM-011
Comment

Acquisition Date 5/18/2023 5:27:20 PM
Operator BDAL@DE
Instrument compact 8255754.20127

Acquisition Parameter

Source Type	ESI	Ion Polarity	Positive	Set Nebulizer	2.0 Bar
Focus	Active	Set Capillary	4500 V	Set Dry Heater	200 °C
Scan Begin	50 m/z	Set End Plate Offset	-500 V	Set Dry Gas	8.0 l/min
Scan End	3000 m/z	Set Charging Voltage	2000 V	Set Divert Valve	Waste
		Set Corona	0 nA	Set APCI Heater	0 °C

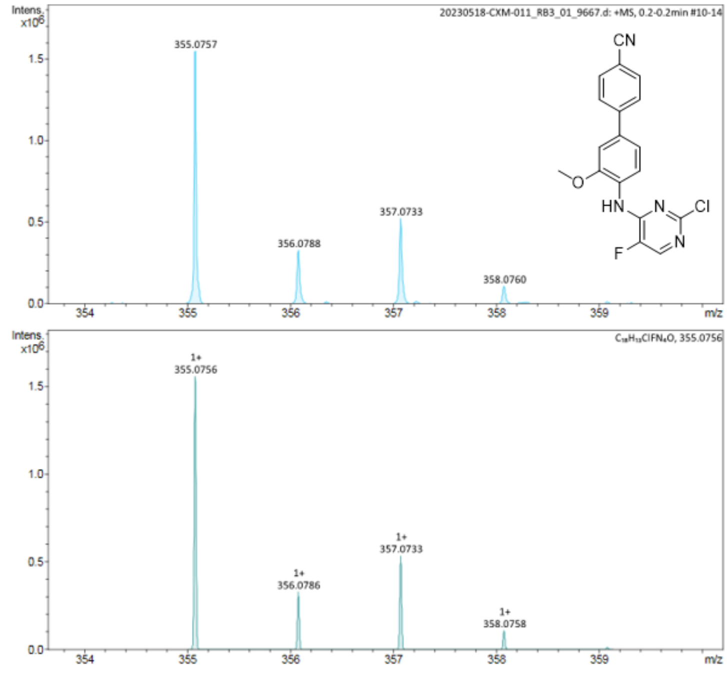

20230518-CXM-011_RB3_01_9667.d
Bruker Compass DataAnalysis 4.2 printed: 5/19/2023 9:38:08 PM by: BDAL@DE Page 1 of 1

^{1}H NMR, ^{13}C NMR, ^{19}F NMR, HRMS spectra of 10e

^{1}H NMR

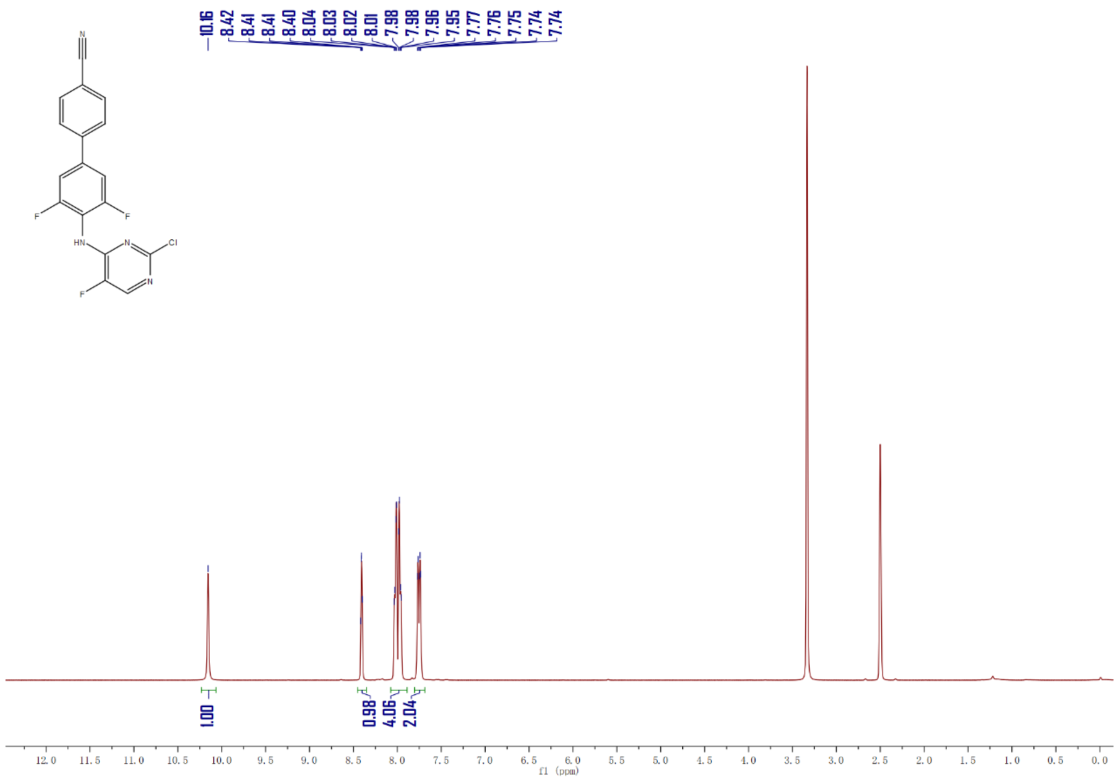

^{13}C NMR

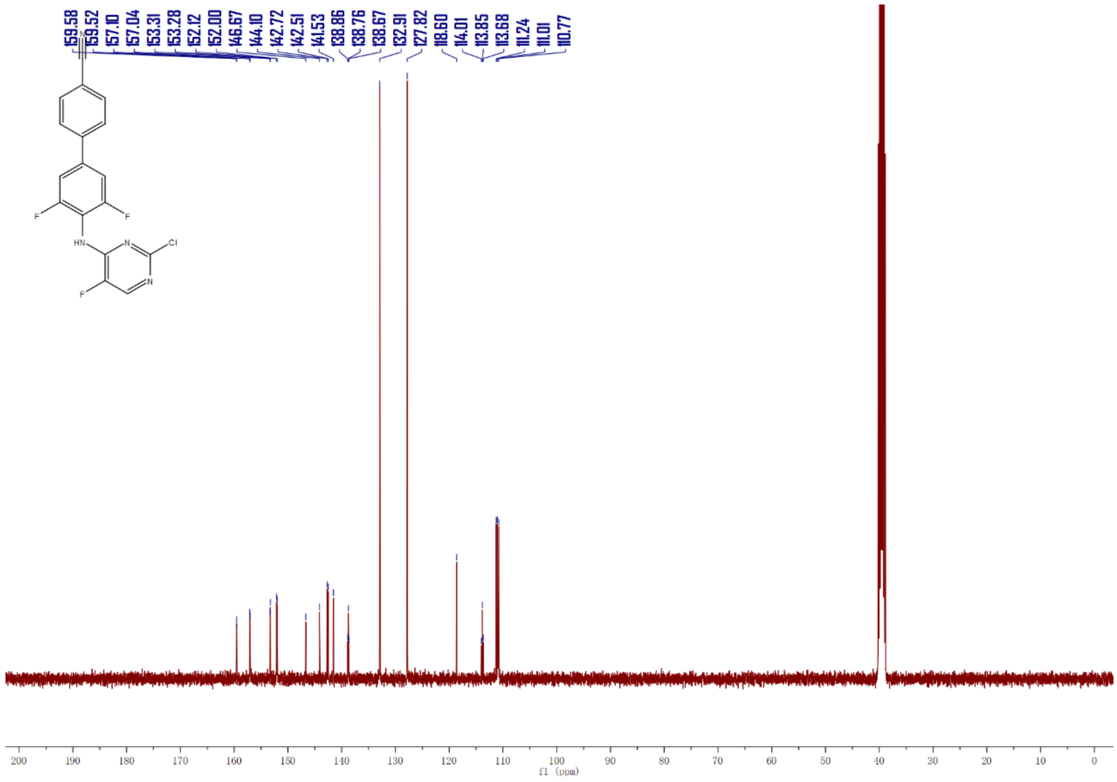

^{19}F NMR

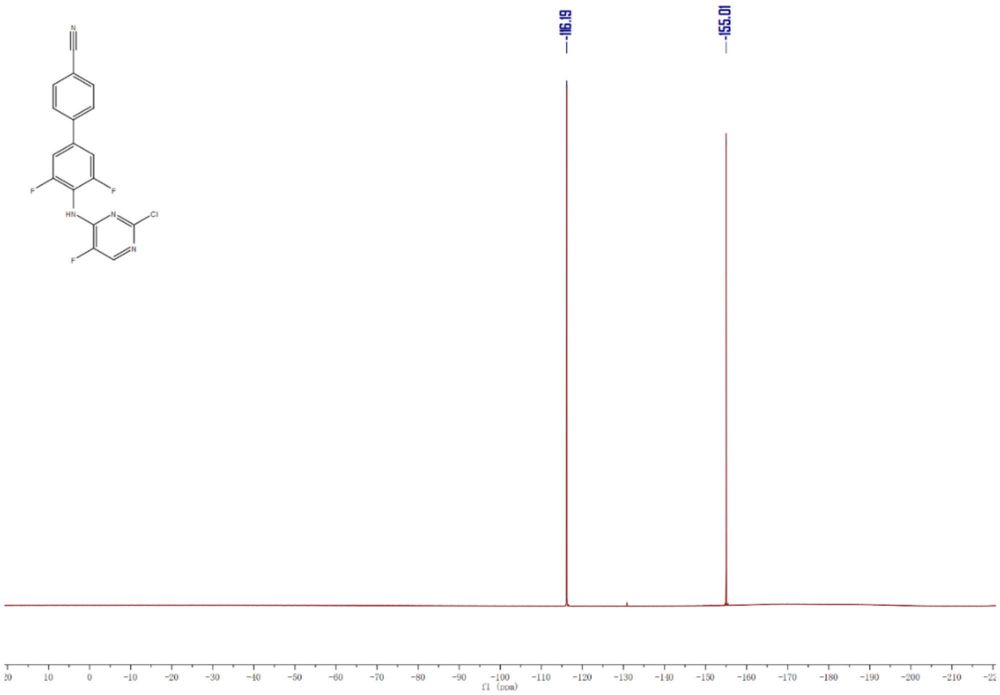

HRMS

Display Report

Analysis Info

Analysis Name	D:\CXM\CXM-20230518\20230518-CXM-012_RB4_01_9668.d	Acquisition Date	5/18/2023 5:30:05 PM
Method	MS-2MIN-POS.m	Operator	BDAL@DE
Sample Name	20230518-CXM-012	Instrument	compact 8255754.20127
Comment			

Acquisition Parameter

Source Type	ESI	Ion Polarity	Positive	Set Nebulizer	2.0 Bar
Focus	Active	Set Capillary	4500 V	Set Dry Heater	200 °C
Scan Begin	50 m/z	Set End Plate Offset	-500 V	Set Dry Gas	8.0 l/min
Scan End	3000 m/z	Set Charging Voltage	2000 V	Set Divert Valve	Waste
		Set Corona	0 nA	Set APCI Heater	0 °C

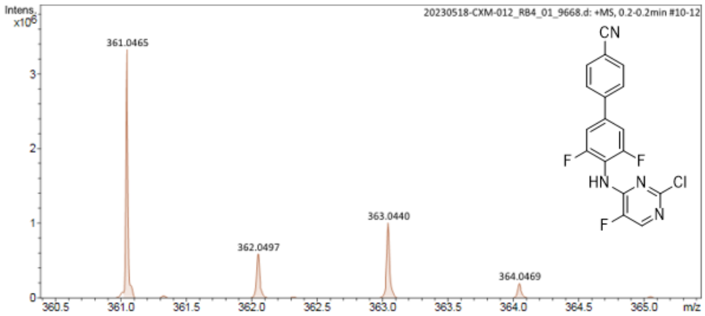

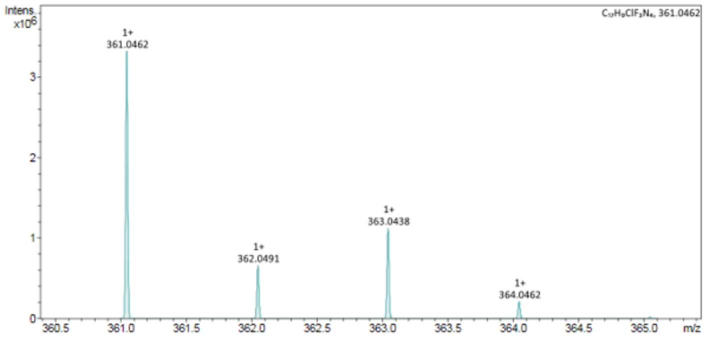

20230518-CXM-012_RB4_01_9668.d

Bruker Compass DataAnalysis 4.2 printed: 5/19/2023 9:41:48 PM by: BDAL@DE Page 1 of 1

^{1}H NMR, ^{13}C NMR, ^{19}F NMR, HRMS spectra of 10f

^{1}H NMR

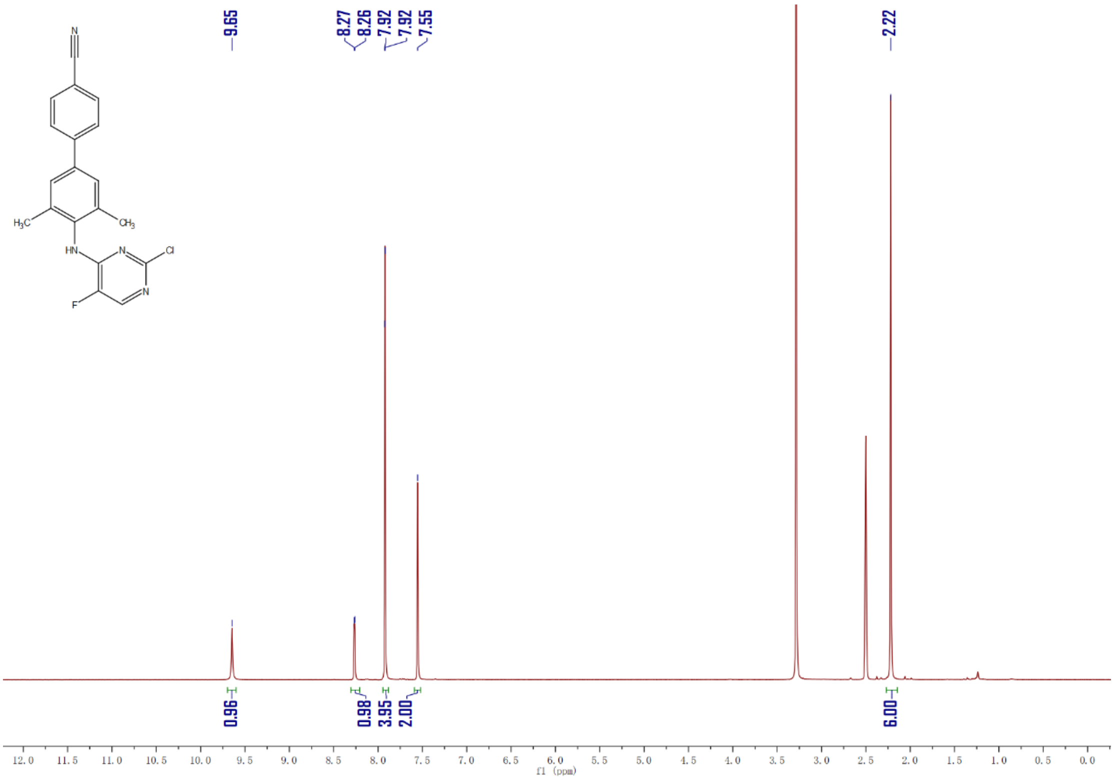

^{13}C NMR

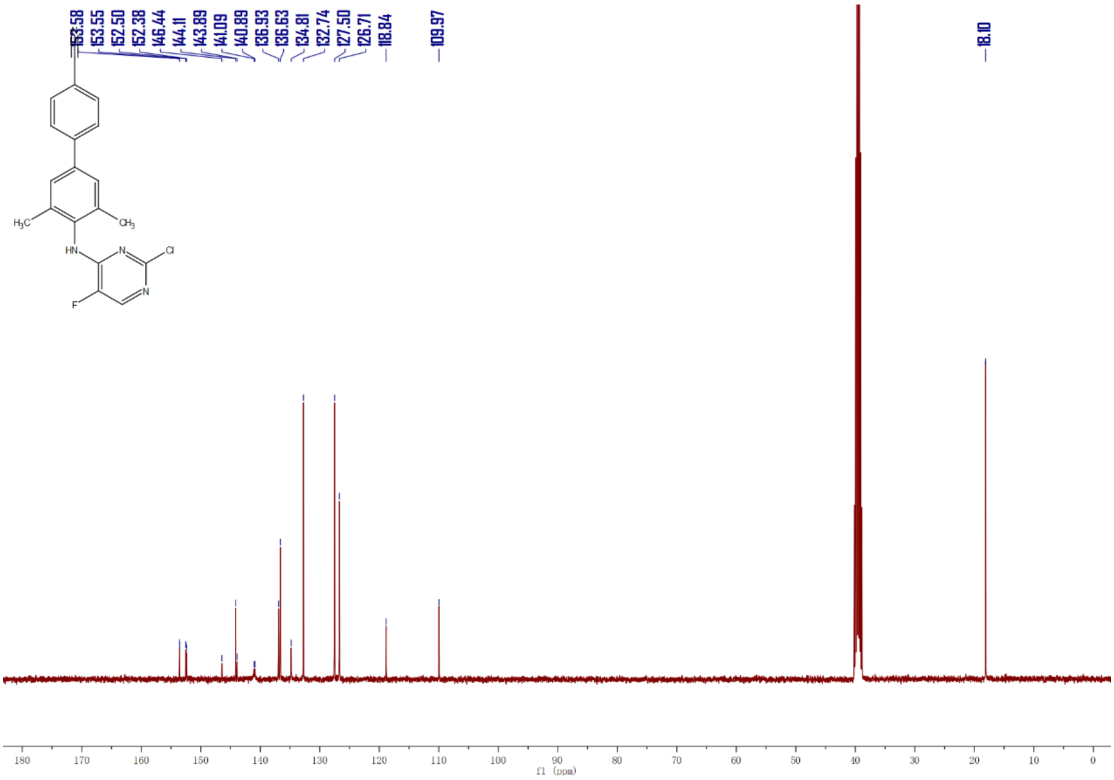

^{19}F NMR

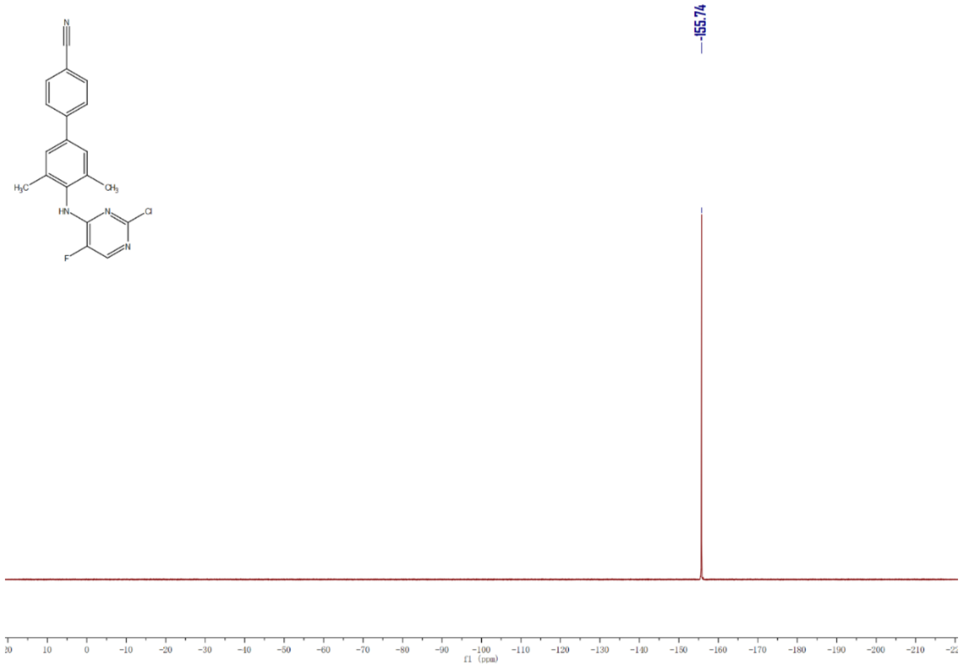

HRMS

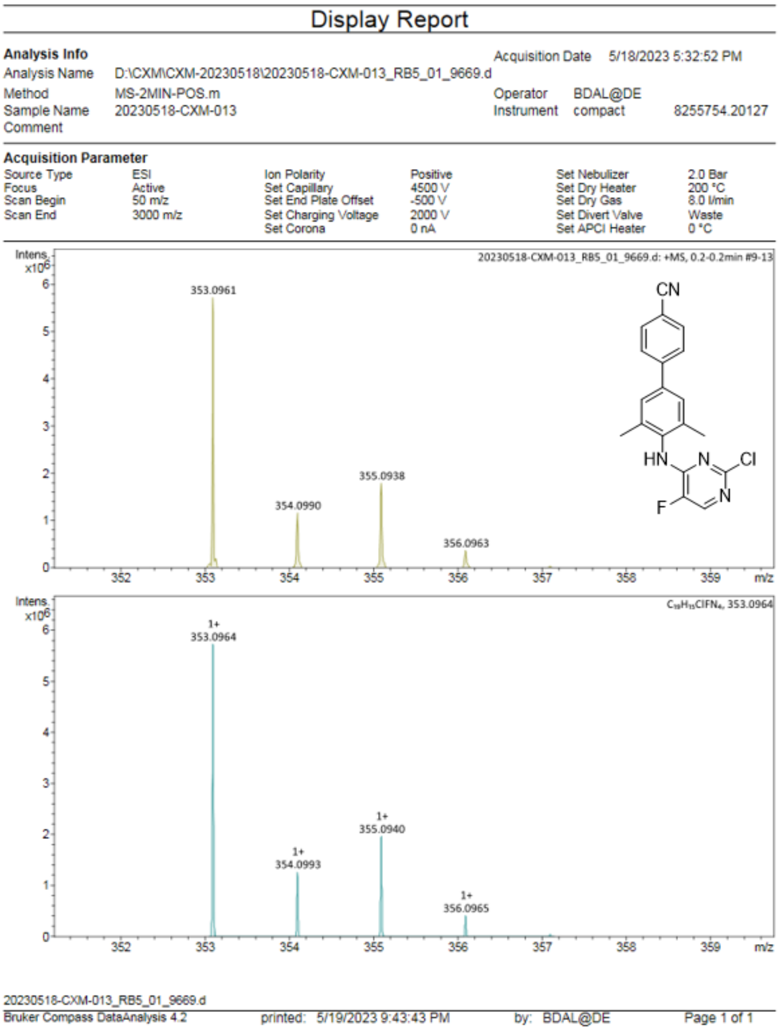

Display Report

Analysis Info

Analysis Name D:\CXM\CXM-20230518\20230518-CXM-013_RB5_01_9669.d

Method MS-2MIN-POS.m

Sample Name 20230518-CXM-013

Comment

Acquisition Date 5/18/2023 5:32:52 PM

Operator BDAL@DE

Instrument compact 8255754.20127

Acquisition Parameter

Source Type	ESI	Ion Polarity	Positive	Set Nebulizer	2.0 Bar
Focus	Active	Set Capillary	4500 V	Set Dry Heater	200 °C
Scan Begin	50 m/z	Set End Plate Offset	-500 V	Set Dry Gas	8.0 l/min
Scan End	3000 m/z	Set Charging Voltage	2000 V	Set Divert Valve	Waste
		Set Corona	0 nA	Set APCI Heater	0 °C

20230518-CXM-013_RB5_01_9669.d

Bruker Compass DataAnalysis 4.2 printed: 5/19/2023 9:43:43 PM by: BDAL@DE Page 1 of 1

^{1}H NMR, ^{13}C NMR, ^{19}F NMR, HRMS spectra of 10g

^{1}H NMR

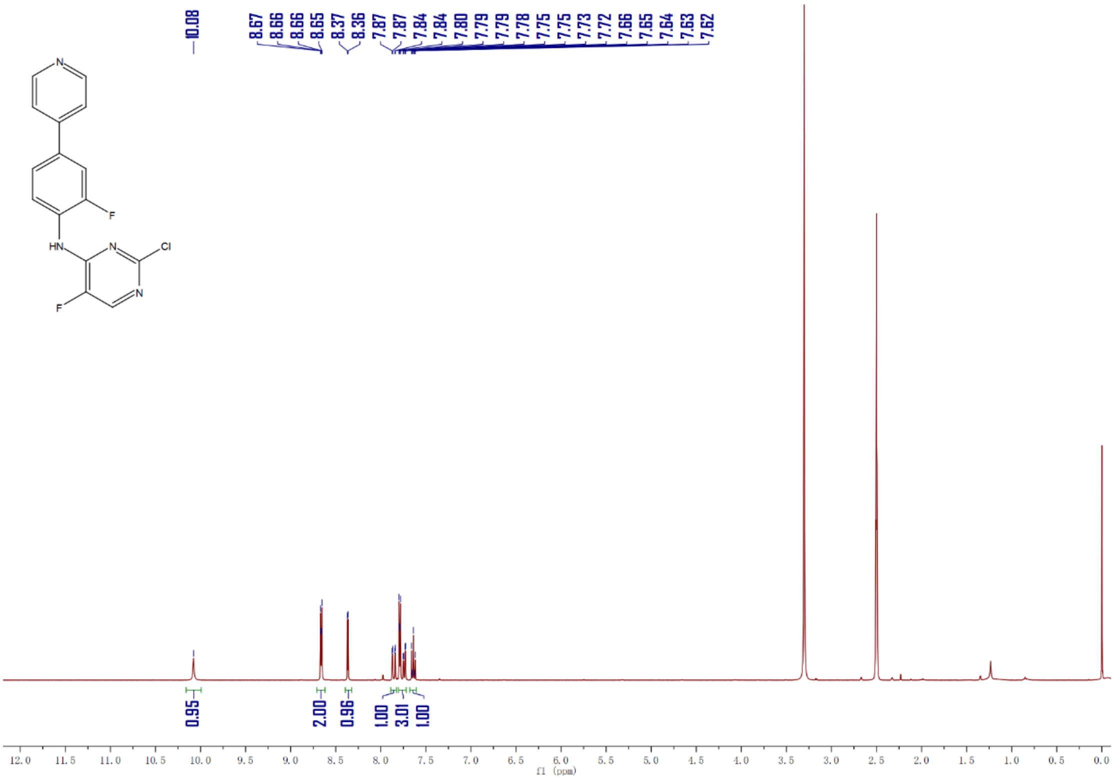

^{13}C NMR

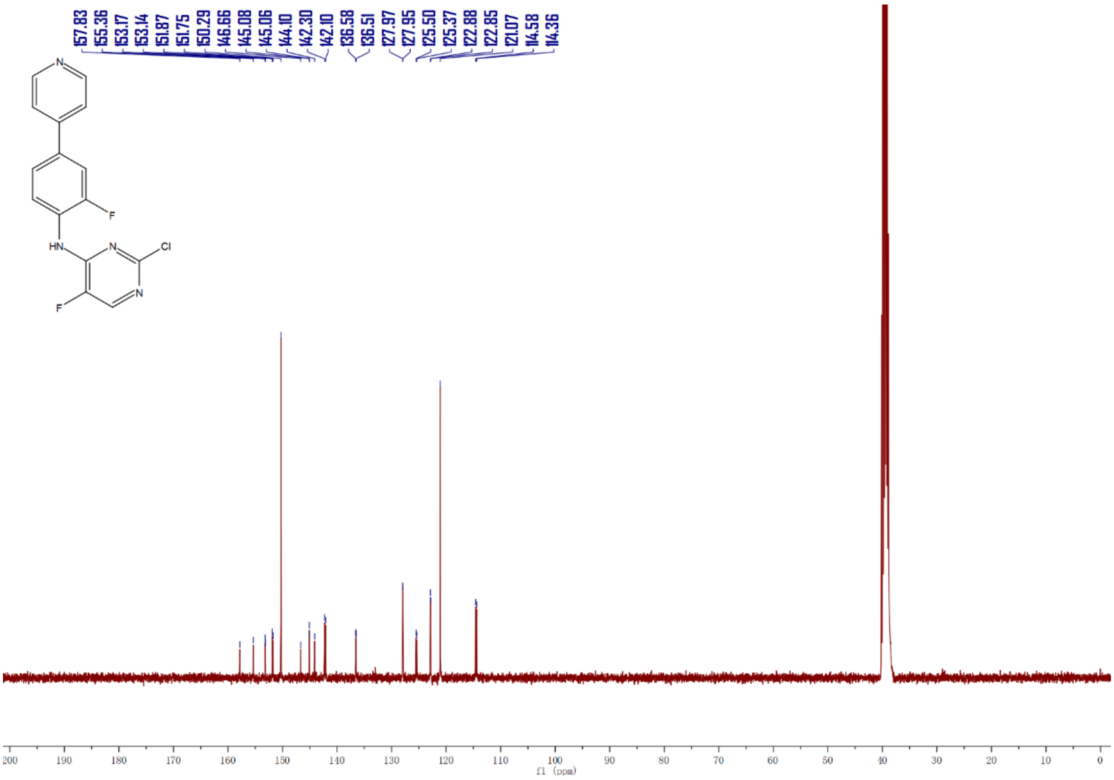

^{19}F NMR

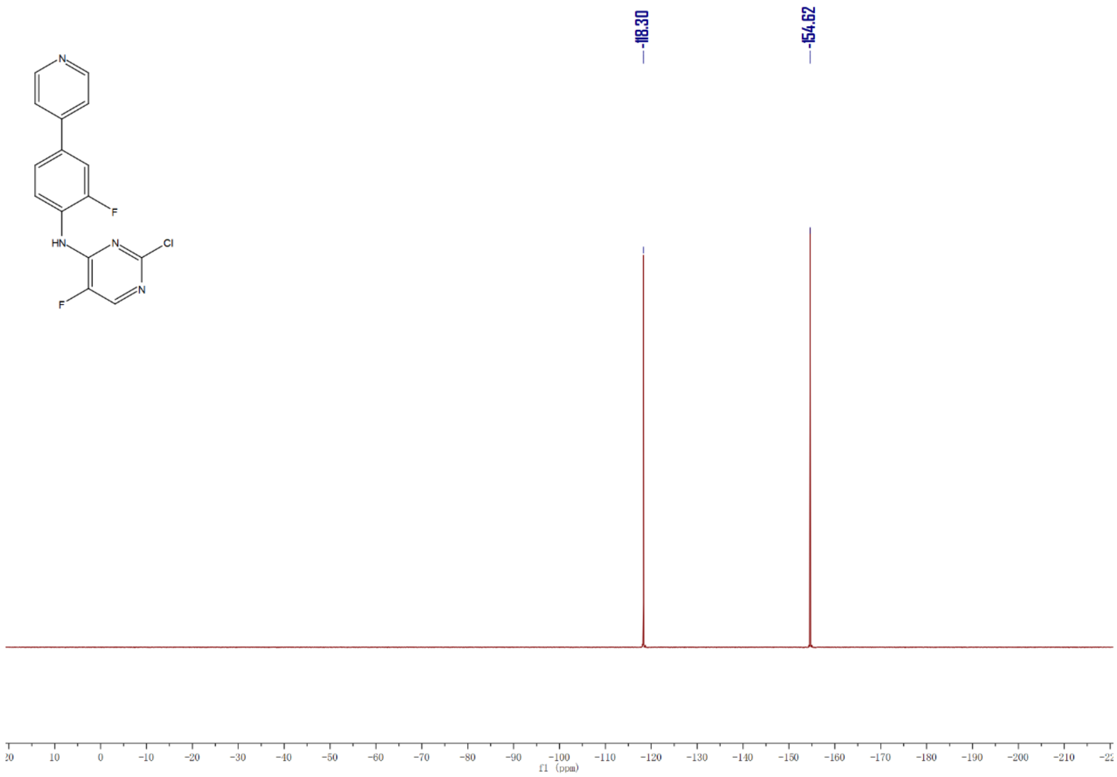

HRMS

Display Report

Analysis Info

Analysis Name	D:\CXM\CXM-20230518\20230518-CXM-017_RC1_01_9673.d	Acquisition Date	5/18/2023 5:43:54 PM
Method	MS-2MIN-POS.m	Operator	BDAL@DE
Sample Name	20230518-CXM-017	Instrument	compact 8255754.20127
Comment			

Acquisition Parameter

Source Type	ESI	Ion Polarity	Positive	Set Nebulizer	2.0 Bar
Focus	Active	Set Capillary	4500 V	Set Dry Heater	200 °C
Scan Begin	50 m/z	Set End Plate Offset	-500 V	Set Dry Gas	8.0 l/min
Scan End	3000 m/z	Set Charging Voltage	2000 V	Set Divert Valve	Waste
		Set Corona	0 nA	Set APCI Heater	0 °C

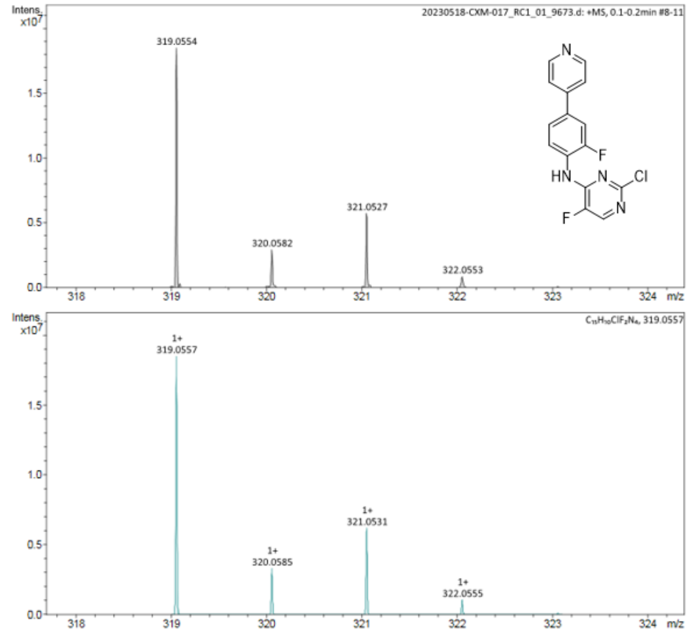

20230518-CXM-017_RC1_01_9673.d
Bruker Compass DataAnalysis 4.2 printed: 5/19/2023 10:00:04 PM by: BDAL@DE Page 1 of 1

^{1}H NMR, ^{13}C NMR, ^{19}F NMR, HRMS spectra of 10h

^{1}H NMR

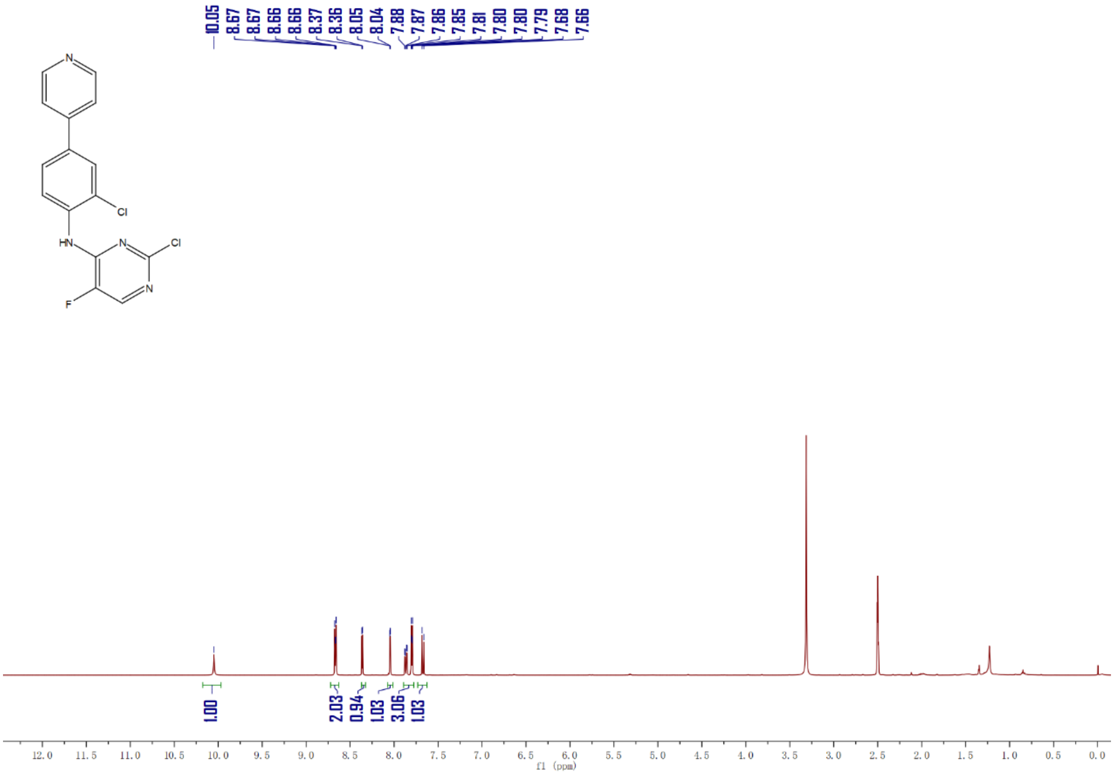

^{13}C NMR

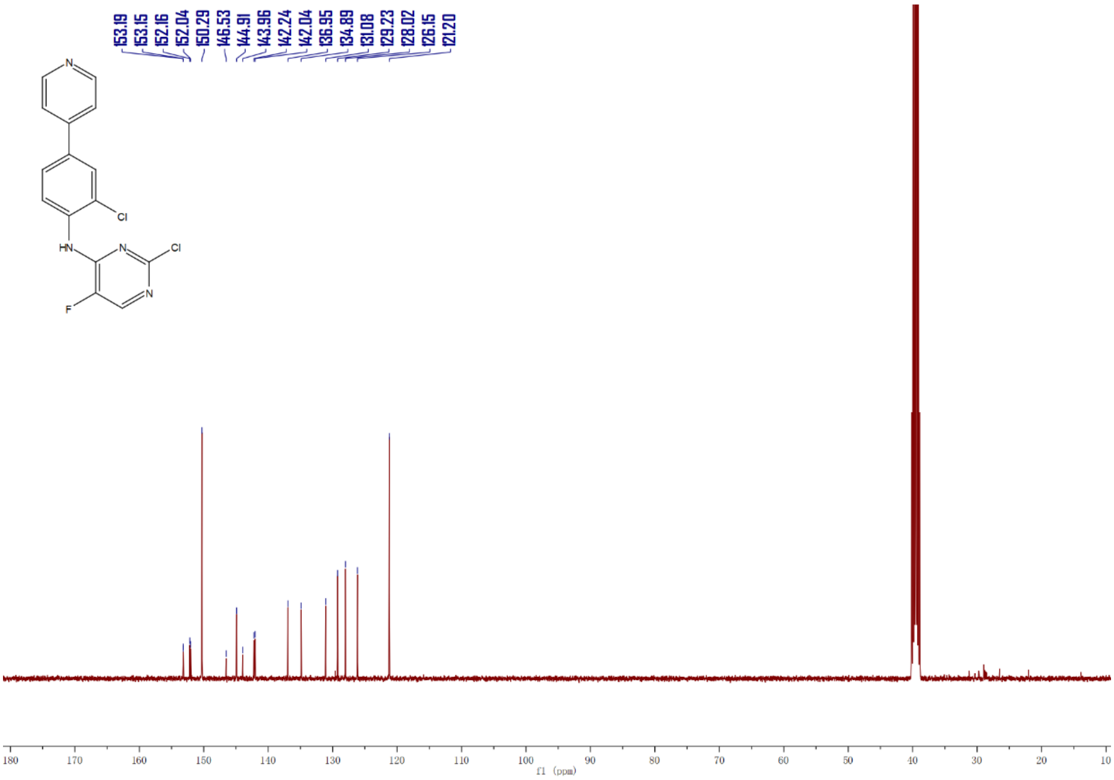

^{19}F NMR

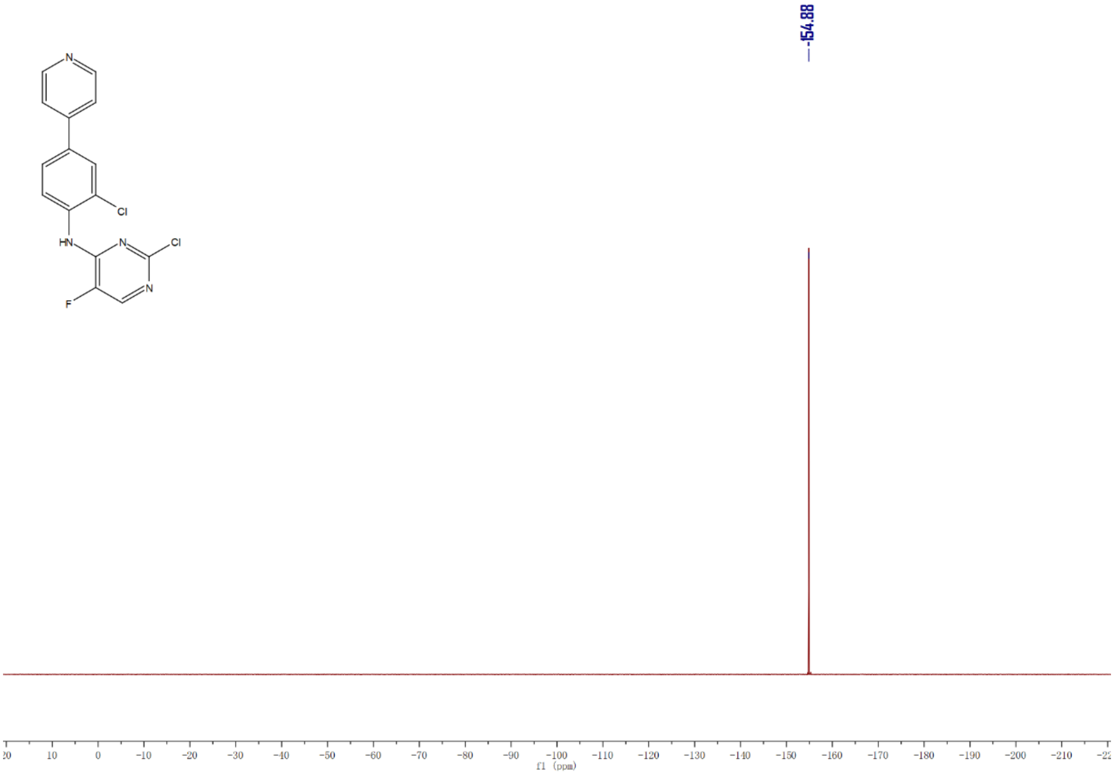

HRMS

Display Report

Analysis Info

Analysis Name	D:\CXM\CXM-20230518\20230518-CXM-018_RC2_01_9674.d	Acquisition Date	5/18/2023 5:46:39 PM
Method	MS-2MIN-POS.m	Operator	BDAL@DE
Sample Name	20230518-CXM-018	Instrument	compact 8255754.20127
Comment			

Acquisition Parameter

Source Type	ESI	Ion Polarity	Positive	Set Nebulizer	2.0 Bar
Focus	Active	Set Capillary	4500 V	Set Dry Heater	200 °C
Scan Begin	50 m/z	Set End Plate Offset	-500 V	Set Dry Gas	8.0 l/min
Scan End	3000 m/z	Set Charging Voltage	2000 V	Set Divert Valve	Waste
		Set Corona	0 nA	Set APCI Heater	0 °C

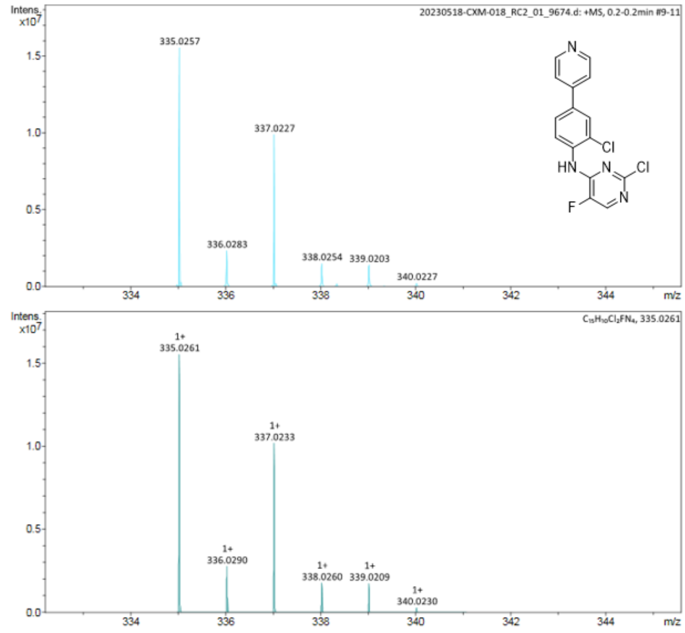

20230518-CXM-018_RC2_01_9674.d
Bruker Compass DataAnalysis 4.2 printed: 5/19/2023 10:01:40 PM by: BDAL@DE Page 1 of 1

^{1}H NMR, ^{13}C NMR, ^{19}F NMR, HRMS spectra of 10i

^{1}H NMR

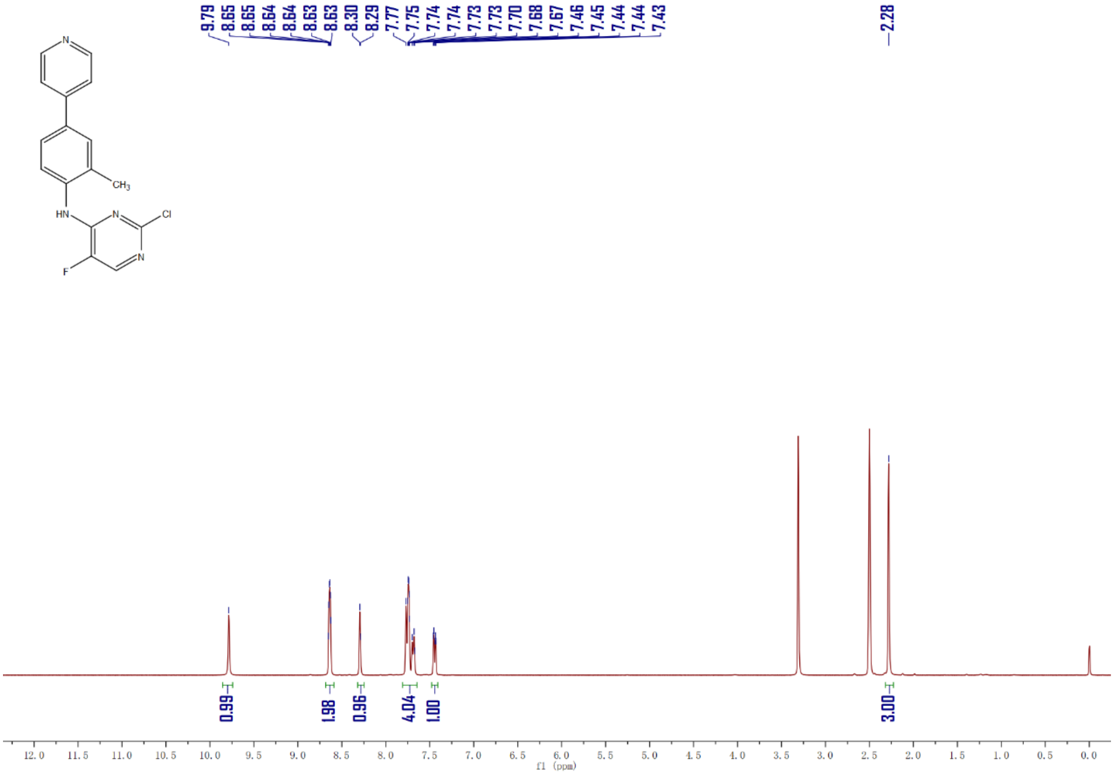

^{13}C NMR

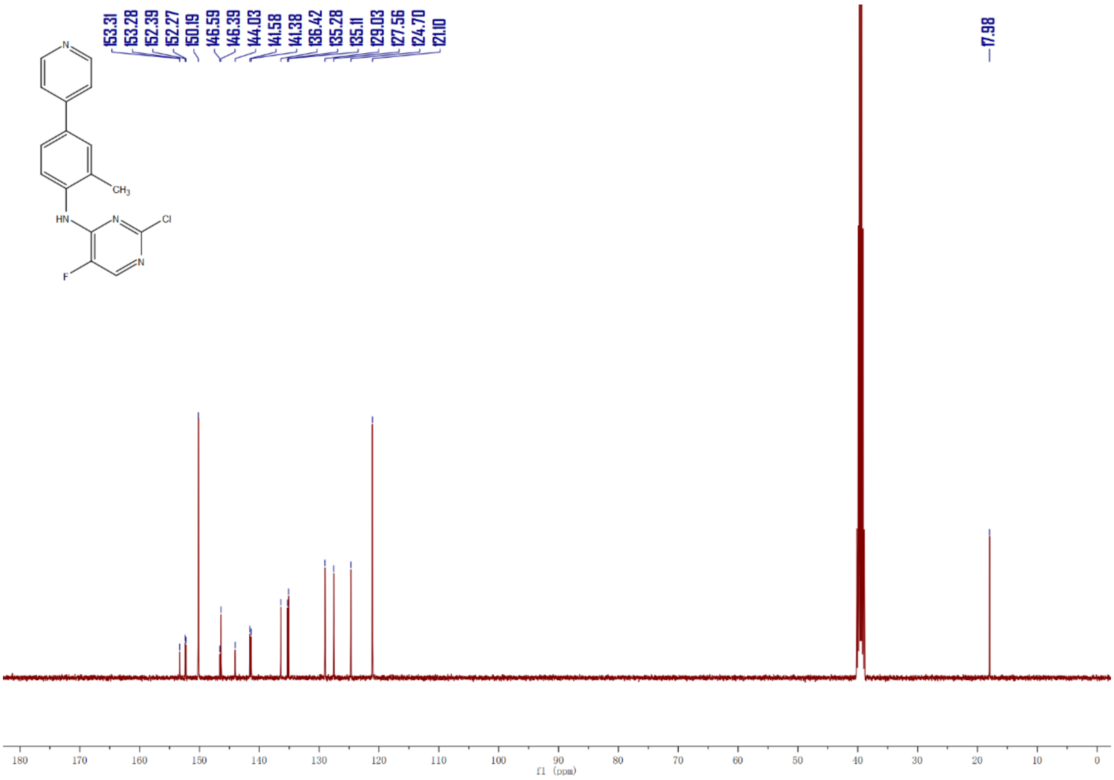

^{19}F NMR

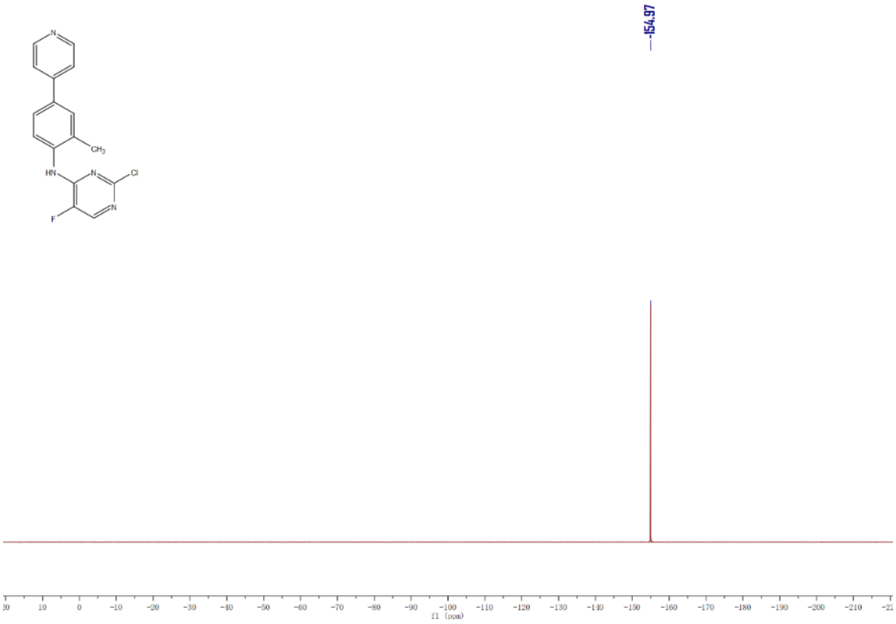

—154.97
N
CH3
HN
Cl
F
f1 (ppm)

HRMS

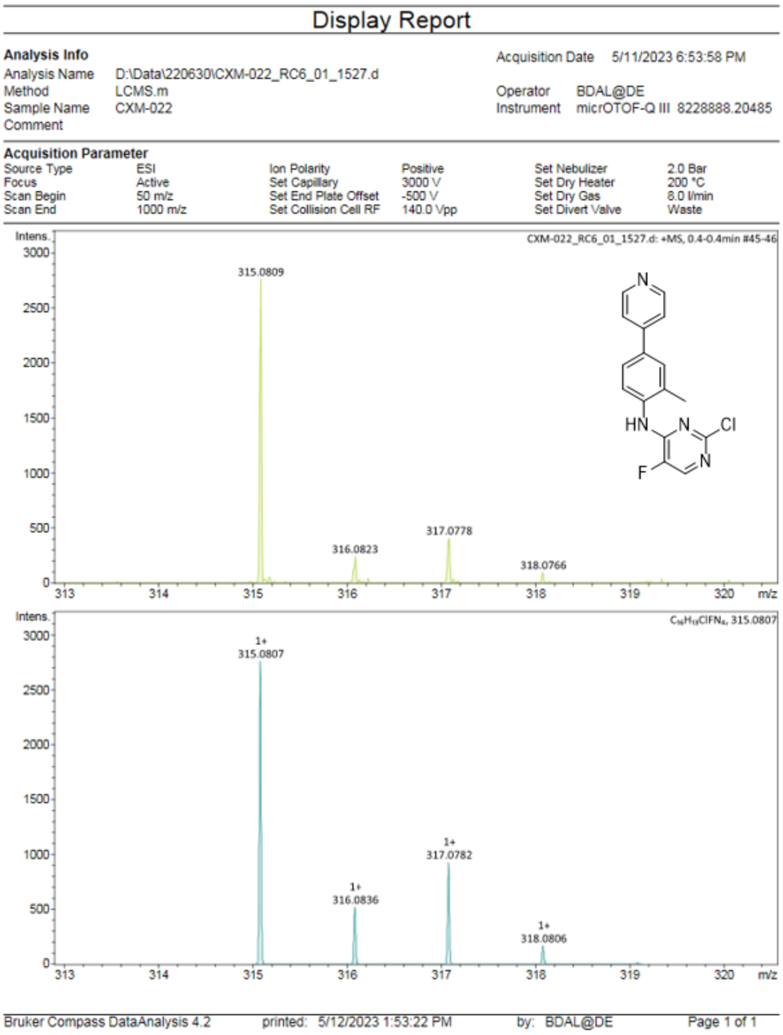

Display Report
Analysis Info
Analysis Name D:\Data\220630\CXM-022_RC6_01_1527.d
Method LCMS.m
Sample Name CXM-022
Comment
Acquisition Date 5/11/2023 6:53:58 PM
Operator BDAL@DE
Instrument microTOF-Q III 8228888.20485
Acquisition Parameter
Source Type ESI
Focus Active
Scan Begin 50 m/z
Scan End 1000 m/z
Ion Polarity Positive
Set Capillary 3000 V
Set End Plate Offset -500 V
Set Collision Cell RF 140.0 Vpp
Set Nebulizer 2.0 Bar
Set Dry Heater 200 °C
Set Dry Gas 8.0 l/min
Set Divert Valve Waste
CXM-022_RC6_01_1527.d: +MS, 0.4-0.4min #45-46
Intens.
315.0809
316.0823
317.0778
318.0766
m/z
C16H13ClFN4, 315.0807
1+ 315.0807
1+ 316.0836
1+ 317.0782
1+ 318.0806
HN
N
Cl
F
Bruker Compass DataAnalysis 4.2
printed: 5/12/2023 1:53:22 PM
by: BDAL@DE
Page 1 of 1

^{1}H NMR, ^{13}C NMR, ^{19}F NMR, HRMS spectra of 10j

^{1}H NMR

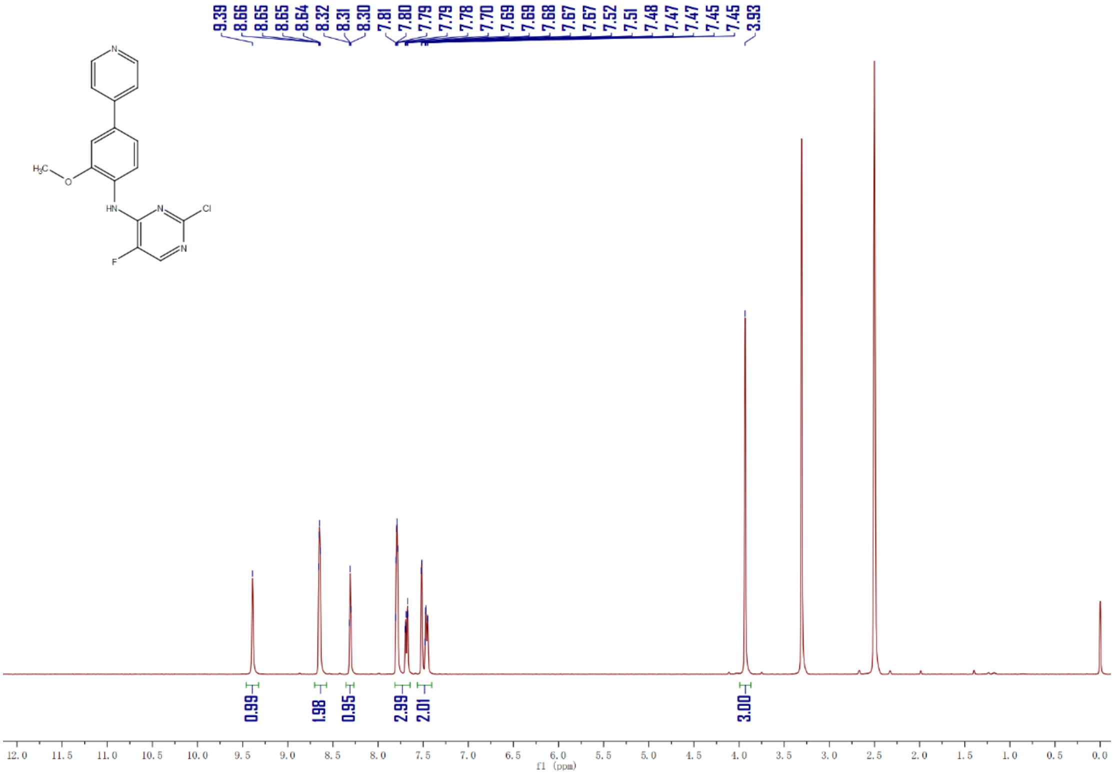

^{13}C NMR

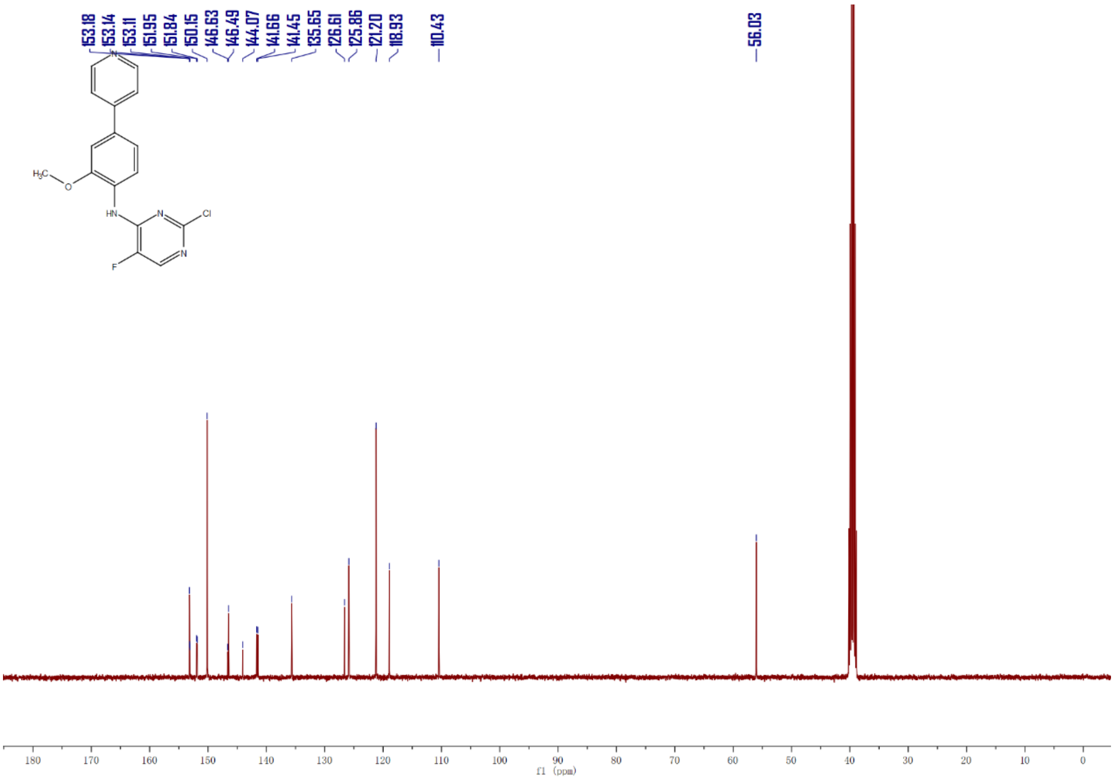

^{19}F NMR

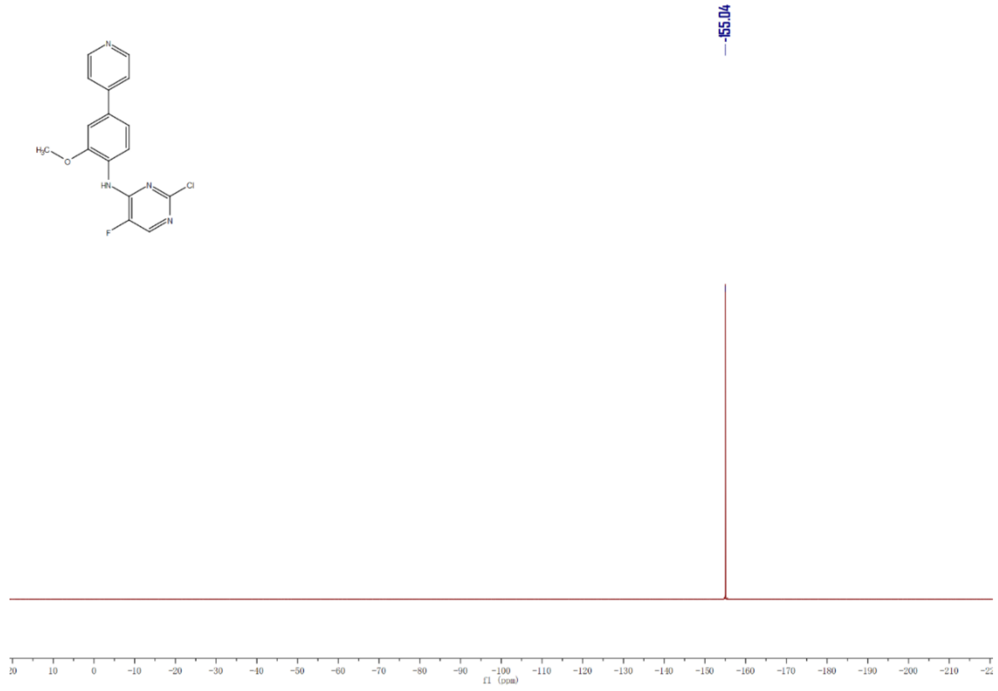

HRMS

Display Report

Analysis Info

Analysis Name	D:\Data\220630\CXM-023_RC7_01_1528.d	Acquisition Date	5/11/2023 6:56:45 PM
Method	LCMS.m	Operator	BDAL@DE
Sample Name	CXM-023	Instrument	micrOTOF-Q III 8228888.20485
Comment			

Acquisition Parameter

Source Type	ESI	Ion Polarity	Positive	Set Nebulizer	2.0 Bar
Focus	Active	Set Capillary	3000 V	Set Dry Heater	200 °C
Scan Begin	50 m/z	Set End Plate Offset	-500 V	Set Dry Gas	8.0 l/min
Scan End	1000 m/z	Set Collision Cell RF	140.0 Vpp	Set Divert Valve	Waste

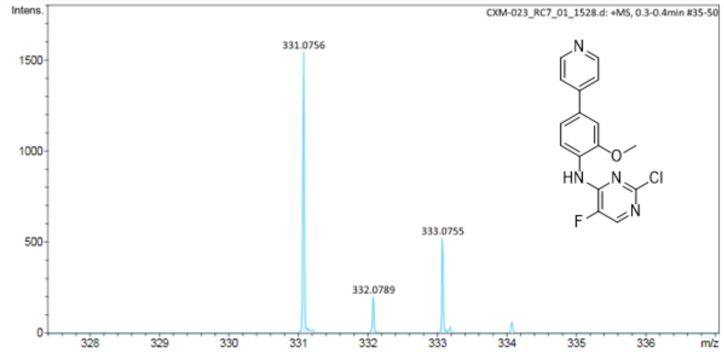

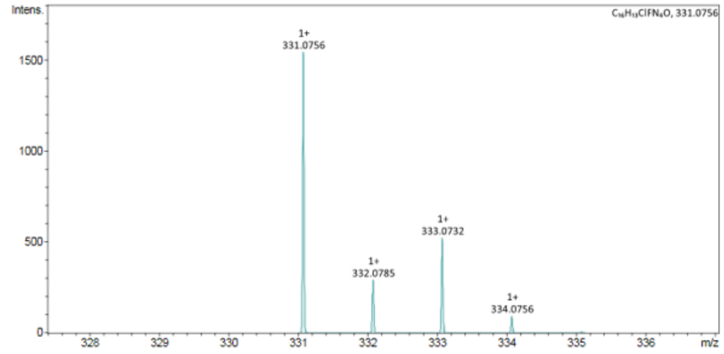

Bruker Compass DataAnalysis 4.2 printed: 5/12/2023 2:01:18 PM by: BDAL@DE Page 1 of 1

^{1}H NMR, ^{13}C NMR, ^{19}F NMR, HRMS spectra of 10k

^{1}H NMR

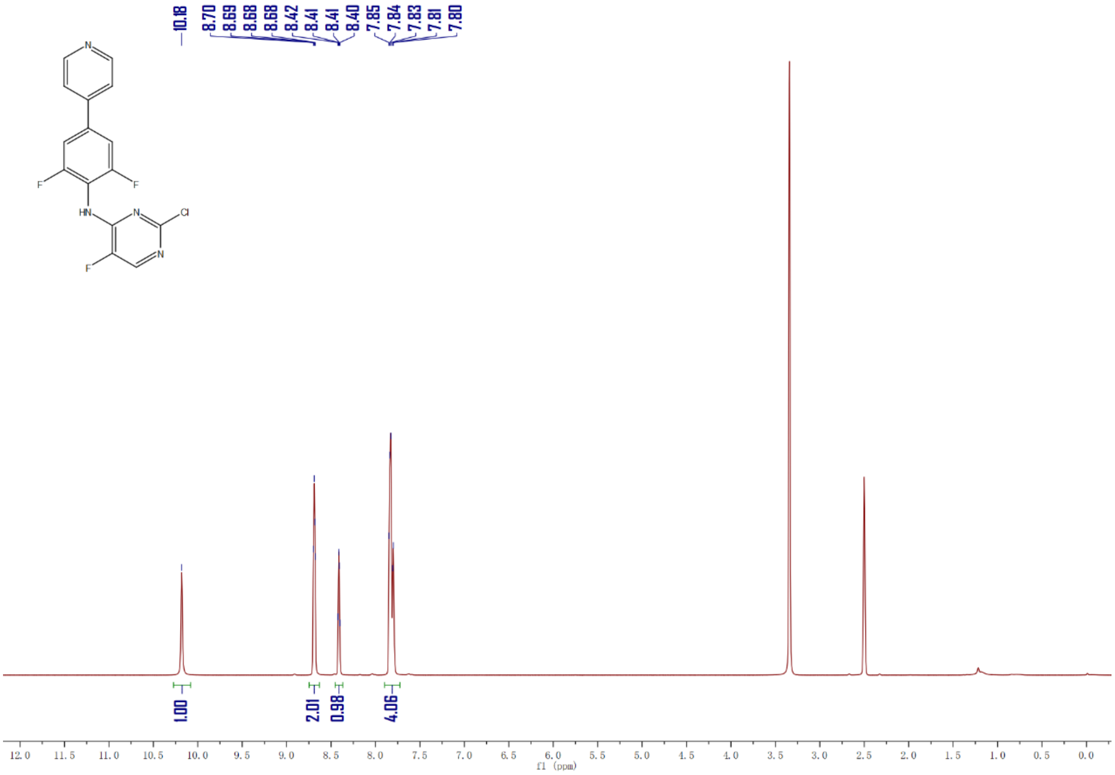

^{13}C NMR

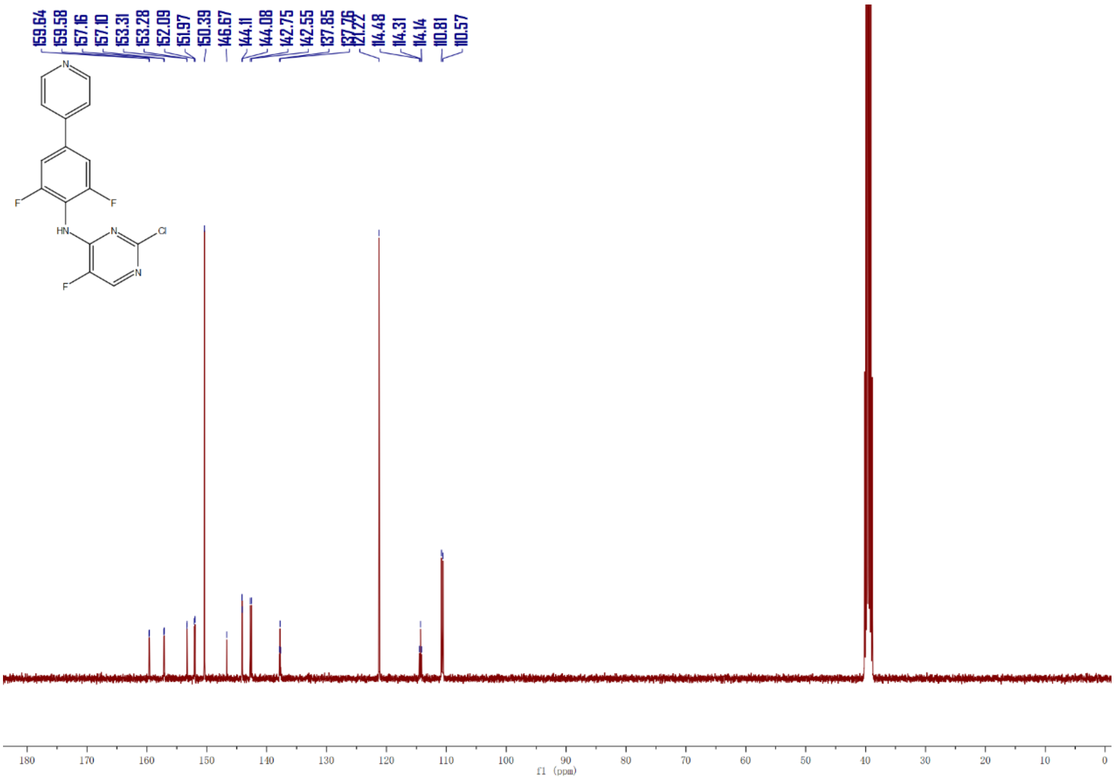

^{19}F NMR

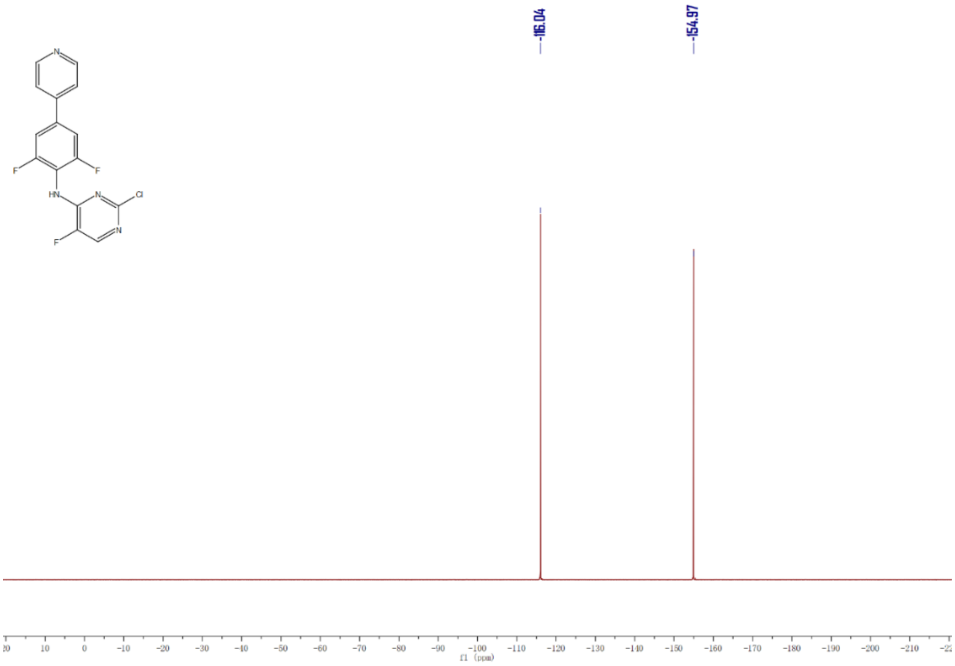

HRMS

Display Report

Analysis Info

Analysis Name	D:\CXM\CXM-20230518\20230518-CXM-019_RC3_01_9675.d	Acquisition Date	5/18/2023 5:49:26 PM
Method	MS-2MIN-POS.m	Operator	BDAL@DE
Sample Name	20230518-CXM-019	Instrument	compact 8255754.20127
Comment			

Acquisition Parameter

Source Type	ESI	Ion Polarity	Positive	Set Nebulizer	2.0 Bar
Focus	Active	Set Capillary	4500 V	Set Dry Heater	200 °C
Scan Begin	50 m/z	Set End Plate Offset	-500 V	Set Dry Gas	8.0 l/min
Scan End	3000 m/z	Set Charging Voltage	2000 V	Set Divert Valve	Waste
		Set Corona	0 nA	Set APCI Heater	0 °C

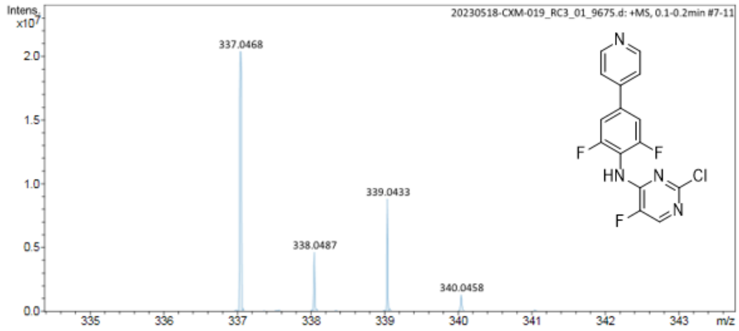

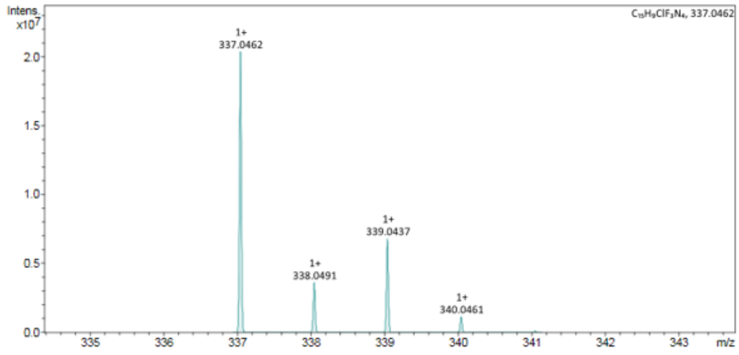

20230518-CXM-019_RC3_01_9675.d

Bruker Compass DataAnalysis 4.2 printed: 5/19/2023 10:03:13 PM by: BDAL@DE Page 1 of 1

^{1}H NMR, ^{13}C NMR, ^{19}F NMR, HRMS spectra of 10l

^{1}H NMR

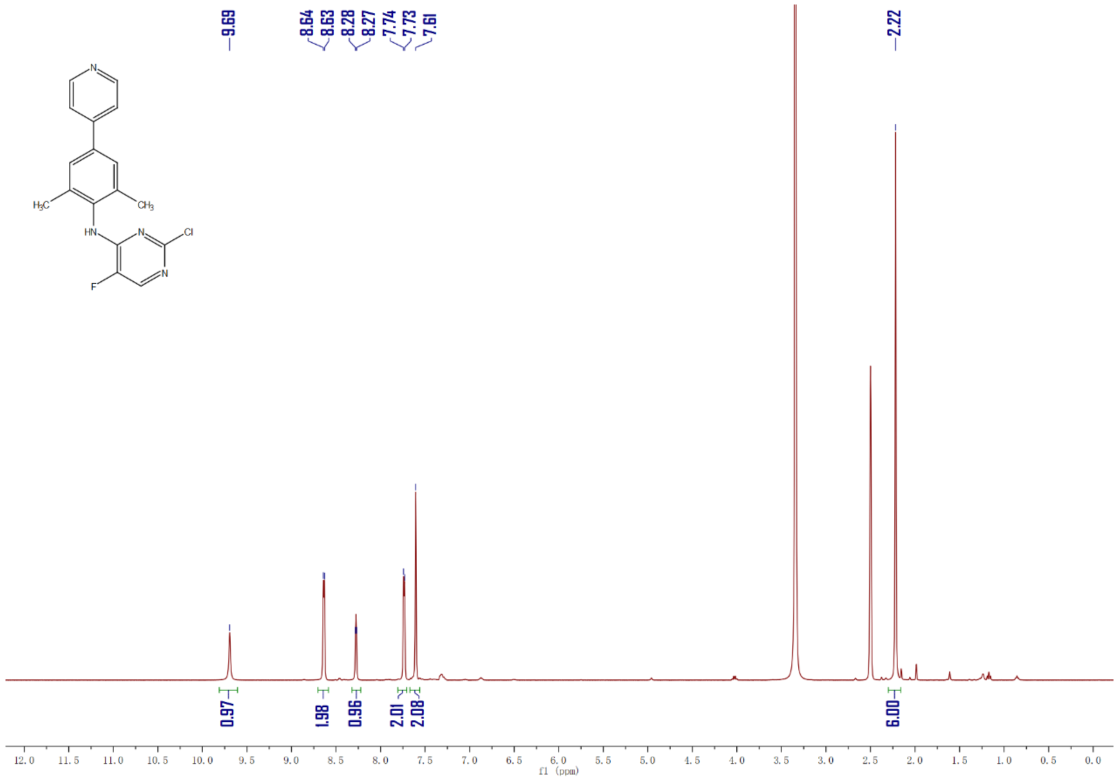

^{13}C NMR

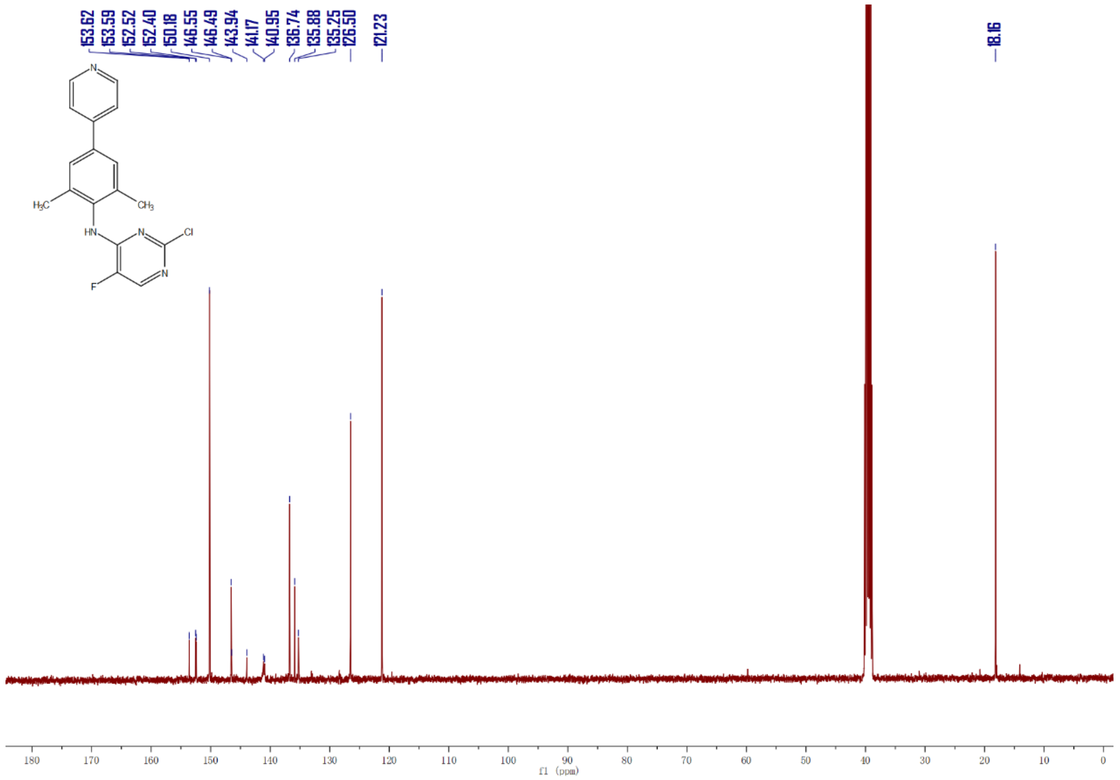

^{19}F NMR

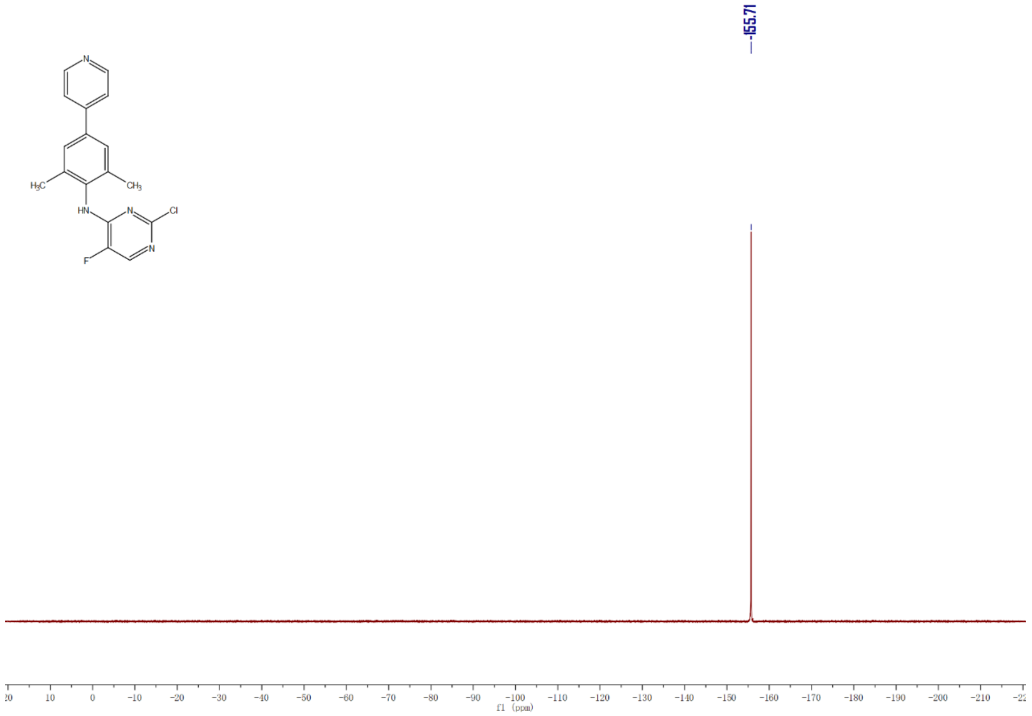

-155.71
HN
Cl
F
N
H3C
CH3
f1 (ppm)

HRMS

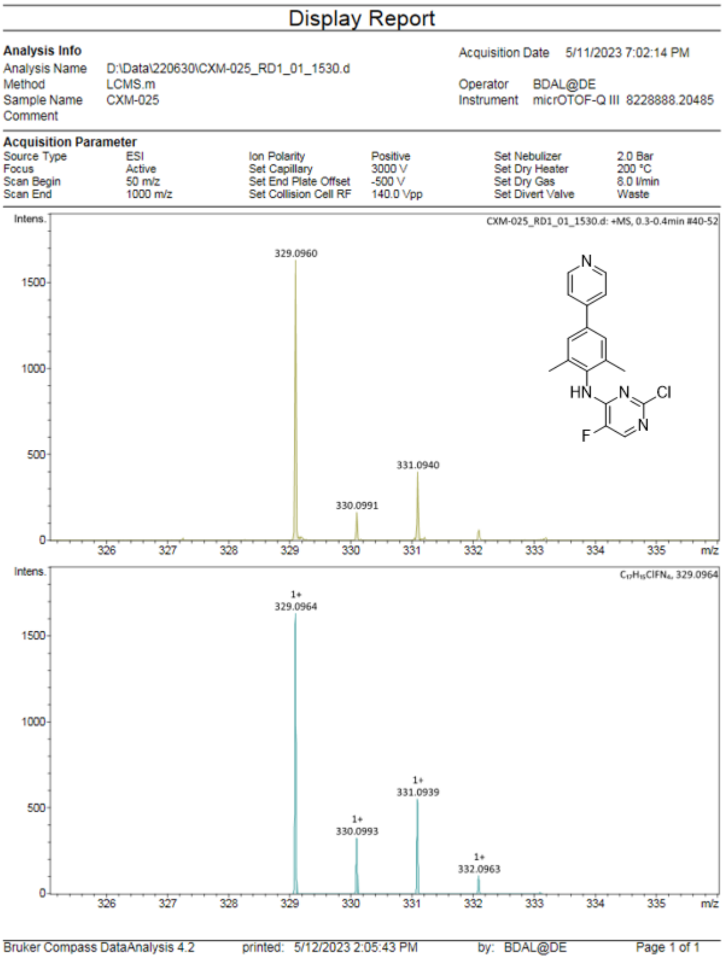

Display Report
Analysis Info
Acquisition Date 5/11/2023 7:02:14 PM
Analysis Name D:\Data\220630\CXM-025_RD1_01_1530.d
Method LCMS.m
Operator BDAL@DE
Sample Name CXM-025
Instrument micrOTOF-Q III 8228888.20485
Comment
Acquisition Parameter
Source Type ESI
Focus Active
Scan Begin 50 m/z
Scan End 1000 m/z
Ion Polarity Positive
Set Capillary 3000 V
Set End Plate Offset -500 V
Set Collision Cell RF 140.0 Vpp
Set Nebulizer 2.0 Bar
Set Dry Heater 200 °C
Set Dry Gas 8.0 l/min
Set Divert Valve Waste
Intens.
CXM-025_RD1_01_1530.d: +MS, 0.3-0.4min #40-52
329.0960
330.0991
331.0940
m/z
HN
N
Cl
F
Intens.
C17H15ClFN4, 329.0964
1+
329.0964
1+
330.0993
1+
331.0939
1+
332.0963
m/z
Bruker Compass DataAnalysis 4.2
printed: 5/12/2023 2:05:43 PM
by: BDAL@DE
Page 1 of 1

^{1}H NMR, ^{13}C NMR, ^{19}F NMR, HRMS spectra of 10m

^{1}H NMR

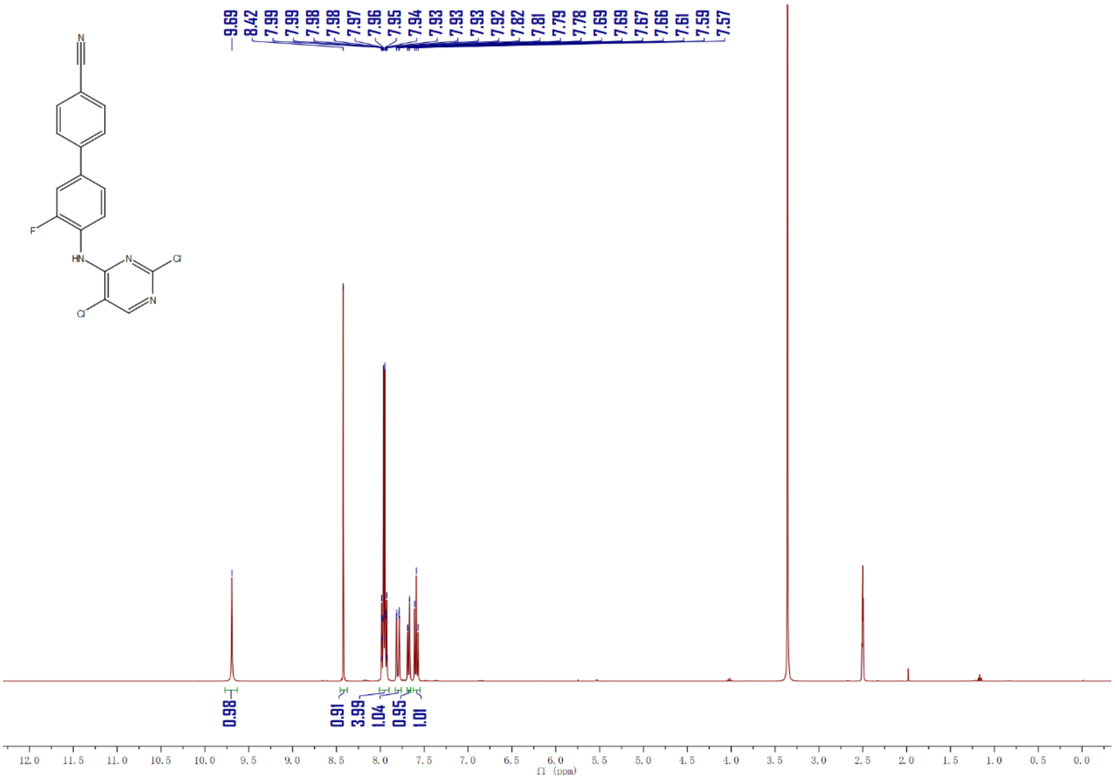

^{13}C NMR

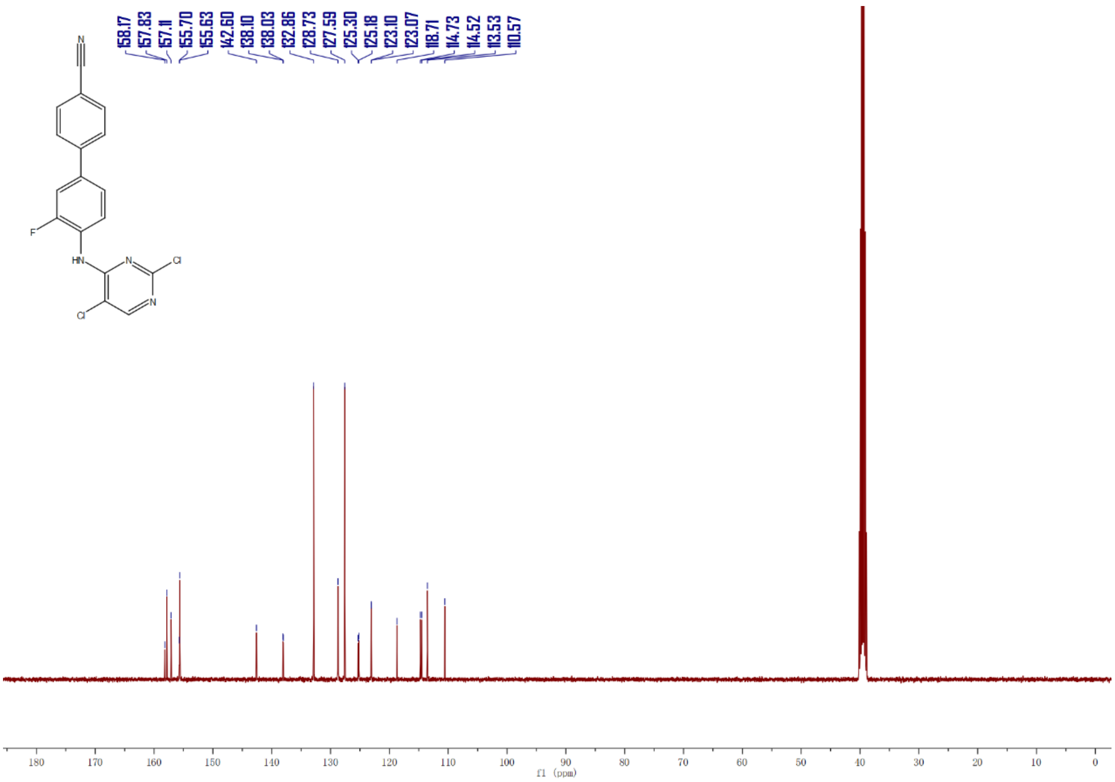

^{19}F NMR

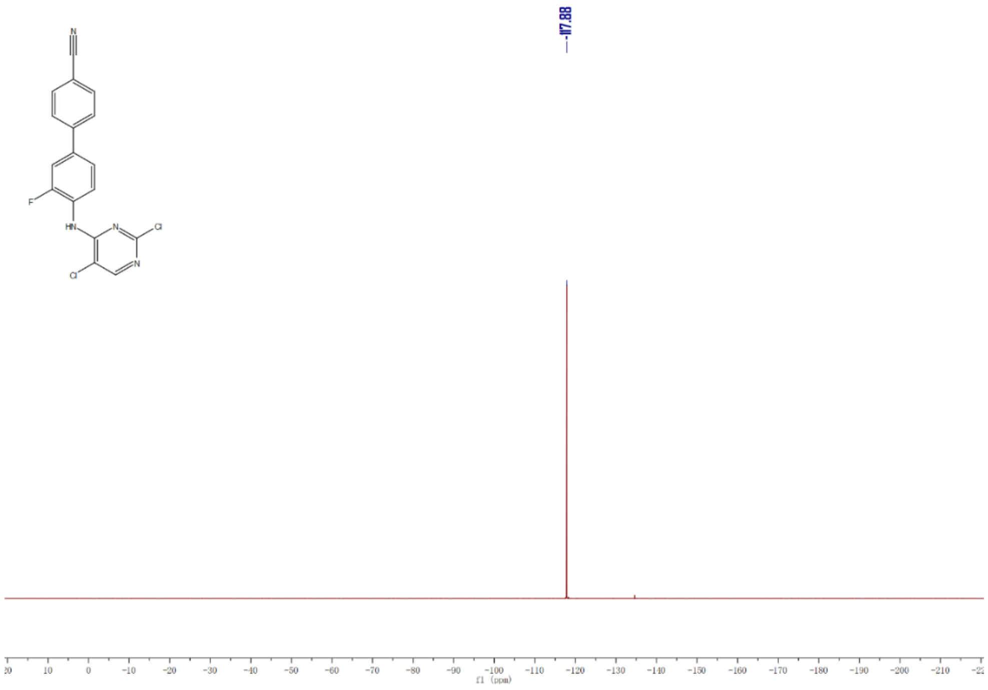

-117.88
f1 (ppm)

HRMS

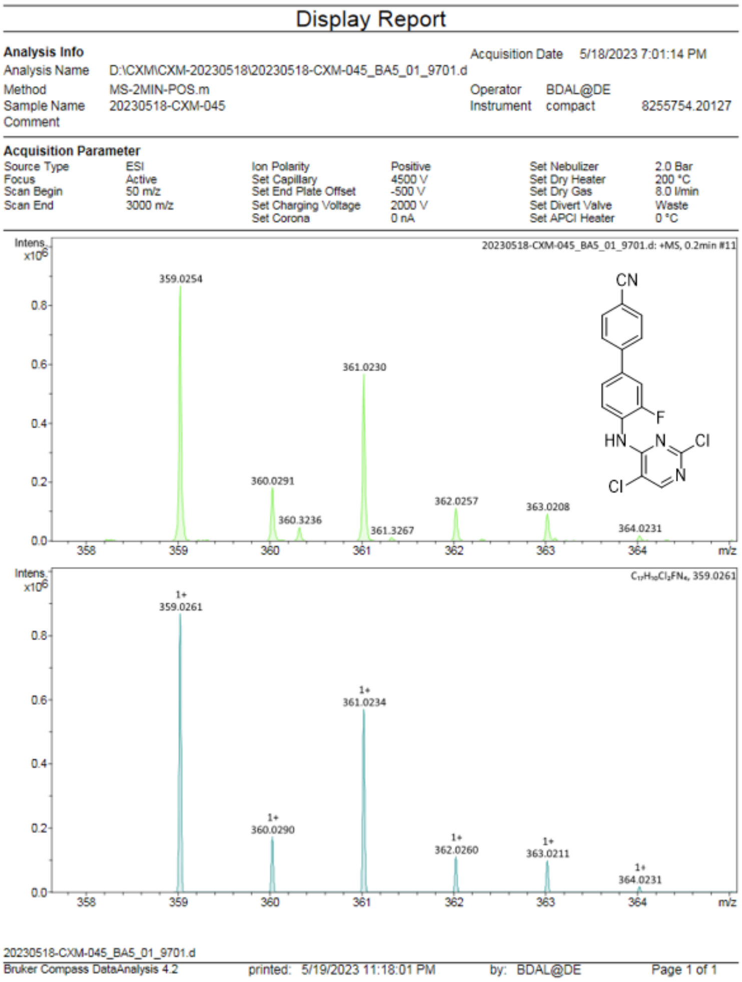

Display Report
Analysis Info
Analysis Name D:\CXM\CXM-20230518\20230518-CXM-045_BA5_01_9701.d
Method MS-2MIN-POS.m
Sample Name 20230518-CXM-045
Comment
Acquisition Date 5/18/2023 7:01:14 PM
Operator BDAL@DE
Instrument compact 8255754.20127
Acquisition Parameter
Source Type ESI
Focus Active
Scan Begin 50 m/z
Scan End 3000 m/z
Ion Polarity Positive
Set Capillary 4500 V
Set End Plate Offset -500 V
Set Charging Voltage 2000 V
Set Corona 0 nA
Set Nebulizer 2.0 Bar
Set Dry Heater 200 °C
Set Dry Gas 8.0 l/min
Set Divert Valve Waste
Set APCI Heater 0 °C
20230518-CXM-045_BA5_01_9701.d: +MS, 0.2min #11
359.0254
360.0291
360.3236
361.0230
361.3267
362.0257
363.0208
364.0231
C17H10Cl2FN4, 359.0261
359.0261
360.0290
361.0234
362.0260
363.0211
364.0231
20230518-CXM-045_BA5_01_9701.d
Bruker Compass DataAnalysis 4.2
printed: 5/19/2023 11:18:01 PM
by: BDAL@DE
Page 1 of 1

^{1}H NMR, ^{13}C NMR, HRMS spectra of 10n

^{1}H NMR

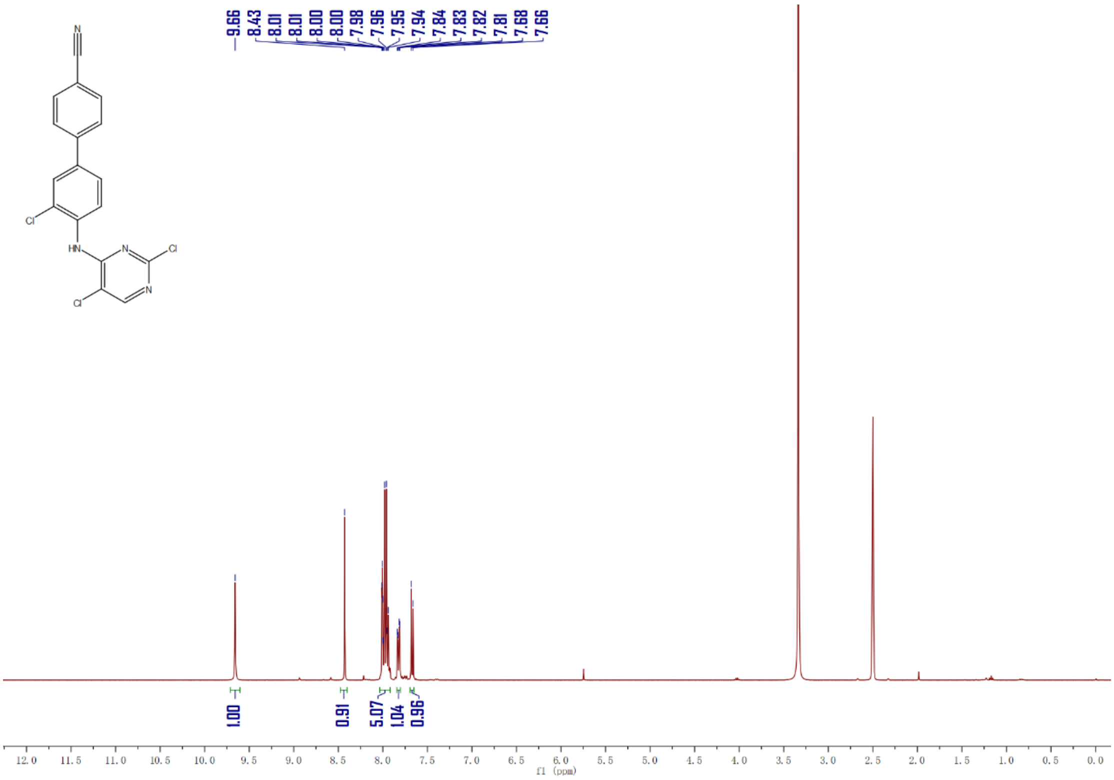

^{13}C NMR

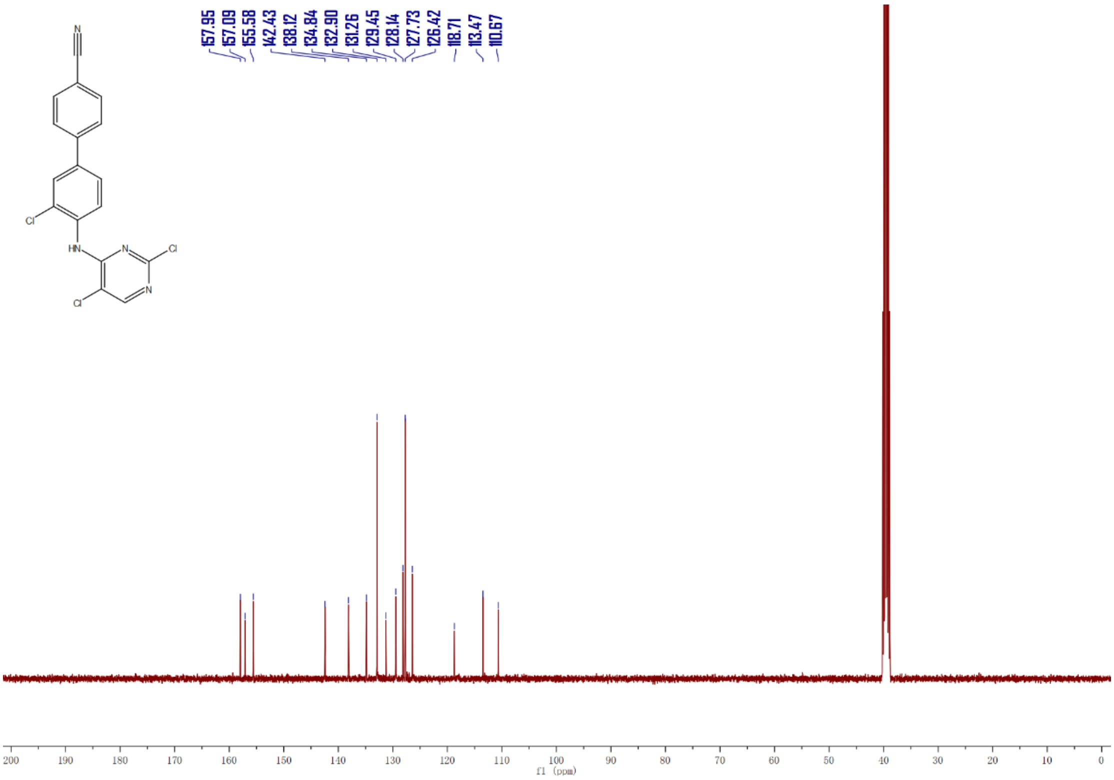

HRMS

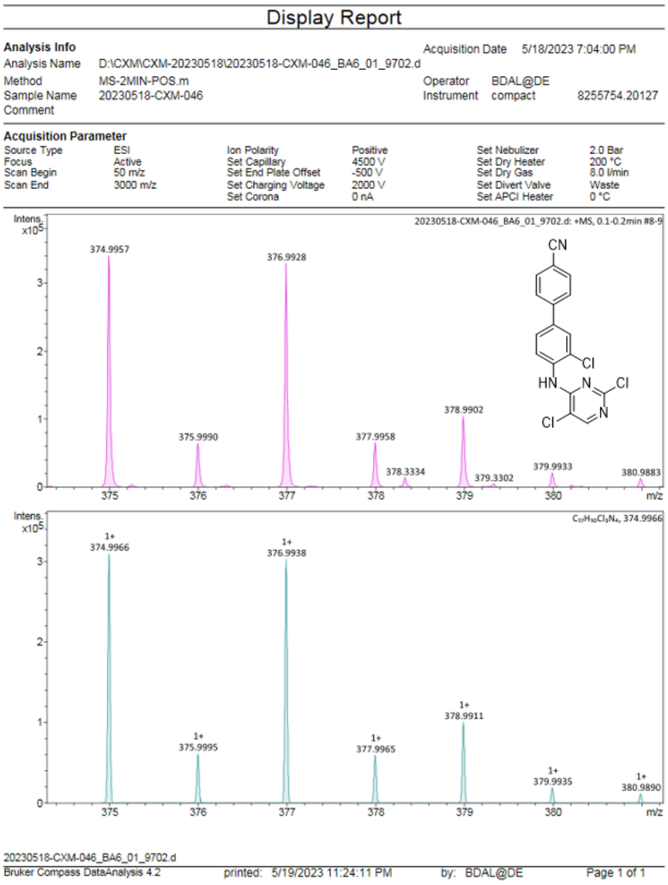

^{1}H NMR, ^{13}C NMR, HRMS spectra of 10o

^{1}H NMR

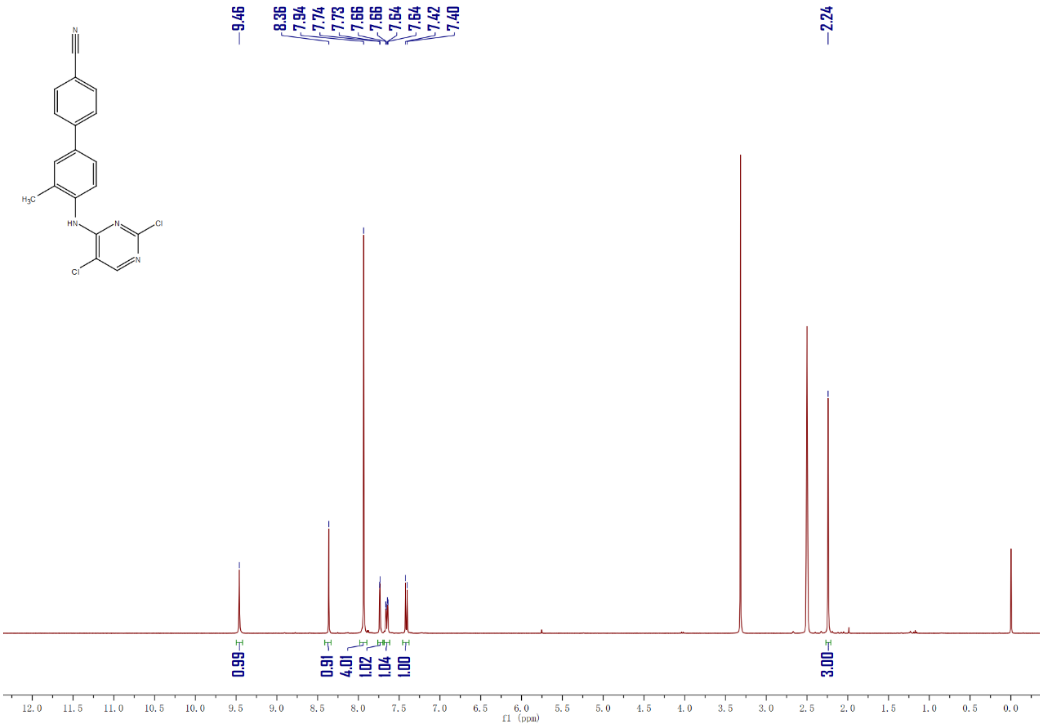

^{13}C NMR

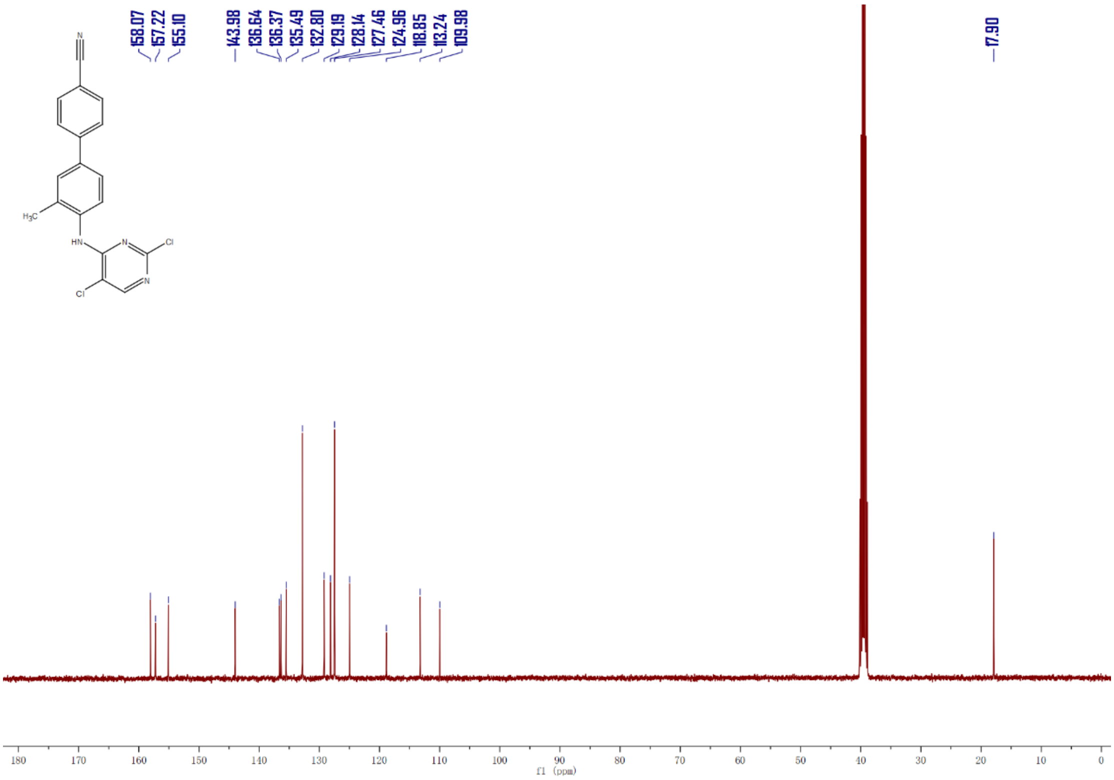
158.07
157.22
155.10
143.98
136.64
136.37
135.49
132.80
129.19
128.14
127.46
124.96
118.85
113.24
109.98
17.90
f1 (ppm)

HRMS

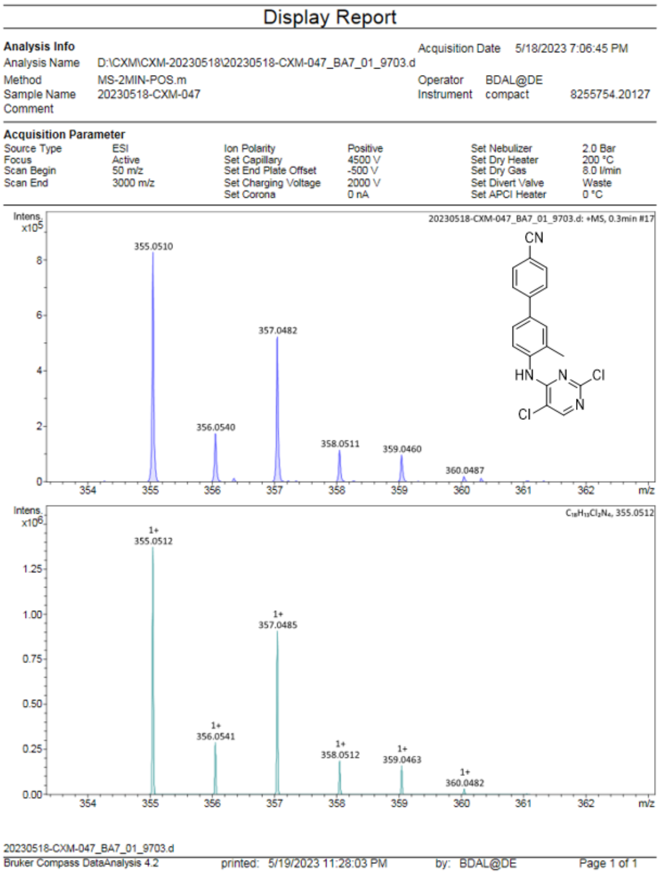
Display Report
Analysis Info
Analysis Name D:\CXM\CXM-20230518\20230518-CXM-047_BA7_01_9703.d
Method MS-2MIN-POS.m
Sample Name 20230518-CXM-047
Comment
Acquisition Date 5/18/2023 7:06:45 PM
Operator BDAL@DE
Instrument compact 8255754.20127
Acquisition Parameter
Source Type ESI
Focus Active
Scan Begin 50 m/z
Scan End 3000 m/z
Ion Polarity Positive
Set Capillary 4500 V
Set End Plate Offset -500 V
Set Charging Voltage 2000 V
Set Corona 0 nA
Set Nebulizer 2.0 Bar
Set Dry Heater 200 °C
Set Dry Gas 8.0 l/min
Set Divert Valve Waste
Set APCI Heater 0 °C
20230518-CXM-047_BA7_01_9703.d: +MS, 0.3min #17
355.0510
356.0540
357.0482
358.0511
359.0460
360.0487
355.0512
356.0541
357.0485
358.0512
359.0463
360.0482
20230518-CXM-047_BA7_01_9703.d
Bruker Compass DataAnalysis 4.2
printed: 5/19/2023 11:28:03 PM
by: BDAL@DE
Page 1 of 1

^{1}H NMR, ^{13}C NMR, HRMS spectra of 10p

^{1}H NMR

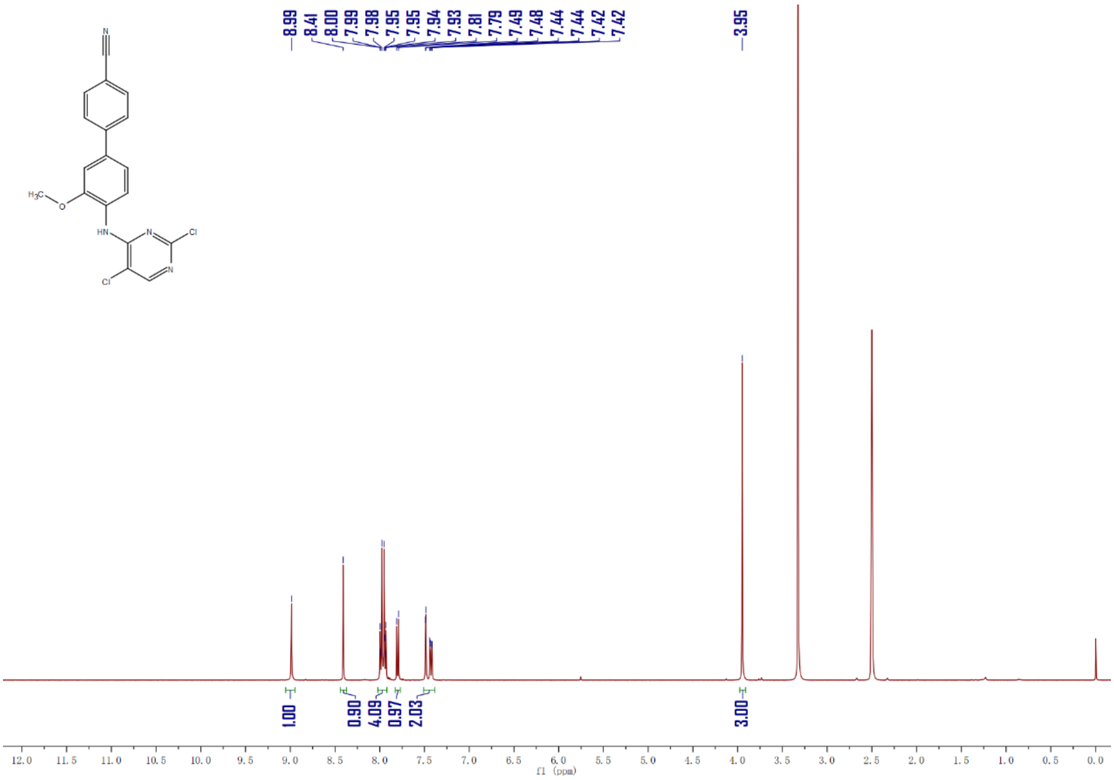

^{13}C NMR

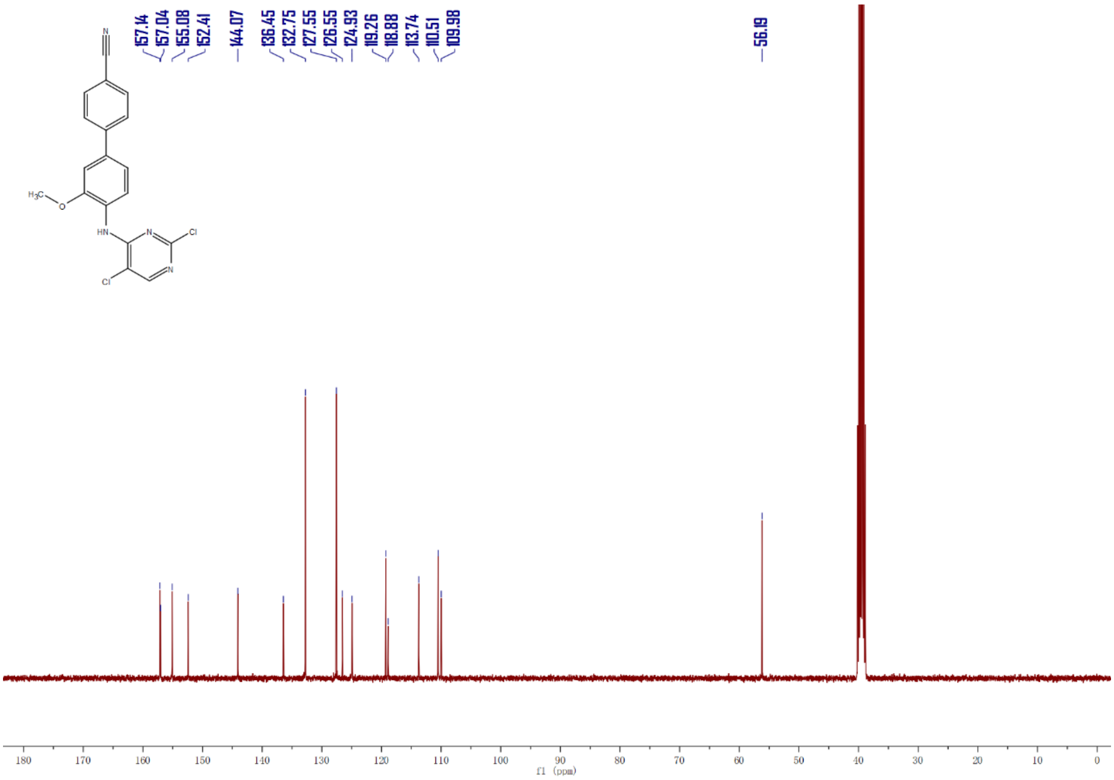

HRMS

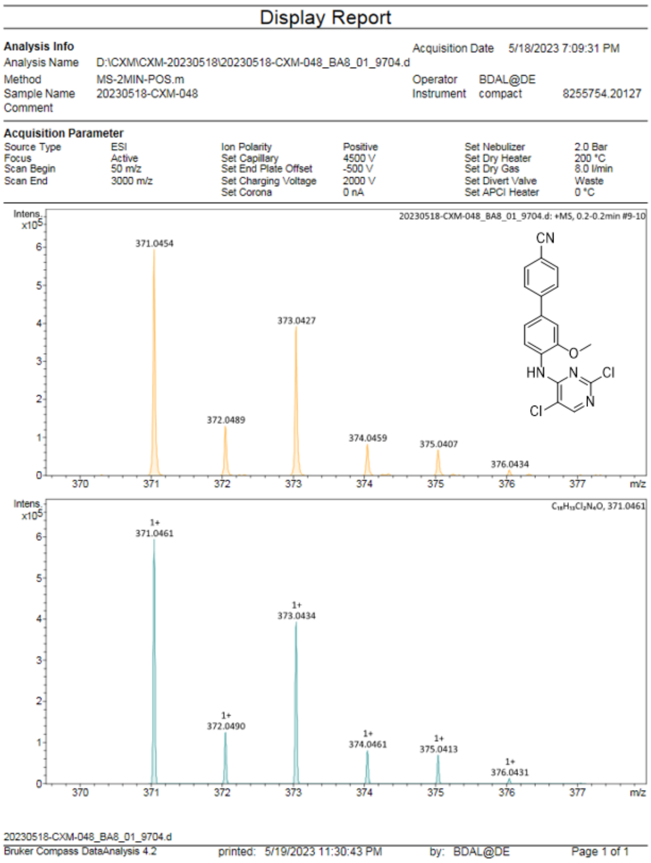

^{1}H NMR, ^{13}C NMR, ^{19}F NMR, HRMS spectra of 10q

^{1}H NMR

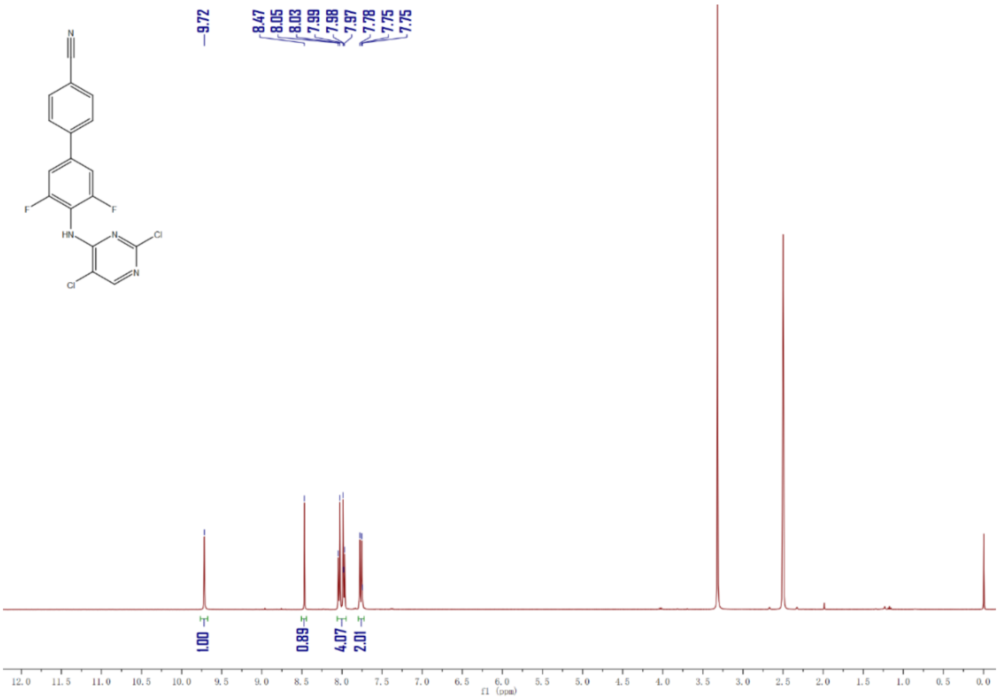

^{13}C NMR

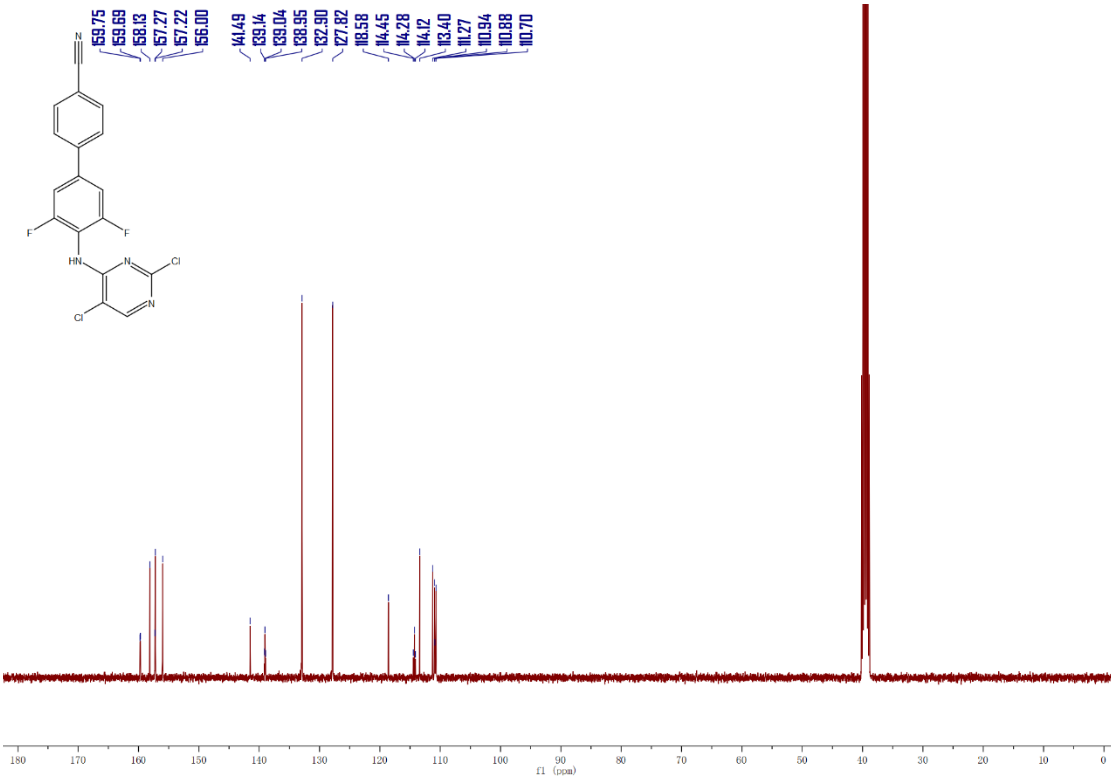

^{19}F NMR

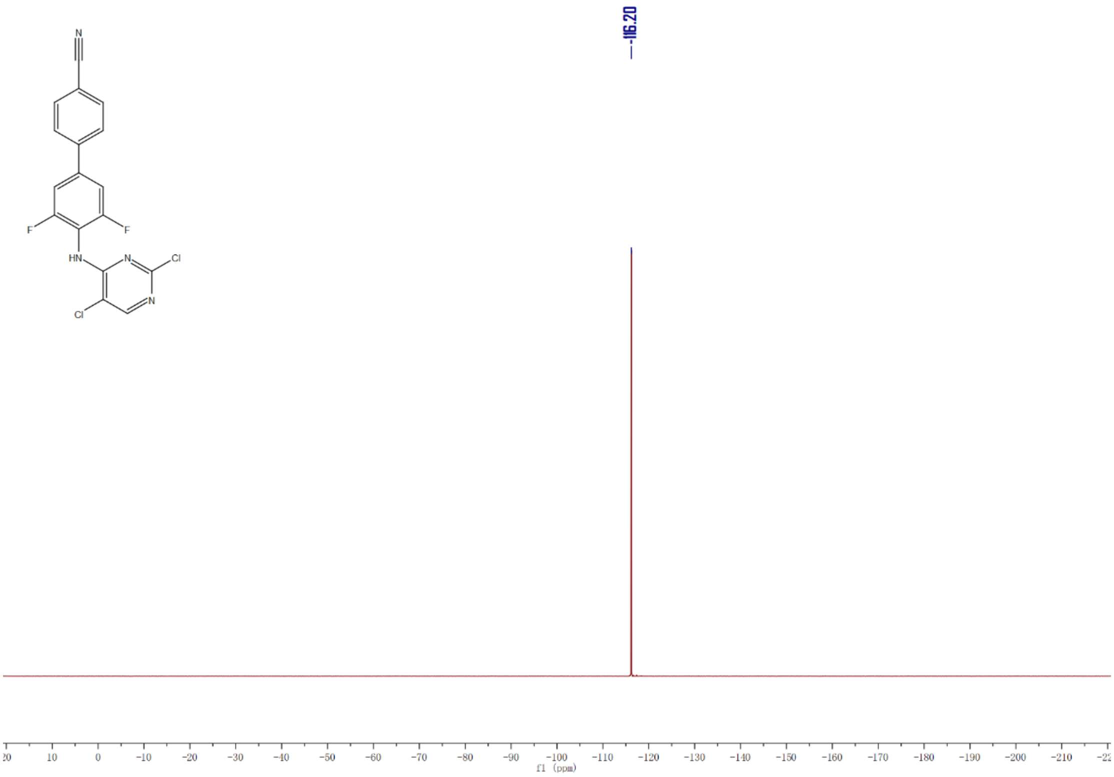

HRMS

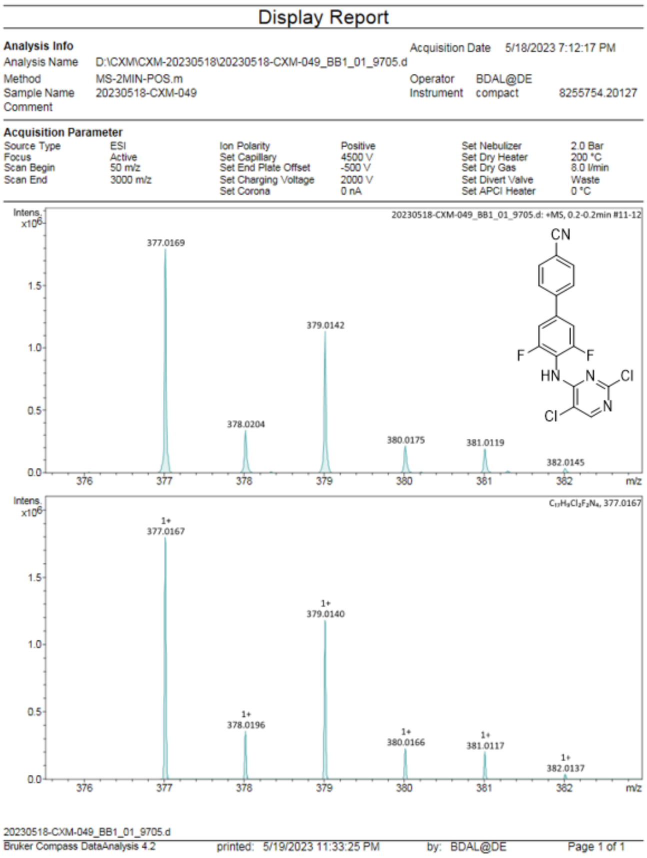

^{1}H NMR, ^{13}C NMR, HRMS spectra of 10r

^{1}H NMR

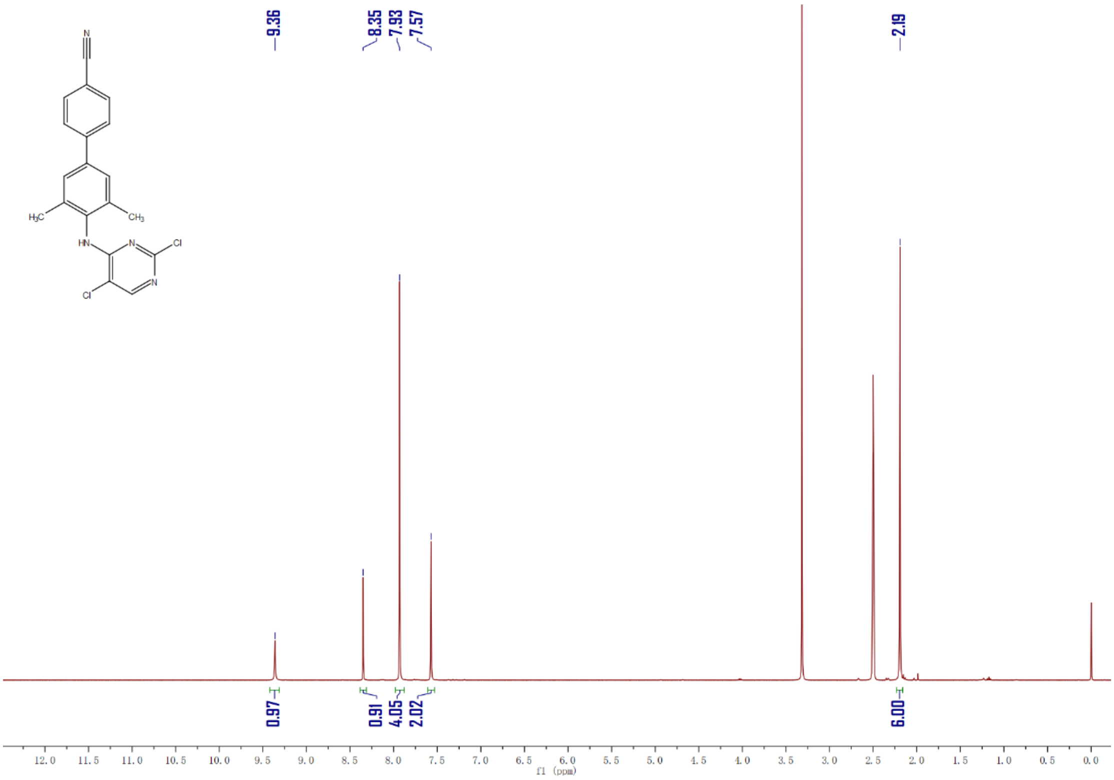

^{13}C NMR

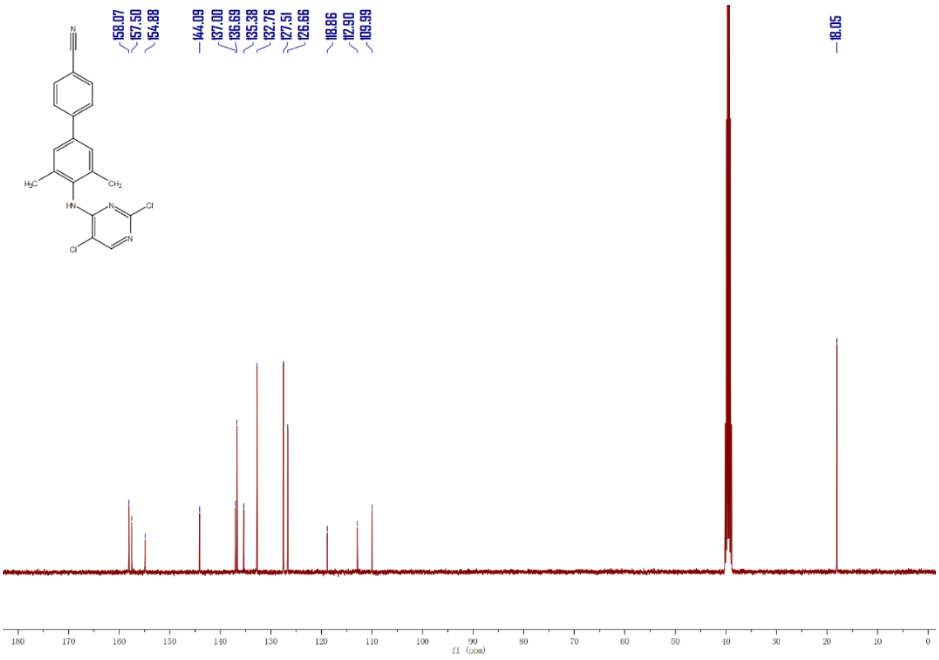

HRMS

Display Report

Analysis Info

Analysis Name	D:\CXM\CXM-20230518\20230518-CXM-050_BB2_01_9706.d	Acquisition Date	5/18/2023 7:15:03 PM
Method	MS-2MIN-POS.m	Operator	BDAL@DE
Sample Name	20230518-CXM-050	Instrument	compact 8255754.20127
Comment			

Acquisition Parameter

Source Type	ESI	Ion Polarity	Positive	Set Nebulizer	2.0 Bar
Focus	Active	Set Capillary	4500 V	Set Dry Heater	200 °C
Scan Begin	50 m/z	Set End Plate Offset	-500 V	Set Dry Gas	8.0 l/min
Scan End	3000 m/z	Set Charging Voltage	2000 V	Set Divert Valve	Waste
		Set Corona	0 nA	Set APCI Heater	0 °C

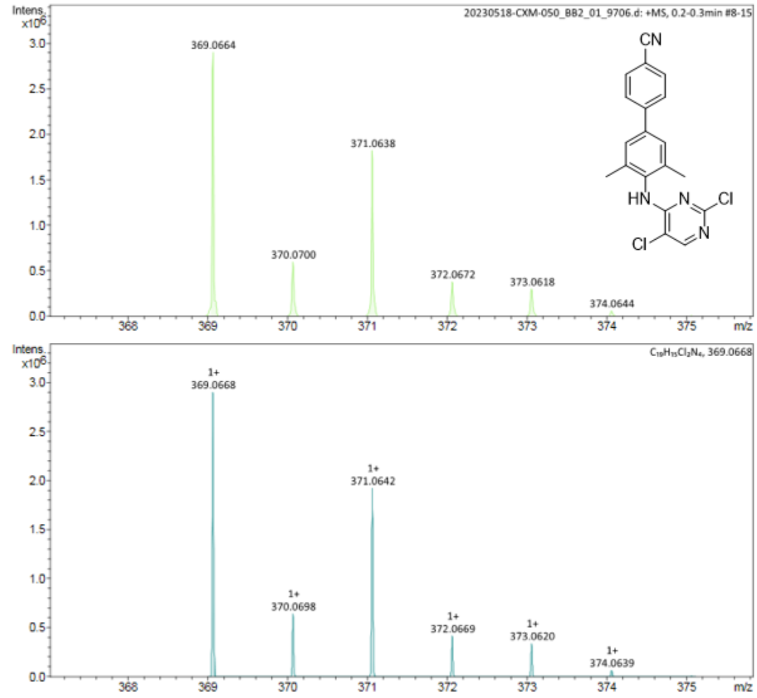

20230518-CXM-050_BB2_01_9706.d

Bruker Compass DataAnalysis 4.2 printed: 5/19/2023 11:35:01 PM by: BDAL@DE Page 1 of 1

^{1}H NMR, ^{13}C NMR, ^{19}F NMR, HRMS spectra of 10s

^{1}H NMR

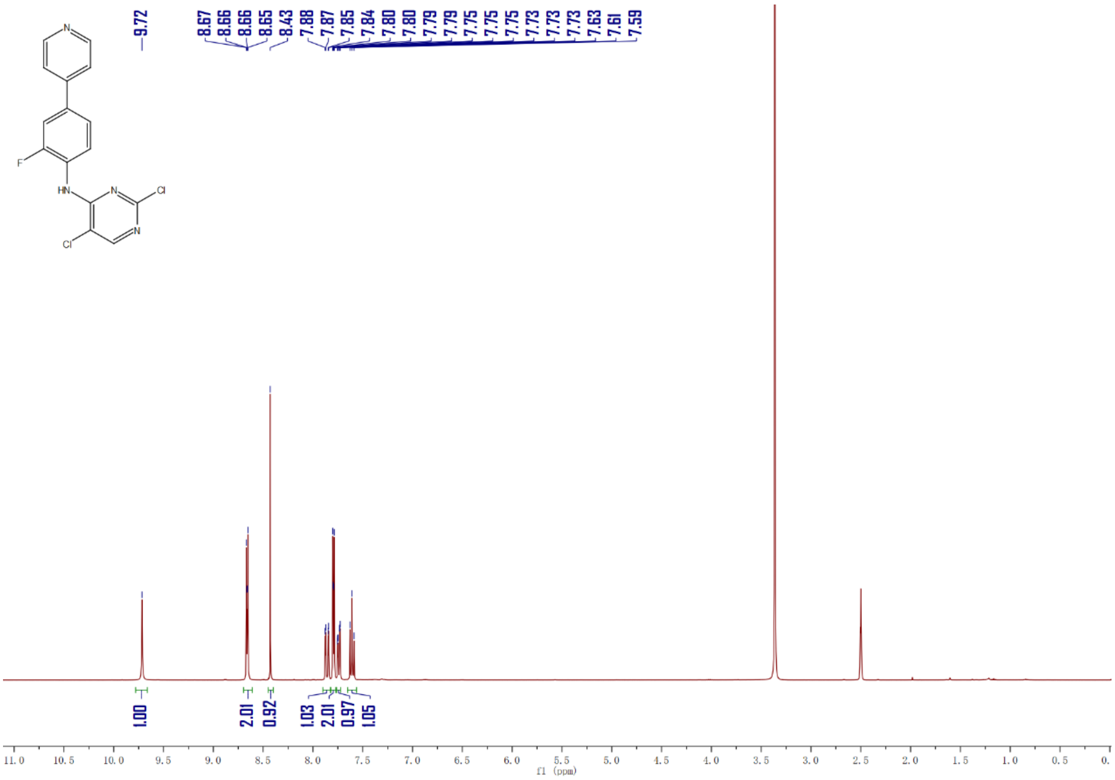

^{13}C NMR

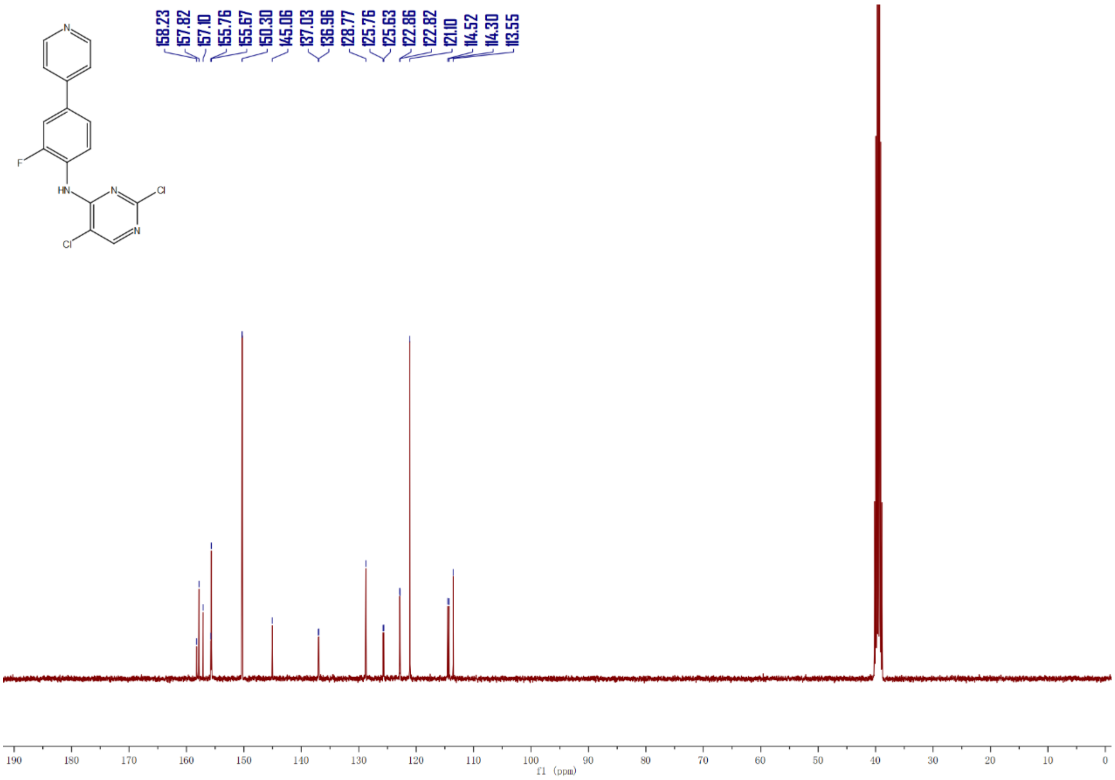

^{19}F NMR

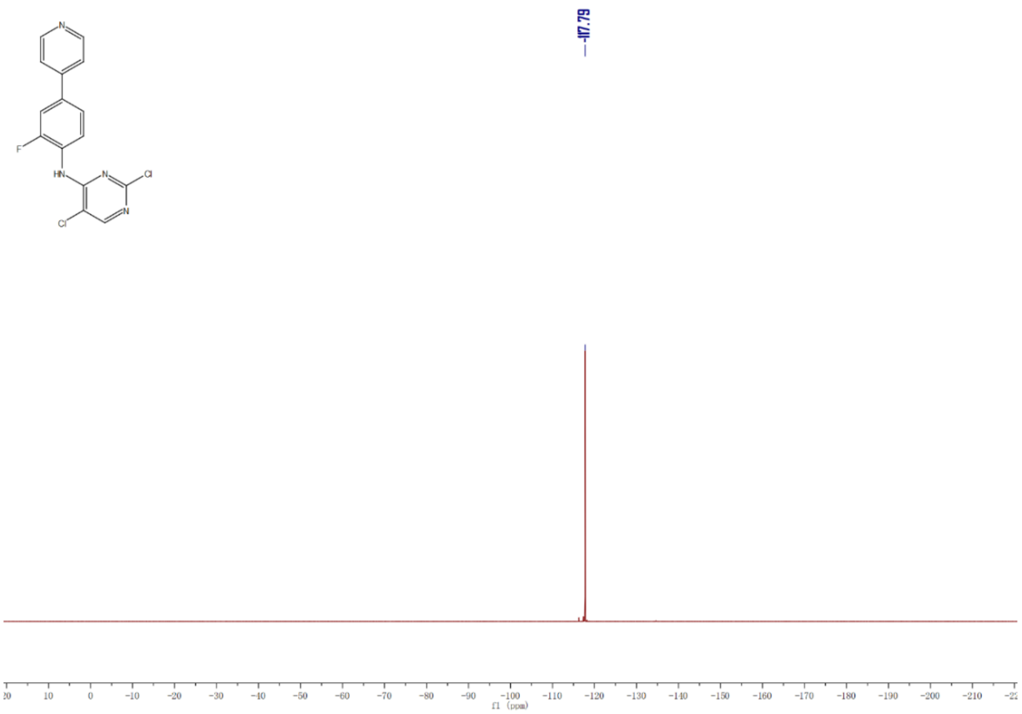

HRMS

Display Report

Analysis Info

Analysis Name	D:\CXM\CXM-20230518\20230518-CXM-054_BB6_01_9710.d	Acquisition Date	5/18/2023 7:26:05 PM
Method	MS-2MIN-POS.m	Operator	BDAL@DE
Sample Name	20230518-CXM-054	Instrument	compact 8255754.20127
Comment			

Acquisition Parameter

Source Type	ESI	Ion Polarity	Positive	Set Nebulizer	2.0 Bar
Focus	Active	Set Capillary	4500 V	Set Dry Heater	200 °C
Scan Begin	50 m/z	Set End Plate Offset	-500 V	Set Dry Gas	8.0 l/min
Scan End	3000 m/z	Set Charging Voltage	2000 V	Set Divert Valve	Waste
		Set Corona	0 nA	Set APCI Heater	0 °C

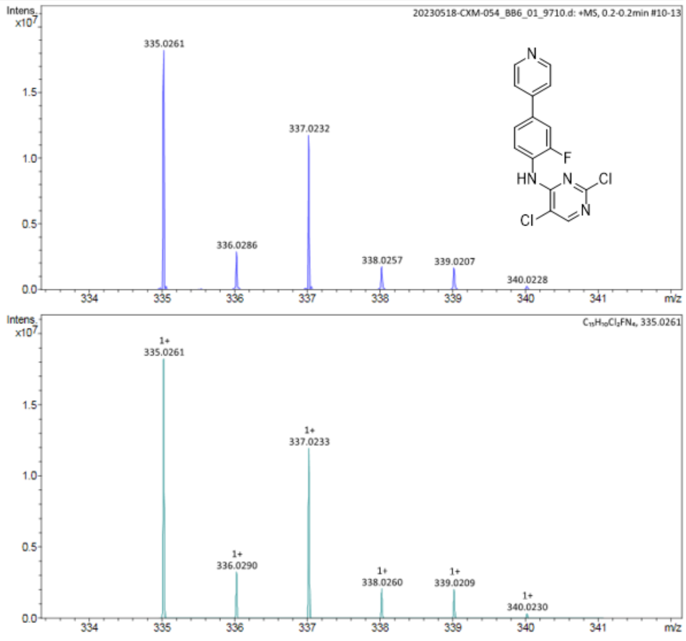

20230518-CXM-054_BB6_01_9710.d
Bruker Compass DataAnalysis 4.2 printed: 5/19/2023 11:44:22 PM by: BDAL@DE Page 1 of 1

^{1}H NMR, ^{13}C NMR, HRMS spectra of 10t

^{1}H NMR

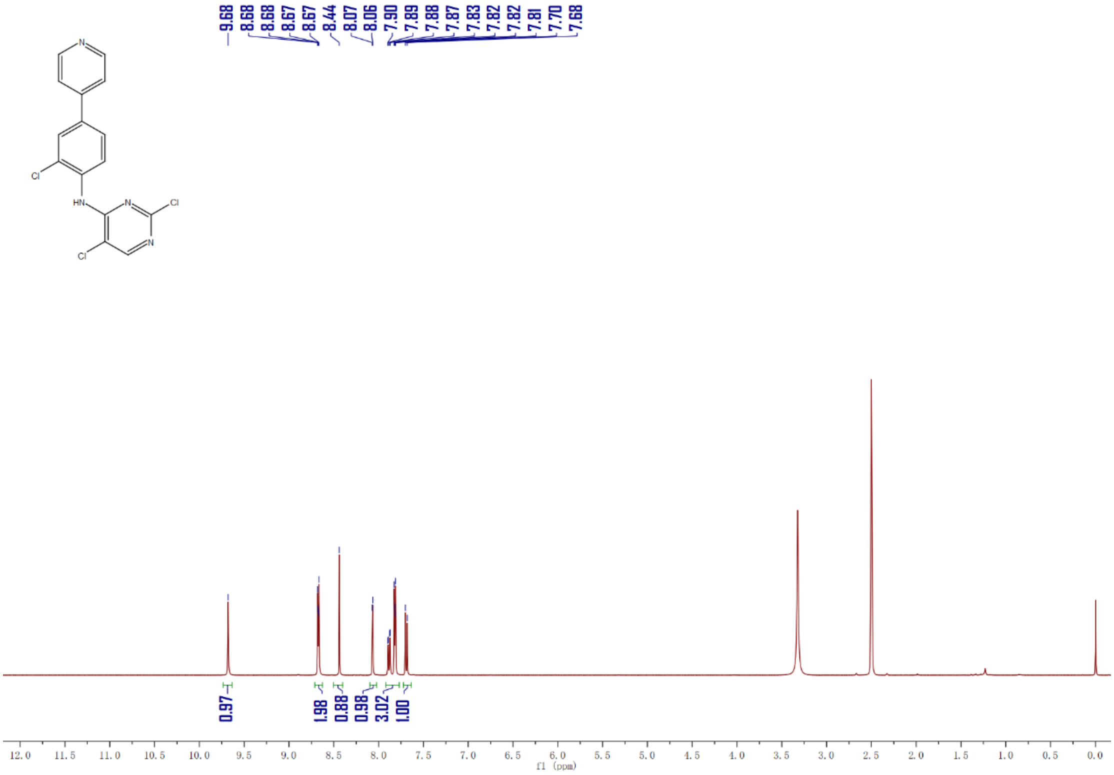

^{13}C NMR

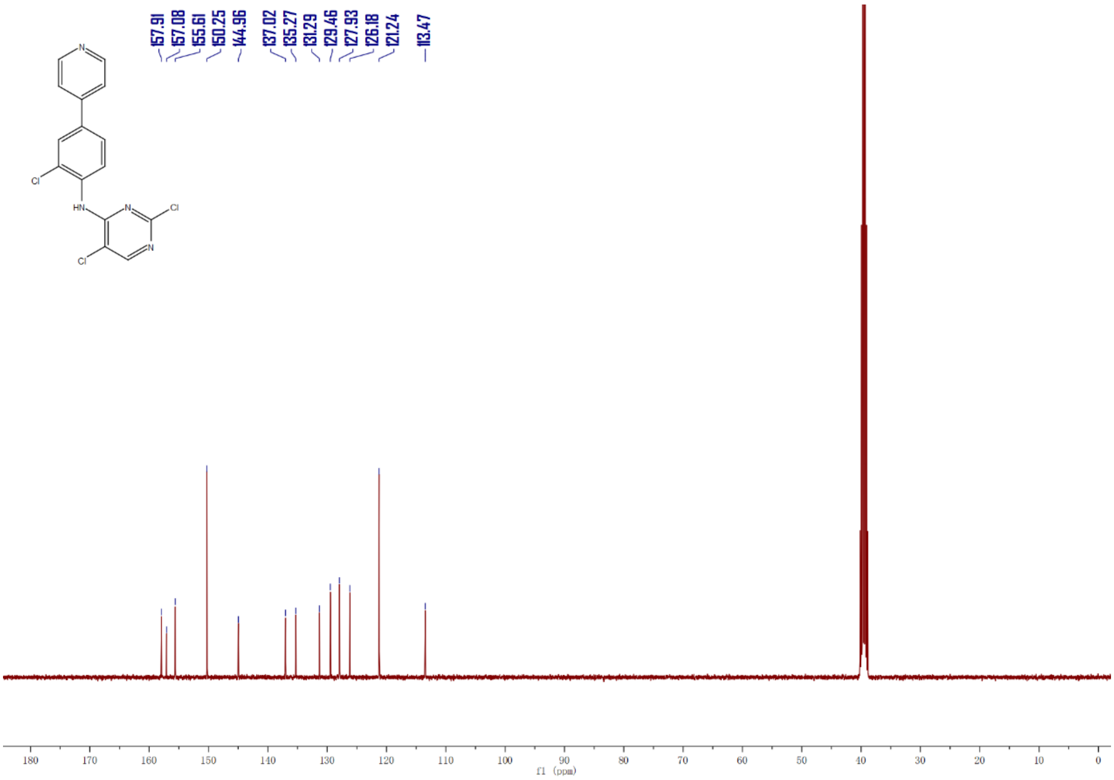

HRMS

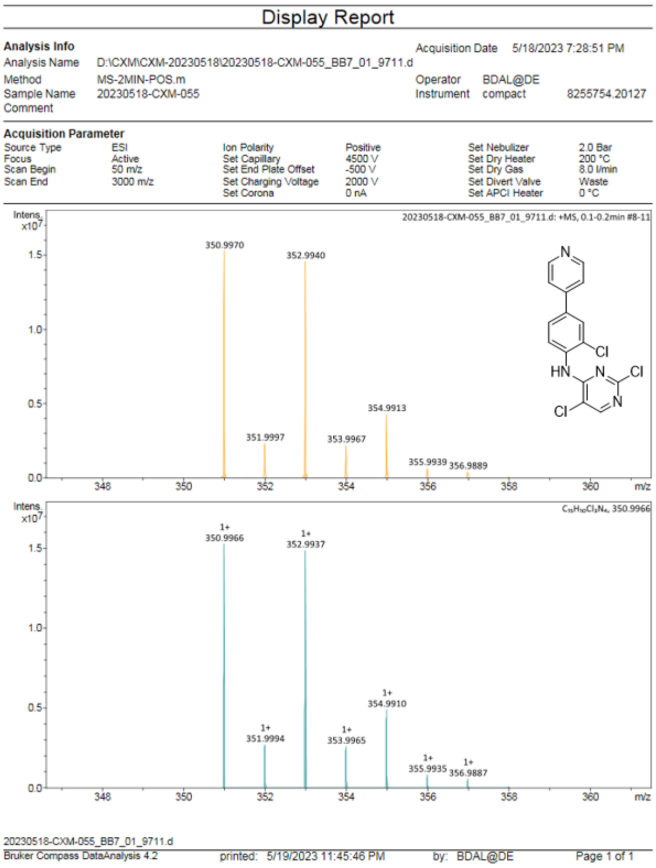

^{1}H NMR, ^{13}C NMR, HRMS spectra of 10u

^{1}H NMR

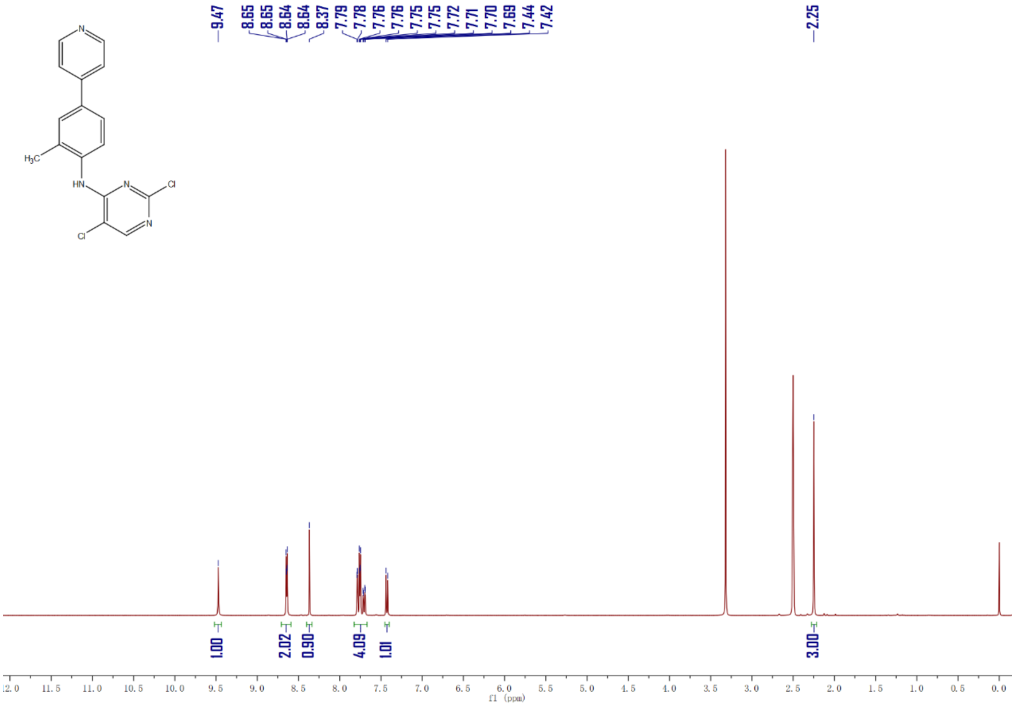

^{13}C NMR

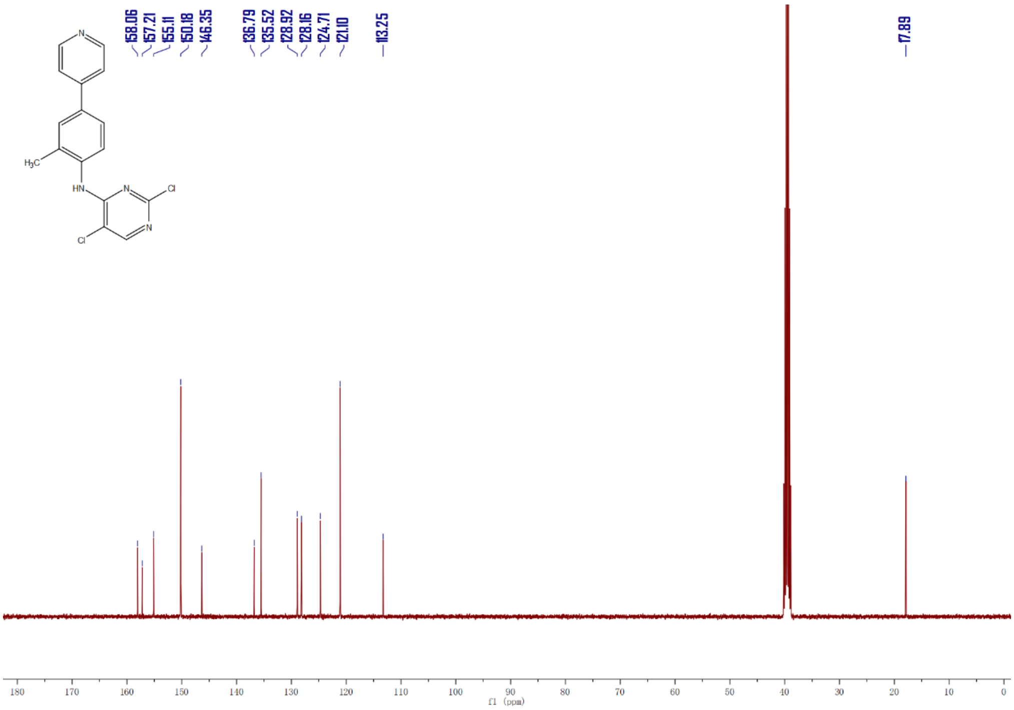

158.06
157.21
155.11
150.18
146.35
136.79
135.52
128.92
128.16
124.71
121.10
113.25
17.89
N
H3C
HN
N
Cl
Cl
N
180
170
160
150
140
130
120
110
100
90
80
70
60
50
40
30
20
10
0
f1 (ppm)

HRMS

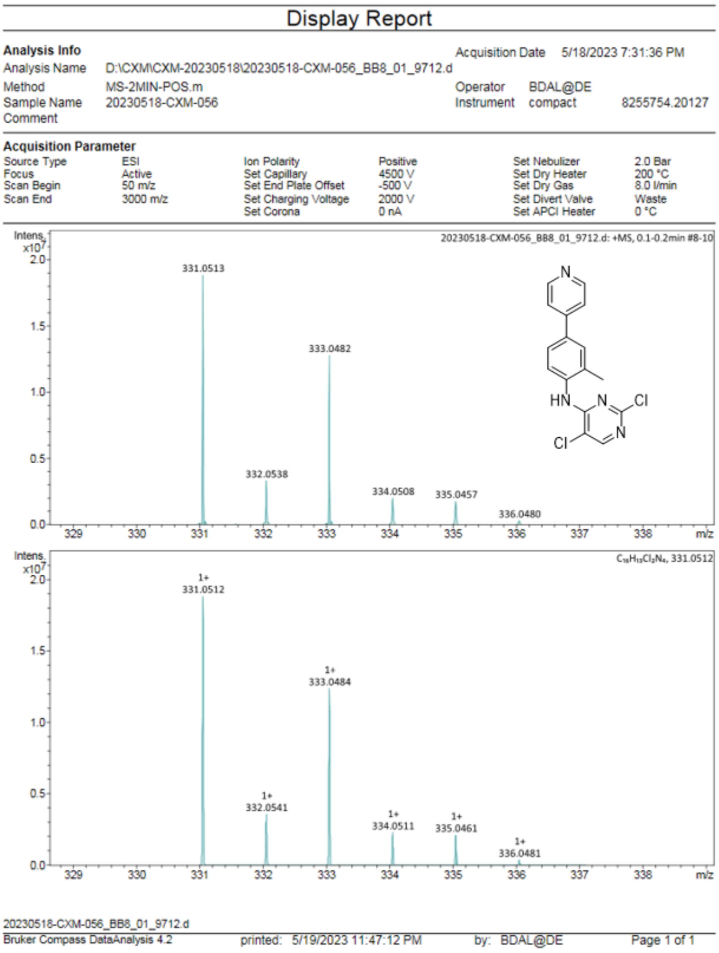

Display Report
Analysis Info
Acquisition Date 5/18/2023 7:31:36 PM
Analysis Name D:\CXM\CXM-20230518\20230518-CXM-056_BB8_01_9712.d
Method MS-2MIN-POS.m
Operator BDAL@DE
Sample Name 20230518-CXM-056
Instrument compact 8255754.20127
Comment
Acquisition Parameter
Source Type ESI
Focus Active
Scan Begin 50 m/z
Scan End 3000 m/z
Ion Polarity Positive
Set Capillary 4500 V
Set End Plate Offset -500 V
Set Charging Voltage 2000 V
Set Corona 0 nA
Set Nebulizer 2.0 Bar
Set Dry Heater 200 °C
Set Dry Gas 8.0 l/min
Set Divert Valve Waste
Set APCI Heater 0 °C
Intens. x10^7
20230518-CXM-056_BB8_01_9712.d: +MS, 0.1-0.2min #8-10
331.0513
333.0482
332.0538
334.0508
335.0457
336.0480
N
HN
N
Cl
Cl
N
m/z
C16H13Cl2N4, 331.0512
1+ 331.0512
1+ 333.0484
1+ 332.0541
1+ 334.0511
1+ 335.0461
1+ 336.0481
20230518-CXM-056_BB8_01_9712.d
Bruker Compass DataAnalysis 4.2
printed: 5/19/2023 11:47:12 PM
by: BDAL@DE
Page 1 of 1

^{1}H NMR, ^{13}C NMR, HRMS spectra of 10v

^{1}H NMR

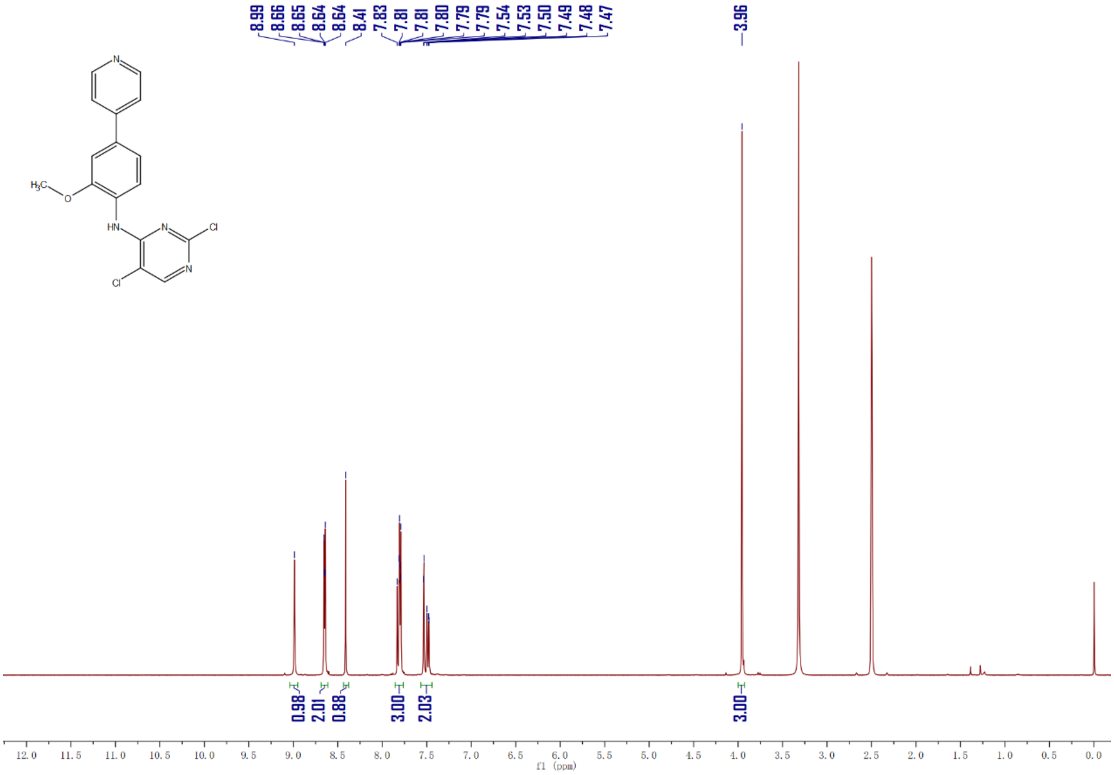

^{13}C NMR

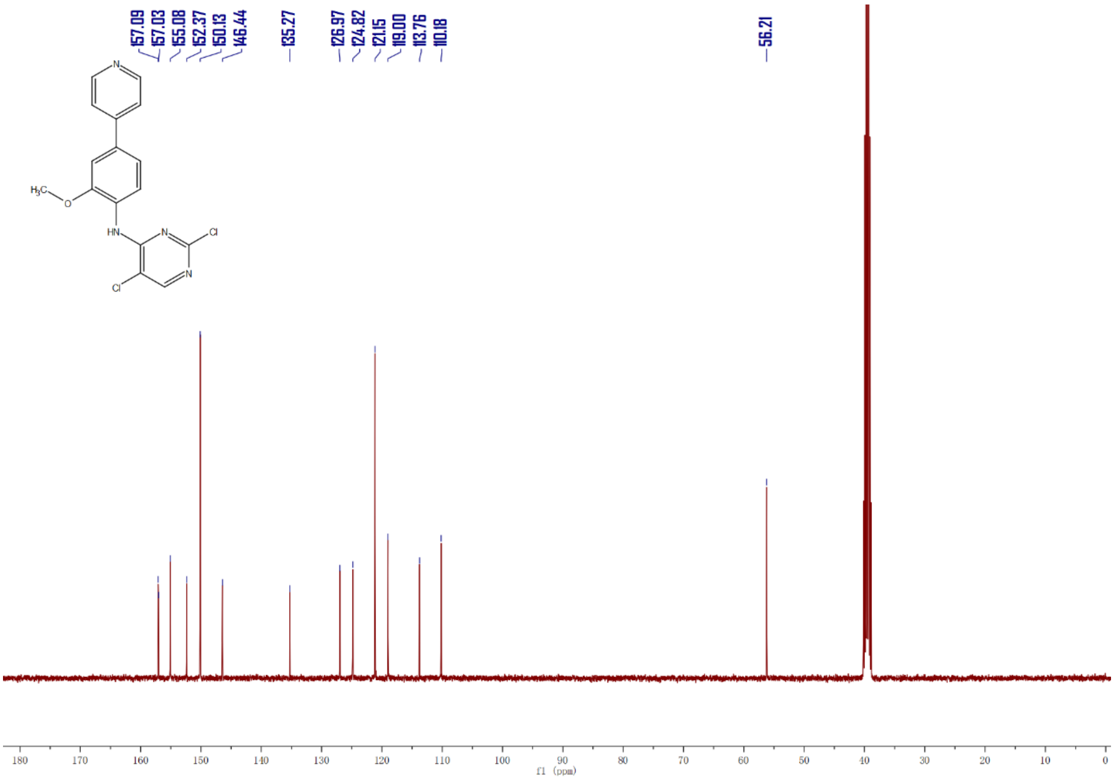

HRMS

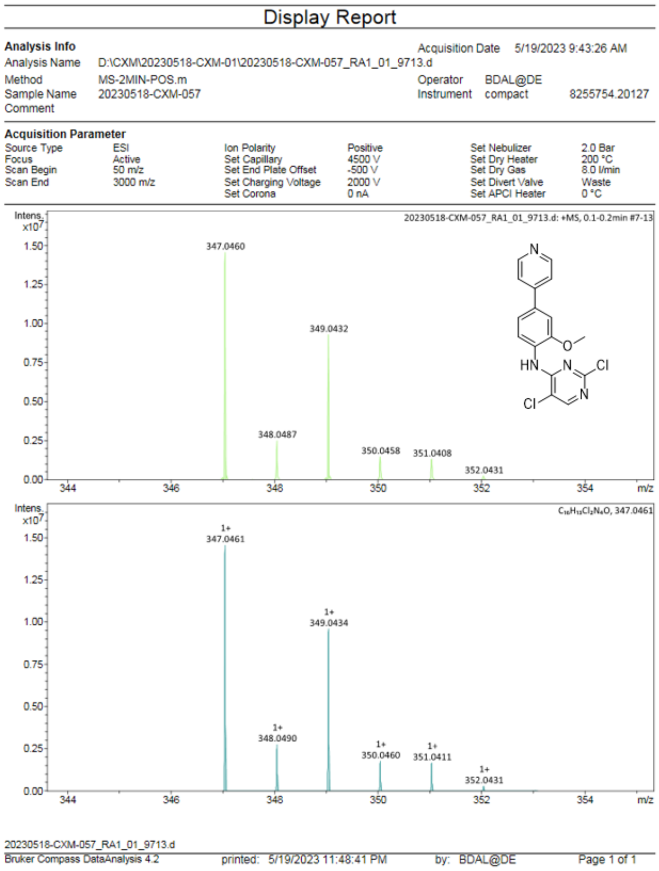

^{1}H NMR, ^{13}C NMR, ^{19}F NMR, HRMS spectra of 10w

^{1}H NMR

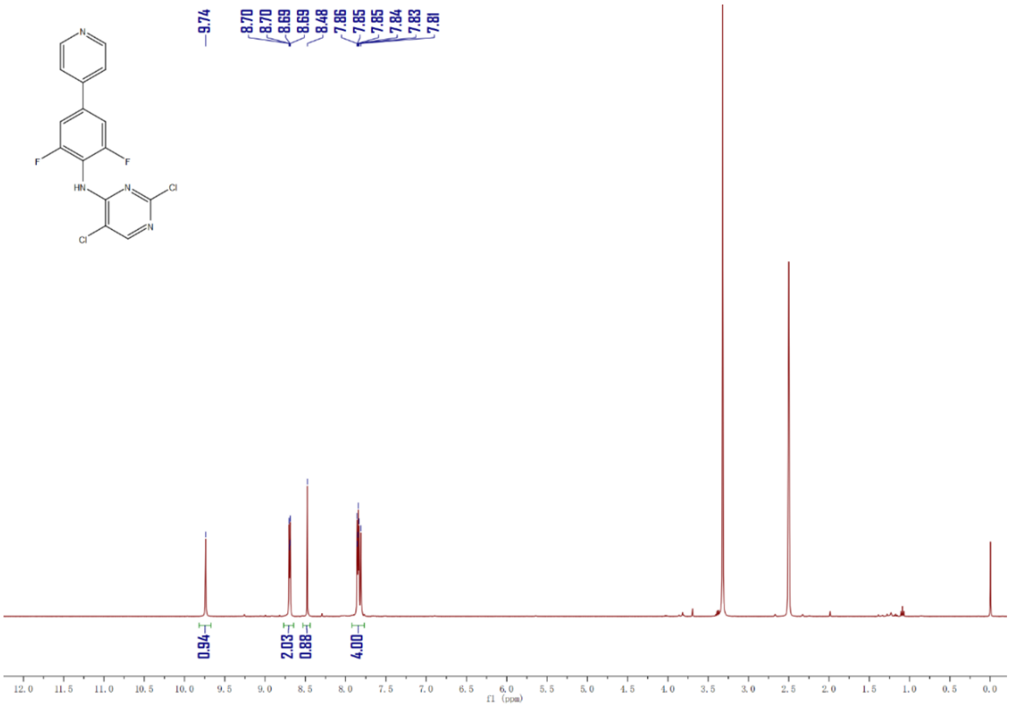

^{13}C NMR

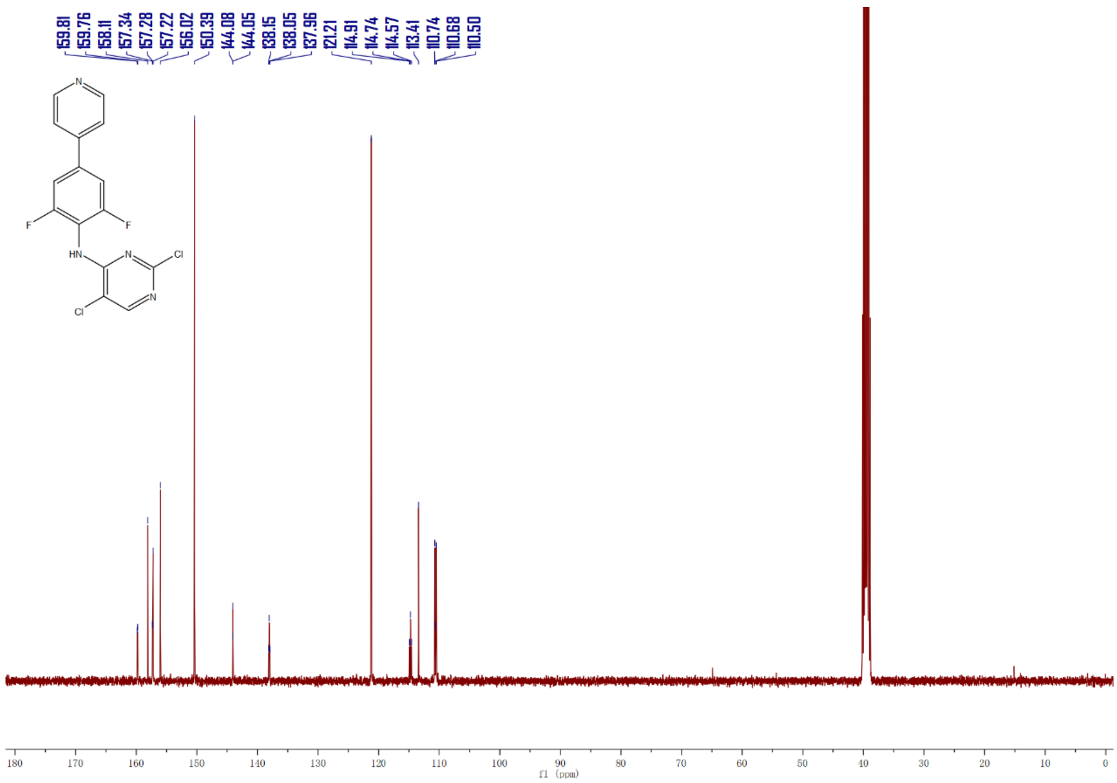

^{19}F NMR

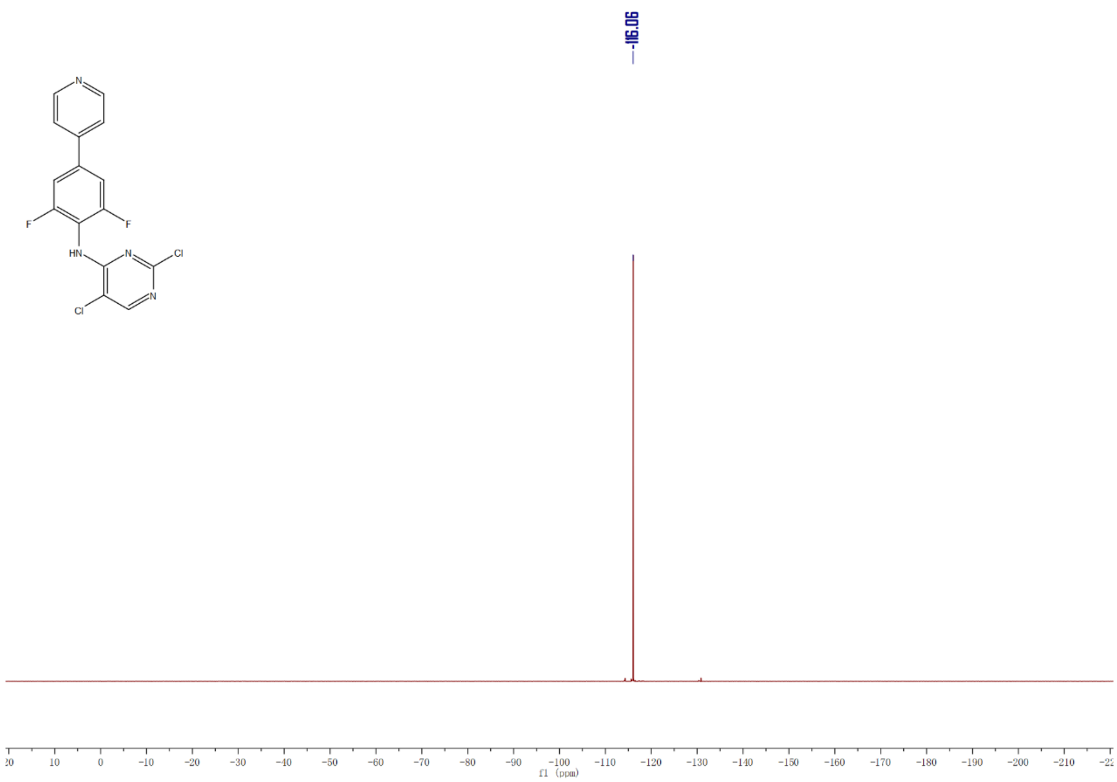

HRMS

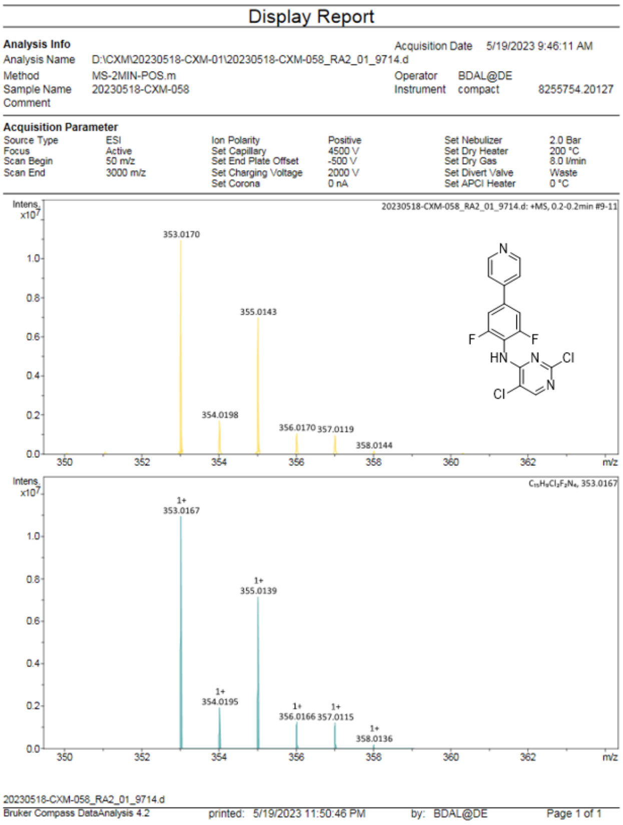

^{1}H NMR, ^{13}C NMR, HRMS spectra of 10x

^{1}H NMR

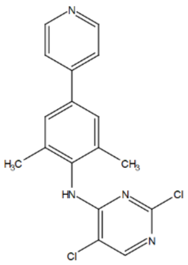

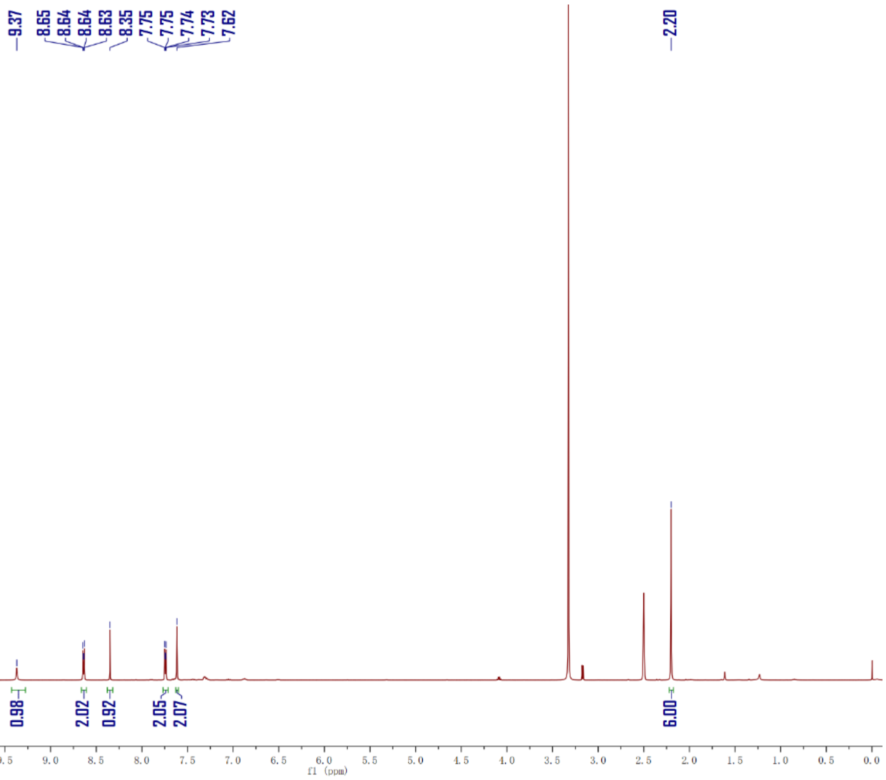

^{13}C NMR

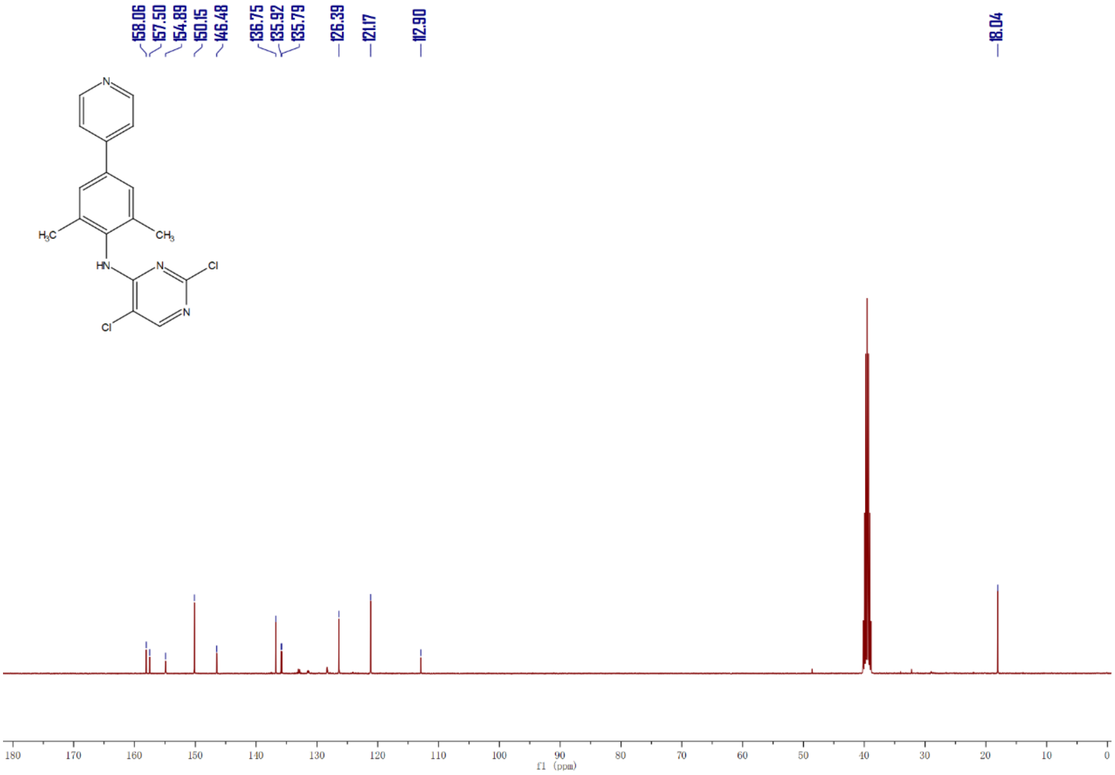

158.06
157.50
154.89
150.15
146.48
136.75
135.92
135.79
126.39
121.17
112.90
18.04
HN
N
Cl
H3C
CH3
180 170 160 150 140 130 120 110 100 90 80 70 60 50 40 30 20 10 0
f1 (ppm)

HRMS

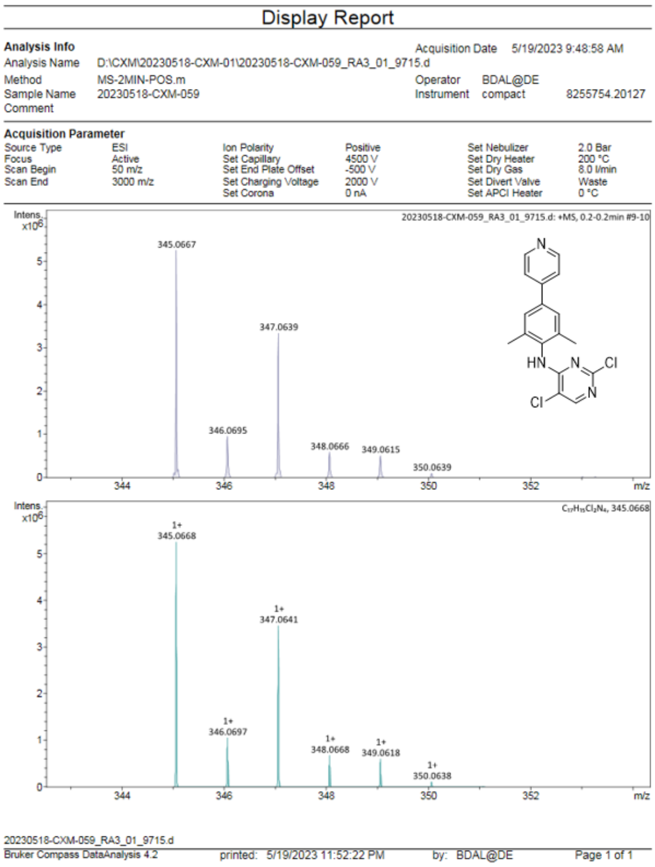

Display Report
Analysis Info
Analysis Name D:\CXM\20230518-CXM-01\20230518-CXM-059_RA3_01_9715.d
Method MS-2MIN-POS.m
Sample Name 20230518-CXM-059
Comment
Acquisition Date 5/19/2023 9:48:58 AM
Operator BDAL@DE
Instrument compact 8255754.20127
Acquisition Parameter
Source Type ESI
Focus Active
Scan Begin 50 m/z
Scan End 3000 m/z
Ion Polarity Positive
Set Capillary 4500 V
Set End Plate Offset -500 V
Set Charging Voltage 2000 V
Set Corona 0 nA
Set Nebulizer 2.0 Bar
Set Dry Heater 200 °C
Set Dry Gas 8.0 l/min
Set Divert Valve Waste
Set APCI Heater 0 °C
20230518-CXM-059_RA3_01_9715.d: +MS, 0.2-0.2min #9-10
Intens. x10^6
345.0667
346.0695
347.0639
348.0666
349.0615
350.0639
C17H15Cl2N4, 345.0668
1+ 345.0668
1+ 346.0697
1+ 347.0641
1+ 348.0668
1+ 349.0618
1+ 350.0638
m/z
20230518-CXM-059_RA3_01_9715.d
Bruker Compass DataAnalysis 4.2 printed: 5/19/2023 11:52:22 PM by: BDAL@DE Page 1 of 1

2. ^{1}H NMR, ^{13}C NMR, ^{19}F NMR, HRMS, HPLC spectra of A1−A24, B1−B22

^{1}H NMR, ^{13}C NMR, ^{19}F NMR, HRMS, HPLC spectra of A1

^{1}H NMR

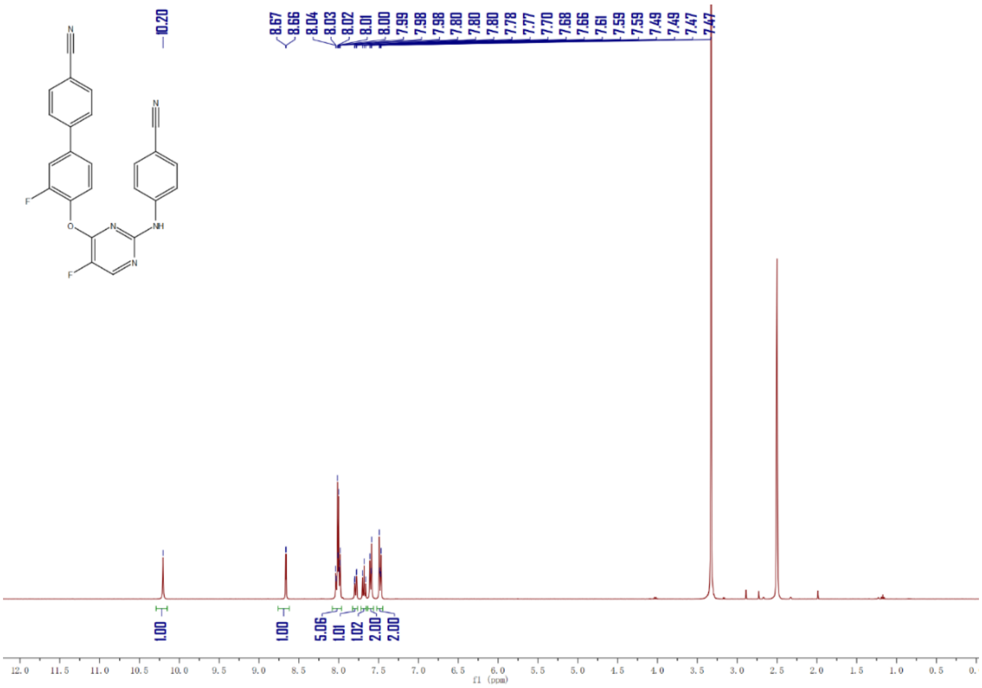

^{13}C NMR

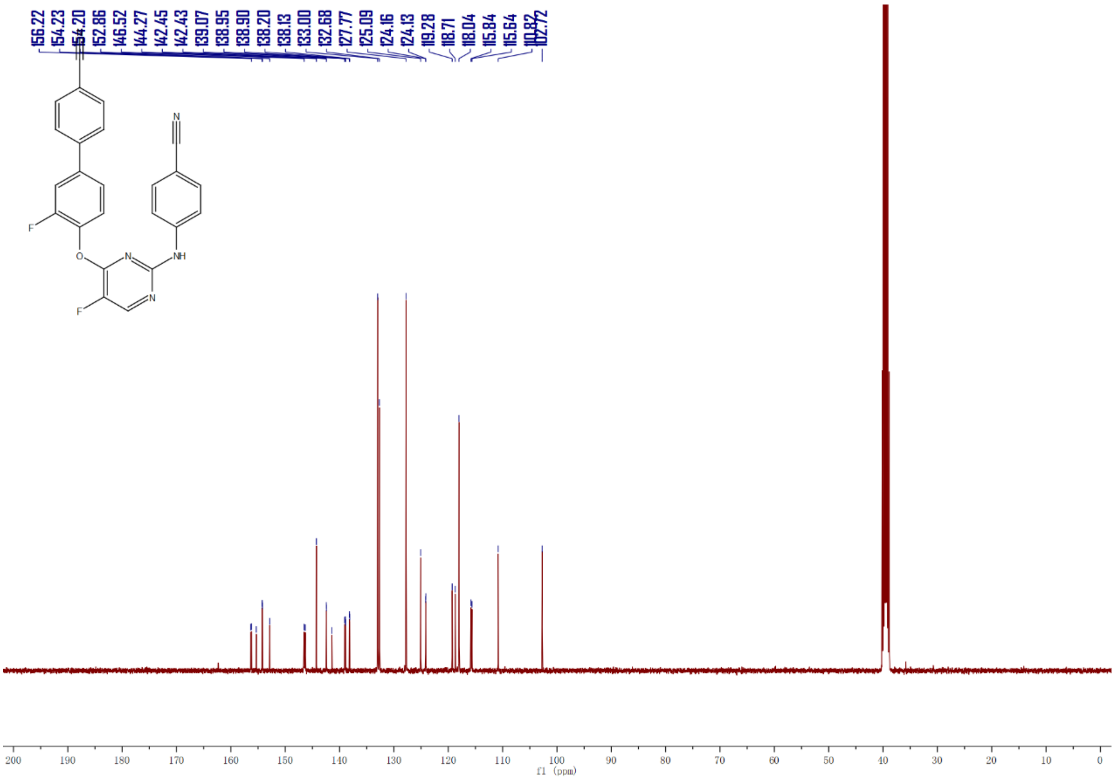

^{19}F NMR

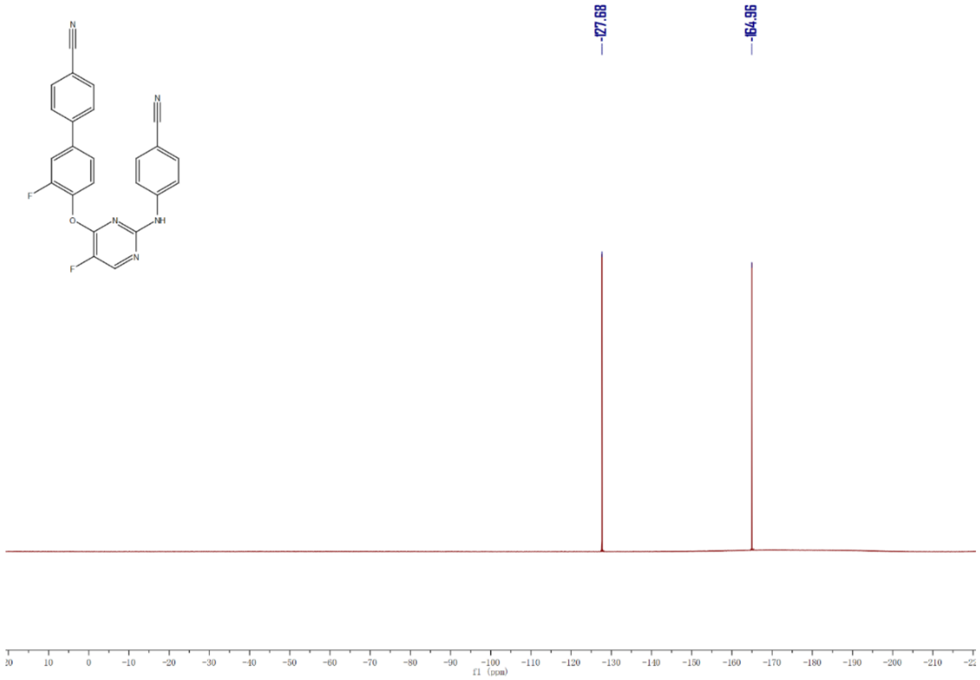

HRMS

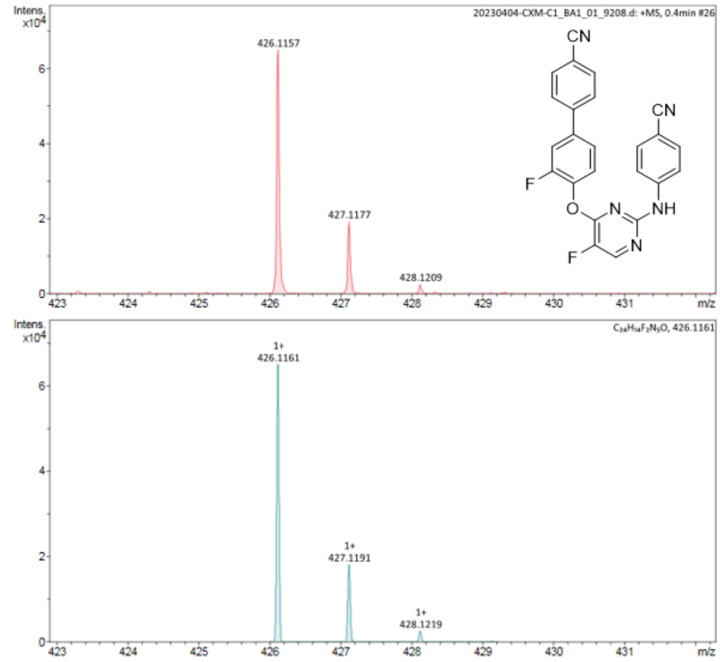

Display Report

Analysis Info

Analysis Name	D:\CXM\20230404\20230404-CXM-C1_BA1_01_9208.d	Acquisition Date	4/4/2023 4:11:30 PM
Method	MS-2MIN-POS.m	Operator	BDAL@DE
Sample Name	20230404-CXM-C1	Instrument	compact 8255754.20127
Comment			

Acquisition Parameter

Source Type	ESI	Ion Polarity	Positive	Set Nebulizer	2.0 Bar
Focus	Active	Set Capillary	4500 V	Set Dry Heater	200 °C
Scan Begin	50 m/z	Set End Plate Offset	-500 V	Set Dry Gas	8.0 l/min
Scan End	3000 m/z	Set Charging Voltage	2000 V	Set Divert Valve	Waste
		Set Corona	0 nA	Set APCI Heater	0 °C

20230404-CXM-C1_BA1_01_9208.d
Bruker Compass DataAnalysis 4.2 printed: 4/8/2023 9:37:56 PM by: BDAL@DE Page 1 of 1

HPLC

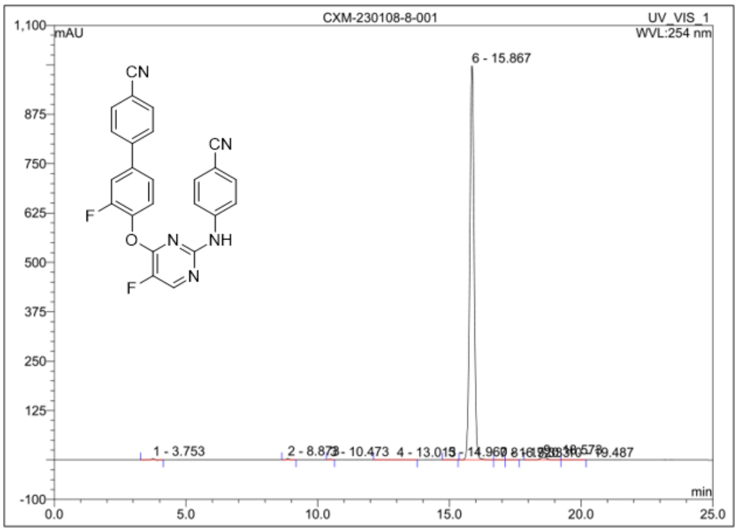

No.	Ret.Time min	Peak Name	Height mAU	Area mAU*s	Rel.Area %	Amount	Type
1	3.75	n.a.	2.839	27.96	0.24	n.a.	BMB*
2	8.87	n.a.	2.905	21.62	0.19	n.a.	BMB
3	10.47	n.a.	1.349	8.48	0.07	n.a.	BMB
4	13.01	n.a.	0.549	12.74	0.11	n.a.	BMB*
5	14.96	n.a.	1.184	13.84	0.12	n.a.	BMB
6	15.87	n.a.	998.053	11378.53	98.02	n.a.	BM
7	16.92	n.a.	0.330	4.67	0.04	n.a.	M
8	17.33	n.a.	0.406	5.67	0.05	n.a.	MB
9	18.57	n.a.	6.559	124.49	1.07	n.a.	BM
10	19.49	n.a.	0.313	9.85	0.08	n.a.	MB
Total:			1014.486	11607.852	100.00	0.000	

^{1}H NMR, ^{13}C NMR, ^{19}F NMR, HRMS, HPLC spectra of A2

^{1}H NMR

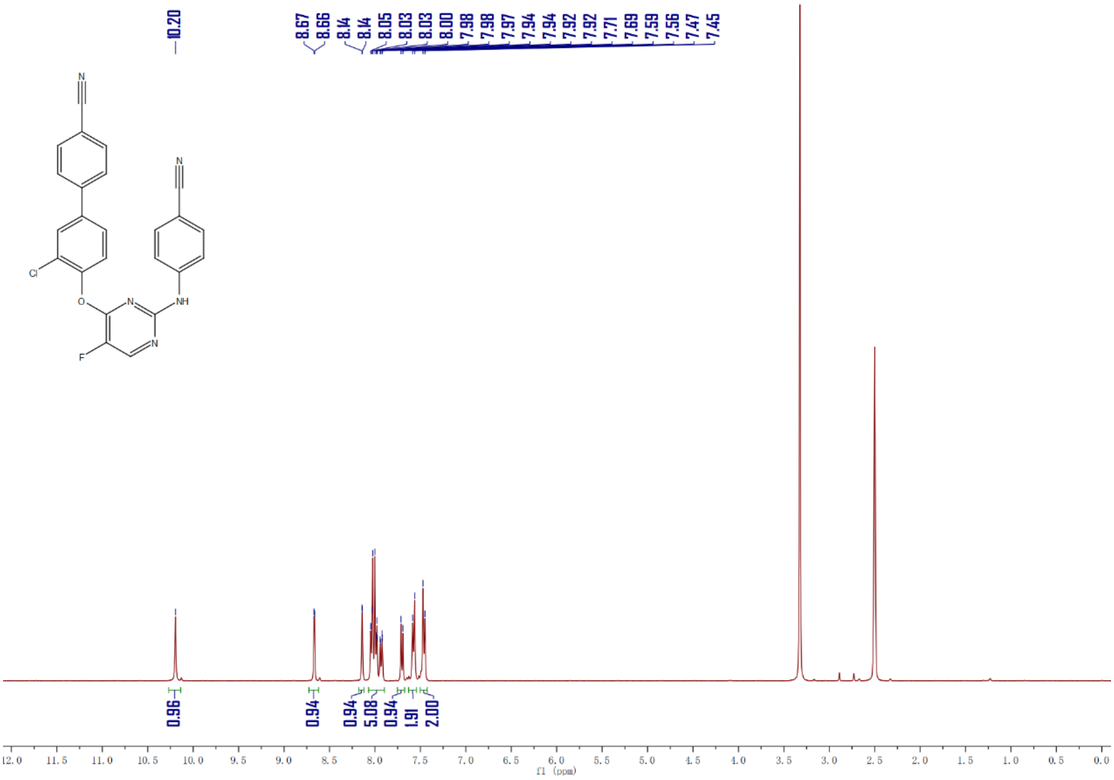

^{13}C NMR

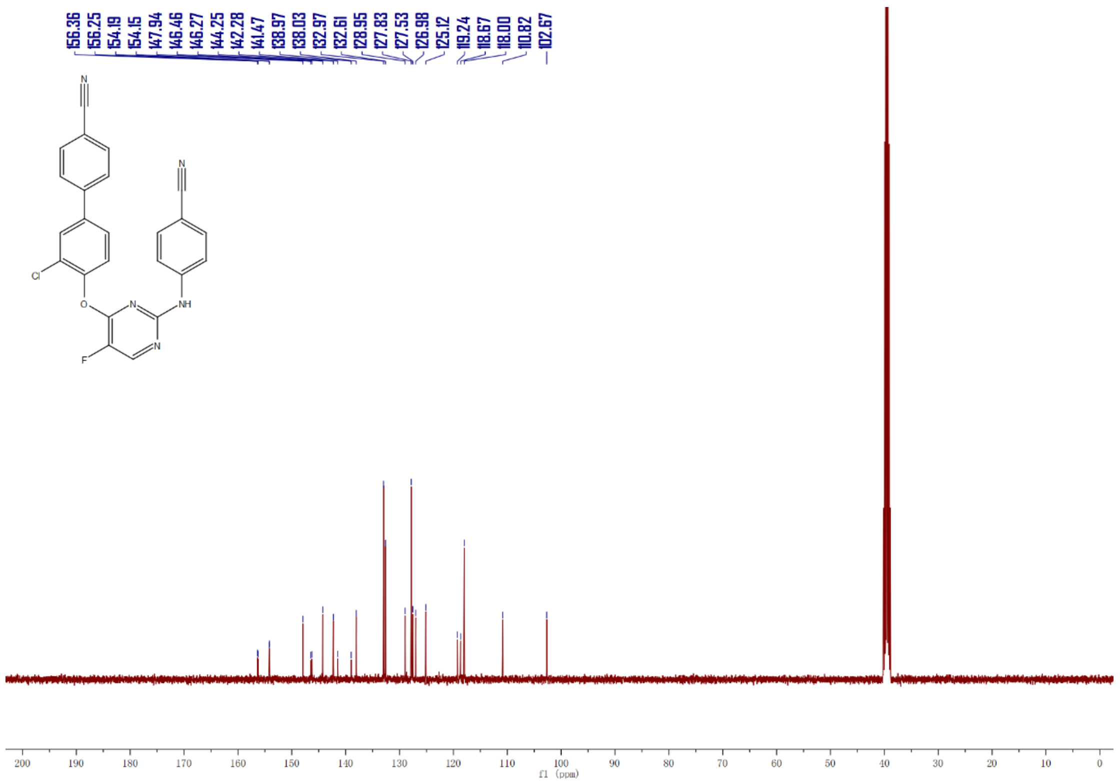

^{19}F NMR

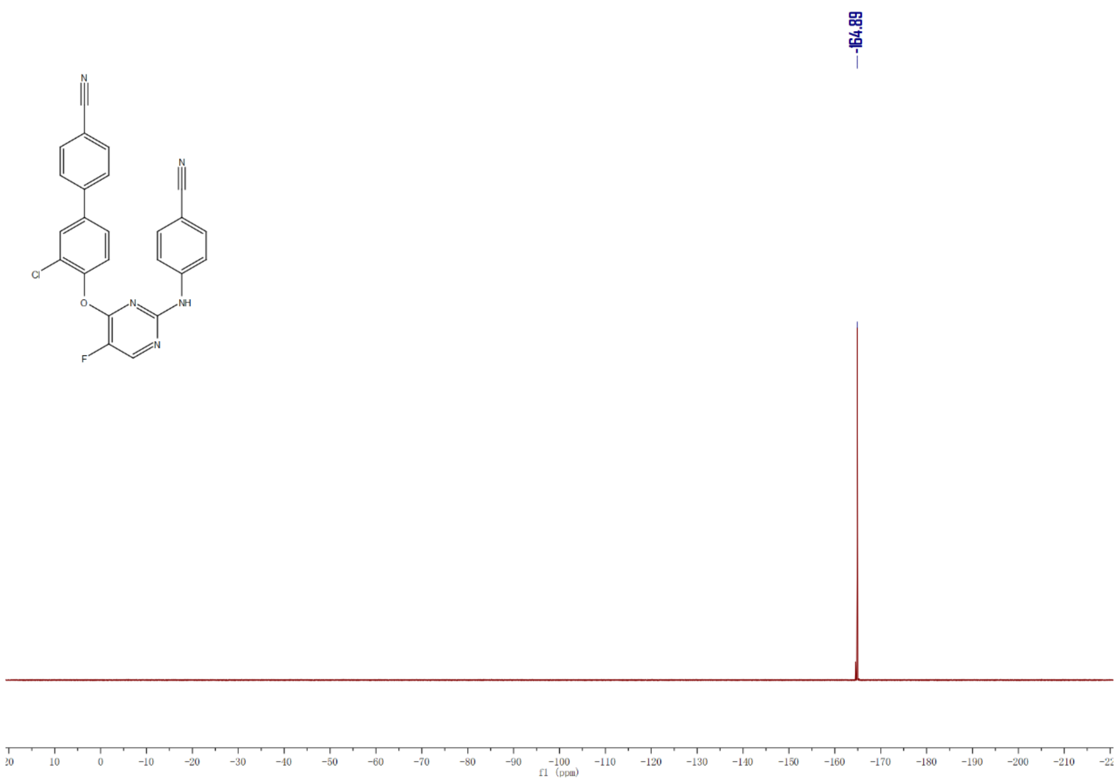

HRMS

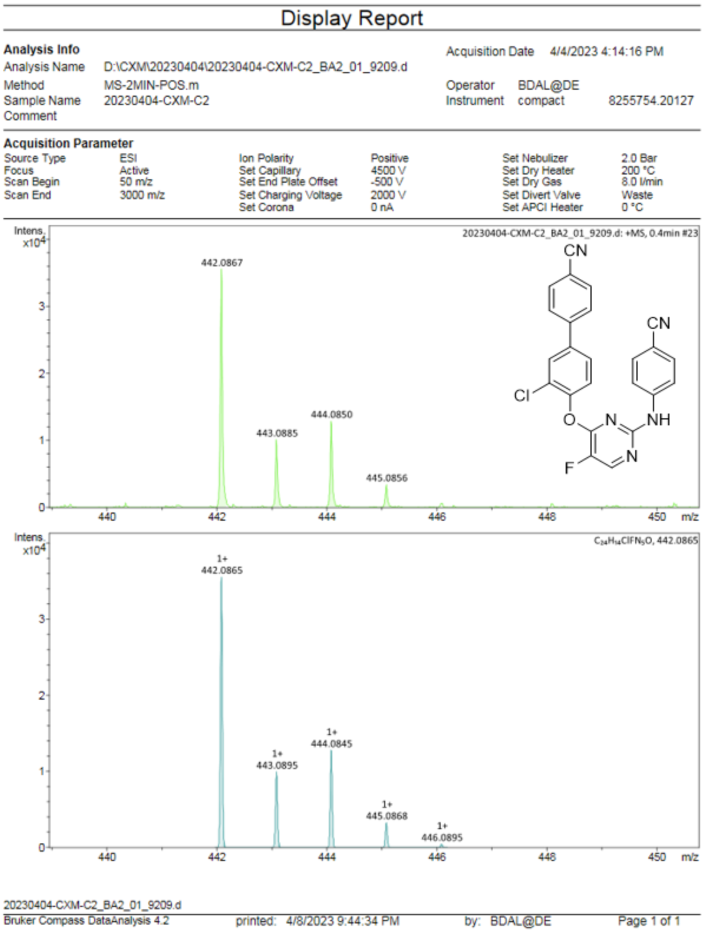

HPLC

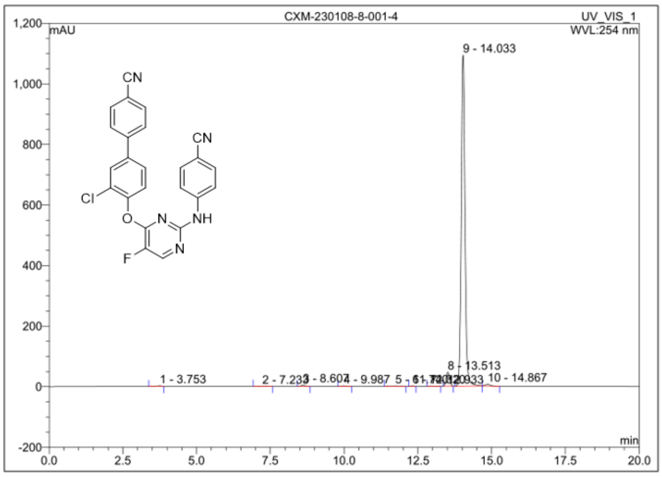

No.	Ret.Time min	Peak Name	Height mAU	Area mAU*s	Rel.Area %	Amount	Type
1	3.75	n.a.	2.262	27.52	0.27	n.a.	BMB*
2	7.23	n.a.	0.043	0.93	0.01	n.a.	BMB*
3	8.61	n.a.	4.033	28.48	0.28	n.a.	BMB
4	9.99	n.a.	0.993	5.72	0.06	n.a.	BMB*
5	11.74	n.a.	0.850	11.72	0.12	n.a.	BMB*
6	12.32	n.a.	1.100	6.67	0.07	n.a.	BMB
7	12.93	n.a.	0.703	3.42	0.03	n.a.	BMB*
8	13.51	n.a.	46.280	356.72	3.51	n.a.	BMB*
9	14.03	n.a.	1093.698	9667.39	95.05	n.a.	BMB*
10	14.87	n.a.	6.025	62.44	0.61	n.a.	BMB*
Total:			1155.987	10171.020	100.00	0.000	

^{1}H NMR, ^{13}C NMR, ^{19}F NMR, HRMS, HPLC spectra of A3

^{1}H NMR

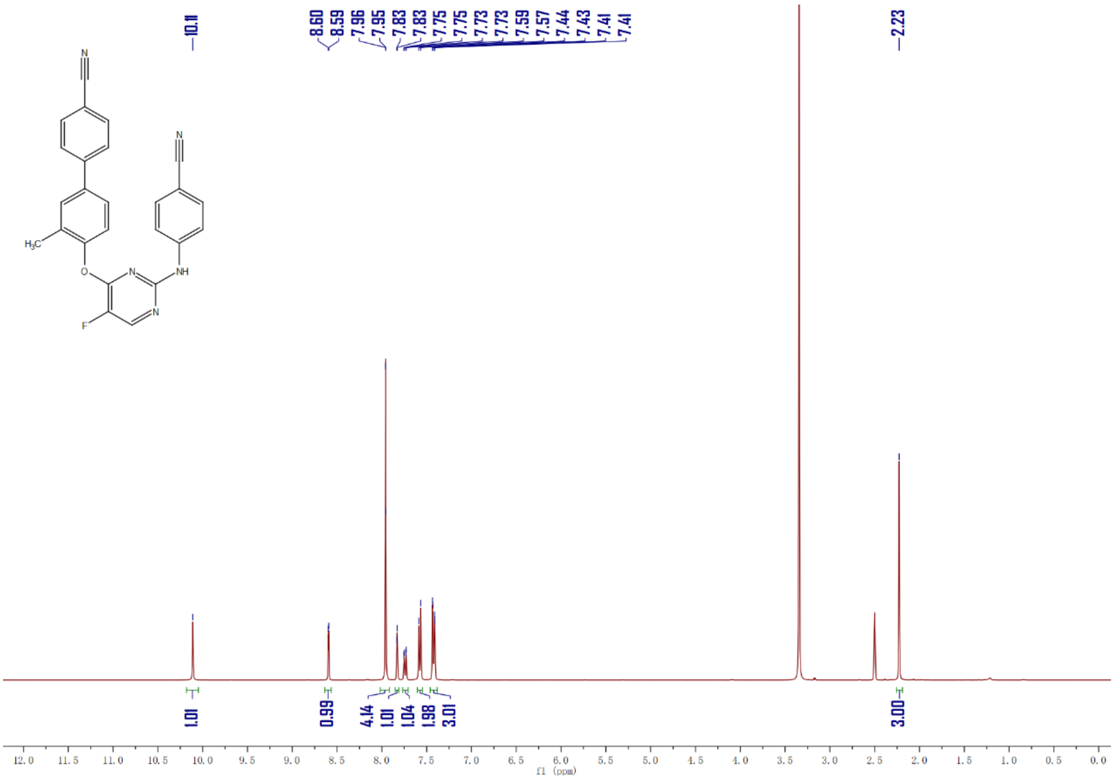

^{13}C NMR

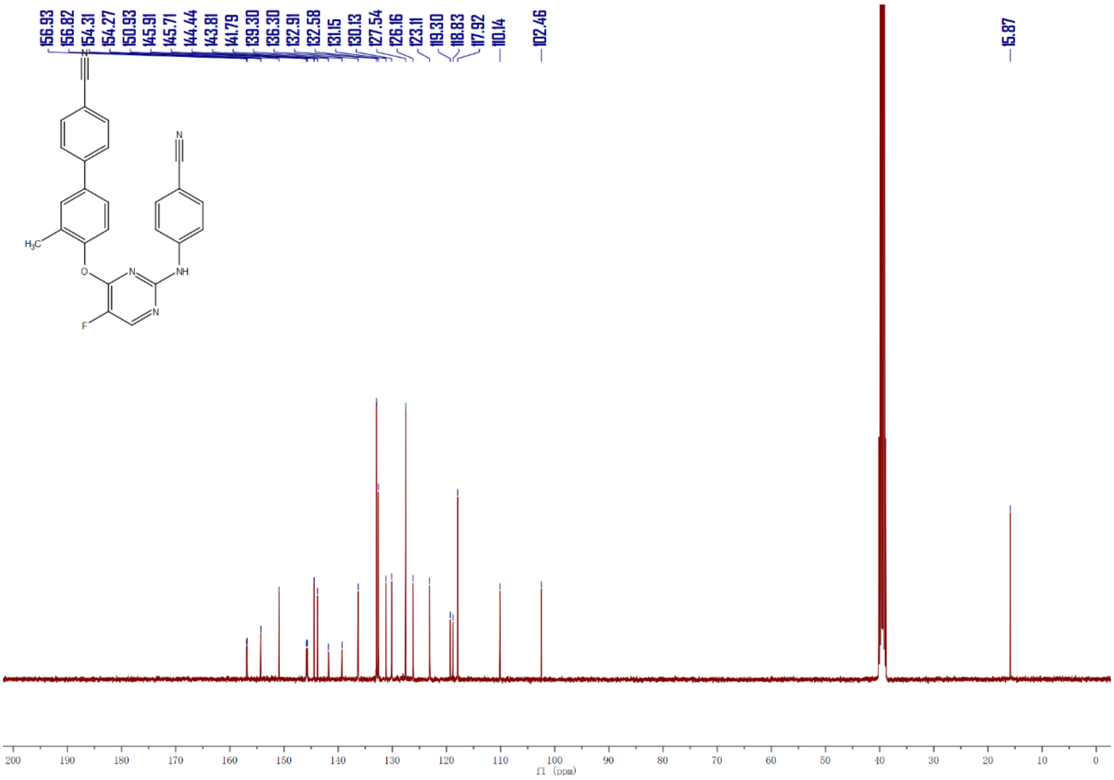

^{19}F NMR

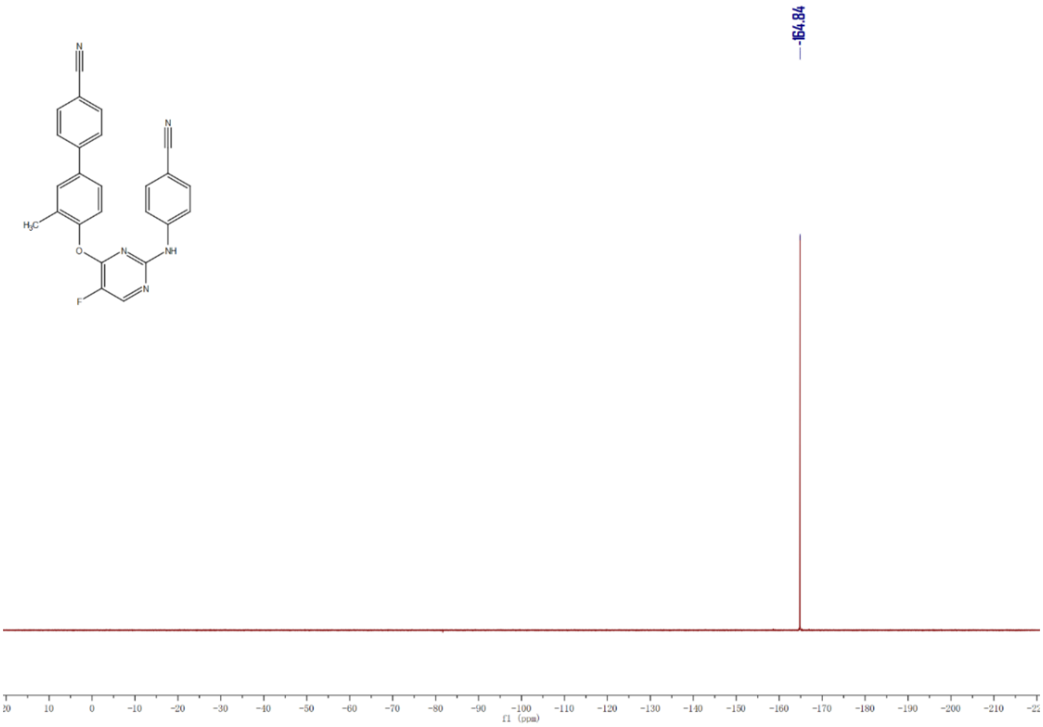

-164.84
f1 (ppm)

HRMS

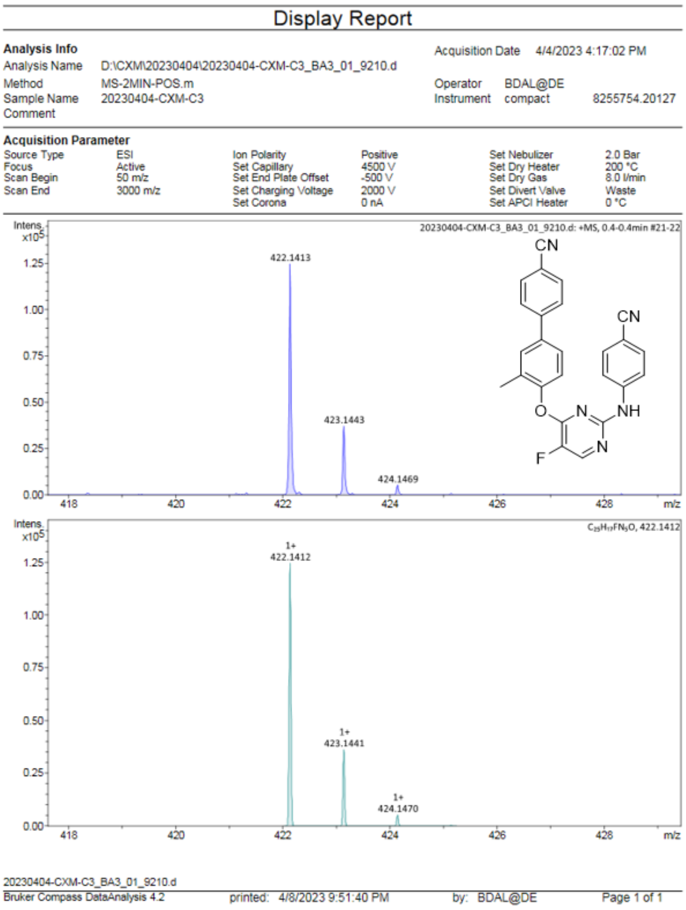

Display Report
Analysis Info
Analysis Name D:\CXM\20230404\20230404-CXM-C3_BA3_01_9210.d
Method MS-2MIN-POS.m
Sample Name 20230404-CXM-C3
Comment
Acquisition Date 4/4/2023 4:17:02 PM
Operator BDAL@DE
Instrument compact 8255754.20127
Acquisition Parameter
Source Type ESI
Focus Active
Scan Begin 50 m/z
Scan End 3000 m/z
Ion Polarity Positive
Set Capillary 4500 V
Set End Plate Offset -500 V
Set Charging Voltage 2000 V
Set Corona 0 nA
Set Nebulizer 2.0 Bar
Set Dry Heater 200 °C
Set Dry Gas 8.0 l/min
Set Divert Valve Waste
Set APCI Heater 0 °C
20230404-CXM-C3_BA3_01_9210.d: +MS, 0.4-0.4min #21-22
422.1413
423.1443
424.1469
422.1412
423.1441
424.1470
m/z
20230404-CXM-C3_BA3_01_9210.d
Bruker Compass DataAnalysis 4.2 printed: 4/8/2023 9:51:40 PM by: BDAL@DE Page 1 of 1

HPLC

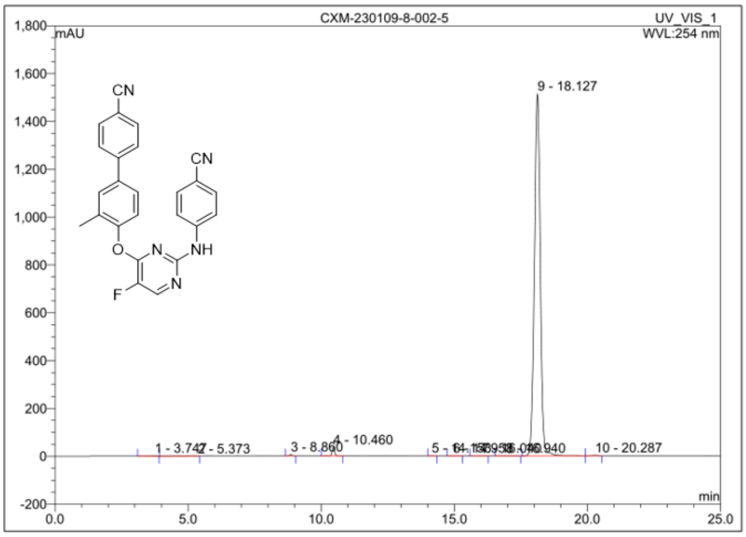

No.	Ret.Time min	Peak Name	Height mAU	Area mAU*s	Rel.Area %	Amount	Type
1	3.75	n.a.	2.417	36.48	0.15	n.a.	BMB
2	5.37	n.a.	0.042	25.98	0.11	n.a.	BMB*
3	8.86	n.a.	6.130	44.86	0.19	n.a.	BMB
4	10.46	n.a.	35.720	245.24	1.03	n.a.	BMB*
5	14.15	n.a.	1.543	13.40	0.06	n.a.	BMB
6	14.95	n.a.	0.732	8.60	0.04	n.a.	BMB
7	16.04	n.a.	0.678	11.44	0.05	n.a.	BMB
8	16.94	n.a.	0.814	24.10	0.10	n.a.	BMB
9	18.13	n.a.	1515.381	23398.14	98.19	n.a.	BMb*
10	20.29	n.a.	1.327	22.00	0.09	n.a.	bMB*
Total:			1564.785	23830.247	100.00	0.000	

^{1}H NMR, ^{13}C NMR, ^{19}F NMR, HRMS, HPLC spectra of A4

^{1}H NMR

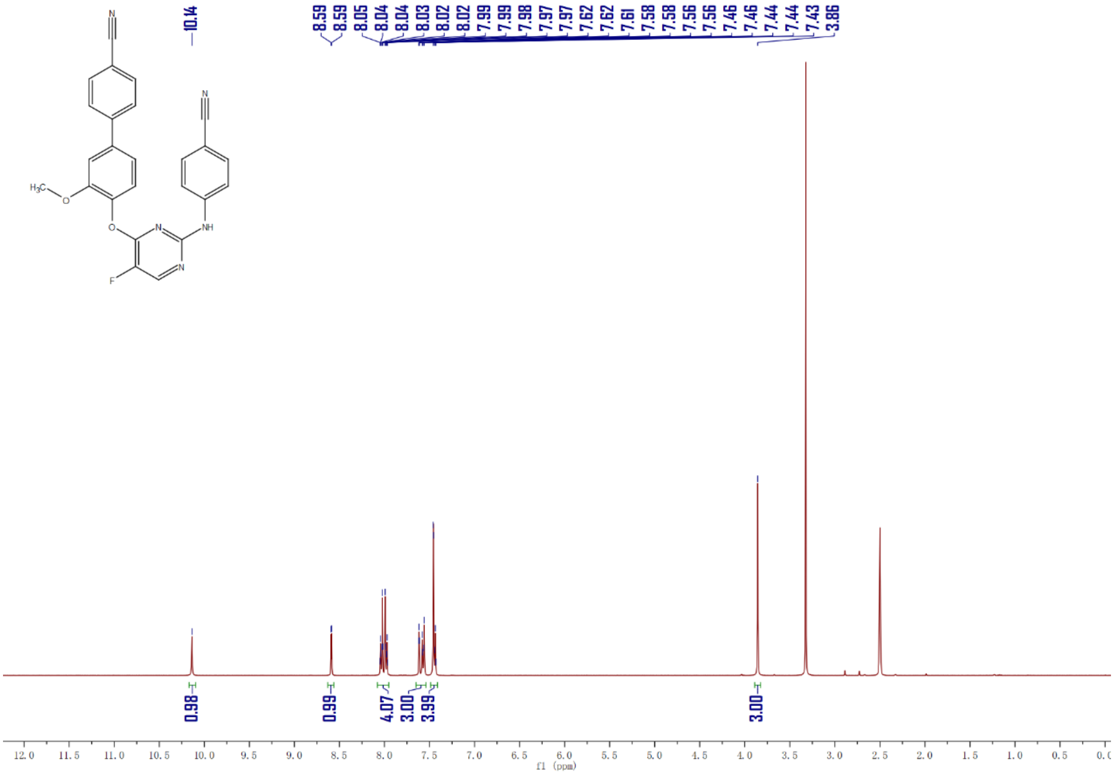

^{13}C NMR

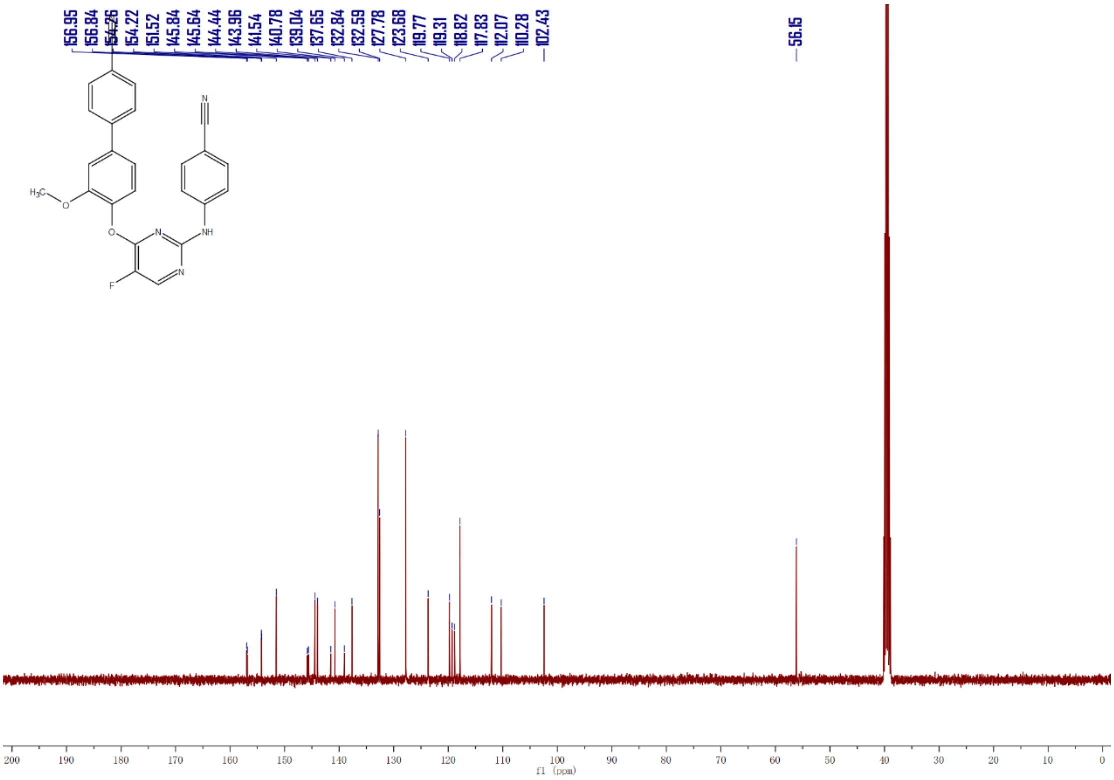

^{19}F NMR

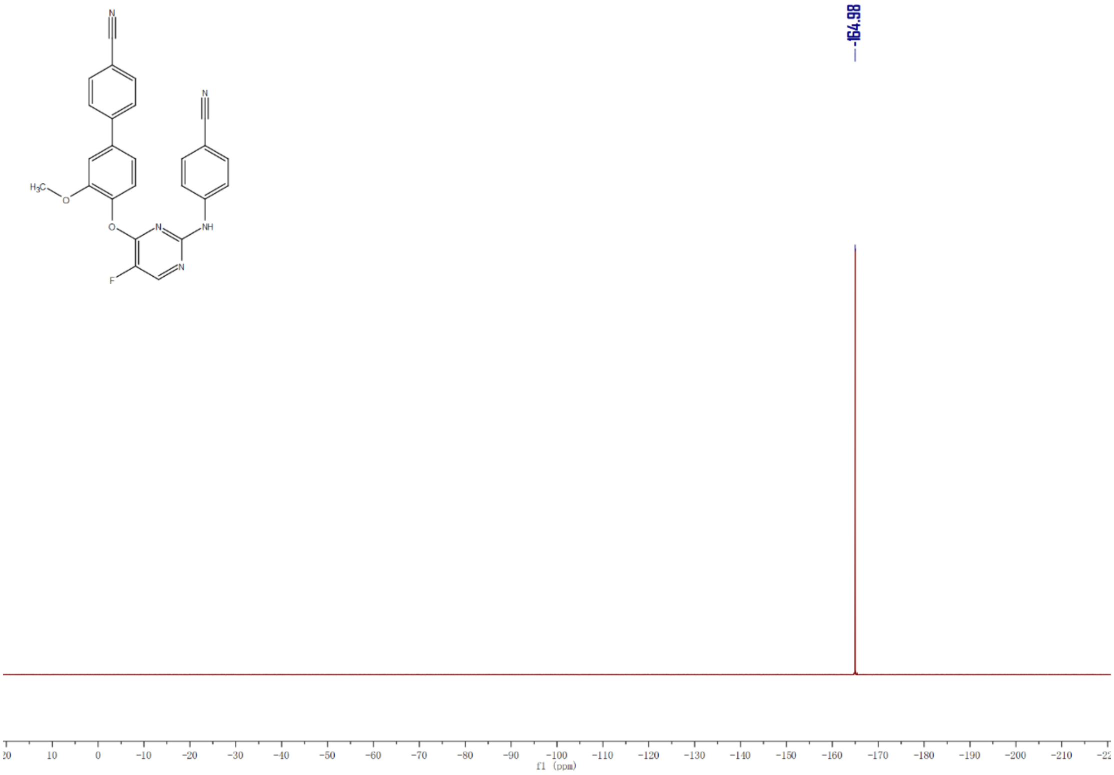

HRMS

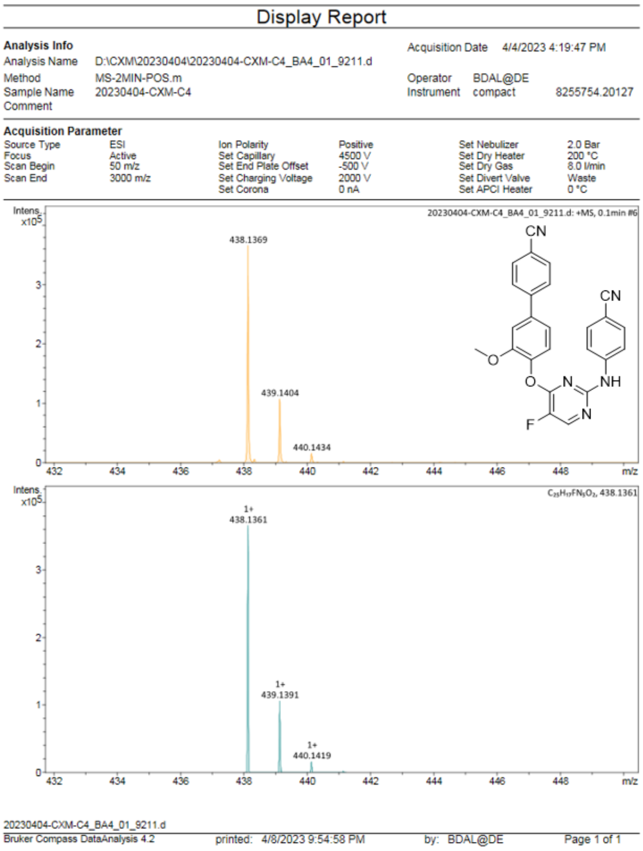

HPLC

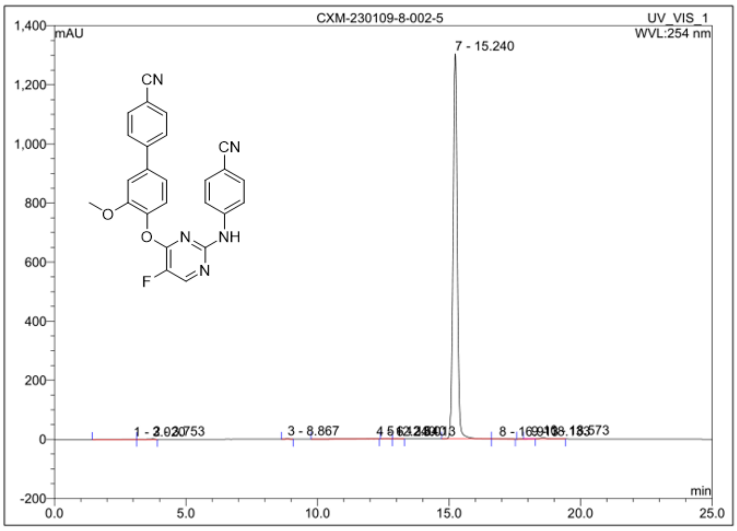

No.	Ret.Time min	Peak Name	Height mAU	Area mAU*s	Rel.Area %	Amount	Type
1	3.02	n.a.	0.045	9.76	0.07	n.a.	BMb
2	3.75	n.a.	2.299	35.72	0.24	n.a.	bMB
3	8.87	n.a.	2.708	19.99	0.14	n.a.	BMB
4	12.24	n.a.	0.209	27.19	0.18	n.a.	BMB*
5	12.64	n.a.	0.958	7.43	0.05	n.a.	BMB
6	13.01	n.a.	0.320	3.17	0.02	n.a.	BMB
7	15.24	n.a.	1304.391	14470.59	98.44	n.a.	BMb
8	16.91	n.a.	0.270	7.86	0.05	n.a.	bMB
9	18.13	n.a.	0.579	7.84	0.05	n.a.	Ru
10	18.57	n.a.	4.500	110.35	0.75	n.a.	BMB
Total:			1316.278	14699.898	100.00	0.000	

^{1}H NMR, ^{13}C NMR, ^{19}F NMR, HRMS, HPLC spectra of A5

^{1}H NMR

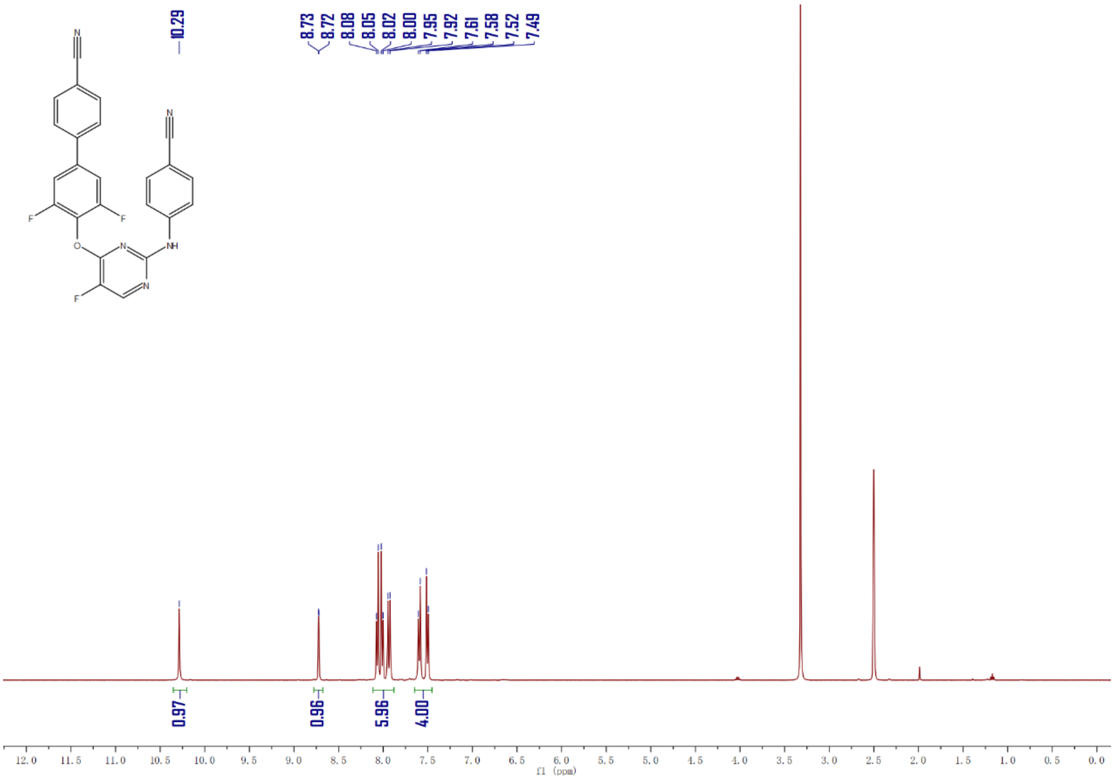

^{13}C NMR

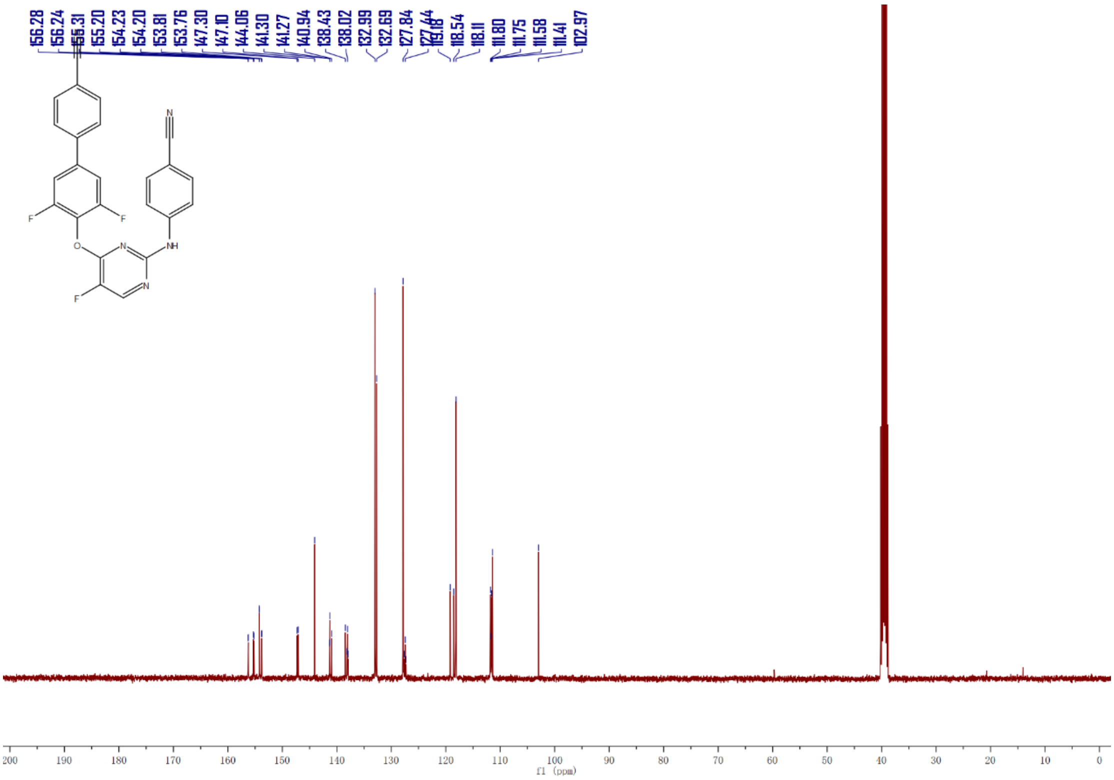

^{19}F NMR

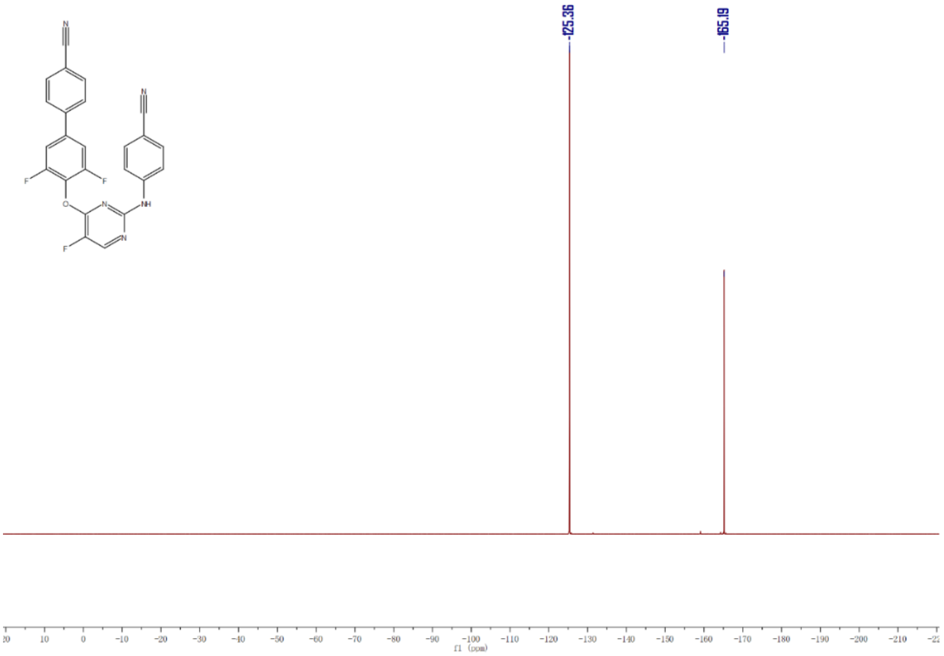

HRMS

Display Report

Analysis Info

Analysis Name	D:\CXM\20230404\20230404-CXM-C5_BA5_01_9212.d	Acquisition Date	4/4/2023 4:22:32 PM
Method	MS-2MIN-POS.m	Operator	BDAL@DE
Sample Name	20230404-CXM-C5	Instrument	compact 8255754.20127
Comment			

Acquisition Parameter

Source Type	ESI	Ion Polarity	Positive	Set Nebulizer	2.0 Bar
Focus	Active	Set Capillary	4500 V	Set Dry Heater	200 °C
Scan Begin	50 m/z	Set End Plate Offset	-500 V	Set Dry Gas	8.0 l/min
Scan End	3000 m/z	Set Charging Voltage	2000 V	Set Divert Valve	Waste
		Set Corona	0 nA	Set APCI Heater	0 °C

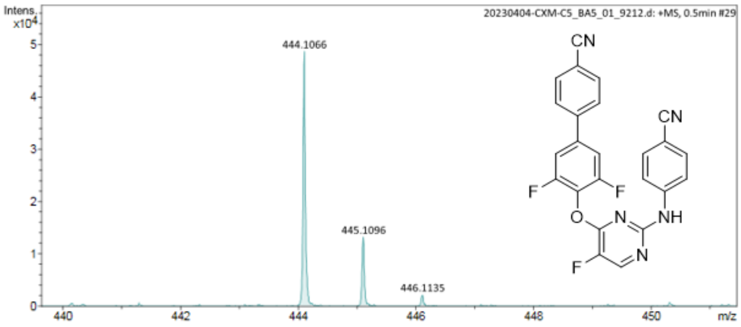

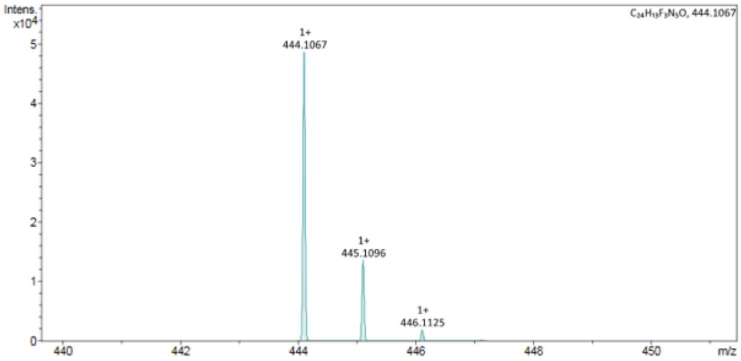

20230404-CXM-C5_BA5_01_9212.d

Bruker Compass DataAnalysis 4.2 printed: 4/8/2023 10:00:06 PM by: BDAL@DE Page 1 of 1

HPLC

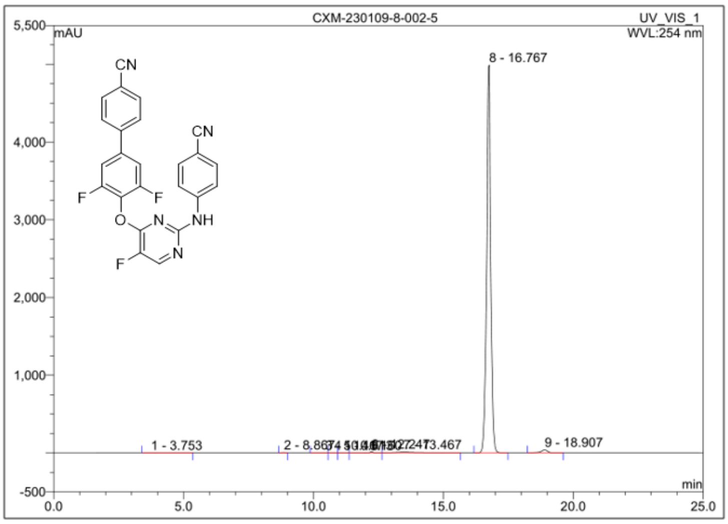

No.	Ret.Time min	Peak Name	Height mAU	Area mAU*s	Rel.Area %	Amount	Type
1	3.75	n.a.	1.850	13.29	0.02	n.a.	BMB*
2	8.87	n.a.	5.548	40.13	0.07	n.a.	BMB*
3	10.47	n.a.	0.322	4.80	0.01	n.a.	BM *
4	10.81	n.a.	0.775	10.26	0.02	n.a.	M *
5	11.16	n.a.	0.633	13.27	0.02	n.a.	M *
6	12.25	n.a.	16.118	185.80	0.33	n.a.	M *
7	13.47	n.a.	9.894	701.25	1.26	n.a.	MB*
8	16.77	n.a.	4989.223	53795.61	96.93	n.a.	BMB*
9	18.91	n.a.	39.296	732.30	1.32	n.a.	BMB
Total:			5063.660	55496.710	100.00	0.000	

^{1}H NMR, ^{13}C NMR, ^{19}F NMR, HRMS, HPLC spectra of A6

^{1}H NMR

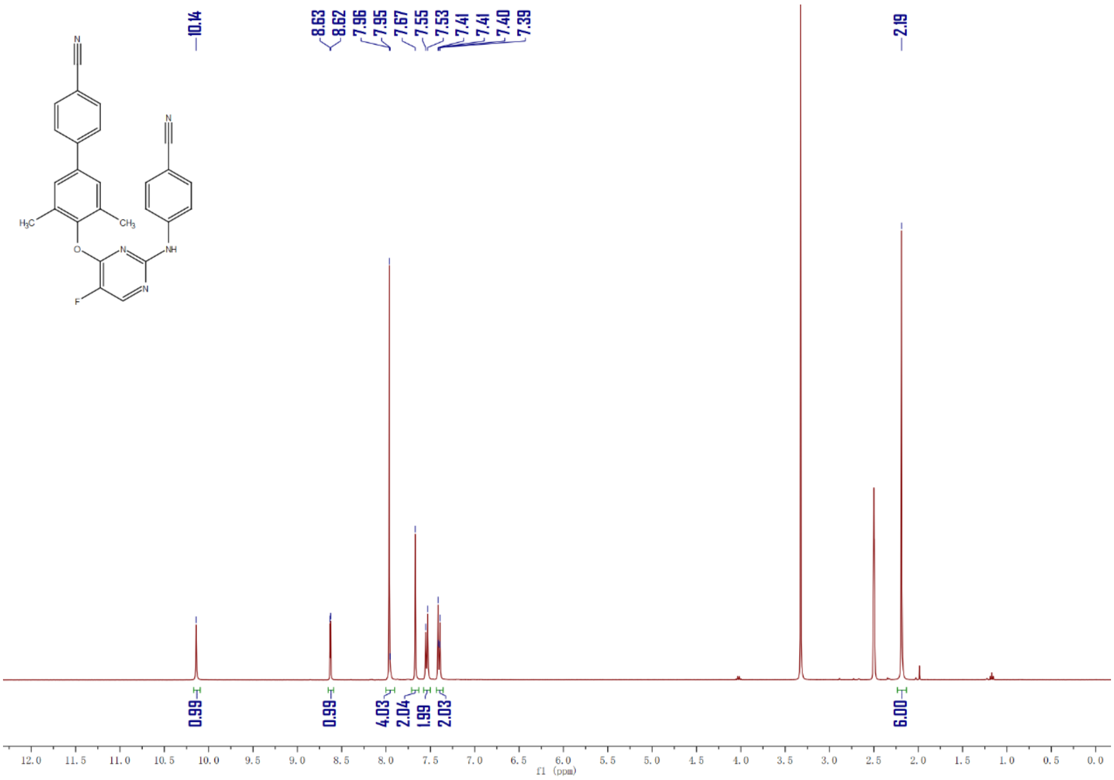

^{13}C NMR

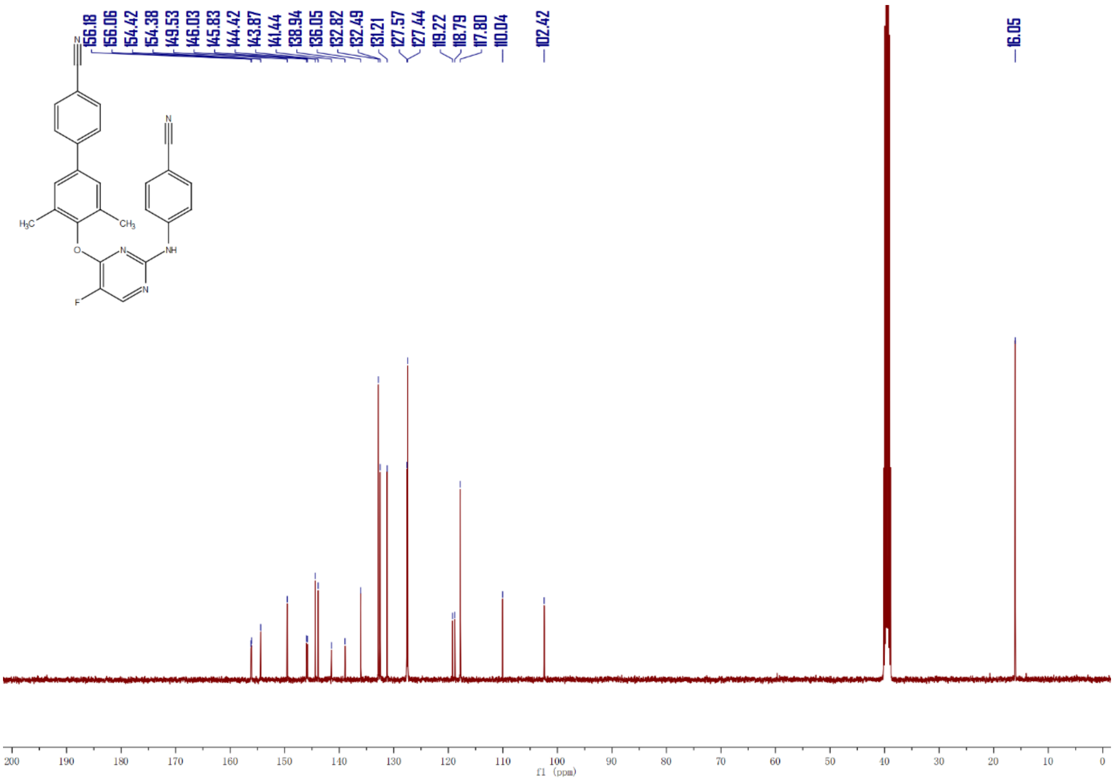

^{19}F NMR

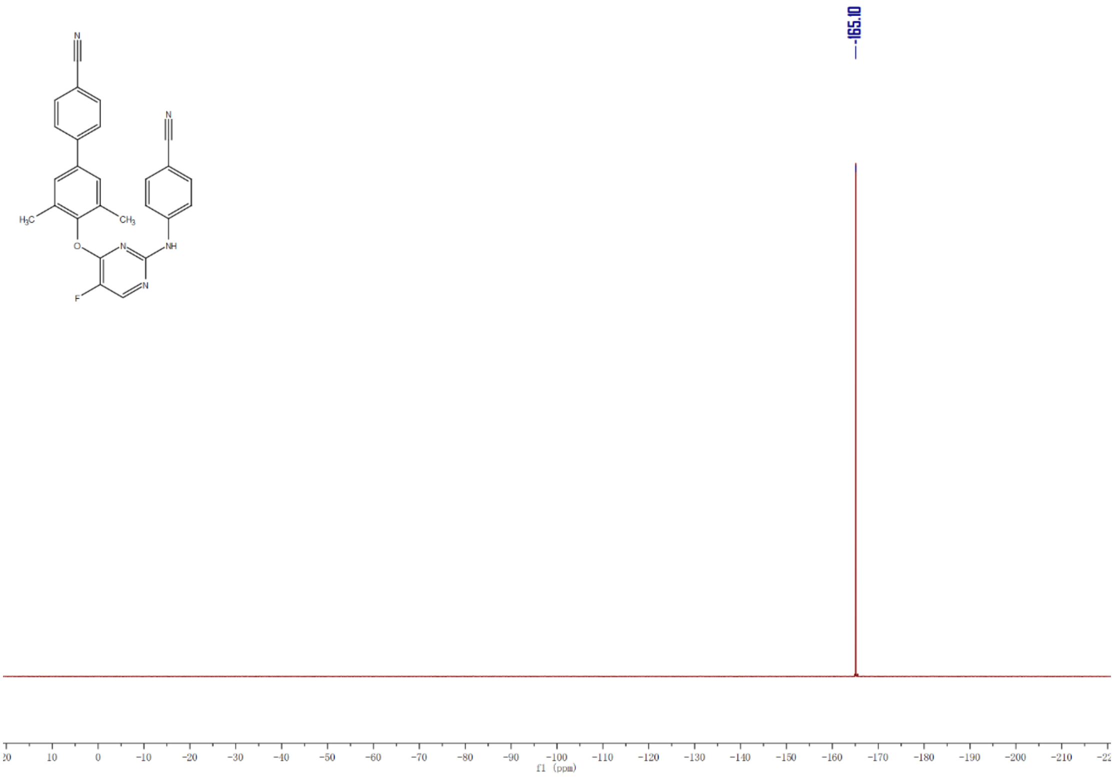

HRMS

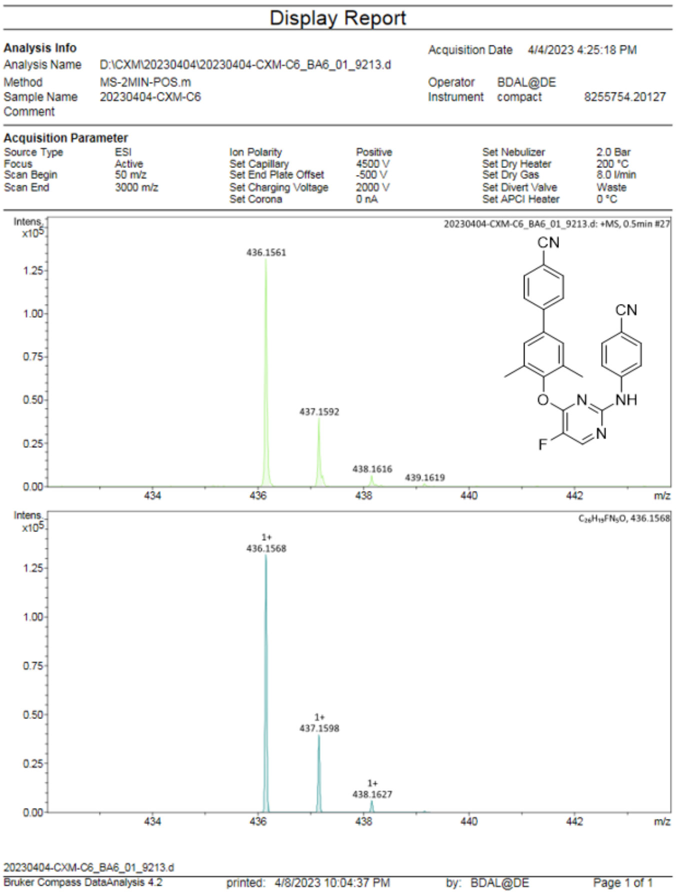

HPLC

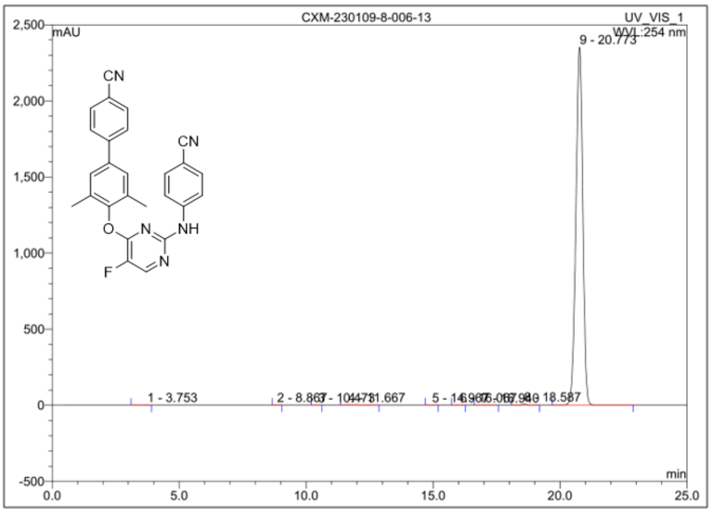

No.	Ret.Time min	Peak Name	Height mAU	Area mAU*s	Rel.Area %	Amount	Type
1	3.75	n.a.	2.797	39.45	0.09	n.a.	BMB
2	8.87	n.a.	2.463	18.07	0.04	n.a.	BMB
3	10.47	n.a.	1.322	9.20	0.02	n.a.	BMB
4	11.67	n.a.	2.275	79.73	0.18	n.a.	BMB*
5	14.97	n.a.	0.734	8.23	0.02	n.a.	BMB
6	16.07	n.a.	0.819	10.84	0.02	n.a.	BMB
7	16.94	n.a.	0.356	8.62	0.02	n.a.	BMB
8	18.59	n.a.	4.775	86.85	0.20	n.a.	BMB
9	20.77	n.a.	2352.860	44208.45	99.41	n.a.	BMB
Total:			2368.402	44469.427	100.00	0.000	

^{1}H NMR, ^{13}C NMR, ^{19}F NMR, HRMS, HPLC spectra of A7

^{1}H NMR

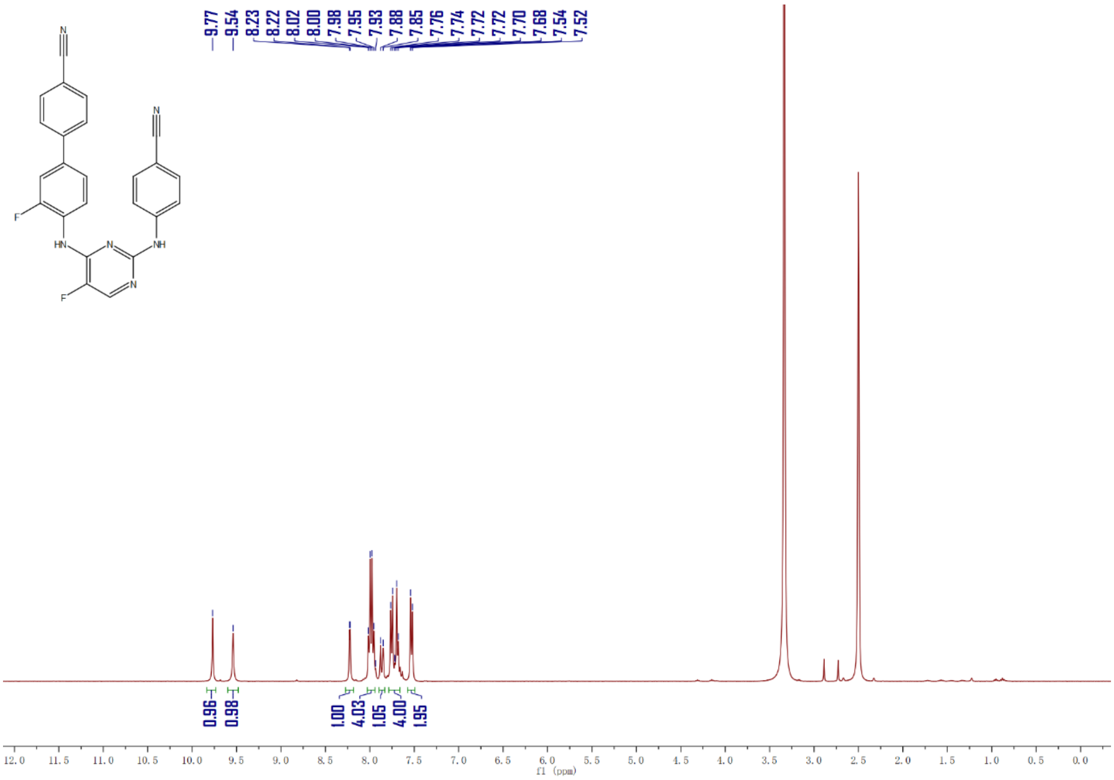

^{13}C NMR

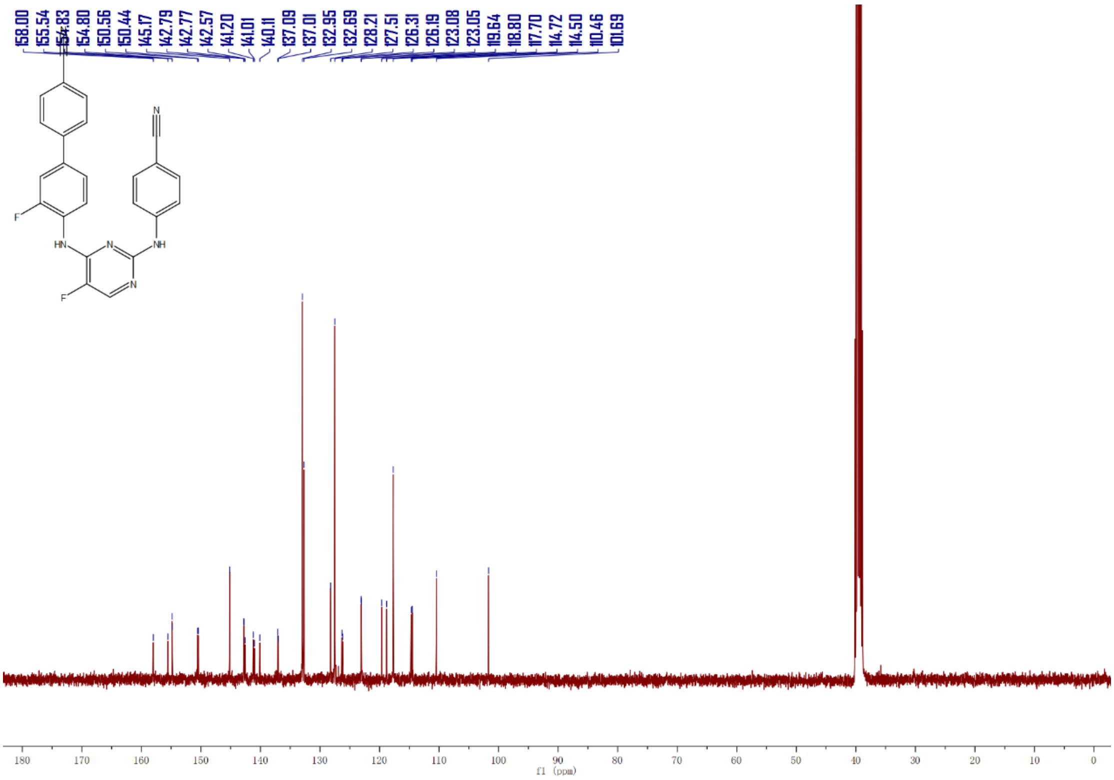

^{19}F NMR

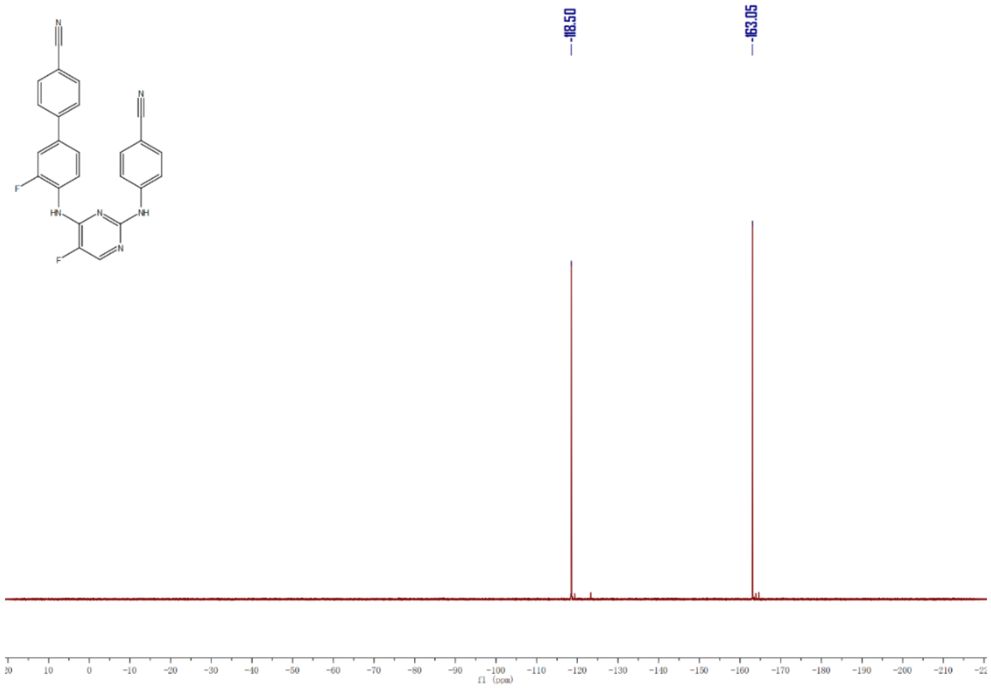

HRMS

Display Report

**Analysis Info**		Acquisition Date	4/4/2023 4:28:05 PM
Analysis Name	D:\CXM\20230404\20230404-CXM-C7_BA7_01_9214.d		
Method	MS-2MIN-POS.m	Operator	BDAL@DE
Sample Name	20230404-CXM-C7	Instrument	compact 8255754.20127
Comment			

Acquisition Parameter

Source Type	ESI	Ion Polarity	Positive	Set Nebulizer	2.0 Bar
Focus	Active	Set Capillary	4500 V	Set Dry Heater	200 °C
Scan Begin	50 m/z	Set End Plate Offset	-500 V	Set Dry Gas	8.0 l/min
Scan End	3000 m/z	Set Charging Voltage	2000 V	Set Divert Valve	Waste
		Set Corona	0 nA	Set APCI Heater	0 °C

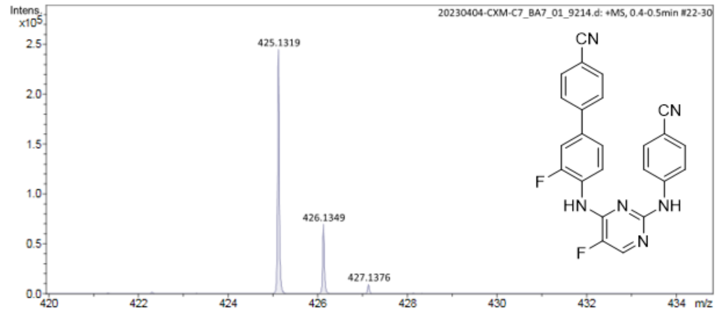

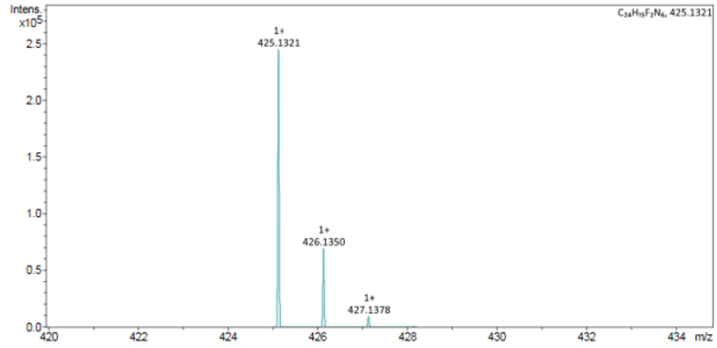

20230404-CXM-C7_BA7_01_9214.d
Bruker Compass DataAnalysis 4.2 printed: 4/8/2023 10:14:23 PM by: BDAL@DE Page 1 of 1

HPLC

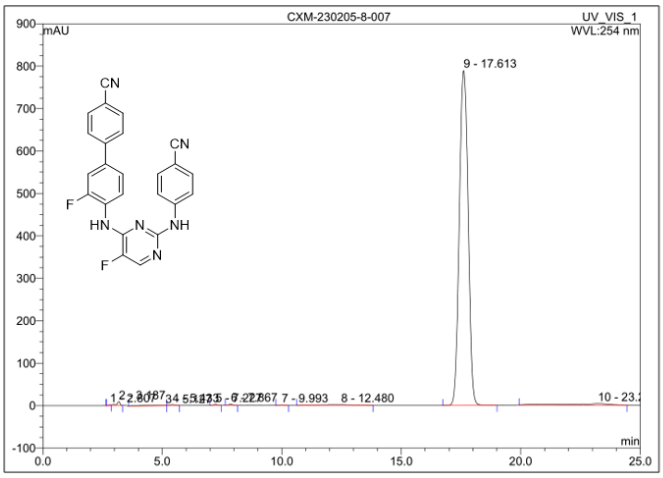

No.	Ret.Time min	Peak Name	Height mAU	Area mAU*s	Rel.Area %	Amount	Type
1	2.81	n.a.	0.768	3.23	0.02	n.a.	Ru
2	3.19	n.a.	9.181	105.44	0.50	n.a.	BMB*
3	5.13	n.a.	0.047	44.75	0.21	n.a.	BMb*
4	5.43	n.a.	1.941	22.37	0.11	n.a.	bMB
5	7.23	n.a.	2.088	22.21	0.10	n.a.	BMB
6	7.87	n.a.	2.715	30.73	0.15	n.a.	BMB
7	9.99	n.a.	1.219	14.95	0.07	n.a.	BMB
8	12.48	n.a.	1.716	156.80	0.74	n.a.	BMB
9	17.61	n.a.	789.679	20315.92	95.96	n.a.	BMB*
10	23.23	n.a.	3.496	453.91	2.14	n.a.	BMB*
Total:			812.852	21170.321	100.00	0.000	

^1H NMR, ^{13}C NMR, ^{19}F NMR, HRMS, HPLC spectra of A8

^1H NMR

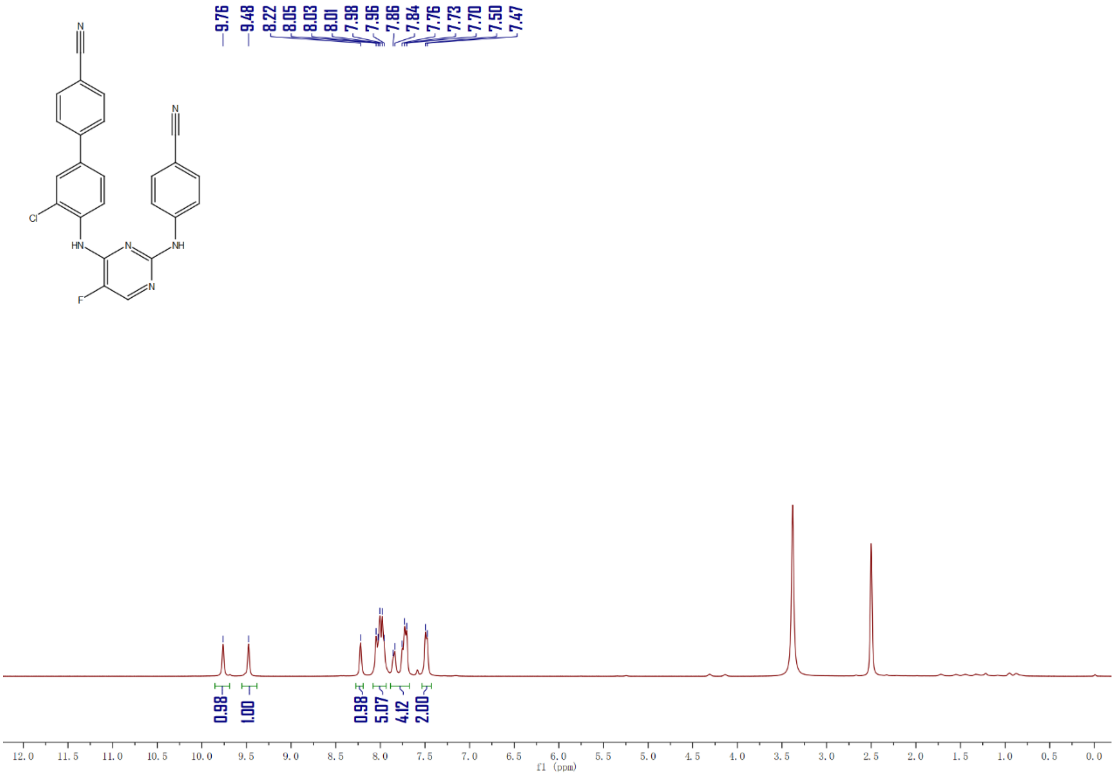

^{13}C NMR

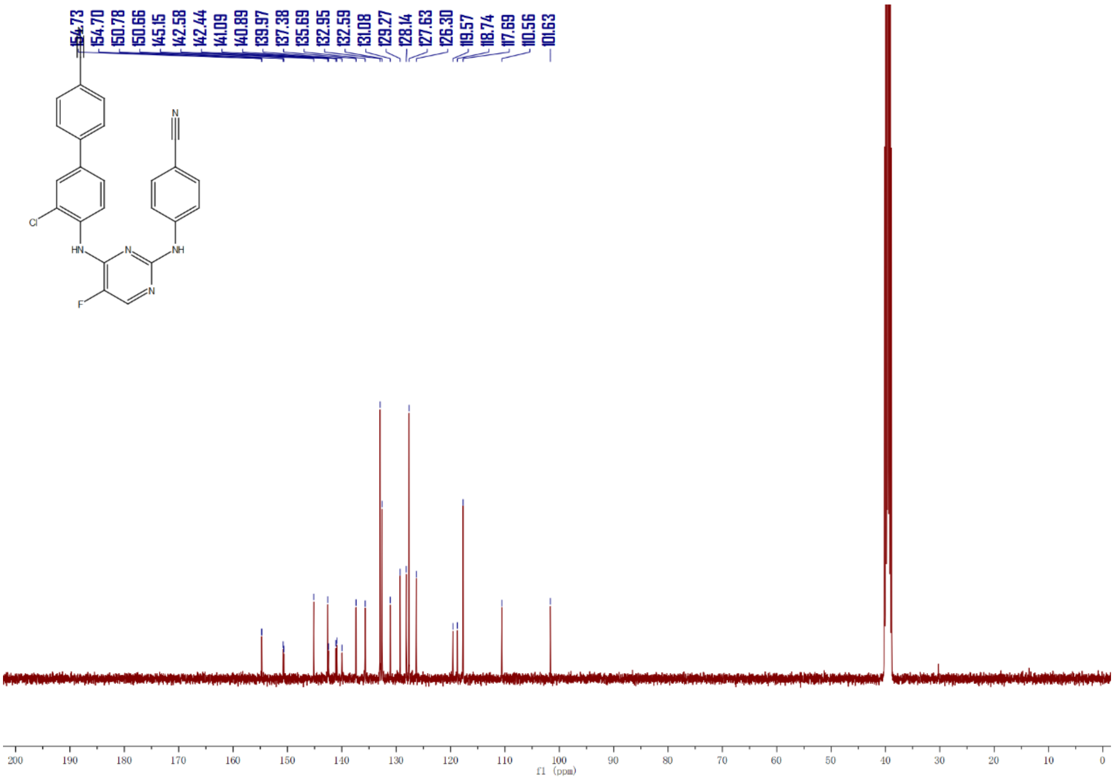

^{19}F NMR

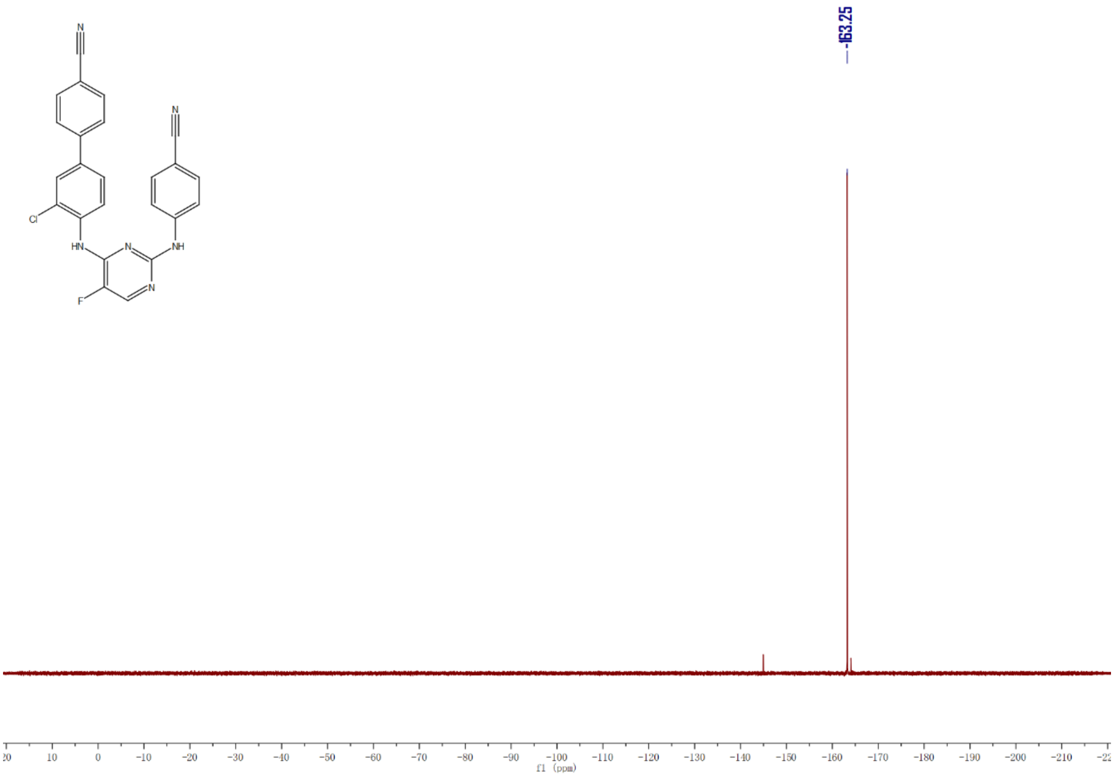

HRMS

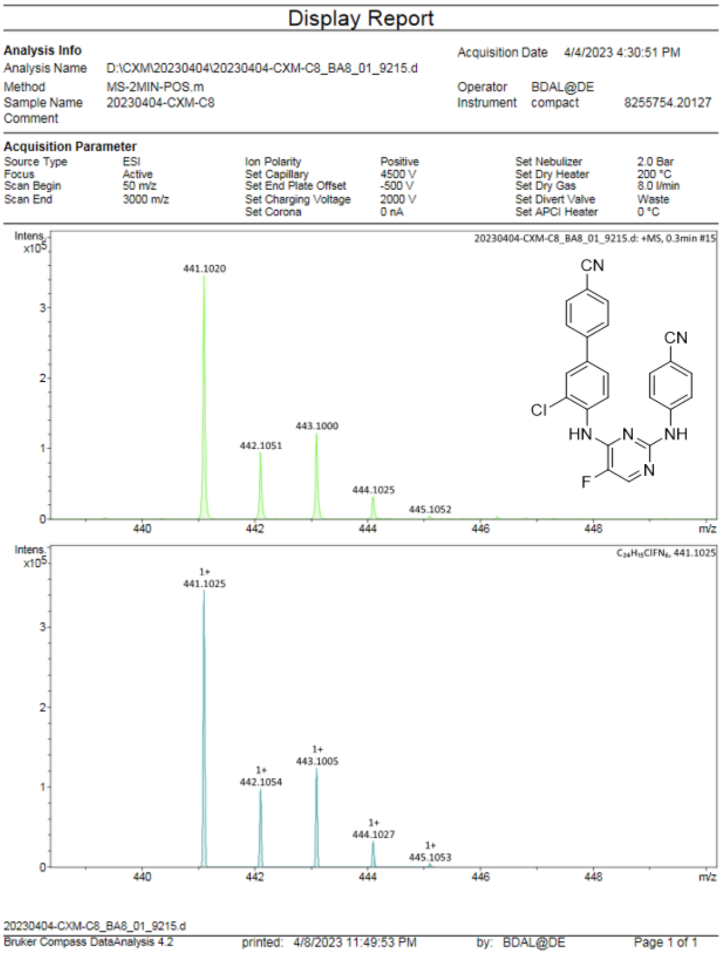

HPLC

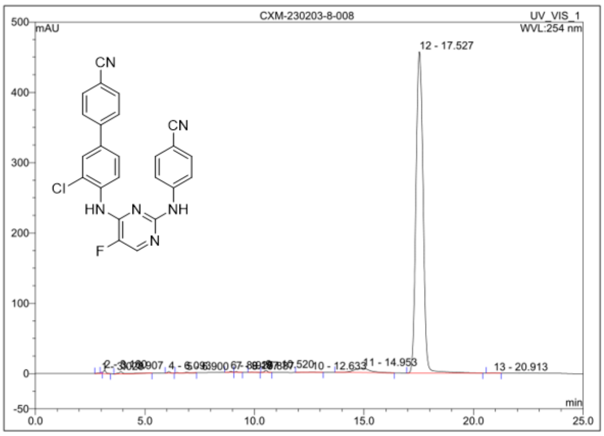

No.	Ret.Time min	Peak Name	Height mAU	Area mAU*s	Rel.Area %	Amount	Type
1	3.02	n.a.	0.437	1.51	0.01	n.a.	Ru
2	3.16	n.a.	4.660	52.89	0.49	n.a.	BMB
3	3.91	n.a.	3.364	59.72	0.55	n.a.	BMB
4	6.09	n.a.	1.934	15.74	0.14	n.a.	BMB
5	6.90	n.a.	0.745	8.62	0.08	n.a.	BMB*
6	8.93	n.a.	1.429	12.52	0.12	n.a.	BM
7	9.17	n.a.	0.896	8.91	0.08	n.a.	MB
8	9.89	n.a.	0.549	5.55	0.05	n.a.	BMB
9	10.52	n.a.	3.024	31.80	0.29	n.a.	BMB
10	12.63	n.a.	0.426	16.29	0.15	n.a.	BMB*
11	14.95	n.a.	6.275	320.90	2.95	n.a.	BMB*
12	17.53	n.a.	457.050	10335.27	95.04	n.a.	BMB*
13	20.91	n.a.	0.201	4.87	0.04	n.a.	BMB*
Total:			480.991	10874.579	100.00	0.000	

^{1}H NMR, ^{13}C NMR, ^{19}F NMR, HRMS, HPLC spectra of A9

^{1}H NMR

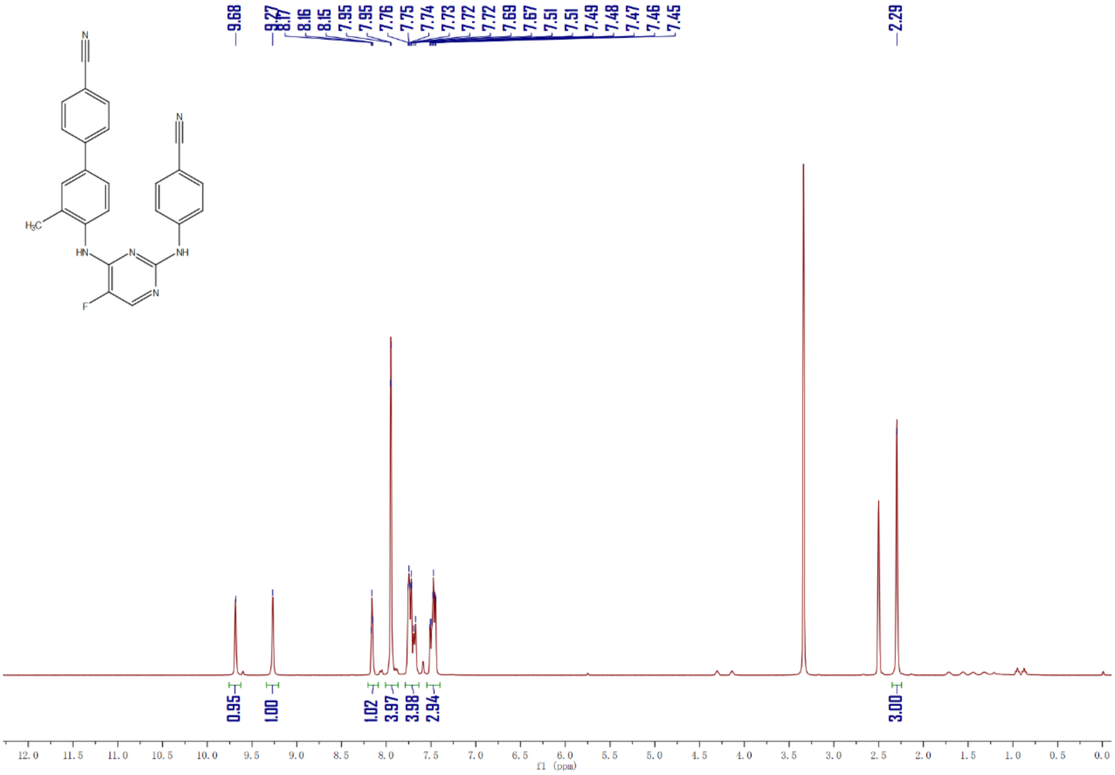

^{13}C NMR

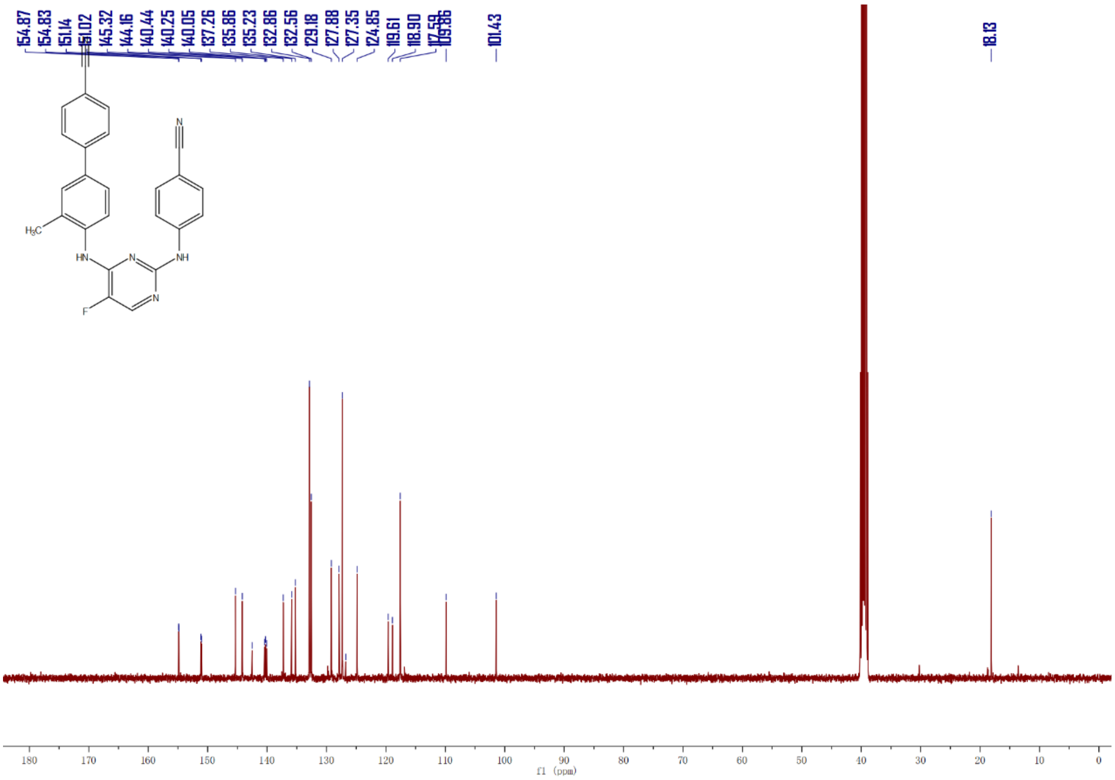

^{19}F NMR

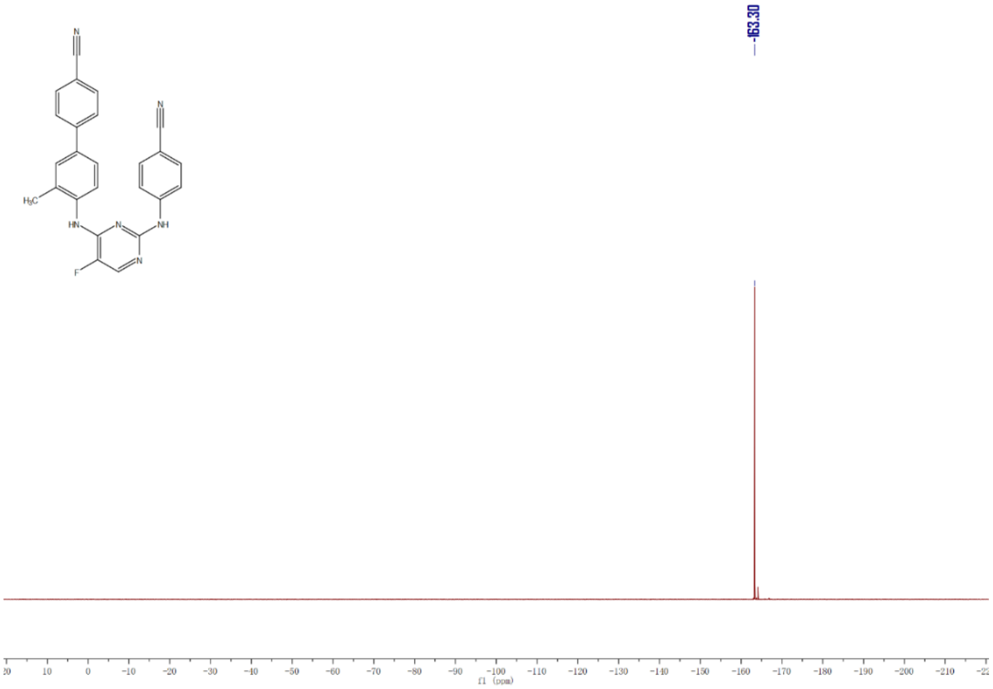

-163.30
f1 (ppm)

HRMS

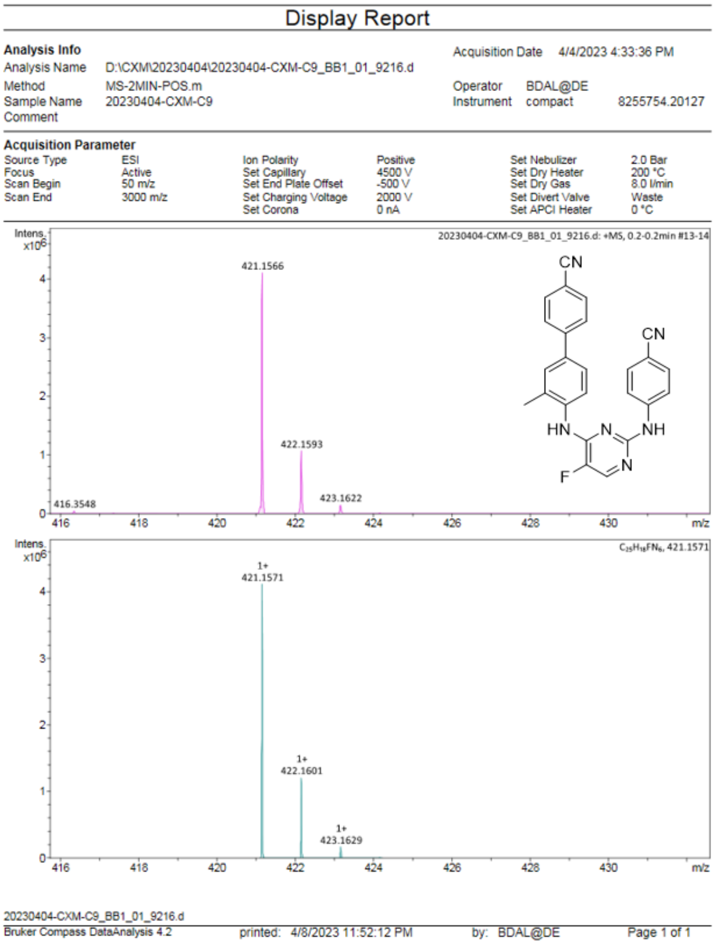

Display Report
Analysis Info
Analysis Name D:\CXM\20230404\20230404-CXM-C9_BB1_01_9216.d
Method MS-2MIN-POS.m
Sample Name 20230404-CXM-C9
Comment
Acquisition Date 4/4/2023 4:33:36 PM
Operator BDAL@DE
Instrument compact 8255754.20127
Acquisition Parameter
Source Type ESI
Focus Active
Scan Begin 50 m/z
Scan End 3000 m/z
Ion Polarity Positive
Set Capillary 4500 V
Set End Plate Offset -500 V
Set Charging Voltage 2000 V
Set Corona 0 nA
Set Nebulizer 2.0 Bar
Set Dry Heater 200 °C
Set Dry Gas 8.0 l/min
Set Divert Valve Waste
Set APCI Heater 0 °C
20230404-CXM-C9_BB1_01_9216.d: +MS, 0.2-0.2min #13-14
421.1566
422.1593
423.1622
416.3548
CN
HN
NH
N
F
C25H18FN6, 421.1571
421.1571
422.1601
423.1629
m/z
20230404-CXM-C9_BB1_01_9216.d
Bruker Compass DataAnalysis 4.2
printed: 4/8/2023 11:52:12 PM
by: BDAL@DE
Page 1 of 1

HPLC

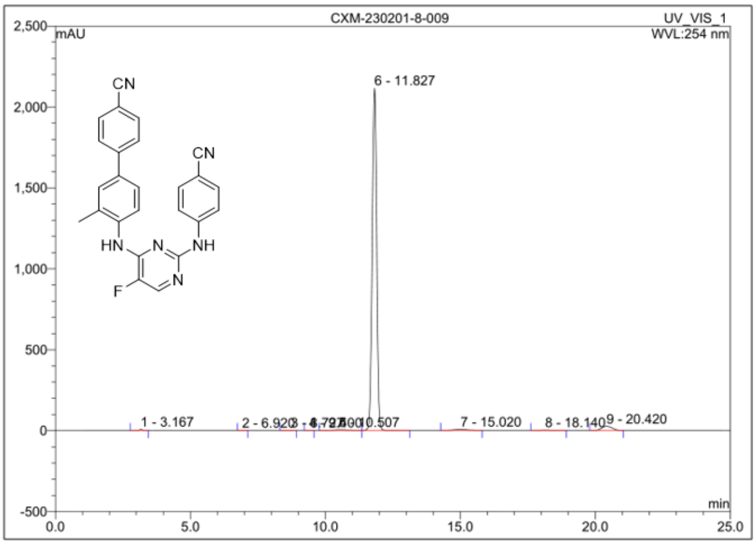

No.	Ret.Time min	Peak Name	Height mAU	Area mAU*s	Rel.Area %	Amount	Type
1	3.17	n.a.	6.618	56.31	0.22	n.a.	BMB
2	6.92	n.a.	0.580	4.85	0.02	n.a.	BMB
3	8.73	n.a.	0.323	3.51	0.01	n.a.	BMB*
4	9.40	n.a.	0.745	5.76	0.02	n.a.	BMB
5	10.51	n.a.	3.401	155.32	0.60	n.a.	BM
6	11.83	n.a.	2115.043	24572.53	95.32	n.a.	MB
7	15.02	n.a.	6.174	213.51	0.83	n.a.	BMB*
8	18.14	n.a.	2.758	66.13	0.26	n.a.	BMB*
9	20.42	n.a.	25.370	700.40	2.72	n.a.	BMB*
Total:			2161.014	25778.323	100.00	0.000	

^{1}H NMR, ^{13}C NMR, ^{19}F NMR, HRMS, HPLC spectra of A10

^{1}H NMR

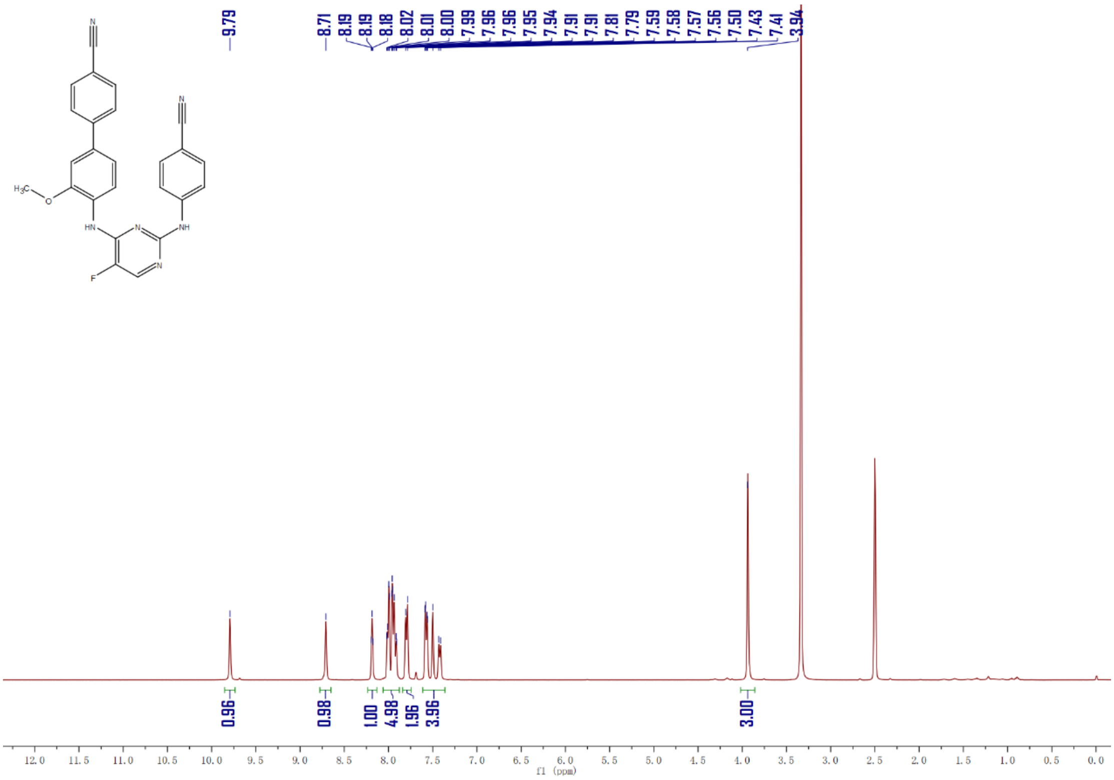

^{13}C NMR

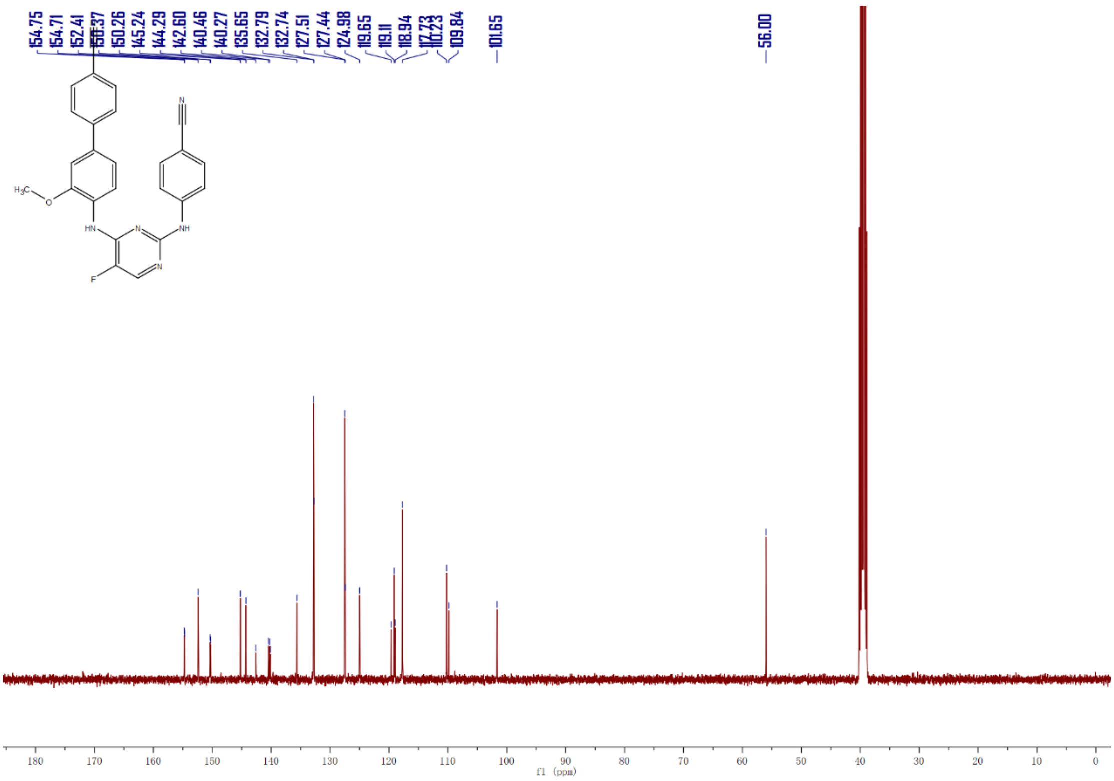

^{19}F NMR

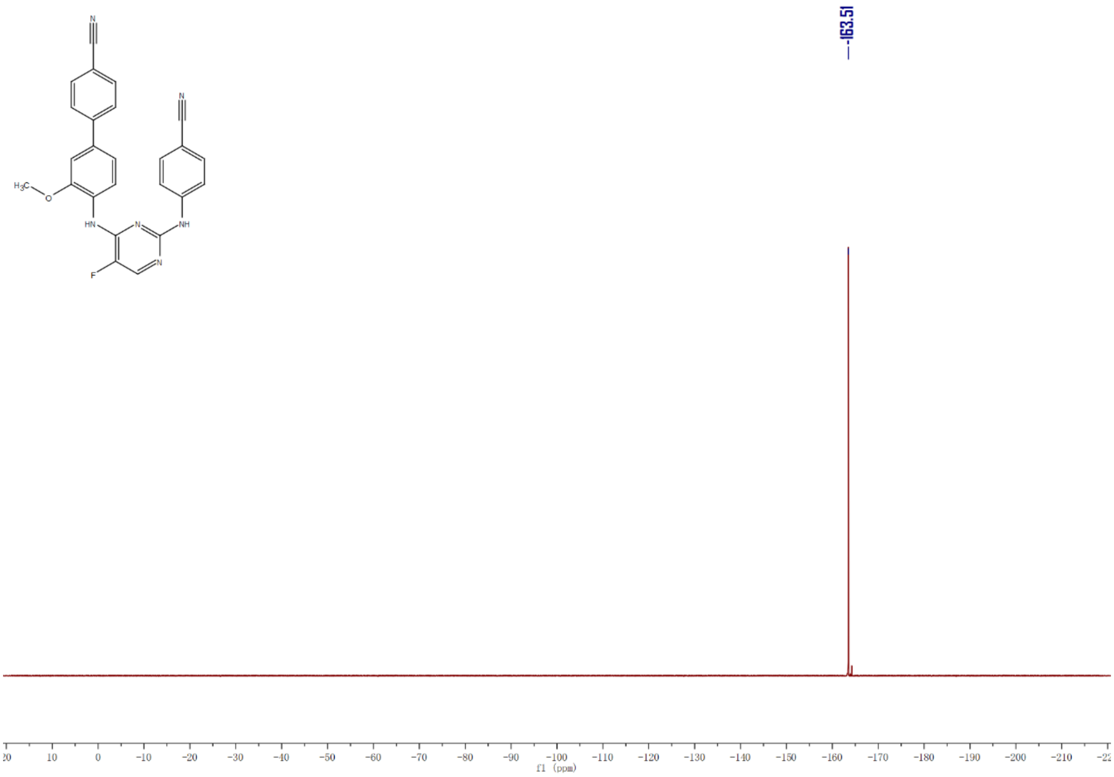

HRMS

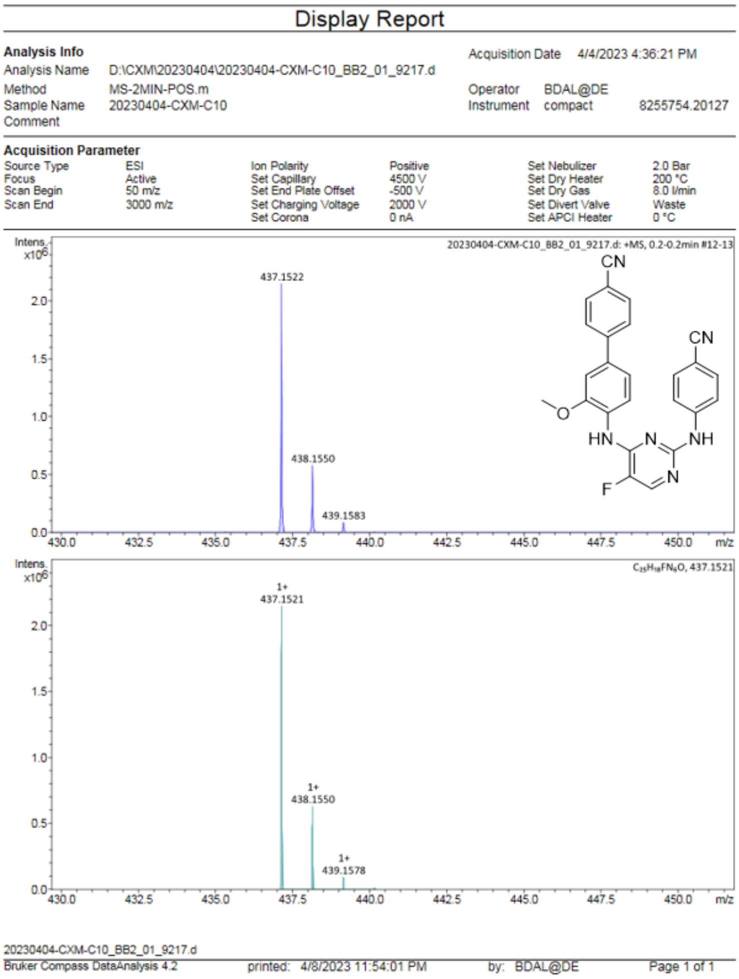

HPLC

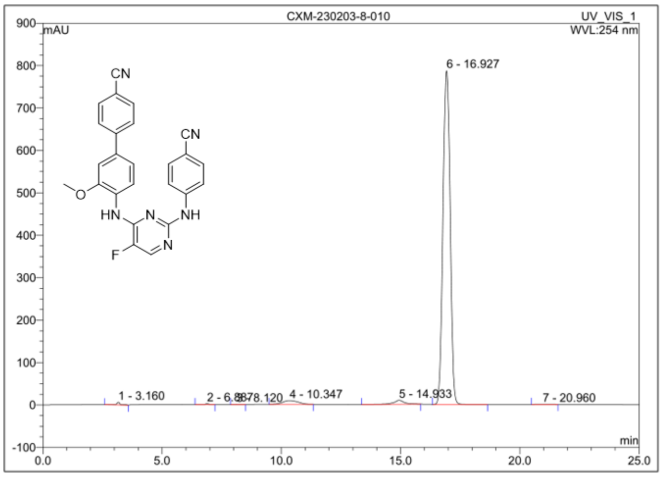

No.	Ret.Time min	Peak Name	Height mAU	Area mAU*s	Rel.Area %	Amount	Type
1	3.16	n.a.	6.406	72.74	0.42	n.a.	BMB
2	6.89	n.a.	1.938	16.06	0.09	n.a.	BMB*
3	8.12	n.a.	0.172	1.19	0.01	n.a.	BMB*
4	10.35	n.a.	8.814	395.79	2.29	n.a.	BMB*
5	14.93	n.a.	9.461	306.56	1.77	n.a.	BMB*
6	16.93	n.a.	786.893	16477.59	95.32	n.a.	BMB*
7	20.96	n.a.	0.487	17.20	0.10	n.a.	BMB*
Total:			814.170	17287.123	100.00	0.000	

^1H NMR, ^{13}C NMR, ^{19}F NMR, HRMS, HPLC spectra of A11

^1H NMR

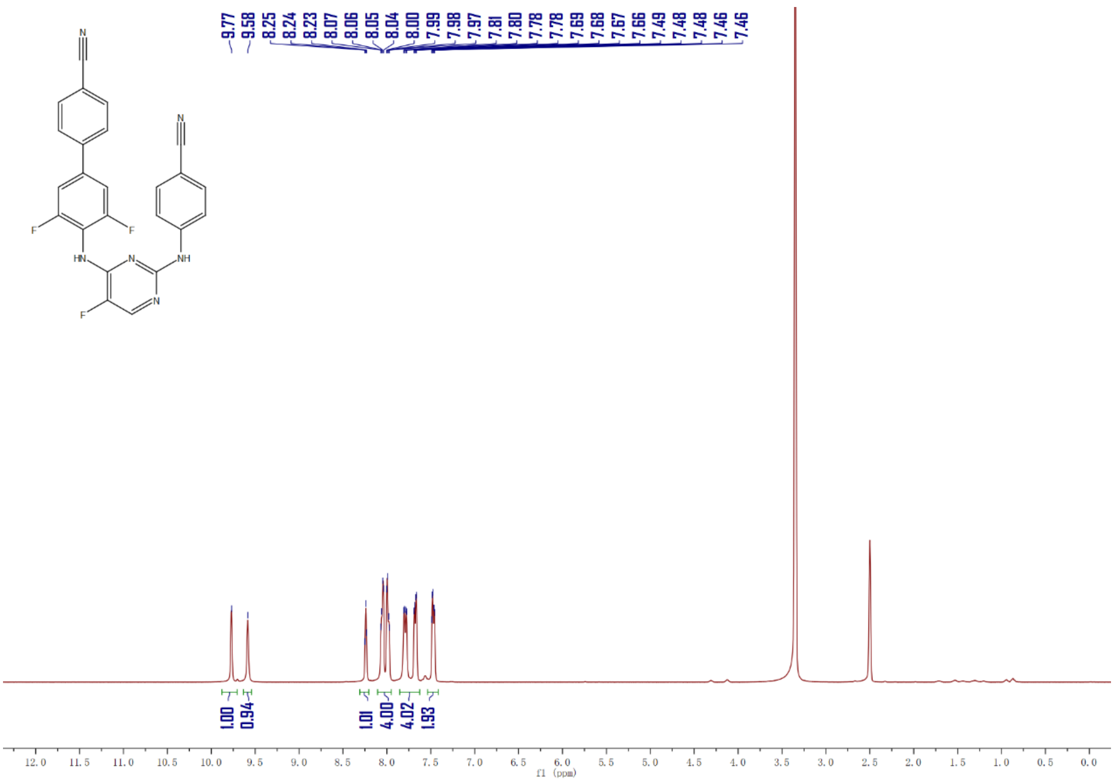

^{13}C NMR

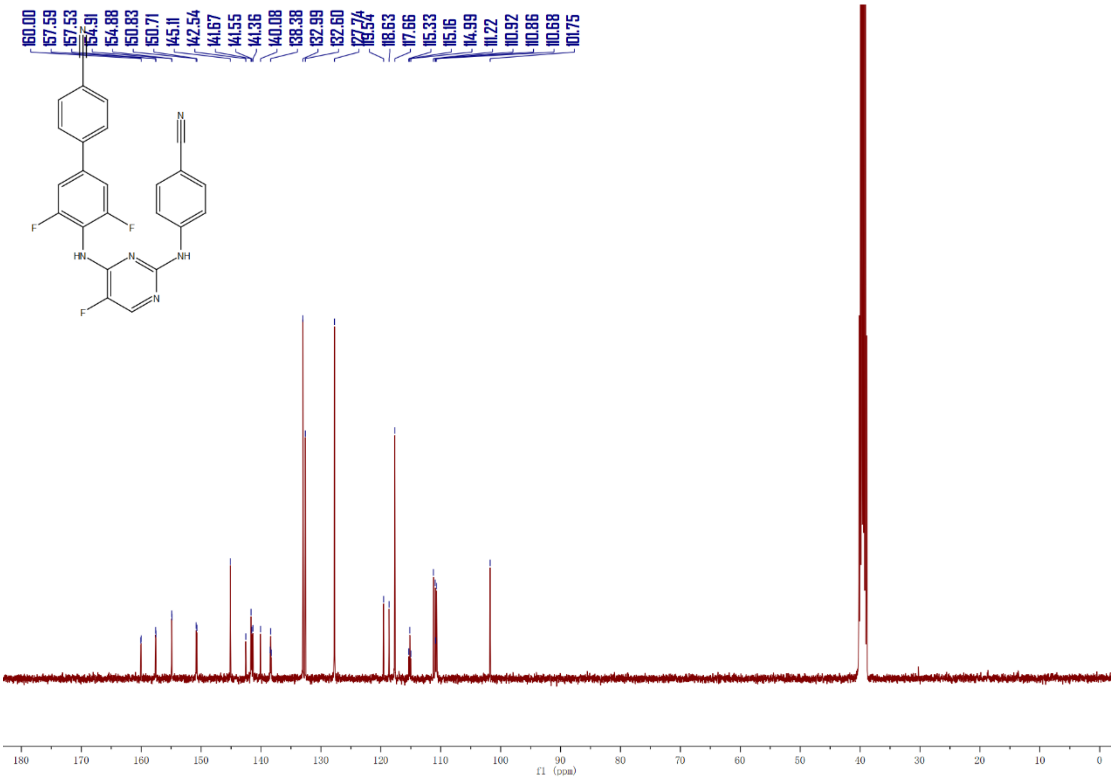

^{19}F NMR

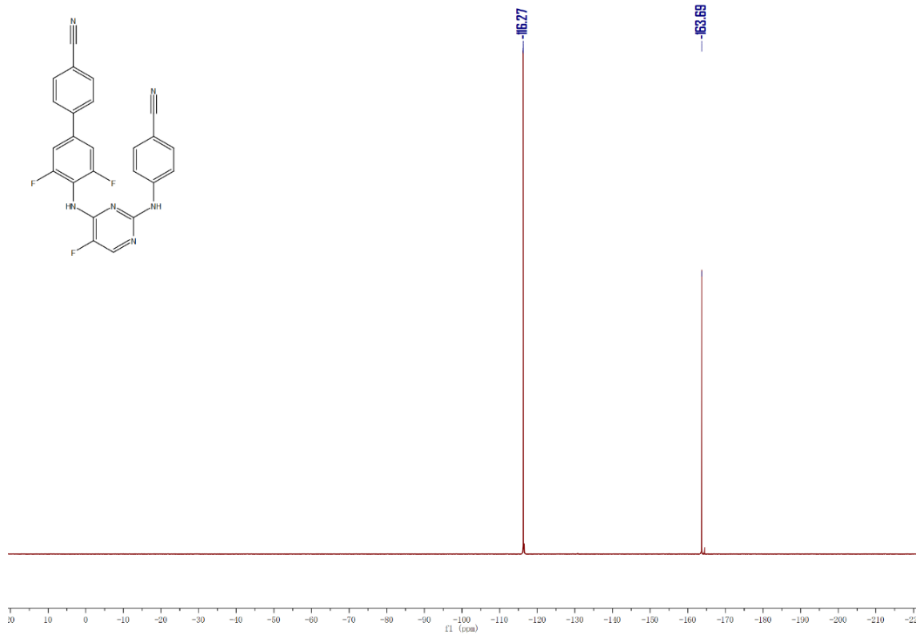

HRMS

Display Report

Analysis Info

Analysis Name	D:\CXM\20230404\20230404-CXM-C11_BB3_01_9219.d	Acquisition Date	4/4/2023 4:46:09 PM
Method	MS-2MIN-POS.m	Operator	BDAL@DE
Sample Name	20230404-CXM-C11	Instrument	compact 8255754.20127
Comment			

Acquisition Parameter

Source Type	ESI	Ion Polarity	Positive	Set Nebulizer	2.0 Bar
Focus	Active	Set Capillary	4500 V	Set Dry Heater	200 °C
Scan Begin	50 m/z	Set End Plate Offset	-500 V	Set Dry Gas	8.0 l/min
Scan End	3000 m/z	Set Charging Voltage	2000 V	Set Divert Valve	Waste
		Set Corona	0 nA	Set APCI Heater	0 °C

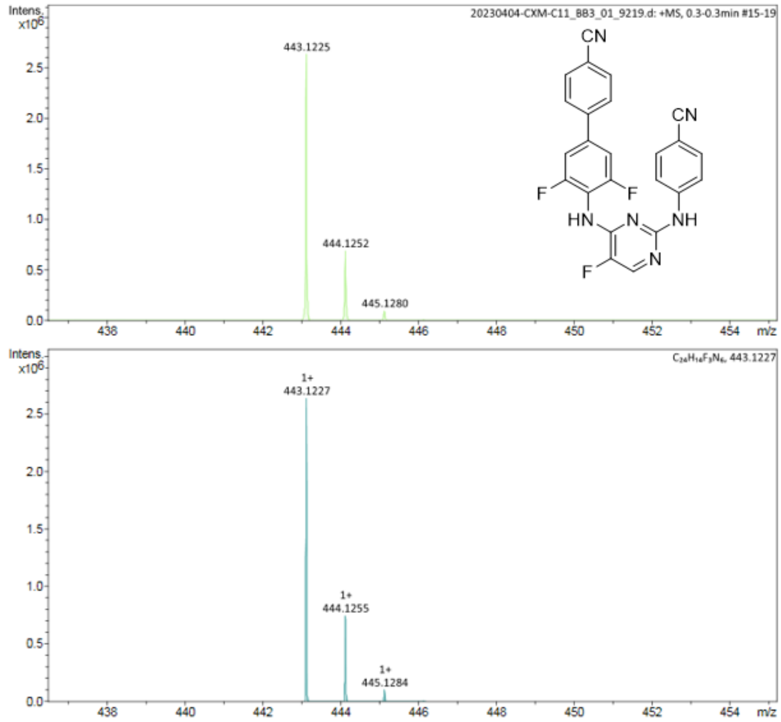

20230404-CXM-C11_BB3_01_9219.d

Bruker Compass DataAnalysis 4.2 printed: 4/8/2023 11:56:49 PM by: BDAL@DE Page 1 of 1

HPLC

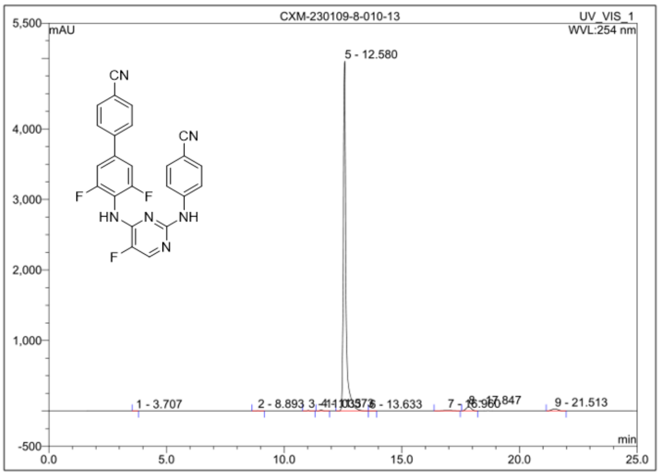

No.	Ret.Time min	Peak Name	Height mAU	Area mAU*s	Rel.Area %	Amount	Type
1	3.71	n.a.	1.223	9.52	0.02	n.a.	BMB*
2	8.89	n.a.	4.778	39.80	0.10	n.a.	BMB
3	11.03	n.a.	7.835	55.03	0.14	n.a.	BMB
4	11.57	n.a.	11.734	106.98	0.27	n.a.	BMB
5	12.58	n.a.	4961.022	37538.63	95.16	n.a.	BM *
6	13.63	n.a.	1.184	12.26	0.03	n.a.	MB
7	16.96	n.a.	7.152	233.69	0.59	n.a.	BMB*
8	17.85	n.a.	57.201	867.08	2.20	n.a.	BMB*
9	21.51	n.a.	29.253	585.52	1.48	n.a.	BMB*
Total:			5081.383	39448.497	100.00	0.000	

^{1}H NMR, ^{13}C NMR, ^{19}F NMR, HRMS, HPLC spectra of A12

^{1}H NMR

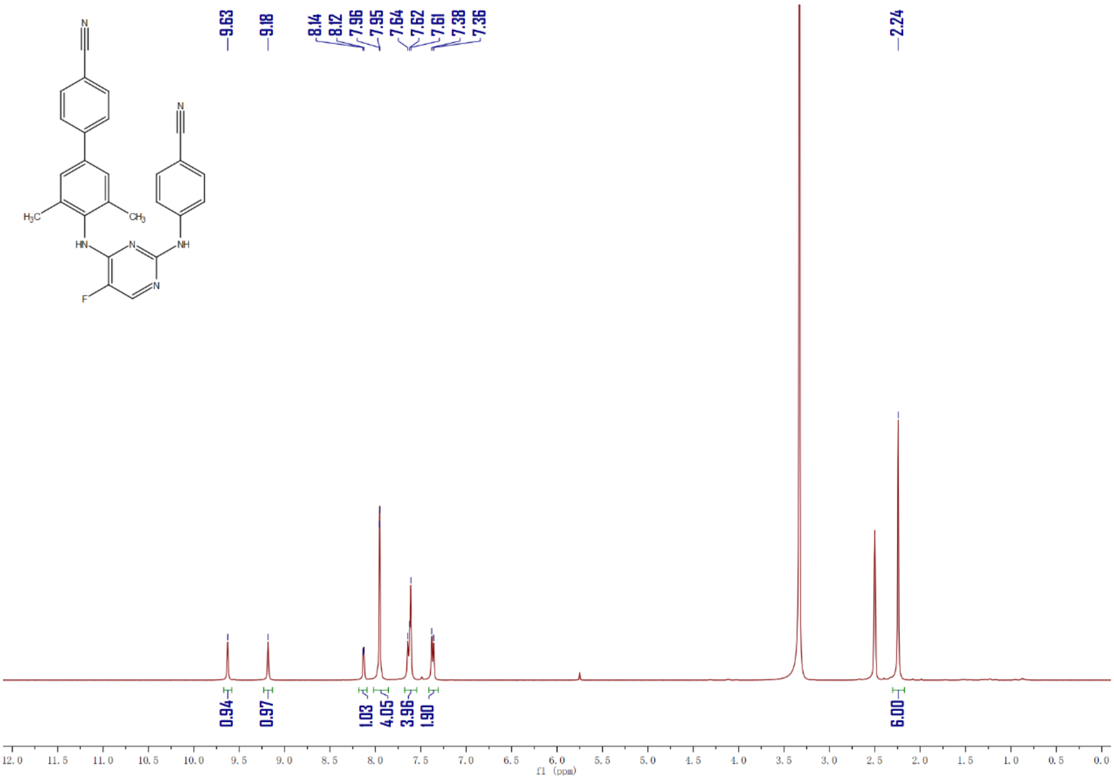

^{13}C NMR

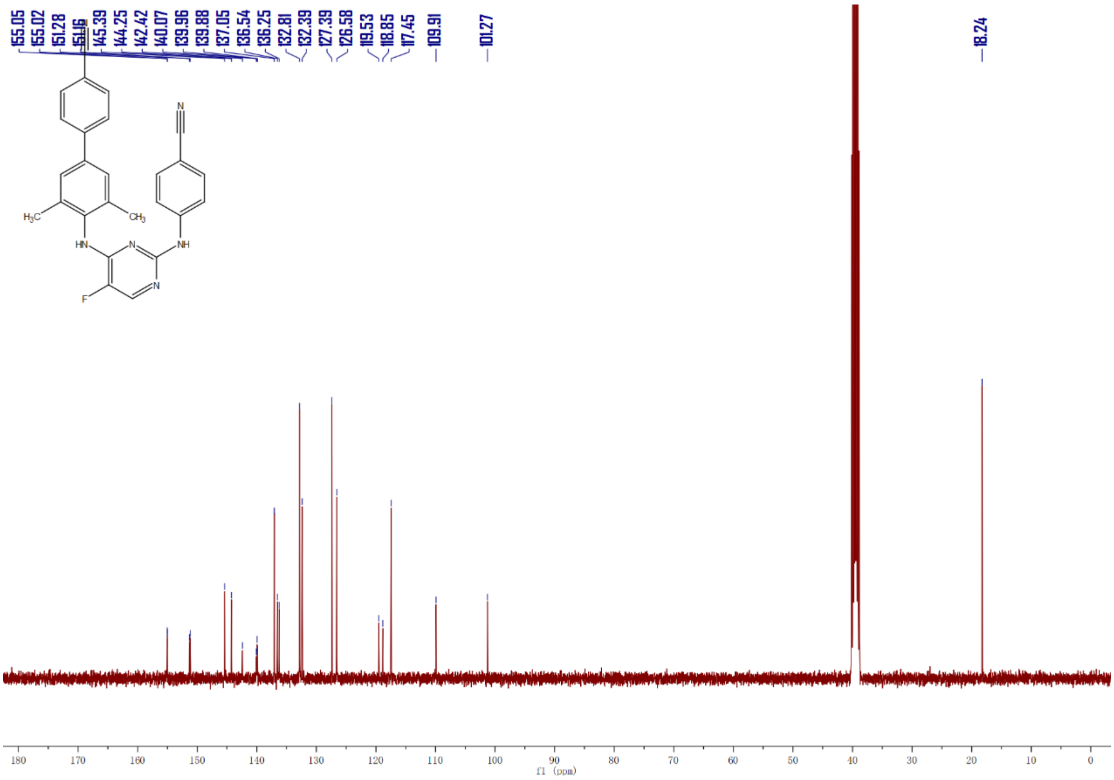

^{19}F NMR

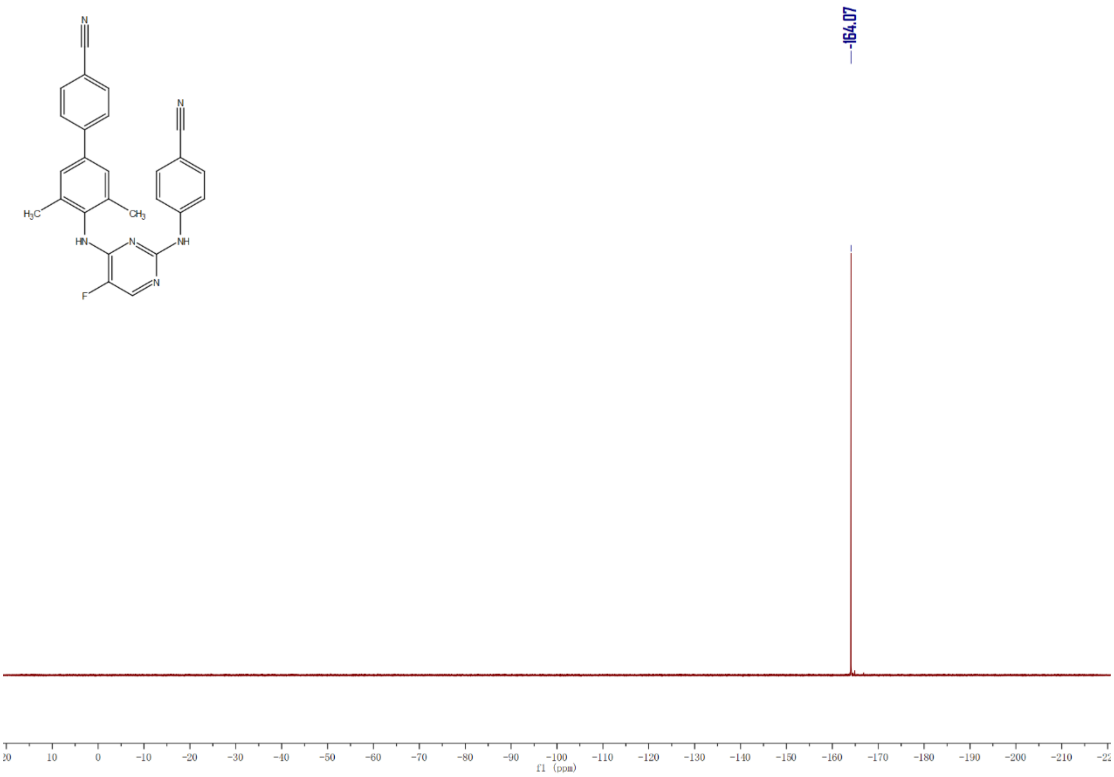

HRMS

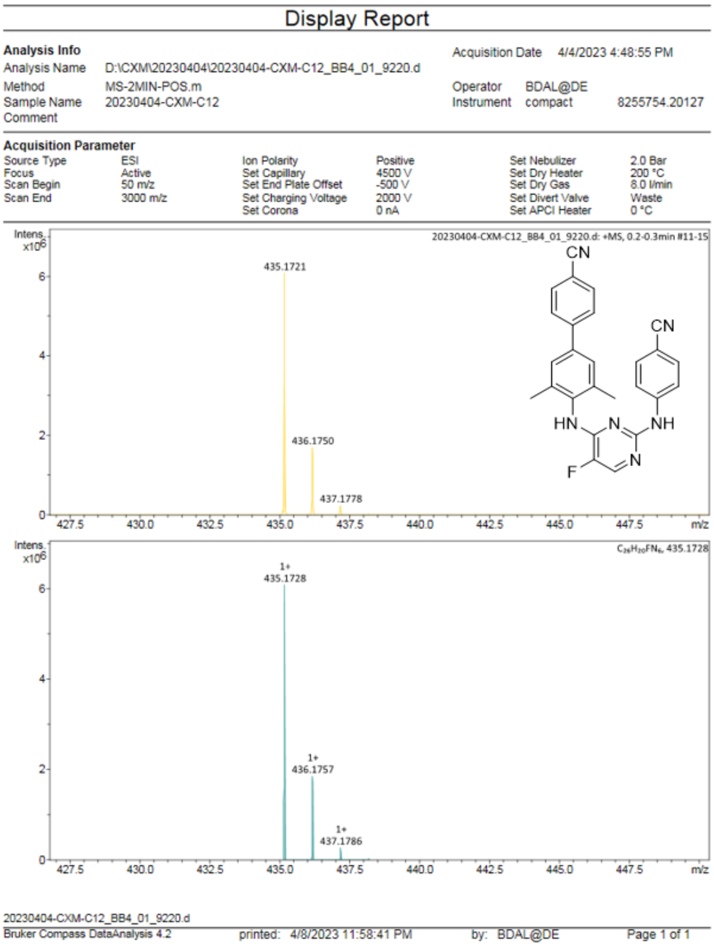

HPLC

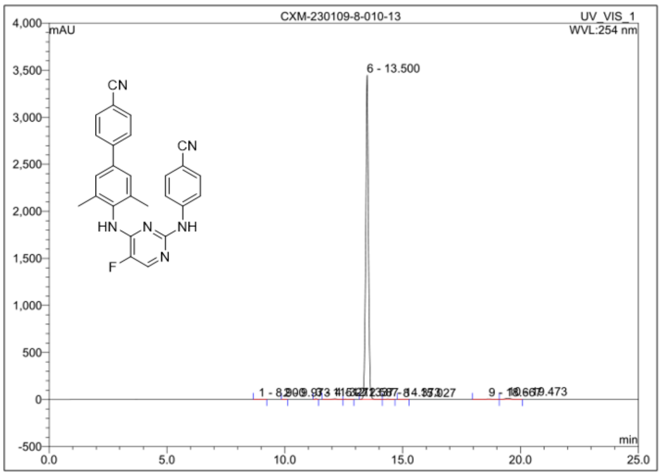

No.	Ret.Time min	Peak Name	Height mAU	Area mAU*s	Rel.Area %	Amount	Type
1	8.90	n.a.	2.182	18.55	0.07	n.a.	BMB
2	9.97	n.a.	1.509	9.77	0.04	n.a.	BMB
3	11.33	n.a.	5.278	31.87	0.11	n.a.	BMB
4	12.13	n.a.	4.213	77.75	0.28	n.a.	BM
5	12.59	n.a.	2.086	25.98	0.09	n.a.	MB
6	13.50	n.a.	3447.648	27348.94	98.16	n.a.	BMB
7	14.37	n.a.	3.491	33.73	0.12	n.a.	BMB
8	15.03	n.a.	0.765	9.14	0.03	n.a.	BMB
9	18.67	n.a.	4.556	91.60	0.33	n.a.	BM
10	19.47	n.a.	11.940	213.45	0.77	n.a.	MB
Total:			3483.667	27860.789	100.00	0.000	

^{1}H NMR, ^{13}C NMR, ^{19}F NMR, HRMS, HPLC spectra of A13

^{1}H NMR

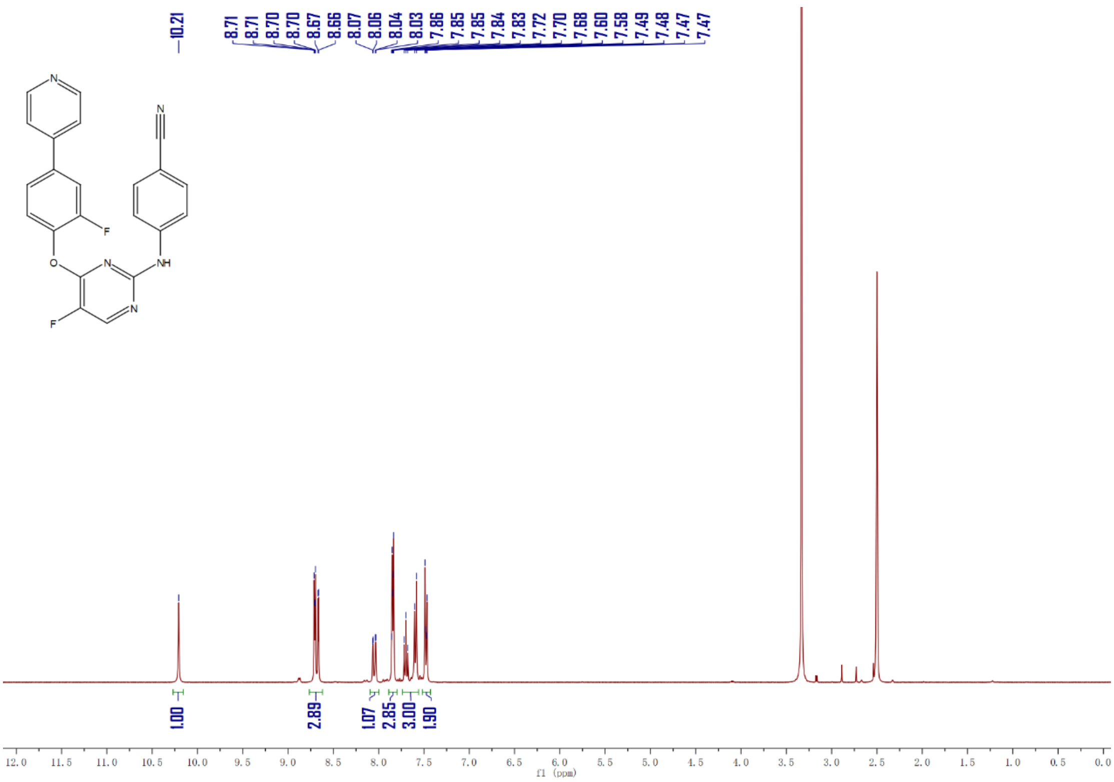

^{13}C NMR

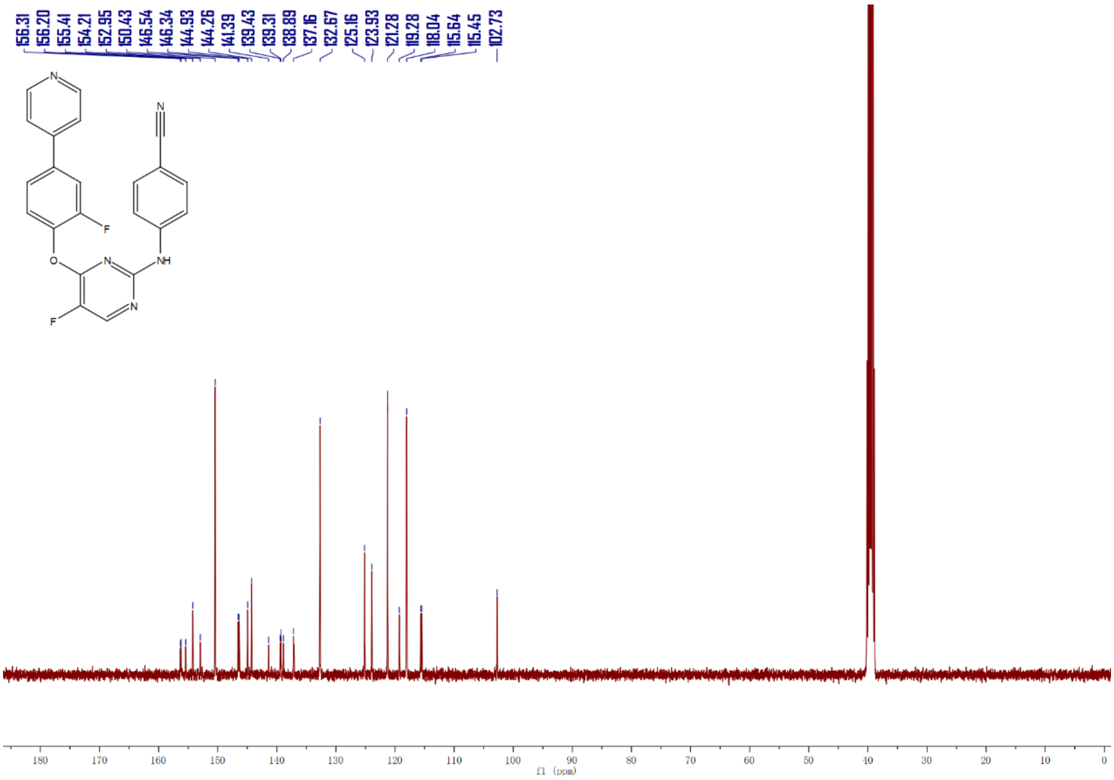

^{19}F NMR

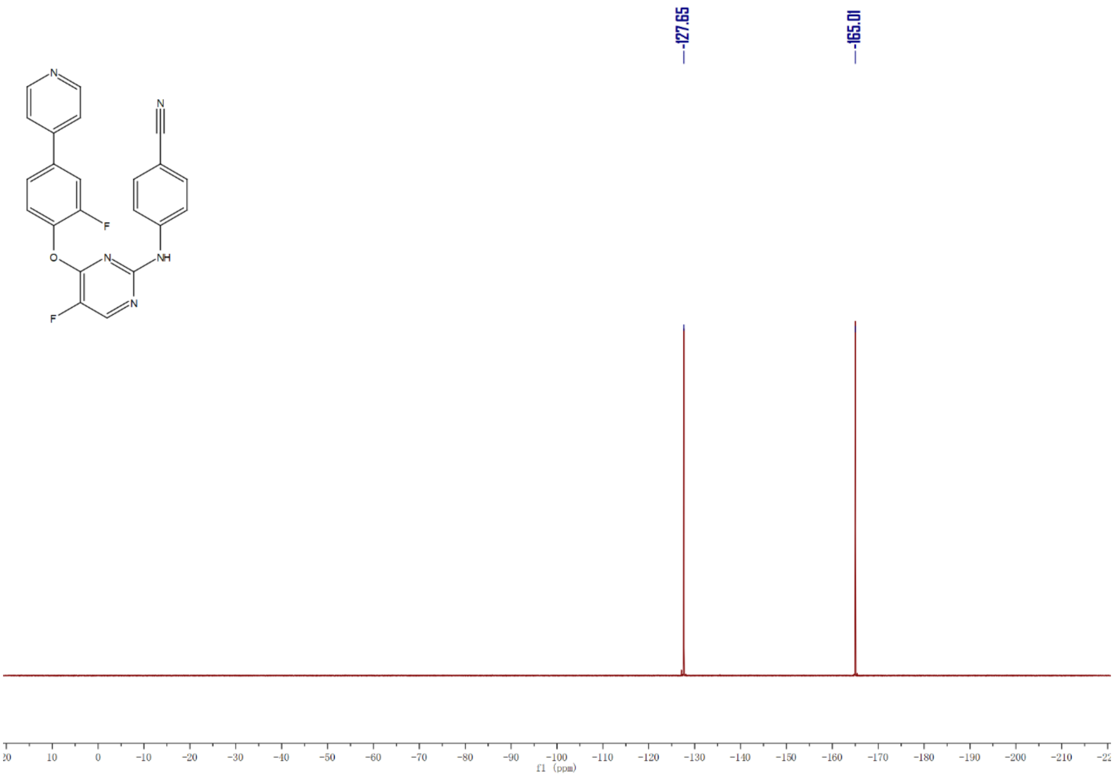

-127.65
-165.01
N
F
O
N
NH
N
F
N
20 10 0 -10 -20 -30 -40 -50 -60 -70 -80 -90 -100 -110 -120 -130 -140 -150 -160 -170 -180 -190 -200 -210
f1 (ppm)

HRMS

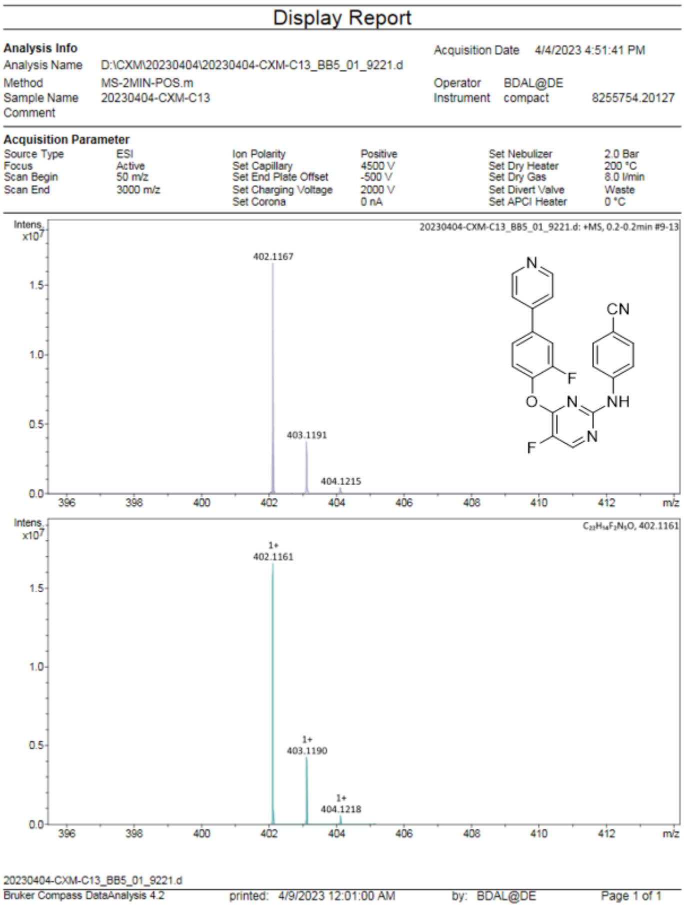

Display Report
Analysis Info
Analysis Name D:\CXM\20230404\20230404-CXM-C13_BB5_01_9221.d
Method MS-2MIN-POS.m
Sample Name 20230404-CXM-C13
Comment
Acquisition Date 4/4/2023 4:51:41 PM
Operator BDAL@DE
Instrument compact 8255754.20127
Acquisition Parameter
Source Type ESI
Focus Active
Scan Begin 50 m/z
Scan End 3000 m/z
Ion Polarity Positive
Set Capillary 4500 V
Set End Plate Offset -500 V
Set Charging Voltage 2000 V
Set Corona 0 nA
Set Nebulizer 2.0 Bar
Set Dry Heater 200 °C
Set Dry Gas 8.0 l/min
Set Divert Valve Waste
Set APCI Heater 0 °C
Intens. x10^7
20230404-CXM-C13_BB5_01_9221.d: +MS, 0.2-0.2min #9-13
402.1167
403.1191
404.1215
N
CN
F
O
N
NH
F
N
m/z
C22H14F2N5O, 402.1161
1+
402.1161
1+
403.1190
1+
404.1218
20230404-CXM-C13_BB5_01_9221.d
Bruker Compass DataAnalysis 4.2
printed: 4/9/2023 12:01:00 AM
by: BDAL@DE
Page 1 of 1

HPLC

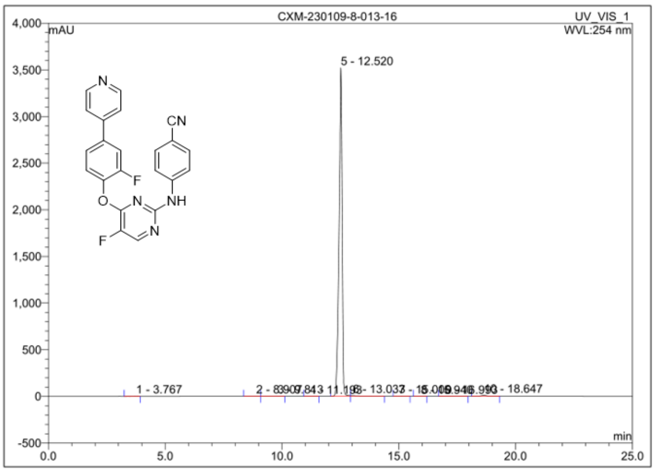

No.	Ret.Time min	Peak Name	Height mAU	Area mAU*s	Rel.Area %	Amount	Type
1	3.77	n.a.	4.338	56.32	0.18	n.a.	BMB*
2	8.91	n.a.	3.025	25.50	0.08	n.a.	BM
3	9.81	n.a.	1.414	21.17	0.07	n.a.	MB
4	11.19	n.a.	0.322	5.47	0.02	n.a.	BMB
5	12.52	n.a.	3522.678	31689.25	98.50	n.a.	BM
6	13.03	n.a.	6.285	154.26	0.48	n.a.	MB*
7	15.00	n.a.	2.274	26.90	0.08	n.a.	BMB
8	15.94	n.a.	0.423	6.09	0.02	n.a.	BMB
9	16.99	n.a.	0.552	11.95	0.04	n.a.	BMB
10	18.65	n.a.	9.745	175.22	0.54	n.a.	BMB
Total:			3551.057	32172.117	100.00	0.000	

^{1}H NMR, ^{13}C NMR, ^{19}F NMR, HRMS, HPLC spectra of A14

^{1}H NMR

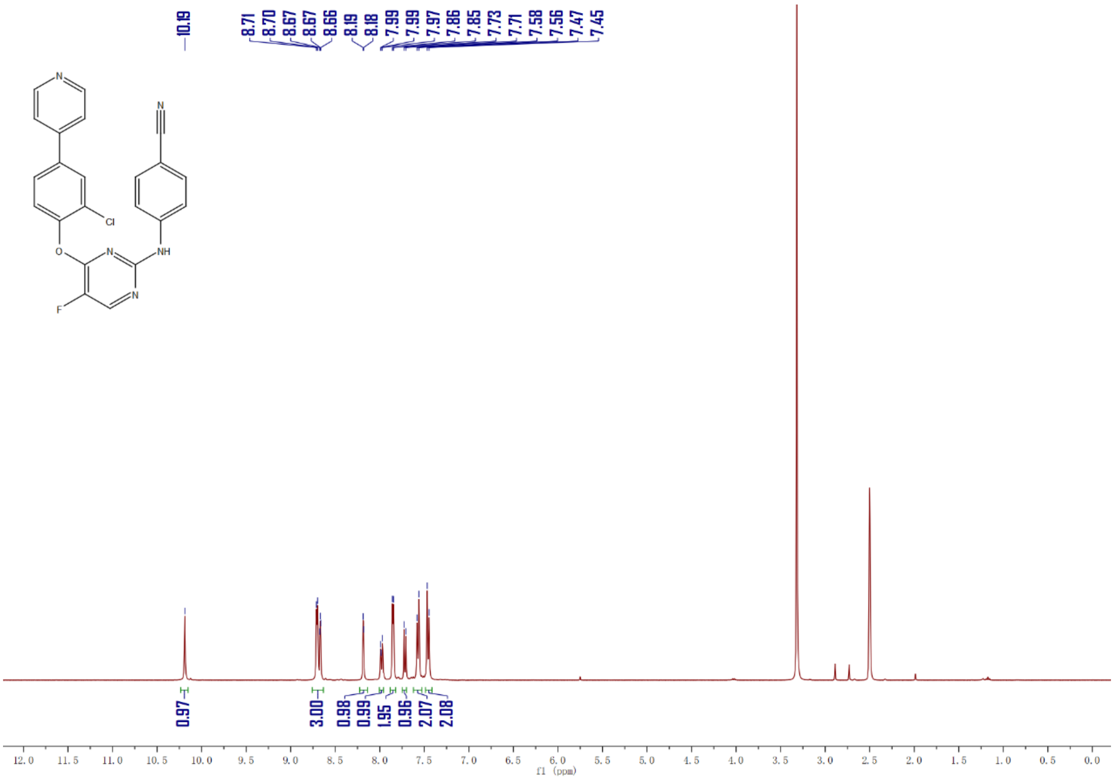

^{13}C NMR

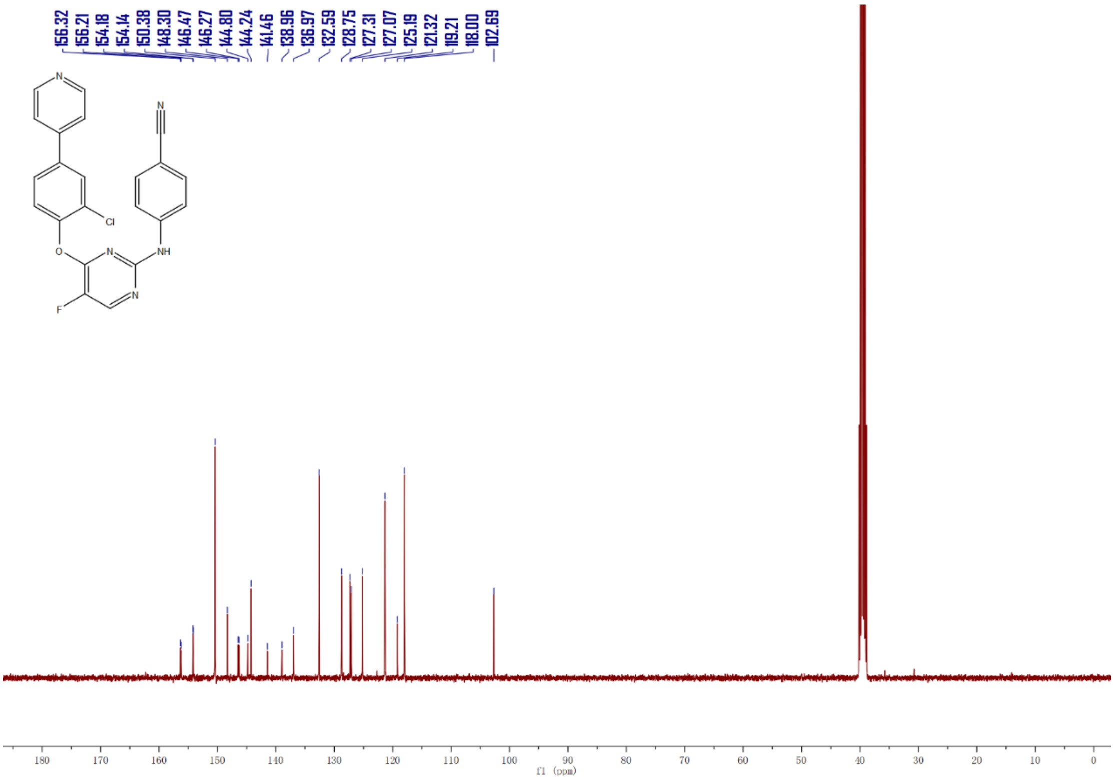

^{19}F NMR

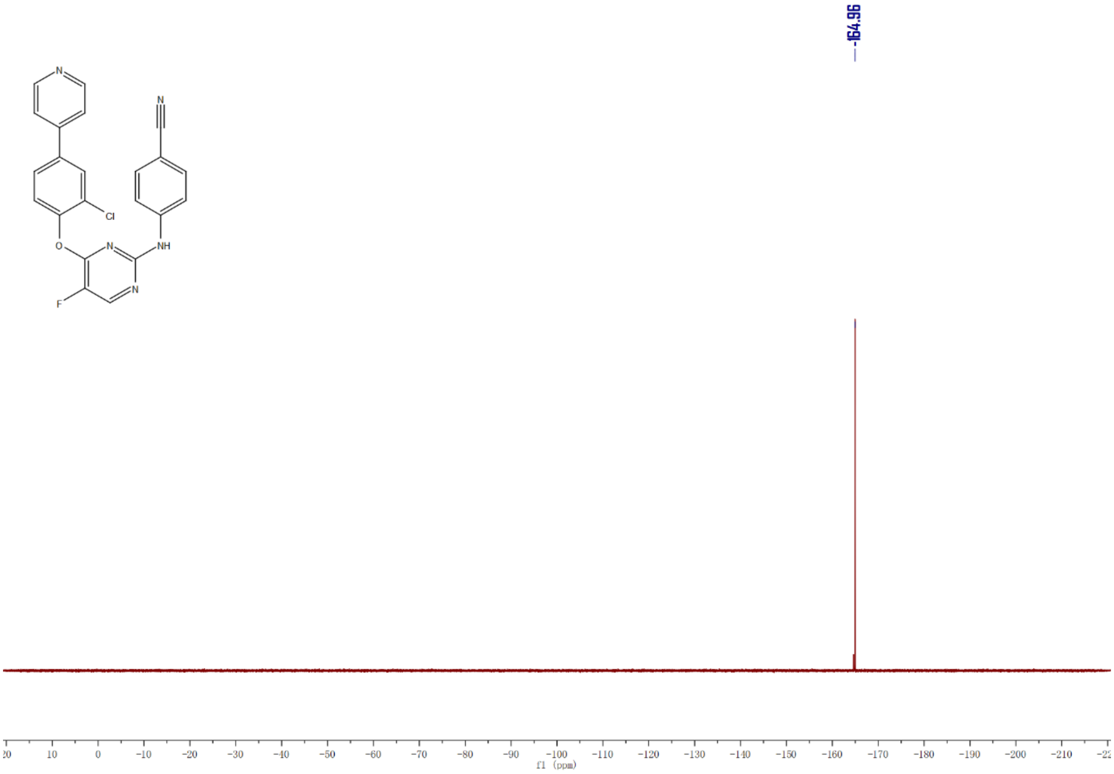

HRMS

Display Report

Analysis Info

Analysis Name D:\CXM\20230404\20230404-CXM-C14_BB6_01_9222.d
Method MS-2MIN-POS.m
Sample Name 20230404-CXM-C14
Comment

Acquisition Date 4/4/2023 4:54:27 PM
Operator BDAL@DE
Instrument compact 8255754.20127

Acquisition Parameter

Source Type	ESI	Ion Polarity	Positive	Set Nebulizer	2.0 Bar
Focus	Active	Set Capillary	4500 V	Set Dry Heater	200 °C
Scan Begin	50 m/z	Set End Plate Offset	-500 V	Set Dry Gas	8.0 l/min
Scan End	3000 m/z	Set Charging Voltage	2000 V	Set Divert Valve	Waste
		Set Corona	0 nA	Set APCI Heater	0 °C

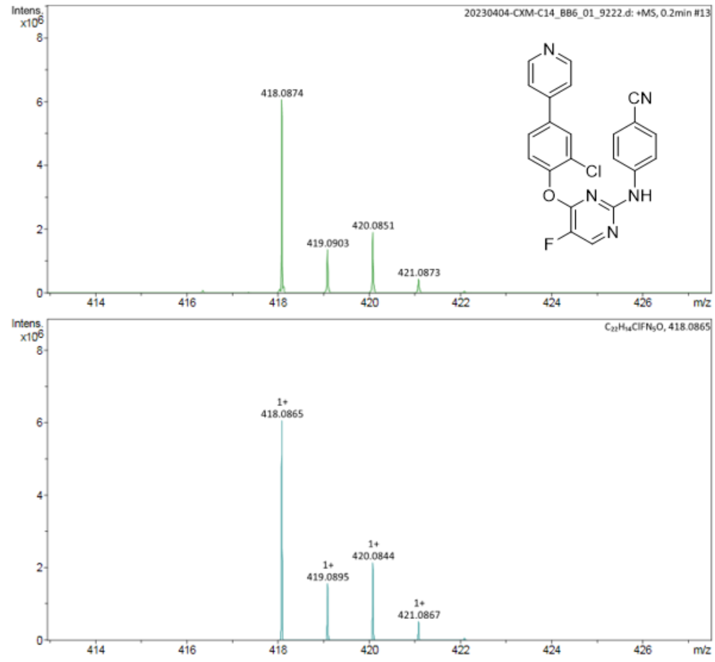

20230404-CXM-C14_BB6_01_9222.d
Bruker Compass DataAnalysis 4.2 printed: 4/9/2023 12:09:09 AM by: BDAL@DE Page 1 of 1

HPLC

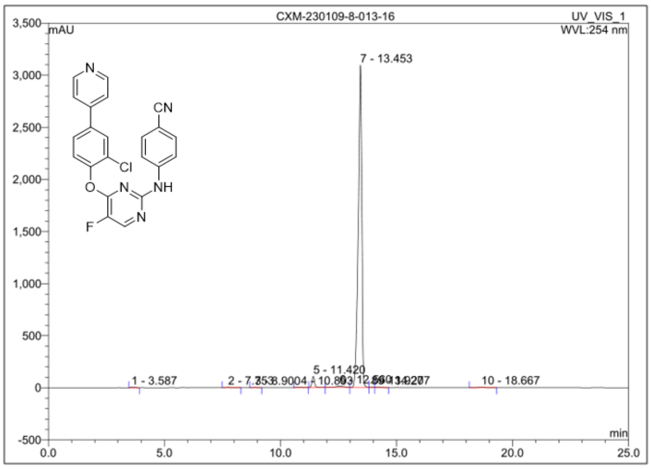

No.	Ret.Time min	Peak Name	Height mAU	Area mAU*s	Rel.Area %	Amount	Type
1	3.59	n.a.	4.852	72.49	0.21	n.a.	BMB*
2	7.75	n.a.	4.898	43.10	0.12	n.a.	BMB
3	8.90	n.a.	2.861	24.02	0.07	n.a.	BMB
4	10.89	n.a.	2.663	46.70	0.14	n.a.	BMB*
5	11.42	n.a.	107.188	846.04	2.45	n.a.	BMb*
6	12.56	n.a.	13.017	324.30	0.94	n.a.	bM
7	13.45	n.a.	3093.505	32994.27	95.45	n.a.	M
8	13.93	n.a.	5.196	60.48	0.17	n.a.	M
9	14.21	n.a.	5.127	62.31	0.18	n.a.	MB
10	18.67	n.a.	4.963	93.91	0.27	n.a.	BMB
Total:			3244.269	34567.620	100.00	0.000	

^{1}H NMR, ^{13}C NMR, ^{19}F NMR, HRMS, HPLC spectra of A15

^{1}H NMR

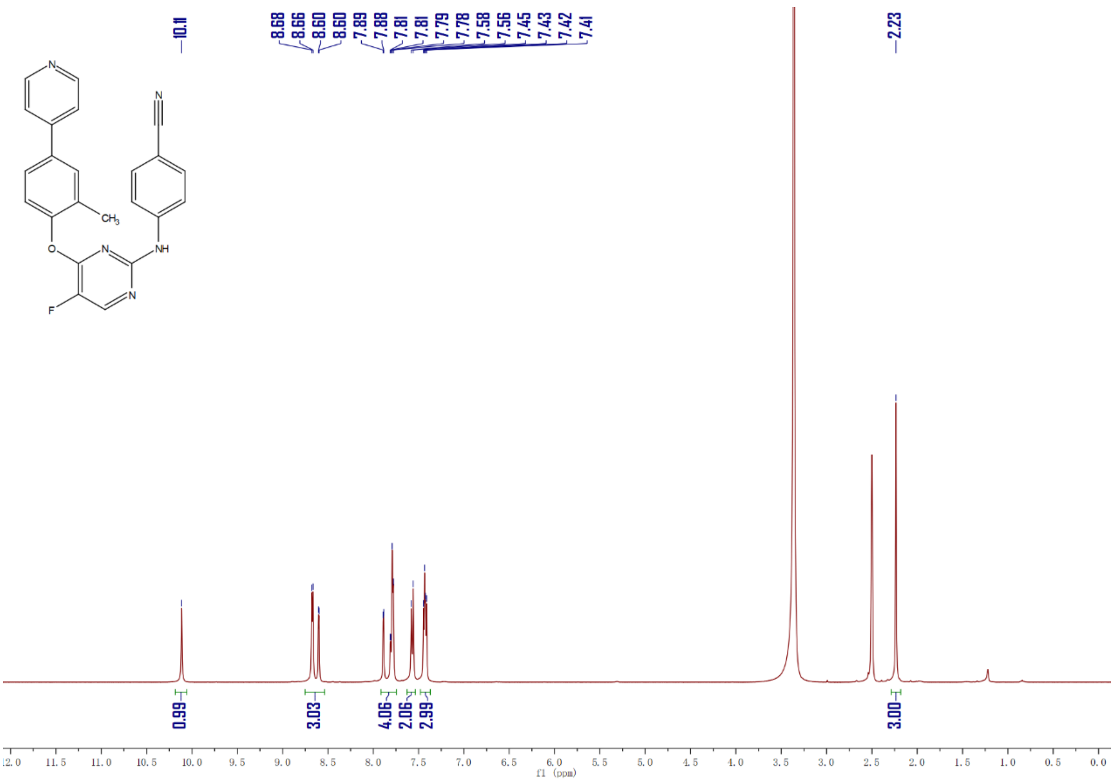

^{13}C NMR

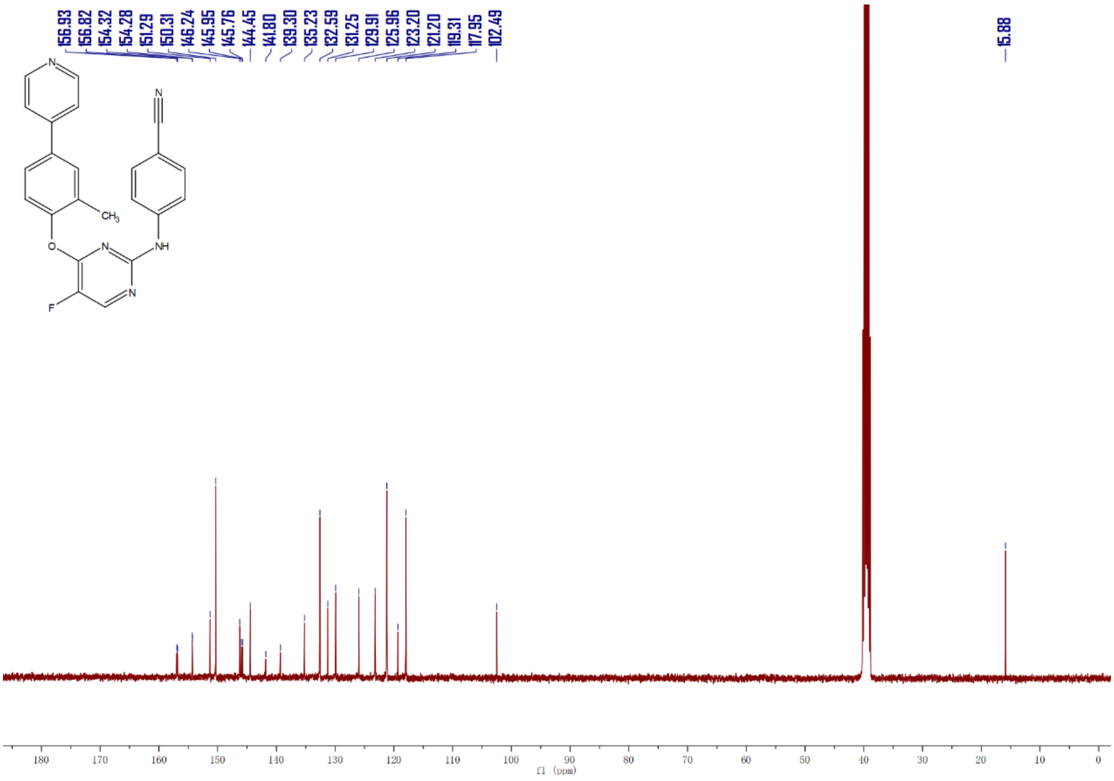

^{19}F NMR

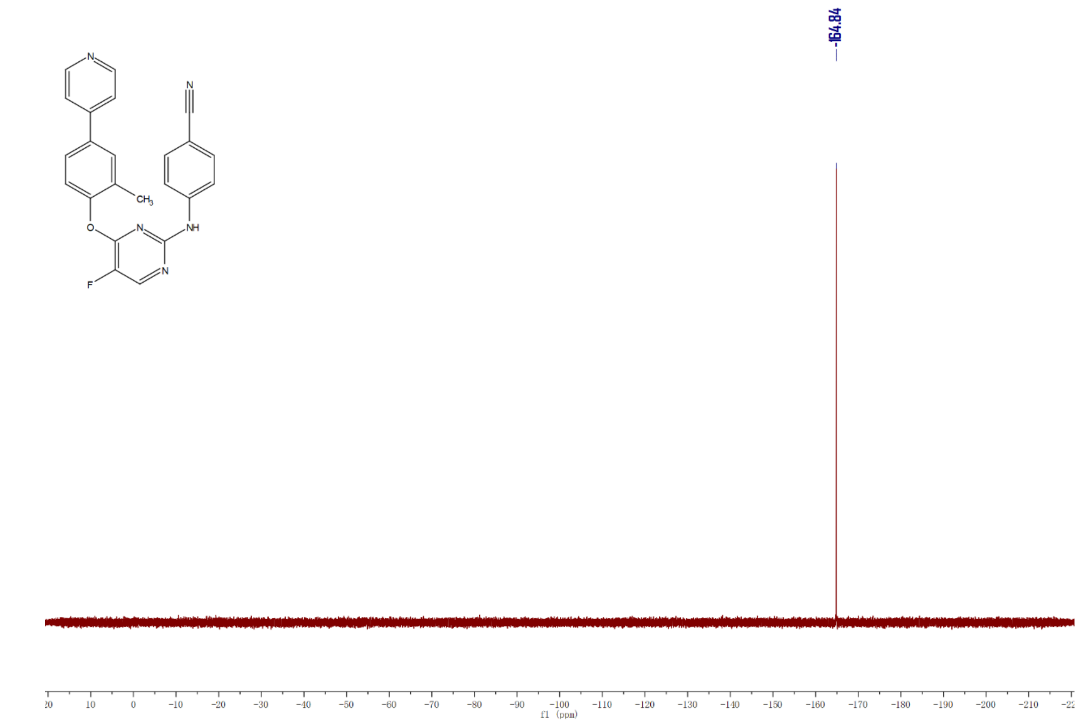

-164.84
N
NH
CH3
O
F
f1 (ppm)

HRMS

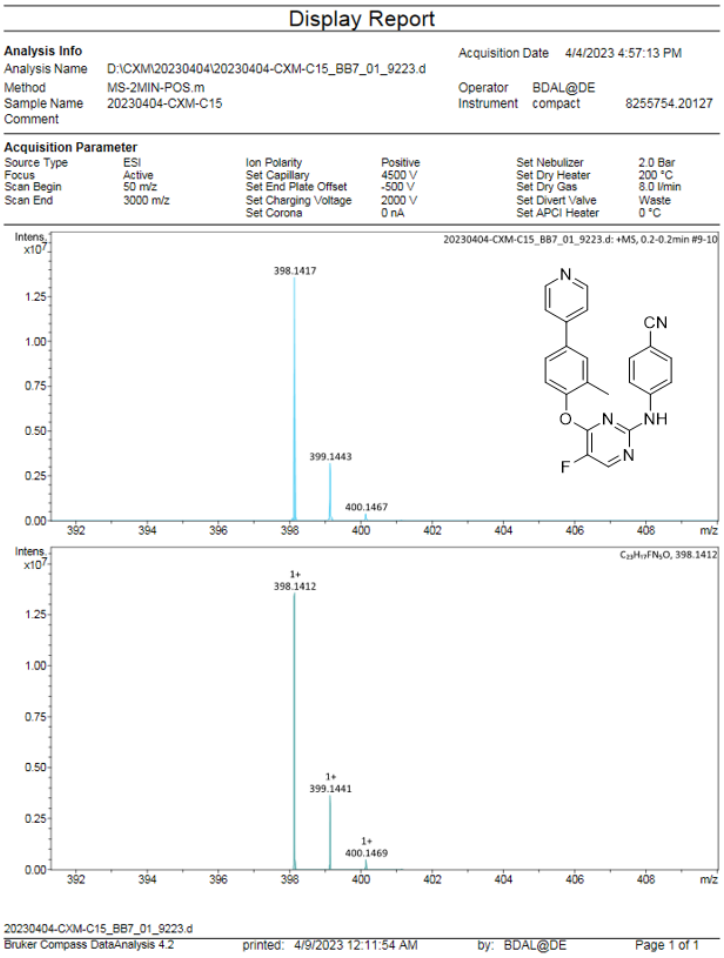

Display Report
Analysis Info
Analysis Name D:\CXM\20230404\20230404-CXM-C15_BB7_01_9223.d
Method MS-2MIN-POS.m
Sample Name 20230404-CXM-C15
Comment
Acquisition Date 4/4/2023 4:57:13 PM
Operator BDAL@DE
Instrument compact 8255754.20127
Acquisition Parameter
Source Type ESI
Focus Active
Scan Begin 50 m/z
Scan End 3000 m/z
Ion Polarity Positive
Set Capillary 4500 V
Set End Plate Offset -500 V
Set Charging Voltage 2000 V
Set Corona 0 nA
Set Nebulizer 2.0 Bar
Set Dry Heater 200 °C
Set Dry Gas 8.0 l/min
Set Divert Valve Waste
Set APCI Heater 0 °C
Intens. x10^7
20230404-CXM-C15_BB7_01_9223.d: +MS, 0.2-0.2min #9-10
398.1417
399.1443
400.1467
m/z
C23H17FN5O, 398.1412
1+ 398.1412
1+ 399.1441
1+ 400.1469
20230404-CXM-C15_BB7_01_9223.d
Bruker Compass DataAnalysis 4.2 printed: 4/9/2023 12:11:54 AM by: BDAL@DE Page 1 of 1

HPLC

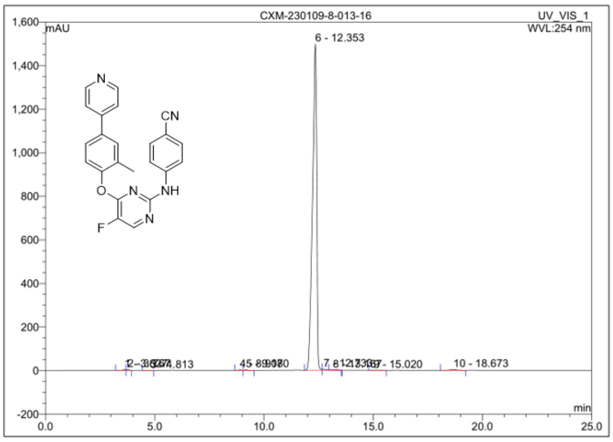

No.	Ret.Time min	Peak Name	Height mAU	Area mAU*s	Rel.Area %	Amount	Type
1	3.63	n.a.	3.807	31.22	0.15	n.a.	BM
2	3.77	n.a.	4.633	39.43	0.19	n.a.	MB
3	4.81	n.a.	0.540	5.72	0.03	n.a.	BMB
4	8.91	n.a.	2.986	22.74	0.11	n.a.	BM
5	9.18	n.a.	2.935	23.16	0.11	n.a.	MB
6	12.35	n.a.	1497.144	20088.74	98.53	n.a.	BM
7	12.73	n.a.	4.515	75.47	0.37	n.a.	MB
8	13.17	n.a.	0.737	16.23	0.08	n.a.	Rd
9	15.02	n.a.	0.720	11.49	0.06	n.a.	BMB
10	18.67	n.a.	3.966	74.25	0.36	n.a.	BMB
Total:			1521.983	20388.443	100.00	0.000	

^{1}H NMR, ^{13}C NMR, ^{19}F NMR, HRMS, HPLC spectra of A16

^{1}H NMR

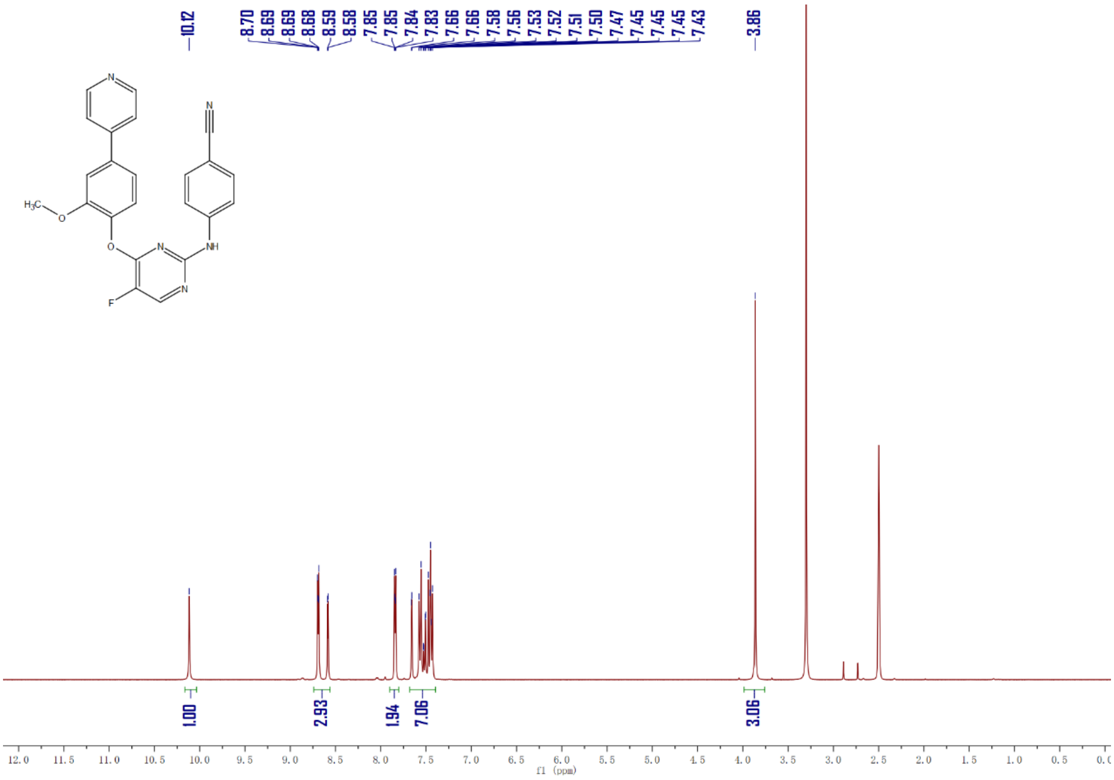

^{13}C NMR

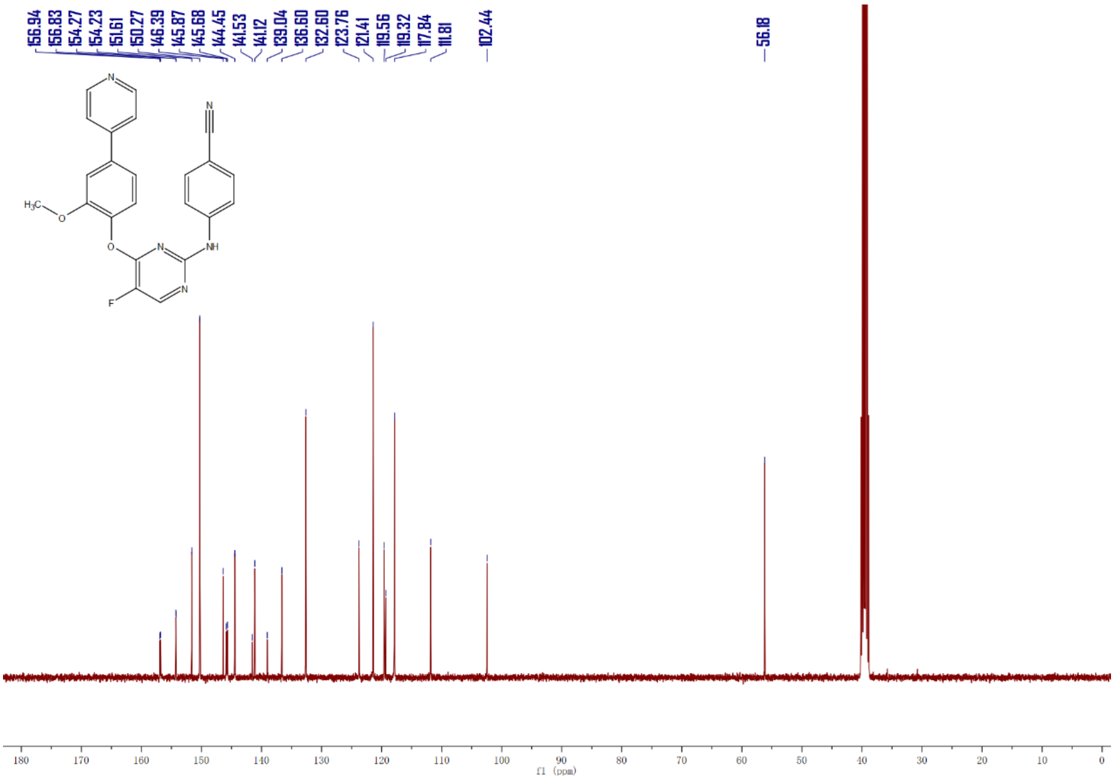

^{19}F NMR

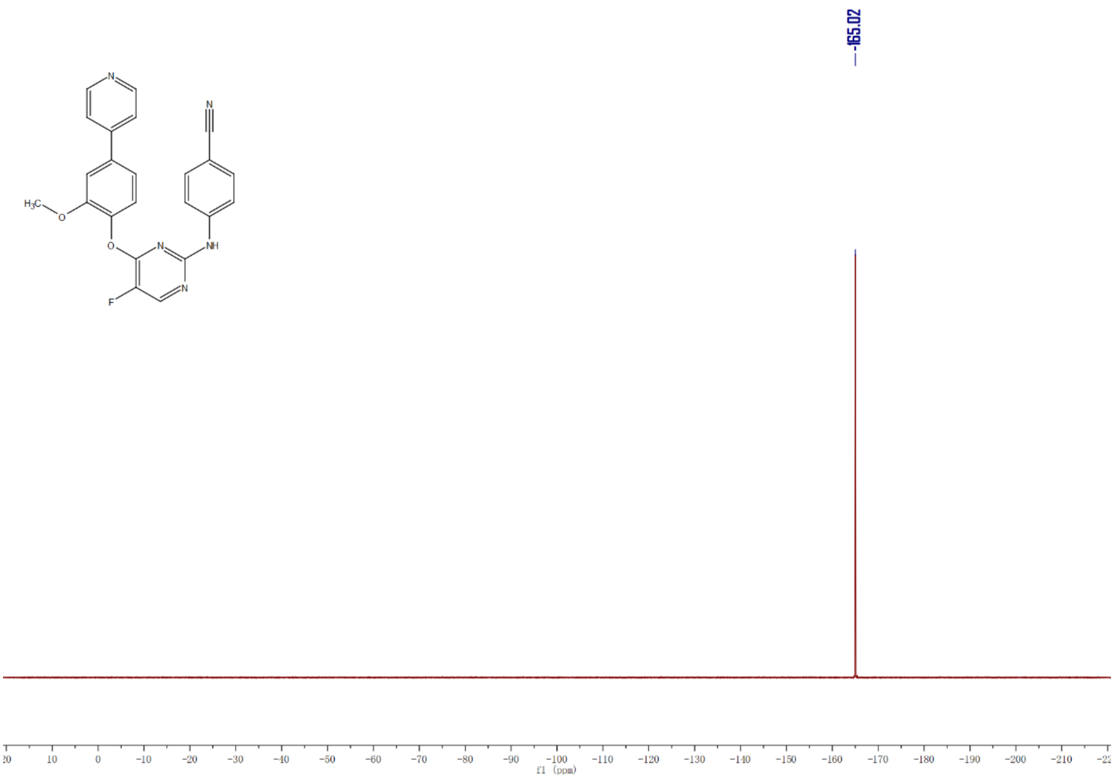

HRMS

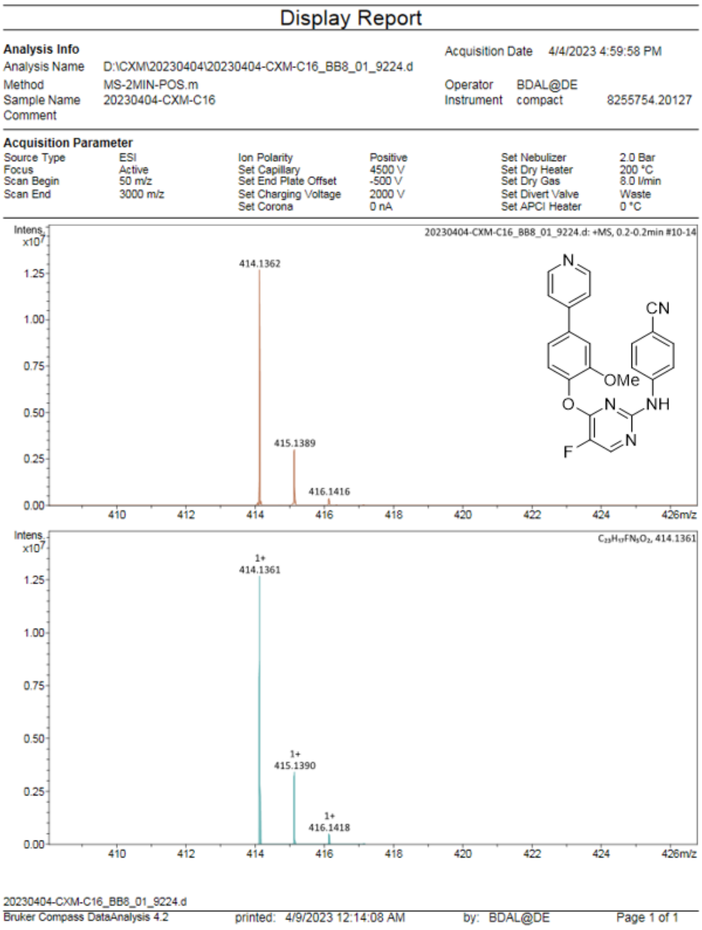

HPLC

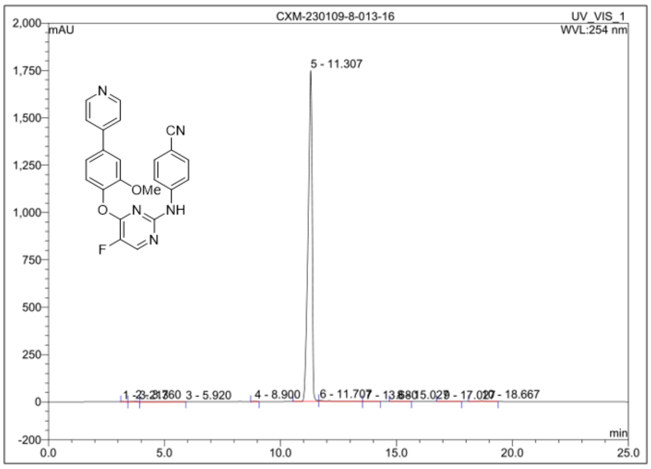

No.	Ret.Time min	Peak Name	Height mAU	Area mAU*s	Rel.Area %	Amount	Type
1	3.21	n.a.	0.752	3.92	0.02	n.a.	BMb
2	3.76	n.a.	2.979	37.18	0.18	n.a.	bMB
3	5.92	n.a.	0.000	33.81	0.16	n.a.	BMB
4	8.90	n.a.	3.373	25.38	0.12	n.a.	BMB
5	11.31	n.a.	1748.816	20780.19	97.96	n.a.	BM *
6	11.71	n.a.	5.916	226.44	1.07	n.a.	M *
7	13.68	n.a.	0.533	10.37	0.05	n.a.	MB*
8	15.03	n.a.	0.803	11.31	0.05	n.a.	BMB
9	17.03	n.a.	0.231	6.29	0.03	n.a.	BMB
10	18.67	n.a.	4.045	78.13	0.37	n.a.	BMB
Total:			1767.447	21213.019	100.00	0.000	

^{1}H NMR, ^{13}C NMR, ^{19}F NMR, HRMS, HPLC spectra of A17

^{1}H NMR

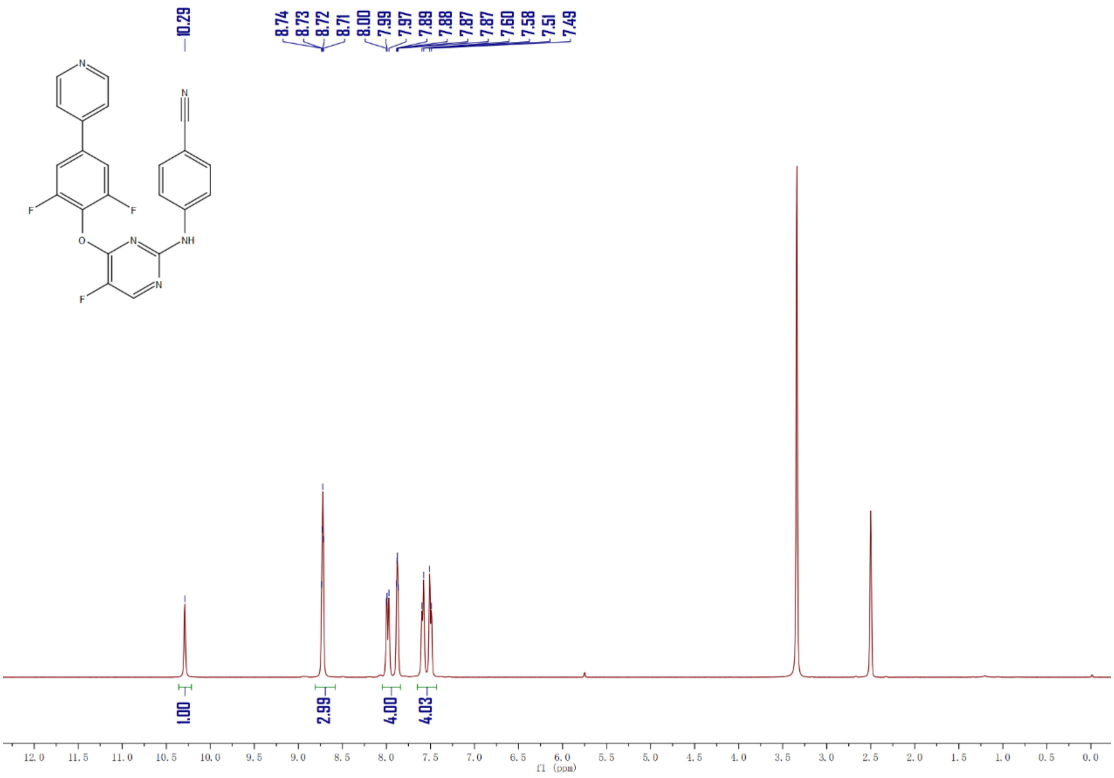

^{13}C NMR

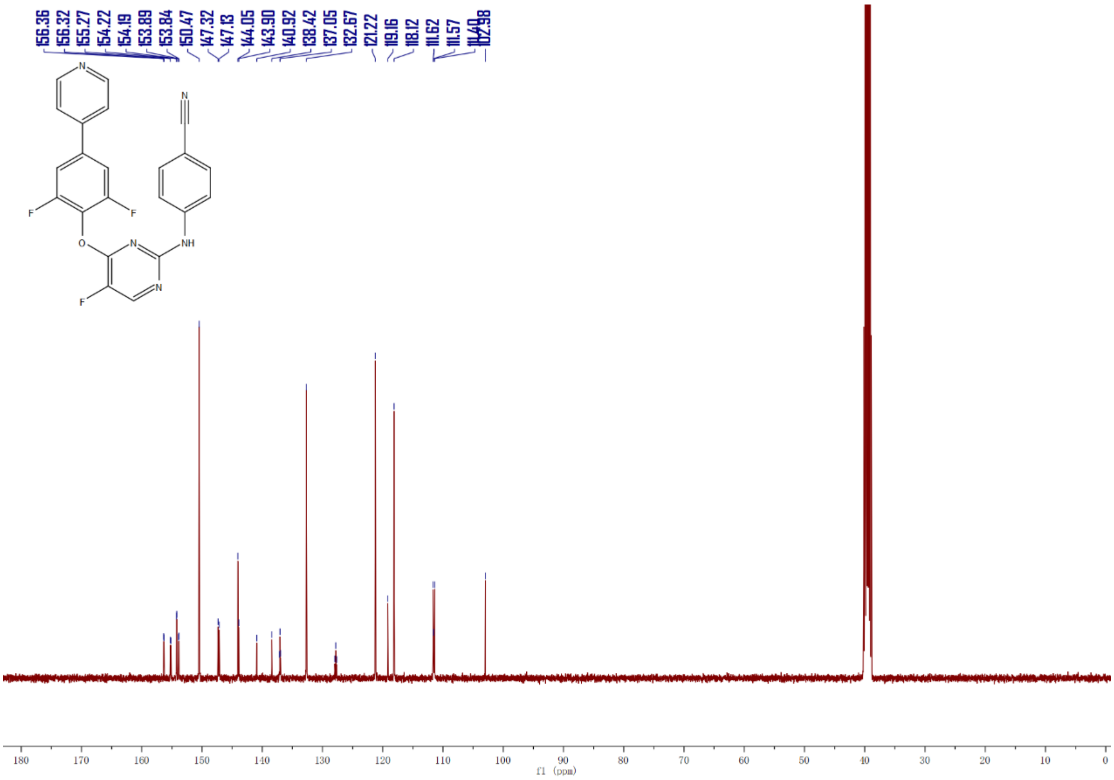

^{19}F NMR

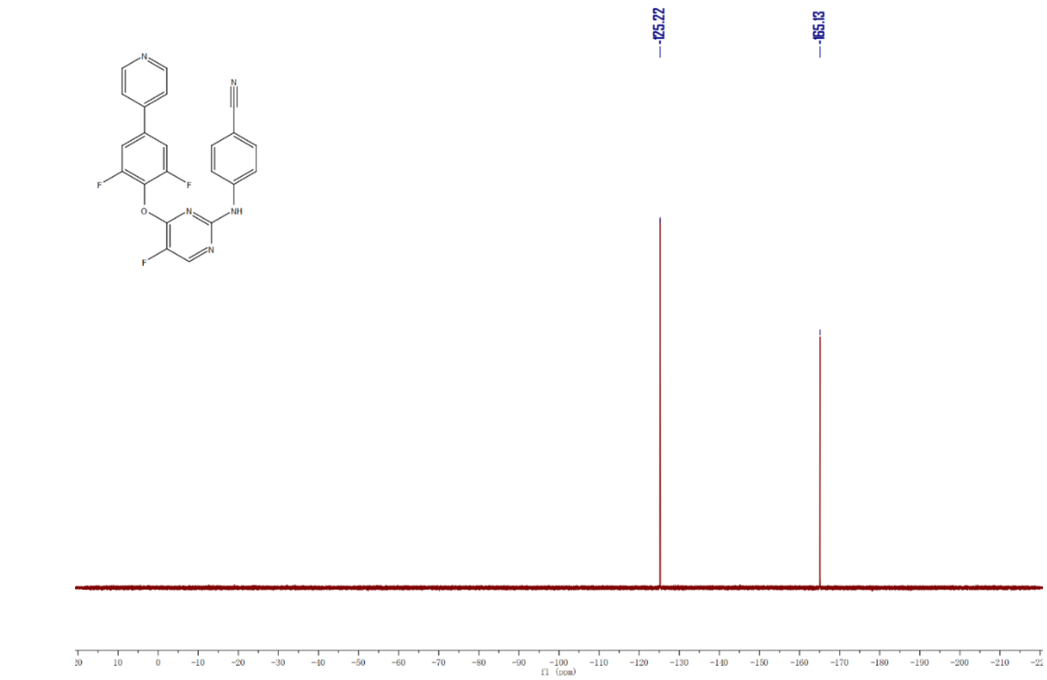

-125.22
-165.13
f1 (ppm)

HRMS

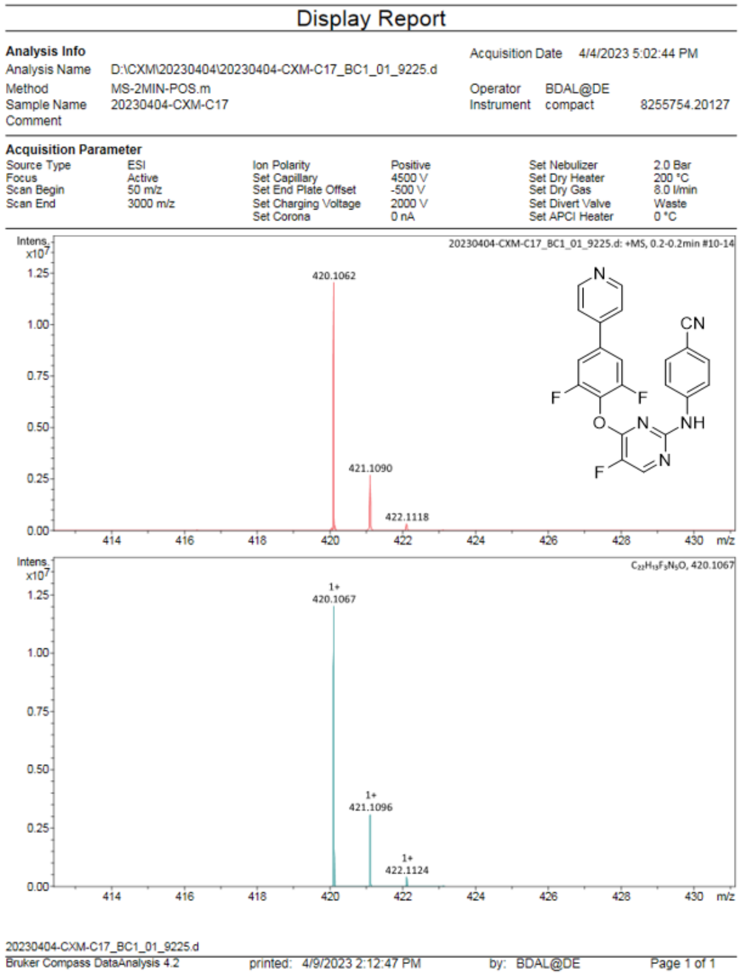

Display Report
Analysis Info
Analysis Name D:\CXM\20230404\20230404-CXM-C17_BC1_01_9225.d
Method MS-2MIN-POS.m
Sample Name 20230404-CXM-C17
Comment
Acquisition Date 4/4/2023 5:02:44 PM
Operator BDAL@DE
Instrument compact 8255754.20127
Acquisition Parameter
Source Type ESI
Focus Active
Scan Begin 50 m/z
Scan End 3000 m/z
Ion Polarity Positive
Set Capillary 4500 V
Set End Plate Offset -500 V
Set Charging Voltage 2000 V
Set Corona 0 nA
Set Nebulizer 2.0 Bar
Set Dry Heater 200 °C
Set Dry Gas 8.0 l/min
Set Divert Valve Waste
Set APCI Heater 0 °C
20230404-CXM-C17_BC1_01_9225.d: +MS, 0.2-0.2min #10-14
420.1062
421.1090
422.1118
420.1067
421.1096
422.1124
20230404-CXM-C17_BC1_01_9225.d
Bruker Compass DataAnalysis 4.2
printed: 4/9/2023 2:12:47 PM
by: BDAL@DE
Page 1 of 1

HPLC

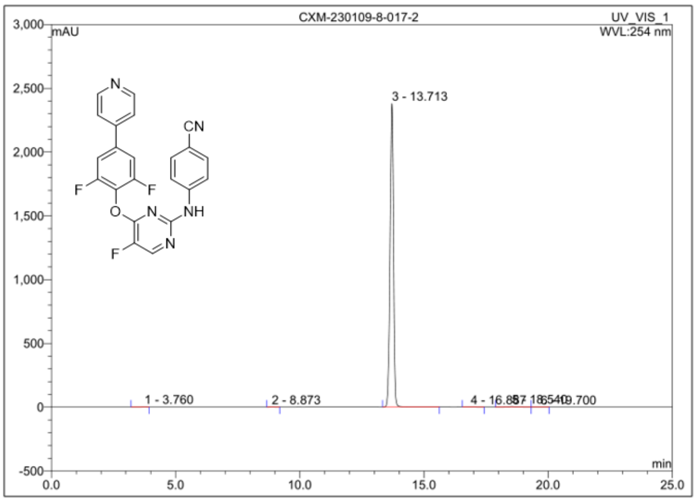

No.	Ret.Time min	Peak Name	Height mAU	Area mAU*s	Rel.Area %	Amount	Type
1	3.76	n.a.	3.041	30.51	0.13	n.a.	BMB
2	8.87	n.a.	1.601	12.33	0.05	n.a.	BMB
3	13.71	n.a.	2379.390	22616.05	99.45	n.a.	BMB
4	16.89	n.a.	0.242	4.92	0.02	n.a.	BMB
5	18.54	n.a.	3.650	73.65	0.32	n.a.	BM
6	19.70	n.a.	0.184	4.08	0.02	n.a.	MB
Total:			2388.108	22741.534	100.00	0.000	

^{1}H NMR, ^{13}C NMR, ^{19}F NMR, HRMS, HPLC spectra of A18

^{1}H NMR

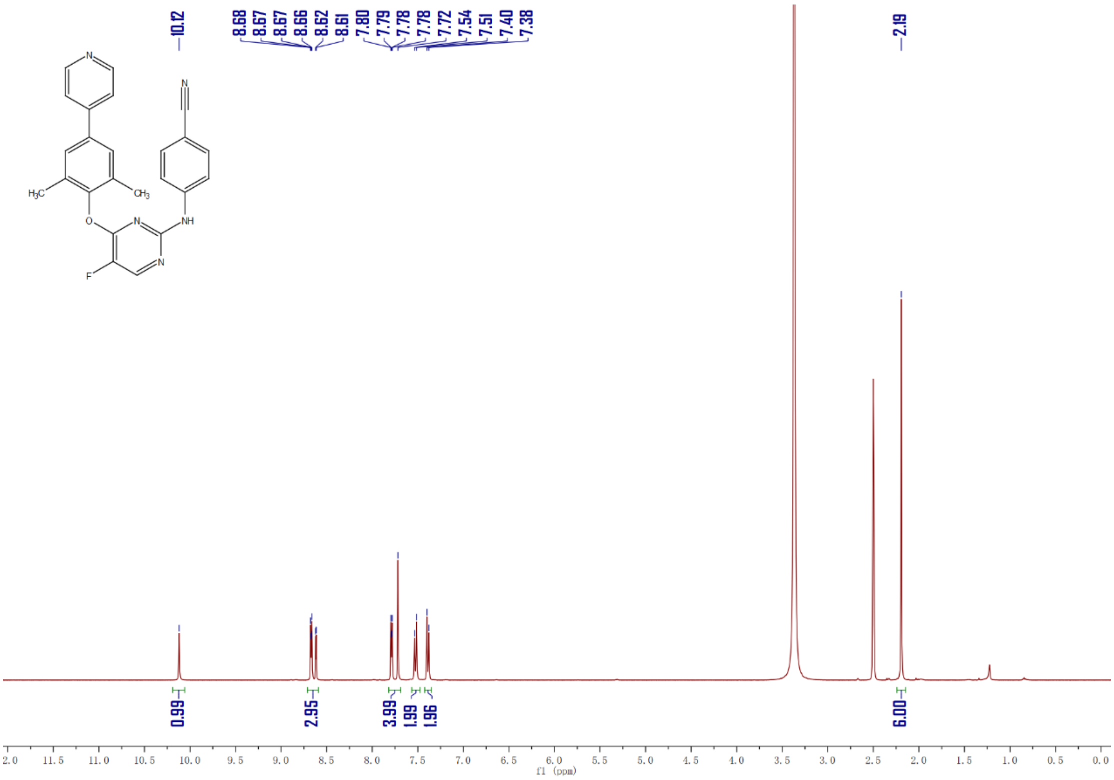

^{13}C NMR

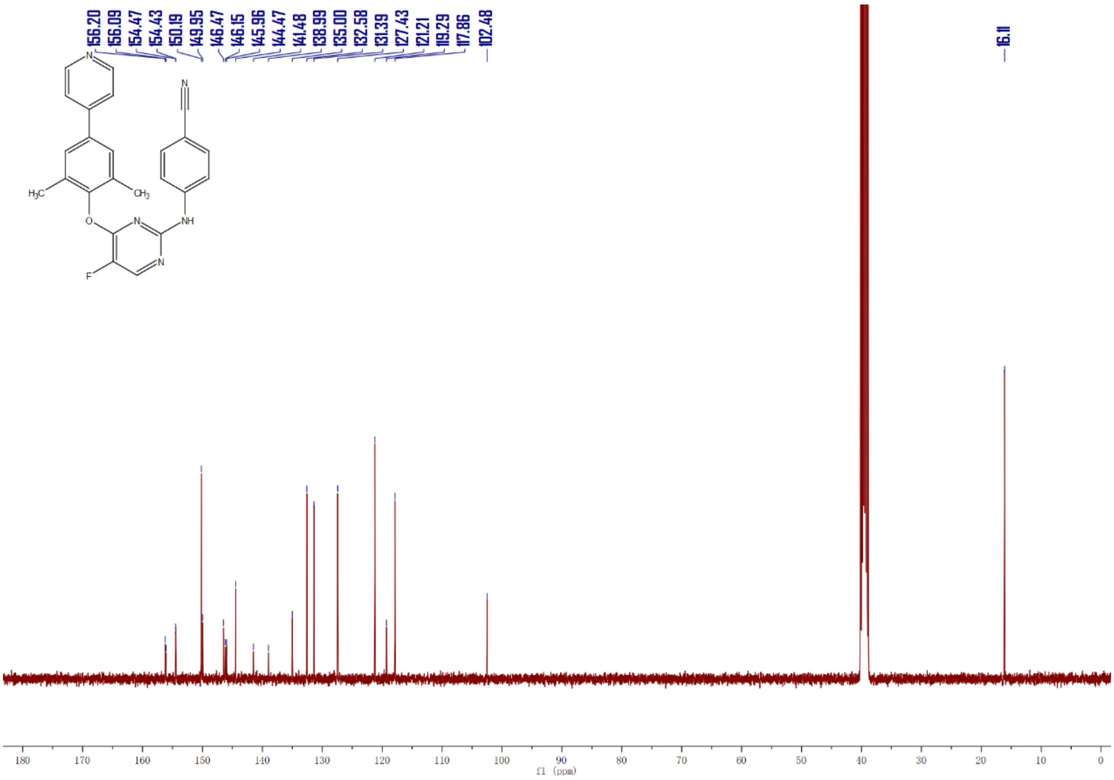

^{19}F NMR

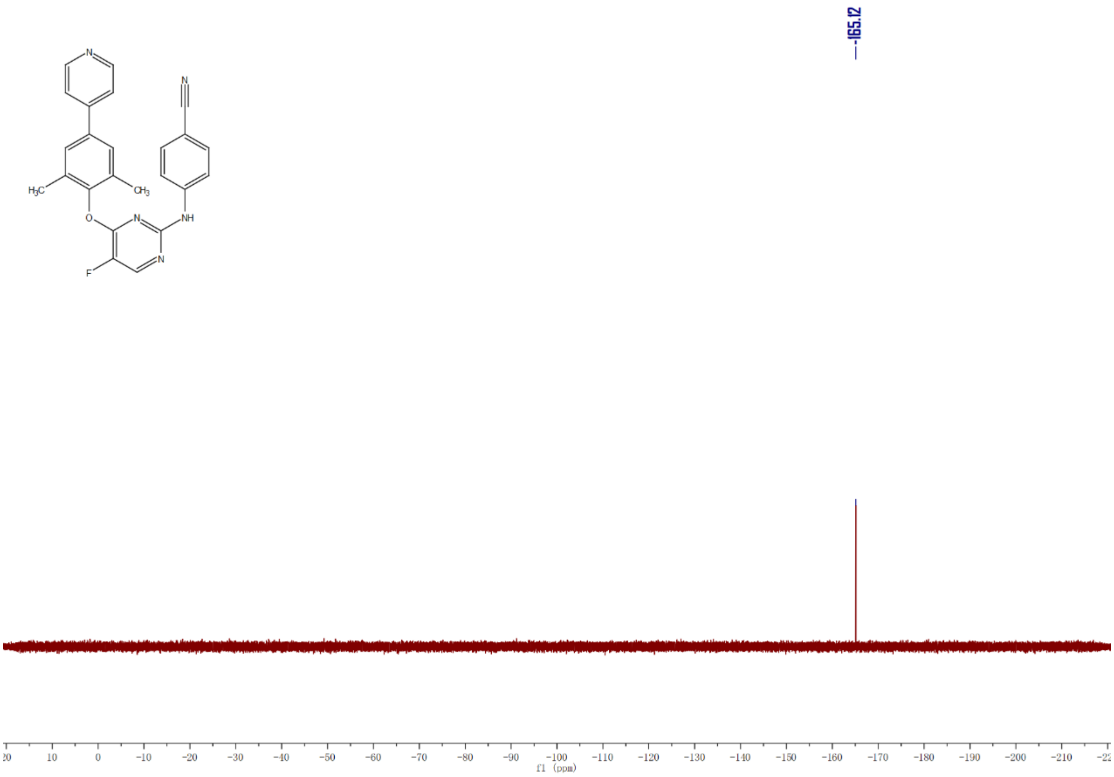

HRMS

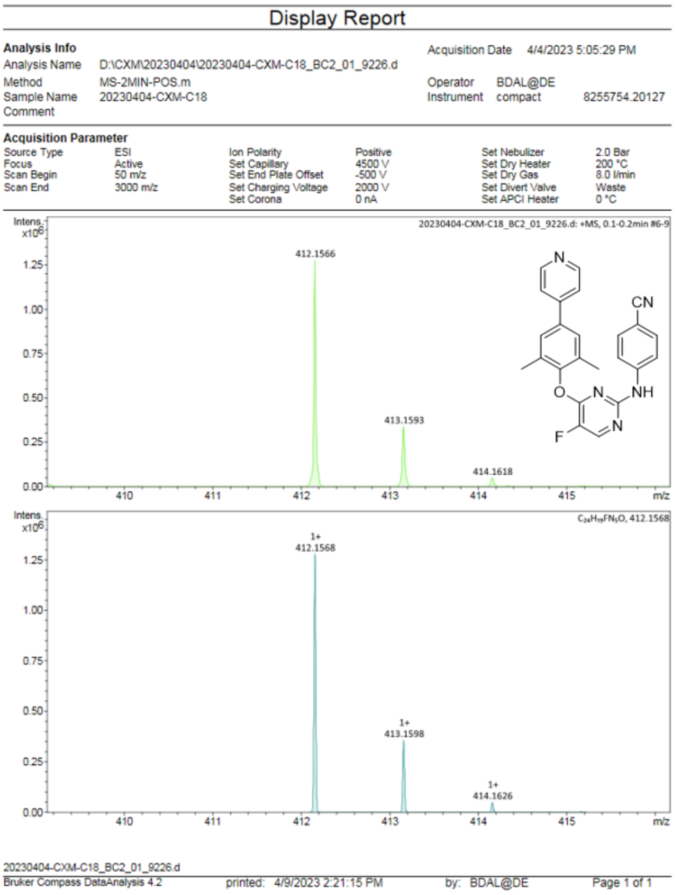

HPLC

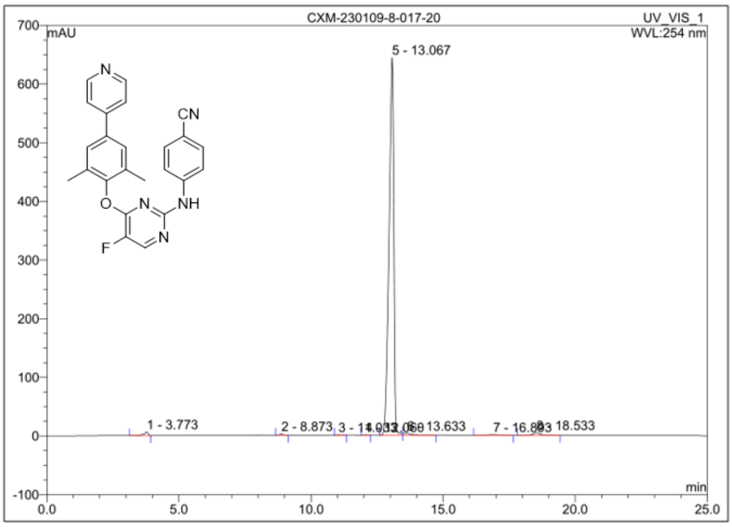

No.	Ret.Time min	Peak Name	Height mAU	Area mAU*s	Rel.Area %	Amount	Type
1	3.77	n.a.	6.465	73.72	0.88	n.a.	BMB
2	8.87	n.a.	2.556	20.58	0.24	n.a.	BMB
3	11.03	n.a.	0.414	4.70	0.06	n.a.	BMB
4	12.06	n.a.	0.392	3.08	0.04	n.a.	BMB
5	13.07	n.a.	643.601	8121.85	96.63	n.a.	BM
6	13.63	n.a.	2.942	64.34	0.77	n.a.	MB
7	16.89	n.a.	0.912	30.71	0.37	n.a.	BMB
8	18.53	n.a.	4.446	86.23	1.03	n.a.	BMB
Total:			661.728	8405.216	100.00	0.000	

^{1}H NMR, ^{13}C NMR, ^{19}F NMR, HRMS, HPLC spectra of A19

^{1}H NMR

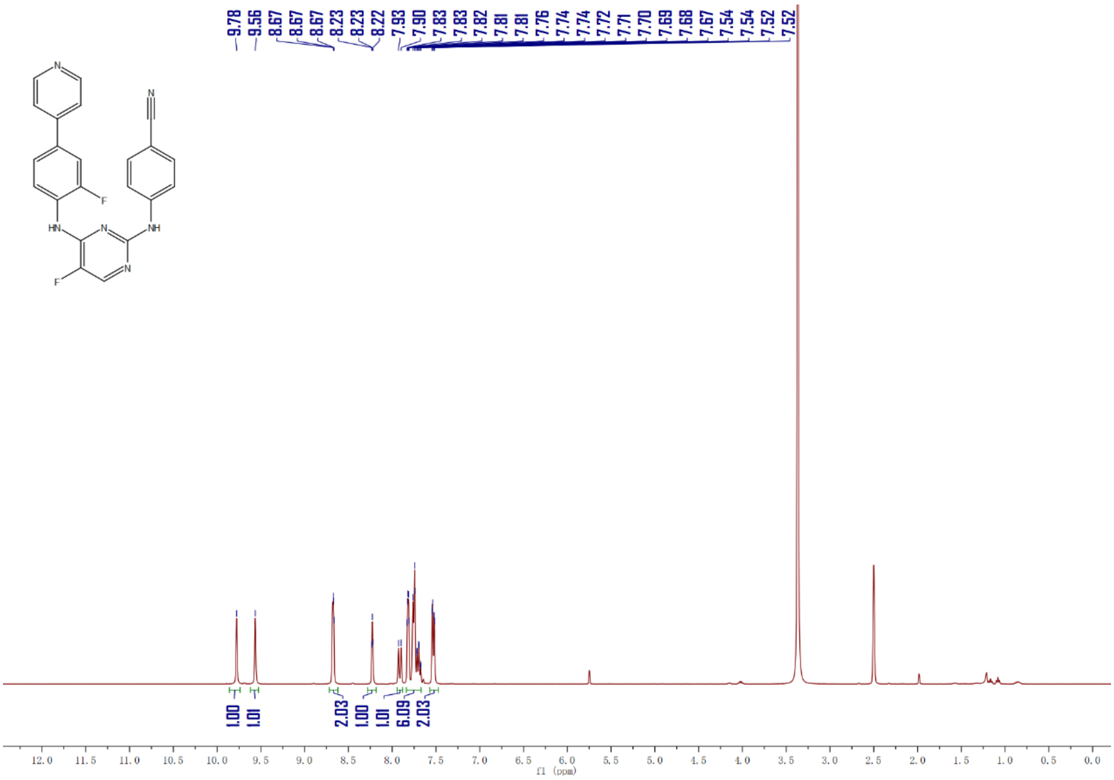

^{13}C NMR

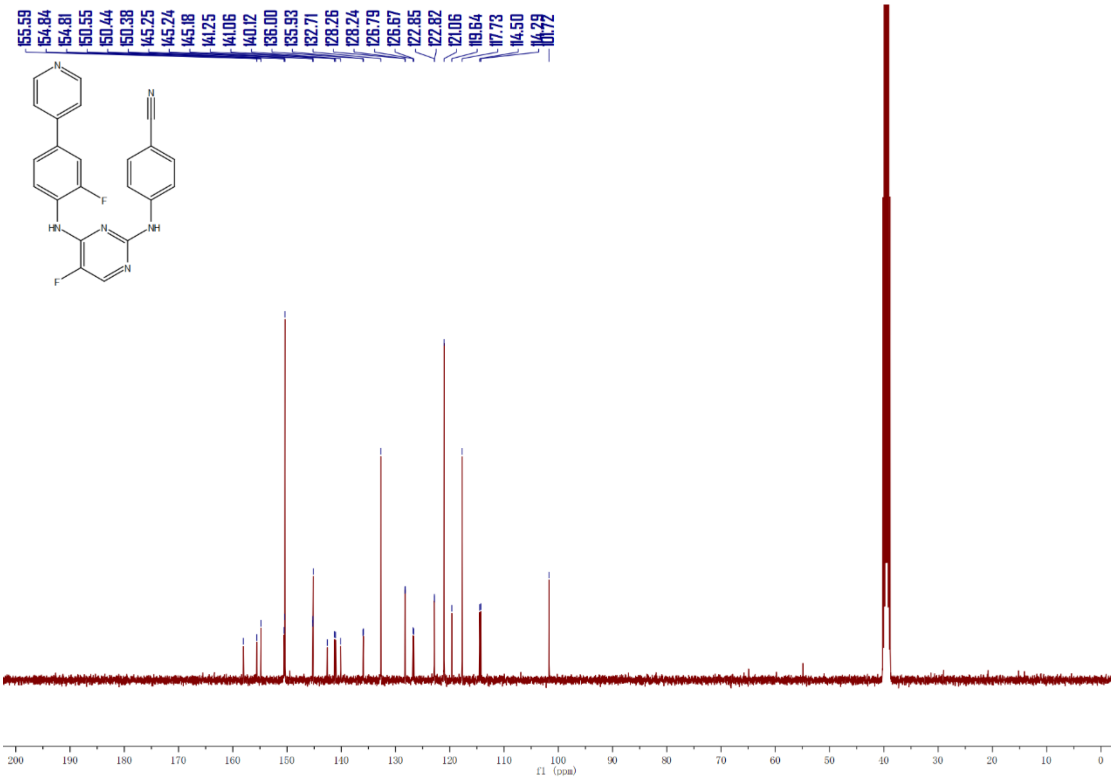

^{19}F NMR

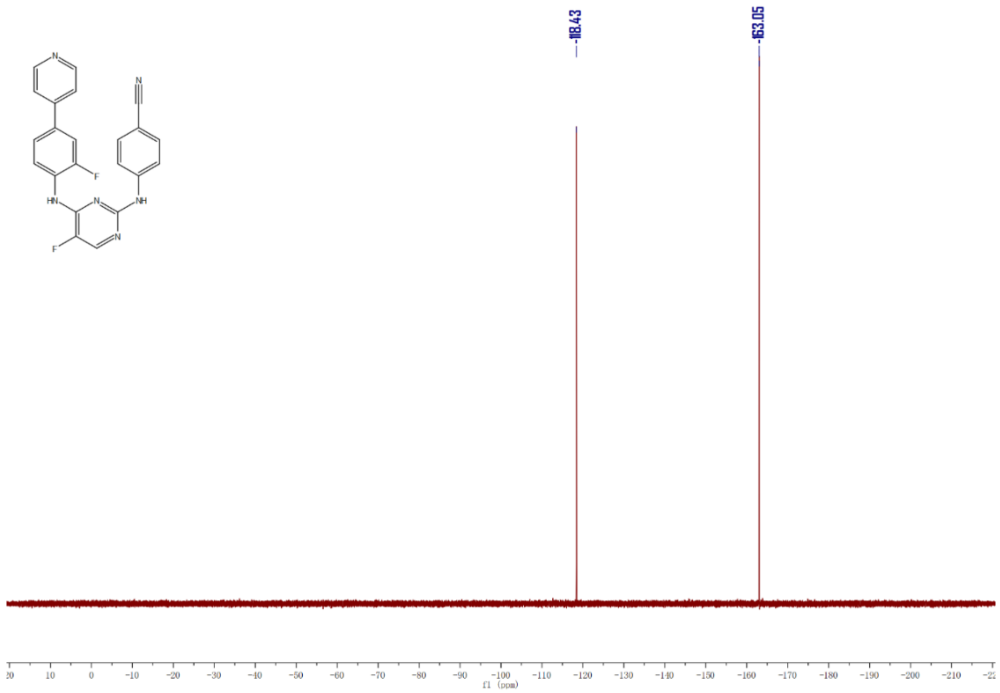

-118.43
-153.05
f1 (ppm)

HRMS

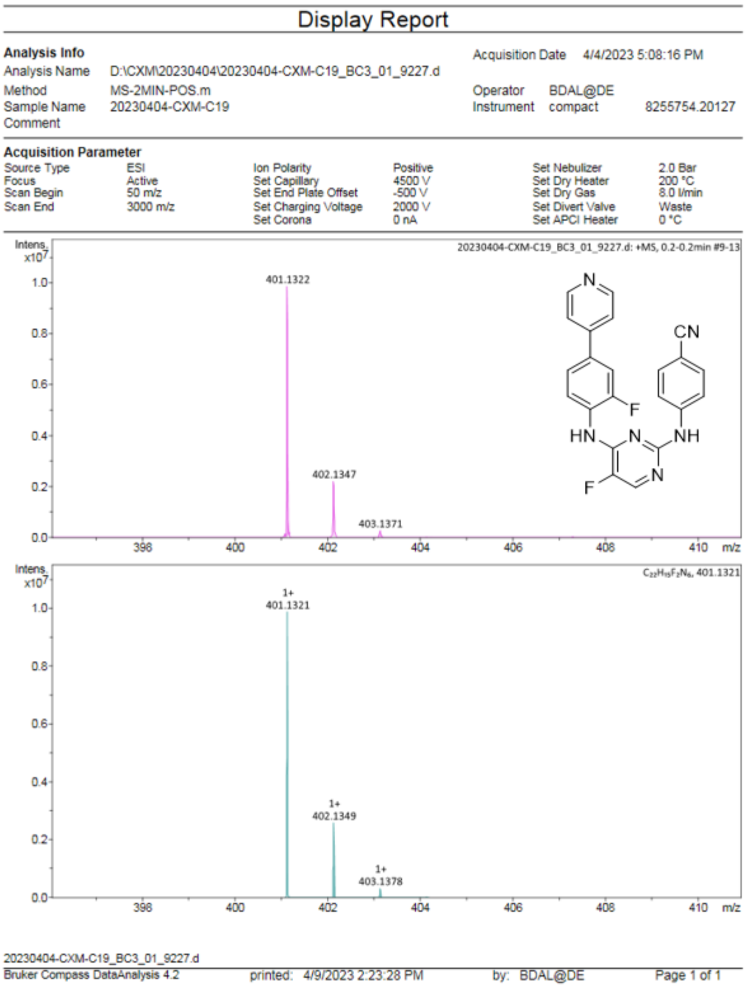

Display Report
Analysis Info
Analysis Name D:\CXM\20230404\20230404-CXM-C19_BC3_01_9227.d
Method MS-2MIN-POS.m
Sample Name 20230404-CXM-C19
Comment
Acquisition Date 4/4/2023 5:08:16 PM
Operator BDAL@DE
Instrument compact 8255754.20127
Acquisition Parameter
Source Type ESI
Focus Active
Scan Begin 50 m/z
Scan End 3000 m/z
Ion Polarity Positive
Set Capillary 4500 V
Set End Plate Offset -500 V
Set Charging Voltage 2000 V
Set Corona 0 nA
Set Nebulizer 2.0 Bar
Set Dry Heater 200 °C
Set Dry Gas 8.0 l/min
Set Divert Valve Waste
Set APCI Heater 0 °C
20230404-CXM-C19_BC3_01_9227.d: +MS, 0.2-0.2min #9-13
401.1322
402.1347
403.1371
C22H15F2N6, 401.1321
401.1321
402.1349
403.1378
20230404-CXM-C19_BC3_01_9227.d
Bruker Compass DataAnalysis 4.2
printed: 4/9/2023 2:23:28 PM
by: BDAL@DE
Page 1 of 1

HPLC

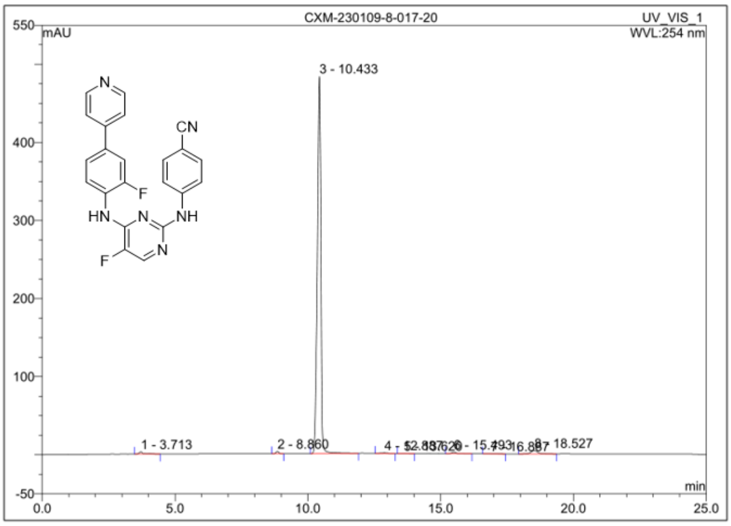

No.	Ret.Time min	Peak Name	Height mAU	Area mAU*s	Rel.Area %	Amount	Type
1	3.71	n.a.	2.886	43.40	0.93	n.a.	BMB*
2	8.86	n.a.	2.886	23.70	0.51	n.a.	BMB
3	10.43	n.a.	483.108	4466.14	95.59	n.a.	BMB
4	12.89	n.a.	0.670	13.63	0.29	n.a.	BMB
5	13.62	n.a.	0.280	4.31	0.09	n.a.	BMB
6	15.49	n.a.	2.121	32.90	0.70	n.a.	BMB
7	16.89	n.a.	0.223	4.48	0.10	n.a.	BMB
8	18.53	n.a.	4.314	83.64	1.79	n.a.	BMB
Total:			496.487	4672.204	100.00	0.000	

^{1}H NMR, ^{13}C NMR, ^{19}F NMR, HRMS, HPLC spectra of A20

^{1}H NMR

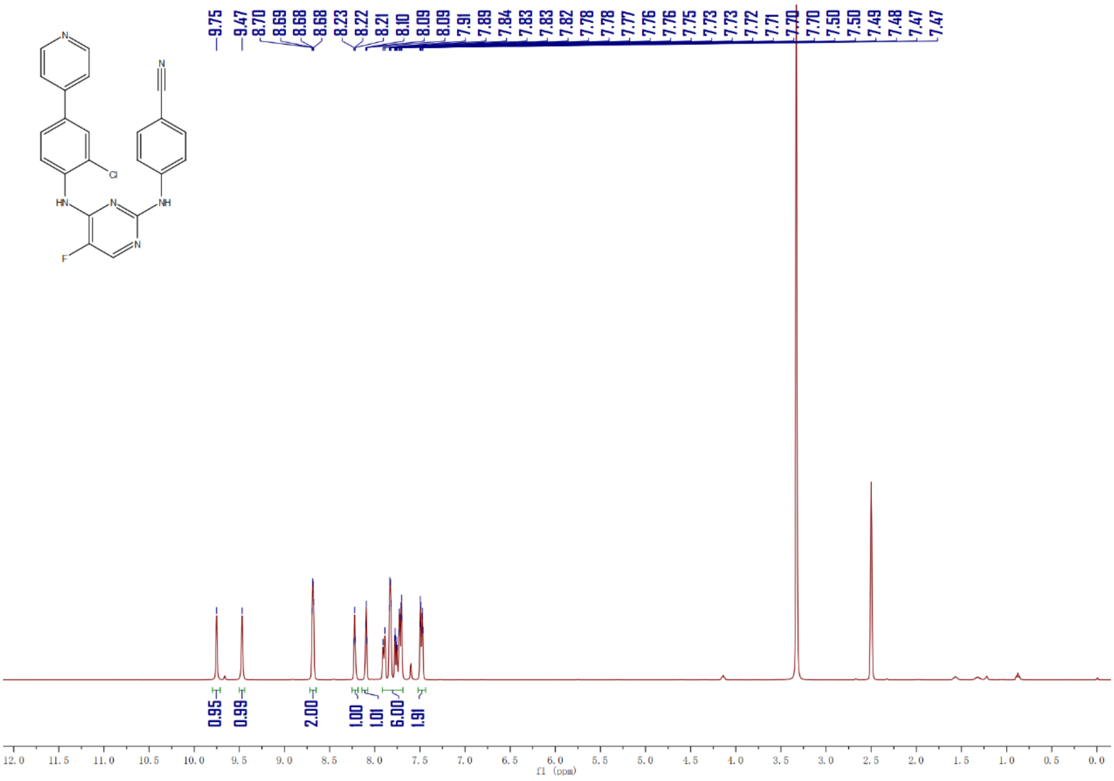

^{13}C NMR

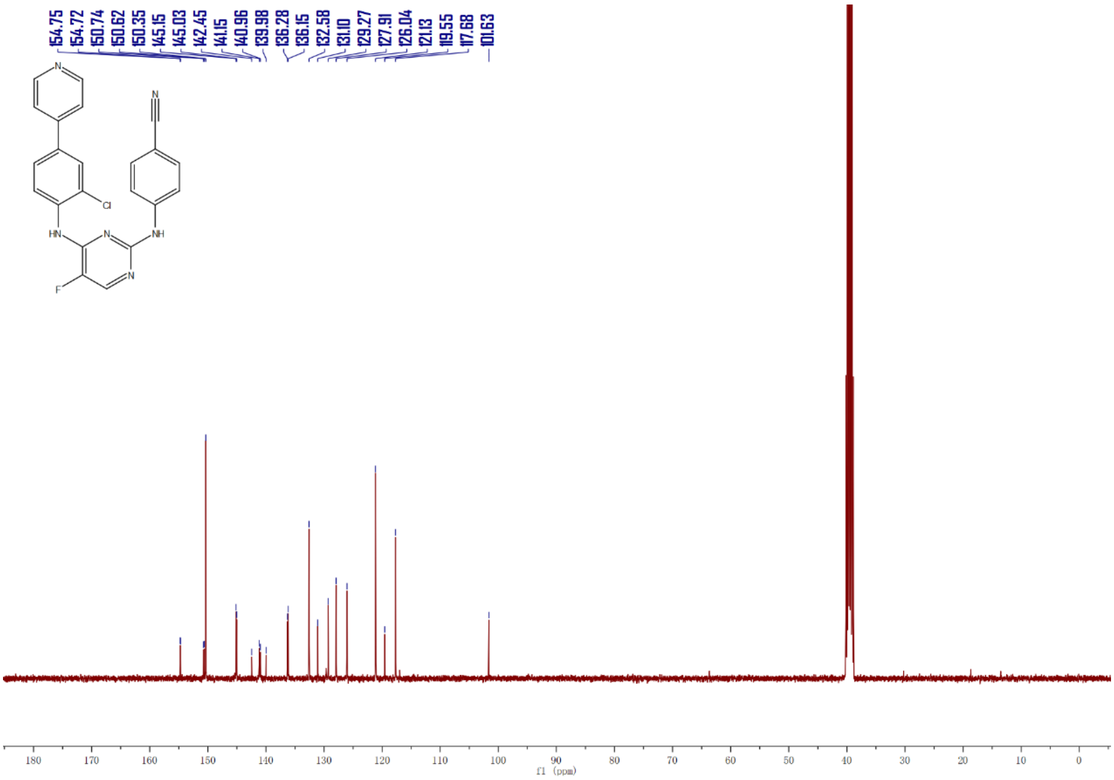

^{19}F NMR

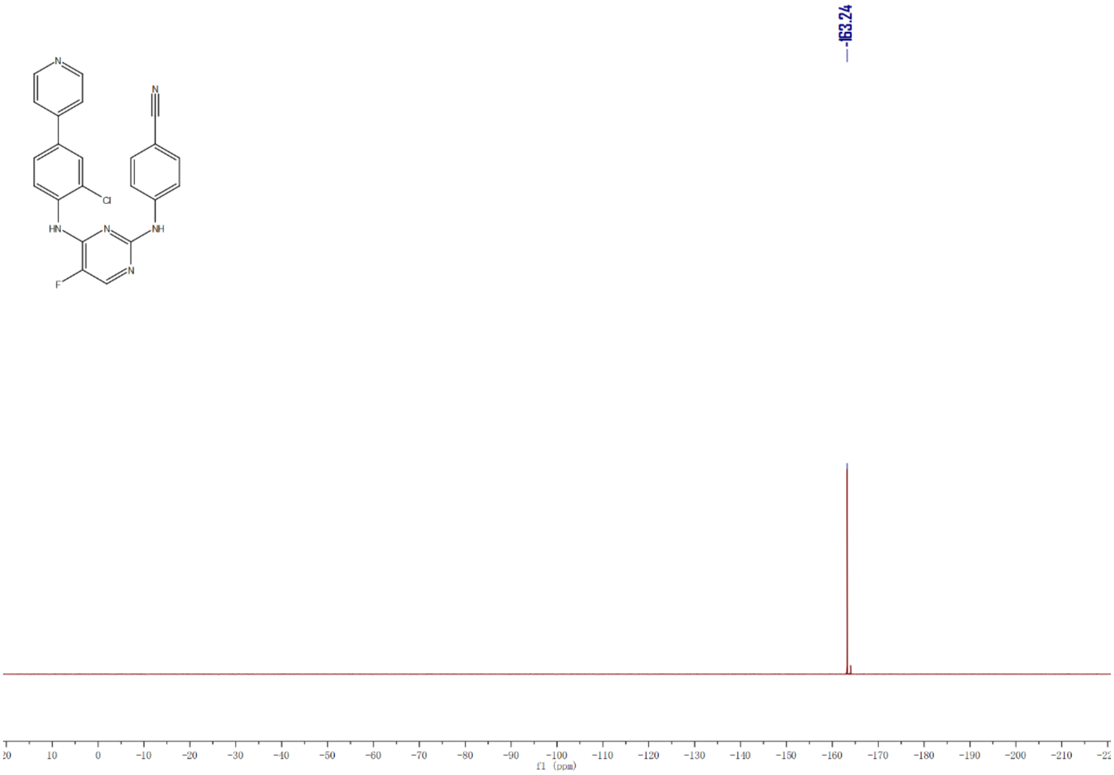

HRMS

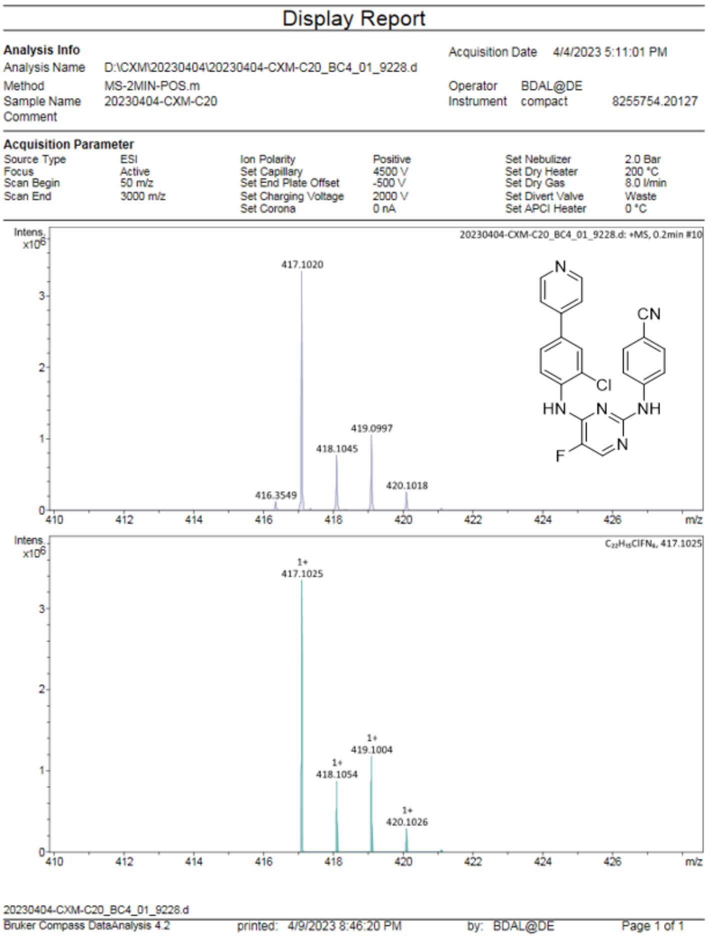

HPLC

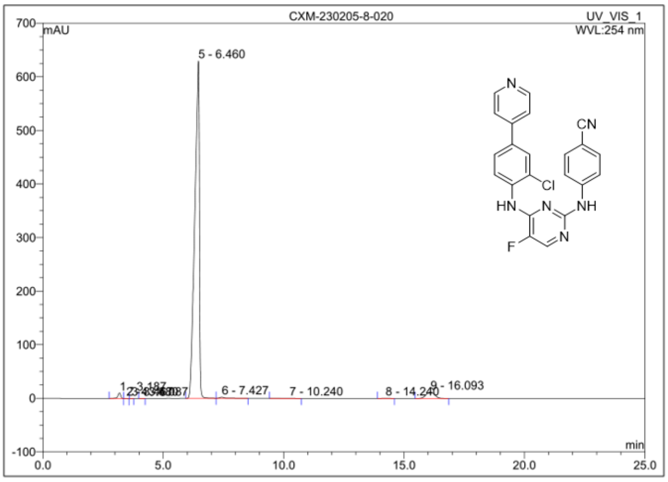

No.	Ret.Time min	Peak Name	Height mAU	Area mAU*s	Rel.Area %	Amount	Type
1	3.19	n.a.	10.077	86.91	1.00	n.a.	BMB
2	3.47	n.a.	0.554	4.37	0.05	n.a.	BMB
3	3.68	n.a.	0.279	1.65	0.02	n.a.	BMB*
4	4.09	n.a.	0.086	0.57	0.01	n.a.	BMB*
5	6.46	n.a.	629.744	8294.23	95.10	n.a.	BM *
6	7.43	n.a.	2.033	33.46	0.38	n.a.	MB
7	10.24	n.a.	0.133	2.59	0.03	n.a.	BMB*
8	14.24	n.a.	0.281	5.61	0.06	n.a.	BMB
9	16.09	n.a.	11.565	292.58	3.35	n.a.	BMB
Total:			654.752	8721.977	100.00	0.000	

^{1}H NMR, ^{13}C NMR, ^{19}F NMR, HRMS, HPLC spectra of A21

^{1}H NMR

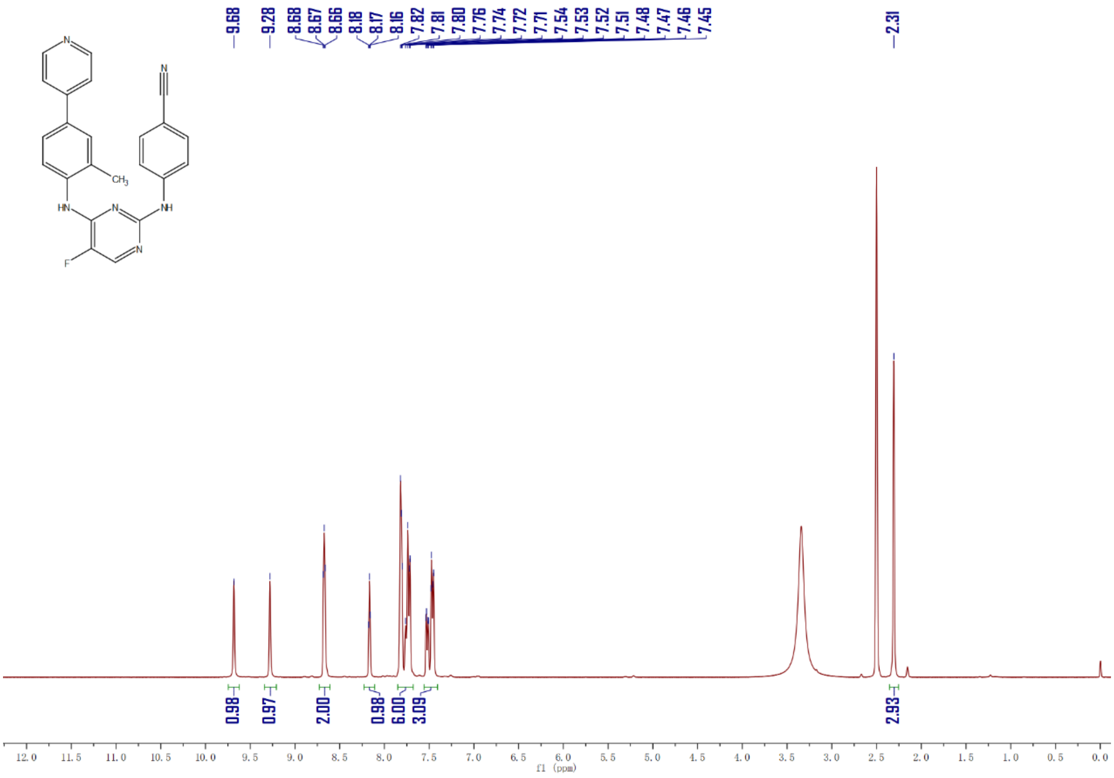

^{13}C NMR

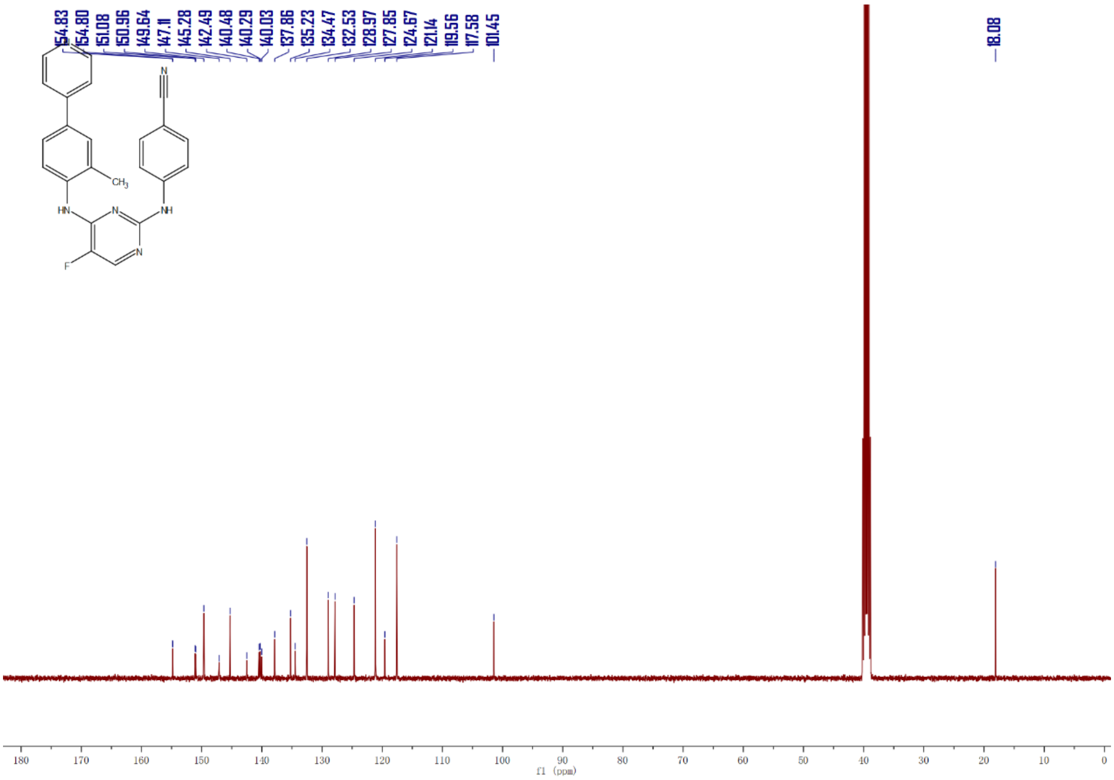

^{19}F NMR

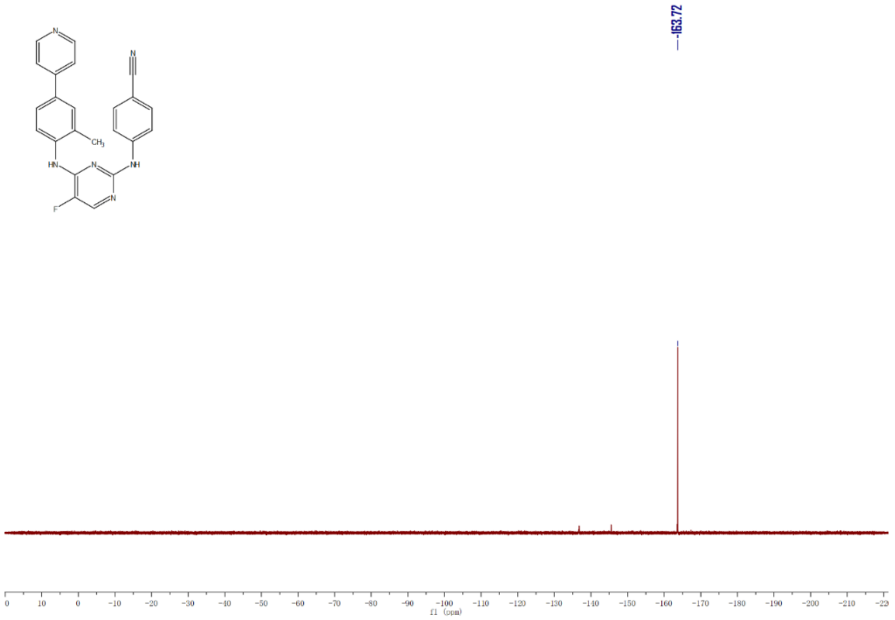

HRMS

Display Report

Analysis Info

Analysis Name	D:\CXM\20230404\20230404-CXM-C21_BC5_01_9229.d	Acquisition Date	4/4/2023 5:13:47 PM
Method	MS-2MIN-POS.m	Operator	BDAL@DE
Sample Name	20230404-CXM-C21	Instrument	compact 8255754.20127
Comment			

Acquisition Parameter

Source Type	ESI	Ion Polarity	Positive	Set Nebulizer	2.0 Bar
Focus	Active	Set Capillary	4500 V	Set Dry Heater	200 °C
Scan Begin	50 m/z	Set End Plate Offset	-500 V	Set Dry Gas	8.0 l/min
Scan End	3000 m/z	Set Charging Voltage	2000 V	Set Divert Valve	Waste
		Set Corona	0 nA	Set APCI Heater	0 °C

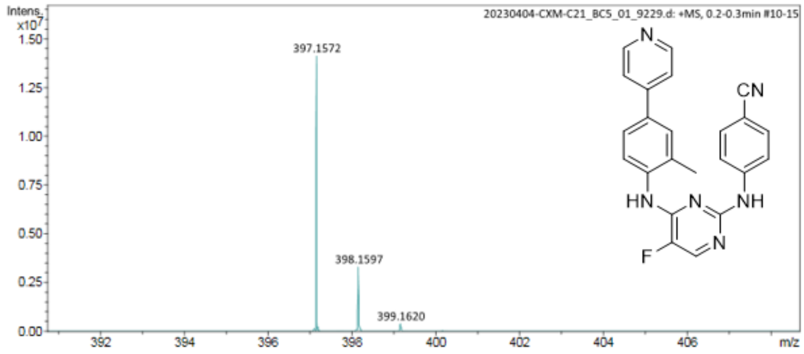

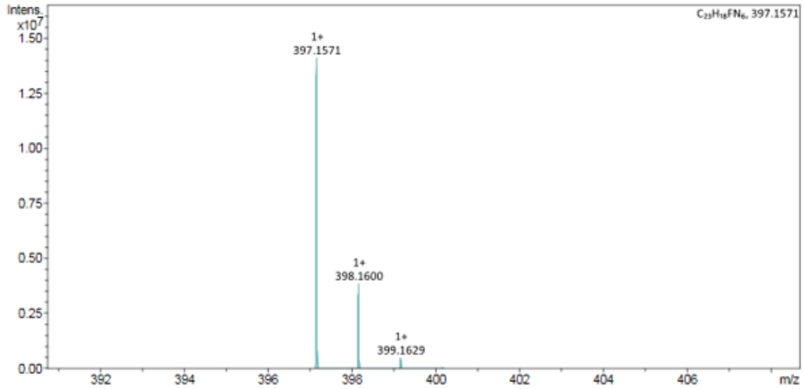

20230404-CXM-C21_BC5_01_9229.d

Bruker Compass DataAnalysis 4.2 printed: 4/9/2023 5:02:32 PM by: BDAL@DE Page 1 of 1

HPLC

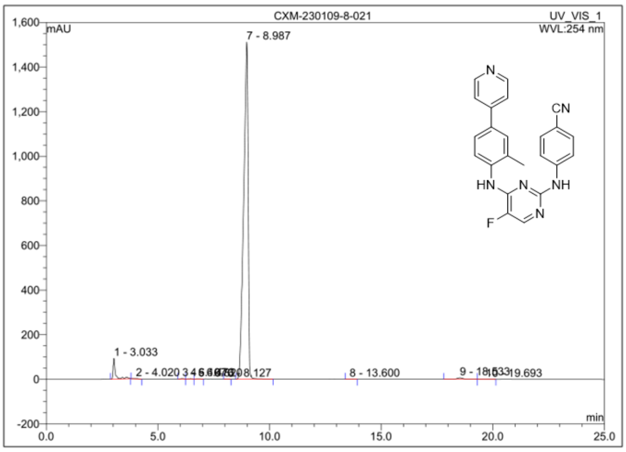

No.	Ret.Time min	Peak Name	Height mAU	Area mAU*s	Rel.Area %	Amount	Type
1	3.03	n.a.	93.490	667.02	3.07	n.a.	BMB*
2	4.02	n.a.	1.378	21.38	0.10	n.a.	BMB*
3	6.11	n.a.	1.776	22.19	0.10	n.a.	BM
4	6.47	n.a.	1.591	23.59	0.11	n.a.	M
5	6.82	n.a.	1.115	19.34	0.09	n.a.	MB*
6	8.13	n.a.	0.751	6.19	0.03	n.a.	BMB
7	8.99	n.a.	1511.079	20844.00	95.87	n.a.	BMB
8	13.60	n.a.	0.585	8.07	0.04	n.a.	BMB*
9	18.53	n.a.	6.218	120.73	0.56	n.a.	BM
10	19.69	n.a.	0.420	9.70	0.04	n.a.	MB
Total:			1618.401	21742.207	100.00	0.000	

^{1}H NMR, ^{13}C NMR, ^{19}F NMR, HRMS, HPLC spectra of A22

^{1}H NMR

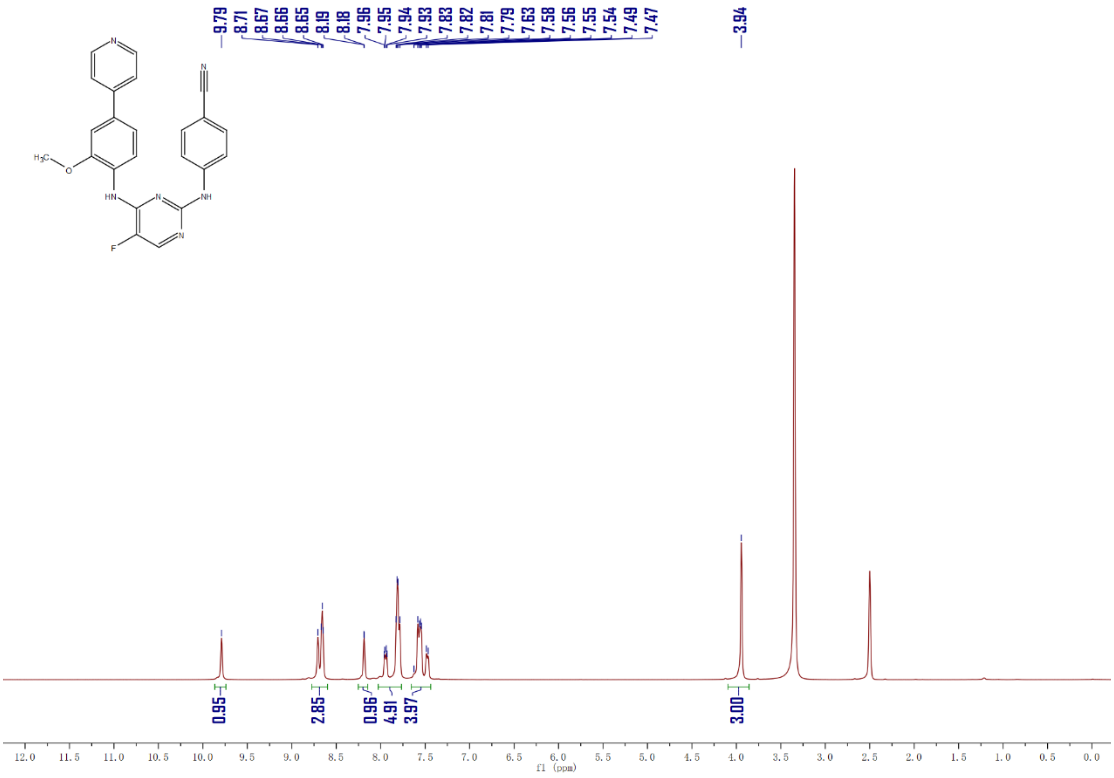

^{13}C NMR

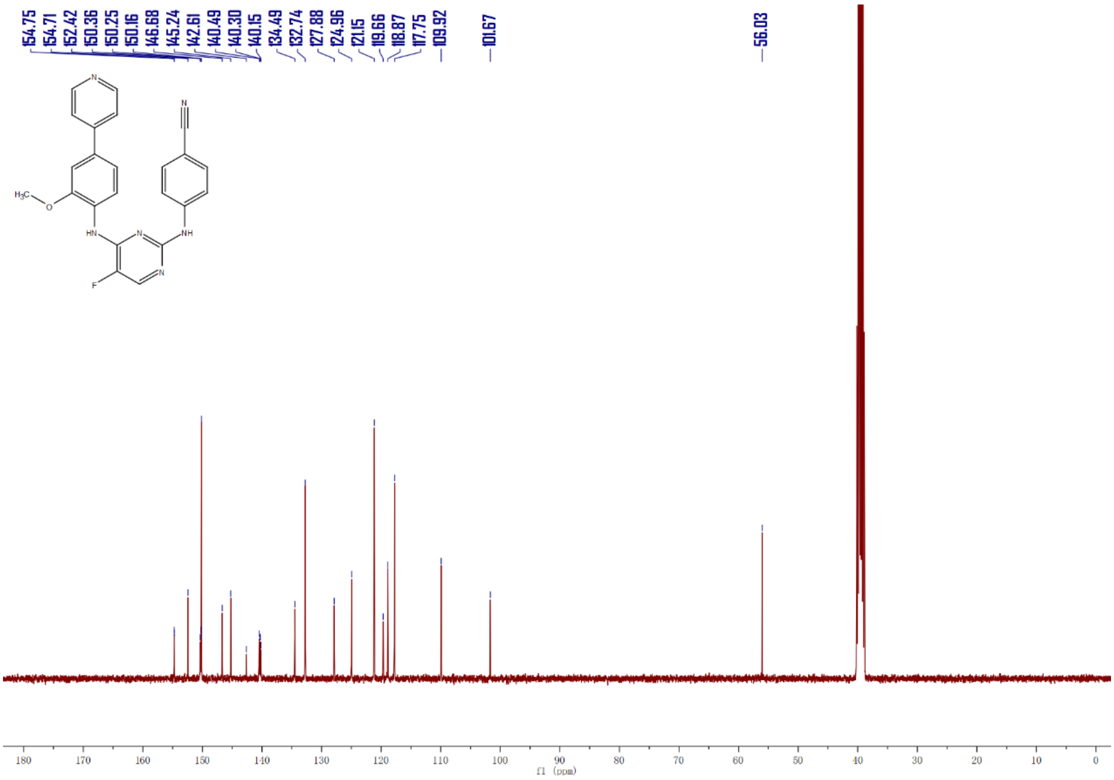

^{19}F NMR

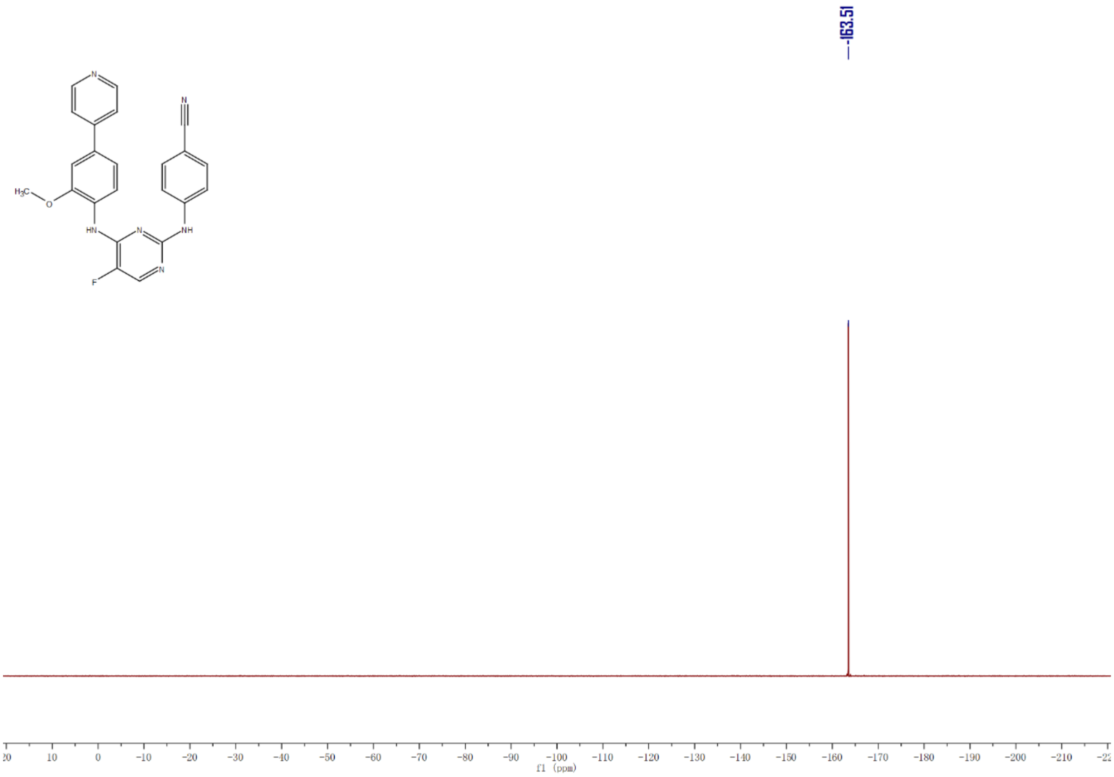

HRMS

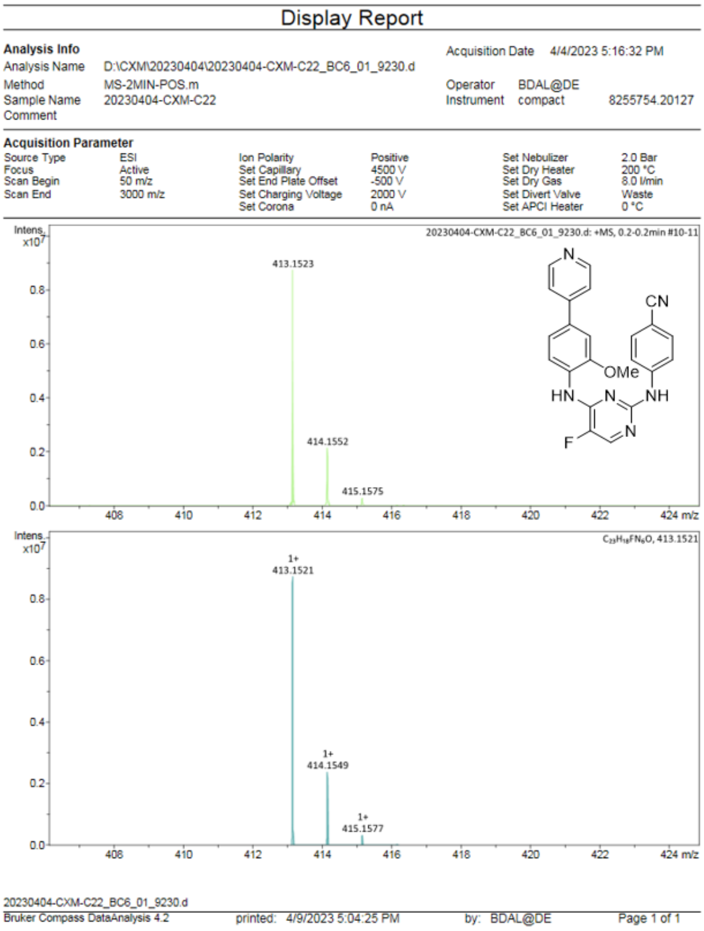

HPLC

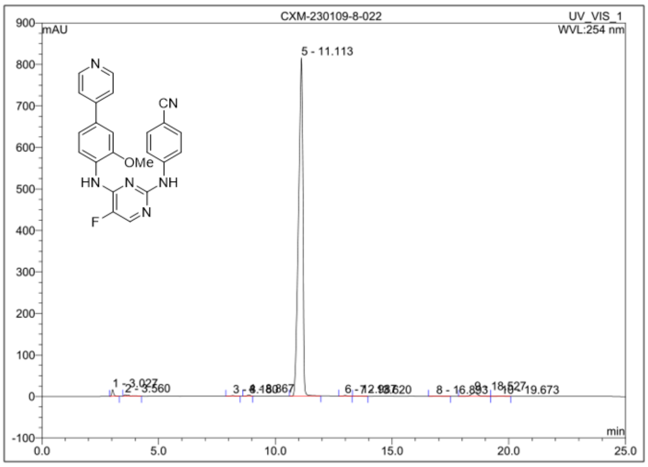

No.	Ret.Time min	Peak Name	Height mAU	Area mAU*s	Rel.Area %	Amount	Type
1	3.03	n.a.	16.166	90.48	0.83	n.a.	BMB
2	3.56	n.a.	3.216	54.80	0.50	n.a.	BMB*
3	8.18	n.a.	1.368	16.10	0.15	n.a.	BMB
4	8.87	n.a.	3.026	25.22	0.23	n.a.	BMB
5	11.11	n.a.	814.798	10485.29	96.28	n.a.	BMB
6	12.99	n.a.	1.394	14.53	0.13	n.a.	BMB
7	13.62	n.a.	0.475	7.19	0.07	n.a.	BMB
8	16.89	n.a.	0.447	8.87	0.08	n.a.	BMB
9	18.53	n.a.	9.429	175.77	1.61	n.a.	BM
10	19.67	n.a.	0.527	12.42	0.11	n.a.	MB
Total:			850.847	10890.653	100.00	0.000	

^{1}H NMR, ^{13}C NMR, ^{19}F NMR, HRMS, HPLC spectra of A23

^{1}H NMR

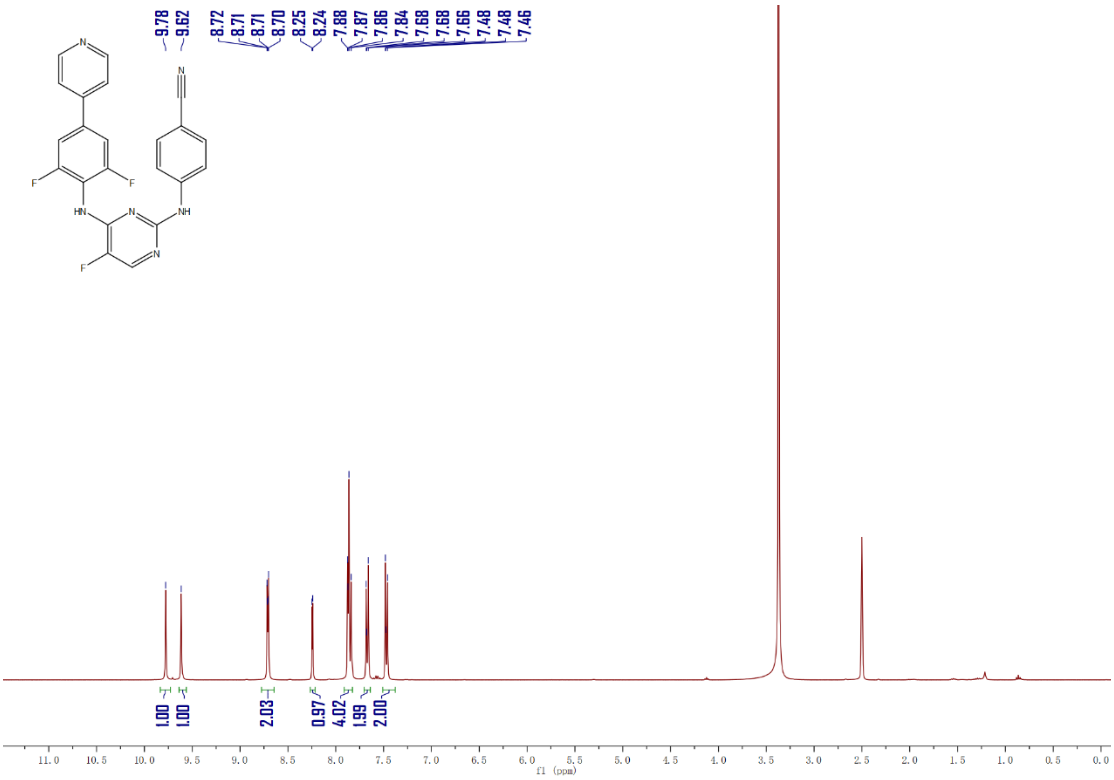

^{13}C NMR

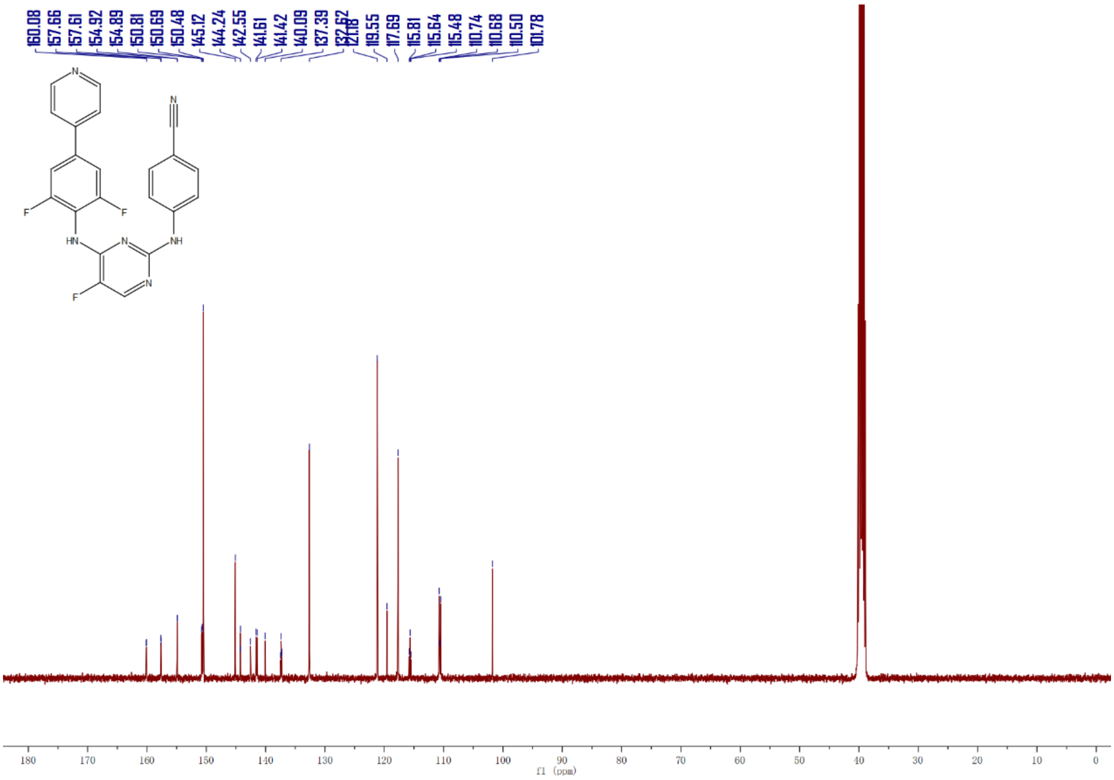

^{19}F NMR

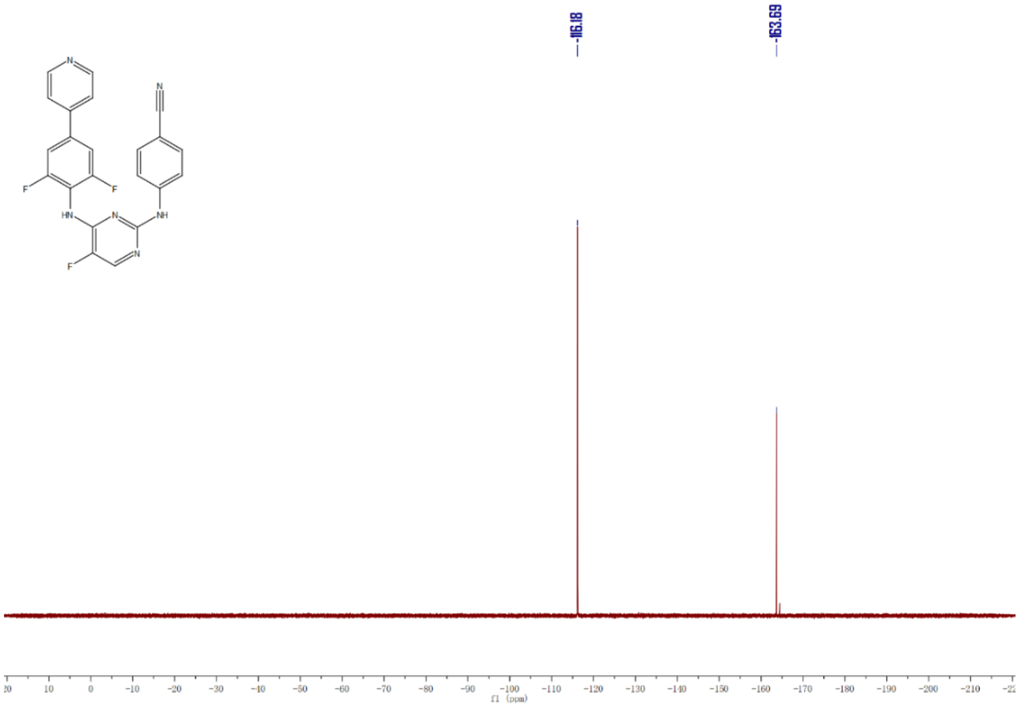

-116.18
-163.69
f1 (ppm)

HRMS

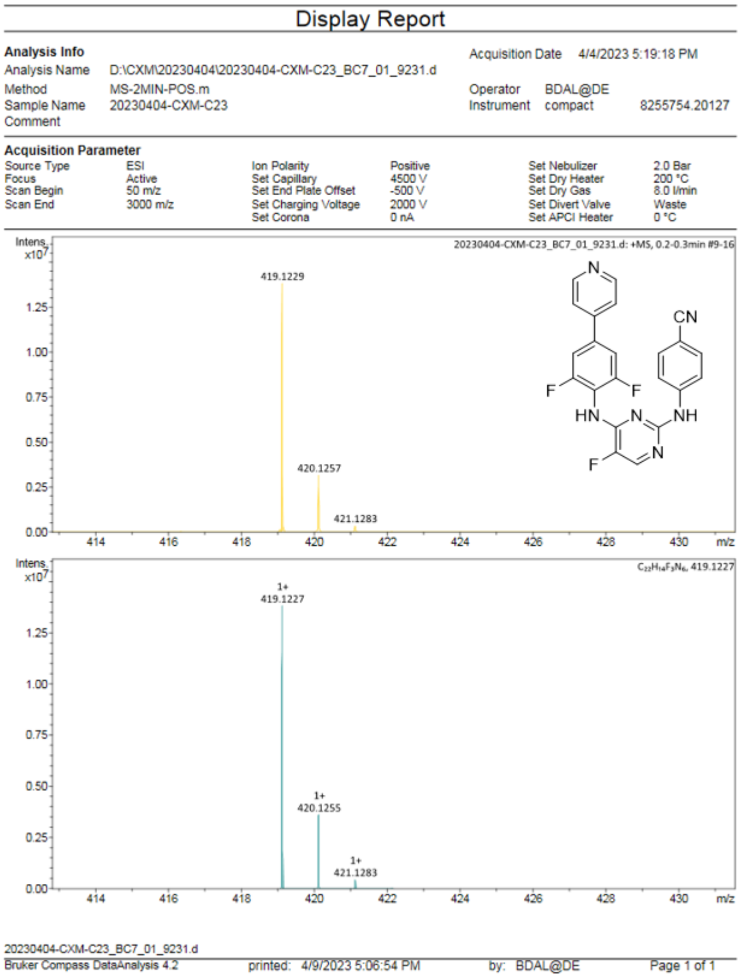

Display Report
Analysis Info
Analysis Name D:\CXM\20230404\20230404-CXM-C23_BC7_01_9231.d
Method MS-2MIN-POS.m
Sample Name 20230404-CXM-C23
Comment
Acquisition Date 4/4/2023 5:19:18 PM
Operator BDAL@DE
Instrument compact 8255754.20127
Acquisition Parameter
Source Type ESI
Focus Active
Scan Begin 50 m/z
Scan End 3000 m/z
Ion Polarity Positive
Set Capillary 4500 V
Set End Plate Offset -500 V
Set Charging Voltage 2000 V
Set Corona 0 nA
Set Nebulizer 2.0 Bar
Set Dry Heater 200 °C
Set Dry Gas 8.0 l/min
Set Divert Valve Waste
Set APCI Heater 0 °C
20230404-CXM-C23_BC7_01_9231.d: +MS, 0.2-0.3min #9-16
419.1229
420.1257
421.1283
419.1227
420.1255
421.1283
20230404-CXM-C23_BC7_01_9231.d
Bruker Compass DataAnalysis 4.2
printed: 4/9/2023 5:06:54 PM
by: BDAL@DE
Page 1 of 1

HPLC

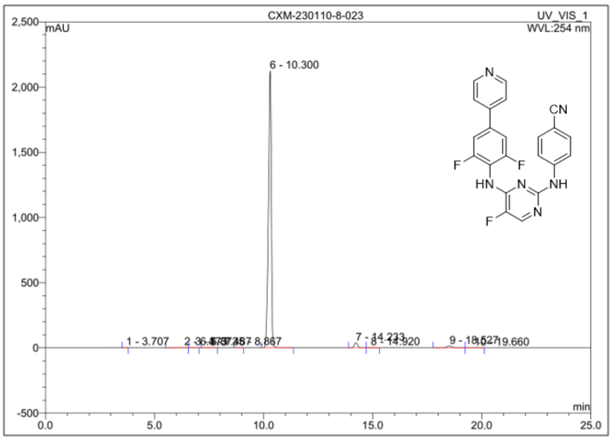

No.	Ret.Time min	Peak Name	Height mAU	Area mAU*s	Rel.Area %	Amount	Type
1	3.71	n.a.	2.137	17.05	0.08	n.a.	BMB
2	6.37	n.a.	2.250	22.39	0.11	n.a.	BMB*
3	6.87	n.a.	1.200	14.50	0.07	n.a.	BMB*
4	7.49	n.a.	0.983	16.34	0.08	n.a.	BMB*
5	8.87	n.a.	2.059	17.30	0.08	n.a.	BMB
6	10.30	n.a.	2122.940	19617.51	96.13	n.a.	BMB
7	14.23	n.a.	39.138	420.12	2.06	n.a.	BM
8	14.92	n.a.	1.363	18.71	0.09	n.a.	MB
9	18.53	n.a.	13.063	244.55	1.20	n.a.	BM
10	19.66	n.a.	0.861	19.63	0.10	n.a.	MB
Total:			2185.995	20408.085	100.00	0.000	

^{1}H NMR, ^{13}C NMR, ^{19}F NMR, HRMS, HPLC spectra of A24

^{1}H NMR

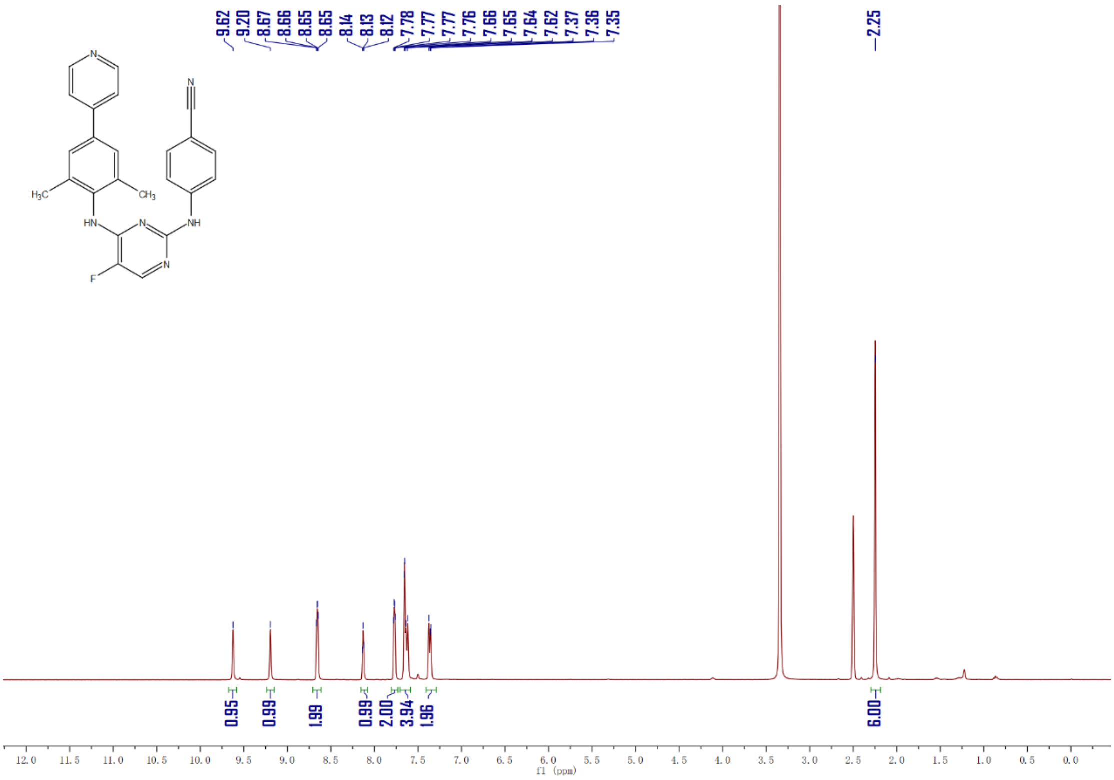

^{13}C NMR

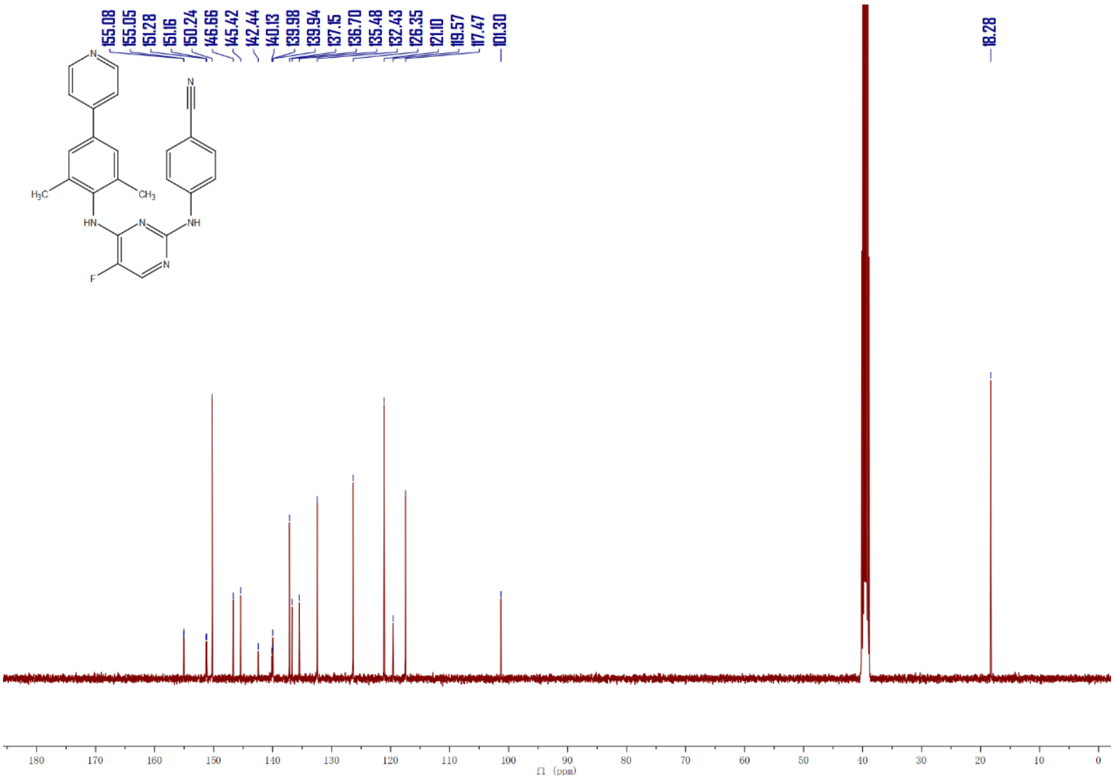

^{19}F NMR

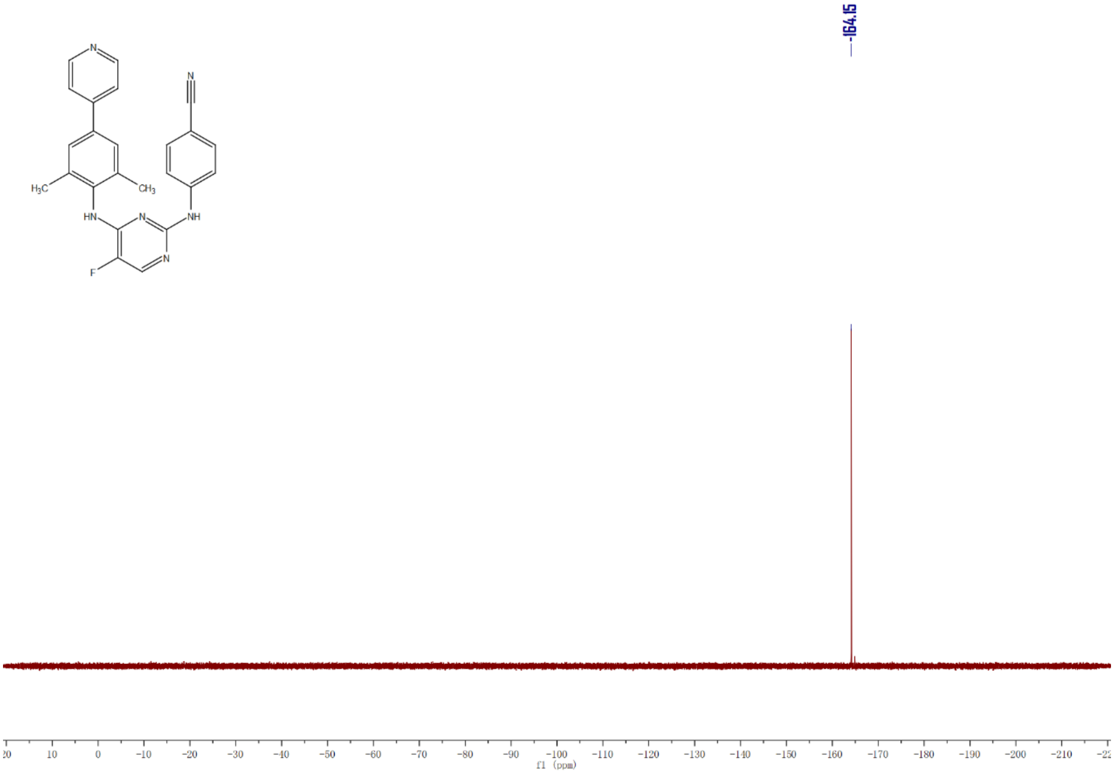

HRMS

Display Report

Analysis Info

Analysis Name	D:\CXM\20230404\20230404-CXM-C24_BC8_01_9232.d	Acquisition Date	4/4/2023 5:22:04 PM
Method	MS-2MIN-POS.m	Operator	BDAL@DE
Sample Name	20230404-CXM-C24	Instrument	compact 8255754.20127
Comment			

Acquisition Parameter

Source Type	ESI	Ion Polarity	Positive	Set Nebulizer	2.0 Bar
Focus	Active	Set Capillary	4500 V	Set Dry Heater	200 °C
Scan Begin	50 m/z	Set End Plate Offset	-500 V	Set Dry Gas	8.0 l/min
Scan End	3000 m/z	Set Charging Voltage	2000 V	Set Divert Valve	Waste
		Set Corona	0 nA	Set APCI Heater	0 °C

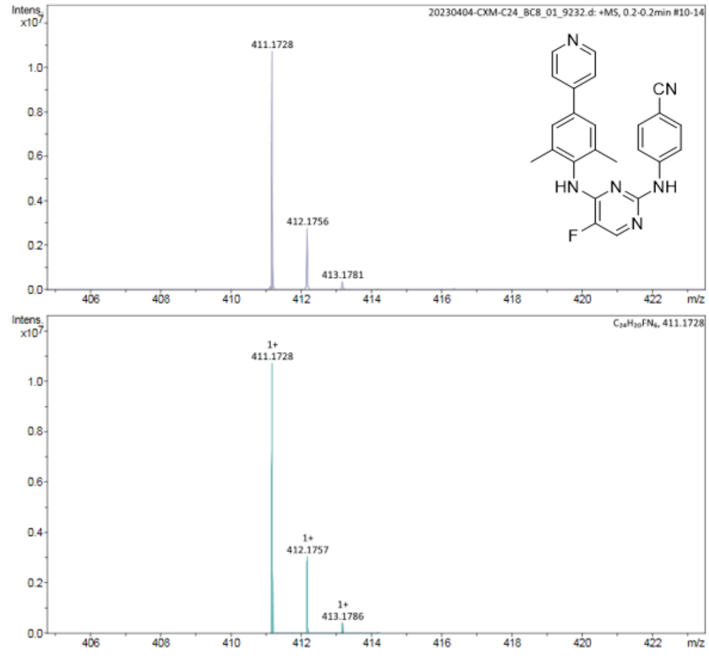

20230404-CXM-C24_BC8_01_9232.d

Bruker Compass DataAnalysis 4.2 printed: 4/9/2023 5:08:59 PM by: BDAL@DE Page 1 of 1

HPLC

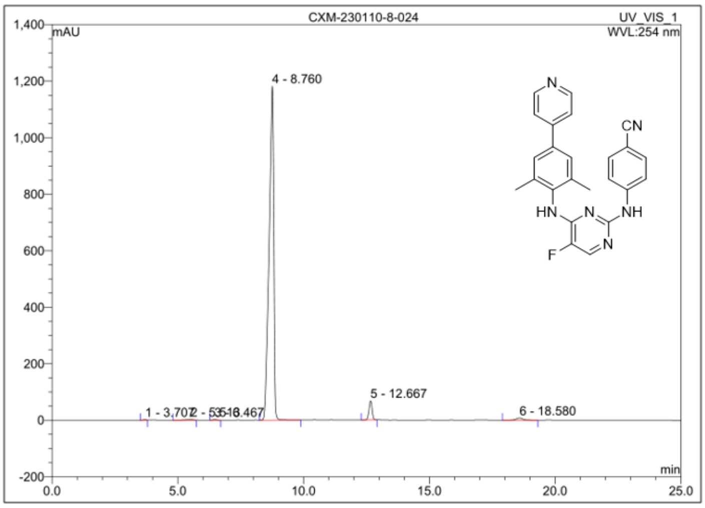

No.	Ret.Time min	Peak Name	Height mAU	Area mAU*s	Rel.Area %	Amount	Type
1	3.71	n.a.	1.763	13.34	0.08	n.a.	BMB
2	5.51	n.a.	2.297	38.07	0.24	n.a.	BMB
3	6.47	n.a.	1.993	16.45	0.10	n.a.	BMB
4	8.76	n.a.	1181.863	15207.74	95.02	n.a.	BMB
5	12.67	n.a.	68.023	586.94	3.67	n.a.	BMB*
6	18.58	n.a.	7.656	142.62	0.89	n.a.	BMB*
Total:			1263.594	16005.158	100.00	0.000	

^{1}H NMR, ^{13}C NMR, ^{19}F NMR, HRMS, HPLC spectra of B1

^{1}H NMR

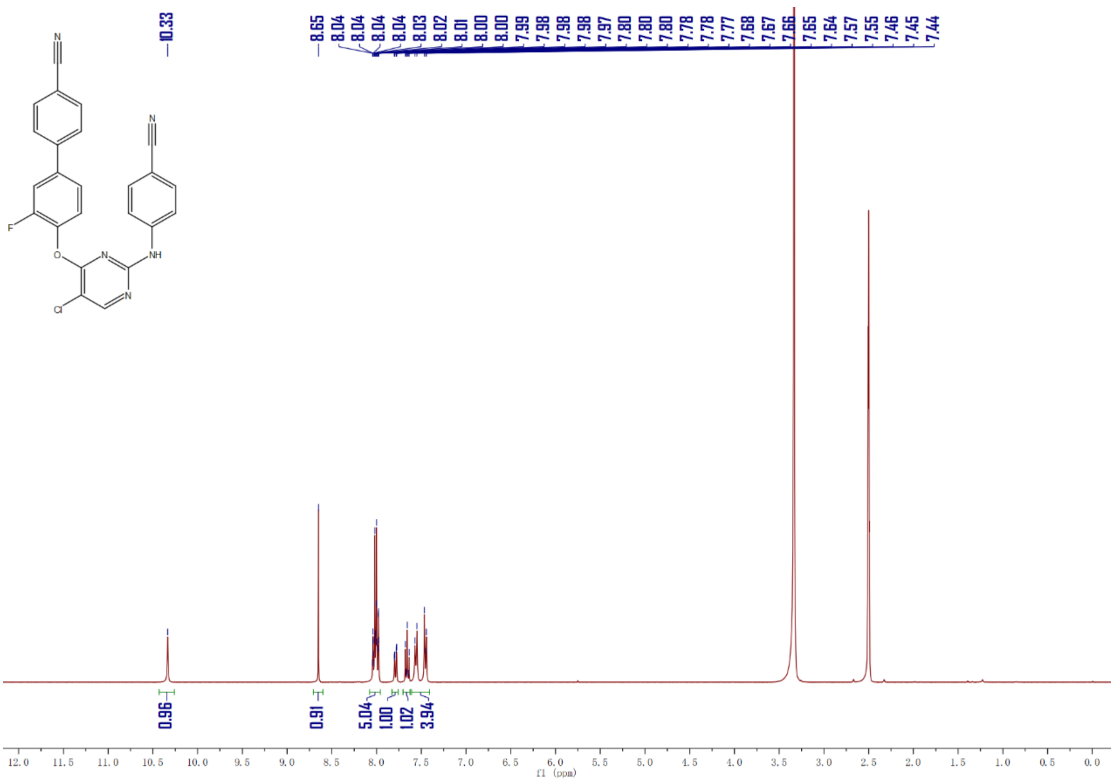

^{13}C NMR

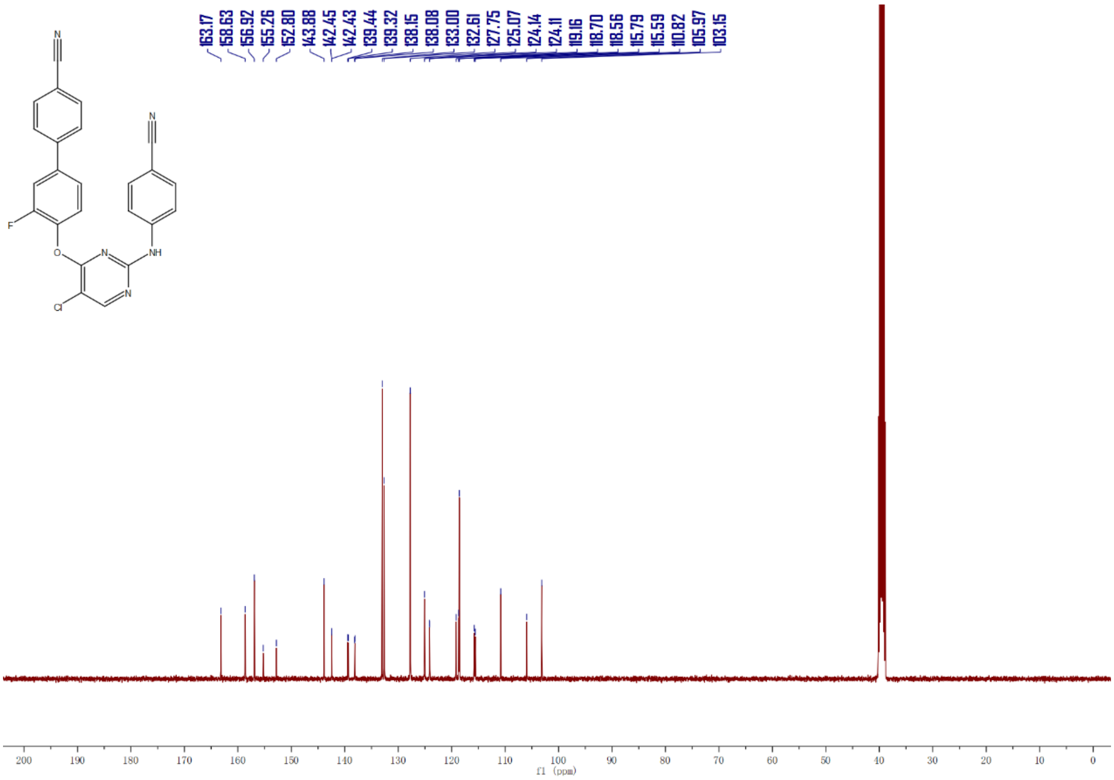

^{19}F NMR

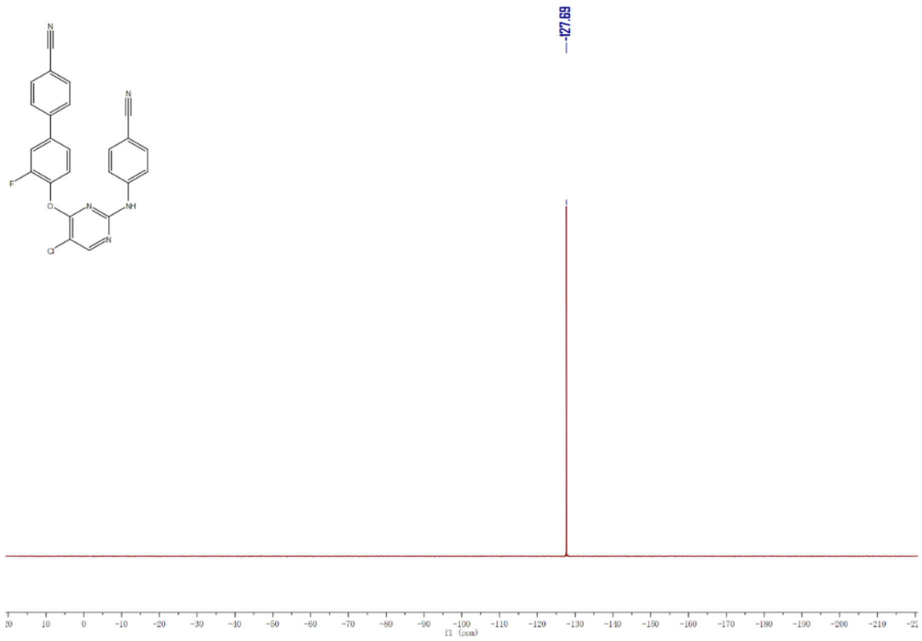

HRMS

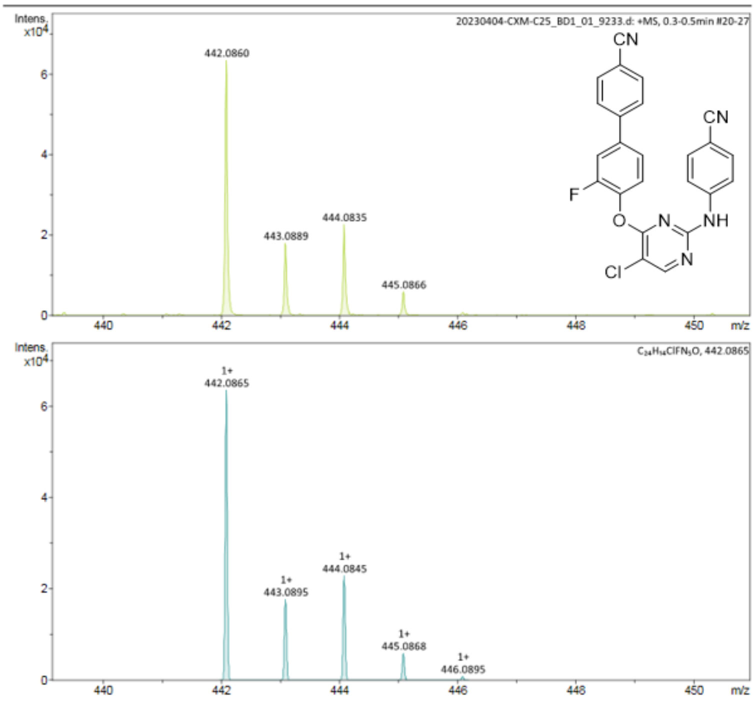

Display Report

Analysis Info

Analysis Name	D:\CXM\20230404\20230404-CXM-C25_BD1_01_9233.d	Acquisition Date	4/4/2023 5:24:49 PM
Method	MS-2MIN-POS.m	Operator	BDAL@DE
Sample Name	20230404-CXM-C25	Instrument	compact 8255754.20127
Comment			

Acquisition Parameter

Source Type	ESI	Ion Polarity	Positive	Set Nebulizer	2.0 Bar
Focus	Active	Set Capillary	4500 V	Set Dry Heater	200 °C
Scan Begin	50 m/z	Set End Plate Offset	-500 V	Set Dry Gas	8.0 l/min
Scan End	3000 m/z	Set Charging Voltage	2000 V	Set Divert Valve	Waste
		Set Corona	0 nA	Set APCI Heater	0 °C

20230404-CXM-C25_BD1_01_9233.d

Bruker Compass DataAnalysis 4.2 printed: 4/9/2023 5:12:07 PM by: BDAL@DE Page 1 of 1

HPLC

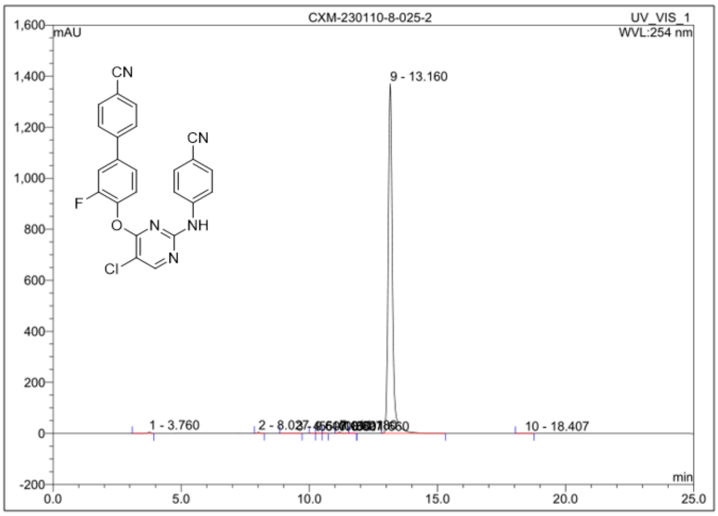

No.	Ret.Time min	Peak Name	Height mAU	Area mAU*s	Rel.Area %	Amount	Type
1	3.76	n.a.	4.617	54.35	0.35	n.a.	BMB
2	8.03	n.a.	3.172	21.36	0.14	n.a.	BMB
3	9.51	n.a.	0.536	17.79	0.11	n.a.	BMB*
4	10.16	n.a.	0.303	1.95	0.01	n.a.	BMb*
5	10.38	n.a.	0.472	3.11	0.02	n.a.	bMB*
6	10.63	n.a.	1.086	6.53	0.04	n.a.	BMB
7	11.18	n.a.	4.079	39.33	0.25	n.a.	BMB
8	11.66	n.a.	0.341	2.66	0.02	n.a.	Rd
9	13.16	n.a.	1369.861	15492.74	99.03	n.a.	BMB
10	18.41	n.a.	0.237	4.51	0.03	n.a.	BMB
Total:			1384.704	15644.321	100.00	0.000	

^{1}H NMR, ^{13}C NMR, HRMS, HPLC spectra of B2

^{1}H NMR

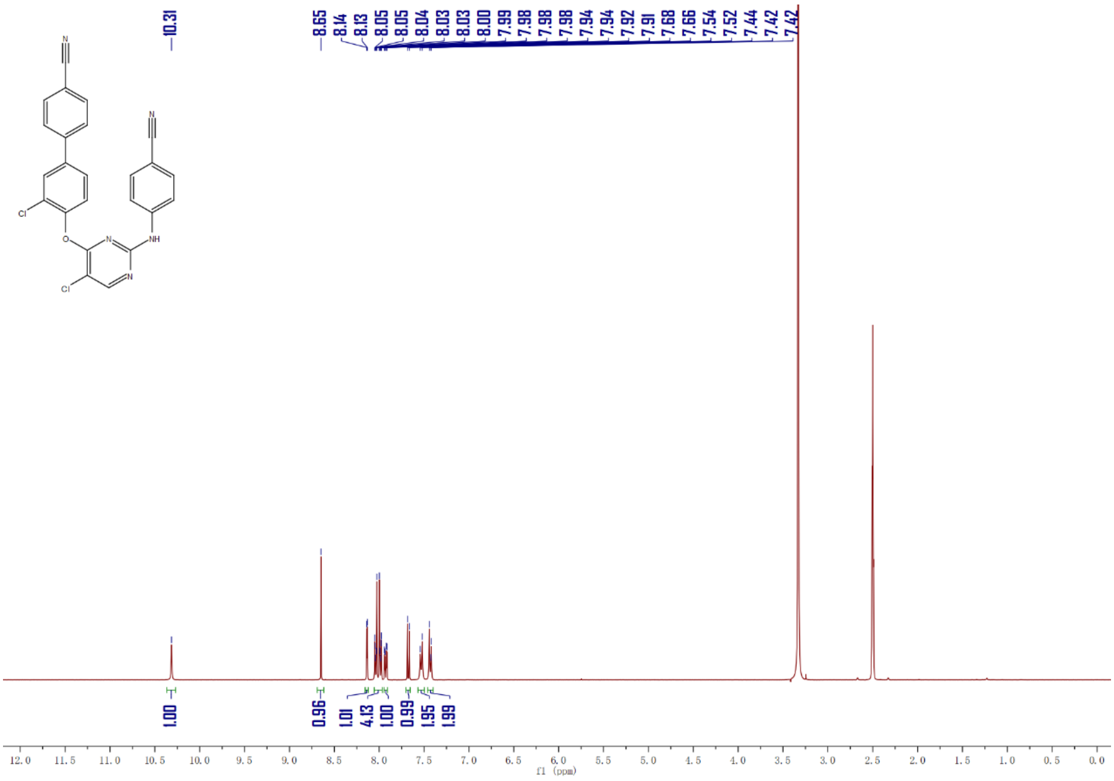

^{13}C NMR

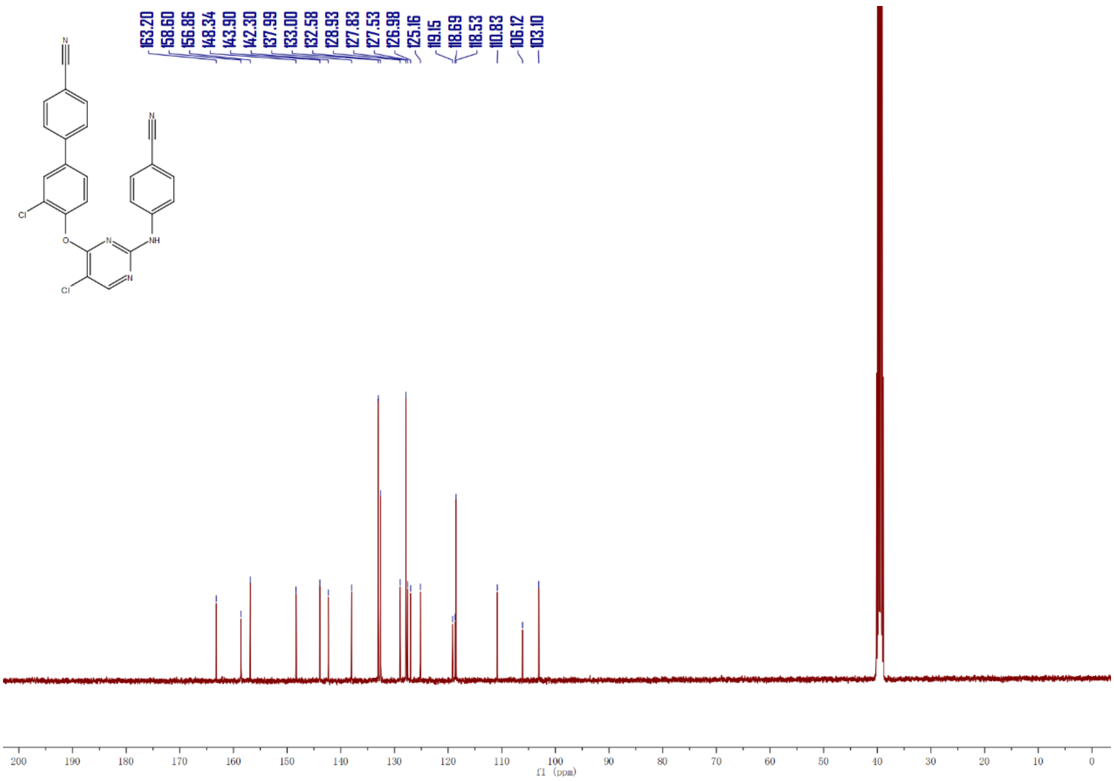

163.20
158.60
156.86
148.34
143.90
142.30
137.99
133.00
132.58
128.93
127.83
127.53
126.98
125.16
119.15
118.69
118.53
110.83
106.12
103.10
200 190 180 170 160 150 140 130 120 110 100 90 80 70 60 50 40 30 20 10 0
f1 (ppm)

HRMS

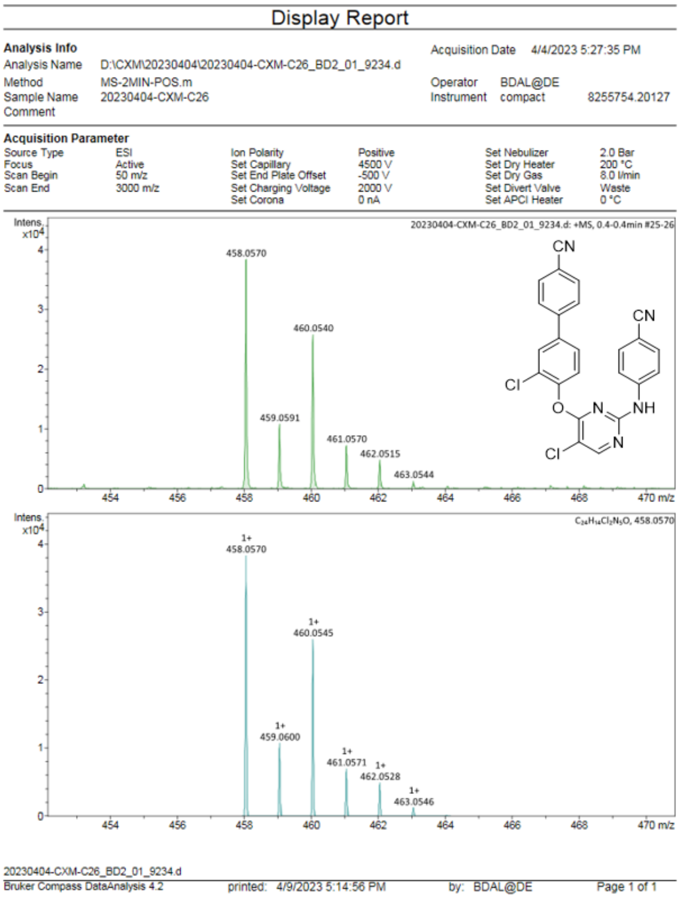

Display Report
Analysis Info
Analysis Name D:\CXM\20230404\20230404-CXM-C26_BD2_01_9234.d
Method MS-2MIN-POS.m
Sample Name 20230404-CXM-C26
Comment
Acquisition Date 4/4/2023 5:27:35 PM
Operator BDAL@DE
Instrument compact 8255754.20127
Acquisition Parameter
Source Type ESI
Focus Active
Scan Begin 50 m/z
Scan End 3000 m/z
Ion Polarity Positive
Set Capillary 4500 V
Set End Plate Offset -500 V
Set Charging Voltage 2000 V
Set Corona 0 nA
Set Nebulizer 2.0 Bar
Set Dry Heater 200 °C
Set Dry Gas 8.0 l/min
Set Divert Valve Waste
Set APCI Heater 0 °C
Intens. x10^4
20230404-CXM-C26_BD2_01_9234.d: +MS, 0.4-0.4min #25-26
458.0570
460.0540
459.0591
461.0570
462.0515
463.0544
CN
CN
Cl
O
N
NH
Cl
N
454 456 458 460 462 464 466 468 470 m/z
Intens. x10^4
C24H14Cl2N5O, 458.0570
1+ 458.0570
1+ 460.0545
1+ 459.0600
1+ 461.0571
1+ 462.0528
1+ 463.0546
454 456 458 460 462 464 466 468 470 m/z
20230404-CXM-C26_BD2_01_9234.d
Bruker Compass DataAnalysis 4.2
printed: 4/9/2023 5:14:56 PM
by: BDAL@DE
Page 1 of 1

HPLC

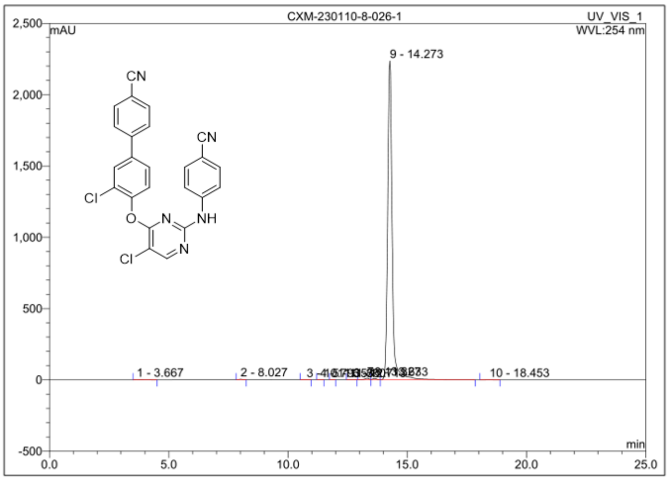

No.	Ret.Time min	Peak Name	Height mAU	Area mAU*s	Rel.Area %	Amount	Type
1	3.67	n.a.	2.273	56.49	0.19	n.a.	BMB*
2	8.03	n.a.	3.569	30.09	0.10	n.a.	BMB
3	10.79	n.a.	0.556	7.68	0.03	n.a.	BMB
4	11.35	n.a.	0.598	4.81	0.02	n.a.	BMB
5	11.86	n.a.	0.730	5.23	0.02	n.a.	BMB
6	12.71	n.a.	0.886	8.56	0.03	n.a.	BMB
7	13.33	n.a.	10.464	134.68	0.45	n.a.	BM *
8	13.63	n.a.	8.769	97.65	0.33	n.a.	Mb*
9	14.27	n.a.	2236.374	29535.29	98.82	n.a.	bMB*
10	18.45	n.a.	0.360	6.87	0.02	n.a.	BMB*
Total:			2264.578	29887.354	100.00	0.000	

^{1}H NMR, ^{13}C NMR, HRMS, HPLC spectra of B3

^{1}H NMR

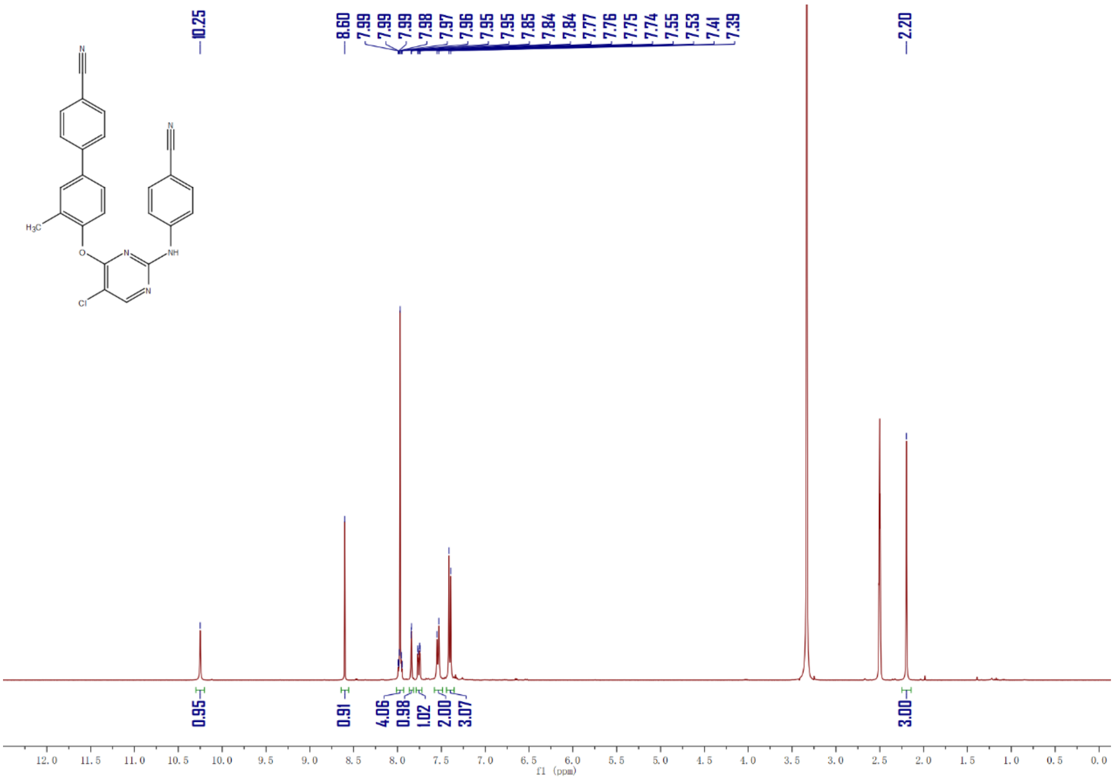

^{13}C NMR

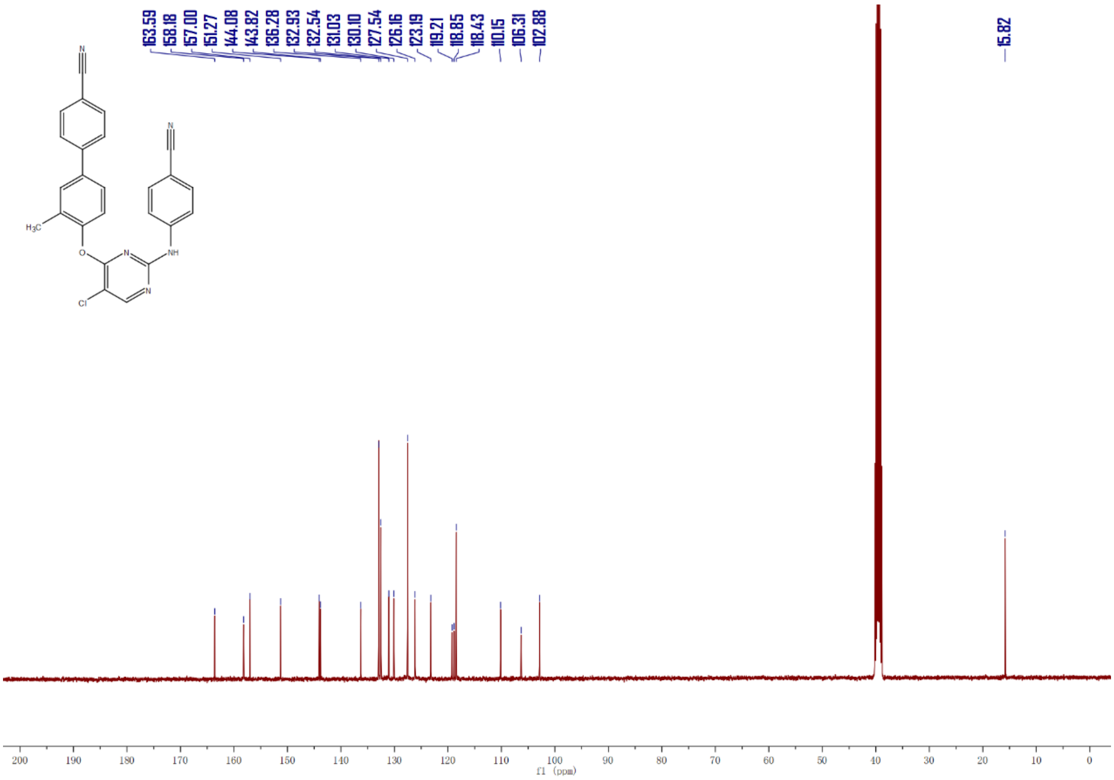

HRMS

Display Report

**Analysis Info**		Acquisition Date	4/4/2023 5:30:22 PM
Analysis Name	D:\CXM\20230404\20230404-CXM-C27_BD3_01_9235.d		
Method	MS-2MIN-POS.m	Operator	BDAL@DE
Sample Name	20230404-CXM-C27	Instrument	compact 8255754.20127
Comment			

Acquisition Parameter

Source Type	ESI	Ion Polarity	Positive	Set Nebulizer	2.0 Bar
Focus	Active	Set Capillary	4500 V	Set Dry Heater	200 °C
Scan Begin	50 m/z	Set End Plate Offset	-500 V	Set Dry Gas	8.0 l/min
Scan End	3000 m/z	Set Charging Voltage	2000 V	Set Divert Valve	Waste
		Set Corona	0 nA	Set APCI Heater	0 °C

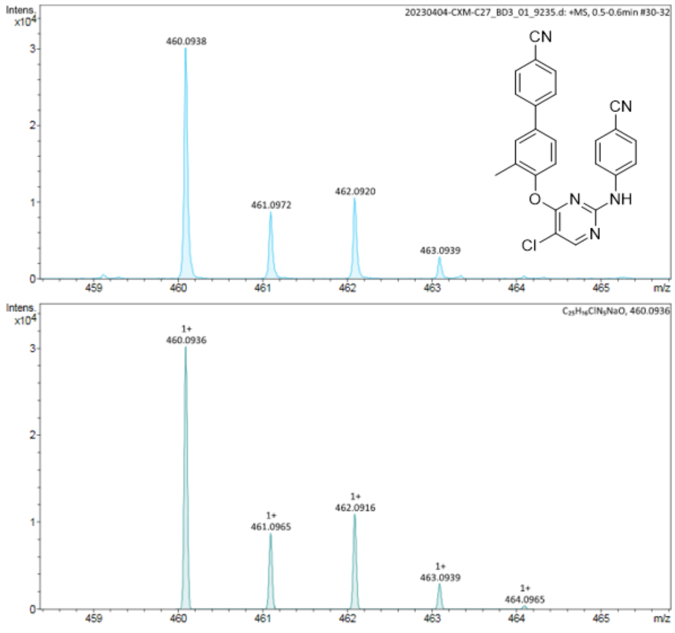

20230404-CXM-C27_BD3_01_9235.d
Bruker Compass DataAnalysis 4.2 printed: 4/9/2023 5:20:26 PM by: BDAL@DE Page 1 of 1

HPLC

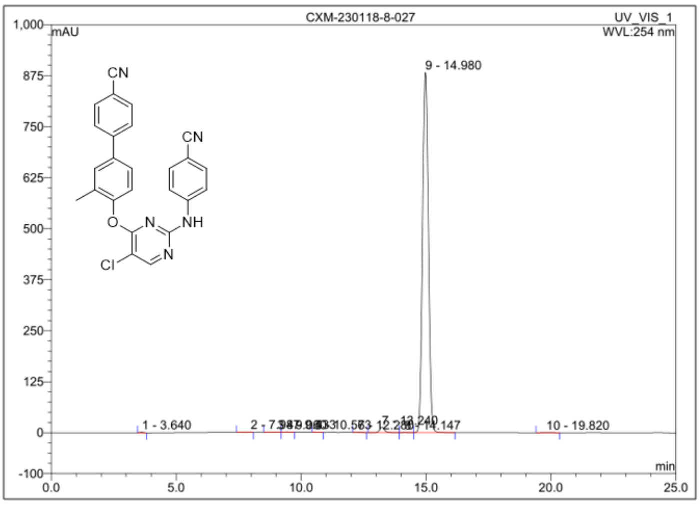

No.	Ret.Time min	Peak Name	Height mAU	Area mAU*s	Rel.Area %	Amount	Type
1	3.64	n.a.	1.362	15.29	0.10	n.a.	BMB*
2	7.99	n.a.	1.012	14.06	0.10	n.a.	BMB*
3	9.06	n.a.	0.561	10.77	0.07	n.a.	BM
4	9.43	n.a.	0.921	12.00	0.08	n.a.	MB
5	10.57	n.a.	0.655	8.33	0.06	n.a.	BMB
6	12.28	n.a.	0.438	7.16	0.05	n.a.	BMB
7	13.24	n.a.	12.693	173.53	1.18	n.a.	BMB
8	14.15	n.a.	0.294	4.04	0.03	n.a.	BMB
9	14.98	n.a.	882.474	14391.06	98.26	n.a.	BMB
10	19.82	n.a.	0.388	10.14	0.07	n.a.	BMB
Total:			900.797	14646.376	100.00	0.000	

^{1}H NMR, ^{13}C NMR, HRMS, HPLC spectra of B4

^{1}H NMR

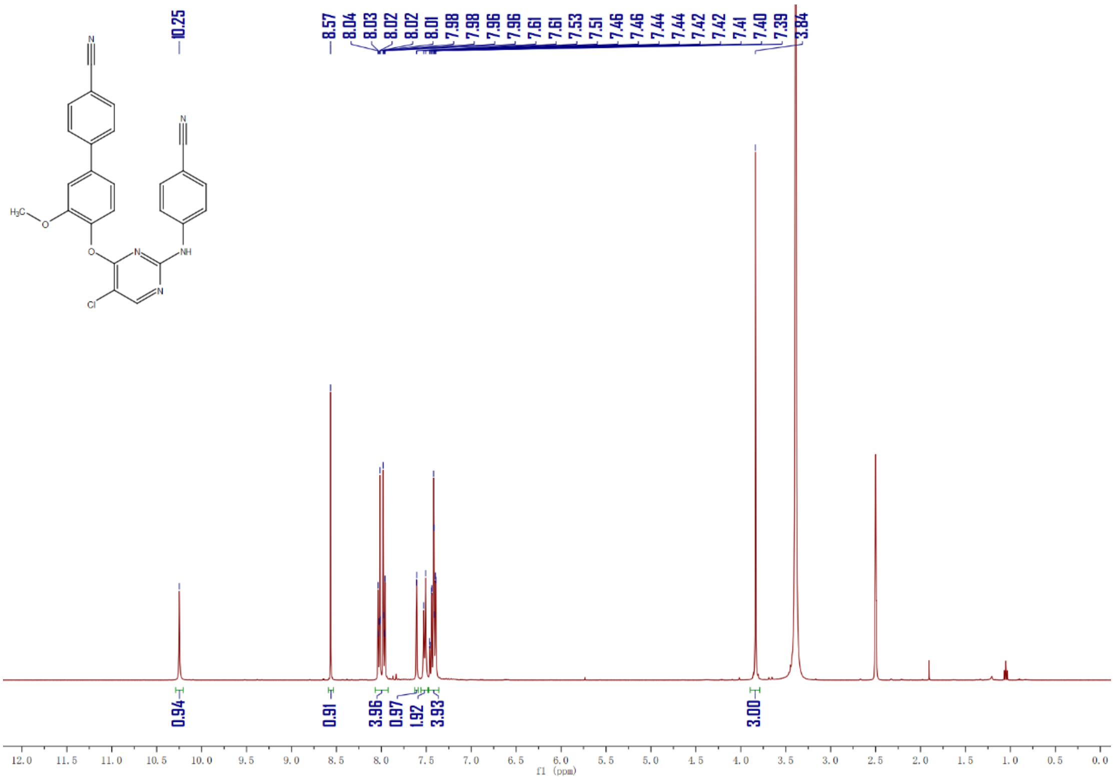

^{13}C NMR

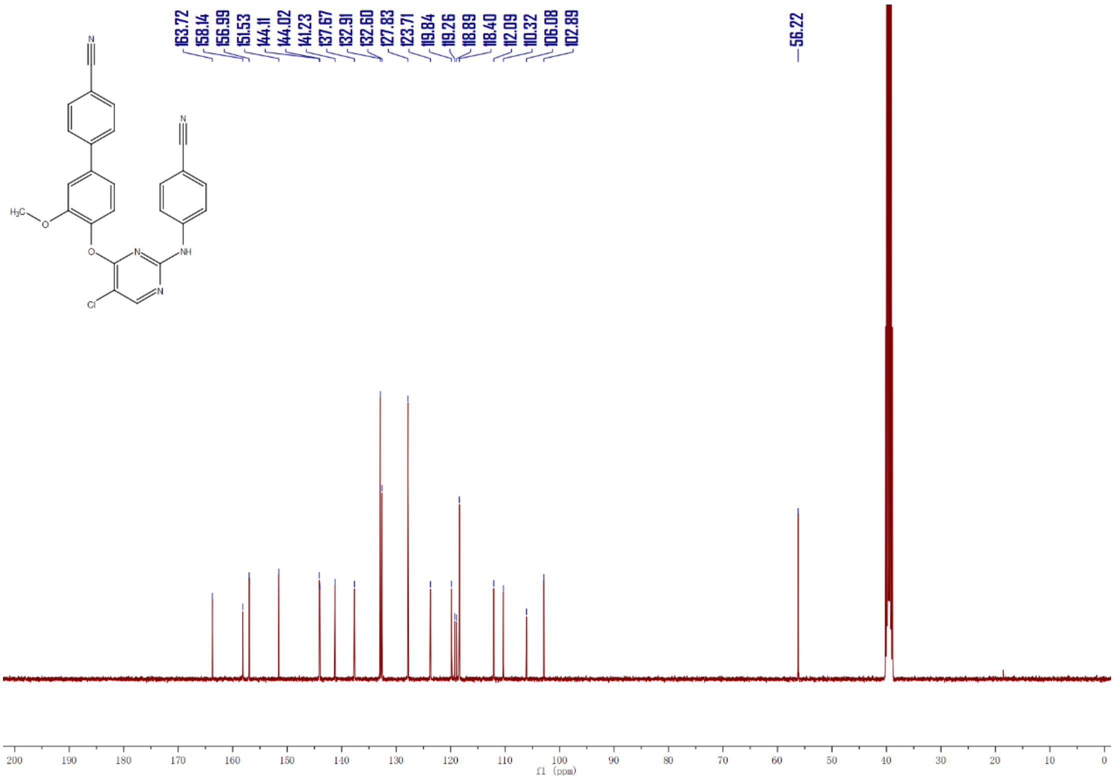

HRMS

Display Report

Analysis Info

Analysis Name	D:\CXM\20230404\20230404-CXM-C28_BD4_01_9236.d	Acquisition Date	4/4/2023 5:33:06 PM
Method	MS-2MIN-POS.m	Operator	BDAL@DE
Sample Name	20230404-CXM-C28	Instrument	compact 8255754.20127
Comment			

Acquisition Parameter

Source Type	ESI	Ion Polarity	Positive	Set Nebulizer	2.0 Bar
Focus	Active	Set Capillary	4500 V	Set Dry Heater	200 °C
Scan Begin	50 m/z	Set End Plate Offset	-500 V	Set Dry Gas	8.0 l/min
Scan End	3000 m/z	Set Charging Voltage	2000 V	Set Divert Valve	Waste
		Set Corona	0 nA	Set APCI Heater	0 °C

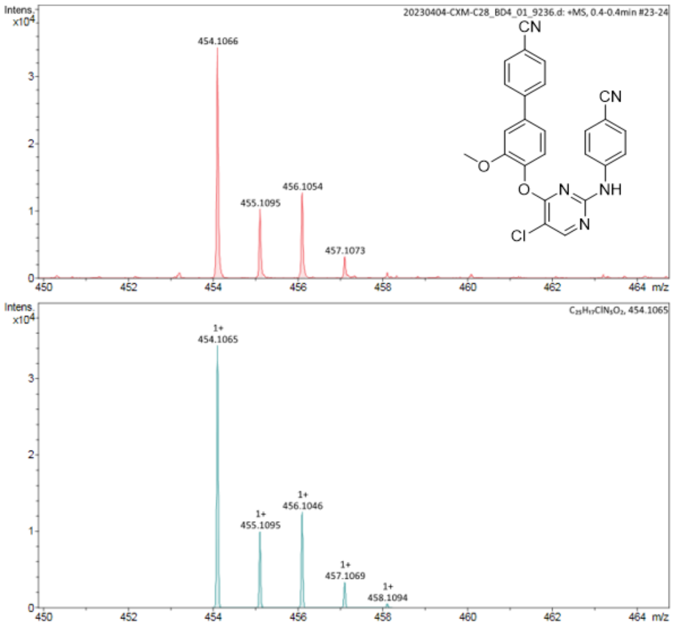

20230404-CXM-C28_BD4_01_9236.d
Bruker Compass DataAnalysis 4.2 printed: 4/9/2023 5:25:51 PM by: BDAL@DE Page 1 of 1

HPLC

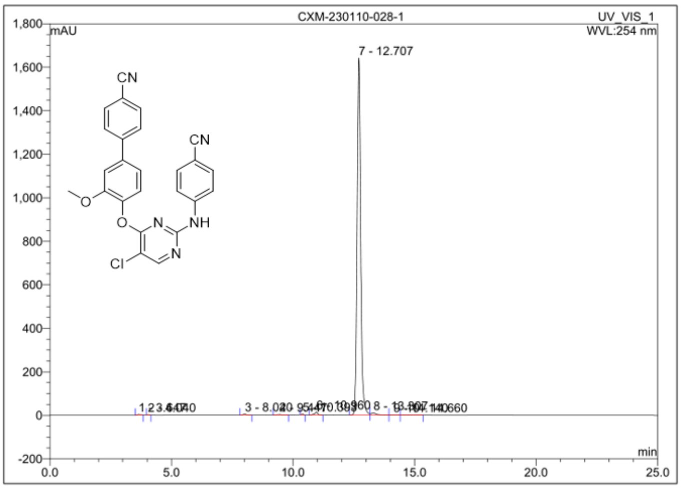

No.	Ret.Time min	Peak Name	Height mAU	Area mAU*s	Rel.Area %	Amount	Type
1	3.65	n.a.	2.449	25.15	0.15	n.a.	BMB
2	4.04	n.a.	1.188	7.04	0.04	n.a.	BMB
3	8.02	n.a.	4.329	35.73	0.21	n.a.	BMB
4	9.45	n.a.	1.656	22.22	0.13	n.a.	BMB
5	10.39	n.a.	2.889	15.36	0.09	n.a.	BMB
6	10.96	n.a.	13.083	118.21	0.68	n.a.	BMB
7	12.71	n.a.	1641.798	16872.22	97.70	n.a.	BM *
8	13.31	n.a.	9.940	152.59	0.88	n.a.	M *
9	14.14	n.a.	0.410	6.36	0.04	n.a.	MB*
10	14.66	n.a.	0.590	14.14	0.08	n.a.	BMB*
Total:			1678.333	17269.015	100.00	0.000	

^{1}H NMR, ^{13}C NMR, HRMS, HPLC spectra of B5

^{1}H NMR

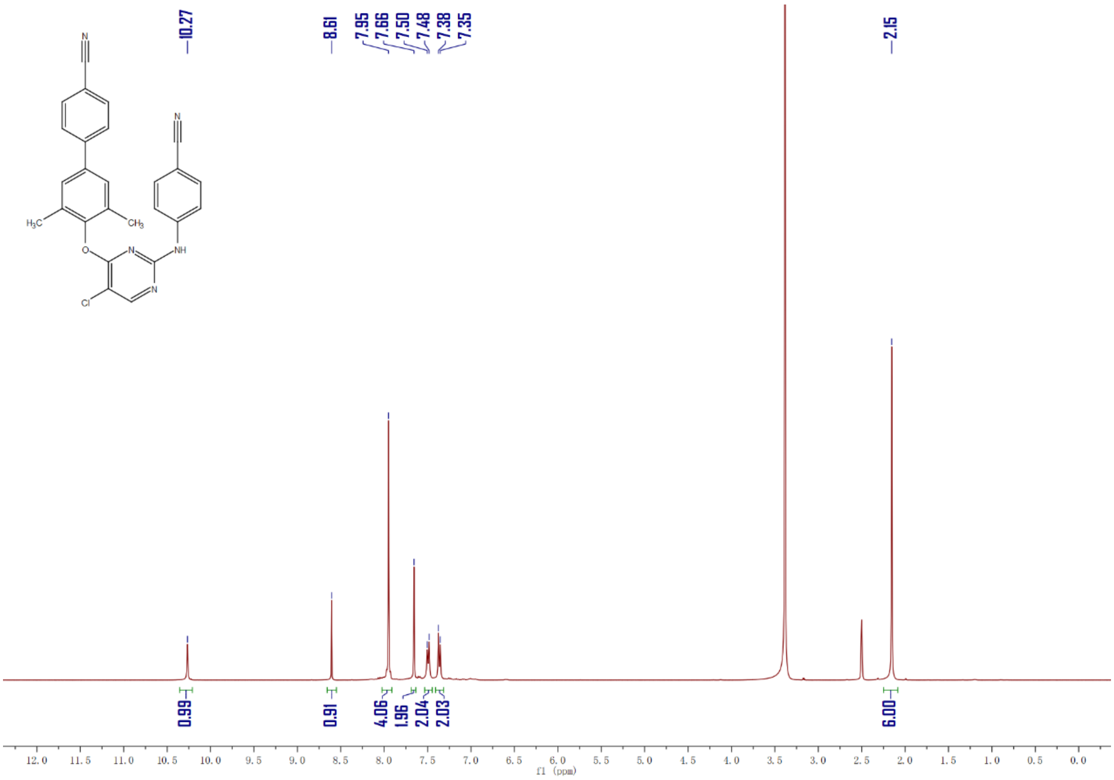

^{13}C NMR

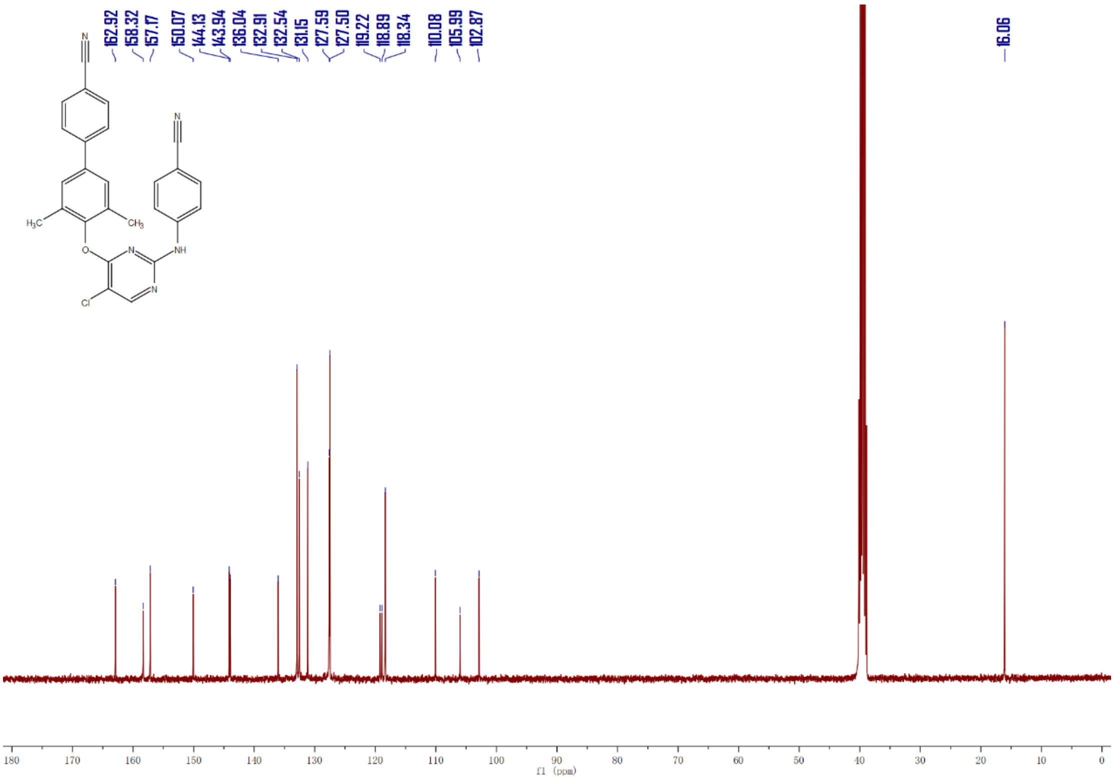

162.92
158.32
157.17
150.07
144.13
143.94
136.04
132.91
132.54
131.15
127.59
127.50
119.22
118.89
118.34
110.08
105.99
102.87
16.06
f1 (ppm)

HRMS

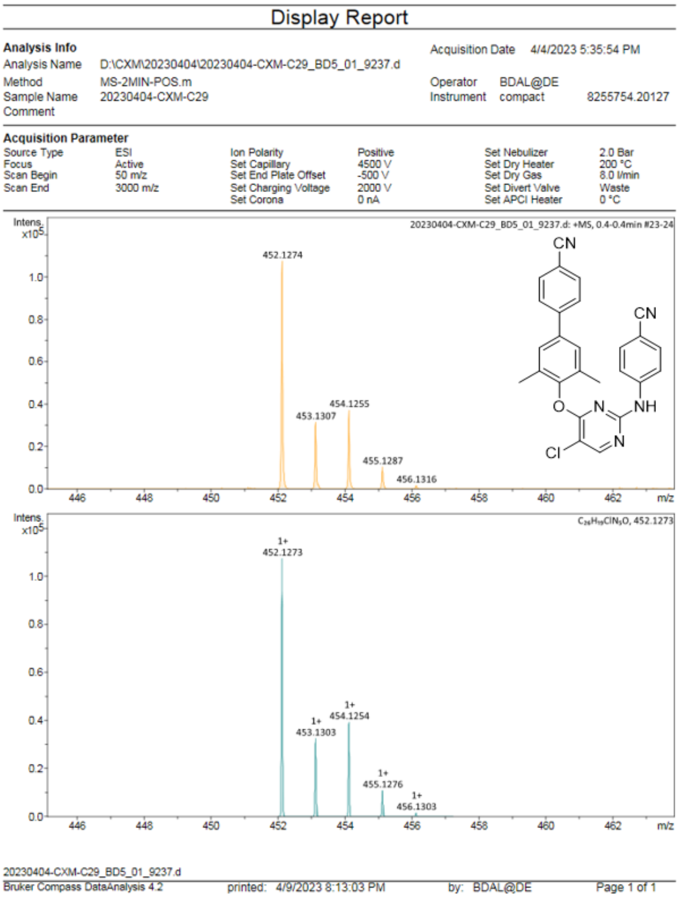

Display Report
Analysis Info
Analysis Name D:\CXM\20230404\20230404-CXM-C29_BD5_01_9237.d
Method MS-2MIN-POS.m
Sample Name 20230404-CXM-C29
Comment
Acquisition Date 4/4/2023 5:35:54 PM
Operator BDAL@DE
Instrument compact 8255754.20127
Acquisition Parameter
Source Type ESI
Focus Active
Scan Begin 50 m/z
Scan End 3000 m/z
Ion Polarity Positive
Set Capillary 4500 V
Set End Plate Offset -500 V
Set Charging Voltage 2000 V
Set Corona 0 nA
Set Nebulizer 2.0 Bar
Set Dry Heater 200 °C
Set Dry Gas 8.0 l/min
Set Divert Valve Waste
Set APCI Heater 0 °C
20230404-CXM-C29_BD5_01_9237.d: +MS, 0.4-0.4min #23-24
452.1274
453.1307
454.1255
455.1287
456.1316
C29H19ClN5O, 452.1273
452.1273
453.1303
454.1254
455.1276
456.1303
m/z
20230404-CXM-C29_BD5_01_9237.d
Bruker Compass DataAnalysis 4.2
printed: 4/9/2023 8:13:03 PM
by: BDAL@DE
Page 1 of 1

HPLC

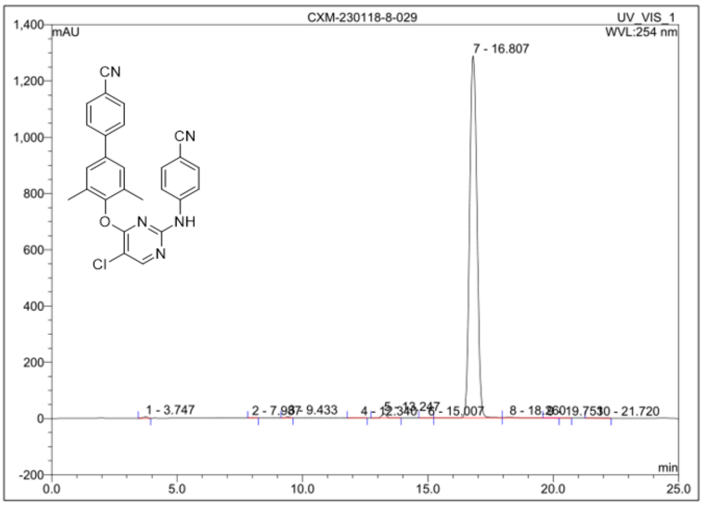

No.	Ret.Time min	Peak Name	Height mAU	Area mAU*s	Rel.Area %	Amount	Type
1	3.75	n.a.	5.374	64.02	0.24	n.a.	BMB*
2	7.99	n.a.	1.335	9.69	0.04	n.a.	BMB
3	9.43	n.a.	4.103	38.20	0.14	n.a.	BMB
4	12.34	n.a.	0.418	11.04	0.04	n.a.	BMB*
5	13.25	n.a.	16.371	218.06	0.81	n.a.	BMB
6	15.01	n.a.	0.365	7.50	0.03	n.a.	BM
7	16.81	n.a.	1287.854	26230.96	98.03	n.a.	M
8	18.26	n.a.	2.332	166.71	0.62	n.a.	MB
9	19.75	n.a.	0.172	4.63	0.02	n.a.	Rd
10	21.72	n.a.	0.271	8.15	0.03	n.a.	BMB
Total:			1318.595	26758.960	100.00	0.000	

^{1}H NMR, ^{13}C NMR, ^{19}F NMR, HRMS, HPLC spectra of B6

^{1}H NMR

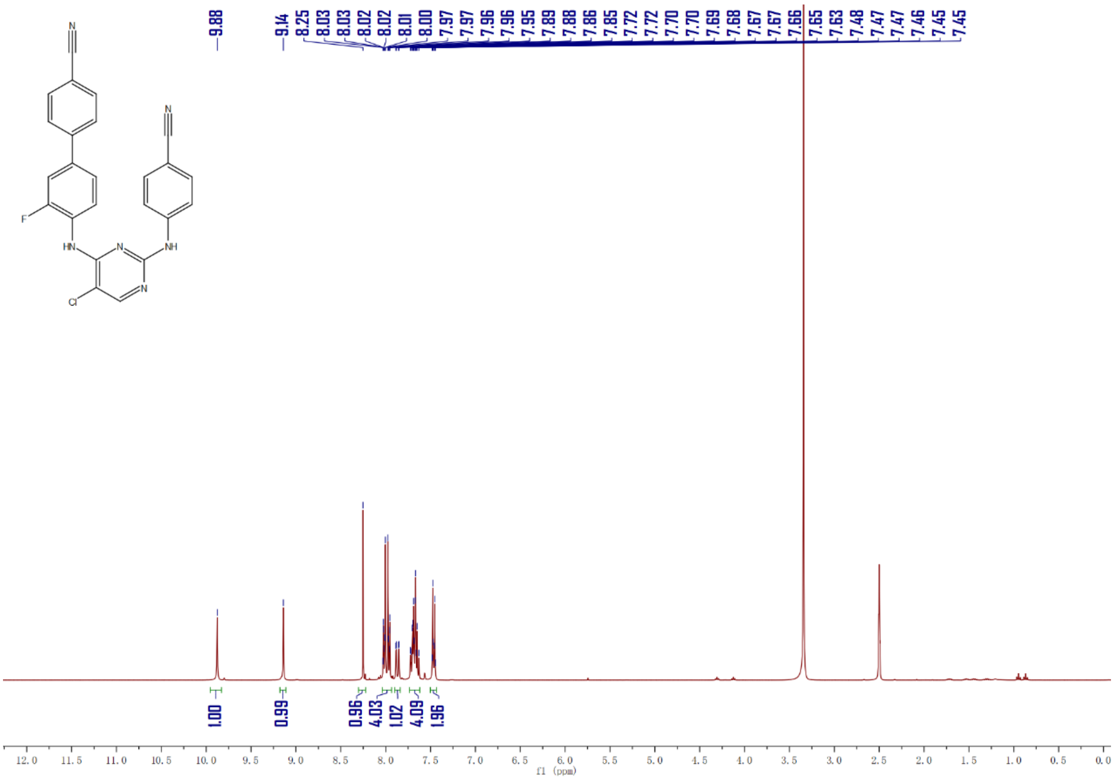

^{13}C NMR

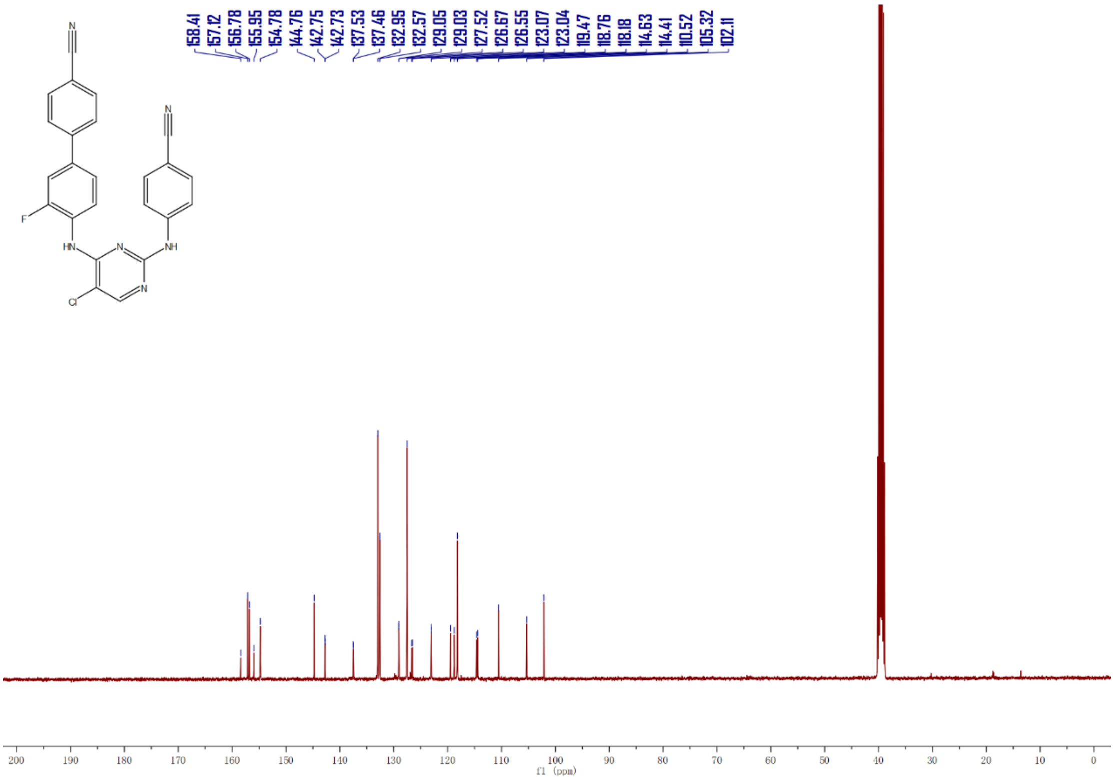

^{19}F NMR

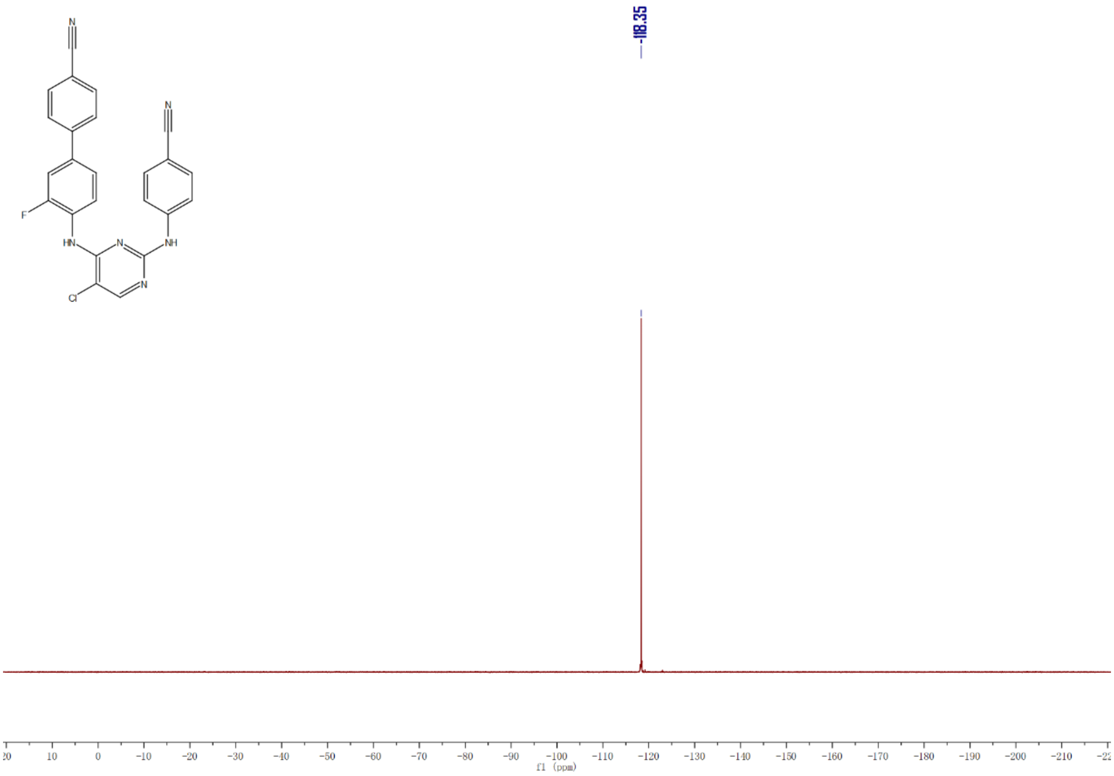

HRMS

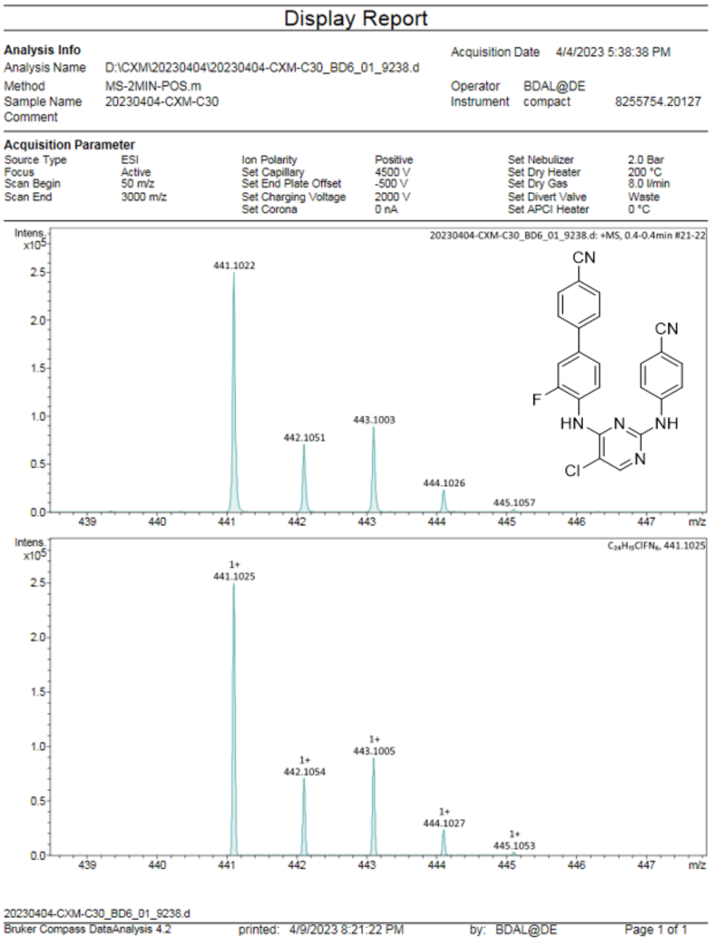

HPLC

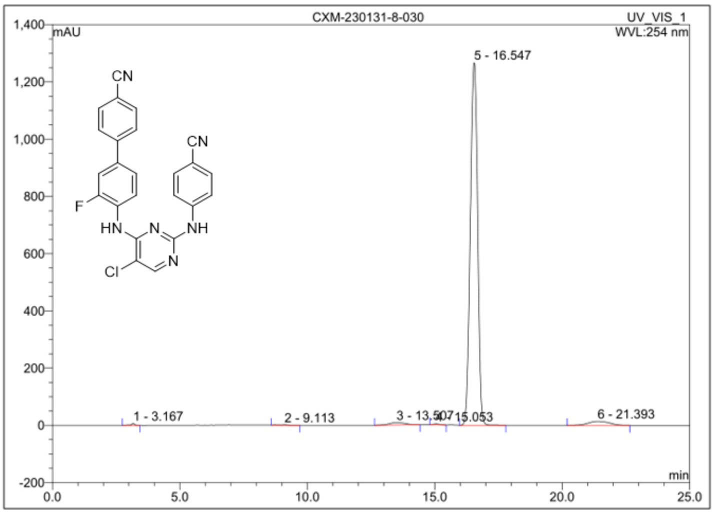

No.	Ret.Time min	Peak Name	Height mAU	Area mAU*s	Rel.Area %	Amount	Type
1	3.17	n.a.	6.592	56.36	0.21	n.a.	BMB
2	9.11	n.a.	1.357	18.90	0.07	n.a.	BMB*
3	13.51	n.a.	7.917	355.82	1.33	n.a.	BMB*
4	15.05	n.a.	2.682	47.55	0.18	n.a.	BMB*
5	16.55	n.a.	1267.033	25396.27	95.16	n.a.	BMB*
6	21.39	n.a.	13.925	813.55	3.05	n.a.	BMB*
Total:			1299.507	26688.452	100.00	0.000	

^{1}H NMR, ^{13}C NMR, ^{19}F NMR, HRMS, HPLC spectra of B7

^{1}H NMR

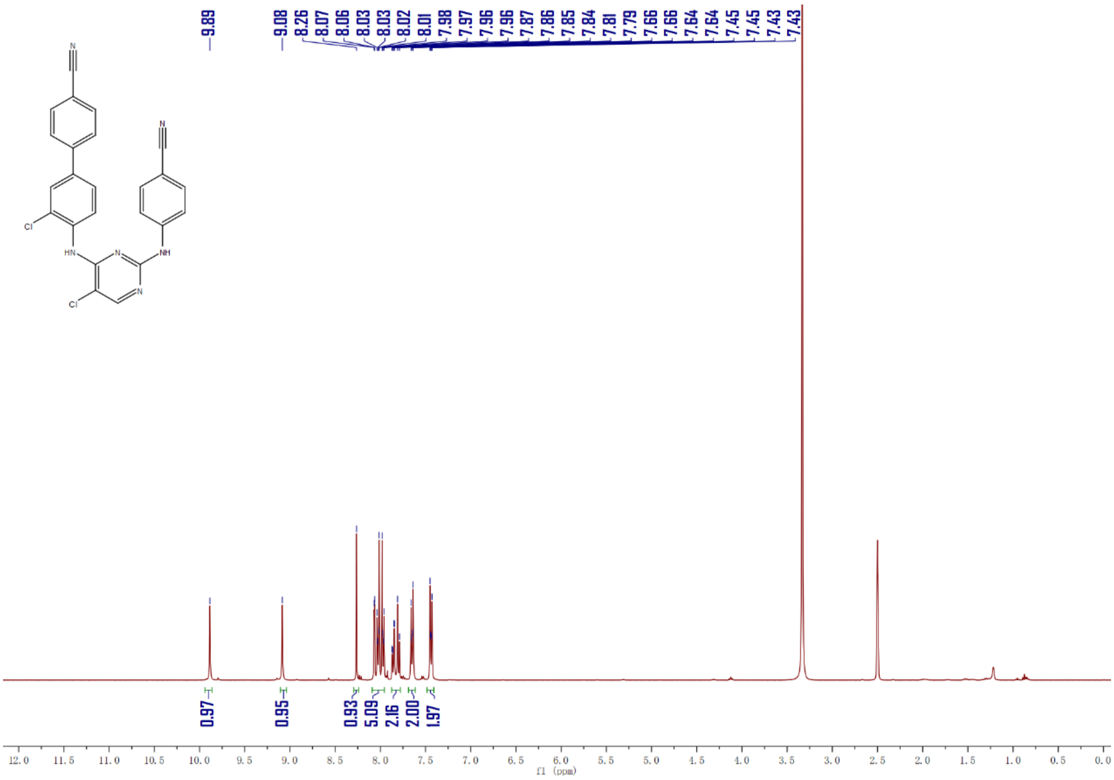

^{13}C NMR

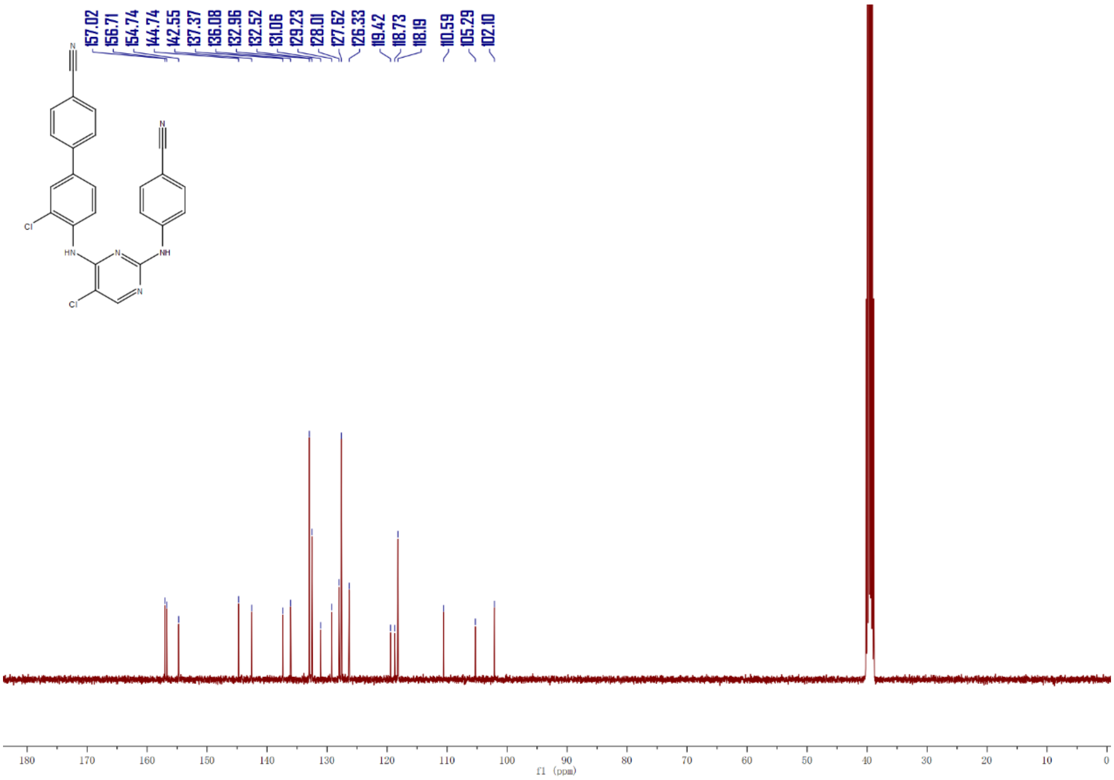

^{19}F NMR

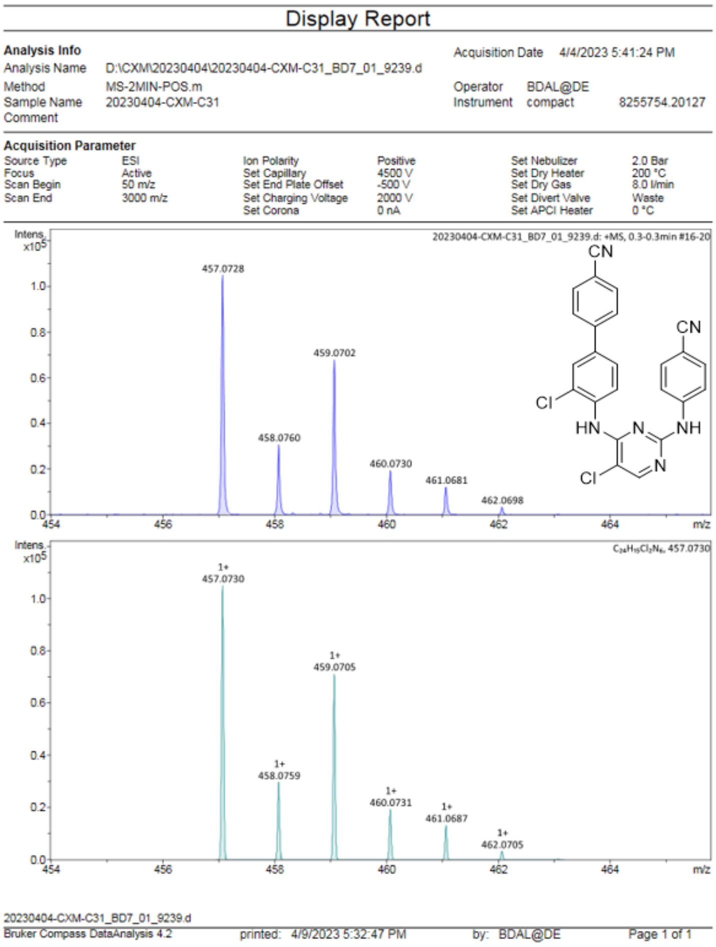

HRMS

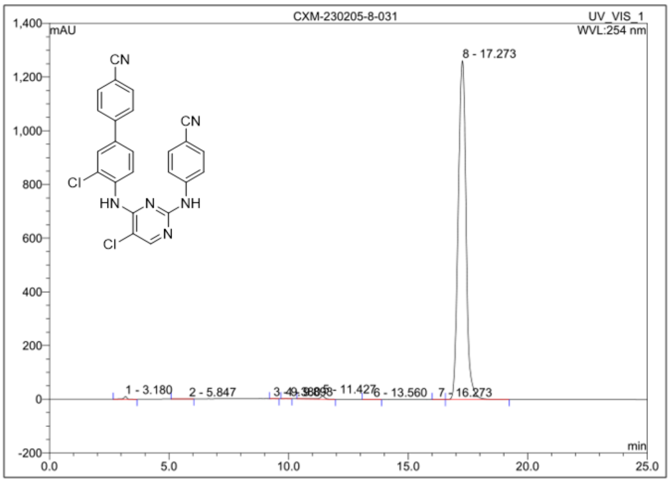

No.	Ret.Time min	Peak Name	Height mAU	Area mAU*s	Rel.Area %	Amount	Type
1	3.18	n.a.	10.416	99.26	0.34	n.a.	BMB*
2	5.85	n.a.	0.287	4.64	0.02	n.a.	BMB
3	9.38	n.a.	0.569	4.50	0.02	n.a.	BMB
4	9.89	n.a.	0.303	3.54	0.01	n.a.	BMB
5	11.43	n.a.	11.575	164.72	0.57	n.a.	BMB
6	13.56	n.a.	0.564	12.05	0.04	n.a.	BMB
7	16.27	n.a.	0.701	11.95	0.04	n.a.	BM
8	17.27	n.a.	1262.617	28694.35	98.96	n.a.	MB
Total:			1287.032	28995.002	100.00	0.000	

^{1}H NMR, ^{13}C NMR, HRMS, HPLC spectra of B8

^{1}H NMR

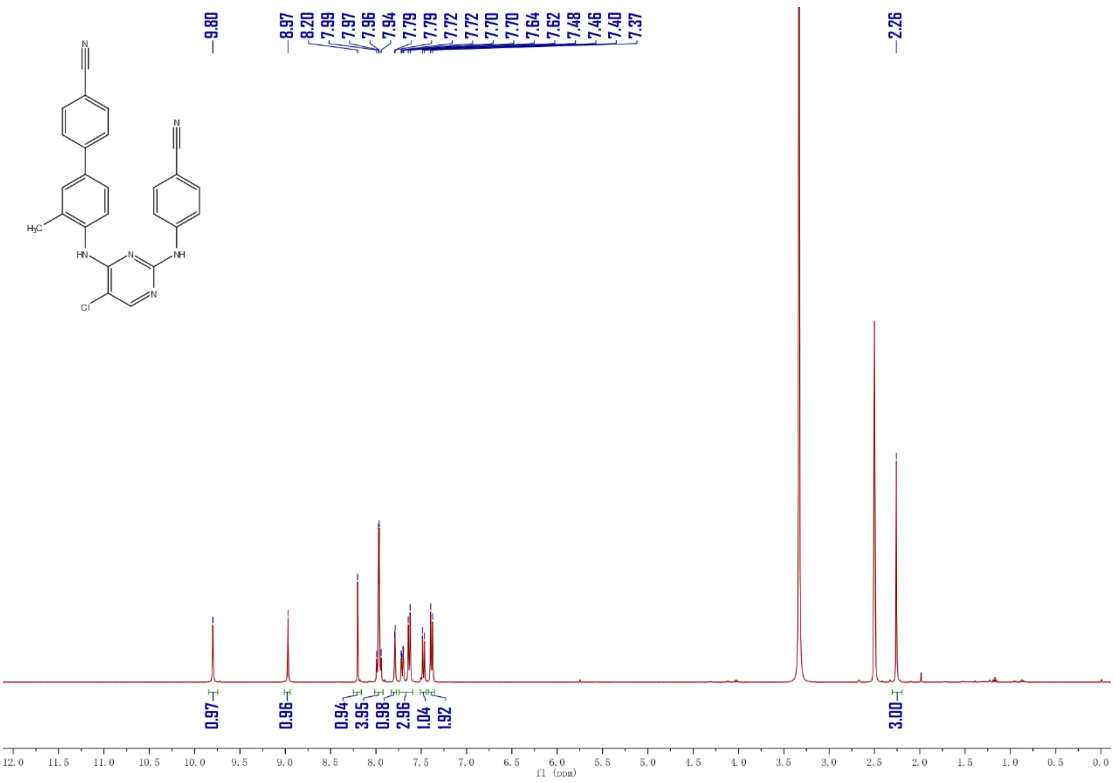

^{13}C NMR

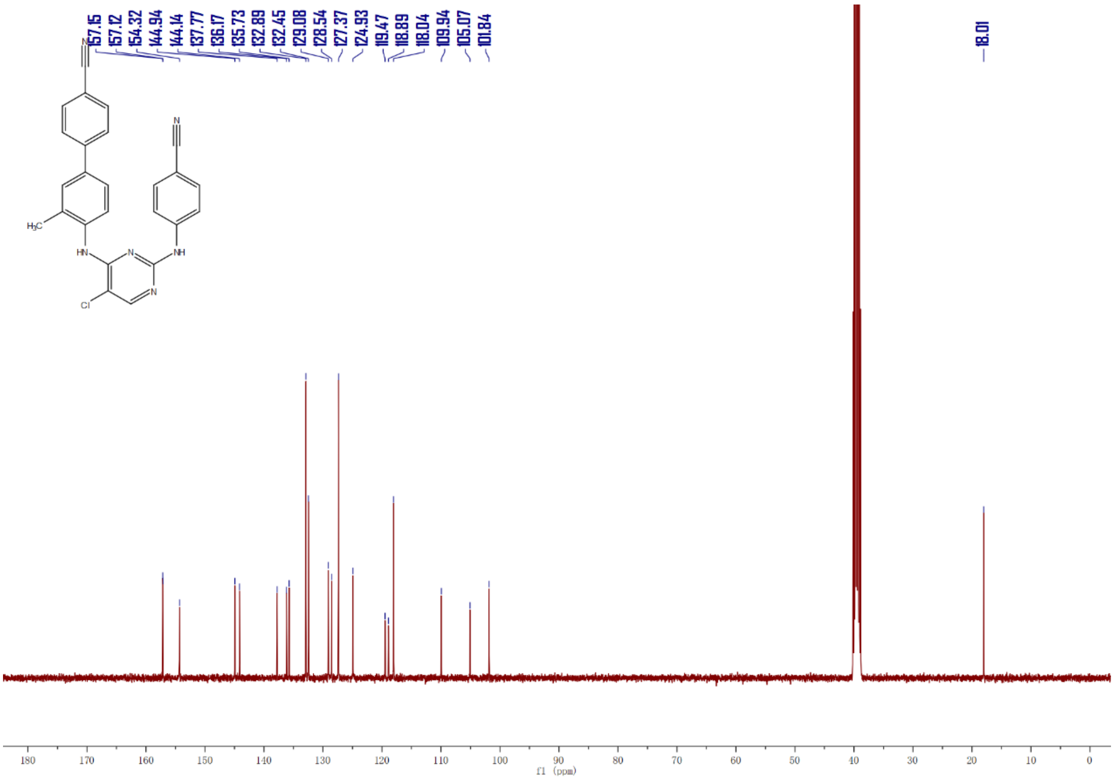

HRMS

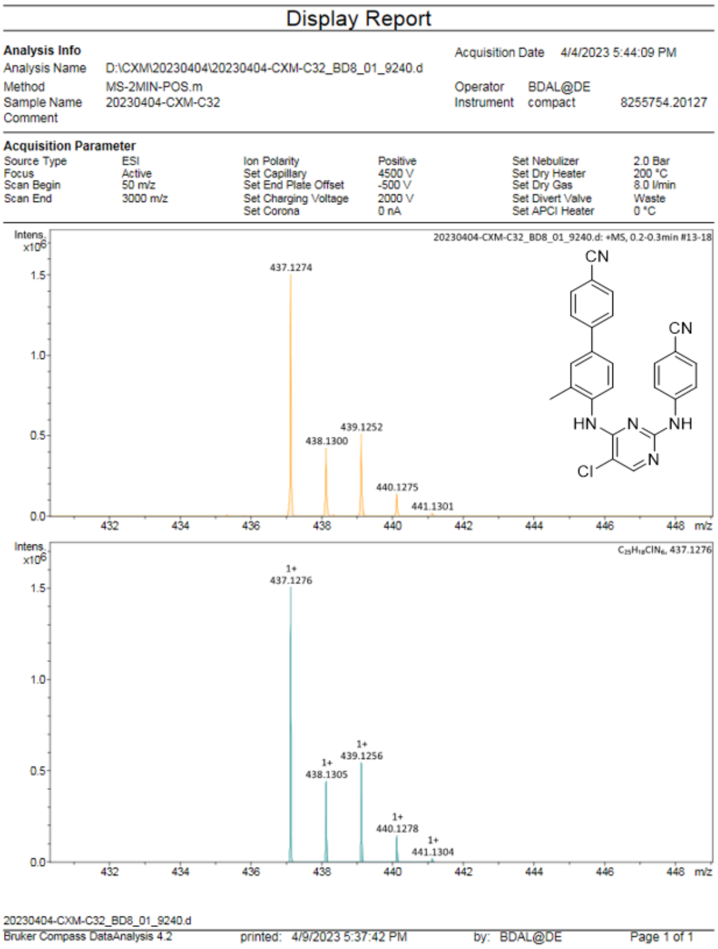

HPLC

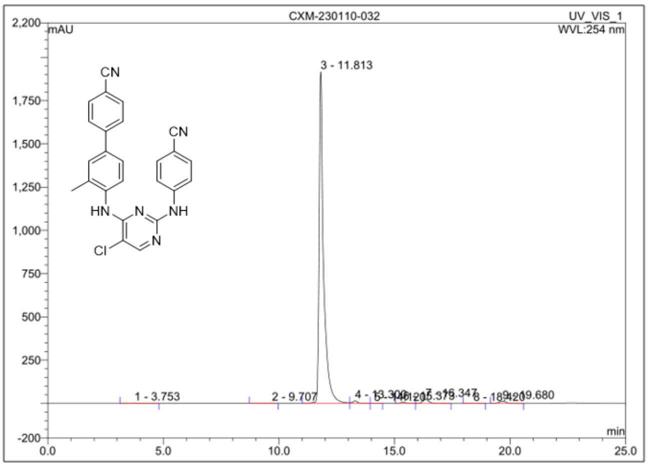

No.	Ret.Time min	Peak Name	Height mAU	Area mAU*s	Rel.Area %	Amount	Type
1	3.75	n.a.	2.647	27.07	0.10	n.a.	BMB*
2	9.71	n.a.	0.764	27.33	0.10	n.a.	BMB*
3	11.81	n.a.	1916.499	25817.75	95.91	n.a.	BM
4	13.30	n.a.	14.555	224.44	0.83	n.a.	M
5	14.12	n.a.	0.484	7.20	0.03	n.a.	MB
6	15.37	n.a.	6.320	104.35	0.39	n.a.	BM
7	16.35	n.a.	23.215	433.25	1.61	n.a.	MB
8	18.42	n.a.	0.577	12.77	0.05	n.a.	BMB
9	19.68	n.a.	11.600	264.78	0.98	n.a.	BMB
Total:			1976.662	26918.952	100.00	0.000	

^{1}H NMR, ^{13}C NMR, HRMS, HPLC spectra of B9

^{1}H NMR

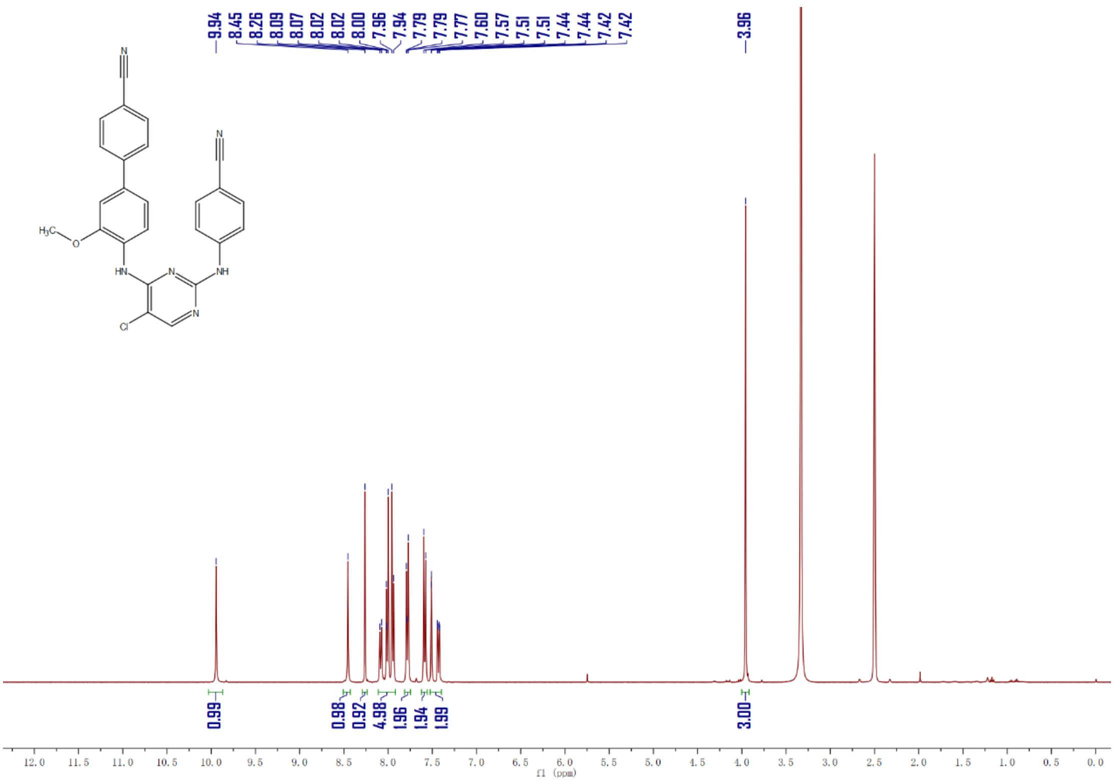

^{13}C NMR

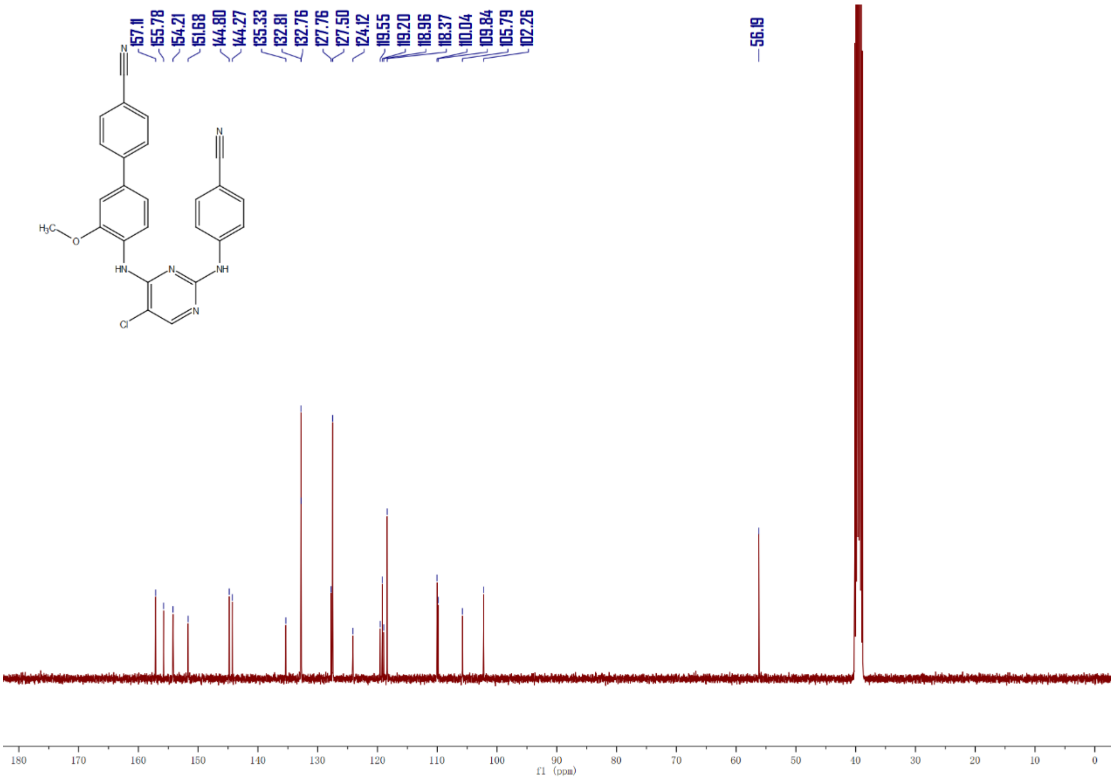

HRMS

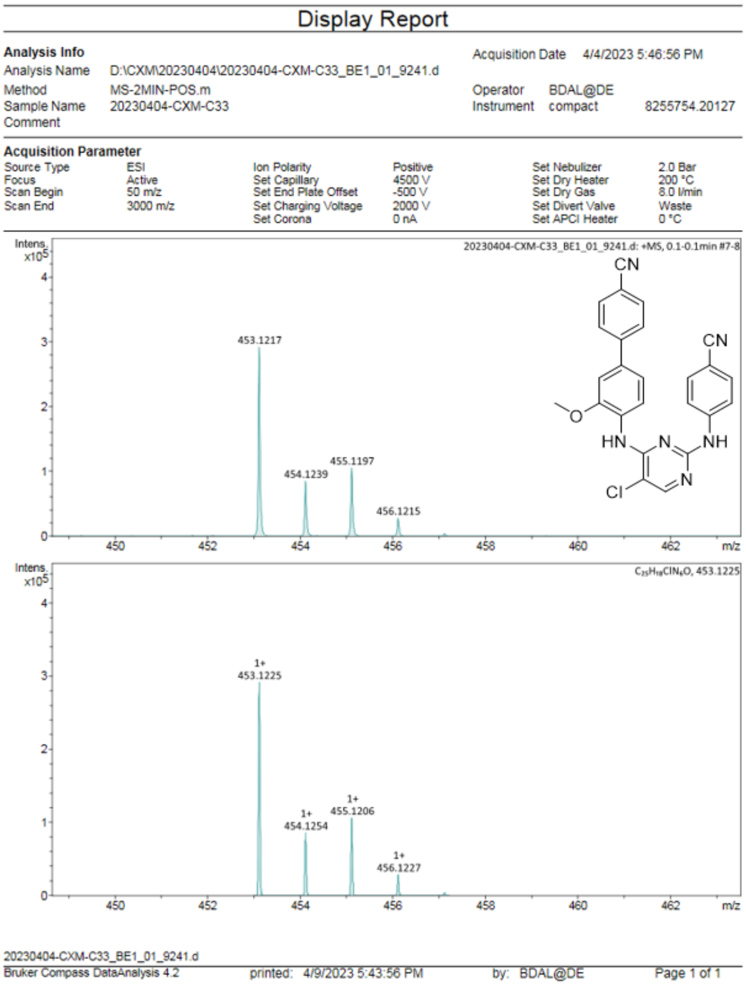

HPLC

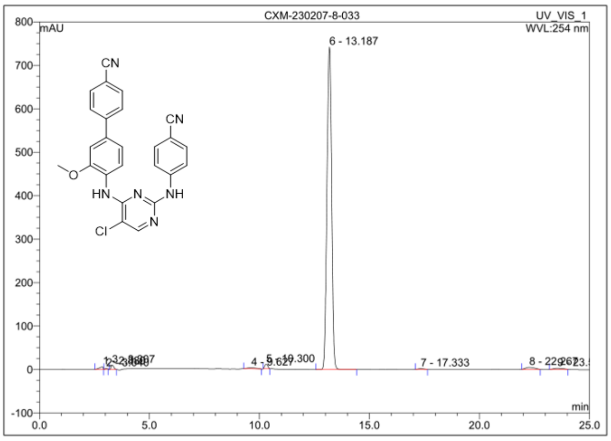

No.	Ret.Time min	Peak Name	Height mAU	Area mAU*s	Rel.Area %	Amount	Type
1	2.88	n.a.	5.379	66.17	0.57	n.a.	BMB*
2	3.04	n.a.	1.737	10.04	0.09	n.a.	BMB*
3	3.31	n.a.	10.201	88.31	0.76	n.a.	BMB*
4	9.63	n.a.	2.287	69.14	0.60	n.a.	BMB*
5	10.30	n.a.	9.978	101.50	0.87	n.a.	BMB*
6	13.19	n.a.	741.915	11037.31	95.04	n.a.	BMB*
7	17.33	n.a.	1.724	32.64	0.28	n.a.	BMB*
8	22.27	n.a.	4.597	132.63	1.14	n.a.	BMB*
9	23.54	n.a.	2.578	75.49	0.65	n.a.	BMB*
Total:			780.396	11613.229	100.00	0.000	

^{1}H NMR, ^{13}C NMR, ^{19}F NMR, HRMS, HPLC spectra of B10

^{1}H NMR

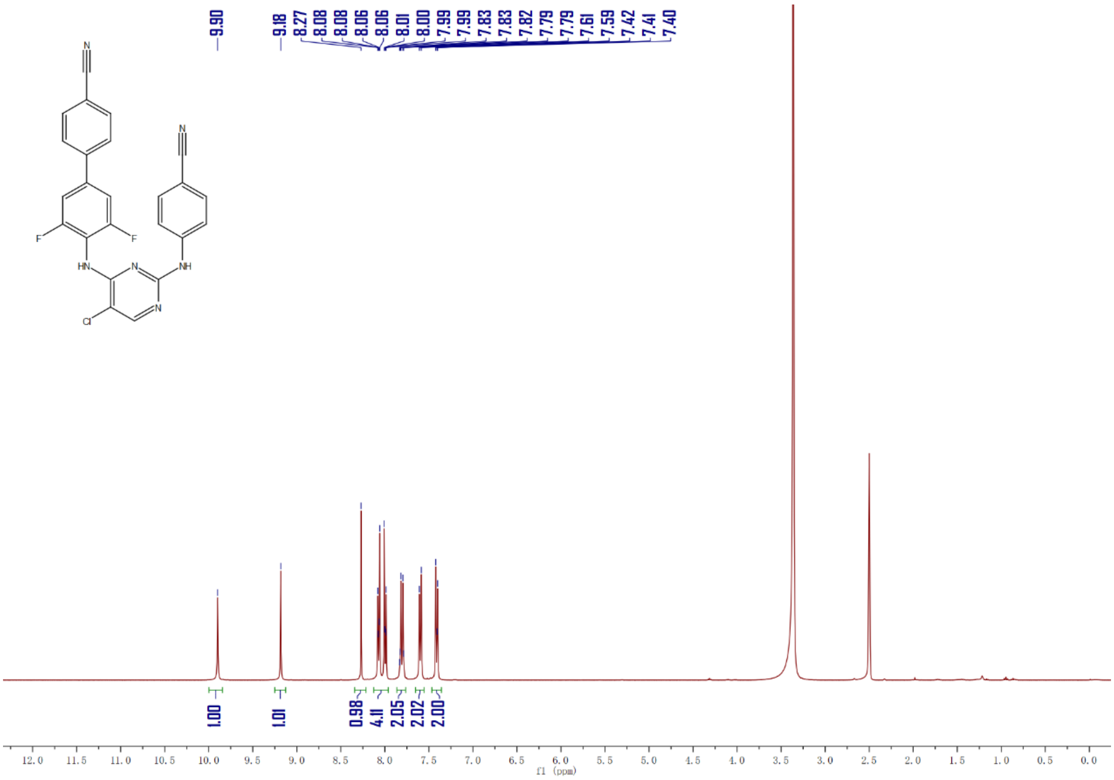

^{13}C NMR

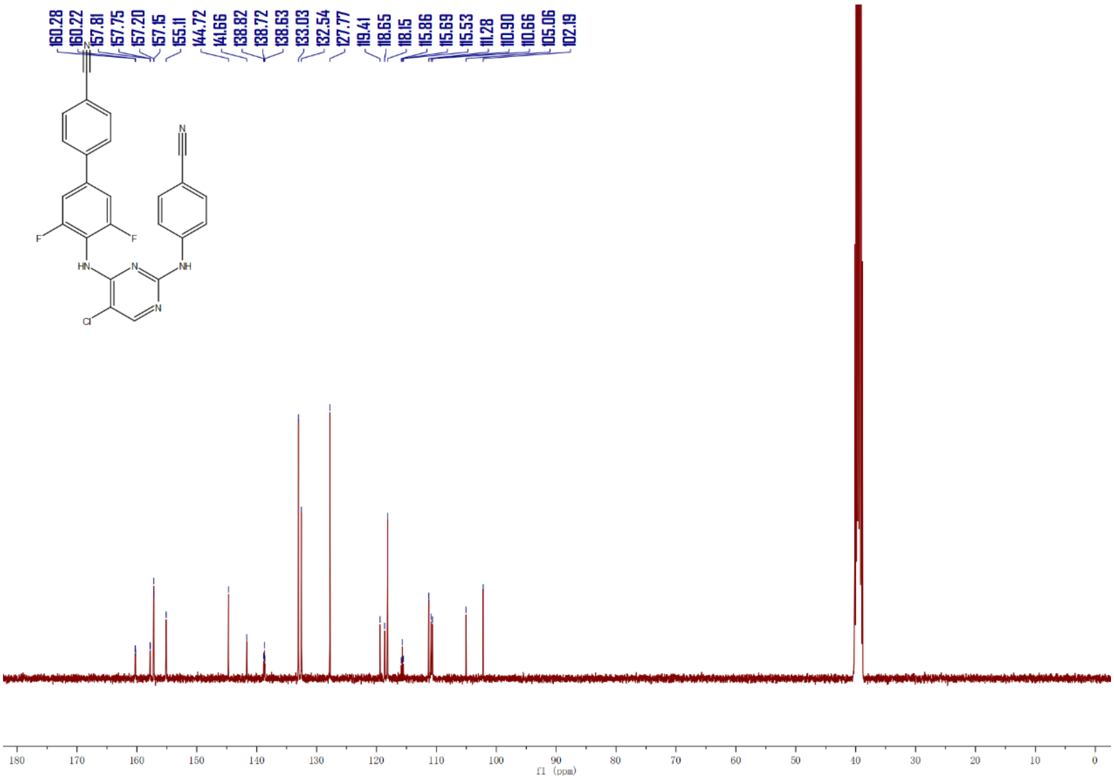

^{19}F NMR

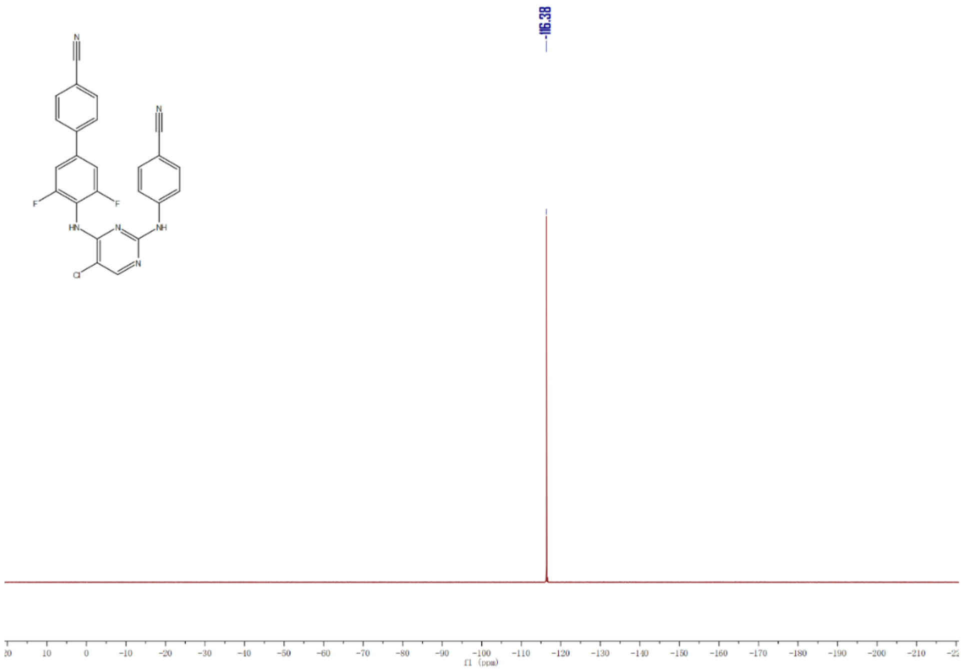

-116.38
f1 (ppm)

HRMS

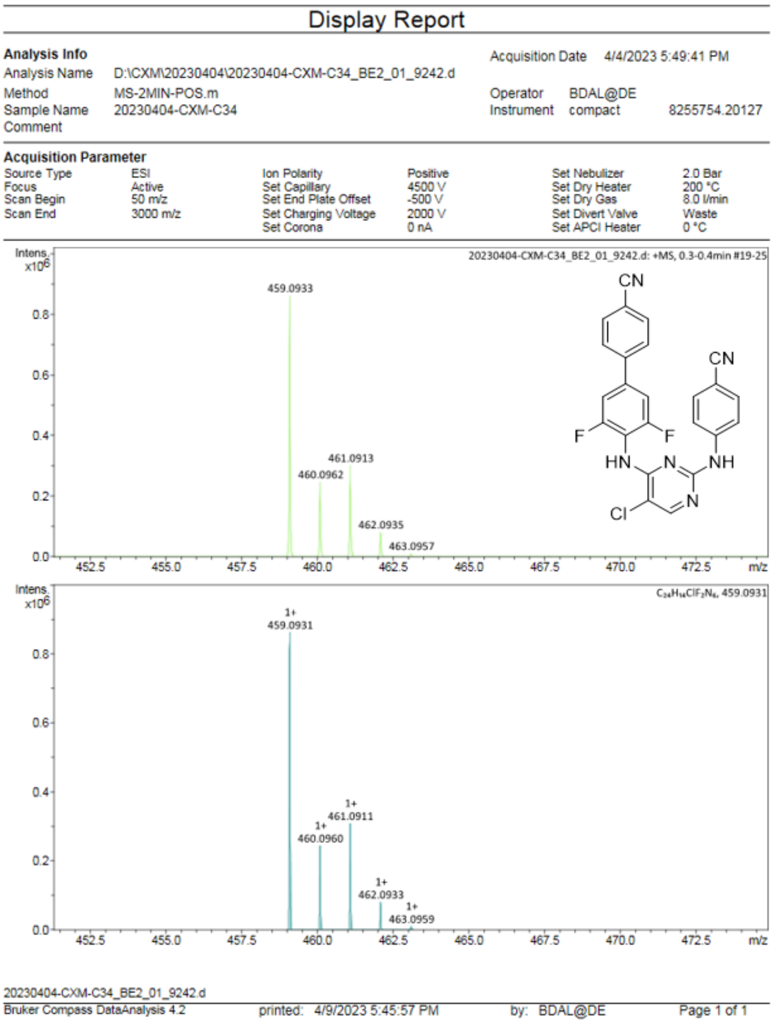

Display Report
Analysis Info
Analysis Name D:\CXM\20230404\20230404-CXM-C34_BE2_01_9242.d
Method MS-2MIN-POS.m
Sample Name 20230404-CXM-C34
Comment
Acquisition Date 4/4/2023 5:49:41 PM
Operator BDAL@DE
Instrument compact 8255754.20127
Acquisition Parameter
Source Type ESI
Focus Active
Scan Begin 50 m/z
Scan End 3000 m/z
Ion Polarity Positive
Set Capillary 4500 V
Set End Plate Offset -500 V
Set Charging Voltage 2000 V
Set Corona 0 nA
Set Nebulizer 2.0 Bar
Set Dry Heater 200 °C
Set Dry Gas 8.0 l/min
Set Divert Valve Waste
Set APCI Heater 0 °C
20230404-CXM-C34_BE2_01_9242.d: +MS, 0.3-0.4min #19-25
459.0933
460.0962
461.0913
462.0935
463.0957
$C_{24}H_{14}ClF_2N_6$, 459.0931
459.0931
460.0960
461.0911
462.0933
463.0959
20230404-CXM-C34_BE2_01_9242.d
Bruker Compass DataAnalysis 4.2 printed: 4/9/2023 5:45:57 PM by: BDAL@DE Page 1 of 1

HPLC

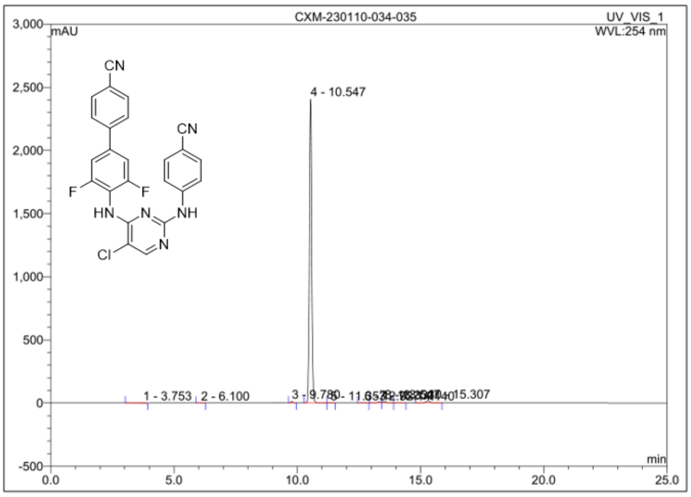

No.	Ret.Time min	Peak Name	Height mAU	Area mAU*s	Rel.Area %	Amount	Type
1	3.75	n.a.	1.972	23.62	0.15	n.a.	BMB
2	6.10	n.a.	0.782	5.74	0.04	n.a.	BMB
3	9.78	n.a.	11.692	66.36	0.42	n.a.	BMB
4	10.55	n.a.	2407.635	15313.30	96.57	n.a.	BM
5	11.35	n.a.	1.320	10.86	0.07	n.a.	MB
6	12.73	n.a.	1.074	10.02	0.06	n.a.	BMB
7	13.33	n.a.	8.365	92.76	0.58	n.a.	BM
8	13.55	n.a.	10.215	117.92	0.74	n.a.	Mb
9	14.14	n.a.	0.547	6.84	0.04	n.a.	bMB
10	15.31	n.a.	14.635	210.02	1.32	n.a.	BMB
Total:			2458.237	15857.452	100.00	0.000	

^{1}H NMR, ^{13}C NMR, HRMS, HPLC spectra of B11

^{1}H NMR

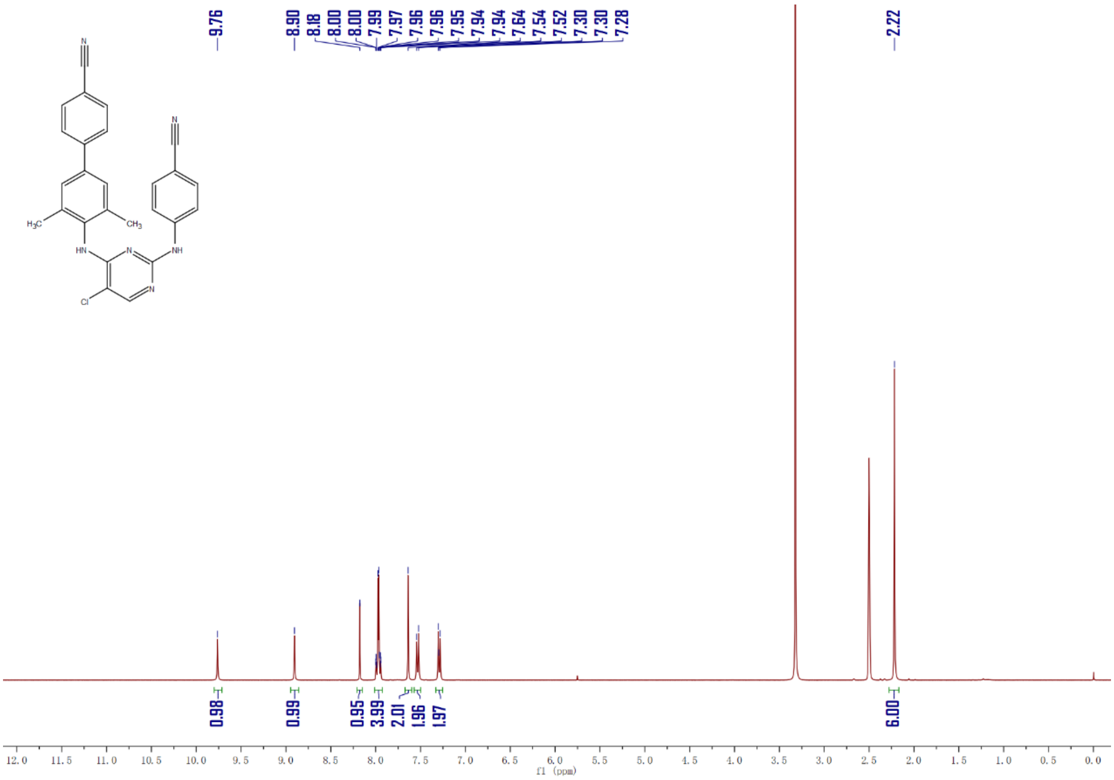

^{13}C NMR

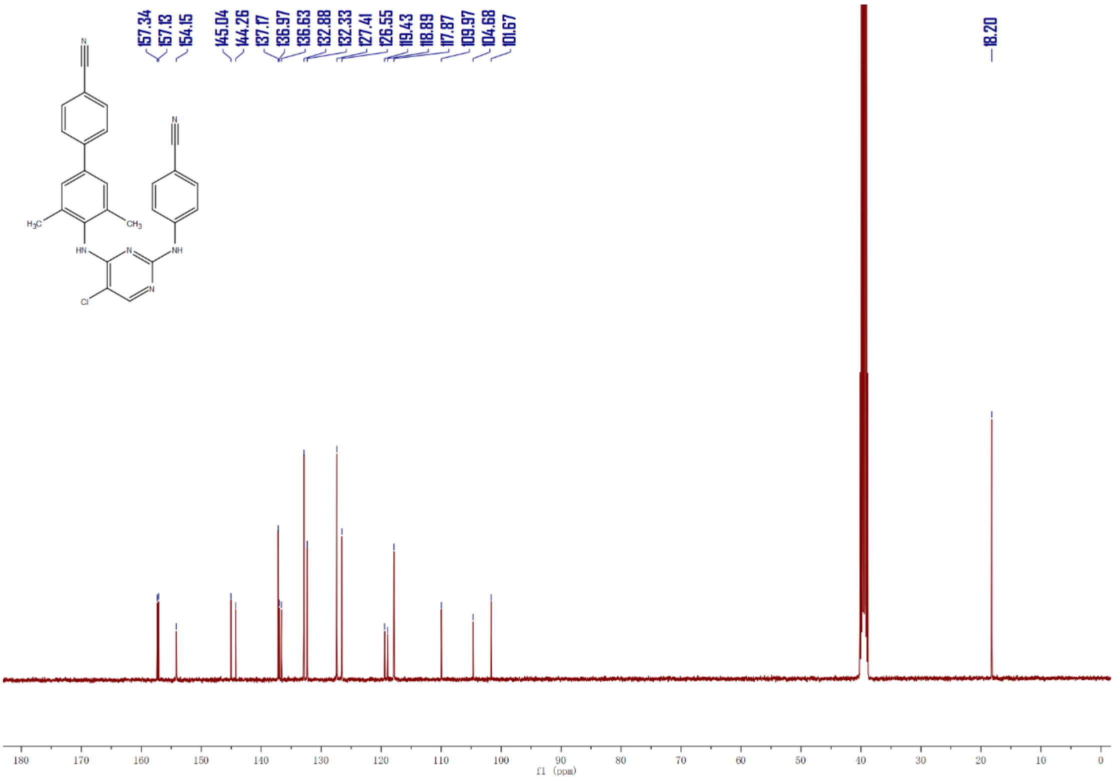

157.34
157.13
154.15
145.04
144.26
137.17
136.97
136.63
132.88
132.33
127.41
126.55
119.43
118.89
117.87
109.97
104.68
101.67
18.20
N
H3C
CH3
HN
NH
Cl
180 170 160 150 140 130 120 110 100 90 80 70 60 50 40 30 20 10 0
f1 (ppm)

HRMS

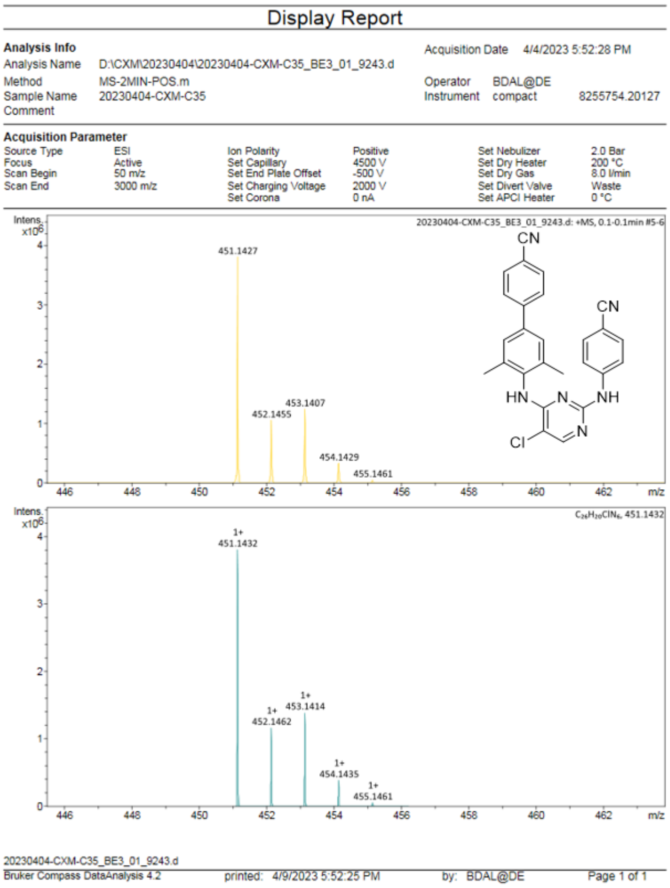

Display Report
Analysis Info
Analysis Name D:\CXM\20230404\20230404-CXM-C35_BE3_01_9243.d
Method MS-2MIN-POS.m
Sample Name 20230404-CXM-C35
Comment
Acquisition Date 4/4/2023 5:52:28 PM
Operator BDAL@DE
Instrument compact 8255754.20127
Acquisition Parameter
Source Type ESI
Focus Active
Scan Begin 50 m/z
Scan End 3000 m/z
Ion Polarity Positive
Set Capillary 4500 V
Set End Plate Offset -500 V
Set Charging Voltage 2000 V
Set Corona 0 nA
Set Nebulizer 2.0 Bar
Set Dry Heater 200 °C
Set Dry Gas 8.0 l/min
Set Divert Valve Waste
Set APCI Heater 0 °C
Intens. x10^6
20230404-CXM-C35_BE3_01_9243.d: +MS, 0.1-0.1min #5-6
451.1427
452.1455
453.1407
454.1429
455.1461
CN
CN
HN
N
NH
Cl
N
446 448 450 452 454 456 458 460 462 m/z
Intens. x10^6
C26H20ClN6, 451.1432
1+ 451.1432
1+ 452.1462
1+ 453.1414
1+ 454.1435
1+ 455.1461
446 448 450 452 454 456 458 460 462 m/z
20230404-CXM-C35_BE3_01_9243.d
Bruker Compass DataAnalysis 4.2
printed: 4/9/2023 5:52:25 PM
by: BDAL@DE
Page 1 of 1

HPLC

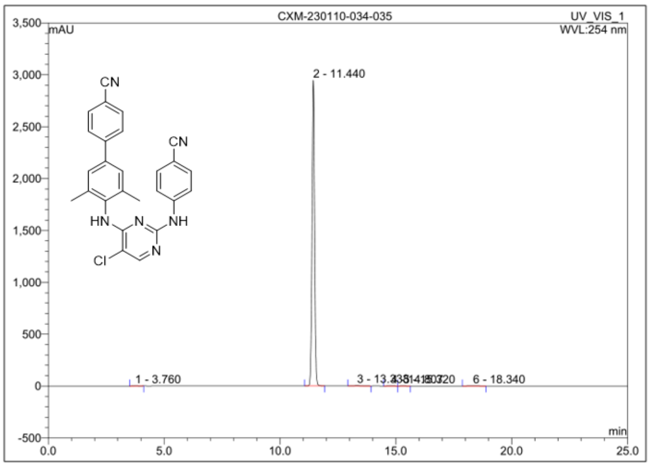

No.	Ret.Time min	Peak Name	Height mAU	Area mAU*s	Rel.Area %	Amount	Type
1	3.76	n.a.	1.671	13.37	0.06	n.a.	BMB*
2	11.44	n.a.	2946.866	22354.45	99.50	n.a.	BMB
3	13.33	n.a.	3.843	51.60	0.23	n.a.	BMB
4	14.81	n.a.	0.883	12.08	0.05	n.a.	BMB
5	15.32	n.a.	0.350	4.74	0.02	n.a.	BMB
6	18.34	n.a.	1.493	30.60	0.14	n.a.	BMB
Total:			2955.107	22466.845	100.00	0.000	

^{1}H NMR, ^{13}C NMR, ^{19}F NMR, HRMS, HPLC spectra of B12

^{1}H NMR

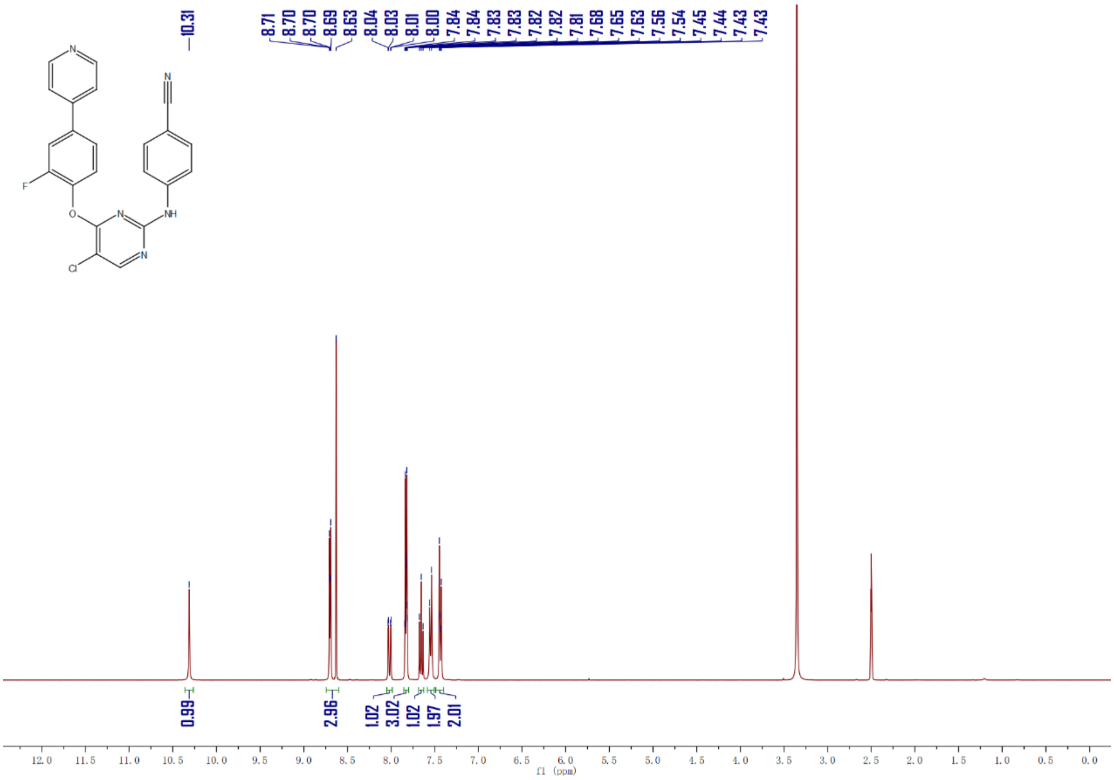

^{13}C NMR

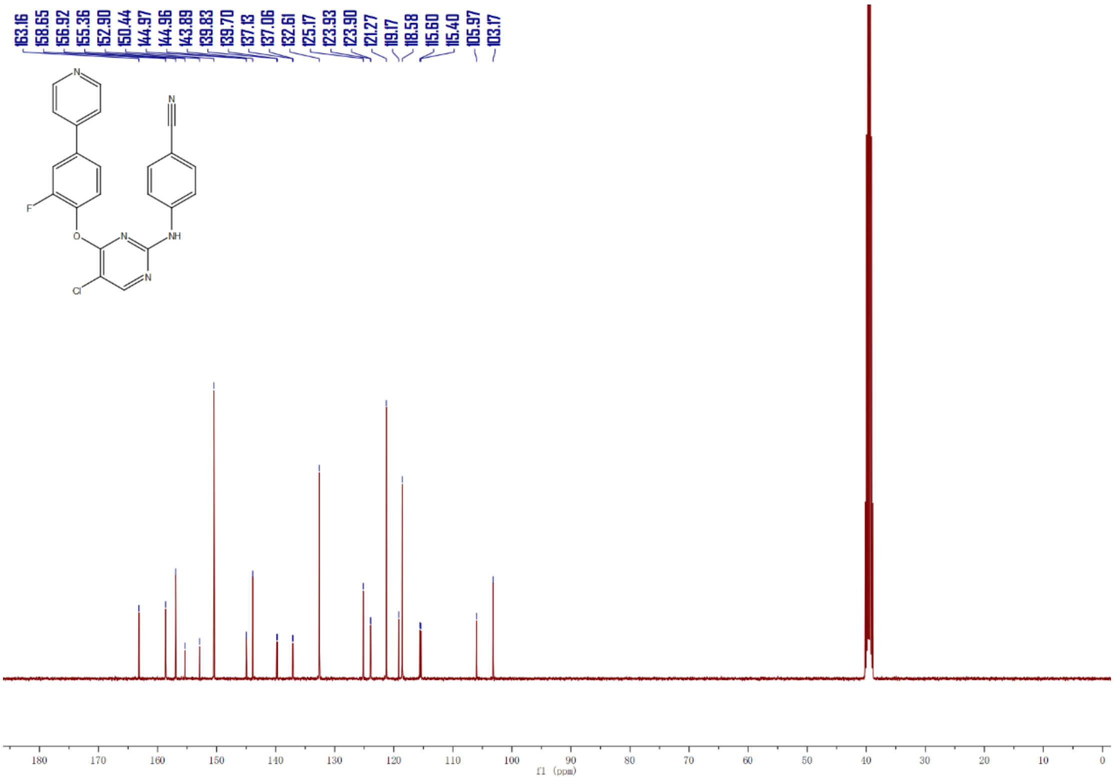

^{19}F NMR

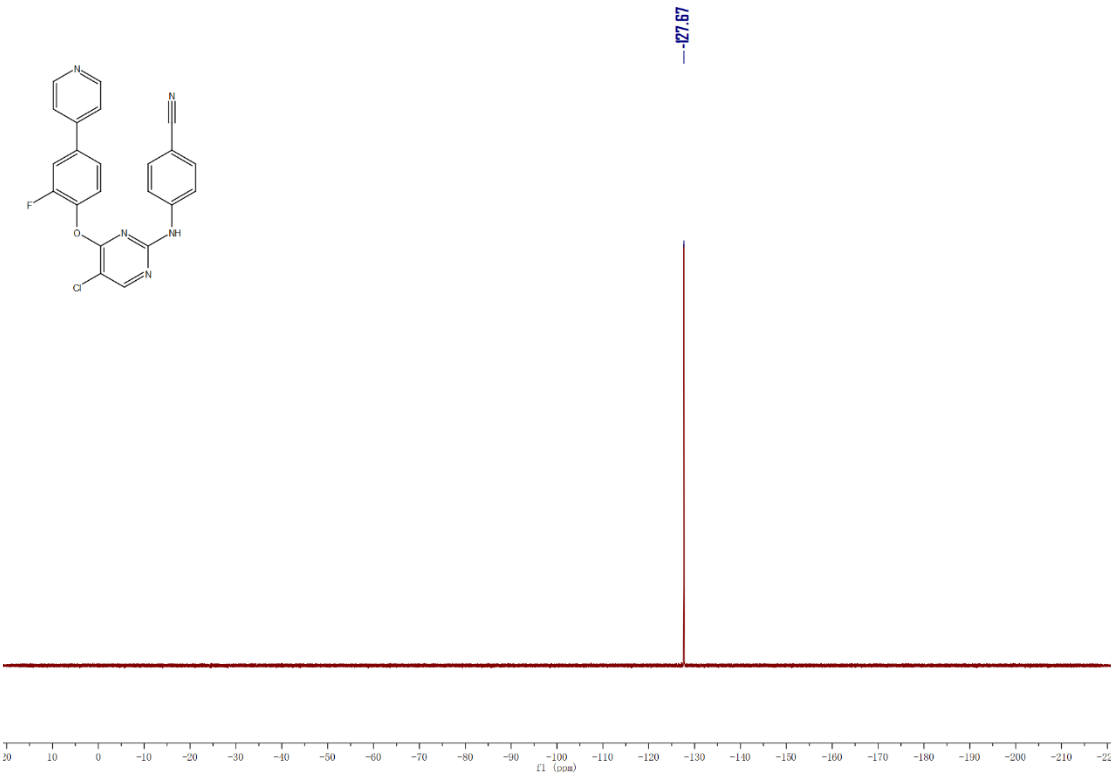

HRMS

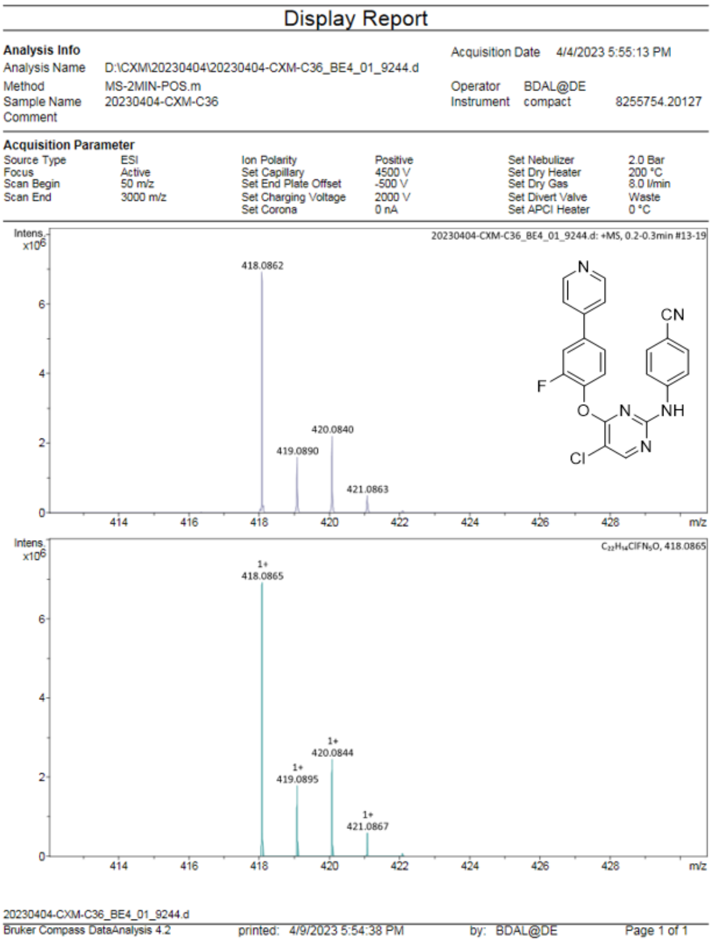

HPLC

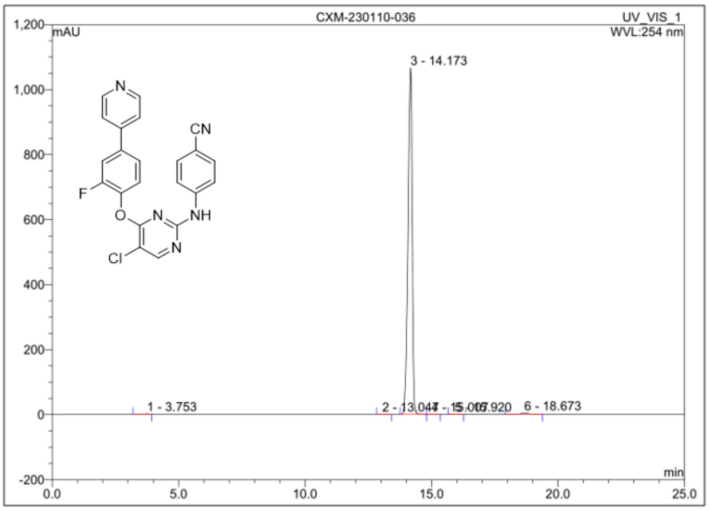

No.	Ret.Time min	Peak Name	Height mAU	Area mAU*s	Rel.Area %	Amount	Type
1	3.75	n.a.	1.997	22.70	0.18	n.a.	BMB
2	13.05	n.a.	0.295	4.23	0.03	n.a.	BMB
3	14.17	n.a.	1065.346	12338.99	99.12	n.a.	BM
4	15.01	n.a.	0.559	9.07	0.07	n.a.	MB
5	15.92	n.a.	0.173	3.05	0.02	n.a.	BMB
6	18.67	n.a.	3.391	70.01	0.56	n.a.	BMB
Total:			1071.761	12448.058	100.00	0.000	

^{1}H NMR, ^{13}C NMR, HRMS, HPLC spectra of B13

^{1}H NMR

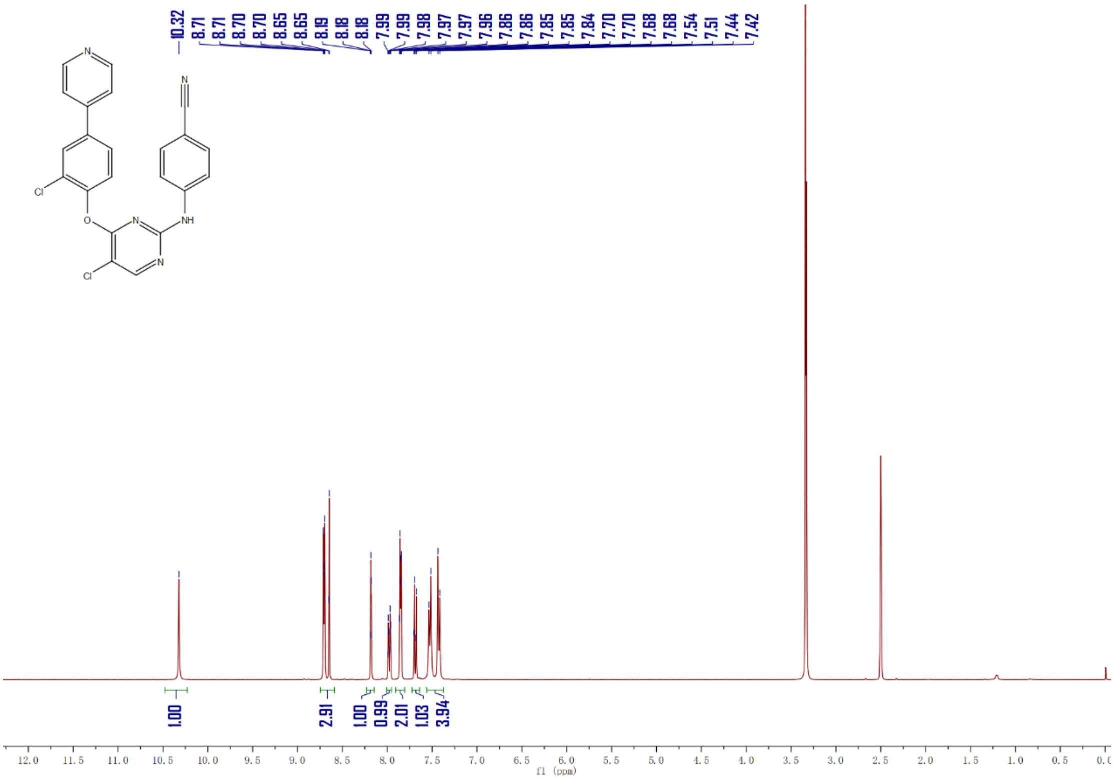

^{13}C NMR

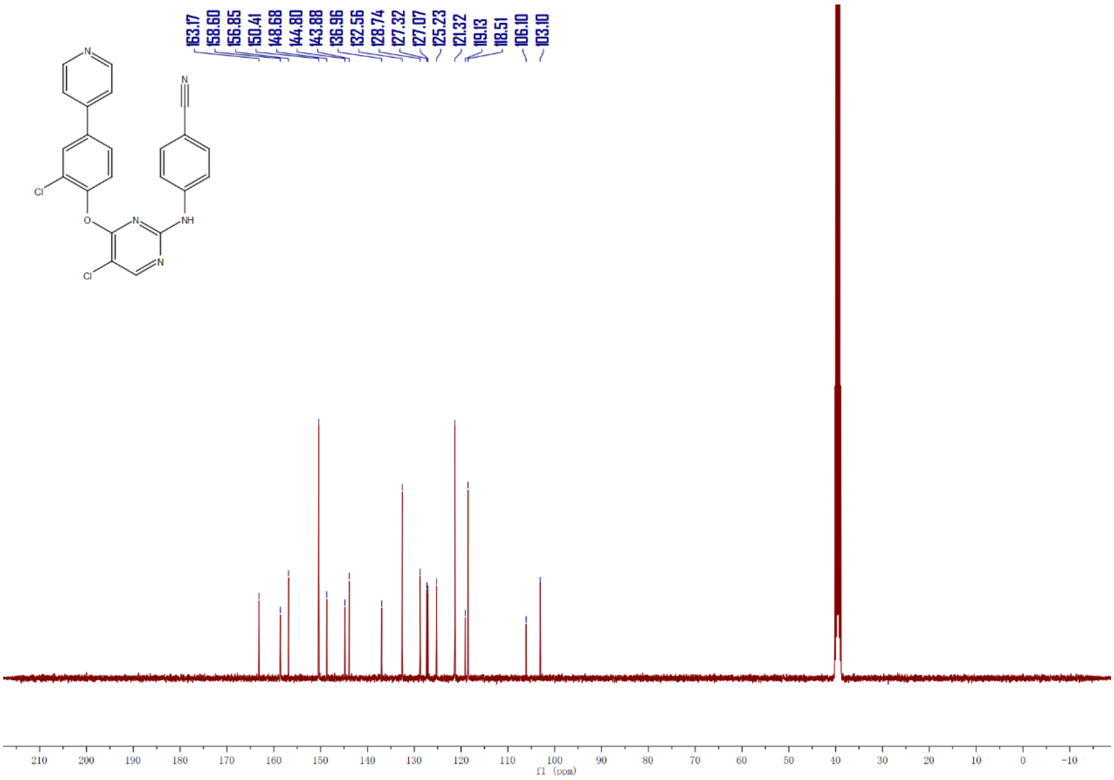

HRMS

Display Report

Analysis Info

Analysis Name	D:\CXM\20230404\20230404-CXM-C37_BE5_01_9245.d	Acquisition Date	4/4/2023 5:57:59 PM
Method	MS-2MIN-POS.m	Operator	BDAL@DE
Sample Name	20230404-CXM-C37	Instrument	compact 8255754.20127
Comment			

Acquisition Parameter

Source Type	ESI	Ion Polarity	Positive	Set Nebulizer	2.0 Bar
Focus	Active	Set Capillary	4500 V	Set Dry Heater	200 °C
Scan Begin	50 m/z	Set End Plate Offset	-500 V	Set Dry Gas	8.0 l/min
Scan End	3000 m/z	Set Charging Voltage	2000 V	Set Divert Valve	Waste
		Set Corona	0 nA	Set APCI Heater	0 °C

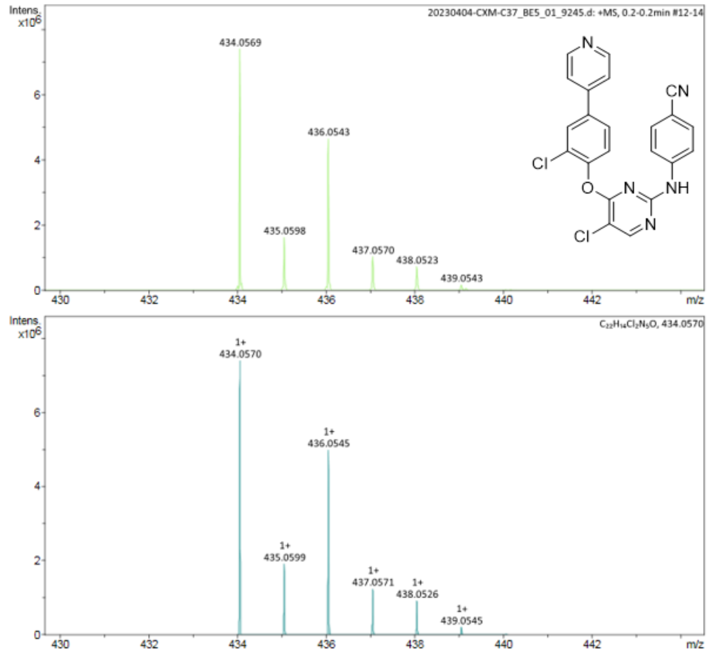

20230404-CXM-C37_BE5_01_9245.d

Bruker Compass DataAnalysis 4.2 printed: 4/9/2023 8:23:28 PM by: BDAL@DE Page 1 of 1

HPLC

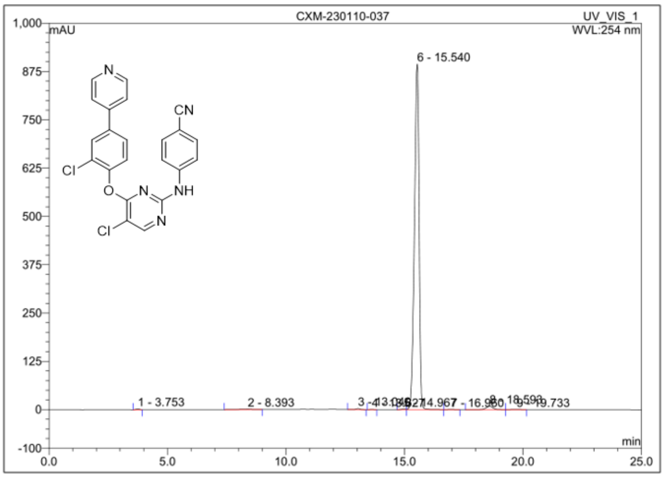

No.	Ret.Time min	Peak Name	Height mAU	Area mAU*s	Rel.Area %	Amount	Type
1	3.75	n.a.	2.077	21.62	0.17	n.a.	BMB
2	8.39	n.a.	0.952	24.79	0.20	n.a.	BMB
3	13.04	n.a.	2.379	27.35	0.22	n.a.	BMB
4	13.63	n.a.	0.408	4.17	0.03	n.a.	BMB
5	14.97	n.a.	1.455	17.07	0.14	n.a.	BM
6	15.54	n.a.	894.458	12292.25	97.73	n.a.	MB
7	16.96	n.a.	0.378	6.43	0.05	n.a.	BMB
8	18.59	n.a.	8.724	171.79	1.37	n.a.	BMB
9	19.73	n.a.	0.556	12.33	0.10	n.a.	bMB
Total:			911.387	12577.792	100.00	0.000	

^{1}H NMR, ^{13}C NMR, ^{19}F NMR, HRMS, HPLC spectra of B14

^{1}H NMR

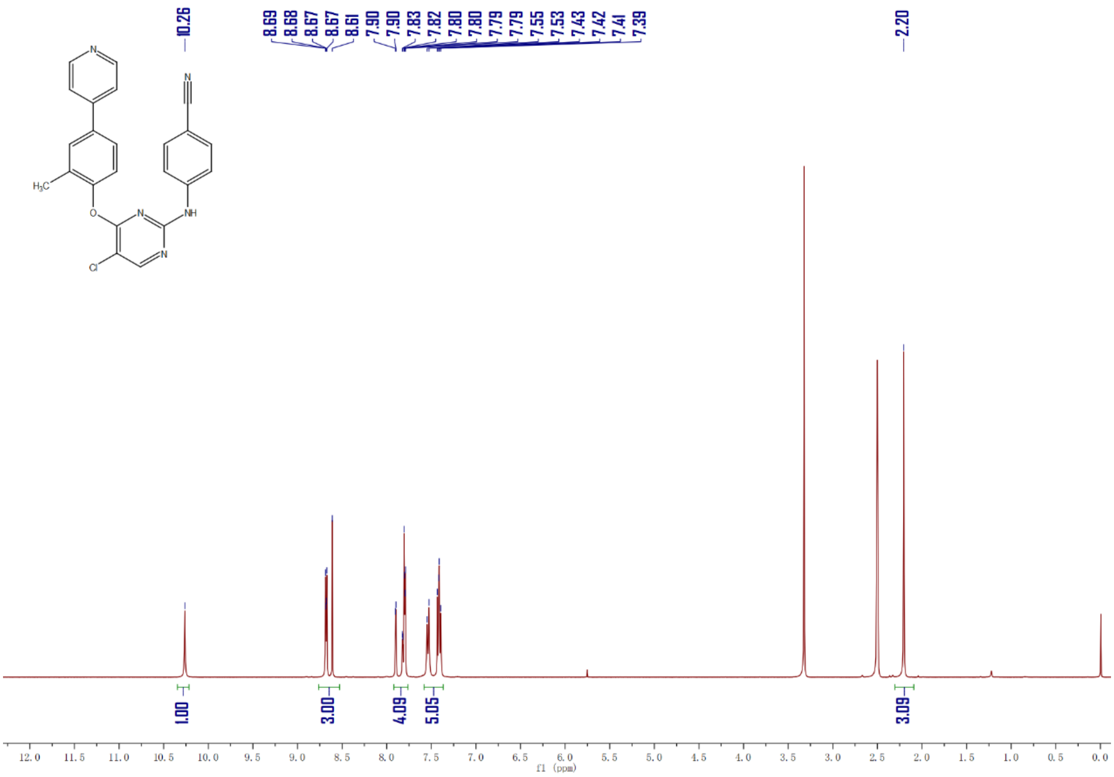

^{13}C NMR

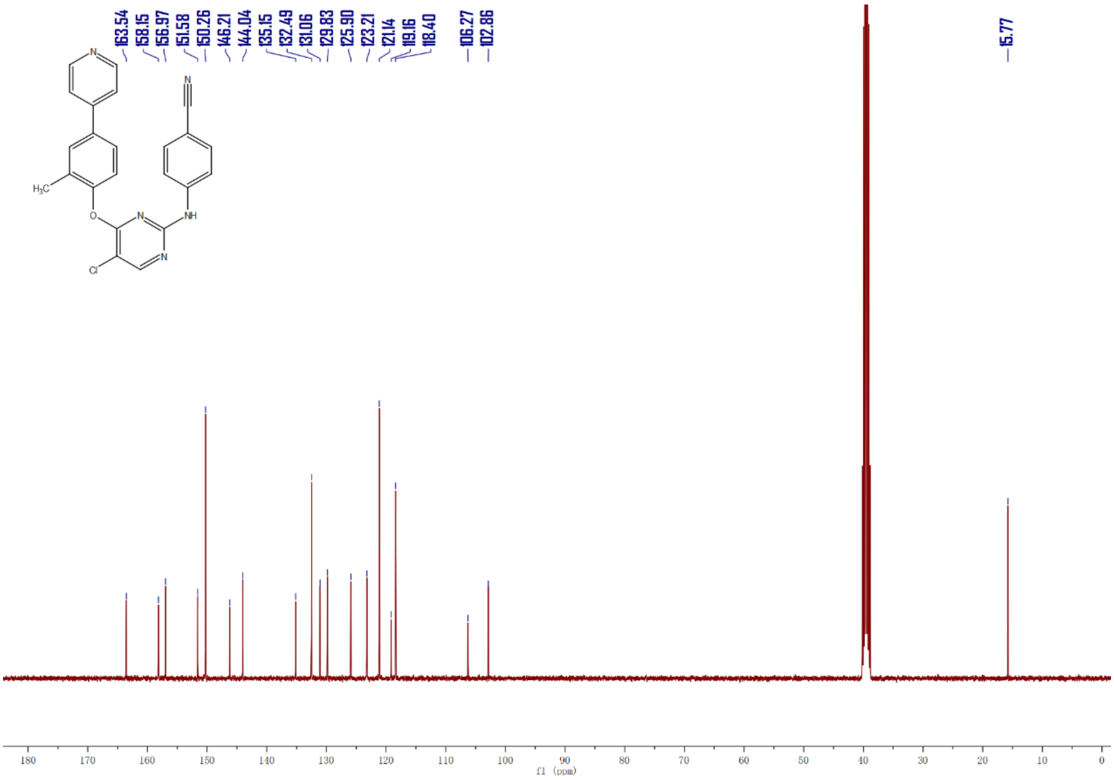

HRMS

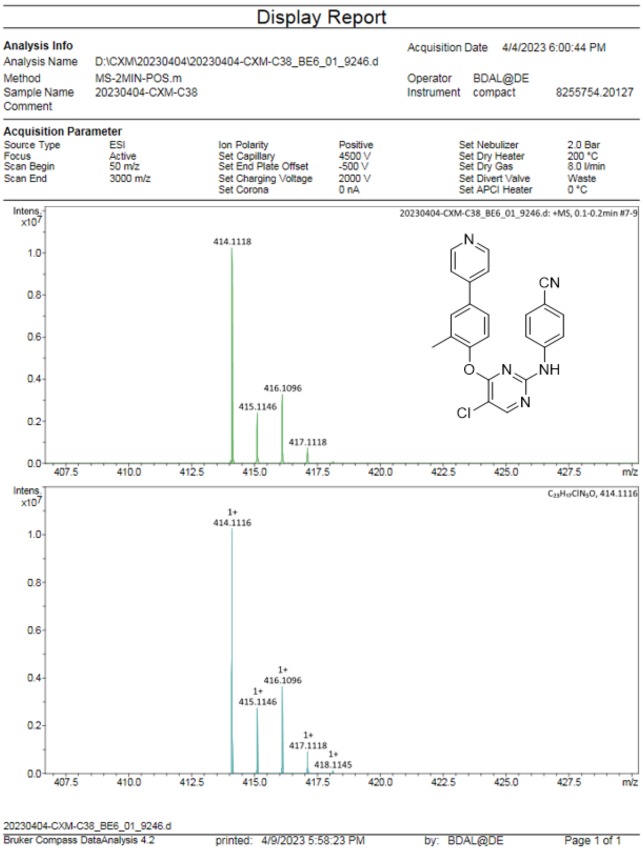

HPLC

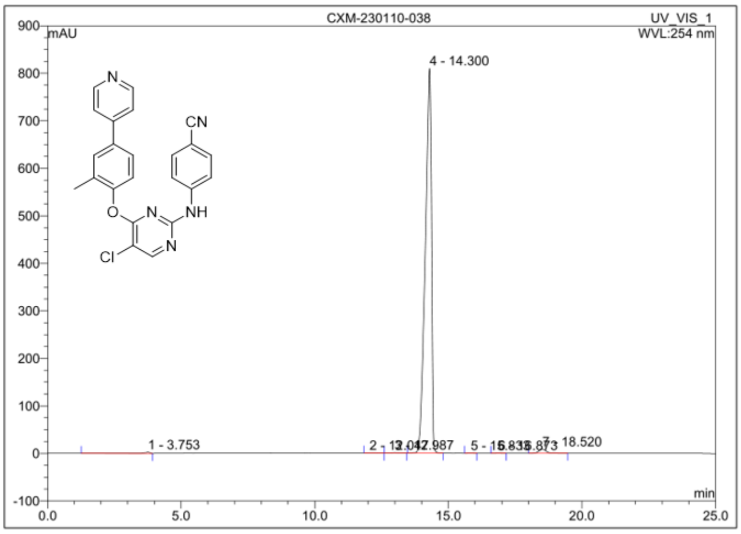

No.	Ret.Time min	Peak Name	Height mAU	Area mAU*s	Rel.Area %	Amount	Type
1	3.75	n.a.	2.773	72.09	0.54	n.a.	BMB
2	12.05	n.a.	0.276	6.05	0.05	n.a.	BMB
3	12.99	n.a.	0.902	14.22	0.11	n.a.	BMB
4	14.30	n.a.	809.754	12973.07	98.00	n.a.	BMB
5	15.83	n.a.	0.242	3.19	0.02	n.a.	BMB
6	16.87	n.a.	0.250	3.91	0.03	n.a.	BMB
7	18.52	n.a.	8.429	165.18	1.25	n.a.	BMB
Total:			822.626	13237.699	100.00	0.000	

^{1}H NMR, ^{13}C NMR, HRMS, HPLC spectra of B15

^{1}H NMR

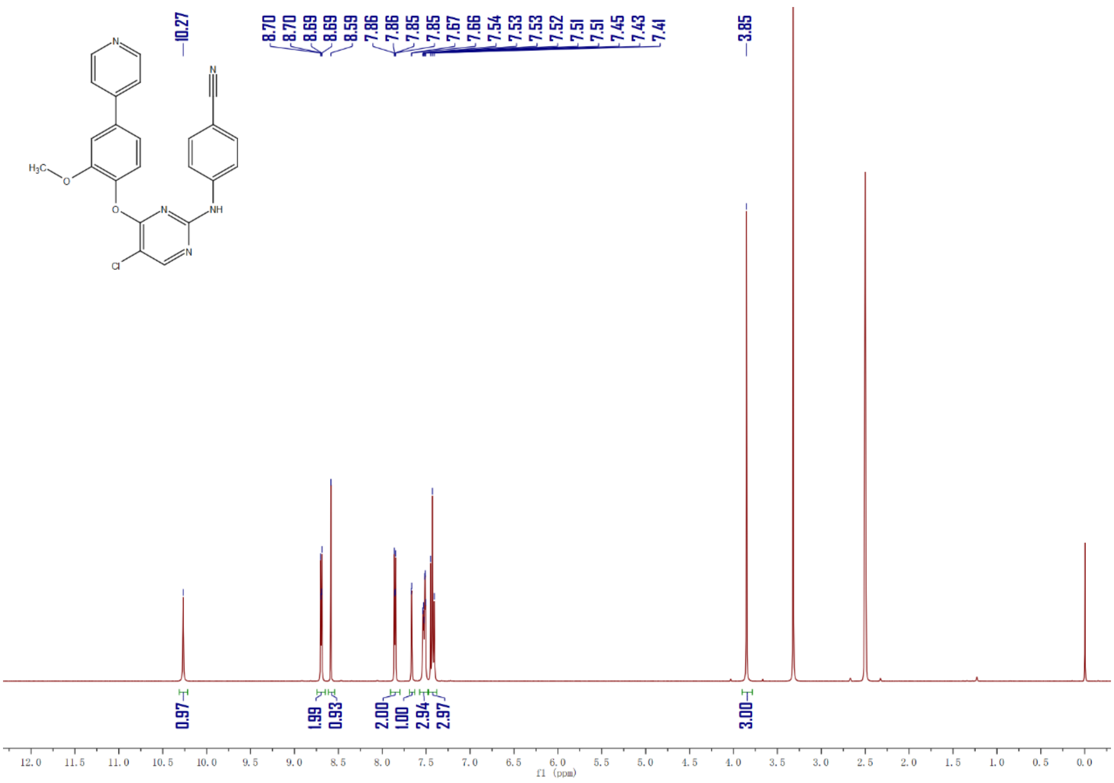

^{13}C NMR

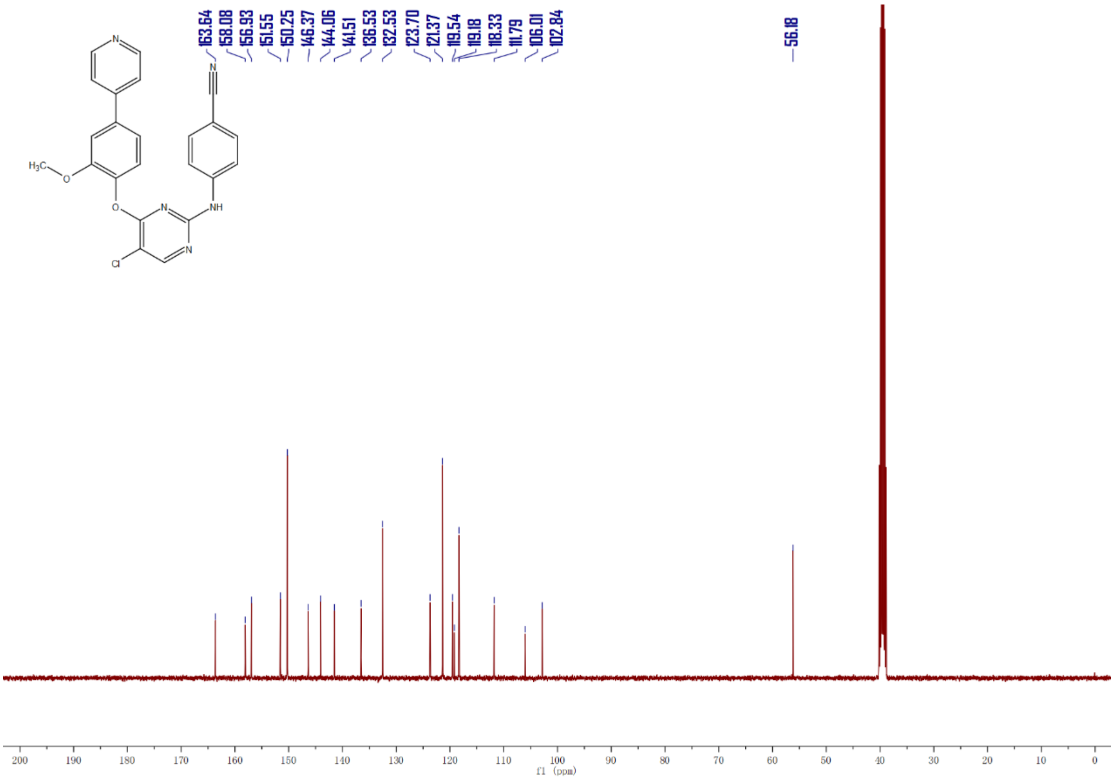

HRMS

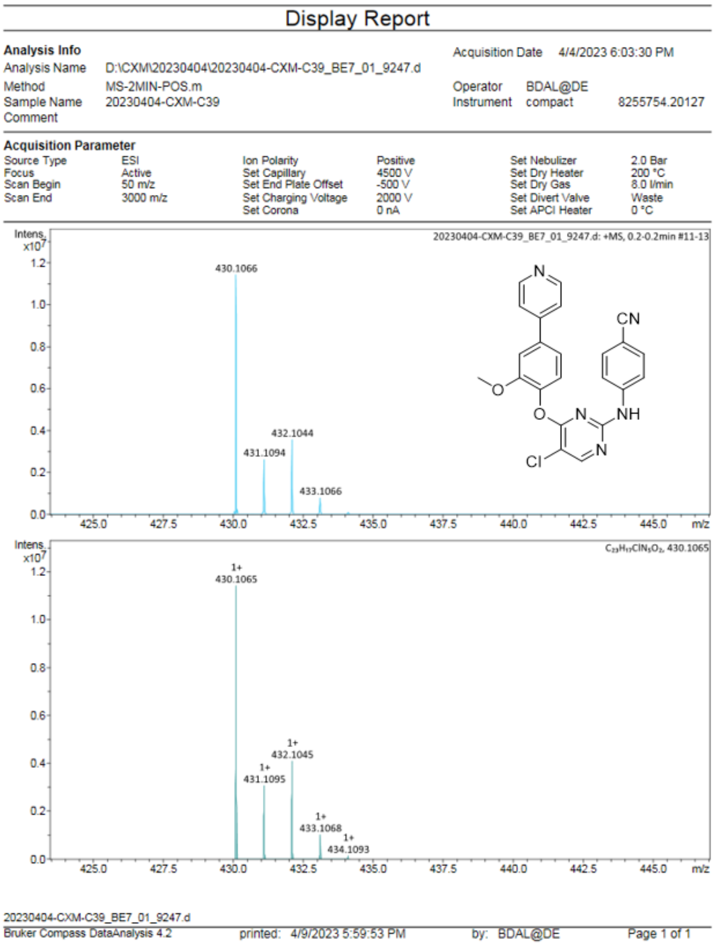

HPLC

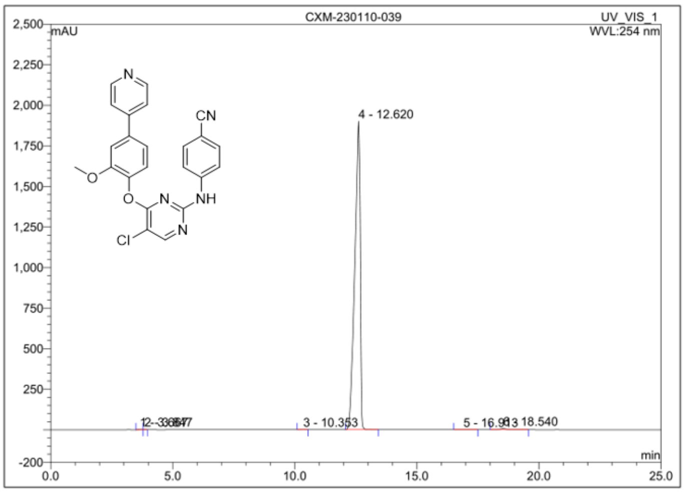

No.	Ret.Time min	Peak Name	Height mAU	Area mAU*s	Rel.Area %	Amount	Type
1	3.67	n.a.	2.810	31.04	0.11	n.a.	BM
2	3.85	n.a.	2.219	12.55	0.04	n.a.	MB
3	10.35	n.a.	1.390	12.07	0.04	n.a.	BMB
4	12.62	n.a.	1900.167	29196.34	99.31	n.a.	BMB
5	16.91	n.a.	0.293	7.43	0.03	n.a.	BMB
6	18.54	n.a.	6.993	139.36	0.47	n.a.	BMB
Total:			1913.873	29398.803	100.00	0.000	

^{1}H NMR, ^{13}C NMR, HRMS, HPLC spectra of B16

^{1}H NMR

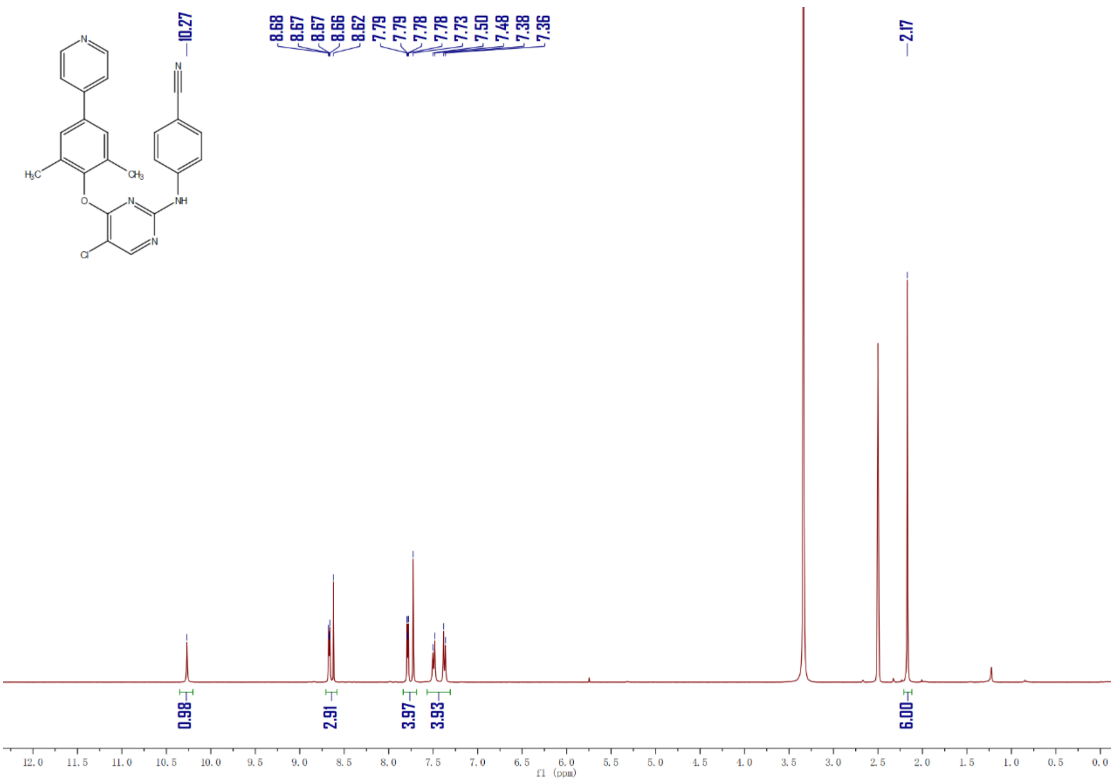

^{13}C NMR

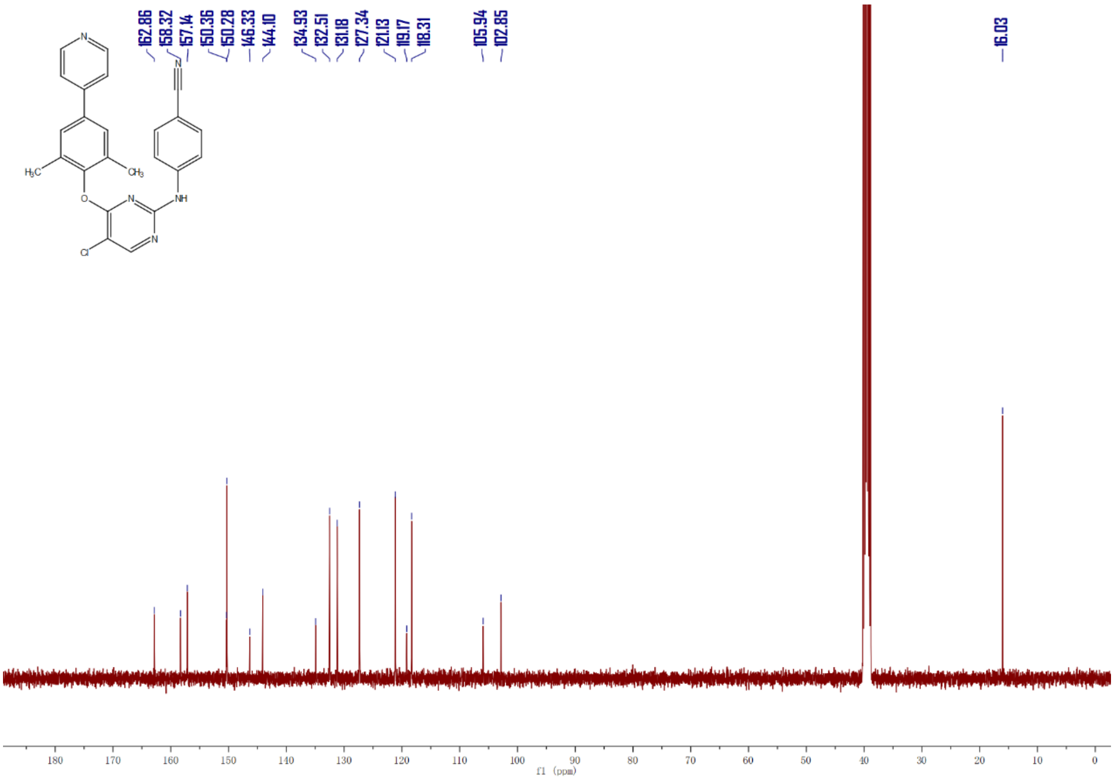

HRMS

Display Report

Analysis Info

Analysis Name	D:\CXM\20230404\20230404-CXM-C40_BE8_01_9248.d	Acquisition Date	4/4/2023 6:06:15 PM
Method	MS-2MIN-POS.m	Operator	BDAL@DE
Sample Name	20230404-CXM-C40	Instrument	compact 8255754.20127
Comment			

Acquisition Parameter

Source Type	ESI	Ion Polarity	Positive	Set Nebulizer	2.0 Bar
Focus	Active	Set Capillary	4500 V	Set Dry Heater	200 °C
Scan Begin	50 m/z	Set End Plate Offset	-500 V	Set Dry Gas	8.0 l/min
Scan End	3000 m/z	Set Charging Voltage	2000 V	Set Divert Valve	Waste
		Set Corona	0 nA	Set APCI Heater	0 °C

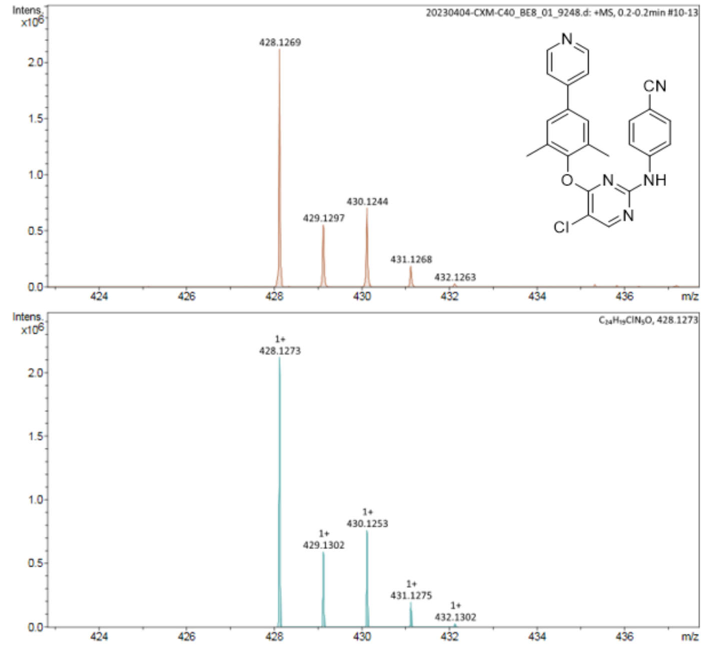

20230404-CXM-C40_BE8_01_9248.d
Bruker Compass DataAnalysis 4.2 printed: 4/9/2023 6:15:52 PM by: BDAL@DE Page 1 of 1

HPLC

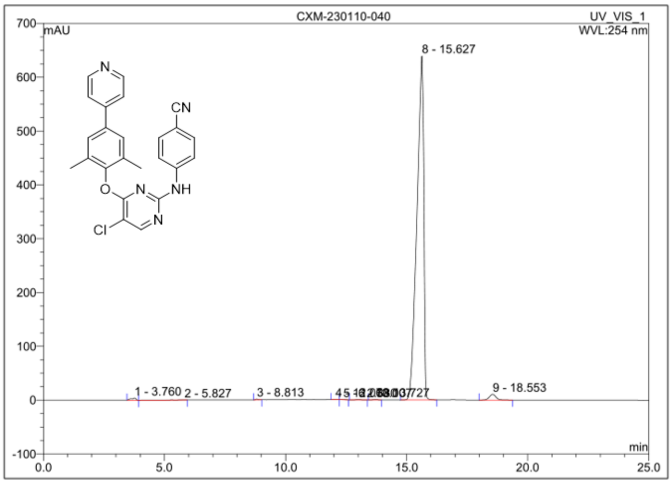

No.	Ret.Time min	Peak Name	Height mAU	Area mAU*s	Rel.Area %	Amount	Type
1	3.76	n.a.	4.236	60.76	0.47	n.a.	BMb*
2	5.83	n.a.	0.167	33.37	0.26	n.a.	bMB*
3	8.81	n.a.	1.460	8.40	0.07	n.a.	BMB
4	12.06	n.a.	0.503	3.95	0.03	n.a.	BMb
5	12.38	n.a.	0.328	3.81	0.03	n.a.	bMB
6	13.01	n.a.	0.861	10.93	0.08	n.a.	BMB
7	13.73	n.a.	1.119	13.57	0.11	n.a.	BMB
8	15.63	n.a.	639.367	12575.62	97.37	n.a.	BMB
9	18.55	n.a.	10.659	204.58	1.58	n.a.	BMB
Total:			658.701	12915.007	100.00	0.000	

^{1}H NMR, ^{13}C NMR, ^{19}F NMR, HRMS, HPLC spectra of B17

^{1}H NMR

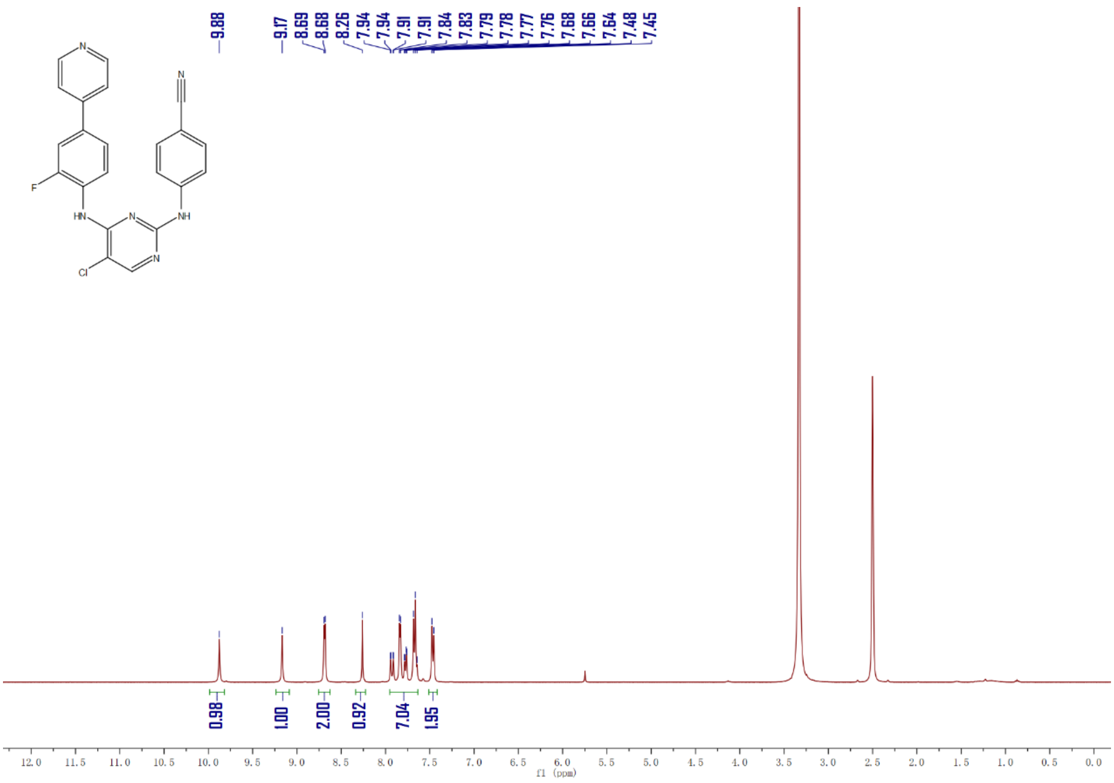

^{13}C NMR

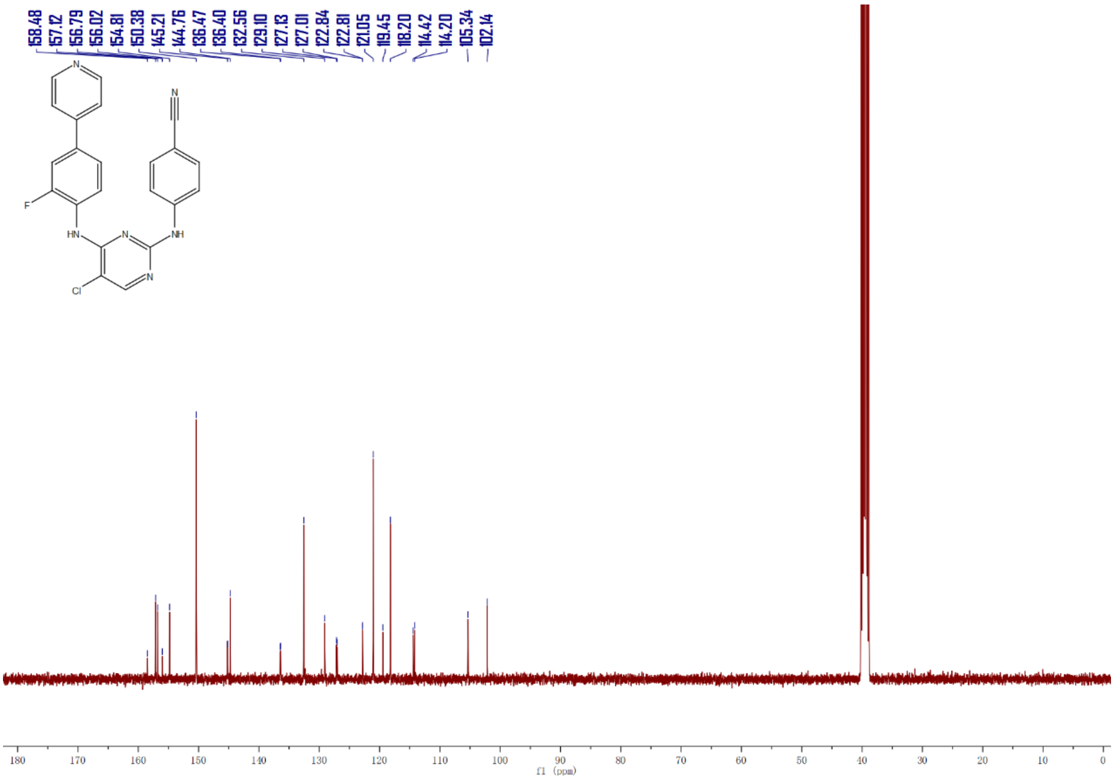

^{19}F NMR

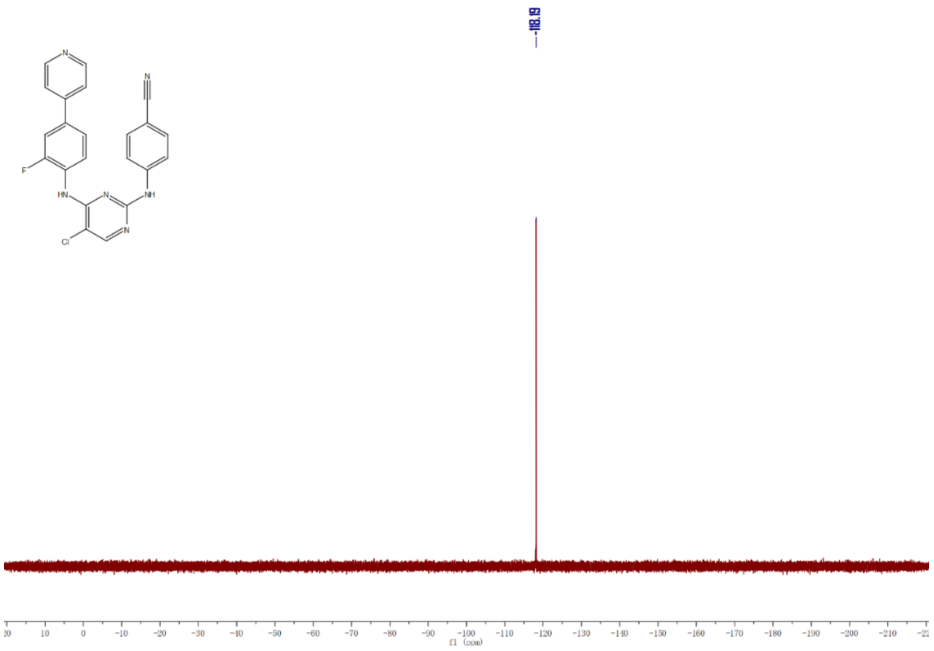

HRMS

Display Report

Analysis Info

Analysis Name	D:\CXM\20230404\20230404-CXM-C41_GA1_01_9249.d	Acquisition Date	4/4/2023 6:09:02 PM
Method	MS-2MIN-POS.m	Operator	BDAL@DE
Sample Name	20230404-CXM-C41	Instrument	compact 8255754.20127
Comment			

Acquisition Parameter

Source Type	ESI	Ion Polarity	Positive	Set Nebulizer	2.0 Bar
Focus	Active	Set Capillary	4500 V	Set Dry Heater	200 °C
Scan Begin	50 m/z	Set End Plate Offset	-500 V	Set Dry Gas	8.0 l/min
Scan End	3000 m/z	Set Charging Voltage	2000 V	Set Divert Valve	Waste
		Set Corona	0 nA	Set APCI Heater	0 °C

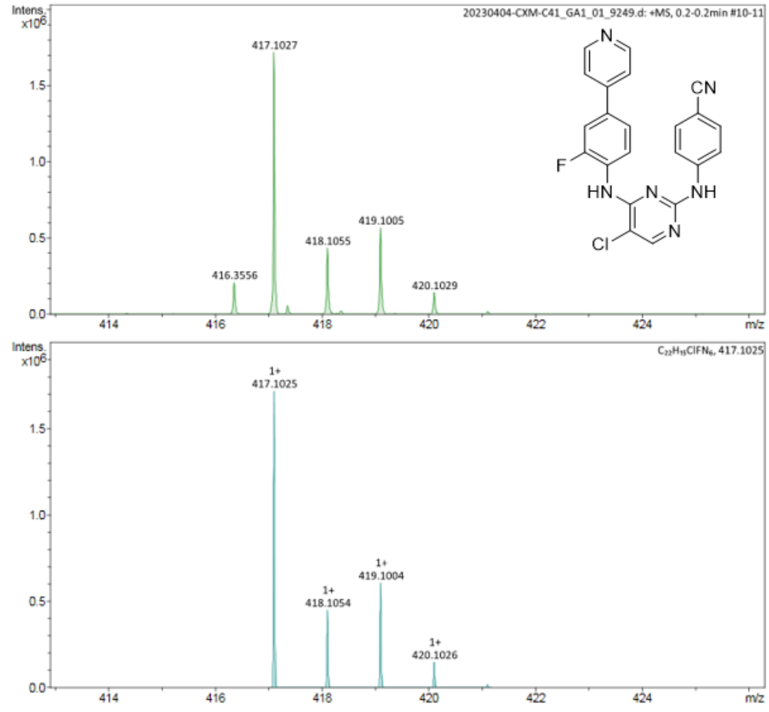

20230404-CXM-C41_GA1_01_9249.d
Bruker Compass DataAnalysis 4.2 printed: 4/9/2023 9:12:34 PM by: BDAL@DE Page 1 of 1

HPLC

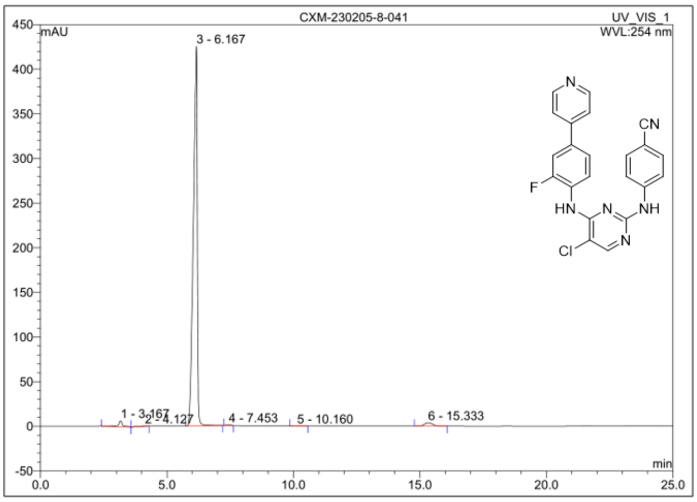

No.	Ret.Time min	Peak Name	Height mAU	Area mAU*s	Rel.Area %	Amount	Type
1	3.17	n.a.	6.852	89.77	1.86	n.a.	BMb*
2	4.13	n.a.	0.340	18.49	0.38	n.a.	bMB*
3	6.17	n.a.	425.432	4618.53	95.65	n.a.	BMB*
4	7.45	n.a.	0.745	8.32	0.17	n.a.	BMB*
5	10.16	n.a.	0.093	1.97	0.04	n.a.	BMB
6	15.33	n.a.	3.919	91.29	1.89	n.a.	BMB
Total:			437.381	4828.373	100.00	0.000	

^{1}H NMR, ^{13}C NMR, ^{19}F NMR, HRMS, HPLC spectra of B18

^{1}H NMR

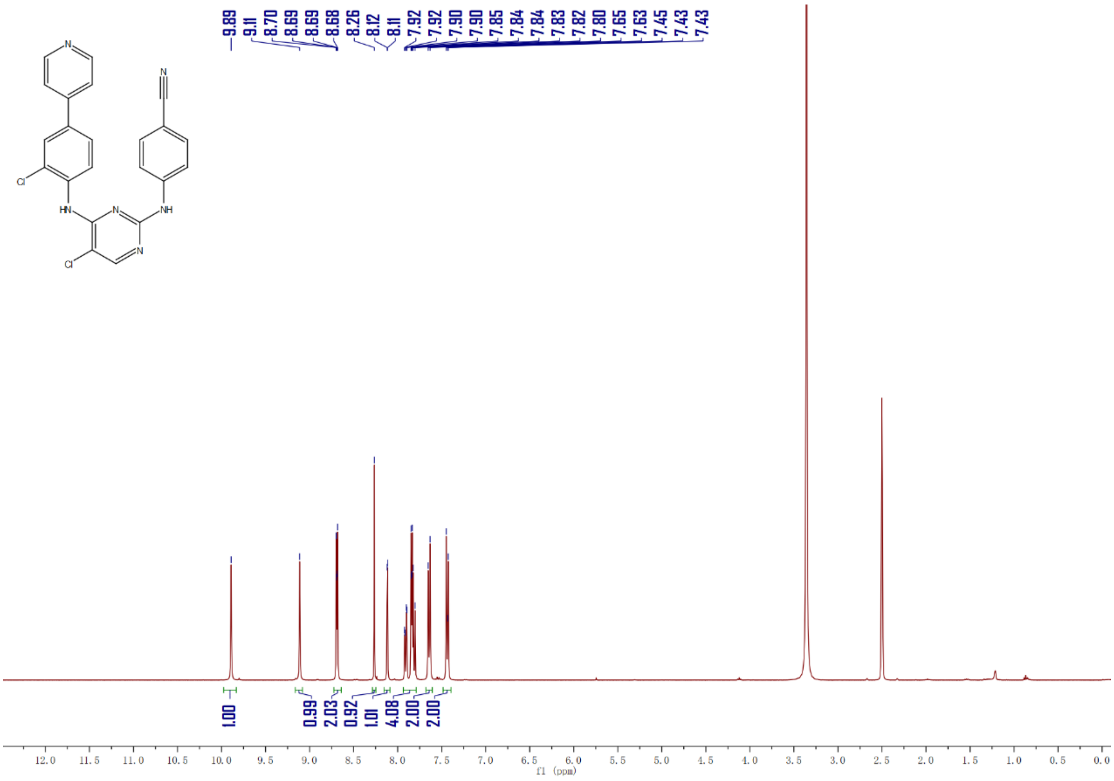

^{13}C NMR

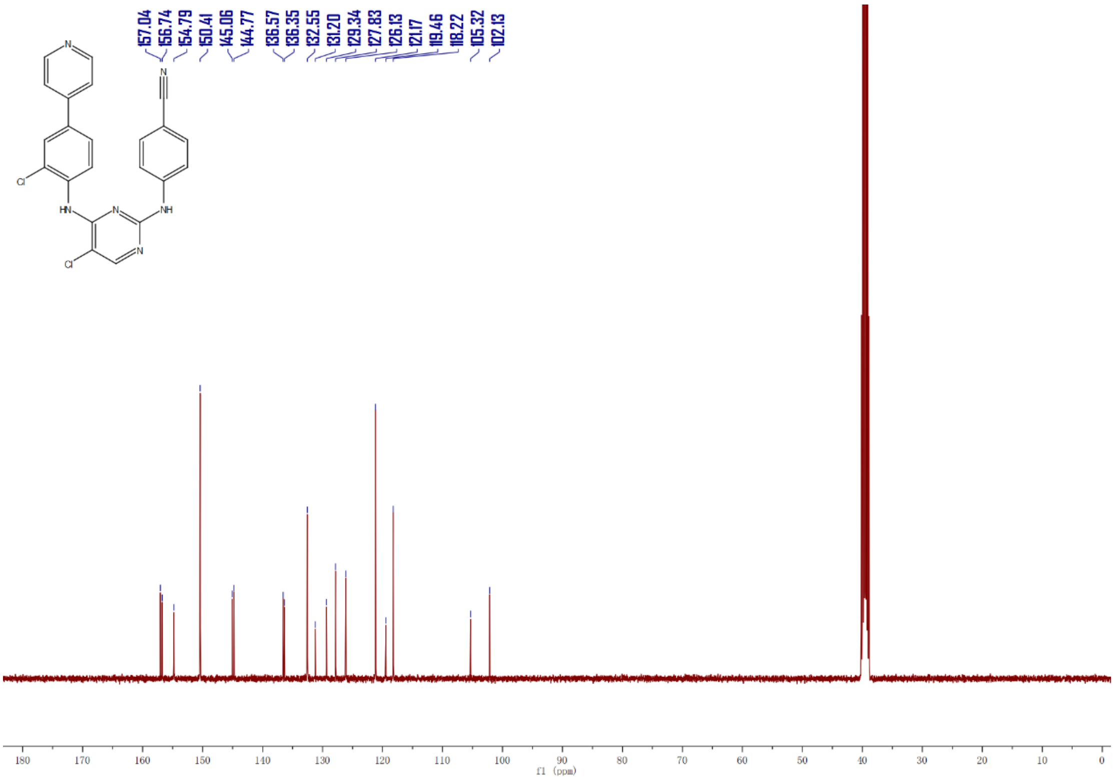

HRMS

Display Report

Analysis Info

Analysis Name	D:\CXM\20230404\20230404-CXM-C42_GA2_01_9250.d	Acquisition Date	4/4/2023 6:11:47 PM
Method	MS-2MIN-POS.m	Operator	BDAL@DE
Sample Name	20230404-CXM-C42	Instrument	compact 8255754.20127
Comment			

Acquisition Parameter

Source Type	ESI	Ion Polarity	Positive	Set Nebulizer	2.0 Bar
Focus	Active	Set Capillary	4500 V	Set Dry Heater	200 °C
Scan Begin	50 m/z	Set End Plate Offset	-500 V	Set Dry Gas	8.0 l/min
Scan End	3000 m/z	Set Charging Voltage	2000 V	Set Divert Valve	Waste
		Set Corona	0 nA	Set APCI Heater	0 °C

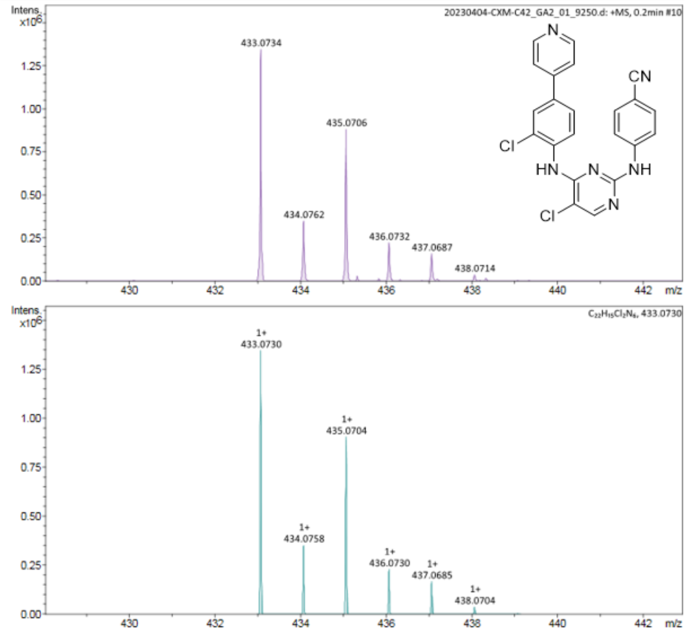

20230404-CXM-C42_GA2_01_9250.d

Bruker Compass DataAnalysis 4.2 printed: 4/9/2023 6:14:46 PM by: BDAL@DE Page 1 of 1

HPLC

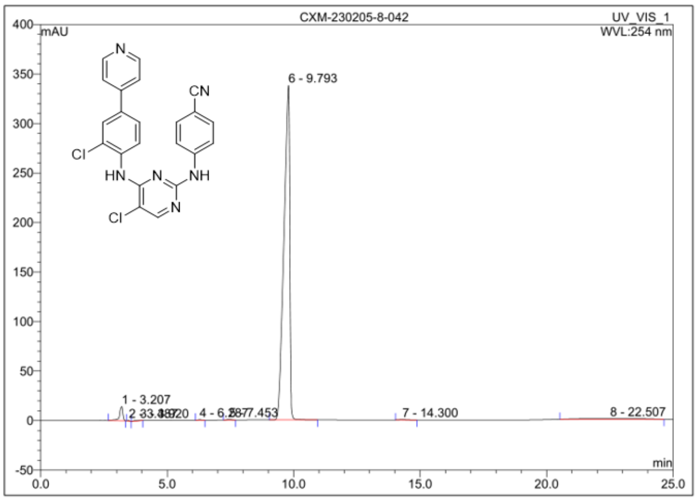

No.	Ret.Time min	Peak Name	Height mAU	Area mAU*s	Rel.Area %	Amount	Type
1	3.21	n.a.	14.432	131.35	2.32	n.a.	BMB*
2	3.49	n.a.	0.620	3.75	0.07	n.a.	BMB*
3	3.92	n.a.	0.281	9.47	0.17	n.a.	BMB*
4	6.29	n.a.	0.447	4.03	0.07	n.a.	BMB*
5	7.45	n.a.	0.636	7.62	0.13	n.a.	BMB*
6	9.79	n.a.	337.785	5392.19	95.21	n.a.	BMB*
7	14.30	n.a.	0.285	5.68	0.10	n.a.	BMB*
8	22.51	n.a.	0.558	109.62	1.94	n.a.	BMB*
Total:			355.044	5663.706	100.00	0.000	

^{1}H NMR, ^{13}C NMR, HRMS, HPLC spectra of B19

^{1}H NMR

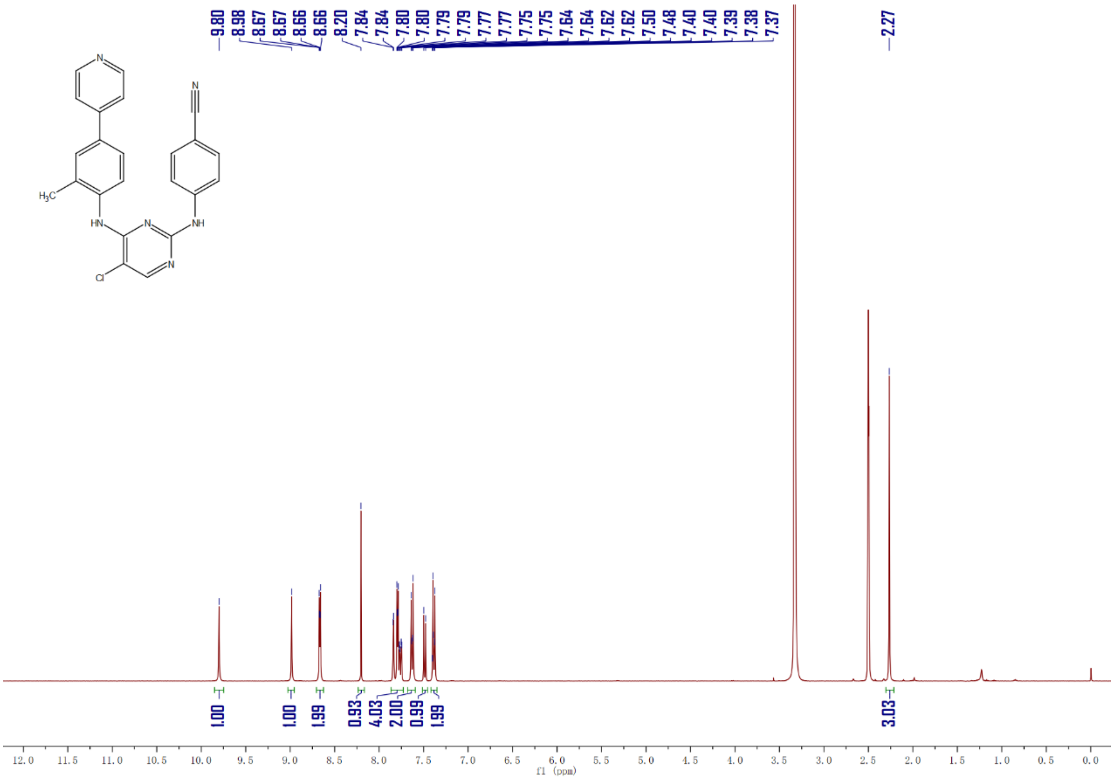

^{13}C NMR

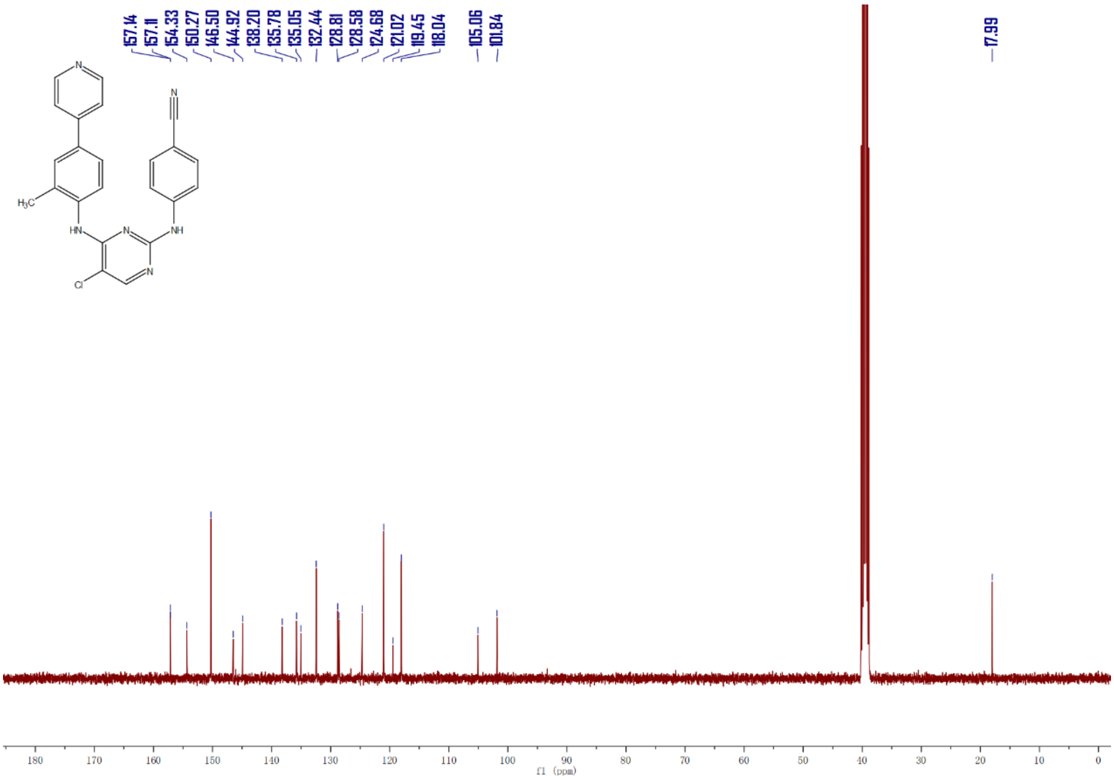

HRMS

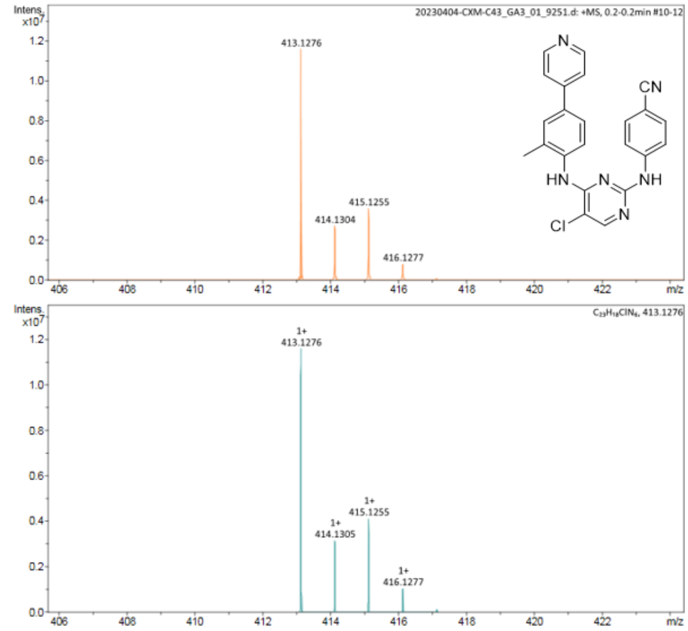

Display Report

Analysis Info

Analysis Name	D:\CXM\20230404\20230404-CXM-C43_GA3_01_9251.d	Acquisition Date	4/4/2023 6:14:34 PM
Method	MS-2MIN-POS.m	Operator	BDAL@DE
Sample Name	20230404-CXM-C43	Instrument	compact 8255754.20127
Comment			

Acquisition Parameter

Source Type	ESI	Ion Polarity	Positive	Set Nebulizer	2.0 Bar
Focus	Active	Set Capillary	4500 V	Set Dry Heater	200 °C
Scan Begin	50 m/z	Set End Plate Offset	-500 V	Set Dry Gas	8.0 l/min
Scan End	3000 m/z	Set Charging Voltage	2000 V	Set Divert Valve	Waste
		Set Corona	0 nA	Set APCI Heater	0 °C

20230404-CXM-C43_GA3_01_9251.d
Bruker Compass DataAnalysis 4.2 printed: 4/9/2023 6:18:05 PM by: BDAL@DE Page 1 of 1

HPLC

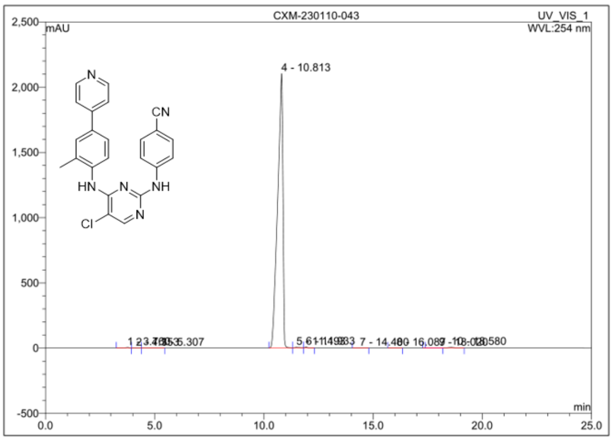

No.	Ret.Time min	Peak Name	Height mAU	Area mAU*s	Rel.Area %	Amount	Type
1	3.76	n.a.	5.161	69.30	0.20	n.a.	BMB
2	4.15	n.a.	0.583	12.41	0.04	n.a.	BM
3	5.31	n.a.	0.157	15.16	0.04	n.a.	MB
4	10.81	n.a.	2103.293	35005.28	98.96	n.a.	BM
5	11.49	n.a.	3.941	75.04	0.21	n.a.	M
6	11.93	n.a.	6.374	53.31	0.15	n.a.	MB
7	14.40	n.a.	0.570	12.44	0.04	n.a.	BMB
8	16.09	n.a.	0.342	6.50	0.02	n.a.	BMB
9	18.02	n.a.	0.195	6.65	0.02	n.a.	Ru
10	18.58	n.a.	4.739	116.41	0.33	n.a.	BMB*
Total:			2125.356	35372.506	100.00	0.000	

^{1}H NMR, ^{13}C NMR, HRMS, HPLC spectra of B20

^{1}H NMR

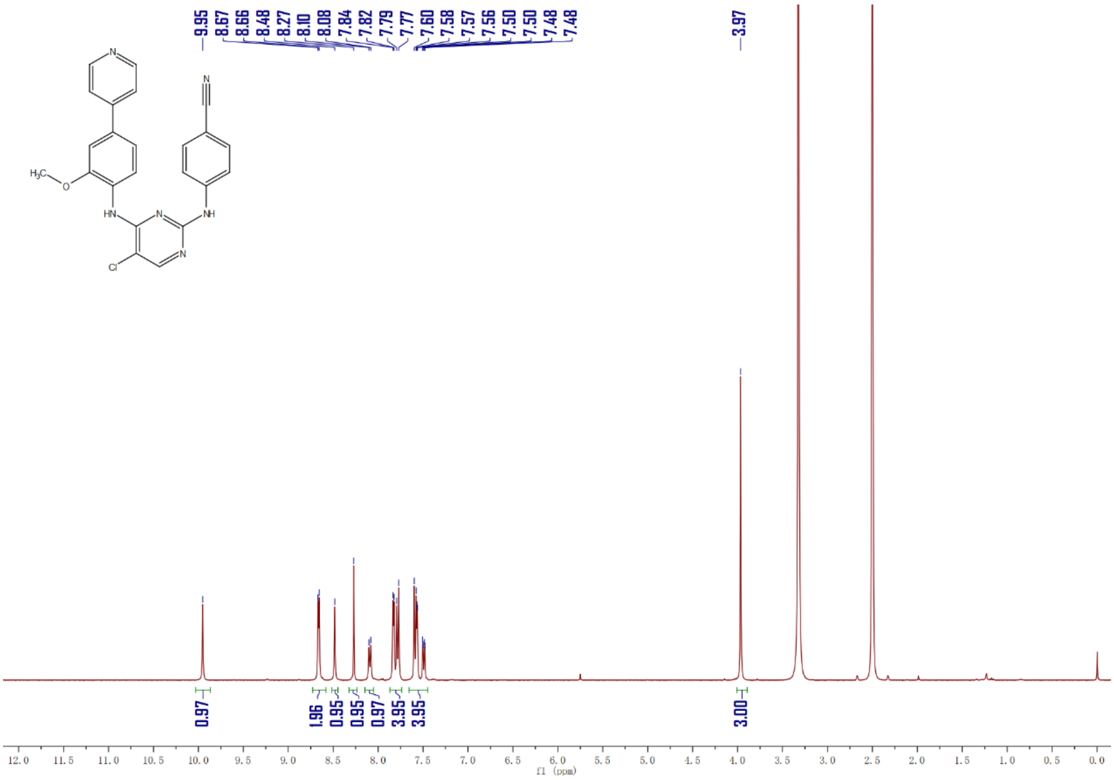

^{13}C NMR

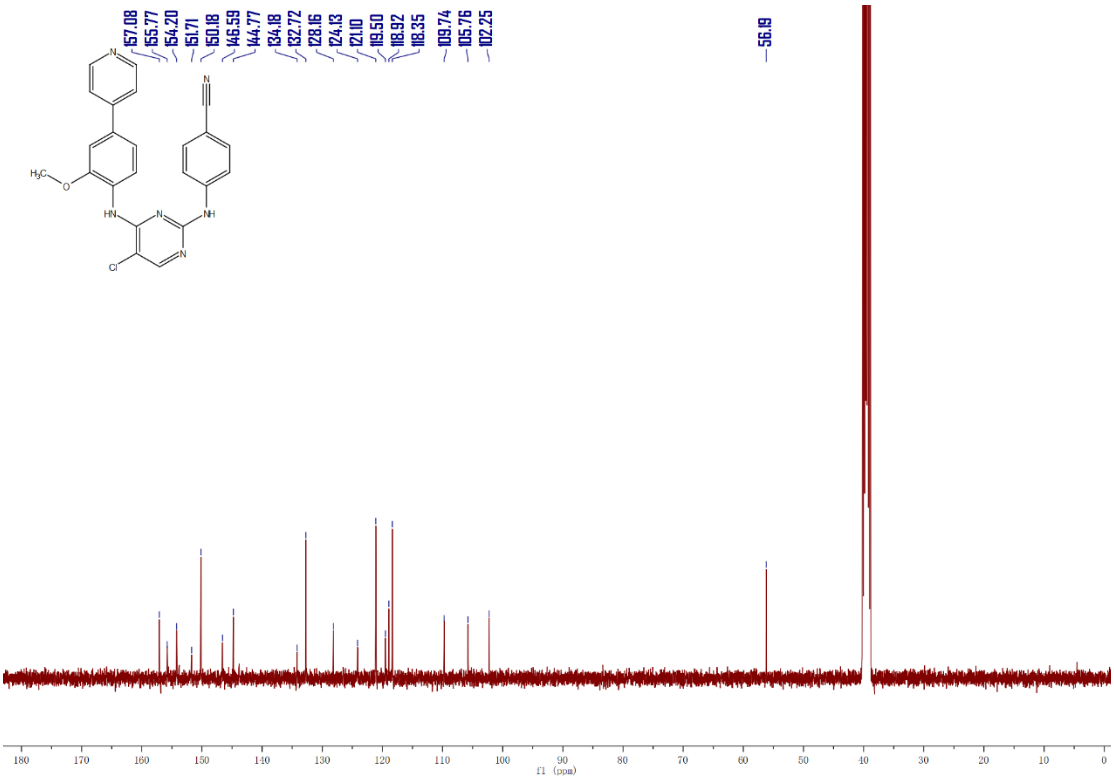

HRMS

Display Report

Analysis Info

Analysis Name	D:\CXM\20230404\20230404-CXM-C44_GA4_01_9252.d	Acquisition Date	4/4/2023 6:17:18 PM
Method	MS-2MIN-POS.m	Operator	BDAL@DE
Sample Name	20230404-CXM-C44	Instrument	compact 8255754.20127
Comment			

Acquisition Parameter

Source Type	ESI	Ion Polarity	Positive	Set Nebulizer	2.0 Bar
Focus	Active	Set Capillary	4500 V	Set Dry Heater	200 °C
Scan Begin	50 m/z	Set End Plate Offset	-500 V	Set Dry Gas	8.0 l/min
Scan End	3000 m/z	Set Charging Voltage	2000 V	Set Divert Valve	Waste
		Set Corona	0 nA	Set APCI Heater	0 °C

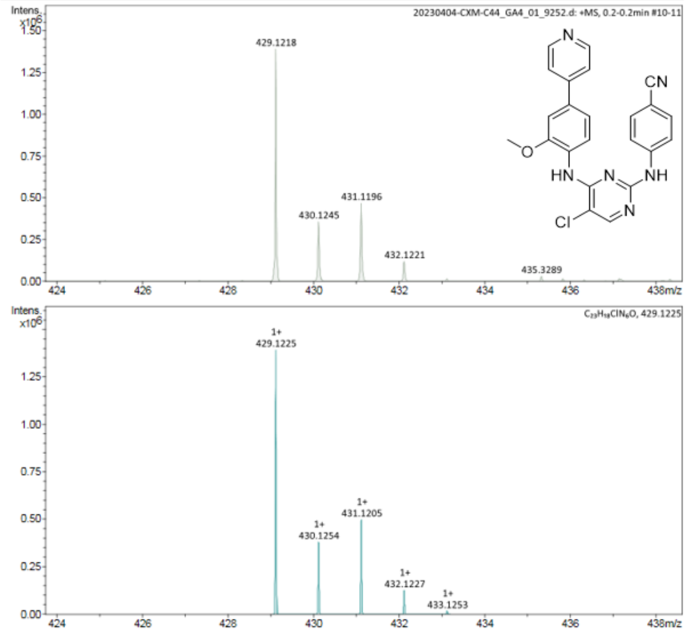

20230404-CXM-C44_GA4_01_9252.d
Bruker Compass DataAnalysis 4.2 printed: 4/9/2023 6:20:13 PM by: BDAL@DE Page 1 of 1

HPLC

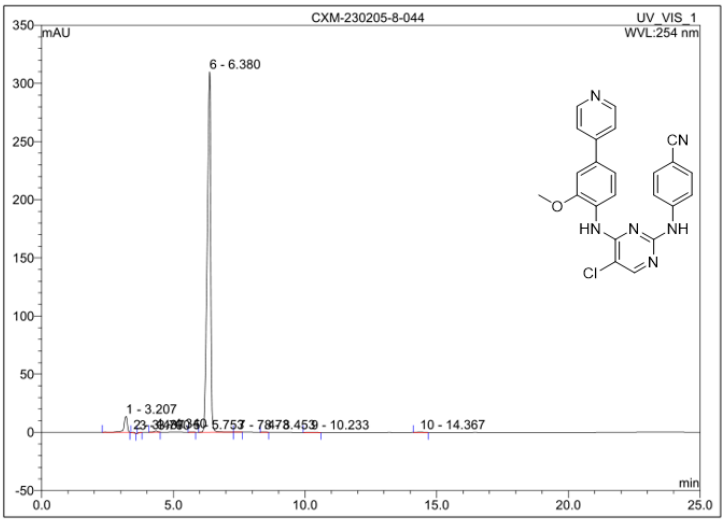

No.	Ret.Time min	Peak Name	Height mAU	Area mAU*s	Rel.Area %	Amount	Type
1	3.21	n.a.	13.716	119.87	4.03	n.a.	BMB*
2	3.49	n.a.	0.647	4.47	0.15	n.a.	BMB*
3	3.70	n.a.	0.408	2.79	0.09	n.a.	BMB*
4	4.34	n.a.	1.114	9.38	0.32	n.a.	BMB*
5	5.75	n.a.	0.019	0.19	0.01	n.a.	BMB*
6	6.38	n.a.	309.867	2827.07	95.06	n.a.	BMB*
7	7.47	n.a.	0.051	0.52	0.02	n.a.	BMB*
8	8.45	n.a.	0.173	1.62	0.05	n.a.	BMB*
9	10.23	n.a.	0.184	4.03	0.14	n.a.	BMB*
10	14.37	n.a.	0.220	4.20	0.14	n.a.	BMB*
Total:			326.399	2974.133	100.00	0.000	

^{1}H NMR, ^{13}C NMR, ^{19}F NMR, HRMS, HPLC spectra of B21

^{1}H NMR

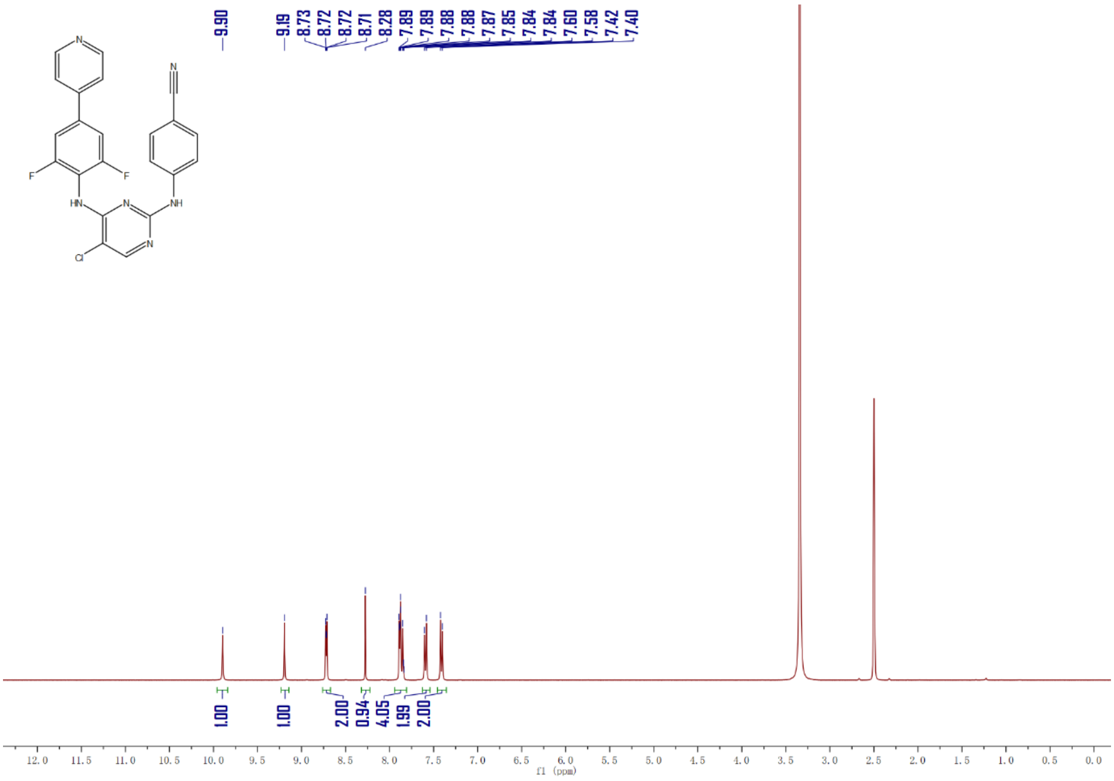

^{13}C NMR

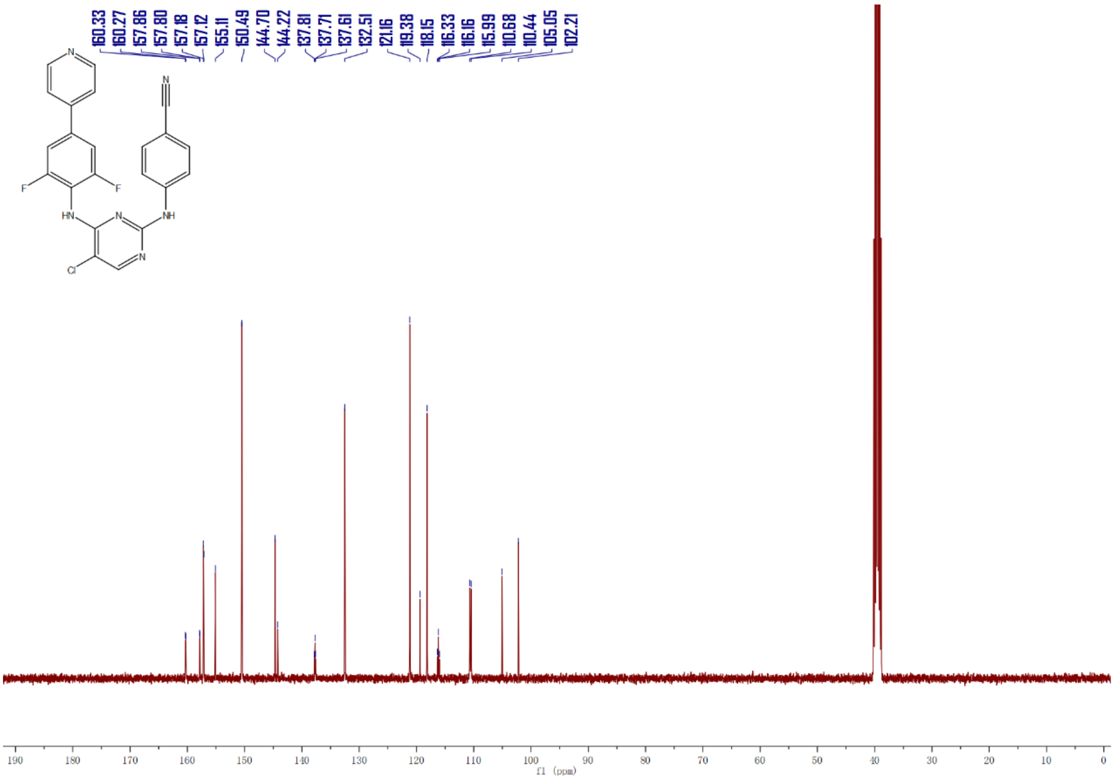

^{19}F NMR

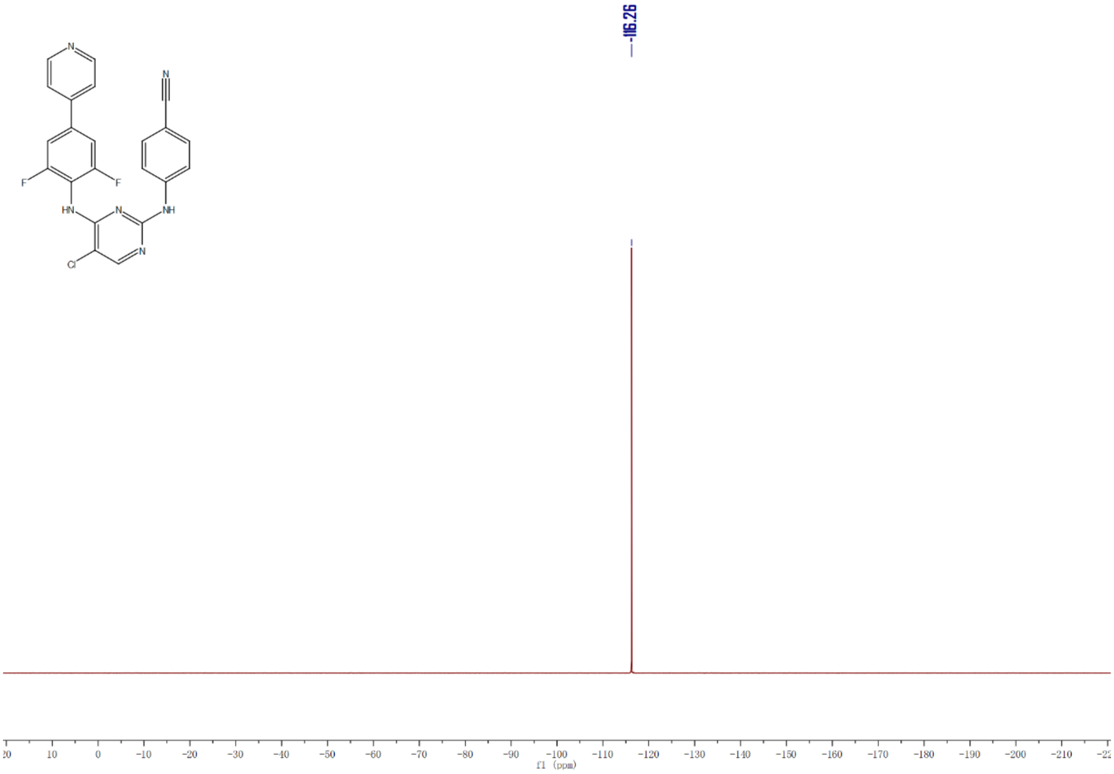

HRMS

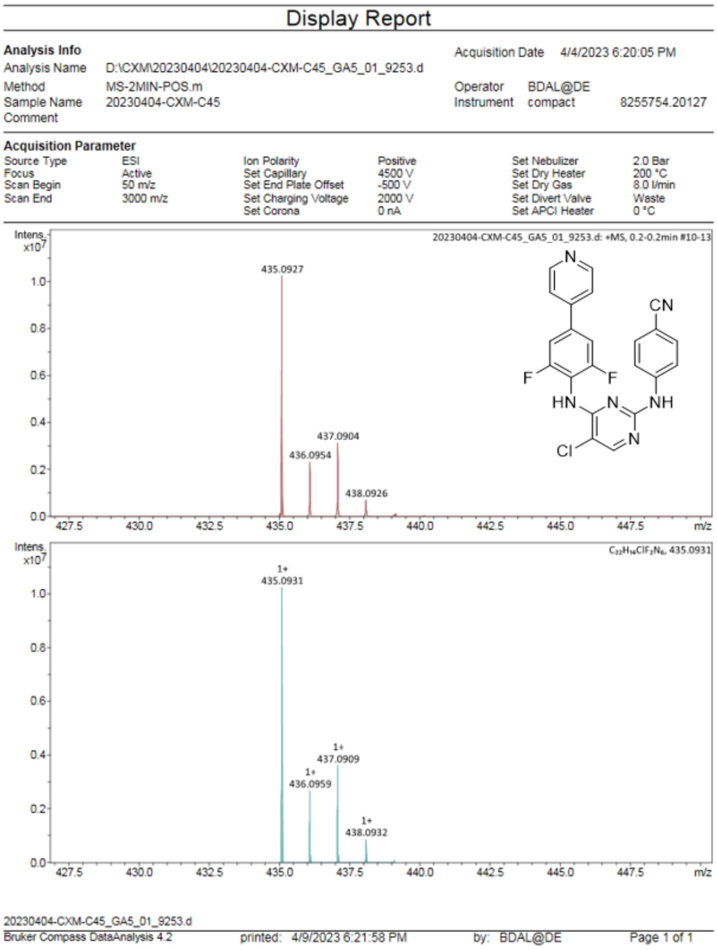

HPLC

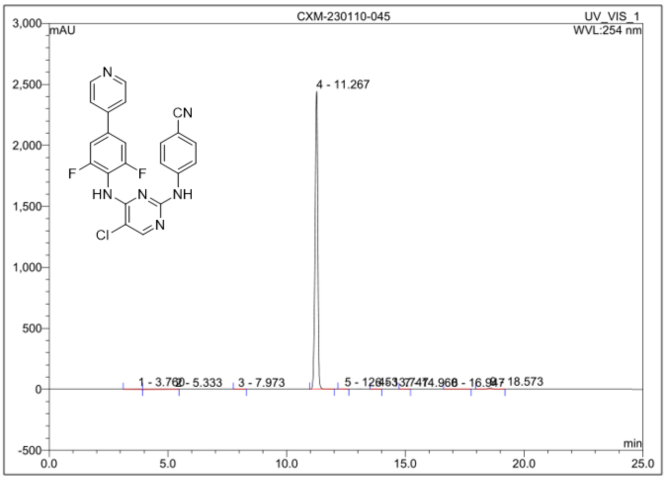

No.	Ret.Time min	Peak Name	Height mAU	Area mAU*s	Rel.Area %	Amount	Type
1	3.76	n.a.	3.272	43.14	0.22	n.a.	BMB
2	5.33	n.a.	0.125	21.84	0.11	n.a.	BMB
3	7.97	n.a.	0.840	7.55	0.04	n.a.	BMB
4	11.27	n.a.	2442.330	19424.82	98.64	n.a.	BMB
5	12.45	n.a.	2.165	18.08	0.09	n.a.	BMB
6	13.75	n.a.	1.113	12.35	0.06	n.a.	BMB
7	14.96	n.a.	0.415	4.99	0.03	n.a.	BMB
8	16.95	n.a.	0.324	8.49	0.04	n.a.	BMB
9	18.57	n.a.	7.802	152.06	0.77	n.a.	BMB
Total:			2458.386	19693.306	100.00	0.000	

^{1}H NMR, ^{13}C NMR, HRMS, HPLC spectra of B22

^{1}H NMR

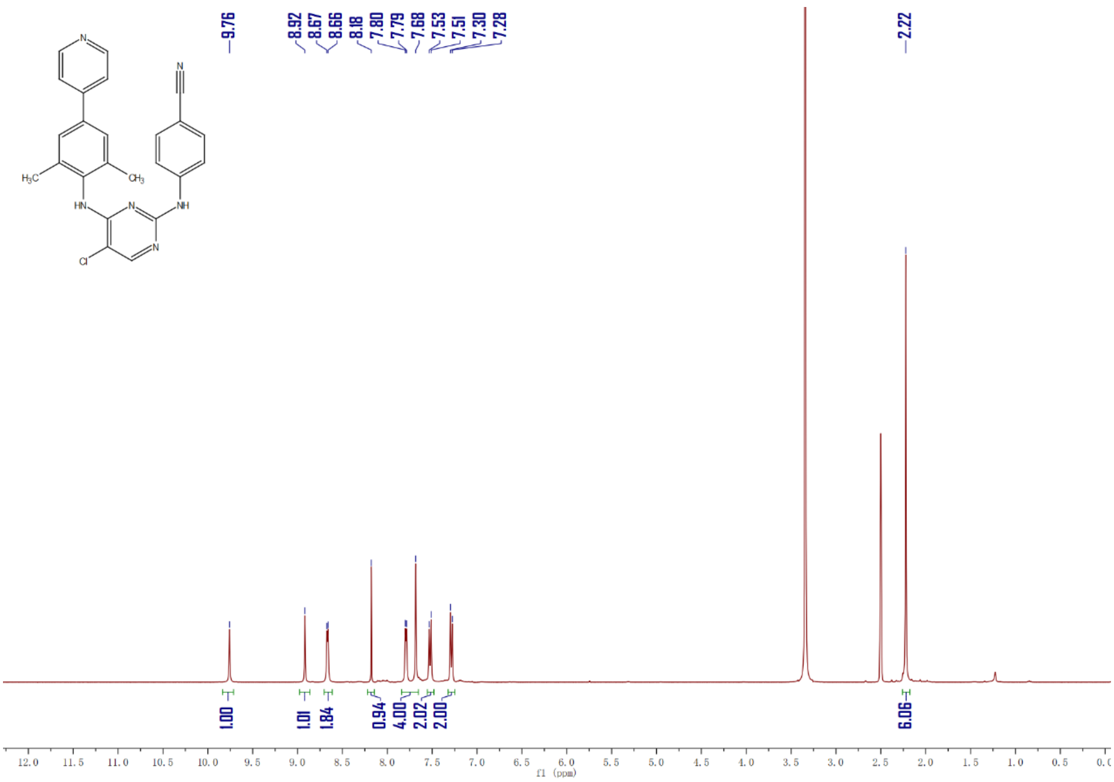

^{13}C NMR

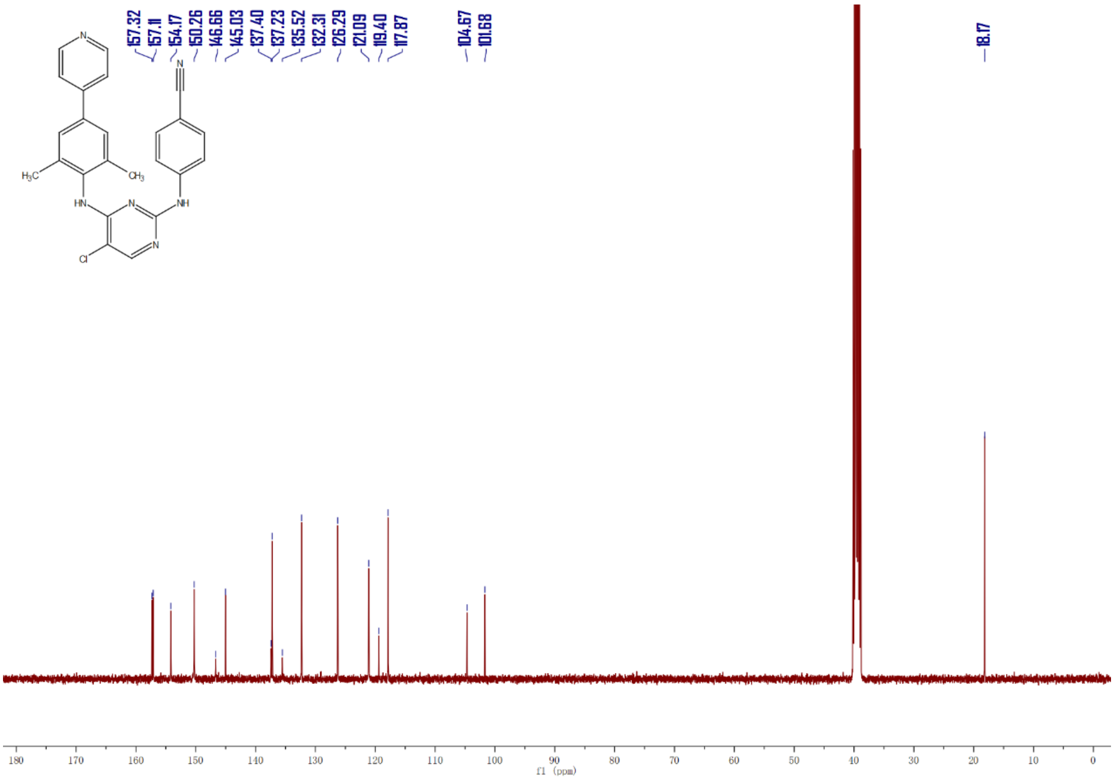

HRMS

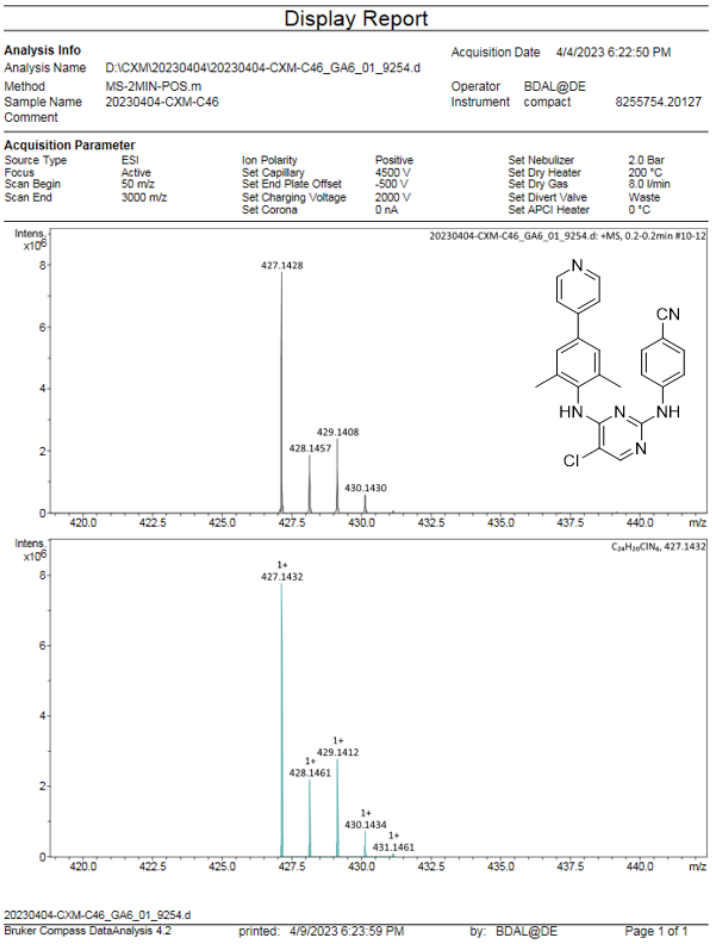

HPLC

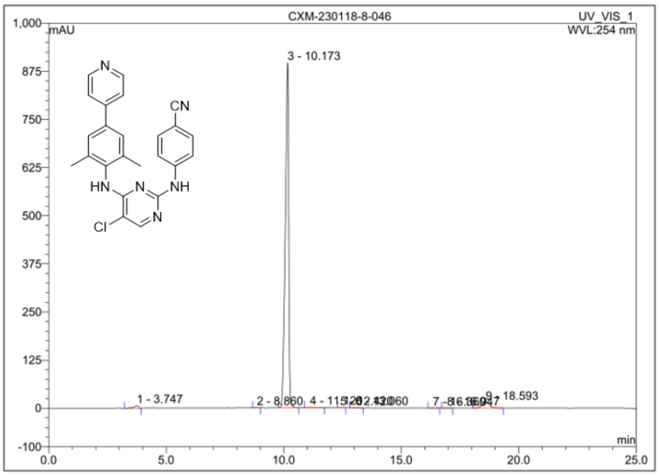

No.	Ret.Time min	Peak Name	Height mAU	Area mAU*s	Rel.Area %	Amount	Type
1	3.75	n.a.	6.442	83.40	0.87	n.a.	BMB*
2	8.86	n.a.	1.112	9.24	0.10	n.a.	BMB
3	10.17	n.a.	896.571	9089.36	95.03	n.a.	BMB
4	11.12	n.a.	1.231	17.09	0.18	n.a.	BMB
5	12.42	n.a.	0.632	5.61	0.06	n.a.	BMB
6	13.06	n.a.	1.437	18.65	0.19	n.a.	BMB
7	16.36	n.a.	0.198	3.20	0.03	n.a.	BMB*
8	16.95	n.a.	0.287	4.39	0.05	n.a.	BMB*
9	18.59	n.a.	15.216	334.27	3.49	n.a.	BMB
Total:			923.126	9565.222	100.00	0.000	
